# Newly developed Pd/ZU-Phos/DBN cooperative catalysis enables enantioselective [4+2] annulations for efficient synthesis of diverse spirocyclic scaffolds

**DOI:** 10.1093/nsr/nwaf443

**Published:** 2025-10-20

**Authors:** Linlin Shi, Mengyan Guo, Mengya Xu, Er-Qing Li, Junbiao Chang, Bin Yu

**Affiliations:** College of Chemistry, State Key Laboratory of Antiviral Drugs, Pingyuan Laboratory, Zhengzhou University, Zhengzhou 450001, China; College of Chemistry, State Key Laboratory of Antiviral Drugs, Pingyuan Laboratory, Zhengzhou University, Zhengzhou 450001, China; College of Chemistry, State Key Laboratory of Antiviral Drugs, Pingyuan Laboratory, Zhengzhou University, Zhengzhou 450001, China; College of Chemistry, State Key Laboratory of Antiviral Drugs, Pingyuan Laboratory, Zhengzhou University, Zhengzhou 450001, China; College of Chemistry, State Key Laboratory of Antiviral Drugs, Pingyuan Laboratory, Zhengzhou University, Zhengzhou 450001, China; College of Chemistry, State Key Laboratory of Antiviral Drugs, Pingyuan Laboratory, Zhengzhou University, Zhengzhou 450001, China; State Key Laboratory of Metabolic Dysregulation & Prevention and Treatment of Esophageal Cancer, Tianjian Laboratory of Advanced Biomedical Sciences, Institute of Advanced Biomedical Sciences, Zhengzhou University, Zhengzhou 450001, China

**Keywords:** ligand system, cooperative catalysis, asymmetric catalysis, diverse spirocyclic scaffolds

## Abstract

Chiral ligands are essential for asymmetric catalysis, as they greatly improve the chemical selectivity of the catalytic process. The cooperative action of multiple ligands may enhance catalytic efficiency and selectivity by optimizing individual steps within the catalytic cycle. Here, we synthesized a series of novel P-chiral ligands (**ZU-Phos**) that enabled highly efficient asymmetric [4+2] annulations of 1,2-disubstituted allylic carbonates with activated olefins, yielding a variety of optically active and medicinally relevant spirocyclic scaffolds in yields of ≤96% and with ≤98% ee through a novel dual coordination mechanism. Mechanistic investigations disclosed that 1,5-diazabicyclo[4.3.0]non-5-ene (DBN) served a dual function as a Brønsted base and formed a unique mixed ligated Pd/**ZU-Phos**/DBN complex. This study highlights the distinctive benefits of Pd/**ZU-Phos** ligand catalysis and underscores the efficacy of the dual-ligand system in broadening the scope of palladium-catalysed reactions. Density functional theory calculations showcase that DBN modulated the catalytic activity of the palladium catalysts via weak coordination and enhanced product enantioselectivity by stabilizing the chiral environment through weak interactions with phosphorus ligands.

## Teaser

New Pd/WQ-Phos/DBN complex enables asymmetric [4+2] annulations, yielding optically active and biologically relevant spiro scaffolds with up to 96% yield and 98% ee.

## INTRODUCTION

**Figure 1.**
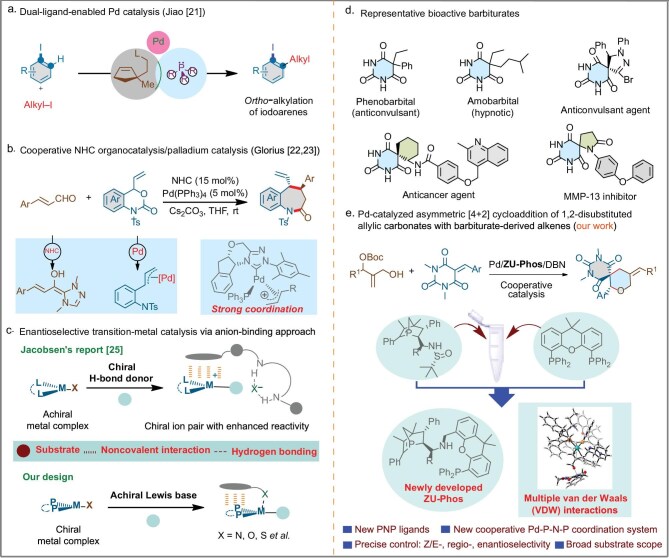
Background and strategy. (a) Dual-ligand-enabled Pd catalysis. (b) Cooperative NHC organocatalysis/palladium catalytic system. (c) Enantioselective transition-metal catalysis via an anion-binding approach and our design. (d) Representative bioactive barbiturates. (e) Our proposed new cooperative palladium/**ZU-Phos**/DBN coordination system for asymmetric cycloadditions of 1,2-substituted allylic carbonates for efficient access to the spirocyclic scaffolds.

In the field of chemical synthesis, asymmetric catalysis has always been a hotspot and challenge for scientists as an efficient means of obtaining chiral molecules. Traditional asymmetric catalytic systems often rely on the coordination of a single chiral ligand with the metal center to achieve selective control over chiral products [1–5]. However, this single-ligand mode may encounter challenges such as geometric constraints in coordination and poor stereochemical communication in certain cases [6]. Based on this, a dual/multiple-catalysis system has been developed to overcome the challenge; however, this strategy frequently encounters challenges in the compatibility of different catalysts and substrate scope limitations [7–9]. Recently, the strategy of dual-ligand coordination in asymmetric metal catalysis has been gradually emerging as a brilliant new star in the realm of asymmetric catalysis due to its unique innovativeness and significance [10–14]. The core of this strategy lies in the synergistic coordination of two or more ligands with the metal center, which not only optimizes the geometric and electronic structures of the catalyst, but also significantly enhances the selectivity and efficiency of the catalytic reaction, thereby opening up new avenues for the synthesis of complex chiral molecules. By introducing a second ligand, it is possible to adjust the coordination environment of the metal center, enhance the activity of the catalyst and achieve fine-tuning of the reaction pathway through interactions between the two ligands [15–20]. For example, by combining Pd/olefin ligand cooperative catalysis with bulky trialkylphosphine ligand-promoted C(sp^2^)-I reductive elimination, the group of Jiao have established an *ortho*-alkylative Catellani-type reaction with the aryl–iodine bond reconstruction as the final step, which opens new synthetic opportunities within Catellani-type reactions (Fig. 1a) [21]. This cooperative coordination effect enables the catalyst to exhibit excellent asymmetric induction capabilities across a broader range of substrates, greatly expanding the application scope of asymmetric catalysis. Nevertheless, enhancing the effectiveness, activity and stereoselectivity of metal catalysts by using two distinct ligands with the same metal in a mixed coordination mode remains a big challenge in the asymmetric field. The key challenge lies in studying the precise coordination between the metal and the two ligands, preventing ligand interference and quenching the active centers of the metal catalyst. A significant breakthrough in this field was made by the group of Glorius by using a mixed ligand palladium/PPh_3_/chiral *N*-heterocyclic carbene (NHC) complex to realize the asymmetric [4+3] annulation reaction of vinyl benzoxazinones and enals, providing the corresponding chiral benzazepines in good yields with high enantioselectivities (Fig. 1b) [22,23]. In 2021, Gong and co-workers developed a switchable interaction strategy between NHCs and a copper complex, realizing mixed ligated copper/chiral phosphine/chiral NHC complex catalysed [3+3] annulations of *N*-tosylaziridines with isatin-derived enals [24]. However, achievements in this field predominantly stem from mixed metal/NHC/phosphine dual catalytic systems that rely on strong coordination. This means that, although various mixed coordination modes are being increasingly used in a variety of asymmetric transformations, these methods have not reached the same level of sophistication.

The introduction of weak coordination modes in a dual-ligand cooperative asymmetric metal–organic catalytic system adds new vitality to this strategy. Weak ligands typically do not form strong coordination bonds with the metal center, but maintain a certain connection with the metal complex through non-covalent interactions (such as hydrogen bonds, *π*–*π* stacking, etc.). This weak coordination mode not only preserves the active sites of the metal center, facilitating substrate approach and reaction, but also exerts a profound influence on the selectivity and rate of the catalytic reaction through subtle electronic and steric effects. Compared with traditional strong coordination modes, weak coordination modes provide sufficient stability while endowing the catalyst with higher flexibility and adaptability, offering possibilities for the precise control of complex asymmetric catalytic reactions. In 2023, Jacobsen and co-workers reported a cooperative anion-binding effect of chiral bis-thiourea neutral hydrogen-bond donors (HBDs) to lead to high enantioselectivity in intramolecular ruthenium-catalysed propargylic substitution reactions, demonstrating the attractive interactions between electron-deficient arene components of the HBD and the metal complex that underlie enantioinduction and the acceleration effect (Fig. 1c) [25]. Encouraged by this, we envisage that metal/phosphine complexes, as a class of highly enantioselective catalysts, have been extensively applied in asymmetric transformations to efficiently generate chiral structures. Nitrogen ligands, due to their stronger basicity and weaker nucleophilicity compared with phosphine ligands, typically exhibit unique reactivity and selectivity in asymmetric metal catalysis [26,27]. Considering these facts, incorporating nitrogen ligands into metal/phosphine complexes could alter the spatial and electronic characteristics of the metal catalysts, thereby further enhancing specialized catalytic functions and achieving unprecedented stereocontrol—a feat not attainable with either metal/nitrogen or metal/phosphorus ligands alone. As of now, a mixed coordinated system involving palladium/nitrogen ligand/phosphine ligand by weak coordination modes has not been reported [28]. The compatibility issue between nitrogen and phosphine ligands in the same coordination system remains unresolved. Hence, developing new ligands to realize novel hybrid ligated systems continues to be a challenging task.

Spirobarbiturates are well known as an important class of central nervous system depressants that are widely used as sedatives, anesthetic, anxiolytic and anticonvulsant agents (Fig. 1d) [29–32]. In this context, the development of straightforward methods for the synthesis of these chiral molecules is highly desirable. The Pd-catalysed allyl annulation of zwitterion synthons has emerged as a promising method for the synthesis of optically pure compounds [33–37]. Among them, allylic carbonates, developed by Trost and other groups [38], have been extensively investigated. However, 1,2-disubstituted allylic carbonates are still relatively underexplored due to unresolved difficulties in three aspects [39,40]: (i) the energy difference between the *syn-* and *anti*-intermediates of 1,2-disubstituted π-allylpalladium complexes decreases due to steric repulsion between the R^1^ and R^2^ groups; (ii) the lower reactivity of the 1,2-substituted allylic precursors makes it more challenging to achieve high efficiency and enantioselectivity; (iii) the regioselectivity of the three reactive sites on the π-allylpalladium intermediate and the *Z*/*E* control of the linear trisubstituted allylic products are not yet well resolved. To address the above challenges, here, we have developed a new type of phosphine-nitrogen-phosphine (PNP) ligand (**ZU-Phos**) that works effectively in a novel mixed dual coordination mode for the highly stereoselective synthesis of various biologically relevant and optically active spirobarbiturates (Fig. 1e).

## RESULTS AND DISCUSSION

**Figure 2.**
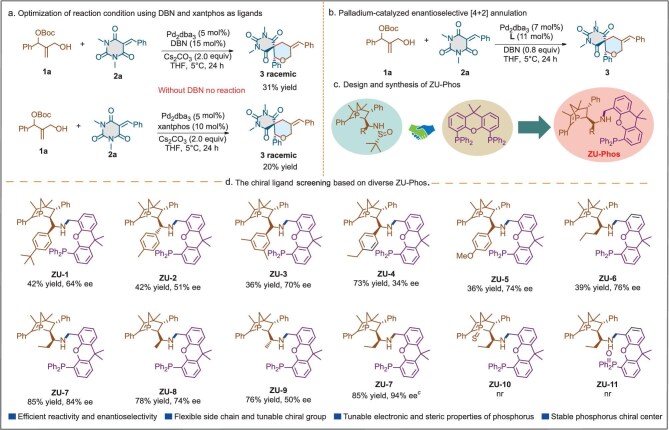
Design and optimization of the **ZU-Phos** ligands. (a) Optimization of reaction conditions using DBN and xantphos as ligands. (b) Palladium-catalysed enantioselective [4+2] annulation: conditions of all reactions: **1a** (0.1 mmol), **2a** (0.2 mmol), Pd_2_dba_3_ (7 mol%), **L** (11 mol%), Cs_2_CO_3_ (2.0 equiv.) THF (1 mL), N_2_, 5°C, 48 h; isolated yield. nr: no reaction (c) Design and synthesis of **ZU-Phos**. (d) Chiral ligand screening based on diverse **ZU-Phos**.

In 2021, Chen and co-workers reported palladium-catalysed [1+2]/[3+2] annulations based on palladium-trimethylenemethane 1,3-dipoles via a deprotonation strategy. In this report, the authors found that the allyl precursor showed very low reactivity and stereoselectivity by using diverse chiral ligands, probably due to steric reasons [41]. Encouraged by the advantage of dual ligated modes, we anticipated overcoming the ongoing challenge by using a dual-ligand mixed coordination mode, achieving high enantioselective transformation. Initially, we chose barbiturate-derived alkene **1a** and *tert*-butyl (2-(hydroxymethyl)-1-phenylallyl) carbonate **2a** as the model substrate to test different phosphine ligands (PPh_3_, X-Phos, dppe, deppf, etc.) and nitrogen ligands (2,2′-bipyridine, 1,10-phenanthroline, 1,5-diazabicyclo[4.3.0]non-5-ene (DBN), etc.) and found that only Pd/xantphos complexes and the Pd/DBN complex could trigger the transformation, affording the desired racemic product **3** in 20% yield and 31% yield, respectively (Fig. 2a). Inspired by this finding, we chose some well-known commercially available chiral ligands and a series of *P*-chiral 1-phosphanorbornene ligands (**ZD-Phos**) developed by us (including **Gan-Phos** [42], **Jia-Phos** [43], **Yue-Phos** [44] and **Meng-Phos** [45]) with xantphos or DBN to form a Pd/xantphos/chiral ligand or Pd/DBN/chiral ligand mixed coordination system and examined the chiral version [46]. Unfortunately, the reaction did not give the desired product in all cases (see Table S1) (Fig. 2b). We envisaged that, as the DBN could trigger the transformation via the change in Lewis acidity of the palladium catalyst through subtle weak interactions with the metal center, higher activity and enantioselectivity could be obtained by improving the skeletons and steric hindrance of the ligands. Thus, a new chiral ligand needs to be developed to address these challenges.

Based on this, we synthesized a novel chiral ligand, **ZU-Phos**, incorporating a xantphos fragment and a 1-phosphonorbornene motif, which facilitated the allyl cyclization of barbiturate-derived alkene **1a** and *tert*-butyl (2-(hydroxymethyl)-1-phenylallyl) carbonate **2a** via forming a mixed dual ligated system. This new ligand offers several advantages: (i) stable phosphorus chiral center; (ii) two phosphorus moieties with varying electronic and steric properties; (iii) flexible side chains; (iv) adjustable chiral groups (for the synthesis of **ZU-Phos**, see Page S2) (Fig. 2c). Compared with the existing methods for synthesizing similar spiro compounds, our approach utilizing the **ZU-Phos** ligand in a mixed dual coordination mode offers distinct advantages. It achieves superior enantioselectivity and yield under milder reaction conditions, addressing the challenges of low reactivity and regioselectivity in 1,2-disubstituted allylic systems. Additionally, the cost-effectiveness of our method is enhanced by the use of readily available starting materials and the avoidance of complex catalyst systems, making it a promising alternative for the synthesis of optically active spirobarbiturates.

To further examine the effectiveness of the Pd/**ZU-Phos**/DBN complex, the Pd/**ZU-1** complex was first used; the desired product **3** was obtained in 42% yield and with 64% ee (Fig. 2d). Next, various **ZU-Phos** ligands with different R substituent groups were designed and screened, and we found that the R substituent and its stereochemistry had a varied effect on the stereoselectivity control. **ZU-7** (R = Et) showed superior stereoselectivity control in this reaction to deliver **3** in 85% yield and with 84% ee. For further improving the enantioselectivity, various bases, palladium sources, solvents and temperatures were investigated and we found that the Pd_2_dba_3_/**ZU-7** complex as the catalyst and DBN as the base in tetrahydrofuran (THF) at 5°C for 24 h delivered the desired product **3** in 85% yield and with 84% ee (see Tables S1–S9). When **Yue-Phos** and **GF-Phos** (previously developed by our group and Prof. Zhang [47,48]) were used in the reaction, no desired product **3** was obtained, demonstrating the essential role of the new *P*-chiral ligand **ZU-Phos** in the reaction. To our satisfaction, the concurrent addition of the palladium source, **ZU-Phos** and DBN to the reaction system yielded compound **3** in 85% yield and with 93% ee, demonstrating enhanced enantioselectivity. These results confirmed the efficacy of the mixed Pd/**ZU-Phos**/DBN system in achieving high yields and stereoselectivity, with DBN playing a pivotal role. To further explore the roles of the ligand and the base in the Pd-catalysed [4+2] cycloaddition, **ZU-10** and **ZU-11** were used, while no desired product was obtained under the optimized conditions. A ^31^P nuclear magnetic resonance (^31^P NMR) spectrum tracking experiment showed that **ZU-10** and **ZU-11** could effectively coordinate with Pd_2_dba_3_, suggesting that the 1-phosphonorbornene motif and the xantphos motif were indispensable for the Pd-catalysed [4+2] cycloaddition of *tert*-butyl (2-(hydroxymethyl)-1-phenylallyl) carbonate and barbiturate-derived alkene. In addition, a ^31^P NMR tracking experiment on the Pd/**ZU-Phos** complex showed that **ZU-Phos** served as a bidentate ligand (see Figs S2 and S3).

**Figure 3.**
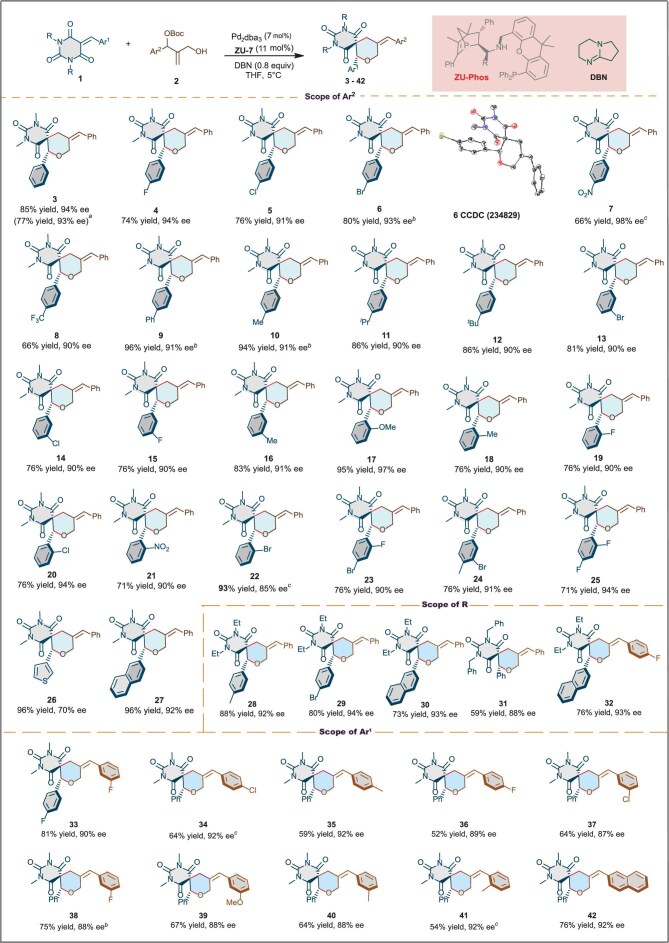
Substrate scope of barbiturate-derived alkenes. Reaction conditions: **1** (0.1 mmol), **2** (0.2 mmol), Pd_2_(dba)_3_ (7 mol%), **ZU-7** (11 mol%), DBN (0.8 equiv.), THF (1 mL), N_2_, 5°C, 24–48 h; isolated yield. *^a^***1** (1 mmol) and **2** (2 mmol) were used. *^b^*Ethyl acetate was used as solvent. *^c^*Pd_2_(dba)_3_ (5 mol%) and **ZU-7** (8 mol%) were used.

With the optimized reaction conditions in hand, we next investigated the substrate scope for a variety of 1,2-disubstituted allylic carbonates **2** (Fig. 3). First, we examined the substituent effect of the aryl groups of barbiturate-derived alkenes on the reactivity and enantioselectivity. Diverse functional groups on the alkene moiety such as an electroneutral group (4-Ph, H), electron-withdrawing groups (4-F, 4-Cl, 4-Br, 4-NO_2_, 4-CF_3_) or electron-donating groups (4-Me, 4-*^i^*Pr, 4-*^t^*Bu) were tolerated competently, affording the corresponding chiral spirobarbituric acid derivatives **3**–**12** in 66%–96% yields and with 90%–98% ee. Next, deviations in the substitution positions on the aromatic rings were also tested; *m*-positions or *o*-positions on the aromatic rings of the barbiturate-derived alkenes offered the desired products **13**–**22** in 71%–95% yields and with 85%–97% ee. The structure and configuration of **6** were unambiguously determined via X-ray diffraction analysis. Even compounds **7, 8** and **21** with strong electron-withdrawing groups (such as ester, NO_2_ or CF_3_) on the phenyl ring were obtained in good yields and with excellent ee values. Disubstituted aryl groups were also well tolerated in this reaction; the desired products **23–25** were afforded in 71%–76% yields and with 90%–94% ee. When the substrate contained a 3-thiethyl, an excellent yield (96%) of **26** was obtained with a lowering ee (70%). The substrate containing a naphthyl motif worked well under optimal conditions to afford the desired product **27** in 96% yield and with 92% ee. The substrate containing an aliphatic motif (cyclopropyl) did not result in a good outcome. We then investigated the scope of the *N*-protecting group (R) of the barbiturate-derived alkenes. The results suggested that the *N*-protecting group played a key role in the reactivity. *N*-ethyl-protected barbiturate-derived alkenes showed higher reactivity; the desired products **28**–**30** and **32** were delivered with satisfactory results (73%–88% yields, 92%–94% ee). *N*-Bn-protected barbiturate-derived alkenes could afford the desired product **31** with 88% ee and in an acceptable 59% yield, while *N*-aryl-protected or *N*-unprotected barbiturate-derived alkenes were not well tolerated in the reaction. The scope of the substituents (Ar^2^) of 1,2-disubstituted allylic carbonates were also investigated; in terms of the electrophiles part, the reactions performed well in all cases and the desired products **33**–**41** were obtained with excellent *Z*/*E, b*/*l* as well as enantioselectivity. For instance, the protocol was applicable to 1,2-disubstituted allylic carbonates **2** bearing various electron-withdrawing groups (3-F, 4-Cl, 4-F, 3-Cl) on the aromatic rings, yielding products in ≤81% isolated yield and with ≤92% ee. Allylic carbonates **2** with electron-donating groups (4-Me, 3-Me, 2-Me, 3-MeO) were also compatible under the standard conditions and could be effectively converted. In addition, substrate **2** with a 2-naphthyl group was well tolerated, affording the desired product **42** in 76% yield and with 92% ee. The large-scale experiment showed the desired product **3** was obtained in 77% yield and with 93% ee, which demonstrated the practicality. Unfortunately, when substrate **2** with aliphatic groups was used, no desired products were obtained. The results demonstrated that our newly developed chiral diphosphate ligand featured excellent chemo-, regio- and enantiocontrol ability.

**Figure 4.**
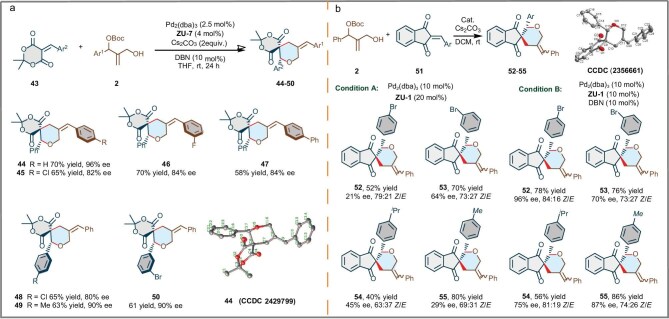
Substrate scope of Meldrum’s acid-derived alkenes and vinylidene ketones. (a) Substrate scope of Meldrum’s acid-derived alkenes: **43** (0.1 mmol), **2** (0.2 mmol), Pd_2_(dba)_3_ (2.5 mol%), **ZU-7** (4 mol%), DBN (10% equiv.), Cs_2_CO_3_ (2.0 equiv.), THF (1 mL), N_2_, rt, 24–48 h; isolated yield. (b) Substrate scope of vinylidene ketones: **51** (0.1 mmol), **2** (0.2 mmol), Pd_2_(dba)_3_ (10 mol%), **ZU-7** (10 mol%), DBN (10% equiv.), Cs_2_CO_3_ (2.0 equiv.), THF (1 mL), N_2_, rt, 24–48 h; isolated yield.

To further explore the utility of this strategy for diversity-oriented synthesis, we examined other trisubstituted olefins derived from biologically active cores for the synthesis of medicinally important spirocyclic scaffolds. The results revealed that both Meldrum’s acid-derived alkenes **43** were highly reactive substrates. It was noted that no reaction occurred in the presence of the Pd_2_(dba)_3_/**ZU-Phos** complex. When Pd_2_(dba)_3_ (7 mol%)/**ZU-7** (11 mol%)/DBN (0.8 equiv.) was used as a catalyst in the reaction, only trace product was observed. To our delight, a satisfactory result (**44** in 70% yield, 96% ee) was obtained by using Pd_2_(dba)_3_ (2.5 mol%)/**ZU-7** (4 mol%)/DBN (10 mol%) as a co-catalyst and Cs_2_CO_3_ (2.0 equiv.) as a base. As indicated in Fig. 4a, the Pd-catalysed [4+2] cycloaddition of various 1,2-disubstituted allylic carbonates **2** and Meldrum’s acid-derived alkenes **43** was performed, giving the corresponding products **44**–**50** in good to excellent yields (58%–70%) with excellent enantioselectivity (80%–96% ee). In addition, further experiments using various different indandione-derived alkenes were performed as outlined in Fig. 4b. When *tert*-butyl (2-(hydroxymethyl)-1-phenylallyl) carbonate **2** and 2-arylidene-1*H*-indene-1,3-(2*H*)-diones **51** were used as substrates in the presence of the Pd_2_dba_3_/**ZU-Phos** complex, 21%–64% ee were obtained. While the mixed Pd_2_dba_3_/**ZU-Phos**/DBN system was used as a co-catalyst, the corresponding products showed significantly improved ee values (70%–96% ee). These results highlight the effectiveness of mixed dual coordination in asymmetric cycloaddition. With these spiro barbituric acid derivatives in hand, we initially evaluated their inhibitory activity against HepG2 cells, demonstrating that compounds **13, 14, 23** and **33** exhibited good tumor cell inhibitory activity, with IC_50_ values ranging from 125 to 750 nM (see Fig. S5). This finding underscores their potential as lead compounds for further development.

To explore the effectiveness of the Pd/**ZU-Phos**/DBN catalytic system, we investigated the effects of the catalyst loading and reaction time on the reactivity and enantioselectivity. The results showed that lowering the catalyst loading resulted in reduced reactivity (see Fig. S4). When 2.5 mol% loading of Pd_2_dba_3_ was used, compound **3** was obtained in only 41% yield and with 93% ee. Extending the reaction time to 96 h resulted in a satisfactory yield of 82% without affecting the enantioselectivity (92% ee). When the loading of catalyst was further reduced to 1 mol%, the desired product **3** was obtained in 21% yield and with 93% ee. When the reaction time was prolonged to 192 h, compound **3** was afforded in 73% yield and with 82% ee. We speculate that the Pd/**ZU-7**/DBN complex was destroyed due to the prolonged reaction time, leading to a decreased ee. This was further confirmed by the ^31^P NMR tracking experiment (see Fig. S1).

**Figure 5.**
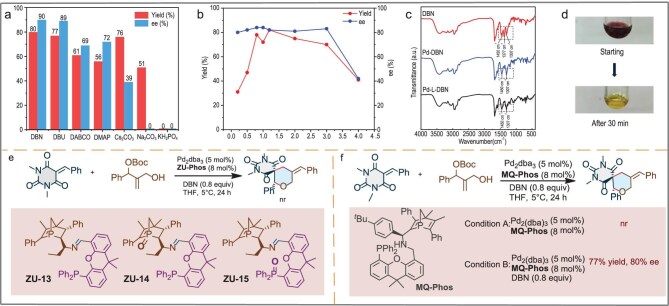
Mechanistic study and control experiment. (a) Screening of bases. (b) Influence of the loading of bases. (c) IR analysis of the C–N stretching vibration of DBN. (d) Color change before and after coordination. (e) Control experiment. (f) Using **MQ-Phos** as chiral ligand in Pd-catalysed [4+2] annulation. nr: no reaction.

As shown in Fig 5a, the base played a key role in the reaction and an organic base (DBN, DBU, DABCO, DMAP) resulted in higher enantioselectivity compared with an inorganic base (Cs_2_CO_3_, Na_2_CO_3_). First, a ^31^P NMR tracking experiment showcased that adding a moderate amount of DBN did not disrupt the coordination between Pd_2_dba_3_ and **ZU-Phos**. Next, the screening of DBN loading showed that lowering the DBN loading resulted in a low yield and slightly decreased ee. When 4.0 equivalent of DBN loading was used, a considerable reduction in the enantioselectivity of **3** was observed. The results suggest that excess DBN could destroy the coordination of Pd_2_dba_3_ with **ZU-Phos** in the reaction (Fig. 5b), which was further verified by the ^31^P NMR tracking experiment (see Fig. S2). This indicated that multiple weak interactions of DBN to the palladium catalyst and **ZU-Phos** were critical in the reaction. According to the infrared diffraction (IR) analysis of the crude reaction mixture, the C–N stretching vibration (1377 cm^−^) of the DBN disappeared, indicating that the interaction of the DBN with the Pd_2_dba_3_ had occurred (Fig. 5c). When Pd_2_dba_3_ and DBN were added to the THF, the color of the reaction mixture changed from fuchsia to bright yellow after 30 min. These results suggest that DBN may coordinate with Pd_2_dba_3_ (Fig. 5d). Next, when **ZU-13** was used, no desired product was obtained under the optimized conditions (Fig. 5e). In addition, when the Pd/**MQ-Phos**/DBN system was used in the reaction, the desired product **3** was obtained in 77% yield and with 80% ee, while no desired product **3** was obtained when using the Pd/**MQ-Phos** complex [49]. We guessed that it was due to the crowded steric hindrance preventing the formation of strong coordination bonds with the metal center, but a certain connection with the metal complex was maintained through non-covalent interactions (such as hydrogen bonds, *π*–*π* stacking, etc.) (Fig. 5f).

**Figure 6.**
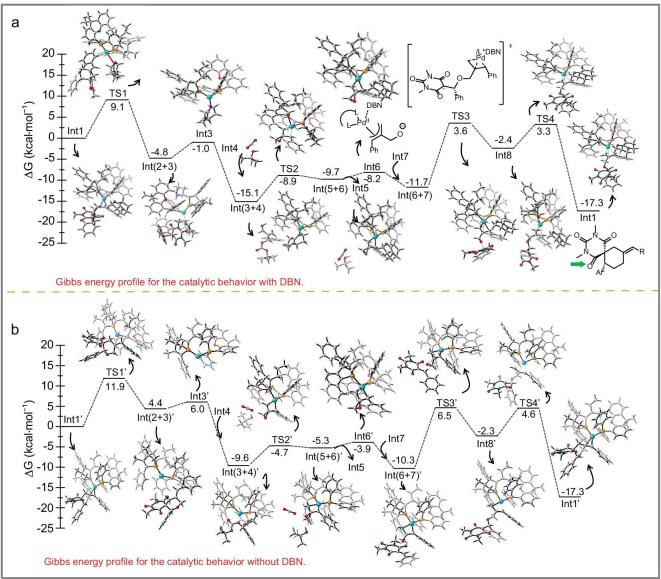
(a) Calculated energy profile of Pd-catalysed asymmetric [4 + 2] annulations with DBN as the co-ligand. (b) Calculated energy profile of Pd-catalysed asymmetric [4 + 2] annulations without DBN.

To investigate the reaction mechanism, we conducted detailed density functional theory (DFT) analysis; the structural diagrams and coordinates are provided in the Supporting information (see Figs S6 and S7) [50]. The reaction is exothermic, releasing a total amount of energy of −17.3 kcal/mol, confirming its thermodynamic feasibility. In the presence of DBN, transition states **TS1**–**TS4** along the reaction pathway exhibit relatively low energy barriers (9.1–15.3 kcal/mol), with **TS3** identified as the rate-determining step. DBN forms multiple van der Waals (VDW) interactions with reactants and the catalytic substrate, stabilizing **TS3** and confirming the kinetic feasibility of the reaction (15.3 kcal/mol, Fig. 6a). Without DBN, transition states **TS1**′–**TS4**′ also show relatively low energy barriers, with **TS3**′ having the highest barrier (15.3 kcal/mol). For the formation of unfavored product, the energy barrier is 16.3 kcal/mol, which is slightly higher than that for favored product, leading to a mixture of configurations of favored product and unfavored product, with the favored product predominating due to its lower barrier, consistently with experimental observations. DBN effectively regulates the product ratio, favoring the chiral unfavored product by hindering the formation of unfavored product (Fig. 6b). Steric hindrance prevents strong coordination between DBN’s nitrogen atom and the palladium metal center, resulting in weak interactions at a chemical distance of 3.92 Å instead of a conventional strong bond (>1.85 Å) [51,52]. Through multiple VDW interactions with the metal and the ligand, DBN enables the efficient construction of the desired spiro compounds with high reactivity and enantioselectivity. This strategy circumvents catalyst-compatibility issues in dual/multicatalytic systems and addresses the limitations of traditional metal catalysis, such as weak stereocontrol in certain reaction systems.

**Figure 7.**
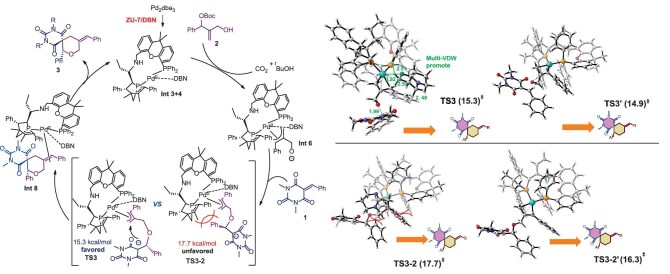
Possible mechanism of palladium-catalysed enantioselective [4 + 2] annulations.

According to the DFT calculation and the experimental result, we proposed a possible mechanism as outlined in Fig. 7. Initially, the active chiral Pd(0) catalyst, derived from the mixed coordination of Pd, **ZU-7** and DBN, facilitated the decarboxylation of *tert*-butyl (2-(hydroxymethyl)-1-phenylallyl) carbonate **2** through oxidative addition, yielding the π-allylpalladium **Int 6**. Next, the nucleophilic oxygen anion of **Int 6** attacked barbiturate-derived alkenes **1** to provide the Pd-coordinated **TS3**. In the presence of DBN, **TS3** (15.3 kcal/mol) showed lower transition-state energy compared with **TS3′** (17.7 kcal/mol), which resulted in excellent enantioselectivity of the desired product **3**. Then, an intramolecular annulation occurred to give **Int 8**. Finally, reductive elimination proceeded to afford the desired product **3** and regenerate the Pd(0) catalyst.

## CONCLUSION

In conclusion, following our previous work on *P*-chiral ligands, we herein have designed a novel type of PNP ligand (**ZU-Phos**) derived from **Jia-Phos** with xantphos. The mixed Pd/**ZU-Phos**/DBN coordination system, as a novel type of chiral catalyst, is effective for the asymmetric [4 + 2] annulations between 1,2-disubstituted allylic carbonates and activated olefins for the synthesis of various biologically relevant and enantioenriched spiral barbituric acid derivatives in good yields and with high ee values. The cooperatively mixed dual coordination system is also applicable to other electron-deficient olefins, giving the desired products with higher enantioselectivity. Interestingly, DBN acts as a Brønsted base and forms a novel mixed ligated Pd/**ZU-Phos**/DBN complex through multiple VDW interactions. The new Pd/**ZU-Phos**/DBN complex not only enhances the enantioselective control of the palladium complex, but also improves its catalytic activity. The mixed Pd/**ZU-Phos**/DBN system outperforms normal Pd/**ZU-Phos** complexes. Overall, compared with known dual catalysis, this new coordination system provides new opportunities for conventionally challenging chemical transformations and allows high levels of enantiocontrol by improving the electrical property and steric hindrance of metal complexes using easily accessible and stable ligands. The mixed ligated system may have potential for other asymmetric transition-metal-catalysed reactions that are currently underway in our laboratory.

## Supplementary Material

nwaf443_Supplemental_File
