# Supplementary material for: Newly developed Pd/ZU-Phos/DBN cooperative catalysis enables enantioselective [4+2] annulations for efficient synthesis of diverse spirocyclic scaffolds
Source: Natl Sci Rev. 2025 Oct 20;13(1):nwaf443. doi: 10.1093/nsr/nwaf443 (PMC12805827; doi:10.1093/nsr/nwaf443)
Supplement: nwaf443_Supplemental_File [file nwaf443_supplemental_file.pdf]

Supporting Information

Newly Developed Pd/ZU-Phos/DBN Cooperative Catalysis Enables Enantioselective [4+2] Annulations for Efficient Synthesis of Diverse Spirocyclic Scaffolds

Linlin Shi, Mengyan Guo, Mengya Xu, Er-Qing Li*, Junbiao Chang* and Bin Yu*

College of Chemistry, State Key Laboratory of Antiviral Drugs, Pingyuan Laboratory, Zhengzhou Uni-versity, Zhengzhou 450001, China.

E-mail: lierqing@zzu.edu.cn; changjunbiao@zzu.edu.cn; yubin@zzu.edu.cn

Contents

1. General information..S1

2. Preparation of starting materials..S2

3. Reaction Optimization..S16

4. General Procedure for Reactions..S22

5. Copies of ^{31}P NMR, ^{1}H NMR, ^{13}C NMR spectra..........................S55

6. Copies of HPLC Chromatograms..S123

7. X-ray crystal structures..S177

8. Biological activity experiments..S181

9. Computational details...S182

1. General information

All reactions were performed under nitrogen atmosphere. Unless otherwise noted, all reagents and solvents were purchased from commercial suppliers and used without further purification. ^{1}H NMR and ^{13}C NMR spectra were recorded at 25 ºC on a Bruker Advance 400 MHz or 300 MHz NMR spectrometers ($CDCl_3$ as solvent). Chemical shifts (δ) are reported in ppm from tetramethylsilane (TMS) with the solvent resonance as the internal standard. Proton signal multiplicities are given as s (singlet), d (doublet), t (triplet), q (quartet), m (multiplet), br (broad) or a combination of them. *J*-values are in Hz. HRMS (ESI-Q-TOF) spectra were recorded on Bruker Impact-IImass spectrometer. IR spectra were recorded on a JASCO FT/IR-4600 Fourier Transform Infrared spectrometer and are reported as cm^{-1}. High-resolution mass spectra (HRMS) were recorded on a JEOL JMS–600 spectrometer. Enantiomer ratios were determined by HPLC (AD-H, IA-H or IG-H columns). Chiral HPLC analysis recorded on Shanghaiyice instruments and Equipment Co. Ltd. and Shimadzu LC-20A. Silica gel (200-300 mesh) was used for the chromatographic separations.

The DFT calculations were performed using the Gaussian16 program[1]. All structures were optimized at the M06-L[2-4]/6-31G(d, p)/SMD_{THF} level, and the corresponding vibrational frequencies were calculated at the same level. Then, frequency calculations at the same level of theory were carried out to identify all of the stationary points as minima (zero imaginary frequency) or transition state (only one frequency), and to provide free energies. The NCI analysis was plotted using Multiwfn[5].

2. Preparation of starting materials

2.1 General procedure for the preparation of chiral ligands

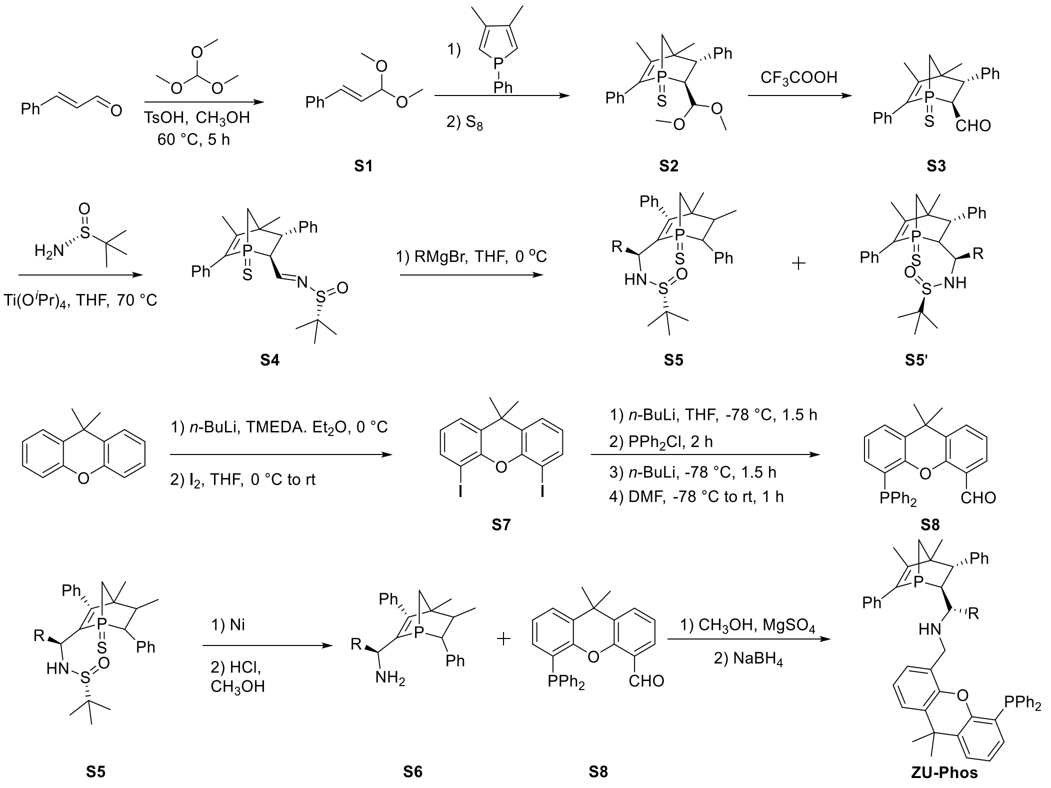

Cinnamaldehyde (13.2 g, 100 mmol, 1.0 equiv) was dissolved in CH_3OH (30 mL), followed by the addition of trimethyl orthoformate (21.22 g, 200 mmol, 2.0 equiv) and TsOH·H_2O (380 mg, 2 mmol, 0.02 equiv). The product was refluxed at 60 ºC (metal sand bath) for 4 hours, and then was purified by flash column chromatrography directly (petroleum ether : ethyl acetate = 80 : 1) to afford 6.2 g the product **S1** (70% yield).

A mixture of compound **S1** (4.6 g, 26 mmol, 1.3 equiv) and 3, 4-dimethyl-1-phenyl-1*H*-phosphole (3.8 g, 20 mmol, 1.0 equiv) was heated at 140 °C for 4 h. Afterwards, cool the reaction solution to room temperature, then add sulfur powder (768 mg, 24 mmol, 1.2 equiv) to it and react at 60 ºC (metal sand bath) for one hour. The progress of the reaction was followed by ^{31}P NMR. The product was purified by column chromatography (petroleum ether : ethyl acetate = 80 : 1) to afford 5.7 g the product **S2** (90% yield).

Compound **S2** (5.1 g, 12.8 mmol, 1.0 equiv) was dissolved in DCM (50 mL) at 0 ºC, and CF_3COOH (2.0 mL, 2.0 equiv) was added dropwise for overnight reaction. Then, the reaction was complete as monitored by TLC, and remove the solvent, and rapid column passing (petroleum ether : ethyl acetate = 20 : 1). Then, afford 4.7 g the product **S3** (90% yield).

A mixture of compound **S3** (2.3 g, 6.5 mmol, 1.0 equiv) and (*R*)-*tert*-butyl sulfinyl amide (940 mg, 7.8 mmol, 1.2 equiv) and $Ti(O^iPr)_4$ (4.6 g, 16.3 mmol, 2.5 equiv) was refluxed in THF at 70 °C (metal sand bath) for 3 h, and the reaction was quenched with H_2O. The organic layer was removed, and the aqueous layer was extracted twice with AcOEt. The combined organic layers were dried over $MgSO_4$, filtered, concentrated, and purified by flash chromatography (petroleum ether : ethyl acetate = 5 : 1) afforded the product **S4** (82% yield).

Dissolve **S4** (1.4 g, 3 mmol, 1.0 equiv) in THF at 0 °C was added a solution of 4-*tert*-butyl phenyl magnesium bromide (3 mL, 6 mmol, 2.0 equiv) in THF. Stirring was continued at 0 °C for 0.5 h. When completed, the reaction mixture was quenched by the addition of H_2O and diluted with AcOEt. The organic layer was removed, and the aqueous layer was extracted twice with AcOEt. The combined organic layers were dried over $MgSO_4$, filtered, concentrated, and purified by flash chromatography (petroleum ether : ethyl acetate = 4 : 1) afforded the product **S5a** (36% yield) and **S5a'** (49% yield).

Compound **S5a** (500 mg) after being reduced by Raney Nickel, crude products are obtained by reducing pressure and removing solvents. Then the crude products was dissolved in CH_3OH (30 mL), and HCl (1.0 mL) was added dropwise. After continuous stirring for 2 hours, a saturated $NaHCO_3$ aqueous solution was added to quench the reaction. The organic layer was removed, and the aqueous layer was extracted twice with AcOEt. The combined organic layers were dried over $MgSO_4$, filtered, concentrated to obtain crude product **S6a**, which can be directly used for the next reaction without purification.

To a solution of 9, 9' - dimethyloxanthracene (2.1 g, 10 mmol, 1.0 equiv) dissolved in Et_2O was added TMEDA (2.3 g, 20 mmol, 2.0 equiv) at 0 ºC, and slowly added *n*-BuLi (19 mL, 1.6 M, 30 mmol, 3.0 equiv) dropwise. Subsequently, the reaction was placed at 40 ºC for 4 hours, and after completion, it was cooled to 0 ºC. Then, a THF solution of I_2 substance (6.4 g, 25 mmol, 2.5 equiv) was slowly added, and the reaction lasted overnight. After the reaction was completed, quenched the reaction with saturated $Na_2S_2O_3$ aqueous solution. The organic layer was removed, and the aqueous layer was extracted twice with AcOEt. The combined organic layers were dried over $MgSO_4$, filtered, concentrated, and purified by flash chromatography (petroleum ether : ethyl acetate = 10 : 1) afforded the product **S7** (61% yield).

Compound **S7** (4.6 g, 10 mmol, 1.0 equiv) was dissolved in THF and placed at -78 ºC. *n*-BuLi (4 mL, 2.5 M, 10 mmol, 1.0 equiv) was added dropwise, and PPh_2Cl (1.8 mL, 10 mmol, 1.0 equiv) was slowly added after continuous stirring for 1.5 hours. *n*-BuLi (8 mL, 2.5 M, 20 mmol, 2.0 equiv) was added after continuous reaction for 1 hour. After reaction for 1.5 hours, DMF (2.5 mL, 20 mmol, 2.0 equiv) was added, and after stirring for 2 hours, the reaction was quenched with H_2O. The organic layer was removed, and the aqueous layer was extracted twice with AcOEt. The combined organic layers were dried over $MgSO_4$, filtered, concentrated, and purified by flash chromatography (petroleum ether : ethyl acetate = 15 : 1) afforded the product **S8** (60% yield).

A mixture of **S6a** and compound **S8** (400 mg) and anhydrous $MgSO_4$ refluxed in methanol at 70 °C for 6 hours. After the reaction, DCM (10 mL) and $NaBH_4$ (114 mg, 3.0 equiv) were added for 2 hours. Then was purified by flash column chromatrography directly to afford the corresponding product **ZU-1** (51% yield).

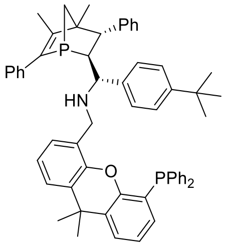

ZU-1

(*S*)-1-(4-(*tert*-butyl)phenyl)-1-((1*R*, 2*R*, 3*S*, 4*S*)-4, 5-dimethyl-3, 6-diphenyl-1-phosphabicyclo[2.2.1]hept-5-en-2-yl)-*N*-((5-(diphenylphosphanyl)-9, 9-dimethyl-9*H*-xanthen-4-yl)methyl)methanamine

White solid. **Yield:** 70%. **MP:** 125 - 126.5 °C. $[\alpha]_D$ = - 111 (*c* = 0.1, CH_2Cl_2, 29.4 °C). **^{31}P NMR (121 MHz, $CDCl_3$)** δ - 16.98 (s), - 20.27 (s) ppm. **^{1}H NMR (300 MHz, $CDCl_3$)** δ 7.50 - 7.29 (m, 13H), 7.26 - 7.13 (m, 8H), 7.09 - 6.92 (m, 8H), 6.56 - 6.52 (m, 1H), 3.98 (dd, *J* = 10.0, 5.5 Hz, 1H), 3.33 - 3.19 (m, 2H), 2.93 (d, *J* = 6.9 Hz, 1H), 2.82 (t, *J* = 6.0 Hz, 1H), 2.41 (s, 1H), 1.67 (s, 3H), 1.63 (s, 3H), 1.54 (s, 3H), 1.47 - 1.41 (m, 1H), 1.39 (s, 9H), 1.18 (s, 3H), 0.84 (t, *J* = 10.6 Hz, 1H) ppm. **^{13}C NMR (75 MHz, $CDCl_3$)** δ 152.91 (s), 151.54 (d, *J* = 15.6 Hz), 149.85 (s), 148.19 (s), 142.29 (s), 142.20 (s), 142.07 (s), 139.25 (s), 139.06 (d, *J* = 2.8 Hz), 138.97 (s), 136.39 (d, *J* = 10.6 Hz), 135.81 (d, *J* = 10.1 Hz), 134.43 (s), 134.17 (s), 133.93 (s), 133.66 (s), 131.26 (s), 129.66 (d, *J* = 1.2 Hz), 129.32 (s), 129.18 (s), 128.76 (s), 128.65 (s), 128.59 (s), 128.54 (s), 128.44 (s), 128.41 (s), 128.32 (s), 128.25 (s), 128.18 (s), 127.86 (s), 127.76 (s), 126.78 (s), 126.31 (s), 126.18 (s), 125.01 (d, *J* = 14.1 Hz), 124.88 (s), 124.23 (s), 123.20 (s), 122.76 (s), 65.33 (d, *J* = 10.1 Hz), 63.82 (d, *J* = 4.9 Hz), 55.27 (d, *J* = 18.0 Hz), 55.03 (d, *J* = 2.1 Hz), 48.53 (d, *J* = 4.1 Hz), 47.07 (s), 34.53 (s), 34.15 (d, *J* = 1.1 Hz), 32.46 (s), 32.27 (s), 31.56 (s), 20.79 (s), 16.56 (s) ppm. **HRMS** (ESI) (m/z) $[M+H]^+$ Calcd for $C_{59}H_{60}NOP_2^+$ 860.4145; found 860.4150 .

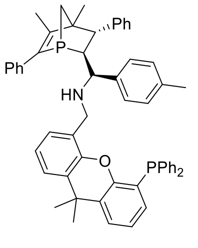

ZU-2

(*R*)-1-((1*R*, 2*R*, 3*S*, 4*S*)-4, 5-dimethyl-3, 6-diphenyl-1-phosphabicyclo[2.2.1]hept-5-en-2-yl)-*N*-((5-(diphenylphosphanyl)-9, 9-dimethyl-9*H*-xanthen-4-yl)methyl)-1-(4-tolyl)methanamine

White solid. **Yield:** 66%. **MP:** 69.0 - 69.7 °C. $[\alpha]_D$ = + 109 (*c* = 0.1, CH_2Cl_2, 28.0 °C).**^{31}P NMR (121 MHz, $CDCl_3$)** *δ* - 16.86 (s), - 20.42 (s) ppm. **^{1}H NMR (300 MHz, $CDCl_3$)** δ 7.42 - 7.37 (m, 5H), 7.35 - 7.26 (m, 6H), 7.23 - 7.11 (m, 11H), 7.03 - 6.81 (m, 8H), 6.50 - 6.46 (m, 1H), 3.87 (dd, *J* = 10.1, 5.8 Hz, 1H), 3.15 (q, *J* = 13.3 Hz, 2H), 2.87 (d, *J* = 6.9 Hz, 1H), 2.77 (t, *J* = 6.2 Hz, 1H), 2.38 (s, 3H), 1.62 (s, 3H), 1.57 (s, 3H), 1.49 (s, 3H), 1.38 - 1.34 (m, 1H), 1.13 (s, 3H), 0.85 - 0.75 (m, 1H) ppm. **^{13}C NMR (101 MHz, $CDCl_3$)** *δ* 152.86 (s), 151.59 (d, *J* = 15.5 Hz), 148.24 (s), 142.26 (s), 142.16 (s), 142.09 (s), 139.27 (s), 139.00 (s), 136.52 (s), 136.40 (s), 135.85 (d, *J* = 10.5 Hz), 134.37 (s), 134.17 (s), 133.82 (s), 133.62 (s), 131.25 (s), 129.71 (s), 129.35 (s), 129.16 (s), 128.77 (s), 128.69 (s), 128.65 (s), 128.61 (s), 128.57 (s), 128.54 (s), 128.48 (s), 128.41 (s), 128.35 (s), 128.28 (s), 128.12 (s), 127.87 (s), 127.76 (s), 126.70 (s), 126.30 (s), 126.10 (s), 124.93 (d, *J* = 14.0 Hz), 124.16 (s), 123.15 (s), 122.69 (s), 65.68 (d, *J* = 10.2 Hz), 63.88 (d, *J* = 4.9 Hz), 55.41 (s), 55.19 (s), 48.50 (s), 47.09 (s), 34.13 (s), 32.32 (s), 32.13 (s), 21.22 (s), 20.70 (s), 16.48 (s) ppm. **HRMS** (ESI) (m/z) $[M+H]^+$ Calcd for $C_{56}H_{54}NOP_2^+$ 818.3675; found 818.3694 .

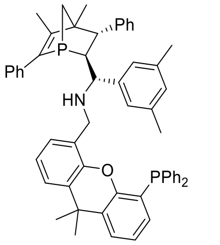

ZU-3

(*S*)-1-((1*R*, 2*R*, 3*S*, 4*S*)-4, 5-dimethyl-3, 6-diphenyl-1-phosphabicyclo[2.2.1]hept-5-en-2-yl)-1-(3, 5-dimethylphenyl)-*N*-((5-(diphenylphosphanyl)-9, 9-dimethyl-9*H*-xanthen-4-yl)methyl)methanamine

White solid. **Yield:** 71%. **MP:** 88.2 - 89.3 °C. $[\alpha]_D$ = - 284 (*c* = 0.1, CH_2Cl_2, 23.4 °C).**^{31}P NMR (121 MHz, $CDCl_3$)** *δ* - 16.76 (s), - 19.52 (s) ppm. **^{1}H NMR (300**

MHz, CDCl₃) δ 7.49 -7.46 (m, 3H), 7.41 - 7.35 (m, 5H), 7.28 - 7.28 (m, 6H), 7.19 - 7.16 (m, 5H), 7.13 - 7.06 (m, 3H), 7.06 - 6.98 (m, 3H), 6.96 - 6.95 (m, 3H), 6.60 - 6.56 (m, 1H), 3.93 (dd, J = 8.8, 6.6 Hz, 1H), 3.28 (q, J = 13.4 Hz, 2H), 3.03 (d, J = 6.7 Hz, 1H), 2.83 (t, J = 6.3 Hz, 1H), 2.40 (s, 6H), 1.68 (s, 3H), 1.65 (s, 3H), 1.56 (s, 3H), 1.50 - 1.47 (m, 1H), 1.23 (s, 3H), 1.07 (t, J = 10.5 Hz, 1H) ppm. **^{13}C NMR (101 MHz, $CDCl_3$)** δ 152.83 (s), 151.69 (d, J = 15.7 Hz), 148.26 (s), 142.53 (d, J = 2.5 Hz), 142.46 (s), 142.38 (s), 142.29 (s), 139.25 (d, J = 20.9 Hz), 137.30 (s), 136.54 (d, J = 10.9 Hz), 136.01 (d, J = 10.5 Hz), 134.45 (s), 134.25 (s), 133.92 (s), 133.72 (s), 131.33 (s), 129.82 (s), 129.41 (s), 129.18 (s), 128.80 (s), 128.77 (s), 128.66 (s), 128.58 (s), 128.51 (s), 128.46 (s), 128.39 (d, J = 1.5 Hz), 128.21 (s), 127.93 (s), 127.78 (s), 126.79 (s), 126.47 (d, J = 3.1 Hz), 126.31 (s), 126.17 (s), 125.06 (d, J = 14.0 Hz), 124.23 (s), 123.26 (s), 122.76 (s), 77.42 (d, J = 11.4 Hz), 77.16 (s), 76.84 (s), 66.33 (d, J = 11.2 Hz), 64.15 (d, J = 4.9 Hz), 55.55 (s), 55.22 (d, J = 17.8 Hz), 48.64 (d, J = 4.7 Hz), 47.27 (s), 34.21 (s), 32.36 (s), 32.26 (s), 29.80 (s), 21.59 (s), 20.85 (s), 16.58 (s) ppm. **HRMS** (ESI) (m/z) $[M+H]^+$ Calcd for $C_{57}H_{56}NOP_2^+$ 832.3832; found 832.3840 .

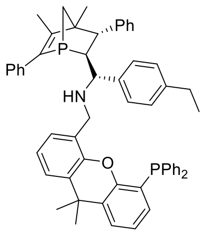

ZU-4

(S)-1-((1R, 2R, 3S, 4S)-4, 5-dimethyl-3, 6-diphenyl-1-phosphabicyclo[2.2.1]hept-5-en-2-yl)-N-((5-(diphenylphosphanyl)-9, 9-dimethyl-9H-xanthen-4-yl)methyl)-1-(4-ethylphenyl)methanamine

White solid. **Yield:** 77%. **MP:** 96.3 - 98.5 °C. $[\alpha]_D$ = - 130 (c = 0.1, CH_2Cl_2, 29.0 °C). **^{31}P NMR (162 MHz, $CDCl_3$)** δ - 16.63 (s), - 20.16 (s) ppm. **^{1}H NMR (400 MHz, $CDCl_3$)** δ 7.40 - 7.36 (m, 5H), 7.30 - 7.26 (m, 2H), 7.25 - 7.25 (m, 3H), 7.20 - 7.07 (m, 11H), 7.02 - 6.81 (m, 8H), 6.48 - 6.46 (m, 1H), 3.90 - 3.86 (m, 4.8 Hz, 1H), 3.23 - 3.13 (m, 13.4 Hz, 2H), 2.85 (d, J = 6.4 Hz, 1H), 2.79 - 2.73 (m, 1H), 2.63 (q, J = 7.5 Hz, 2H), 2.40 - 2.26 (m, 1H), 1.59 (s, 3H), 1.53 (s, 3H), 1.46 (s, 3H), 1.39 -

1.33 (m, 1H), 1.22 (t, *J* = 7.5 Hz, 3H), 1.10 (s, 3H), 0.79 (m, 1H) ppm. **^{13}C NMR (101 MHz, $CDCl_3$)** *δ* 152.91 (s), 151.66 (d, *J* = 15.6 Hz), 148.32 (s), 142.99 (s), 142.41 (s), 142.25 (s), 139.53 (d, *J* = 1.9 Hz), 139.30 (s), 139.09 (s), 136.52 (d, *J* = 10.8 Hz), 135.93 (d, *J* = 10.4 Hz), 134.45 (s), 134.25 (s), 133.93 (s), 133.73 (s), 131.34 (s), 129.78 (s), 129.43 (s), 129.24 (s), 128.80 (s), 128.75 (s), 128.71 (s), 128.67 (s), 128.58 (s), 128.51 (s), 128.45 (s), 128.38 (s), 128.23 (s), 127.96 (s), 127.85 (s), 127.60 (s), 126.81 (s), 126.40 (s), 126.22 (s), 125.05 (d, *J* = 14.0 Hz), 124.27 (s), 123.27 (s), 122.82 (s), 65.66 (d, *J* = 10.2 Hz), 63.95 (d, *J* = 4.9 Hz), 55.40 (d, *J* = 8.7 Hz), 55.39 (s), 48.62 (d, *J* = 4.5 Hz), 47.19 (s), 34.22 (s), 32.48 (s), 32.23 (s), 28.67 (s), 20.83 (s), 16.60 (s), 15.65 (s). **HRMS** (ESI) (m/z) $[M+H]^+$ Calcd for $C_{57}H_{56}NOP_2^+$ 832.3832; found 832.3836 .

ZU-5

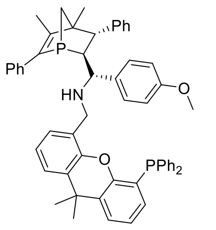

(*S*)-1-((1*R*, 2*R*, 3*S*, 4*S*)-4, 5-dimethyl-3, 6-diphenyl-1-phosphabicyclo[2.2.1]hept-5-en-2-yl)-*N*-((5-(diphenylphosphanyl)-9, 9-dimethyl-9*H*-xanthen-4-yl)methyl)-1-(4-methoxyphenyl)methanamine

White solid. **Yield:** 69%. **MP:** 103.8 - 104.9 °C. $[\alpha]_D$ = - 122 (*c* = 0.1, CH_2Cl_2, 26.6 °C). **^{31}P NMR (162 MHz, $CDCl_3$)** *δ* - 16.81 (s), - 20.52 (s) ppm. **^{1}H NMR (400 MHz, $CDCl_3$)** *δ* 7.42 - 7.36 (m, 5H), 7.32 - 7.22 (m, 5H), 7.22 - 7.07 (m, 9H), 7.00 - 6.80 (m, 10H), 6.47 - 6.44 (m, 1H), 3.81 (s, 1H), 3.14 (dd, *J* = 33.5, 13.4 Hz, 2H), 2.81 (d, *J* = 6.9 Hz, 1H), 2.73 (t, *J* = 6.2 Hz, 1H), 1.60 (s, 3H), 1.54 (s, 3H), 1.47 (s, 3H), 1.43 (s, 1H), 1.38 - 1.33 (m, 1H), 1.10 (s, 3H), 0.81 - 0.74 (m, 1H) ppm. **^{13}C NMR (101 MHz, $CDCl_3$)** *δ* 158.68 (s), 152.87 (s), 151.54 (d, *J* = 16.3 Hz), 148.25 (s), 142.19 (s), 142.11 (s), 142.02 (s), 139.07 (d, *J* = 21.0 Hz), 136.42 (d, *J* = 11.1 Hz), 135.79 (d, *J* = 10.1 Hz), 134.37 (s), 134.17 (s), 133.78 (s), 133.59 (s), 131.25 (s), 129.66 (s), 129.34 (s), 129.15 (s), 128.71 (s), 128.67 (s), 128.58 (s), 128.54 (s), 128.48 (s), 128.42 (s), 128.35 (s), 128.27 (s), 128.22 (s), 128.13 (s), 127.90 (s),

127.77 (s), 126.73 (s), 126.33 (s), 126.12 (s), 124.87 (d, *J* = 13.8 Hz), 124.22 (s), 123.16 (s), 122.70 (s), 113.40 (s), 65.19 (d, *J* = 9.9 Hz), 63.88 (s), 55.41 (s), 55.22 (s), 48.49 (s), 47.14 (s), 34.12 (s), 32.40 (s), 32.10 (s), 26.94 (s), 20.69 (s), 16.50 (s). **HRMS** (ESI) (m/z) $[M+H]^+$ Calcd for $C_{56}H_{54}NO_2P_2^+$ 834.3624; found 834.3605 .

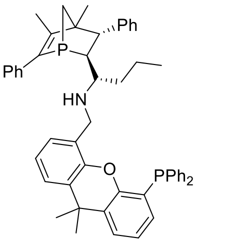

ZU-6

(*S*)-1-((1*R*, 2*R*, 3*S*, 4*S*)-4, 5-dimethyl-3, 6-diphenyl-1-phosphabicyclo[2.2.1]hept-5-en-2-yl)-*N*-((5-(diphenylphosphanyl)-9, 9-dimethyl-9*H*-xanthen-4-yl)methyl) butan-1-amine

White solid. **Yield:** 50%. **MP:** 96.5 - 97.1 °C. $[\alpha]_D$ = - 87 (*c* = 0.1, CH_2Cl_2, 29.8 °C). **^{31}P NMR (162 MHz, $CDCl_3$)** δ - 14.04 (s), - 16.33 (s) ppm. **^{1}H NMR (400 MHz, $CDCl_3$)** δ 7.50 (t, *J* = 7.4 Hz, 3H), 7.47 - 7.45 (m, 2H), 7.43 - 7.41 (m, 5H), 7.39 - 7.36 (m, 5H), 7.32 - 7.29 (m, 2H), 7.23 - 7.13 (m, 5H), 7.08 - 7.03 (m, 2H), 6.99 (t, *J* = 7.6 Hz, 1H), 6.63 - 6.59 (m, 1H), 3.70 - 3.59 (m, 2H), 2.98 (d, *J* = 6.4 Hz, 1H), 2.89 (p, *J* = 5.8 Hz, 1H), 2.41 (t, *J* = 5.7 Hz, 1H), 1.98 - 1.90 (m, 1H), 1.72 (s, 3H), 1.71 - 1.67 (m, 1H), 1.64 (s, 3H), 1.54 (s, 3H), 1.44 - 1.37 (m, 2H), 1.35 (s, 3H), 1.13 (ddd, *J* = 12.9, 11.4, 5.8 Hz, 1H), 0.98 - 0.91 (m, 1H), 0.63 (t, *J* = 7.3 Hz, 3H) ppm. **^{13}C NMR (101 MHz, $CDCl_3$)** δ 152.91 (s), 151.91 (d, *J* = 15.4 Hz), 148.19 (s), 143.18 (s), 142.49 (d, *J* = 15.9 Hz), 139.37 (s), 139.16 (s), 136.55 (d, *J* = 5.6 Hz), 136.44 (d, *J* = 5.2 Hz), 134.32 (s), 134.12 (s), 133.92 (s), 131.34 (s), 129.97 (s), 129.58 (s), 128.90 (s), 128.83 (s), 128.69 (s), 128.63 (s), 128.61 (s), 128.56 (s), 128.53 (s), 128.31 (s), 127.73 (s), 126.69 (s), 126.15 (d, *J* = 16.5 Hz), 125.04 (d, *J* = 14.6 Hz), 123.92 (s), 123.31 (s), 122.63 (s), 64.63 (d, *J* = 4.9 Hz), 59.54 (d, *J* = 12.5 Hz), 54.19 (d, *J* = 2.6 Hz), 52.07 (d, *J* = 16.2 Hz), 49.11 (d, *J* = 3.7 Hz), 46.43 (s), 36.84 (d, *J* = 9.9 Hz), 34.29 (d, *J* = 1.2 Hz), 32.86 (s), 31.25 (s), 20.99 (s), 19.00 (s), 16.41 (s), 14.12 (s) ppm. **HRMS** (ESI) (m/z) $[M+H]^+$ Calcd for $C_{52}H_{54}NOP_2^+$ 770.3675; found 770.3682.

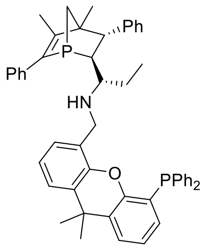

ZU-7

(*S*)-1-((1*R*, 2*R*, 3*S*, 4*S*)-4, 5-dimethyl-3, 6-diphenyl-1-phosphabicyclo[2.2.1] hept-5-en-2-yl)-*N*-((5-(diphenylphosphanyl)-9, 9-dimethyl-9*H*-xanthen-4-yl)methyl) propan-1-amine

White solid. **Yield:** 72%. **MP:** 121.3 - 124.1 °C. $[\alpha]_D$ = - 125 (*c* = 0.1, CH_2Cl_2, 32.2 °C). ^{31}**P NMR (121 MHz, $CDCl_3$)** *δ* - 14.09 (s), - 16.34 (s) ppm. ^{1}**H NMR (400 MHz, $CDCl_3$)** *δ* 7.49 - 7.44 (m, 6H), 7.43 - 7.35 (m, 11H), 7.31 - 7.29 (m, 3H), 7.22 - 7.12 (m, 6H), 7.07 - 6.97 (m, 3H), 6.62 - 6.57 (m, 1H), 3.65 - 3.53 (m, 2H), 2.99 (d, *J* = 6.0 Hz, 1H), 2.84 - 2.77 (m, 1H), 2.42 - 2.38 (m, 1H), 1.90 (t, *J* = 10.4 Hz, 1H), 1.71 (s, 3H), 1.63 (s, 3H), 1.52 (s, 3H), 1.33 (s, 3H), 0.95 - 0.93 (m, 2H), 0.92 - 0.90 (m, 1H), 0.63 (t, *J* = 7.2 Hz, 3H) ppm. ^{13}**C NMR (101 MHz, $CDCl_3$)** *δ* 152.91 (s), 151.85 (d, *J* = 15.8 Hz), 148.02 (s), 143.18 (s), 142.32 (d, *J* = 15.7 Hz), 139.24 (d, *J* = 20.1 Hz), 136.55 (s), 136.46 (s), 136.36 (s), 134.23 (s), 134.16 (s), 134.03 (s), 133.96 (s), 131.36 (s), 129.91 (s), 129.49 (s), 128.87 (s), 128.80 (s), 128.62 (s), 128.57 (s), 128.55 (s), 128.51 (s), 128.29 (s), 127.72 (s), 127.49 (s), 126.73 (s), 126.21 (s), 126.07 (s), 124.98 (d, *J* = 14.2 Hz), 123.89 (s), 123.29 (s), 122.61 (s), 64.74 (d, *J* = 4.6 Hz), 61.85 (d, *J* = 12.7 Hz), 54.47 (s), 51.47 (d, *J* = 16.6 Hz), 49.01 (d, *J* = 3.6 Hz), 46.25 (s), 34.26 (s), 32.83 (s), 31.43 (s), 26.85 (d, *J* = 8.6 Hz), 20.95 (s), 16.40 (s), 10.00 (s) ppm. **HRMS** (ESI) (m/z) $[M+H]^+$ Calcd for $C_{51}H_{52}NOP_2^+$ 756.3519; found 756.3535.

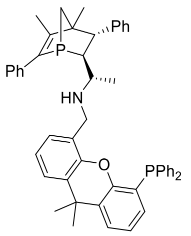

ZU-8

(*S*)-1-((1*R*, 2*R*, 3*S*, 4*S*)-4, 5-dimethyl-3, 6-diphenyl-1-phosphabicyclo[2.2.1]hept-5-en-2-yl)-*N*-((5-(diphenylphosphanyl)-9, 9-dimethyl-9*H*-xanthen-4-yl)methyl) ethan-1-amine

White solid. **Yield:** 78%. **MP:** 107.6 – 110.1 °C. $[\alpha]_D$ = - 96 (*c* = 0.1, CH_2Cl_2, 32.0 °C). **^{31}P NMR (121 MHz, $CDCl_3$)** δ - 16.34 (s), - 16.49 (s) ppm. **^{1}H NMR (300 MHz, $CDCl_3$)** δ 7.45 (m, 7H), 7.37 - 7.32 (m, 10H), 7.30 (m, 2H), 7.13 (m, 4H), 7.03 (m, 1H), 6.93 (s, 1H), 6.58 (m, 1H), 3.56 (d, *J* = 14.3 Hz, 1H), 3.41 (d, *J* = 14.3 Hz, 1H), 2.96 - 2.89 (m, 2H), 2.35 (t, *J* = 6.8 Hz, 1H), 1.67 (s, 3H), 1.65 (s, 3H), 1.51 (s, 3H), 1.33 (m, 1H), 1.31 (s, 3H), 1.23 (d, *J* = 6.2 Hz, 3H), 0.94 (m, 1H) ppm. **^{13}C NMR (75 MHz, $CDCl_3$)** δ 152.54 (s), 151.79 (d, *J* = 15.6 Hz), 147.91 (s), 142.75 (s), 139.12 (d, *J* = 21.0 Hz), 136.52 (s), 136.37 (s), 136.21 (s), 134.21 (s), 134.14 (s), 133.94 (s), 133.87 (s), 131.39 (s), 129.83 (s), 129.42 (s), 128.86 (s), 128.81 (s), 128.76 (s), 128.63 (s), 128.59 (s), 128.57 (s), 128.54 (s), 128.48 (s), 128.43 (s), 128.27 (s), 127.80 (s), 127.17 (s), 126.77 (s), 126.24 (s), 126.20 (s), 124.91 (d, *J* = 14.3 Hz), 123.99 (s), 123.30 (s), 122.74 (s), 64.73 (d, *J* = 4.8 Hz), 56.83 (d, *J* = 13.7 Hz), 55.54 (s), 54.69 (d, *J* = 16.4 Hz), 49.02 (d, *J* = 3.9 Hz), 45.87 (s), 34.21 (s), 32.54 (s), 31.86 (s), 22.71 (s), 20.89 (s), 16.39 (s) ppm. **HRMS** (ESI) (m/z) $[M+H]^+$ Calcd for $C_{50}H_{50}NOP_2^+$ 742.3362; found 742.3358.

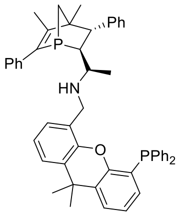

ZU-9

(*R*)-1-((1*R*, 2*R*, 3*S*, 4*S*)-4, 5-dimethyl-3, 6-diphenyl-1-phosphabicyclo[2.2.1]hept-5-en-2-yl)-*N*-((5-(diphenylphosphanyl)-9, 9-dimethyl-9*H*-xanthen-4-yl)methyl) ethan-1-amine

White solid. **Yield:** 78%. **MP:** 92.1 – 92.6 °C. $[\alpha]_D$ = + 178 (*c* = 0.1, CH_2Cl_2, 20.7 °C). **^{31}P NMR (121 MHz, $CDCl_3$)** δ - 16.16 (s), - 16.44 (s) ppm. **^{1}H NMR (600 MHz, $CDCl_3$)** δ 7.45 – 7.33 (m, 7H), 7.33 – 7.27 (m, 7H), 7.26 – 7.19 (m, 5H), 7.12 – 7.02 (m, 4H), 7.01 – 6.95 (m, 1H), 6.93 – 6.85 (m, 1H), 6.56 – 6.49 (m, 1H), 3.55

– 3.43 (m, 1H), 3.43 – 3.32 (m, 1H), 3.02 – 2.74 (m, 2H), 2.33 – 2.26 (m, 1H), 1.65 – 1.58 (m, 7H), 1.45 (d, *J* = 4.8 Hz, 3H), 1.29 – 1.15 (m, 7H) ppm. **^{13}C NMR (151 MHz, CDCl$_3$)** *δ* 152.57, 151.80, 147.92, 142.67, 139.13, 138.98, 136.21, 134.14, 134.02, 133.90, 131.40, 129.82, 129.46, 129.31, 128.90, 128.83, 128.75, 128.60, 128.55, 128.49, 128.31, 128.25, 128.16, 127.79, 126.77, 126.23, 124.94, 124.84, 123.31, 122.76, 64.77, 60.41, 56.85, 55.50, 54.62, 49.02, 45.78, 34.21, 32.49, 31.84, 20.83, 16.35, 14.24 ppm. **HRMS** (ESI) (m/z) $[M+H]^+$ Calcd for $C_{50}H_{49}NNaOP_2^+$ 764.3182; found 764.3237 .

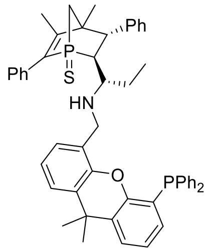

ZU-10

(*S*)-1-((1*R*, 2*R*, 3*S*, 4*S*)-4, 5-dimethyl-3, 6-diphenyl-1-phosphabicyclo[2.2.1]hept-5-en-2-yl)-*N*-((5-(diphenylphosphanyl)-9, 9-dimethyl-9*H*-xanthen-4-yl)methyl) ethan-1-amine

White solid. **Yield:** 71%. **MP:** 112.3 – 112.7 °C. $[\alpha]_D$ = - 18 (*c* = 0.1, CH_2Cl_2, 22.8 °C). **^{31}P NMR (243 MHz, CDCl$_3$)** *δ* – 58.45 (s), - 15.89 (s) ppm. **^{1}H NMR (600 MHz, CDCl$_3$)** *δ* 7.2 (d, *J* = 4.5 Hz, 4H), 7.2 – 7.2 (m, 8H), 7.2 – 7.1 (m, 3H), 7.1 – 7.1 (m, 1H), 7.1 (d, *J* = 6.2 Hz, 1H), 7.0 (s, 1H), 7.0 – 7.0 (m, 3H), 6.9 (d, *J* = 7.4 Hz, 2H), 6.8 – 6.8 (m, 2H), 6.3 – 6.3 (m, 1H), 3.8 (d, *J* = 14.3 Hz, 1H), 3.5 (d, *J* = 14.3 Hz, 1H), 3.1 (d, *J* = 5.9 Hz, 1H), 3.0 – 3.0 (m, 1H), 2.7 – 2.6 (m, 1H), 2.3 – 2.2 (m, 1H), 1.9 – 1.9 (m, 1H), 1.4 (s, 3H), 1.3 (s, 3H), 1.2 (d, *J* = 2.4 Hz, 3H), 1.1 (s, 3H), -0.0 (t, *J* = 7.3 Hz, 3H) ppm. **^{13}C NMR (151 MHz, CDCl$_3$)** *δ* 155.9, 155.8, 151.8, 151.7, 148.6, 140.5, 140.5, 136.5, 136.4, 136.2, 136.1, 136.1, 134.3, 134.2, 134.0, 133.9, 133.2, 133.2, 131.2, 130.0, 129.4, 129.3, 129.3, 129.0, 128.9, 128.9, 128.8, 128.7, 128.6, 128.6, 128.6, 128.5, 128.3, 128.0, 127.3, 126.9, 126.8, 124.7, 124.6, 124.2, 123.3, 122.7, 55.6, 53.0, 52.6, 52.4, 50.5, 50.4, 49.1, 48.8, 47.4, 34.2,

32.8, 31.4, 29.7, 29.3, 28.8, 28.7, 20.2, 20.1, 16.2, 16.2, 10.7 ppm. **HRMS** (ESI) (m/z) $[M+H]^+$ Calcd for $C_{51}H_{52}NOP_2S^+$ 788.3239; found 788.3292 .

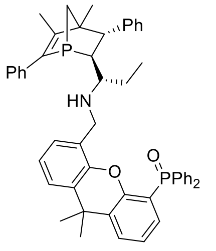

ZU-11

(*R*)-1-((1*R*, 2*R*, 3*S*, 4*S*)-4, 5-dimethyl-3, 6-diphenyl-1-phosphabicyclo[2.2.1]hept-5-en-2-yl)-*N*-((5-(diphenylphosphanyl)-9, 9-dimethyl-9*H*-xanthen-4-yl)methyl) ethan-1-amine

White solid. **Yield:** 56%. **MP:** 121.5 – 122.1 °C. $[\alpha]_D$ = + 66 (*c* = 0.1, CH_2Cl_2, 22.3 °C). **^{31}P NMR (121 MHz, $CDCl_3$)** δ 26.93 (s), - 17.15 (s) ppm. **^{1}H NMR (600 MHz, $CDCl_3$)** δ 7.2 (s, 2H), 7.2 (s, 2H), 7.2 – 7.2 (m, 1H), 7.2 – 7.2 (m, 7H), 7.2 – 7.1 (m, 3H), 7.1 – 7.1 (m, 1H), 7.1 – 7.0 (m, 1H), 7.0 (s, 1H), 7.0 – 7.0 (m, 3H), 6.9 (d, *J* = 7.3 Hz, 2H), 6.8 (td, *J* = 7.6, 2.1 Hz, 2H), 6.3 – 6.3 (m, 1H), 3.8 (d, *J* = 14.3 Hz, 1H), 3.5 (d, *J* = 14.3 Hz, 1H), 3.0 (q, *J* = 9.0 Hz, 2H), 2.7 – 2.6 (m, 1H), 2.3 – 2.2 (m, 1H), 1.4 (s, 3H), 1.3 (s, 3H), 1.2 (d, *J* = 2.4 Hz, 3H), 1.1 (s, 3H), 0.8 – 0.8 (m, 1H), 0.7 – 0.6 (m, 2H), 0.0 (t, *J* = 7.3 Hz, 3H) ppm. **^{13}C NMR (151 MHz, $CDCl_3$)** δ 155.9, 155.8, 151.8, 151.7, 148.6, 140.5, 140.5, 136.5, 136.4, 136.2, 136.1, 136.1, 134.3, 134.2, 134.0, 133.9, 133.2, 133.2, 131.2, 130.0, 129.4, 129.3, 129.3, 129.0, 128.9, 128.9, 128.8, 128.7, 128.6, 128.6, 128.6, 128.5, 128.3, 128.0, 127.3, 126.9, 126.8, 124.7, 124.6, 124.2, 123.3, 122.7, 55.6, 53.0, 52.6, 52.4, 50.5, 50.4, 49.1, 48.8, 47.4, 34.2, 32.8, 31.4, 29.7, 29.3, 28.8, 28.7, 20.2, 20.1, 16.2, 16.2, 10.7 ppm. **HRMS** (ESI) (m/z) $[M+H]^+$ Calcd for $C_{51}H_{52}NO_2P_2^+$ 772.3468; found 772.3450.

2.2 General procedure for the preparation of substrates.[1]

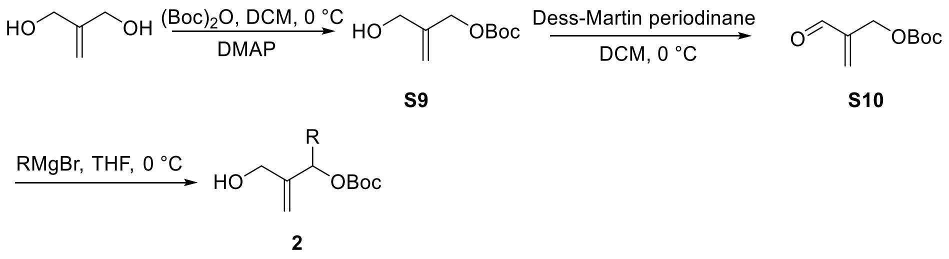

To a solution of 2-methylene-1,3-propanediol (4.4 g, 50 mmol, 1.0 equiv) in DCM at 0 °C was added DMAP (305 mg, 2.5 mmol, 0.05 equiv), and added $(Boc)_2O$ (12.0 g, 55 mmol, 1.1 equiv) solution dissolved in DCM dropwise. After half an hour of reaction, the solvent was evaporated under reduced pressure and purified by column chromatography (petroleum ether : ethyl acetate = 10 : 1) to obtain product **S9** (84% yield).

To a solution of compound **S9** (9.5 g, 50 mmol, 1.0 equiv) dissolved in DCM at 0 °C was slowly added Dess-Martin periodinane (25.5 g, 60 mmol, 1.2 equiv), after half an hour of reaction, the solvent was evaporated under reduced pressure and purified by column chromatography (petroleum ether : ethyl acetate = 10 : 1) to obtain product **S10** (91% yield).

Compound **S10** (0.9 g, 5 mmol, 1.0 equiv) was dissolved in THF, and grignard reagent (10 mmol, 2.0 equiv) was added dropwise at 0 °C. After 10 minutes of reaction, H_2O was added to quench the reaction. The organic layer was removed, and the aqueous layer was extracted twice with AcOEt. Concentrated, and purified by flash chromatography (petroleum ether : ethyl acetate = 10 : 1) afforded the product **2**.

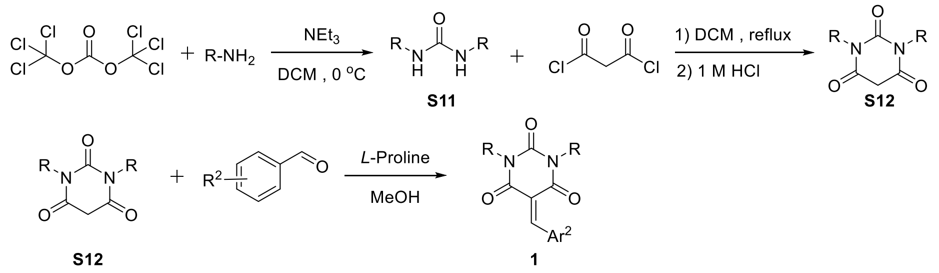

Adding the corresponding amine (60 mmol, 6.0 equiv) and triethylamine (60 mmol, 6.0 equiv) to the round bottom flask, and then slowly drip the dissolved triphosgene (10 mmol, 1.0 equiv) into the round bottom flask at 0 ^{o}C. After 15 minutes of reaction, H_2O was added to the reaction solution, and a large amount of white solid precipitated, then products **S11** obtained through filtration (90% yield).[6-7]

Dissolving the products **S11** (10 mmol, 1.0 equiv) in DCM, slowly add malonyl chloride (11 mmol, 1.1 equiv), and heat and reflux the reaction solution at 50 ^{o}C (metal sand bath) for 8 hours. Then use 1 M hydrochloric acid to treat the reaction solution until it becomes acidic (pH = 2 ~ 3), Using DCM and H_2O extraction, combining organic phases, and vacuum concentration to obtain yellow solid product **S12** (70% yield).

Dissolving the product **S12** (5 mmol, 1.0 equiv) and corresponding aldehydes (5.5 mmol, 1.0 equiv) in methanol, add catalytic amount of *L*-proline (10%), and react at room temperature for 24 hours. After a large amount of solid precipitation, filter and obtain the product **1**.

3. Reaction Optimization

Table S1. Screening of ligand.[a]

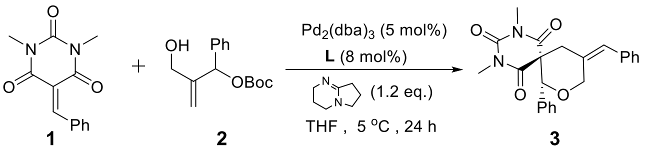

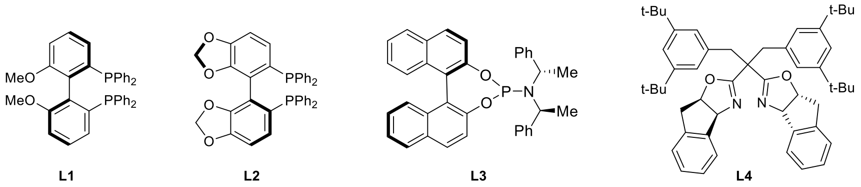

Entry	Ligand	Yield (%)	ee (%) [b]
1	**L1**	NR	
2	**L2**	NR	
3	**L3**	NR	
4	**L4**	NR	

[a] Reactions were performed with **1** (0.10 mmol), **2** (0.05 mmol) in THF (1.0 mL), N_2.

[b] Determined by chiral HPLC analysis.

Table S2. Screening of the amount of base.[a]

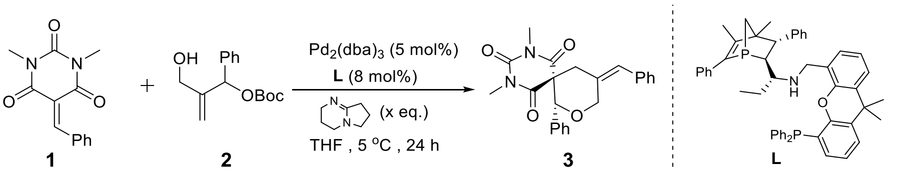

Entry	x	Yield (%)	ee (%) [b]
1	0.2	31	80
2	0.5	47	82
3	0.8	78	84
4	1.0	72	84
5	1.2	82	82
6	2.0	75	81
7	3.0	70	83
8	4.0	41	42

[a] Reactions were performed with **1** (0.10 mmol), **2** (0.05 mmol) in THF (1.0 mL), N_2.

[b] Determined by chiral HPLC analysis.

Table S3. Screening of metal.[a]

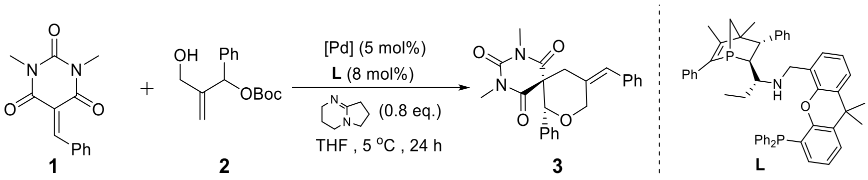

Entry	Metal	Yield (%)	ee (%) [b]
1	$Pd_2(dba)_3$	85	84
2	$Pd(dba)_2$	67	81
3	$Pd_2(dba)_3 \cdot CHCl_3$	63	85
4	$Pd(PPh_3)_4$	60	53
5	$Pd(OAc)_2$	NR	
6	$Pd(COD)Cl_2$	NR	
7	Pd(Allyl)Cl	NR	

[a] Reactions were performed with **1** (0.10 mmol), **2** (0.05 mmol) in THF (1.0 mL), N_2.

[b] Determined by chiral HPLC analysis.

Table S4. Screening of solvent.[a]

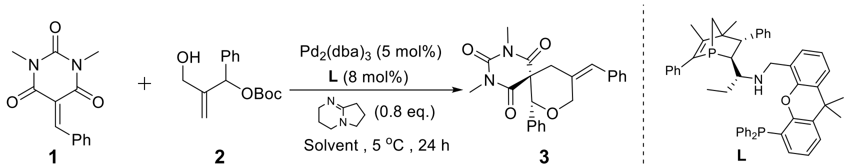

Entry	Solvent	Yield (%)	ee (%) [b]
1	THF	85	84
2	EA	80	90
3	Tol.	91	82
4	MTBE	26	84
5	Et_2O	42	88
6	DEC	78	90
7	DCM	NR	
8	CH_3CN	NR	
9	CH_3OH	NR	
10	1,2-Dimethoxyethane	47	89
11	Fluorobenzene	31	78

12	Bromobenzene	36	80
13	2-Methyl-THF	52	80
14	2-Methoxy-THF	59	89

[a] Reactions were performed with **1** (0.10 mmol), **2** (0.05 mmol) in THF (1.0 mL), N_2.

[b] Determined by chiral HPLC analysis.

Table S5. Screening of base.[a]

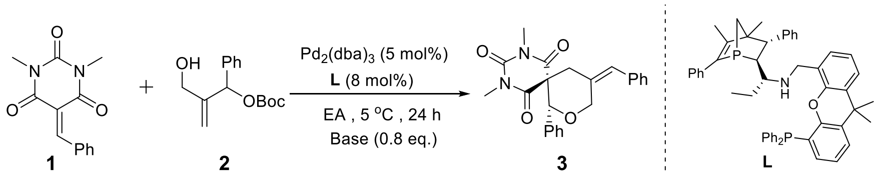

Entry	Base	Yield (%)	ee (%) [b]
1	DBN	80	90
2	DBU	77	89
3	DABCO	61	69
4	DMAP	56	72
5	Cs_2CO_3	76	39
6	Na_2CO_3	51	Rac
7	KH_2PO_4	NR	

[a] Reactions were performed with **1** (0.10 mmol), **2** (0.05 mmol) in THF (1.0 mL), N_2.

[b] Determined by chiral HPLC analysis.

Table S6. Screening of the coordination process.[a]

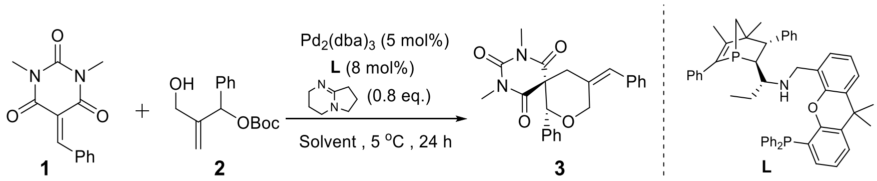

Entry	Base	Yield (%)	ee (%) [b]
1	THF	85	84
2 [c]	THF	85	93
3	EA	80	90
4 [c]	EA	79	92

[a] Reactions were performed with **1** (0.10 mmol), **2** (0.05 mmol) in THF (1.0 mL), N_2.

[b] Determined by chiral HPLC analysis.

[c] DBN participates in the coordination process between metals and ligands.

Table S7. Screening of the loading of catalysts.[a]

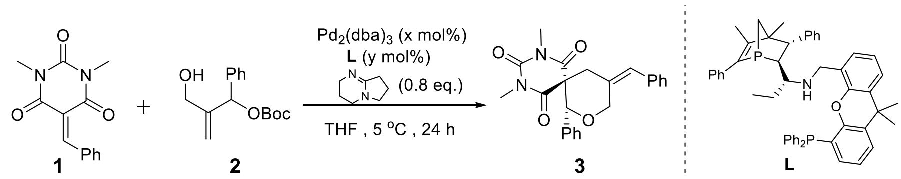

Entry	x	y	Yield (%)	ee (%) [b]
1	1	1.6	21	93
2	2.5	4	41	93
3	5	8	85	93
4 [c]	2.5	4	82	92

[a] Reactions were performed with **1** (0.10 mmol), **2** (0.05 mmol) in THF (1.0 mL), N_2.

[b] Determined by chiral HPLC analysis.

[c] The reaction was processed after 4 days of reaction.

Table S8. Screening of catalyst ratios.[a]

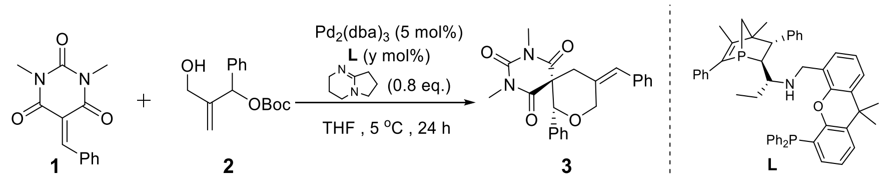

Entry	x	y	Yield (%)	ee (%) [b]
1	5	5	67	93
2	5	8	85	93
3	5	10	83	94
4	5	12	52	93

[a] Reactions were performed with **1** (0.10 mmol), **2** (0.05 mmol) in THF (1.0 mL), N_2.

[b] Determined by chiral HPLC analysis.

Table S9. Screening of catalyst temperature.[a]

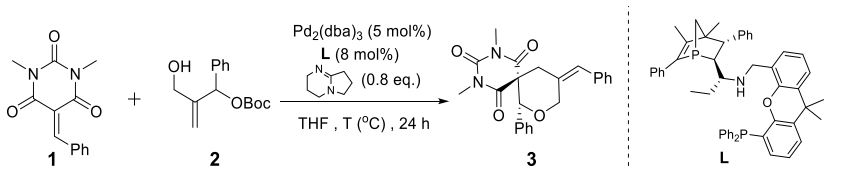

Entry	T (°C)	Yield (%)	ee (%) [b]
1	40	80	38
2	rt	71	42

3	15	65	62
4	10	65	87
5	5	85	93
6	0	67	87

[a] Reactions were performed with **1** (0.10 mmol), **2** (0.05 mmol) in THF (1.0 mL), N_2.

[b] Determined by chiral HPLC analysis.

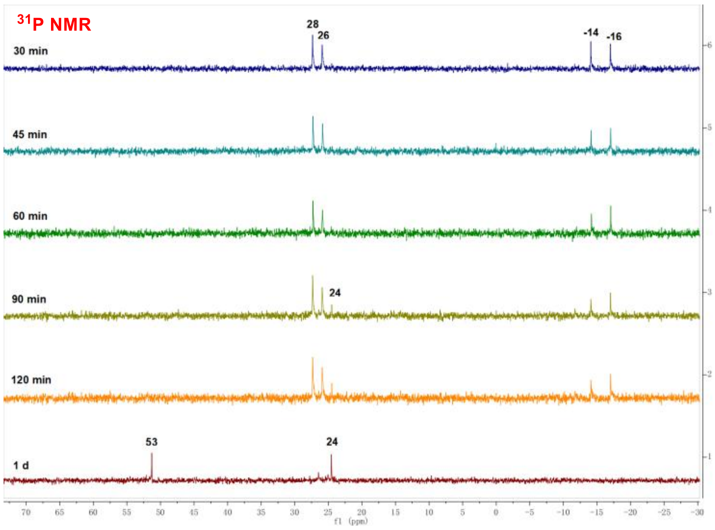

Figure S1. ^{31}PNMR tracking experiment of coordination time.

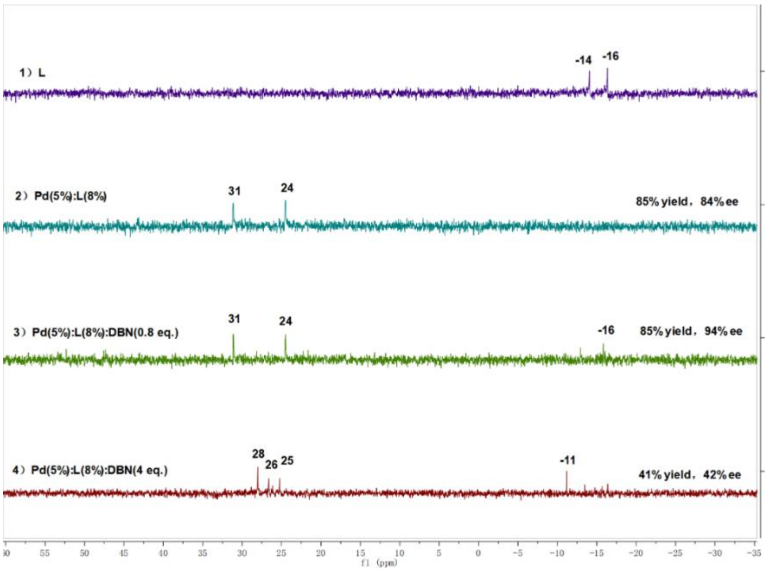

Figure S2. ^{31}PNMR tracking experiment of reaction system.

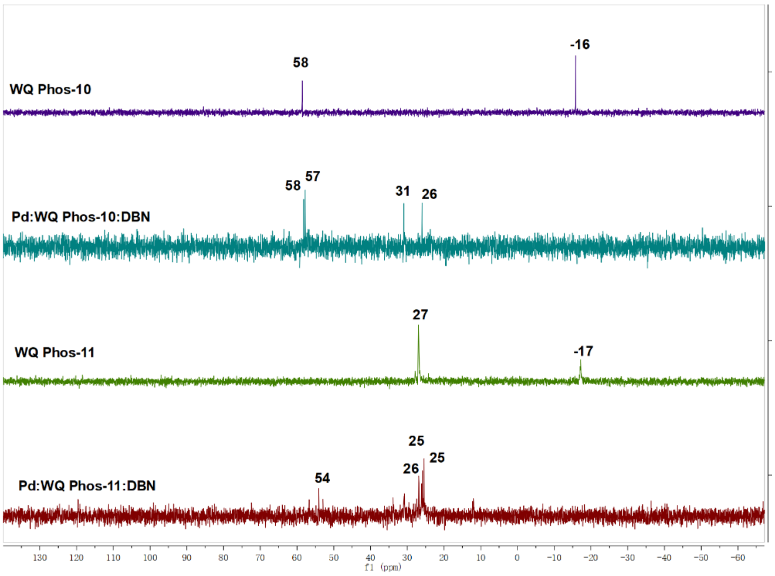

Figure S3. ^{31}PNMR of coordination system.

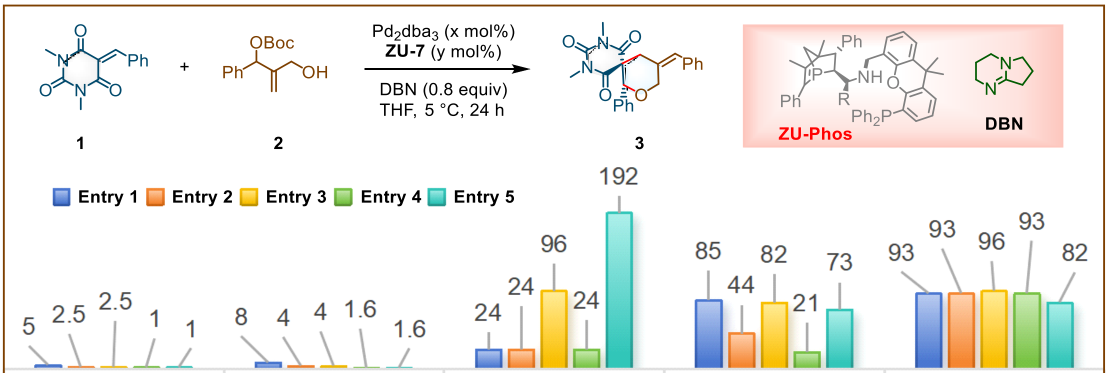

[Pd] (mol%)	ZU-7 (mol%)	Time (h)	Yield (%)	Ee (%)
5	8	24	85	93
2.5	4	24	44	93
2.5	4	96	82	96
1	1.6	24	21	93
1	1.6	192	73	82

Figure S4. The Effectiveness of Pd/ZU-Phos/DBN Catalytic System. Unless otherwise stated, reactions were performed with **1** (0.1 mmol) and **2** (0.05 mmol), in 1.0 mL of THF at 5 ºC. Isolated yield after chromatography. Determined by HPLC analysis.

4. General Procedure for Reactions

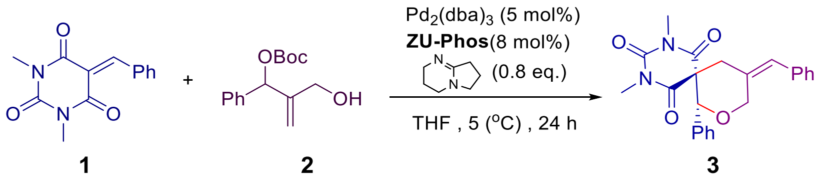

Ligand **ZU-7** (3 mg, 0.004 mmol, 8 mol%)、 $Pd_2(dba)_3$ (2.3 mg, 0.0025 mmol, 5 mol%) and DBN (5 μL, 0.4 mmol, 0.8 equiv.) were dissolved in THF (1.0 mL) in a 10 mL Schlenk tube under N_2. After stirring at room temperature for 30 minutes, compound **1** (0.05 mmol), **2** (0.1 mmol) were added at 5 °C. The reaction mixture was stirred at 40 °C until the substrate was consumed (monitored by TLC), and then was purified by flash column chromatrography (petroleum ether : AcOEt = 10 : 1), afford the corresponding product **3-42**.

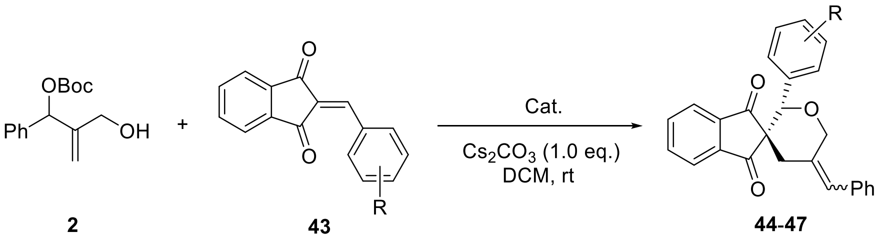

Condition A

Ligand **ZU-7** (7.2 mg, 0.01 mmol, 20 mol%)、 $Pd_2(dba)_3$ (4.6 mg, 0.005 mmol, 10 mol%) were dissolved in THF (1.0 mL) in a 10 mL Schlenk tube under N_2. After stirring at room temperature for 30 minutes, compound **2** (0.1 mmol), **51** (0.05 mmol) and Cs_2CO_3 (16 mg, 0.05 mmol, 1.0 equiv) were added. The reaction mixture was stirred at 5 °C until the substrate was consumed (monitored by TLC), and then was purified by flash column chromatrography (petroleum ether : AcOEt = 10 : 1), afford the corresponding product **52-55**.

Condition B

Ligand **ZU-7** (3.6 mg, 0.005 mmol, 10 mol%)、 $Pd_2(dba)_3$ (4.6 mg, 0.005 mmol, 10 mol%) and DBN (1 μL, 0.005 mmol, 10 mol%) were dissolved in THF (1.0 mL)

in a 10 mL Schlenk tube under N_2. After stirring at room temperature for 30 minutes, compound **2** (0.1 mmol), **51** (0.05 mmol) and Cs_2CO_3 (16 mg, 0.05 mmol, 1.0 equiv) were added. The reaction mixture was stirred at 5 °C until the substrate was consumed (monitored by TLC), and then was purified by flash column chromatrography (petroleum ether : AcOEt = 10 : 1), afford the corresponding product **52-55**.

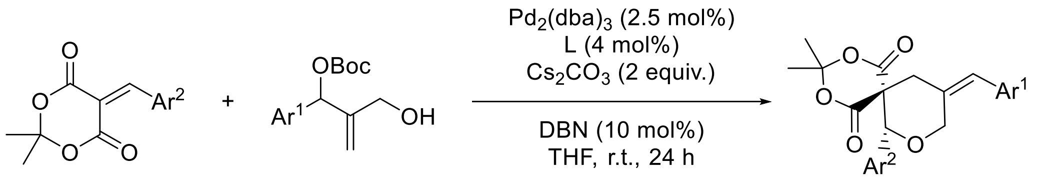

Ligand **ZU-7** (1.44 mg, 0.002 mmol, 4 mol%)、$Pd_2(dba)_3$ (1.2 mg, 0.0025 mmol, 2.5 mol%), DBN (10 mol%) were dissolved in THF (1.0 mL) in a 10 mL Schlenk tube under N_2. After stirring at room temperature for 30 minutes, compound **2** (0.1 mmol), **43** (0.05 mmol) and Cs_2CO_3 (32 mg, 0.10 mmol, 2.0 equiv) were added. The reaction mixture was stirred at 5 °C until the substrate was consumed (monitored by TLC), and then was purified by flash column chromatrography (petroleum ether : AcOEt = 10 : 1), afford the corresponding product **44-50**.

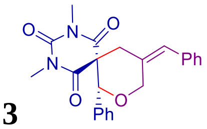

3

(*R,Z*)-10-benzylidene-2,4-dimethyl-7-phenyl-8-oxa-2,4-diazaspiro[5.5]undecane-1,3,5-trione

Yellow oil. **Yield:** 85% yield. $[\alpha]_D$ = + 191 (*c* = 0.055, CH_2Cl_2, 33.5 °C). **^{1}H NMR (600 MHz, $CDCl_3$)** δ 7.35 (t, *J* = 7.5 Hz, 2H), 7.31 – 7.27 (m, 2H), 7.27 – 7.24 (m, 2H, overlapped with the peak of chloroform), 7.22 (d, *J* = 7.5 Hz, 2H), 7.07 (d, *J* = 7.1 Hz, 2H), 6.49 (s, 1H), 5.19 (d, *J* = 13.6 Hz, 1H), 4.89 (s, 1H), 4.48 (d, *J* = 13.5 Hz, 1H), 3.59 (d, *J* = 14.8 Hz, 1H), 3.10 (s, 3H), 2.96 (s, 3H), 2.84 (d, *J* = 14.9 Hz, 1H) ppm. **^{13}C NMR (151 MHz, $CDCl_3$)** δ 169.4, 167.3, 150.2, 136.4, 135.8, 131.0, 129.4, 129.0, 128.3, 128.2, 127.0 126.8, 125.6, 85.1, 68.5, 58.1, 38.2, 28.5, 27.9 ppm. IR (cm^{-}): 2968, 1685, 1467, 1378, 1160, 951, 816. **HRMS** (ESI) (m/z) $[M+H]^+$

Calcd for $C_{23}H_{23}N_2O_4^+$ 391.1652; found 391.1650. **HPLC**: The product was analyzed by HPLC to determine the enantiomeric excess: 94% ee (Chiralpak AD-H, *n*-hexane/*i*-propanol = 70/30, 1 mL/min, 254 nm) t_R = 6.576 min, 8.126 min.

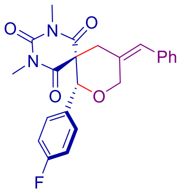

4

(*R*,*Z*)-10-benzylidene-7-(4-fluorophenyl)-2,4-dimethyl-8-oxa-2,4-diazaspiro[5.5] undecane-1,3,5-trione

White solid. **Yield:** 74% yield. **MP:** 146.6 – 147.2 °C. $[\alpha]_D$ = + 156 (*c* = 0.059, CH_2Cl_2, 25.3 °C). **^{1}H NMR (600 MHz, $CDCl_3$)** δ 7.36 (t, *J* = 7.6 Hz, 2H), 7.27 (t, *J* = 9.1 Hz, 2H), 7.22 (d, *J* = 7.5 Hz, 2H), 7.09 – 7.05 (m, 1H, overlapped with the peak of chloroform), 6.96 (t, *J* = 8.6 Hz, 2H), 6.48 (s, 1H), 5.18 (d, *J* = 13.5 Hz, 1H), 4.90 (s, 1H), 4.47 (d, *J* = 13.5 Hz, 1H), 3.57 (d, *J* = 14.8 Hz, 1H), 3.13 (s, 3H), 3.00 (s, 3H), 2.83 (d, *J* = 14.9 Hz, 1H). **^{13}C NMR (151 MHz, $CDCl_3$)** δ 169.4, 167.2, 163.0 (d, J_{C-F} = 248.8 Hz), 150.1, 136.3, 131.8 (d, J_{C-F} = 3.3 Hz), 130.7, 127.5, 127.5, 127.1 (d, J_{C-F} = 8.2 Hz), 115.3 (d, J_{C-F} = 21.6 Hz), 84.2, 68.5, 58.0, 38.4, 28.6, 28.0. IR (cm^-) 2969, 1671, 1378, 1160, 950, 816. **HRMS** (ESI) (m/z) $[M+H]^+$ Calcd for $C_{23}H_{22}FN_2O_4^+$ 409.1558; found 409.1556. **HPLC**: The product was analyzed by HPLC to determine the enantiomeric excess: 94% ee (Chiralpak AD-H, *n*-hexane/*i*-propanol = 85/15, 1 mL/min, 254 nm) t_R = 10.595 min, 11.809 min.

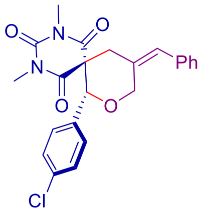

5

(*R*,*Z*)-10-benzylidene-7-(4-chlorophenyl)-2,4-dimethyl-8-oxa-2,4-diazaspiro[5.5] undecane-1,3,5-trione

White solid. **Yield:** 76% yield. **MP:** 163.3 – 163.8 °C. $[\alpha]_D$ = + 212 (*c* = 0.058, CH_2Cl_2, 28.8 °C). **^{1}H NMR (600 MHz, $CDCl_3$)** δ 7.35 (t, *J* = 7.6 Hz, 2H), 7.28 – 7.24 (m, 2H, overlapped with the peak of chloroform), 7.24 – 7.20 (m, 3H), 7.02 (d,

J = 8.4 Hz, 2H), 6.47 (s, 1H), 5.17 (d, *J* = 13.5 Hz, 1H), 4.90 (s, 1H), 4.45 (d, *J* = 13.5 Hz, 1H), 3.55 (d, *J* = 14.8 Hz, 1H), 3.14 (s, 3H), 3.00 (s, 3H), 2.83 (d, *J* = 14.8 Hz, 1H) ppm. **^{13}C NMR (151 MHz, $CDCl_3$)** δ 169.3, 167.1, 150.1, 136.3, 135.2, 134.5, 130.5, 129.0, 128.5, 128.3, 127.2, 127.1, 84.0, 68.4, 57.8, 38.6, 28.6, 28.0 ppm. IR (cm^{-}) 2969, 1671, 1378, 1160, 950, 816. **HRMS** (ESI) (m/z) $[M+H]^+$ Calcd for $C_{23}H_{22}ClN_2O_4^+$ 425.1263; found 425.1274. **HPLC**: The product was analyzed by HPLC to determine the enantiomeric excess: 91% ee (Chiralpak AD-H, *n*-hexane/*i*-propanol = 70/30, 1 mL/min, 254 nm) t_R = 7.547 min, 8.095 min.

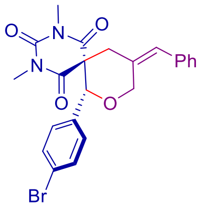

6

(*R*,*Z*)-10-benzylidene-7-(4-bromophenyl)-2,4-dimethyl-8-oxa-2,4-diazaspiro[5.5] undecane-1,3,5-trione

White solid. **Yield:** 80% yield. **MP:** 146.6 – 147.2 °C. $[\alpha]_D$ = + 22 (*c* = 0.214, CH_2Cl_2, 23.5 °C). **^{1}H NMR (600 MHz, $CDCl_3$)** δ 7.40 (d, *J* = 8.1 Hz, 2H), 7.36 (t, *J* = 7.4 Hz, 2H), 7.29 – 7.24 (m, 1H, overlapped with the peak of chloroform), 7.22 (d, *J* = 7.4 Hz, 2H), 6.96 (d, *J* = 8.0 Hz, 2H), 6.48 (s, 1H), 5.17 (d, *J* = 13.5 Hz, 1H), 4.89 (s, 1H), 4.46 (d, *J* = 13.6 Hz, 1H), 3.55 (d, *J* = 14.8 Hz, 1H), 3.15 (s, 3H), 3.00 (s, 3H), 2.83 (d, *J* = 14.8 Hz, 1H) ppm. **^{13}C NMR (151 MHz, $CDCl_3$)** δ 169.3, 167.0, 150.1, 136.2, 135.0, 131.4, 130.5, 129.0, 128.3, 127.4, 127.2, 127.1, 123.3, 84.0, 68.4, 57.7, 38.6, 28.6, 28.0 ppm. IR (cm^{-}) 2920, 1671, 1375, 1198, 910, 749. **HRMS** (ESI) (m/z) $[M+H]^+$ Calcd for $C_{23}H_{22}BrN_2O_4^+$ 469.0757; found 469.0750. **HPLC**: The product was analyzed by HPLC to determine the enantiomeric excess: 93% ee (Chiralpak AD-H, *n*-hexane/*i*-propanol = 70/30, 1 mL/min, 254 nm) t_R = 8.932 min, 9.710 min.

7

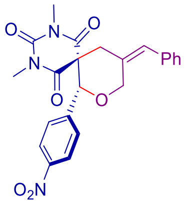

(*R*,*Z*)-10-benzylidene-2,4-dimethyl-7-(4-nitrophenyl)-8-oxa-2,4-diazaspiro[5.5] undecane-1,3,5-trione

White solid. **Yield:** 66% yield. **MP:** > 220 °C. $[\alpha]_D$ = + 211 (*c* = 0.055, CH_2Cl_2, 32.7 °C). **^{1}H NMR (600 MHz, $CDCl_3$)** δ 8.14 (t, *J* = 8.1 Hz, 2H), 7.40 – 7.36 (m, 2H), 7.33 – 7.26 (m, 3H, overlapped with the peak of chloroform), 7.25 – 7.21 (m, 2H), 6.50 (d, *J* = 5.6 Hz, 1H), 5.23 – 5.18 (m, 1H), 5.08 (d, *J* = 7.5 Hz, 1H), 4.51 – 4.46 (m, 1H), 3.59 – 3.53 (m, 1H), 3.18 (d, *J* = 7.9 Hz, 3H), 3.00 (d, *J* = 7.9 Hz, 3H), 2.90 – 2.84 (m, 1H). **^{13}C NMR (151 MHz, $CDCl_3$)** δ 169.1, 166.5, 149.9, 148.2, 143.2, 136.1, 129.8, 129.0, 128.4, 127.8, 127.2, 126.9, 123.4, 83.2, 68.3, 57.4, 39.1, 28.7, 28.1. IR (cm^-) 2920, 1673, 1370, 1276, 1154, 945, 700. **HRMS** (ESI) (m/z) $[M+H]^+$ Calcd for $C_{23}H_{22}N_3O_6^+$ 436.1503; found 436.1507. **HPLC**: The product was analyzed by HPLC to determine the enantiomeric excess: 98% ee (Chiralpak AD-H, *n*-hexane/*i*-propanol = 85/15, 1 mL/min, 254 nm) t_R = 23.159 min, 25.587 min.

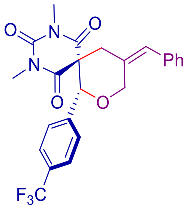

8

(*R*,*Z*)-10-benzylidene-2,4-dimethyl-7-((trifluoromethyl)phenyl)-8-oxa-2,4-diazaspiro[5.5]undecane-1,3,5-trione

White solid. **Yield:** 66% yield. **MP:** 100.5 – 100.9 °C. $[\alpha]_D$ = + 197 (*c* = 0.070, CH_2Cl_2, 25.3 ℃). **^{1}H NMR (600 MHz, $CDCl_3$)** δ 7.53 (d, *J* = 8.0 Hz, 2H), 7.38 – 7.34 (m, 2H), 7.29 – 7.25 (m, 1H, overlapped with the peak of chloroform), 7.25 – 7.20 (m, 4H), 6.49 (s, 1H), 5.19 (d, *J* = 13.5 Hz, 1H), 5.01 (s, 1H), 4.48 (d, *J* = 13.5 Hz, 1H), 3.56 (d, *J* = 14.7 Hz, 1H), 3.15 (s, 3H), 2.97 (s, 3H), 2.86 (d, *J* = 14.8 Hz, 1H) ppm. **^{13}C NMR (151 MHz, $CDCl_3$)** δ 169.3, 166.9, 150.0, 140.1, 136.2, 131.5 (q, $J_{C\text{-}F}$ = 32.9 Hz), 130.3, 129.0, 128.4, 127.4, 127.2, 125.2 (d, $J_{C\text{-}F}$ = 3.4 Hz), 123.7

(q, $J_{C\text{-}F}$ = 272.5 Hz), 83.8, 68.4, 57.7, 38.8, 28.6, 28.0 ppm. IR (cm^{-}) 2950, 1692, 1264, 1066, 896, 734. **HRMS** (ESI) (m/z) $[M+H]^+$ Calcd for $C_{24}H_{22}F_3N_2O_4^+$ 459.1526; found 459.1527. **HPLC**: The product was analyzed by HPLC to determine the enantiomeric excess: 90% ee (Chiralpak IA-H, *n*-hexane/*i*-propanol = 95/5, 1 mL/min, 254 nm) t_R = 17.308 min, 20.796 min.

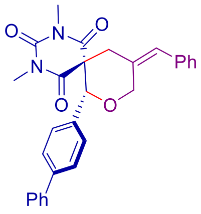

9

(*R*,*Z*)-7-([1,1'-biphenyl]-4-yl)-10-benzylidene-2,4-dimethyl-8-oxa-2,4-diazaspiro [5.5]undecane-1,3,5-trione

White solid. **Yield:** 96% yield. **MP:** 64.8 – 65.2 °C. $[\alpha]_D$ = + 39 (*c* = 0.200, CH_2Cl_2, 34.2 °C). **^{1}H NMR (600 MHz, $CDCl_3$)** δ 7.52 (d, *J* = 7.6 Hz, 2H), 7.49 (d, *J* = 7.6 Hz, 2H), 7.42 (t, *J* = 7.3 Hz, 2H), 7.36 (t, *J* = 7.8 Hz, 3H), 7.28 – 7.21 (m, 3H, overlapped with the peak of chloroform), 7.14 (d, *J* = 7.7 Hz, 2H), 6.49 (s, 1H), 5.21 (d, *J* = 13.5 Hz, 1H), 4.95 (s, 1H), 4.49 (d, *J* = 13.5 Hz, 1H), 3.59 (d, *J* = 14.8 Hz, 1H), 3.14 (s, 3H), 2.99 (s, 3H), 2.86 (d, *J* = 14.8 Hz, 1H) ppm. **^{13}C NMR (151 MHz, $CDCl_3$)** δ 169.5, 167.4, 150.1, 142.3, 140.2, 136.4, 134.8, 131.0, 129.1, 128.8, 128.3, 127.7, 127.2, 127.0, 126.9, 126.9, 126.1, 84.8, 68.5, 58.1, 38.3, 28.6, 28.0 ppm. IR (cm^{-}) 2969, 1673, 1378, 1107, 950, 749. **HRMS** (ESI) (m/z) $[M+H]^+$ Calcd for $C_{29}H_{27}N_2O_4^+$ 467.1965; found 467.1961. **HPLC**: The product was analyzed by HPLC to determine the enantiomeric excess: 91% ee (Chiralpak AD-H, *n*-hexane/*i*-propanol = 85/15, 1 mL/min, 254 nm) t_R = 11.372 min, 15.922 min.

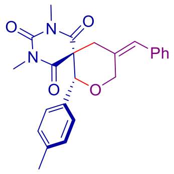

10

(*R*,*Z*)-10-benzylidene-2,4-dimethyl-7-(4-tolyl)-8-oxa-2,4-diazaspiro[5.5] undecane-1,3,5-trione

White solid. **Yield:** 94% yield. **MP:** 181.6 – 182.3 °C. $[\alpha]_D$ = + 68 (*c* = 0.226, CH_2Cl_2, 26.7 °C). **^{1}H NMR (600 MHz, $CDCl_3$)** *δ* 7.35 (t, *J* = 7.6 Hz, 2H), 7.27 – 7.24 (m, 1H, overlapped with the peak of chloroform), 7.22 (d, *J* = 7.5 Hz, 2H), 7.06 (d, *J* = 7.9 Hz, 2H), 6.94 (d, *J* = 8.0 Hz, 2H), 6.48 (s, 1H), 5.18 (d, *J* = 13.5 Hz, 1H), 4.86 (s, 1H), 4.47 (d, *J* = 13.6 Hz, 1H), 3.58 (d, *J* = 14.9 Hz, 1H), 3.11 (s, 3H), 2.98 (s, 3H), 2.83 (d, *J* = 14.9 Hz, 1H), 2.29 (s, 3H) ppm. **^{13}C NMR (151 MHz, $CDCl_3$)** *δ* 169.5, 167.5, 150.2, 139.2, 136.4, 132.7, 131.1, 129.0, 128.9, 128.3, 127.0, 126.8, 125.5, 85.1, 68.5, 58.1, 38.2, 28.5, 28.0, 21.2 ppm. IR (cm^-) 2969, 1673, 1378, 1160, 950, 734. **HRMS** (ESI) (m/z) $[M+H]^+$ Calcd for $C_{24}H_{25}N_2O_4^+$ 405.1809; found 405.1804. **HPLC**: The product was analyzed by HPLC to determine the enantiomeric excess: 91% ee (Chiralpak AD-H, *n*-hexane/*i*-propanol = 70/30, 1 mL/min, 254 nm) t_R = 6.522 min, 7.722 min.

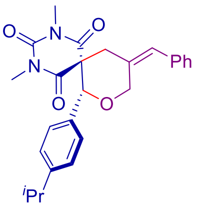

11

(*R*,*Z*)-10-benzylidene-7-(4-isopropylphenyl)-2,4-dimethyl-8-oxa-2,4-diazaspiro [5.5]undecane-1,3,5-trione

White solid. **Yield:** 86% yield. **MP:** 146.3 – 146.6 °C. $[\alpha]_D$ = + 211 (*c* = 0.053, CH_2Cl_2, 29.3 °C). **^{1}H NMR (600 MHz, $CDCl_3$)** δ 7.35 (t, *J* = 7.4 Hz, 2H), 7.25 (d, *J* = 6.5 Hz, 1H), 7.22 (d, *J* = 7.6 Hz, 2H), 7.11 (d, *J* = 7.9 Hz, 2H), 6.97 (d, *J* = 7.8 Hz, 2H), 6.48 (s, 1H), 5.18 (d, *J* = 13.6 Hz, 1H), 4.86 (s, 1H), 4.47 (d, *J* = 13.6 Hz, 1H), 3.58 (d, *J* = 14.9 Hz, 1H), 3.09 (s, 3H), 2.95 (s, 3H), 2.84 (d, *J* = 15.1 Hz, 2H), 1.19 (d, *J* = 6.9 Hz, 6H) ppm. **^{13}C NMR (151 MHz, $CDCl_3$)** *δ* 169.5, 167.5, 150.4, 150.1, 136.4, 133.0, 131.2, 129.1, 128.3, 127.0, 126.7, 126.1, 125.5, 85.1, 68.5, 58.3, 38.1, 33.9, 28.5, 27.9, 23.9, 23.9 ppm. IR (cm^-) 2968, 1673, 1378, 1160, 950, 816. **HRMS** (ESI) (m/z) $[M+H]^+$ Calcd for $C_{26}H_{29}N_2O_4^+$ 433.2122; found 433.2104. **HPLC**: The product was analyzed by HPLC to determine the enantiomeric excess: 90% ee

(Chiralpak AD-H, *n*-hexane/*i*-propanol = 70/30, 1 mL/min, 254 nm) t_R = 5.948 min, 7.036 min.

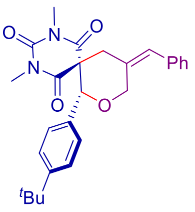

12

(*R*,*Z*)-10-benzylidene-7-(4-(tert-butyl)phenyl)-2,4-dimethyl-8-oxa-2,4-diazaspiro [5.5]undecane-1,3,5-trione

White solid. **Yield:** 86% yield. **MP:** 167.2 – 167.7 °C. $[\alpha]_D$ = + 69 (*c* = 0.185, CH_2Cl_2, 30.3 °C). **^{1}H NMR (600 MHz, $CDCl_3$)** δ 7.35 (t, *J* = 7.6 Hz, 2H), 7.29 – 7.25 (m, 3H, overlapped with the peak of chloroform), 7.24 – 7.21 (m, 2H), 6.98 (d, *J* = 8.3 Hz, 2H), 6.48 (s, 1H), 5.18 (d, *J* = 13.6 Hz, 1H), 4.87 (s, 1H), 4.48 (d, *J* = 13.6 Hz, 1H), 3.58 (d, *J* = 14.9 Hz, 1H), 3.09 (s, 3H), 2.94 (s, 3H), 2.84 (d, *J* = 14.9 Hz, 1H), 1.26 (s, 9H) ppm. **^{13}C NMR (151 MHz, $CDCl_3$)** δ 169.5, 167.5, 152.7, 150.1, 136.4, 132.6, 131.2, 129.1, 128.3, 127.0, 126.7, 125.3, 124.9, 85.1, 68.5, 58.3, 38.0, 34.6, 31.2, 28.5, 27.9 ppm. IR (cm^{-}) 2968, 1673, 1378, 1160, 950, 817. **HRMS** (ESI) (m/z) $[M+H]^+$ Calcd for $C_{27}H_{31}N_2O_4^+$ 447.2278; found 447.2276. **HPLC**: The product was analyzed by HPLC to determine the enantiomeric excess: 90% ee (Chiralpak AD-H, *n*-hexane/*i*-propanol = 85/15, 1 mL/min, 254 nm) t_R = 7.348 min, 7.974 min.

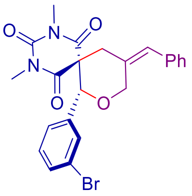

13

(*R*,*Z*)-10-benzylidene-7-(3-bromophenyl)-2,4-dimethyl-8-oxa-2,4-diazaspiro[5.5] undecane-1,3,5-trione

White solid. **Yield:** 81% yield. **MP:** 48.2 – 48.5 °C. $[\alpha]_D$ = + 99 (*c* = 0.100, CH_2Cl_2, 27.5 °C). **^{1}H NMR (600 MHz, $CDCl_3$)** δ 7.42 (d, *J* = 7.9 Hz, 1H), 7.36 (t, *J* = 7.6 Hz, 2H), 7.30 – 7.24 (m, 2H, overlapped with the peak of chloroform), 7.22 (d, *J* = 7.4 Hz, 2H), 7.13 (t, *J* = 7.9 Hz, 1H), 6.99 (d, *J* = 7.8 Hz, 1H), 6.49 (s, 1H), 5.18 (d, *J* =

13.5 Hz, 1H), 4.87 (s, 1H), 4.46 (d, *J* = 13.5 Hz, 1H), 3.58 (d, *J* = 14.8 Hz, 1H), 3.15 (s, 3H), 3.01 (s, 3H), 2.83 (d, *J* = 14.9 Hz, 1H) ppm. **^{13}C NMR (151 MHz, $CDCl_3$)** δ 169.2, 167.0, 150.1, 138.0, 136.2, 132.4, 130.5, 129.7, 129.0, 128.9, 128.3, 127.2, 127.1, 124.2, 122.6, 84.1, 68.4, 57.9, 38.2, 28.6, 28.0 ppm. IR (cm^{-}) 2969, 1673, 1378, 1160, 950, 816. **HRMS** (ESI) (m/z) $[M+H]^+$ Calcd for $C_{23}H_{21}BrN_2NaO_4^+$ 491.0577; found 491.0575. **HPLC**: The product was analyzed by HPLC to determine the enantiomeric excess: 90% ee (Chiralpak AD-H, *n*-hexane/*i*-propanol = 70/30, 1 mL/min, 254 nm) t_R = 6.702 min, 9.550 min.

14 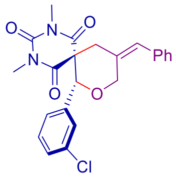

(*R*,*Z*)-10-benzylidene-7-(3-chlorophenyl)-2,4-dimethyl-8-oxa-2,4-diazaspiro[5.5] undecane-1,3,5-trione

White solid. **Yield:** 76% yield. **MP:** > 220 °C. $[\alpha]_D$ = + 80 (*c* = 0.090, CH_2Cl_2, 31.2 °C). **^{1}H NMR (600 MHz, $CDCl_3$)** δ 7.57 (d, *J* = 7.9 Hz, 2H), 7.37 (t, *J* = 7.3 Hz, 2H), 7.28 (d, *J* = 7.4 Hz, 1H), 7.24 – 7.21 (m, 4H), 6.49 (s, 1H), 5.18 (d, *J* = 13.5 Hz, 1H), 5.01 (s, 1H), 4.47 (d, *J* = 13.5 Hz, 1H), 3.54 (d, *J* = 14.7 Hz, 1H), 3.17 (s, 3H), 2.98 (s, 3H), 2.86 (d, *J* = 14.8 Hz, 1H) ppm. **^{13}C NMR (151 MHz, $CDCl_3$)** δ 169.1, 166.6, 149.9, 141.3, 136.1, 132.0, 129.9, 129.0, 128.4, 127.7 127.2, 126.7, 118.0, 113.2, 83.5, 68.3, 57.5, 39.0, 28.7, 28.0 ppm. IR (cm^{-}) 3000, 1683, 1264, 1161, 947, 735. **HRMS** (ESI) (m/z) $[M+H]^+$ Calcd for $C_{23}H_{21}ClN_2NaO_4^+$ 447.1082; found 447.1114. **HPLC**: The product was analyzed by HPLC to determine the enantiomeric excess: 90% ee (Chiralpak AD-H, *n*-hexane/*i*-propanol = 85/15, 1 mL/min, 254 nm) t_R = 7.936 min, 11.556 min.

15 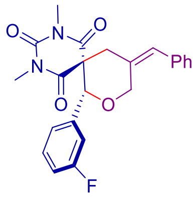

(*R*,*Z*)-10-benzylidene-7-(3-fluorophenyl)-2,4-dimethyl-8-oxa-2,4-diazaspiro[5.5] undecane-1,3,5-trione

White solid. **Yield:** 76% yield. **MP:** 61.6 – 61.9 °C. $[\alpha]_D$ = + 135 (*c* = 0.077, CH_2Cl_2, 30.8 °C). **^{1}H NMR (600 MHz, CDCl3)** δ 7.35 (t, *J* = 7.6 Hz, 2H), 7.28 – 7.24 (m, 1H, overlapped with the peak of chloroform), 7.24 – 7.20 (m, 3H), 7.01 – 6.97 (m, 1H), 6.85 (t, *J* = 9.3 Hz, 2H), 6.48 (s, 1H), 5.18 (d, *J* = 13.5 Hz, 1H), 4.91 (s, 1H), 4.46 (d, *J* = 13.5 Hz, 1H), 3.56 (d, *J* = 14.8 Hz, 1H), 3.14 (s, 3H), 2.99 (s, 3H), 2.83 (d, *J* = 14.9 Hz, 1H) ppm. **^{13}C NMR (151 MHz, $CDCl_3$)** δ 169.3, 167.0, 162.6 (d, J_{C-F} = 247.7 Hz), 150.2, 138.5 (d, J_{C-F} = 7.2 Hz), 136.3, 130.6, 129.8 (d, J_{C-F} = 8.2 Hz), 129.0, 128.3, 127.1, 127.1, 121.3 (d, J_{C-F} = 3.1 Hz), 116.3 (d, J_{C-F} = 21.0 Hz), 112.9 (d, J_{C-F} = 23.0 Hz), 84.0， 68.4, 57.8, 38.4, 28.6, 28.0 ppm. IR (cm^-) 2968, 1675, 1378, 1160, 950, 816. **HRMS** (ESI) (m/z) $[M+H]^+$ Calcd for $C_{23}H_{22}FN_2O_4^+$ 409.1558; found 409.1530. **HPLC**: The product was analyzed by HPLC to determine the enantiomeric excess: 90% ee (Chiralpak AD-H, *n*-hexane/*i*-propanol = 85/15, 1 mL/min, 254 nm) t_R = 8.392 min, 11.207 min.

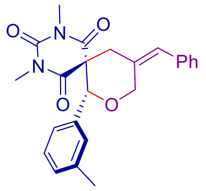

16

(*R*,*Z*)-10-benzylidene-7-(3-tolyl)-2,4-dimethyl-8-oxa-2,4-diazaspiro[5.5] undecane-1,3,5-trione

White solid. **Yield:** 83% yield. **MP:** 64.2 – 64.6 °C. $[\alpha]_D$ = + 162 (*c* = 0.068, CH_2Cl_2, 32.2 °C). **^{1}H NMR (600 MHz, $CDCl_3$)** δ 7.35 (t, *J* = 7.6 Hz, 2H), 7.27 – 7.24 (m, 1H, overlapped with the peak of chloroform), 7.22 (d, *J* = 7.4 Hz, 2H), 7.14 (t, *J* = 7.6 Hz, 1H), 7.10 (d, *J* = 7.6 Hz, 1H), 6.91 (s, 1H), 6.84 (d, *J* = 7.6 Hz, 1H), 6.49 (s, 1H), 5.19 (d, *J* = 13.5 Hz, 1H), 4.85 (s, 1H), 4.48 (d, *J* = 13.5 Hz, 1H), 3.60 (d, *J* = 14.9 Hz, 1H), 3.10 (s, 3H), 2.97 (s, 3H), 2.84 (d, *J* = 14.9 Hz, 1H), 2.28 (s, 3H) ppm. **^{13}C NMR (151 MHz, $CDCl_3$)** δ 169.4, 167.5, 150.2, 138.2, 136.4, 135.6, 131.1, 130.1, 129.0, 128.3, 128.0, 127.0, 126.8, 126.1, 122.7, 85.2, 68.5, 58.2, 38.1, 28.5, 27.9, 21.2 ppm. IR (cm^-) 2968, 1671, 1378, 1160, 950, 816. **HRMS** (ESI) (m/z) $[M+H]^+$

Calcd for $C_{24}H_{25}N_2O_4^+$ 405.1809; found 405.1800. **HPLC**: The product was analyzed by HPLC to determine the enantiomeric excess: 91% ee (Chiralpak AD-H, *n*-hexane/*i*-propanol = 70/30, 1 mL/min, 254 nm) t_R = 5.651 min, 7.627 min.

17 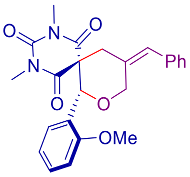

(*R*,*Z*)-10-benzylidene-7-(2-methoxyphenyl)-2,4-dimethyl-8-oxa-2,4-diazaspiro [5.5]undecane-1,3,5-trione

White solid. **Yield:** 95% yield. **MP:** 194.1 – 194.7 °C. $[\alpha]_D$ = + 66 (*c* = 0.090, CH_2Cl_2, 26.4 °C). **^{1}H NMR (600 MHz, $CDCl_3$)** δ 7.35 (t, *J* = 7.7 Hz, 2H), 7.28 – 7.25 (m, 1H, overlapped with the peak of chloroform), 7.25 – 7.23 (m, 2H), 7.22 (d, *J* = 8.2 Hz, 2H), 6.92 (t, *J* = 7.5 Hz, 1H), 6.79 (d, *J* = 8.2 Hz, 1H), 6.50 (s, 1H), 5.27 (s, 1H), 5.13 (d, *J* = 13.9 Hz, 1H), 4.54 (d, *J* = 13.9 Hz, 1H), 3.74 (s, 3H), 3.44 (d, *J* = 14.8 Hz, 1H), 3.13 (s, 3H), 3.05 (d, *J* = 14.9 Hz, 1H), 2.90 (s, 3H) ppm. **^{13}C NMR (151 MHz, $CDCl_3$)** *δ* 169.0, 168.3, 155.2, 150.7, 136.6, 132.0, 130.1, 129.0, 128.3, 127.3, 126.8, 126.3, 124.6, 120.6, 109.8, 77.9, 69.1, 58.3, 55.4, 37.5, 28.7, 28.0 ppm. IR (cm^-) 2969, 1673, 1378, 1159, 950, 816. **HRMS** (ESI) (m/z) $[M+H]^+$ Calcd for $C_{24}H_{25}N_2O_5^+$ 421.1758; found 421.1757. **HPLC**: The product was analyzed by HPLC to determine the enantiomeric excess: 97% ee (Chiralpak AD-H, *n*-hexane/*i*-propanol = 85/15, 1 mL/min, 254 nm) t_R = 10.669 min, 11.713 min.

18 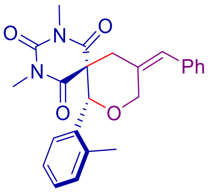

(*R*,*Z*)-10-benzylidene-2,4-dimethyl-7-(2-tolyl)-8-oxa-2,4-diazaspiro[5.5] undecane-1,3,5-trione

White solid. **Yield:** 76% yield. **MP:** 170.6 – 171.2 °C. $[\alpha]_D$ = + 64 (*c* = 0.050, CH_2Cl_2, 25.3 °C). **^{1}H NMR (600 MHz, $CDCl_3$)** *δ* 7.36 (t, *J* = 7.6 Hz, 2H), 7.28 – 7.24 (m, 1H, overlapped with the peak of chloroform), 7.23 (d, *J* = 7.5 Hz, 2H), 7.19

– 7.16 (m, 1H), 7.11 (d, *J* = 4.0 Hz, 2H), 7.09 (d, *J* = 7.6 Hz, 1H), 6.51 (s, 1H), 5.17 (d, *J* = 13.6 Hz, 1H), 5.14 (s, 1H), 4.51 (d, *J* = 13.6 Hz, 1H), 3.64 (d, *J* = 15.0 Hz, 1H), 3.08 (s, 3H), 3.05 (s, 3H), 2.89 (d, *J* = 15.0 Hz, 1H), 2.22 (s, 3H) ppm. **^{13}C NMR (151 MHz, $CDCl_3$)** δ 169.1, 167.9, 150.2, 136.5, 134.7, 133.8, 131.3, 130.6, 129.1, 129.0, 128.3, 127.0, 126.7, 126.5, 125.6, 81.3, 68.9, 57.7, 38.1, 28.7, 28.2, 19.2 ppm. IR (cm^{-}) 2950, 1678, 1362, 1264, 897, 734. **HRMS** (ESI) (m/z) [M+H]$^+$ Calcd for $C_{24}H_{25}N_2O_4^+$ 405.1809; found 405.1804. **HPLC**: The product was analyzed by HPLC to determine the enantiomeric excess: 90% ee (Chiralpak AD-H, *n*-hexane/*i*-propanol = 70/30, 1 mL/min, 254 nm) t_R = 5.934 min, 7.325 min.

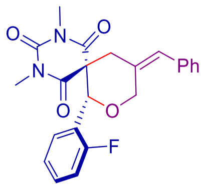

19

(*R*,*Z*)-10-benzylidene-7-(2-fluorophenyl)-2,4-dimethyl-8-oxa-2,4-diazaspiro[5.5] undecane-1,3,5-trione

White solid. **Yield:** 76% yield. **MP:** 72.6 – 73.3 °C. $[\alpha]_D$ = - 6 (*c* = 0.054, CH_2Cl_2, 24.8 °C). **^{1}H NMR (600 MHz, $CDCl_3$)** δ 7.35 (t, *J* = 7.6 Hz, 2H), 7.30 – 7.23 (m, 3H, overlapped with the peak of chloroform), 7.21 (d, *J* = 7.6 Hz, 2H), 7.10 (t, *J* = 7.6 Hz, 1H), 7.00 – 6.96 (m, 1H), 6.52 (s, 1H), 5.18 (s, 1H), 5.15 (d, *J* = 13.6 Hz, 1H), 4.50 (d, *J* = 13.6 Hz, 1H), 3.60 (d, *J* = 14.9 Hz, 1H), 3.16 (s, 3H), 2.96 – 2.90 (m, 4H) ppm. **^{13}C NMR (151 MHz, $CDCl_3$)** δ 168.4, 167.6, 158.7 (d, $J_{C\text{-}F}$ = 247.1 Hz), 150.3, 136.4, 131.0, 130.9 (d, $J_{C\text{-}F}$ = 8.3 Hz), 129.0, 128.3, 127.9 (d, $J_{C\text{-}F}$ = 3.2 Hz), 127.0, 126.9, 124.2 (d, $J_{C\text{-}F}$ = 3.2 Hz), 123.3, 123.2, 115.0 (d, $J_{C\text{-}F}$ = 21.7 Hz), 77.7, 68.8, 58.0, 37.4, 28.8, 28.0 ppm. IR (cm^{-}) 2968, 1673, 1378, 1160, 950, 917. **HRMS** (ESI) (m/z) [M+H]$^+$ Calcd for $C_{23}H_{22}FN_2O_4^+$ 409.1558; found 409.1554. **HPLC**: The product was analyzed by HPLC to determine the enantiomeric excess: 90% ee (Chiralpak AD-H, *n*-hexane/*i*-propanol = 85/15, 1 mL/min, 254 nm) t_R = 9.098 min, 9.788 min.

20 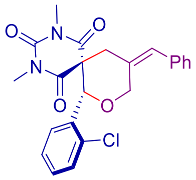

(*R*,*Z*)-10-benzylidene-7-(2-chlorophenyl)-2,4-dimethyl-8-oxa-2,4-diazaspiro[5.5] undecane-1,3,5-trione

White solid. **Yield:** 76% yield. **MP:** 161.3 – 161.9 °C. $[\alpha]_D$ = - 15 (*c* = 0.055, CH_2Cl_2, 24.5 °C). **^{1}H NMR (600 MHz, $CDCl_3$)** δ 7.36 (t, *J* = 7.7 Hz, 2H), 7.31 – 7.28 (m, 2H), 7.29 – 7.25 (m, 1H, overlapped with the peak of chloroform), 7.25 – 7.21 (m, 4H), 6.54 (s, 1H), 5.35 (s, 1H), 5.14 (d, *J* = 13.7 Hz, 1H), 4.55 (d, *J* = 13.8 Hz, 1H), 3.58 (s, 1H), 3.18 (s, 3H), 3.01 (d, *J* = 15.5 Hz, 1H), 2.95 (s, 3H) ppm. **^{13}C NMR (151 MHz, $CDCl_3$)** δ 168.4, 167.8, 150.4, 136.4, 133.9, 131.9, 131.2, 130.4, 129.3, 129.0, 128.6, 128.3, 127.0, 126.8, 126.6, 79.9, 69.0, 57.9, 37.5, 29.3, 28.1 ppm. IR (cm^-) 2968, 1768, 1378, 1159, 950, 816. **HRMS** (ESI) (m/z) $[M+H]^+$ Calcd for $C_{23}H_{21}ClN_2NaO_4^+$ 447.1082; found 447.1080. **HPLC**: The product was analyzed by HPLC to determine the enantiomeric excess: 94% ee (Chiralpak AD-H, *n*-hexane/*i*-propanol = 70/30, 1 mL/min, 254 nm) t_R = 5.948 min, 7.036 min.

21 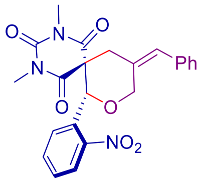

(*R*,*Z*)-10-benzylidene-7-(2-nitrophenyl)-2,4-dimethyl-8-oxa-2,4-diazaspiro[5.5] undecane-1,3,5-trione

Yellow oil. **Yield:** 71% yield. $[\alpha]_D$ = + 157 (*c* = 0.150, CH_2Cl_2, 33.9 ℃). **^{1}H NMR (600 MHz, $CDCl_3$)** δ 7.88 – 7.83 (m, 1H), 7.73 – 7.68 (m, 1H), 7.63 – 7.58 (m, 1H), 7.49 – 7.44 (m, 1H), 7.39 – 7.34 (m, 2H), 7.28 – 7.25 (m, 1H, overlapped with the peak of chloroform), 7.24 – 7.19 (m, 2H), 6.44 (s, 1H), 5.75 – 5.67 (m, 1H), 5.11 (d, *J* = 11.7 Hz, 1H), 4.70 (d, *J* = 13.7 Hz, 1H), 3.27 (d, *J* = 14.1 Hz, 1H), 3.22 – 3.17 (m, 3H), 3.13 (d, *J* = 14.6 Hz, 1H), 2.99 – 2.93 (m, 3H) ppm. **^{13}C NMR (151 MHz, $CDCl_3$)** δ 168.9, 167.8, 150.4, 147.3, 136.3, 132.8, 131.8, 131.3, 129.8, 129.5, 128.9,

128.4, 127.0, 126.7, 124.3, 78.2, 69.0, 58.0, 39.1, 29.0, 28.3 ppm. IR (cm^{-}) 2969, 1678, 1467, 1378, 1160, 951, 817. **HRMS** (ESI) (m/z) $[M+H]^+$ Calcd for $C_{23}H_{21}N_3NaO_6^+$ 458.1323; found 458.1328. **HPLC**: The product was analyzed by HPLC to determine the enantiomeric excess: 90% ee (Chiralpak AD-H, *n*-hexane/*i*-propanol = 85/15, 1 mL/min, 254 nm) t_R = 17.903 min, 23.666 min.

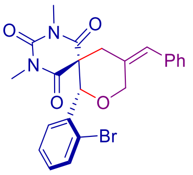

22

(*R*,*Z*)-10-benzylidene-7-(2-nitrophenyl)-2,4-dimethyl-8-oxa-2,4-diazaspiro[5.5] undecane-1,3,5-trione

White solid. **Yield:** 93% yield. **MP:** 152.9 – 153.3 °C. $[\alpha]_D$ = - 5 (*c* = 0.100, CH_2Cl_2, 22.8 °C). **^{1}H NMR (600 MHz, $CDCl_3$)** δ 7.49 (d, *J* = 7.9 Hz, 1H), 7.36 (t, *J* = 7.6 Hz, 2H), 7.28 – 7.24 (m, 3H, overlapped with the peak of chloroform), 7.22 (d, *J* = 7.6 Hz, 2H), 7.18 – 7.14 (m, 1H), 6.53 (s, 1H), 5.34 (s, 1H), 5.12 (d, *J* = 13.8 Hz, 1H), 4.56 (d, *J* = 13.8 Hz, 1H), 3.55 (d, *J* = 14.8 Hz, 1H), 3.20 (s, 3H), 3.02 (d, *J* = 15.0 Hz, 1H), 2.96 (s, 3H) ppm. **^{13}C NMR (151 MHz, $CDCl_3$)** δ 168.3, 167.8, 150.4, 136.4, 135.6, 132.7, 131.2, 130.6, 129.0, 129.0, 128.3, 127.0, 127.0, 126.8, 121.9, 82.1, 69.0, 57.9, 37.6, 29.4, 28.2 ppm. IR (cm^{-}) 3052, 2950, 1556, 1354, 1265, 970, 737. **HRMS** (ESI) (m/z) $[M+H]^+$ Calcd for $C_{23}H_{21}BrN_2NaO_4^+$ 491.0577; found 491.0576. **HPLC**: The product was analyzed by HPLC to determine the enantiomeric excess: 85% ee (Chiralpak AD-H, *n*-hexane/*i*-propanol = 85/15, 1 mL/min, 254 nm) t_R = 8.767 min, 9.979 min.

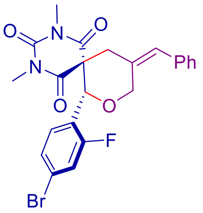

23

(*R*,*Z*)-10-benzylidene-7-(4-bromo-2-fluorophenyl)-2,4-dimethyl-8-oxa-2,4-diazaspiro[5.5]undecane-1,3,5-trione

White solid. **Yield:** 76% yield. **MP:** 117.7 – 118.4 °C. $[\alpha]_D$ = + 104 (*c* = 0.055, CH_2Cl_2, 32.3 °C). **^{1}H NMR (600 MHz, $CDCl_3$)** δ 7.36 (t, *J* = 7.6 Hz, 2H), 7.28 – 7.24 (m, 2H, overlapped with the peak of chloroform), 7.21 (d, *J* = 7.5 Hz, 2H), 7.19 – 7.15 (m, 2H), 6.52 (s, 1H), 5.14 (d, *J* = 10.8 Hz, 2H), 4.49 (d, *J* = 13.6 Hz, 1H), 3.55 (d, *J* = 14.9 Hz, 1H), 3.19 (s, 3H), 2.97 (s, 3H), 2.94 (d, *J* = 15.0 Hz, 1H) ppm. **^{13}C NMR (151 MHz, $CDCl_3$)** δ 168.3, 167.4, 158.4 (d, J_{C-F} = 251.5 Hz), 150.3, 136.2, 130.6, 129.2 (d, J_{C-F} = 4.0 Hz), 129.0, 128.3, 127.7, 127.7, 127.2, 127.1, 123.5 (d, J_{C-F} = 9.5 Hz), 122.7 (d, J_{C-F} = 13.9 Hz), 118.6 (d, J_{C-F} = 25.1 Hz), 77.2, 68.8, 57.6, 37.7, 28.9, 28.1 ppm. IR (cm^{-}) 2950, 1742, 1676, 1370, 1264, 1154, 745, 735. **HRMS** (ESI) (m/z) $[M+H]^+$ Calcd for $C_{23}H_{21}BrFN_2O_4^+$ 487.0663; found 487.0670. **HPLC**: The product was analyzed by HPLC to determine the enantiomeric excess: 90% ee (Chiralpak AD-H, *n*-hexane/*i*-propanol = 85/15, 1 mL/min, 254 nm) t_R = 10.738 min, 12.446 min.

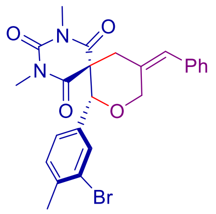

24

(*R*,*Z*)-10-benzylidene-7-(3-bromo-4-methylphenyl)-2,4-dimethyl-8-oxa-2,4-diazaspiro[5.5]undecane-1,3,5-trione

White solid. **Yield:** 76% yield. **MP:** 124.7 – 125.1 °C. $[\alpha]_D$ = + 31 (*c* = 0.120, CH_2Cl_2, 33.1 °C). **^{1}H NMR (600 MHz, $CDCl_3$)** δ 7.35 (t, *J* = 7.5 Hz, 2H), 7.30 (s, 1H), 7.28 – 7.24 (m, 1H, overlapped with the peak of chloroform), 7.21 (d, *J* = 7.5 Hz, 2H), 7.11 (d, *J* = 7.8 Hz, 1H), 6.89 (d, *J* = 7.8 Hz, 1H), 6.48 (s, 1H), 5.17 (d, *J* = 13.5 Hz, 1H), 4.84 (s, 1H), 4.45 (d, *J* = 13.5 Hz, 1H), 3.57 (d, *J* = 14.8 Hz, 1H), 3.15 (s, 3H), 3.02 (s, 3H), 2.82 (d, *J* = 14.9 Hz, 1H), 2.34 (s, 3H) ppm. **^{13}C NMR (151 MHz, $CDCl_3$)** δ 169.3, 167.2, 150.2, 139.1, 136.3, 135.1, 130.7, 130.4, 129.6, 129.0, 128.3, 127.1, 124.8, 124.5, 84.1, 68.5, 57.9, 38.2, 28.6, 28.0, 22.7 ppm. IR (cm^{-}) 2968, 1768, 1378, 1160, 951, 817. **HRMS** (ESI) (m/z) $[M+H]^+$ Calcd for $C_{24}H_{24}BrN_2O_4^+$ 483.0914; found 483.0907. **HPLC**: The product was analyzed by

HPLC to determine the enantiomeric excess: 91% ee (Chiralpak AD-H, *n*-hexane/*i*-propanol = 85/15, 1 mL/min, 254 nm) t_R = 8.220 min, 10.772 min.

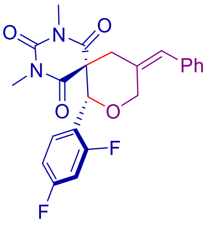

25

(*R*,*Z*)-10-benzylidene-7-(2,4-difluorophenyl)-2,4-dimethyl-8-oxa-2,4-diazaspiro [5.5]undecane-1,3,5-trione

White solid. **Yield:** 71% yield. **MP:** 188.8 – 189.2 °C. $[\alpha]_D$ = + 118 (*c* = 0.055, CH_2Cl_2, 31.8 °C). **^{1}H NMR (600 MHz, $CDCl_3$)** δ 7.36 (t, *J* = 7.6 Hz, 2H), 7.28 – 7.24 (m, 3H, overlapped with the peak of chloroform), 7.21 (d, *J* = 7.5 Hz, 2H), 6.86 – 6.82 (m, 1H), 6.77 – 6.72 (m, 1H), 6.52 (s, 1H), 5.17 – 5.13 (m, 2H), 4.49 (d, *J* = 13.6 Hz, 1H), 3.58 (d, *J* = 14.9 Hz, 1H), 3.19 (s, 2H), 2.98 (s, 2H), 2.93 (d, *J* = 15.0 Hz, 1H) ppm. **^{13}C NMR (151 MHz, $CDCl_3$)** δ 168.3, 167.5, 163.2(dd, $J_{C\text{-}F}$ = 255.2 Hz), 158.9 (dd, $J_{C\text{-}F}$ = 250.7 Hz), 150.3, 136.3, 130.7, 129.2 (q, $J_{C\text{-}F}$ = 9.3 Hz), 129.0, 128.3, 127.1(d, $J_{C\text{-}F}$ =11.5 Hz), 119.5 (dd, $J_{C\text{-}F}$ =13.6 Hz), 111.7 (dd, $J_{C\text{-}F}$ = 18.2 Hz), 103.4, 103.39 (d, $J_{C\text{-}F}$ = 26.7 Hz), 68.8, 57.8, 37.5,29.7, 28.8, 28.1 ppm. IR (cm^-) 2968, 1768, 1378, 1160, 951, 816, 739. **HRMS** (ESI) (m/z) $[M+H]^+$ Calcd for $C_{23}H_{20}F_2N_2NaO_4^+$ 449.1283; found 449.1276. **HPLC**: The product was analyzed by HPLC to determine the enantiomeric excess: 94% ee (Chiralpak AD-H, *n*-hexane/*i*-propanol = 98/2, 1 mL/min, 254 nm) t_R = 33.712 min, 36.197 min.

(*Z*)-10-benzylidene-2,4-dimethyl-7-(thiophen-3-yl)-8-oxa-2,4-diazaspiro[5.5] undecane-1,3,5-trione 26.

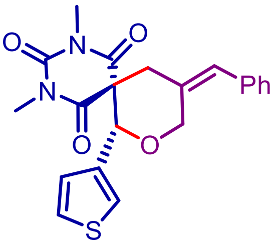

27

White solid. **Yield:** 96% yield. **MP:** 183.6 – 184.5 ℃. $[\alpha]_D$ = + 121 (*c* = 0.090, CH_2Cl_2, 30.3 ℃). **^{1}H NMR (600 MHz, $CDCl_3$)** δ 7.35 (t, *J* = 7.5 Hz, 2H), 7.27 – 7.24 (m, 2H, overlapped with the peak of chloroform), 7.21 (d, *J* = 7.6 Hz, 2H), 7.11 – 7.08 (m, 1H), 6.77 (d, *J* = 4.9 Hz, 1H), 6.48 (s, 1H), 5.17 (d, *J* = 13.5 Hz, 1H), 5.04 (s, 1H), 4.45 (d, *J* = 13.8 Hz, 1H), 3.54 (d, *J* = 14.8 Hz, 1H), 3.17 (s, 3H), 3.03 (s, 3H), 2.83 (d, *J* = 14.9 Hz, 1H). **^{13}C NMR (151 MHz, $CDCl_3$)** δ 169.50, 167.41, 150.34, 137.02, 136.29, 130.74, 129.04, 128.31, 127.04, 126.49, 124.72, 122.25, 81.56, 68.48, 57.83, 38.34, 28.62, 28.14. IR (cm^-) 2969, 1671, 1378, 1160, 950, 816. **HRMS** (ESI) (m/z) $[M+H]^+$ calculated for $C_{21}H_{21}N_2O_4S^+$ 397.1217; found 397.1228 **HPLC** analysis: 70% ee (HPLC condition: Chiralpak AD-H column, *n*-hexane/*i*-propanol = 85/15, 1.0 mL/min, λ = 254 nm) t_R = 11.217 min, 14.272 min.

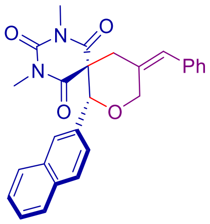

27

(*R*,*Z*)-10-benzylidene-2,4-dimethyl-7-(naphthalen-2-yl)-8-oxa-2,4-diazaspiro[5.5] undecane-1,3,5-trione

White solid. **Yield:** 96% yield. **MP:** 90.3 – 90.6 °C. $[\alpha]_D$ = + 99 (*c* = 0.133, CH_2Cl_2, 27.3 °C). **^{1}H NMR (600 MHz, $CDCl_3$)** δ 7.80 – 7.77 (m, 1H), 7.75 – 7.72 (m, 2H), 7.58 (s, 1H), 7.49 – 7.44 (m, 2H), 7.37 (t, *J* = 7.6 Hz, 2H), 7.28 – 7.23 (m, 3H, overlapped with the peak of chloroform), 7.15 (d, 1H), 6.51 (s, 1H), 5.25 (d, *J* = 13.6 Hz, 1H), 5.08 (s, 1H), 4.53 (d, *J* = 13.6 Hz, 1H), 3.67 – 3.62 (m, 1H), 3.07 (s, 3H), 2.89 (d, 4H) ppm. **^{13}C NMR (151 MHz, $CDCl_3$)** δ 169.5, 167.4, 150.0, 136.4, 133.5, 133.1, 132.8, 131.0, 129.1, 128.3, 128.0, 127.9, 127.8, 127.0, 126.9, 126.7, 126.7, 125.2, 122.9, 85.1, 68.6, 58.2, 38. 4, 28.5, 28.0 ppm. IR (cm^-) 2968, 1678, 1340, 1168, 950, 816. **HRMS** (ESI) (m/z) $[M+H]^+$ Calcd for $C_{27}H_{25}N_2O_4^+$ 441.1809; found 441.1807. **HPLC**: The product was analyzed by HPLC to determine the enantiomeric excess: 92% ee (Chiralpak AD-H, *n*-hexane/*i*-propanol = 85/15, 1 mL/min, 254 nm) t_R = 12.305 min, 16.899 min.

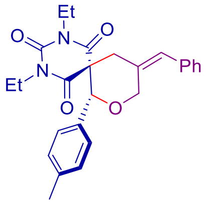

28

(*R*,*Z*)-10-benzylidene-2,4-diethyl-7-(4-tolyl)-8-oxa-2,4-diazaspiro[5.5]undecane-1,3,5-trione

White solid. **Yield:** 88% yield. **MP:** 124.6 – 125.2 °C. $[\alpha]_D$ = + 298 (*c* = 0.055, CH_2Cl_2, 33.8 °C). **^{1}H NMR (600 MHz, $CDCl_3$)** δ 7.34 (t, *J* = 7.6 Hz, 2H), 7.23 (t, *J* = 8.2 Hz, 3H), 7.04 – 6.99 (m, 4H), 6.44 (s, 1H), 5.18 (d, *J* = 13.5 Hz, 1H), 4.91 (s, 1H), 4.47 (d, *J* = 13.6 Hz, 1H), 3.80 – 3.74 (m, 2H), 3.67 – 3.62 (m, 2H), 3.51 (d, *J* = 14.7 Hz, 1H), 2.82 (d, *J* = 14.8 Hz, 1H), 2.27 (s, 3H), 1.11 (t, *J* = 7.1 Hz, 3H), 1.00 (t, *J* = 7.1 Hz, 3H) ppm. **^{13}C NMR (151 MHz, $CDCl_3$)** δ 169.5, 167.1, 149.6, 138.9, 136.4, 132.8, 131.2, 129.1, 128.9, 128.3, 126.9, 126.7, 126.2, 84.6, 68.4, 57.4, 39.2, 37.5, 37.1, 21.2, 13.0, 12.9 ppm. IR (cm^{-}) 2920, 1742, 1671, 1376, 1199, 949, 749. **HRMS** (ESI) (m/z) $[M+H]^+$ Calcd for $C_{26}H_{29}N_2O_4^+$ 433.2122; found 433.2116. **HPLC**: The product was analyzed by HPLC to determine the enantiomeric excess: 92% ee (Chiralpak IA-H, *n*-hexane/*i*-propanol = 95/5, 1 mL/min, 254 nm) t_R = 8.843 min, 9.563 min.

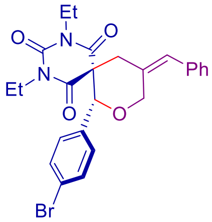

29

(*R*,*Z*)-10-benzylidene-2,4-diethyl-7-(4-bromophenyl)-8-oxa-2,4-diazaspiro[5.5] undecane-1,3,5-trione

White solid. **Yield:** 80% yield. **MP:** 58.6 – 58.9 °C. $[\alpha]_D$ = + 63 (*c* = 0.072, CH_2Cl_2, 24.3 °C). **^{1}H NMR (600 MHz, $CDCl_3$)** δ 7.39 – 7.34 (m, 4H), 7.28 – 7.24 (m, 1H, overlapped with the peak of chloroform), 7.22 (d, *J* = 7.5 Hz, 2H), 7.02 (d, *J* = 8.4 Hz, 2H), 6.45 (s, 1H), 5.17 (d, *J* = 13.5 Hz, 1H), 4.96 (s, 1H), 4.46 (d, *J* = 13.5 Hz,

1H), 3.84 – 3.77 (m, 2H), 3.71 – 3.63 (m, 2H), 3.48 (d, *J* = 14.6 Hz, 1H), 2.82 (d, *J* = 14.7 Hz, 1H), 1.13 (t, *J* = 7.1 Hz, 3H), 0.99 (t, *J* = 7.1 Hz, 3H) ppm. **^{13}C NMR (151 MHz, $CDCl_3$)** δ 169.3, 166.6, 149.5, 136.2, 135.1, 131.4, 130.5, 129.0, 128.3, 128.1, 127.2, 127.1, 123.1, 83.5, 68.3, 57.0, 39.6, 37.6, 37.1, 13.1, 13.0 ppm. IR (cm^-) 2952, 1673, 1363, 1246, 1160, 947, 815, 734. **HRMS** (ESI) (m/z) $[M+H]^+$ Calcd for $C_{25}H_{26}BrN_2O_4^+$ 497.1070; found 497.1062. **HPLC**: The product was analyzed by HPLC to determine the enantiomeric excess: 94% ee (Chiralpak IG-H, *n*-hexane/*i*-propanol = 95/5, 1 mL/min, 254 nm) t_R = 16.144 min, 18.690 min.

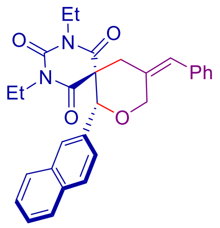

30

(*R*,*Z*)-10-benzylidene-2,4-diethyl-7-(naphthalen-2-yl)-8-oxa-2,4-diazaspiro[5.5] undecane-1,3,5-trione

White solid. **Yield:** 73% yield. **MP:** 132.1 – 132.7 °C. $[\alpha]_D$ = + 54 (*c* = 0.070, CH_2Cl_2, 24.3 °C). **^{1}H NMR (600 MHz, $CDCl_3$)** δ 7.78 – 7.75 (m, 1H), 7.73 – 7.67 (m, 2H), 7.62 (s, 1H), 7.47 – 7.43 (m, 2H), 7.37 (t, *J* = 7.6 Hz, 2H), 7.26 (d, *J* = 18.1 Hz, 3H), 7.23 – 7.20 (m, 1H), 6.48 (s, 1H), 5.26 (d, *J* = 13.6 Hz, 1H), 5.15 (s, 1H), 4.54 (d, *J* = 13.6 Hz, 1H), 3.77 – 3.72 (m, 2H), 3.61 – 3.54 (m, 3H), 2.87 (d, *J* = 14.7 Hz, 1H), 1.05 (t, *J* = 7.1 Hz, 3H), 0.86 (t, *J* = 7.1 Hz, 3H) ppm. **^{13}C NMR (151 MHz, $CDCl_3$)** δ 169.5, 167.0, 149.4, 136.4, 133.4, 133.3, 132.7, 131.0, 129.1, 128.3, 128.1, 128.0, 127.8, 127.0, 127.0, 126.5, 126.5, 125.8, 123.7, 84.7, 68.5, 57.5, 39.5, 37.6, 37.0, 13.0, 12.8 ppm. IR (cm^-) 2968, 1678, 1340, 1168, 950, 816. **HRMS** (ESI) (m/z) $[M+H]^+$ Calcd for $C_{29}H_{29}N_2O_4^+$ 469.2122; found 469.2119. **HPLC**: The product was analyzed by HPLC to determine the enantiomeric excess: 93% ee (Chiralpak IA-H, *n*-hexane/*i*-propanol = 95/5, 1 mL/min, 254 nm) t_R = 12.218 min, 14.826 min.

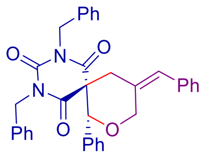

31

(*R*,*Z*)-2,4-dibenzyl-10-benzylidene-7-phenyl-8-oxa-2,4-diazaspiro[5.5]undecane-1,3,5-trione

Yellow oil. **Yield:** 59% yield. $[\alpha]_D$ = + 121 (*c* = 0.120, CH_2Cl_2, 34.3 ℃). **^{1}H NMR (600 MHz, $CDCl_3$)** δ 7.37 – 7.34 (m, 4H), 7.33 – 7.31 (m, 3H), 7.30 – 7.28 (m, 2H), 7.28 – 7.24 (m, 4H, overlapped with the peak of chloroform), 7.22 (d, *J* = 7.5 Hz, 2H), 7.11 – 7.07 (m, 1H), 6.82 (d, *J* = 4.5 Hz, 4H), 6.43 (s, 1H), 5.18 (d, *J* = 13.5 Hz, 1H), 4.91 (d, *J* = 15.1 Hz, 2H), 4.83 – 4.75 (m, 2H), 4.65 (d, *J* = 13.9 Hz, 1H), 4.46 (d, *J* = 13.5 Hz, 1H), 3.49 (d, *J* = 14.6 Hz, 1H), 2.84 (d, *J* = 14.7 Hz, 1H) ppm. **^{13}C NMR (151 MHz, $CDCl_3$)** δ 169.5, 166.9, 150.3, 136.3, 136.0, 135.7, 135.4, 130.7, 129.6, 129.5, 129.1, 128.7, 128.5, 128.4, 128.3, 128.2, 128.0, 127.8, 127.2, 127.1, 126.0, 84.4, 68.4, 57.6, 45.5, 45.0, 39.6 ppm. IR (cm^-) 2968, 1768, 1378, 1160, 952, 817. **HRMS** (ESI) (m/z) $[M+H]^+$ Calcd for $C_{35}H_{31}N_2O_4^+$ 543.2278; found 543.2270. **HPLC**: The product was analyzed by HPLC to determine the enantiomeric excess: 88% ee (Chiralpak AD-H, *n*-hexane/*i*-propanol = 95/5, 1 mL/min, 254 nm) t_R = 14.148 min, 15.610 min.

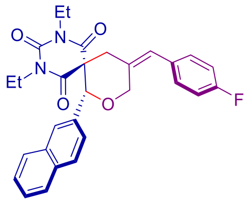

32

(*R*,*Z*)-2,4-diethyl-10-(4-fluorobenzylidene)-7-phenyl-8-oxa-2,4-diazaspiro[5.5] undecane-1,3,5-trione

White solid. **Yield:** 76% yield. **MP:** 142.4 – 142.8 °C. $[\alpha]_D$ = + 90 (*c* = 0.080, CH_2Cl_2, 23.9 °C). **^{1}H NMR (600 MHz, $CDCl_3$)** δ 7.79 – 7.75 (m, 1H), 7.73 – 7.68 (m, 2H), 7.62 (s, 1H), 7.48 – 7.42 (m, 2H), 7.24 – 7.19 (m, 3H), 7.06 (t, *J* = 8.6 Hz, 2H), 6.43 (s, 1H), 5.19 (d, *J* = 13.5 Hz, 1H), 5.14 (s, 1H), 4.51 (d, *J* = 13.6 Hz, 1H), 3.78 – 3.71 (m, 2H), 3.63 – 3.52 (m, 3H), 2.85 (d, *J* = 14.7 Hz, 1H), 1.05 (t, *J* = 7.1 Hz, 3H), 0.87 (t, *J* = 7.1 Hz, 3H) ppm. **^{13}C NMR (151 MHz, $CDCl_3$)** δ 169.4, 167.0, 161.8 (d, J_{C-F} = 246.8 Hz), 149.4, 133.4, 133.1, 132.7, 132.4 (d, J_{C-F} = 3.3 Hz), 131.1, 130.7 (d, J_{C-F} = 8.1 Hz), 128.1, 128.0, 127.8, 126.6, 123.6, 115.2 (d, J_{C-F} = 21.4 Hz),

84.7, 68.3, 57.4, 39.3, 37.6, 37.0, 13.0, 12.8 ppm. IR (cm^{-}) 2968, 1678, 1340, 1168, 950, 816. **HRMS** (ESI) (m/z) $[M+H]^+$ Calcd for $C_{29}H_{28}FN_2O_4^+$ 487.2028; found 487.2052. **HPLC**: The product was analyzed by HPLC to determine the enantiomeric excess: 93% ee (Chiralpak AD-H, *n*-hexane/*i*-propanol = 85/15, 1 mL/min, 254 nm) t_R = 11.272 min, 14.305 min.

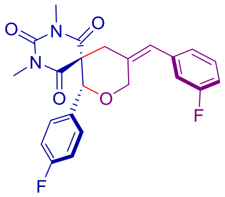

33

(*R*,*Z*)-10-(4-chlorobenzylidene)-2,4-dimethyl-7-phenyl-8-oxa-2,4-diazaspiro[5.5] undecane-1,3,5-trione

White solid. **Yield:** 81% yield. **MP:** 208.8 – 209.2 °C. $[\alpha]_D$ = + 16 (*c* = 0.080, CH_2Cl_2, 21.7 °C). **^{1}H NMR (600 MHz, $CDCl_3$)** δ 7.35 – 7.30 (m, 1H), 7.09 – 7.05 (m, 2H), 7.01 – 6.91 (m, 5H), 6.43 (s, 1H), 5.14 (d, *J* = 13.6 Hz, 1H), 4.90 (s, 1H), 4.45 (d, *J* = 13.6 Hz, 1H), 3.57 (d, *J* = 14.9 Hz, 1H), 3.13 (s, 3H), 3.00 (s, 3H), 2.82 (d, *J* = 14.9 Hz, 1H) ppm. **^{13}C NMR (151 MHz, $CDCl_3$)** δ 169.2, 167.1, 163.0 (d, J_{C-F} = 250.7 Hz), 162.7 (d, J_{C-F} = 246.13 Hz), 150.1, 138.4 (d, J_{C-F} = 7.8 Hz), 132.1, 131.7(d, J_{C-F} = 2.9 Hz), 129.8 (d, J_{C-F} = 8.4 Hz), 127.5 (d, J_{C-F} = 8.2 Hz), 125.8(d, J_{C-F} = 3.0 Hz), 124.8 (d, J_{C-F} = 3.0 Hz), 115.8 (d, J_{C-F} = 21.5 Hz), 115.3 (d, J_{C-F} = 21.6 Hz), 113.4 (d, J_{C-F} = 21.1 Hz), 84.2, 68.3, 57.9, 38.3, 28.6, 28.0 ppm. IR (cm^{-}) 3003, 2950, 1743, 1676, 1264, 1154, 945, 734. **HRMS** (ESI) (m/z) $[M+H]^+$ Calcd for $C_{23}H_{20}NaF_2N_2O_4^+$ 449.1283; found 449.1297. **HPLC**: The product was analyzed by HPLC to determine the enantiomeric excess: 90% ee (Chiralpak AD-H, *n*-hexane/*i*-propanol = 85/15, 1 mL/min, 254 nm) t_R = 10.669 min, 11.910 min.

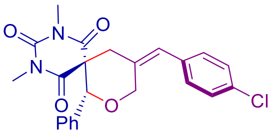

34

(*R*,*Z*)-10-(4-chlorobenzylidene)-2,4-dimethyl-7-phenyl-8-oxa-2,4-diazaspiro[5.5] undecane-1,3,5-trione

White solid. **Yield:** 64% yield. **MP:** 155.4 – 155.8 °C. $[\alpha]_D$ = + 113 (*c* = 0.108, CH_2Cl_2, 28.3 °C). **^{1}H NMR (600 MHz, $CDCl_3$)** δ 7.36 (t, *J* = 7.6 Hz, 2H), 7.30 – 7.25 (m, 4H, overlapped with the peak of chloroform), 7.22 (d, *J* = 7.6 Hz, 2H), 7.07 (d, *J* = 7.0 Hz, 2H), 6.49 (s, 1H), 5.19 (d, *J* = 13.5 Hz, 1H), 4.90 (s, 1H), 4.48 (d, *J* = 13.5 Hz, 1H), 3.59 (d, *J* = 14.9 Hz, 1H), 3.10 (s, 3H), 2.96 (s, 3H), 2.84 (d, *J* = 14.9 Hz, 1H) ppm. **^{13}C NMR (151 MHz, $CDCl_3$)** δ 169.4, 167.4, 150.2, 136.4, 135.7, 131.0, 129.4, 129.0, 128.3, 128.2, 127.0, 126.8, 125.6, 85.1, 68.5, 58.1, 38.2, 28.5, 28.0 ppm. IR (cm^-) 2949, 1680, 1353, 1264, 1147, 735. **HRMS** (ESI) (m/z) $[M+H]^+$ Calcd for $C_{23}H_{21}ClN_2NaO_4^+$ 447.1082; found 447.1076. **HPLC**: The product was analyzed by HPLC to determine the enantiomeric excess: 92% ee (Chiralpak AD-H, *n*-hexane/*i*-propanol = 70/30, 1 mL/min, 254 nm) t_R = 6.520 min, 7.995 min.

35 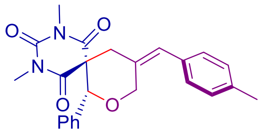

(*R*,*Z*)-2,4-dimethyl-10-(4-methylbenzylidene)-7-phenyl-8-oxa-2,4-diazaspiro[5.5] undecane-1,3,5-trione

White oil. **Yield:** 59% yield. $[\alpha]_D$ = + 70 (*c* = 0.160, CH_2Cl_2, 29.5 ℃). **^{1}H NMR (600 MHz, $CDCl_3$)** δ 7.30 – 7.24 (m, 3H, overlapped with the peak of chloroform), 7.16 (d, *J* = 7.8 Hz, 2H), 7.11 (d, *J* = 7.8 Hz, 2H), 7.06 (d, *J* = 7.2 Hz, 2H), 6.45 (s, 1H), 5.19 (d, *J* = 13.5 Hz, 1H), 4.89 (s, 1H), 4.48 (d, *J* = 13.5 Hz, 1H), 3.57 (d, *J* = 14.9 Hz, 1H), 3.10 (s, 3H), 2.96 (s, 3H), 2.83 (d, *J* = 14.9 Hz, 1H), 2.35 (s, 3H) ppm. **^{13}C NMR (151 MHz, $CDCl_3$)** δ 169.5, 167.4, 150.2, 136.7, 135.8, 133.5, 130.2, 129.4, 129.0, 129.0, 128.2, 126.8, 125.6, 85.1, 68.6, 58.2, 38.2, 28.5, 27.9, 21.2 ppm. IR (cm^-) 2968, 1768, 1467, 1378, 1159, 950, 816. **HRMS** (ESI) (m/z) $[M+H]^+$ Calcd for $C_{24}H_{24}N_2NaO_4^+$ 427.1628; found 427.1624. **HPLC**: The product was analyzed by HPLC to determine the enantiomeric excess: 92% ee (Chiralpak AD-H, *n*-hexane/*i*-propanol = 70/30, 1 mL/min, 254 nm) t_R = 7.877 min, 10.468 min.

36 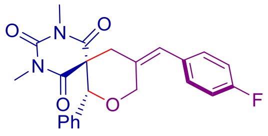

(*R*,*Z*)-10-(4-fluorobenzylidene)-2,4-dimethyl-7-phenyl-8-oxa-2,4-diazaspiro[5.5] undecane-1,3,5-trione

Yellow oil. **Yield:** 52% yield. $[\alpha]_D$ = + 73 (*c* = 0.080, CH_2Cl_2, 33.5 ℃). **^{1}H NMR (600 MHz, $CDCl_3$)** δ 7.30 – 7.25 (m, 3H, overlapped with the peak of chloroform), 7.21 – 7.17 (m, 2H), 7.09 – 7.02 (m, 4H), 6.44 (s, 1H), 5.13 (d, *J* = 13.5 Hz, 1H), 4.89 (s, 1H), 4.46 (d, *J* = 13.5 Hz, 1H), 3.58 (d, *J* = 14.9 Hz, 1H), 3.10 (s, 3H), 2.97 (s, 3H), 2.82 (d, *J* = 14.9 Hz, 1H) ppm. **^{13}C NMR (151 MHz, $CDCl_3$)** δ 169.3, 167.4, 161.8 (d, *J* = 246.6 Hz), 150.1, 135.6, 132.4 (d, *J* = 3.3 Hz), 131.1, 130.7, 130.6, 129.5, 128.2, 125.7, 125.6, 115.3, 115.2, 85.1, 68.3, 58.1, 38.1, 28.5, 27.9 ppm. IR (cm^-) 2920, 1671, 1376, 1198, 949, 749. **HRMS** (ESI) (m/z) $[M+H]^+$ Calcd for $C_{23}H_{22}FN_2O_4^+$ 409.1558; found 409.1557. **HPLC**: The product was analyzed by HPLC to determine the enantiomeric excess: 89% ee (Chiralpak AD-H, *n*-hexane/*i*-propanol = 70/30, 1 mL/min, 254 nm) t_R = 7.434 min, 9.561 min.

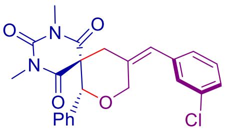

37

(*R*,*Z*)-10-(3-chlorobenzylidene)-2,4-dimethyl-7-phenyl-8-oxa-2,4-diazaspiro[5.5] undecane-1,3,5-trione

White solid. **Yield:** 64% yield. **MP:** 165.8 – 166.2 °C. $[\alpha]_D$ = + 56 (*c* = 0.150, CH_2Cl_2, 33.0 °C). **^{1}H NMR (600 MHz, $CDCl_3$)** δ 7.32 – 7.27 (m, 4H), 7.25 – 7.22 (m, 2H), 7.10 (d, *J* = 7.5 Hz, 1H), 7.06 (d, *J* = 6.9 Hz, 2H), 6.42 (s, 1H), 5.13 (d, *J* = 13.6 Hz, 1H), 4.89 (s, 1H), 4.46 (d, *J* = 13.6 Hz, 1H), 3.59 (d, *J* = 14.9 Hz, 1H), 3.11 (s, 3H), 2.97 (s, 3H), 2.83 (d, *J* = 14.9 Hz, 1H) ppm. **^{13}C NMR (151 MHz, $CDCl_3$)** δ 169.3, 167.3, 150.1, 138.1, 135.6, 134.2, 132.6, 129.5, 129.5, 129.0, 128.2, 127.2, 127.1, 125.6, 125.4, 85.1, 68.2, 58.1, 38.1, 28.5, 28.0 ppm. IR (cm^-) 2969, 1671, 1379, 1161, 951, 817, 749. **HRMS** (ESI) (m/z) $[M+H]^+$ Calcd for $C_{23}H_{22}ClN_2O_4^+$ 425.1263; found 433. 1261. **HPLC**: The product was analyzed by HPLC to determine the enantiomeric excess: 87% ee (Chiralpak AD-H, *n*-hexane/*i*-propanol = 85/15, 1 mL/min, 254 nm) t_R = 8.857 min, 11.248 min.

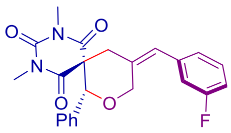

38

(*R*,*Z*)-10-(3-fluorobenzylidene)-2,4-dimethyl-7-phenyl-8-oxa-2,4-diazaspiro[5.5] undecane-1,3,5-trione

Yellow oil. **Yield:** 75% yield. $[\alpha]_D$ = + 21 (*c* = 0.100, CH_2Cl_2, 23.5 ℃). **^{1}H NMR (600 MHz, $CDCl_3$)** δ 7.35 – 7.24 (m, 4H, overlapped with the peak of chloroform), 7.06 (d, *J* = 7.1 Hz, 2H), 7.00 (d, *J* = 7.7 Hz, 1H), 6.98 – 6.92 (m, 2H), 6.44 (s, 1H), 5.15 (d, *J* = 13.6 Hz, 1H), 4.89 (s, 1H), 4.46 (d, *J* = 13.6 Hz, 1H), 3.59 (d, *J* = 14.9 Hz, 1H), 3.10 (s, 3H), 2.96 (s, 1H), 2.83 (d, *J* = 15.0 Hz, 1H) ppm. **^{13}C NMR (151 MHz, $CDCl_3$)** δ 169.3, 167.3, 162.7 (d, $J_{C\text{-}F}$ = 245.9 Hz), 150.1, 138.5 (d, $J_{C\text{-}F}$ = 7.7 Hz), 135.6, 132.4, 129.8 (d, $J_{C\text{-}F}$ = 8.4 Hz), 129.5, 128.2, 125.6, 125.6, 124.8 (d, $J_{C\text{-}F}$ = 2.7 Hz), 115.8 (d, $J_{C\text{-}F}$ = 21.3 Hz), 113.9 (d, $J_{C\text{-}F}$ = 21.1 Hz), 85.1, 68.3, 58.1, 38.1, 28.5, 28.0 ppm. IR (cm^-) 3000, 2950, 2305, 1360, 1264, 1066, 897, 735. **HRMS** (ESI) (m/z) $[M+H]^+$ Calcd for $C_{23}H_{22}FN_2O_4^+$ 409.1558; found 409.1548. **HPLC**: The product was analyzed by HPLC to determine the enantiomeric excess: 88% ee (Chiralpak AD-H, *n*-hexane/*i*-propanol = 70/30, 1 mL/min, 254 nm) t_R = 6.522 min, 8.073 min.

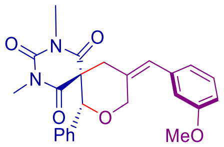

39

(*R*,*Z*)-10-(3-methoxybenzylidene)-2,4-dimethyl-7-phenyl-8-oxa-2,4-diazaspiro [5.5]undecane-1,3,5-trione

Yellow oil. **Yield:** 67% yield. $[\alpha]_D$ = + 4 (*c* = 0.100, CH_2Cl_2, 22.3 °C). **^{1}H NMR (300 MHz, $CDCl_3$)** δ 7.3 – 7.2 (m, 4H, overlapped with the peak of chloroform), 7.1 – 7.0 (m, 2H), 6.9 – 6.8 (m, 3H), 6.5 (s, 1H), 5.2 (d, *J* = 13.5 Hz, 1H), 4.9 (s, 1H), 4.5 (d, *J* = 13.4 Hz, 1H), 3.8 (s, 3H), 3.6 (d, *J* = 14.9 Hz, 1H), 3.1 (s, 3H), 3.0 (s, 3H), 2.8 (d, *J* = 15.0 Hz, 1H) ppm. **^{13}C NMR (151 MHz, $CDCl_3$)** δ 169.4, 167.4, 159.5, 150.2, 137.7, 135.7, 131.3, 129.4, 129.3, 128.2, 126.7, 125.6, 121.6, 114.6, 114.5, 112.7, 85.1, 68.6, 58.2, 55.3, 38.1, 28.5, 28.0 ppm. IR (cm^-) 3006, 2920,

1685, 1375, 1199, 949, 749. **HRMS** (ESI) (m/z) $[M+H]^+$ Calcd for $C_{24}H_{25}N_2O_5^+$ 421.1758; found 421.1751. **HPLC**: The product was analyzed by HPLC to determine the enantiomeric excess: 88% ee (Chiralpak AD-H, *n*-hexane/*i*-propanol = 85/15, 1 mL/min, 254 nm) t_R = 9.674 min, 14.342 min.

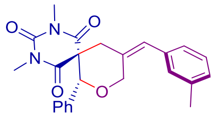

40

(*R*,*Z*)-10-(3-methylbenzylidene)-2,4-dimethyl-7-phenyl-8-oxa-2,4-diazaspiro[5.5] undecane-1,3,5-trione

Yellow oil. **Yield:** 64% yield. $[\alpha]_D$ = - 17 (*c* = 0.100, CH_2Cl_2, 23.0 °C). **^{1}H NMR (600 MHz, $CDCl_3$)** δ 7.30 – 7.27 (m, 2H), 7.27 – 7.25 (m, 1H, overlapped with the peak of chloroform), 7.24 (d, *J* = 7.6 Hz, 1H), 7.09 – 7.05 (m, 4H), 7.01 (d, *J* = 7.6 Hz, 1H), 6.46 (s, 1H), 5.19 (d, *J* = 13.5 Hz, 1H), 4.89 (s, 1H), 4.47 (d, *J* = 13.5 Hz, 1H), 3.58 (d, *J* = 14.8 Hz, 1H), 3.11 (s, 3H), 2.97 (s, 3H), 2.83 (d, *J* = 14.9 Hz, 1H), 2.36 (s, 3H) ppm. **^{13}C NMR (151 MHz, $CDCl_3$)** δ 169.4, 167.3, 150.2, 137.9, 136.3, 135.8, 130.8, 129.8, 129.4, 128.2, 128.2, 127.8, 127.0, 126.1, 125.6, 85.1, 68.6, 58.1, 38.2, 28.5, 28.0, 21.4 ppm. IR (cm^-) 2968, 1768, 1378, 1160, 951, 816, 739. **HRMS** (ESI) (m/z) $[M+H]^+$ Calcd for $C_{24}H_{24}N_2NaO_4^+$ 427.1628 found 427.1623. **HPLC**: The product was analyzed by HPLC to determine the enantiomeric excess: 88% ee (Chiralpak AD-H, *n*-hexane/*i*-propanol = 85/15, 1 mL/min, 254 nm) t_R = 7.027 min, 8.773 min.

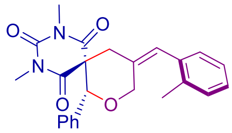

41

(*R*,*Z*)-10-(2-methylbenzylidene)-2,4-dimethyl-7-phenyl-8-oxa-2,4-diazaspiro[5.5] undecane-1,3,5-trione

White solid. **Yield:** 54% yield. **MP:** 148.7 – 149.5 °C. $[\alpha]_D$ = + 148 (*c* = 0.050, CH_2Cl_2, 26.3 °C). **^{1}H NMR (600 MHz, $CDCl_3$)** δ 7.31 – 7.23 (m, 3H, overlapped with the peak of chloroform), 7.23 – 7.15 (m, 3H), 7.10 – 7.04 (m, 3H), 6.44 (s, 1H), 4.92 (d, *J* = 13.4 Hz, 1H), 4.89 (s, 1H), 4.34 (d, *J* = 13.4 Hz, 1H), 3.60 (d, *J* = 14.6

Hz, 1H), 3.10 (s, 3H), 2.97 (s, 3H), 2.84 (d, *J* = 14.7 Hz, 1H), 2.39 (s, 3H) ppm. **^{13}C NMR (151 MHz, $CDCl_3$)** δ 169.5, 167.1, 150.2, 136.8, 135.9, 135.5, 130.6, 129.9, 129.4, 129.3, 128.2, 127.4, 126.1, 125.7, 125.5, 85.3, 68.6, 58.1, 38.2, 28.5, 27.9, 20.0 ppm. IR (cm^{-}) 2950, 1678, 1264, 1066, 897, 733. **HRMS** (ESI) (m/z) $[M+H]^+$ Calcd for $C_{24}H_{24}N_2NaO_4^+$ 427.1628; found 427.1651. **HPLC**: The product was analyzed by HPLC to determine the enantiomeric excess: 92% ee (Chiralpak AD-H, *n*-hexane/*i*-propanol = 70/30, 1 mL/min, 254 nm) t_R = 5.667 min, 6.128 min.

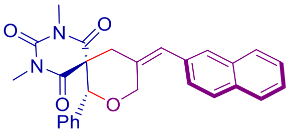

42

(*R*,*Z*)-10-(naphthalen-2-ylmethylene)-2,4-dimethyl-7-phenyl-8-oxa-2,4-diazaspiro[5.5]undecane-1,3,5-trione

Yellow oil. **Yield:** 76% yield. $[\alpha]_D$ = - 56 (*c* = 0.080, CH_2Cl_2, 27.5 ℃). **^{1}H NMR (600 MHz, $CDCl_3$)** δ 7.84 – 7.80 (m, 3H), 7.65 (s, 1H), 7.49 – 7.44 (m, 2H), 7.40 – 7.37 (m, 1H), 7.32 – 7.24 (m, 3H, overlapped with the peak of chloroform), 7.10 – 7.05 (m, 2H), 6.63 (s, 1H), 5.28 (d, *J* = 13.5 Hz, 1H), 4.92 (s, 1H), 4.56 (d, *J* = 13.6 Hz, 1H), 3.64 (d, *J* = 14.9 Hz, 1H), 3.12 (s, 3H), 2.98 (s, 3H), 2.90 (d, *J* = 14.9 Hz, 1H) ppm. **^{13}C NMR (151 MHz, $CDCl_3$)** δ 169.4, 167.4, 150.2, 135.8, 133.9, 133.3, 132.4, 131.5, 129.4, 128.2, 128.0, 127.9, 127.8, 127.7, 127.3, 126.8, 126.2, 125.9, 125.6, 85.1, 68.6, 58.2, 38.3, 28.5, 28.0 ppm. IR (cm^{-}) 2950, 1742, 1371, 1265, 1154, 945, 734. **HRMS** (ESI) (m/z) $[M+H]^+$ Calcd for $C_{27}H_{25}N_2O_4^+$ 441.1809; found 441.1831. **HPLC**: The product was analyzed by HPLC to determine the enantiomeric excess: 92% ee (Chiralpak AD-H, *n*-hexane/*i*-propanol = 85/15, 1 mL/min, 254 nm) t_R = 9.020 min, 11.719 min.

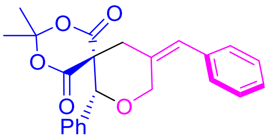

(*R*,*Z*)-10-benzylidene-3,3-dimethyl-7-phenyl-2,4,8-trioxaspiro[5.5]undecane-1,5-dione

44. White solid. **Yield:** 70% yield. **MP**: 183 - 184 ºC. $[\alpha]_D$ = - 14.0 (c = 0.5, CH_2Cl_2, 20.4 ℃). **^{1}H NMR (600 MHz, CDCl$_3$)** δ 7.29 (t, J = 7.6 Hz, 2H), 7.23 (s, 5H), 7.21 (d, J = 7.3 Hz, 1H), 7.14 (d, J = 7.6 Hz, 2H), 6.41 (s, 1H), 5.10 (d, J = 13.3 Hz, 1H), 5.05 (s, 1H), 4.35 (d, J = 13.3 Hz, 1H), 3.42 (d, J = 14.5, 1.9 Hz, 1H), 2.79 (d, J = 14.4 Hz, 1H), 1.52 (s, 3H), 0.79 (s, 3H). **^{13}C NMR (151 MHz, CDCl$_3$)** δ 168.4, 164.1, 136.6, 136.0, 129.4, 129.0, 128.6, 128.4, 128.3, 127.2, 127.1, 105.3, 83.3, 68.0, 56.5, 41.0, 30.0, 27.6. **HRMS** (ESI): (m/z) $[M+H]^+$ Calcd for $[C_{23}H_{23}O_5]^+$ required 379.1540; found 379.1542. **HPLC:** 96% ee (Chiralpak IA-H, n-hexane/i-propanol = 95/5, 1 mL/min, 254 nm) t_R = 15.900 min, 19.698 min.

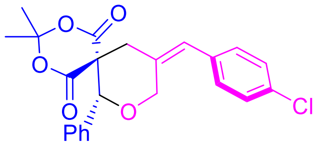

(*R*,*Z*)-10-(4-chlorobenzylidene)-3,3-dimethyl-7-phenyl-2,4,8-trioxaspiro[5.5]undecane-1,5-dione

45. White solid. **Yield:** 65% yield. **MP**: 158 - 159 ºC. $[\alpha]_D$ = - 10.4 (c = 0.5, CH_2Cl_2, 20.0 ℃). **^{1}H NMR (600 MHz, CDCl$_3$)** δ 7.35 – 7.28 (m, 7H), 7.15 (d, J = 8.0 Hz, 2H), 6.42 (s, 1H), 5.10 (d, J = 14.3 Hz, 2H), 4.39 (d, J = 13.4 Hz, 1H), 3.48 (d, J = 14.5 Hz, 1H), 2.84 (d, J = 14.5 Hz, 1H), 1.59 (s, 3H), 0.85 (s, 3H). **^{13}C NMR (151 MHz, CDCl$_3$)** δ 168.2, 136.4, 134.5, 133.1, 130.3, 129.1, 128.6, 128.6, 127.1, 127.1, 105.3, 83.3, 67.8, 40.9, 30.1, 27.5. **HRMS** (ESI): (m/z) $[M+NH_4]^+$ Calcd for $[C_{23}H_{25}ClNO_5]^+$ required 430.1416, found 430.1413. **HPLC**: 82% ee (Chiralpak IA-H, n-hexane/i-propanol = 95/5, 1 mL/min, 254 nm) t_R = 24.846 min, 28.413 min.

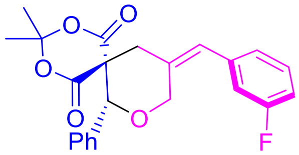

(*R*,*Z*)-10-(3-fluorobenzylidene)-3,3-dimethyl-7-phenyl-2,4,8-trioxaspiro[5.5]undecane-1,5-dione

46. White solid. **Yield**: 70% yield. **MP**: 159 - 160 ºC. $[\alpha]_D$ = - 10.8 (c = 0.5, CH_2Cl_2, 20.0 ℃). **^{1}H NMR (600 MHz, CDCl$_3$)** δ 7.31 (6H, q, J = 5.4, 4.9 Hz), 7.01 – 6.90 (3H, m), 6.44 (1H, s), 5.13 (2H, d, J = 15.9 Hz), 4.40 (1H, d, J = 13.4 Hz), 3.49 (1H,

d, J = 14.5 Hz), 2.84 (1H, d, J = 14.5 Hz), 1.59 (3H, s), 0.85 (3H, s). **^{13}C NMR (151 MHz, $CDCl_3$)** δ 168.2, 164.0, 138.2, 138.1, 136.4, 130.8, 129.8 (d, J = 8.7 Hz), 129.1, 128.6, 127.2 (d, J_{C-F} = 2.1 Hz), 127.1, 124.8 (d, J_{C-F} = 3.0 Hz), 115.9, 115.8 114.2, 114.1, 105.3, 83.3, 67.8, 56.5, 40.8, 30.1, 27.5. **HRMS** (ESI): (m/z) $[M+NH_4]^+$ Calcd for $[C_{23}H_{25}FNO_5]^+$ required 414.1711, found 414.1713. **HPLC**: 84% ee (Chiralpak IA-H, *n*-hexane/*i*-propanol = 95/5, 1 mL/min, 254 nm) t_R = 15.900 min, 19.698 min.

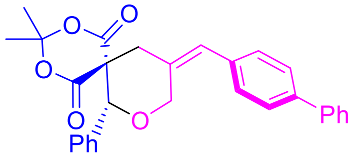

(*R*,*Z*)-10-([1,1'-biphenyl]-4-ylmethylene)-3,3-dimethyl-7-phenyl-2,4,8-trioxaspiro[5.5]undecane-1,5-dione

47. White solid. **Yield**: 58% yield. **MP:** 156 - 157 ºC. $[\alpha]_D$ = - 10.2 (c = 0.5, CH_2Cl_2, 20.1 ℃). **^{1}H NMR (600 MHz, $CDCl_3$)** δ 7.53 (dd, J_1 = 7.8, J_2 = 5.8 Hz, 4H), 7.38 (t, J = 7.6 Hz, 2H), 7.24 (s, 5H), 7.19 (s, 2H), 6.44 (s, 1H), 5.17 (d, J = 13.3 Hz, 1H), 5.07 (s, 1H), 4.39 (d, J = 13.4 Hz, 1H), 3.44 (d, J = 14.5 Hz, 1H), 2.81 (d, J = 14.5 Hz, 1H), 1.53 (s, 3H), 0.79 (s, 3H). **^{13}C NMR (151 MHz, $CDCl_3$)** δ 168.4, 164.1, 140.7, 140.1, 136.6, 135.1, 129.7, 129.5, 129.0, 128.8, 128.6, 128.0, 127.4, 127.1, 105.3, 83.3, 68.0, 56.6, 41.0, 30.1, 27.6. **HRMS** (ESI): (m/z) $[M+NH_4]^+$ Calcd for $[C_{29}H_{30}NO_5]^+$ required 472.2118, found 472.2116. **HPLC**: 84% ee (Chiralpak IA-H, *n*-hexane/*i*-propanol = 90/10, 1 mL/min, 254 nm) t_R = 20.057 min, 21.642 min.

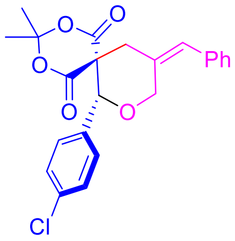

(*R*,*Z*)-10-benzylidene-7-(4-chlorophenyl)-3,3-dimethyl-2,4,8-trioxaspiro[5.5]undecane-1,5-dione

48. White solid. **Yield**: 65% yield. **MP**: 154 - 155 ºC. $[\alpha]_D$ = 10.9 (c = 0.5, CH_2Cl_2, 20.0 ℃). **^{1}H NMR (600 MHz, $CDCl_3$)** δ 7.36 (t, J = 7.5 Hz, 2H), 7.31 – 7.24 (m,

5H), 7.21 (d, J = 7.6 Hz, 2H), 6.49 (s, 1H), 5.16 (d, J = 13.3 Hz, 1H), 5.11 (s, 1H), 4.40 (d, J = 13.3 Hz, 1H), 3.47 (d, J = 14.4 Hz, 1H), 2.86 (d, J = 14.5 Hz, 1H), 1.62 (s, 3H), 1.01 (s, 3H). **^{13}C NMR (151 MHz, $CDCl_3$)** δ 168.2, 164.0, 137.8, 136.4, 134.3, 131.0, 129.6, 129.1, 129.0, 128.6, 127.4, 127.2, 127.1, 126.9, 105.3, 83.3, 67.7, 56.5, 40.8, 30.1, 27.5. **HRMS** (ESI): (m/z) $[M+Na]^+$ Calcd for $[C_{23}H_{21}ClNaO_5]^+$ required 435.0970, found 435.0971. **HPLC**: 90% ee (Chiralpak IG-H, *n*-hexane/*i*-propanol = 85/15, 1 mL/min, 254 nm) t_R = 18.810 min, 20.021 min.

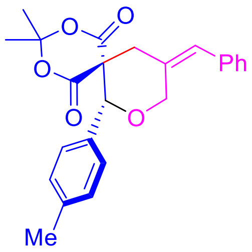

(*R*,*Z*)-10-benzylidene-3,3-dimethyl-7-(*p*-tolyl)-2,4,8-trioxaspiro[5.5]undecane-1,5-dione

49. White solid. **Yield**: 61% yield. **MP**: 161 - 162 ℃. $[\alpha]_D$ = - 9.8 (c = 0.5, CH_2Cl_2, 20.0 ℃). **^{1}H NMR (600 MHz, $CDCl_3$)** δ 7.29 (t, J = 7.5 Hz, 2H), 7.19 (d, J = 2.3 Hz, 2H), 7.16 – 7.04 (m, 5H), 6.42 (s, 1H), 5.85 (s, 1H), 5.38 (d, J = 13.5 Hz, 1H), 4.59 (d, J = 13.4 Hz, 1H), 3.46 (d, J = 14.6 Hz, 1H), 2.93 (d, J = 14.7 Hz, 1H), 2.27 (s, 3H), 1.50 (s, 3H), 0.77 (s, 3H). **^{13}C NMR (151 MHz, $CDCl_3$)** δ 166.8, 163.6, 135.2, 134.5, 134.1, 129.8, 128.8, 128.0, 127.9, 127.3, 127.0, 126.9, 126.1, 125.5, 104.4, 79.6, 68.5, 56.5, 40.1, 31.3, 25.2, 18.4. **HRMS** (ESI): (m/z) $[M+Na]^+$ Calcd for $[C_{24}H_{24}NaO_5]^+$ required 415.1516, found 415.1518. **HPLC**: 80% ee (Chiralpak IG-H, *n*-hexane/*i*-propanol = 85/15, 1 mL/min, 254 nm) t_R = 19,902 min, 25.883 min.

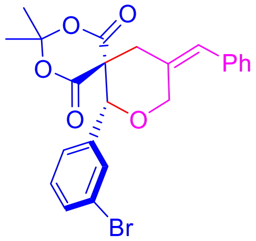

(*R*,*Z*)-10-benzylidene-7-(3-bromophenyl)-3,3-dimethyl-2,4,8-trioxaspiro[5.5]undecane-1,5-dione

50. White solid. **Yield**: 61% yield. **MP**: 176 - 178 ℃. **^{1}H NMR (600 MHz, $CDCl_3$)** δ 7.48 (d, J = 2.1 Hz, 1H), 7.44 (d, J = 7.9 Hz, 1H), 7.36 (td, J_1 = 7.6, J_2 = 2.1 Hz, 2H), 7.33 – 7.18 (m, 5H), 6.49 (s, 1H), 5.16 (d, J = 13.3 Hz, 1H), 5.10 (s, 1H), 4.40 (d, J = 13.4 Hz, 1H), 3.46 (d, J = 14.5 Hz, 1H), 2.87 (d, J = 14.5 Hz, 1H), 1.62 (s, 3H), 1.00 (s, 3H). **^{13}C NMR (151 MHz, $CDCl_3$)** δ 168.2, 163.8, 138.8, 135.9, 132.1, 130.1, 129.0, 128.8, 128.4, 125.7, 122.7, 105.3, 82.3, 68.0, 56.4, 40.9, 30.0, 27.7. **HRMS** (ESI): (m/z) $[M+H]^+$ Calcd for $[C_{23}H_{22}BrO_5]^+$ required 457.0645, found 457.0648 **HPLC**: 90% ee (Chiralpak IA-H, *n*-hexane/*i*-propanol = 90/10, 1 mL/min, 254 nm) t_R = 10.579 min, 17.327 min.

52 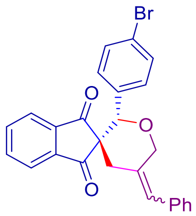

(*S*,*E*)-5'-benzylidene-2'-(4-bromophenyl)-5',6'-dihydro-2'*H*,4'*H*-spiro[indene-2,3'-pyran]-1,3-dione

White solid. **Yield**: 78 %. **MP:** 146.1 – 146.6 °C. $[\alpha]_D$ = + 148 (c = 0.100, CH_2Cl_2, 28.4 °C). **^{1}H NMR (400 MHz, $CDCl_3$)** δ 7.71 - 7.68 (m, 2H), 7.40 - 7.36 (m, 2H), 7.17 - 7.15 (m, 2H), 7.03 - 6.99 (m, 2H), 6.46 (s, 1H), 5.19 (d, J = 13.1 Hz, 1H), 4.96 (s, 1H), 4.38 (d, J = 13.2 Hz, 1H), 3.18 (d, J = 14.3 Hz, 1H), 2.56 (d, J = 14.4 Hz, 1H) ppm. **^{13}C NMR (101 MHz, $CDCl_3$)** δ 201.0 (s), 199.1 (s), 142.1 (s), 136.3 (d, J = 8.0 Hz), 136.0 (s), 135.4 (s), 131.1 (s), 130.7 (s), 129.1 (s), 128.4 (s), 128.3 (s), 128.2 (s), 127.1 (s), 123.0 (s), 81.1 (s), 67.9 (s), 59.8 (s), 37.7 (s), 29.7 (s) ppm. IR (cm^-) 2969, 1373, 1378, 1160, 951, 817. **HRMS** (ESI) (m/z) $[M+Na]^+$ Calcd for $C_{26}H_{19}BrNaO_3^+$ 481.0410; found 481.0413. **HPLC**: The product was analyzed by HPLC to determine the enantiomeric excess: 96% ee (Chiralpak AD-H, *n*-hexane/*i*-propanol = 85/15, 1 mL/min, 254 nm) t_R = 14.481 min, 16.157 min, 20.060 min, 28.138 min.

53 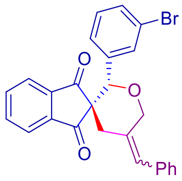

(*S*,*E*)-5'-benzylidene-2'-(3-bromophenyl)-5',6'-dihydro-2'*H*,4'*H*-spiro[indene-2,3'-pyran]-1,3-dione

White solid. **Yield**: 76 %. **MP:** 145.2 – 145.9 °C. $[\alpha]_D$ = + 4 (*c* = 0.050, CH_2Cl_2, 27.3 °C). **^{1}H NMR (400 MHz, $CDCl_3$)** δ 7.83 - 7.76 (m, 2H), 7.72 - 7.68 (m, 2H), 7.40 (t, *J* = 7.4 Hz, 2H), 7.34 - 7.30 (m, 3H), 7.27 (s, 1H), 7.14 - 7.12 (m, 1H), 7.07 - 7.05 (m, 1H), 6.90 (t, *J* = 7.9 Hz, 1H), 6.50 (s, 1H), 5.23 (d, *J* = 13.2 Hz, 1H), 4.97 (s, 1H), 4.41 (d, *J* = 13.2 Hz, 1H), 3.22 (d, *J* = 14.4 Hz, 1H), 2.60 (d, *J* = 14.3 Hz, 1H) ppm. **^{13}C NMR (101 MHz, $CDCl_3$)** δ 200.9 (s), 199.0 (s), 142.2 (s), 140.7 (s), 139.4 (s), 136.3 (s), 135.9 (s), 135.4 (s), 131.2 (s), 130.6 (s), 129.6 (d, *J* = 11.1 Hz), 129.1 (s), 128.3 (s), 128.2 (s), 127.1 (s), 125.2 (s), 122.9 (d, *J* = 5.2 Hz), 122.2 (s), 81.1 (s), 67.9 (s), 59.9 (s), 37.4 (s) ppm. IR (cm^-) 2969, 1671, 1379, 1160, 951, 817. **HRMS** (ESI) (m/z) $[M+Na]^+$ Calcd for $C_{26}H_{19}O_3BrNa^+$ 481.0410; found 481.0414. **HPLC**: The product was analyzed by HPLC to determine the enantiomeric excess: 70% ee (Chiralpak IG-H, *n*-hexane/*i*-propanol = 90/10, 1 mL/min, 254 nm). t_R = 15.150 min, 18.170 min, 27.603 min, 51.433 min.

54 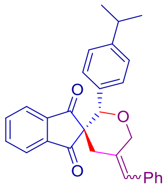

(*S*,*E*)-5'-benzylidene-2'-(4-isopropylphenyl)-5',6'-dihydro-2'*H*,4'*H*-spiro[indene-2,3'-pyran]-1,3-dione

White oil. **Yield**: 56 %. $[\alpha]_D$ = + 46 (*c* = 0.100, CH_2Cl_2, 27.5 °C). **^{1}H NMR (400 MHz, $CDCl_3$)** δ 7.64 - 7.61 (m, 2H), 7.42 - 7.37 (m, 2H), 7.04 - 7.03 (m, 2H), 6.87 (s, 2H), 6.50 (s, 1H), 5.23 (d, *J* = 13.2 Hz, 1H), 4.97 (s, 1H), 4.43 (d, *J* = 13.2 Hz, 1H), 3.23 (d, *J* = 14.4 Hz, 1H), 2.66 - 2.63 (m, 1H), 2.60 (d, *J* = 13.9 Hz, 1H), 1.02 (s, 3H), 1.00 (s, 3H) ppm. **^{13}C NMR (101 MHz, $CDCl_3$)** δ 201.2 (s), 199.8 (s), 148.8

(s), 142.4 (s), 140.9 (s), 135.4 (s), 134.9 (s), 134.4 (s), 131.9 (s), 129.1 (s), 128.3 (s), 127.7 (s), 127.0 (s), 126.9 (s), 126.0 (s), 122.8 (s), 122.7 (s), 82.2 (s), 68.0 (s), 60.3 (s), 37.2 (s), 33.6 (s), 23.9 (s) ppm. IR (cm^{-}) 2950, 1692, 1361, 1264, 1066, 897, 734. **HRMS** (ESI) (m/z) $[M+Na]^+$ Calcd for $C_{29}H_{26}O_3Na^+$ 445.1774; found 445.1784. **HPLC**: The product was analyzed by HPLC to determine the enantiomeric excess: 75% ee (Chiralpak AD-H, *n*-hexane/*i*-propanol = 85/15, 1 mL/min, 254 nm). t_R = 10.363 min, 12.294 min, 13.453 min, 15.140 min.

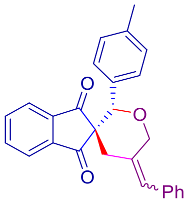

55

(*S*,*E*)-5'-benzylidene-2'-(4-tolyl)-5',6'-dihydro-2'*H*,4'*H*-spiro[indene-2,3'-pyran]-1,3-dione

White solid. **Yield**: 86 %. **MP:** 80.6 – 81.2 °C. $[\alpha]_D$ = + 125 (*c* = 100, CH_2Cl_2, 28.5 °C). **^{1}H NMR (400 MHz, $CDCl_3$)** δ 7.66 - 7.63 (m, 2H), 7.40 - 7.35 (m, 2H), 7.01 - 6.98 (m, 2H), 6.82 - 6.80 (m, 2H), 6.46 (s, 1H), 5.20 (d, *J* = 13.1 Hz, 1H), 4.95 (s, 1H), 4.39 (d, *J* = 13.2 Hz, 1H), 3.19 (d, *J* = 14.3 Hz, 1H), 2.56 (d, *J* = 14.3 Hz, 1H), 2.08 (s, 3H) ppm. **^{13}C NMR (101 MHz, $CDCl_3$)** δ 201.3 (s), 199.6 (s), 142.3 (s), 140.8 (s), 137.7 (s), 136.4 (s), 135.6 (s), 135.1 (s), 131.2 (s), 129.1 (s), 128.6 (s), 128.3 (s), 127.8 (s), 127.0 (s), 126.5 (s), 122.9 (s), 122.8 (s), 82.0 (s), 68.0 (s), 60.1 (s), 37.6 (s), 20.9 (s) ppm. IR (cm^{-}) 2969, 1692, 1379, 1160, 950, 816. **HRMS** (ESI) (m/z) $[M+Na]^+$ Calcd for $C_{26}H_{19}O_3BrNa^+$ 417.1461; found 417.1569. **HPLC**: The product was analyzed by HPLC to determine the enantiomeric excess: 87% ee (Chiralpak AD-H, *n*-hexane/*i*-propanol = 85/15, 1 mL/min, 254 nm) t_R = 13.296 min, 14.341 min, 17.401 min, 23.103 min.

References

1. Frisch, M. J.; Trucks, G. W.; Schlegel, H. B.; Scuseria, G. E.; Robb, M. A.; Cheeseman, J. R.; Scalmani, G.; Barone, V.; Petersson, G. A.; Nakatsuji, H.; Li, X.; Caricato, M.; Marenich, A. V.; Bloino, J.; Janesko, B. G.; Gomperts, R.; Mennucci, B.; Hratchian, H. P.; Ortiz, J. V.; Izmaylov, A. F.; Sonnenberg, J. L.; Williams-Young, D.; Ding, F.; Lipparini, F.; Egidi, F.; Goings, J.; Peng, B.; Petrone, A.; Henderson, T.; Ranasinghe, D.; Zakrzewski, V. G.; Gao, J.; Rega, N.; Zheng, G.; Liang, W.; Hada, M.; Ehara, M.; Toyota, K.; Fukuda, R.; Hasegawa, J.; Ishida, M.; Nakajima, T.; Honda, Y.; Kitao, O.; Nakai, H.; Vreven, T.; Throssell, K.; Montgomery Jr., J. A.; Peralta, J. E.; Ogliaro, F.; Bearpark, M. J.; Heyd, J. J.; Brothers, E. N.; Kudin, K. N.; Staroverov, V. N.; Keith, T. A.; Kobayashi, R.; Normand, J.; Raghavachari, K.; Rendell, A. P.; Burant, J. C.; Iyengar, S. S.; Tomasi, J.; Cossi, M.; Millam, J. M.; Klene, M.; Adamo, C.; Cammi, R.; Ochterski, J. W.; Martin, R. L.; Morokuma, K.; Farkas, O.; Foresman, J. B.; Fox, D. J. Gaussian16, Gaussian, Inc., Wallingford, CT, 2016.

2. Zhao,Y.; Truhlar, D. G. *Theor. Chem. Acc.* **2008**, *120*, 215.

3. Zhao, Y. T.; Truhlar, D. G. *J. Chem. Theory Comput.* **2008**, *4*, 1849.

4. Zhao, Y. T.; Truhlar, D. G. *Acc. Chem. Res.* **2008**, *41*, 157.

5 Lu, T.; Chen, F. *J. Comput. Chem.*, **2011**, *33*, 580.

6. E Fillion, A Kavoosi, K Nguyen, C Ieritano, *Chem. Comm.* 2016, *52 (87)*, 12813-12816.

7. C. Milite, , A. Feoli , K. Sasaki , *J. Med. Chem.* **2015**, *58 (6)*, 2779-2798.

5. Copies of ^{31}P NMR, ^{1}H NMR, ^{13}C NMR spectra

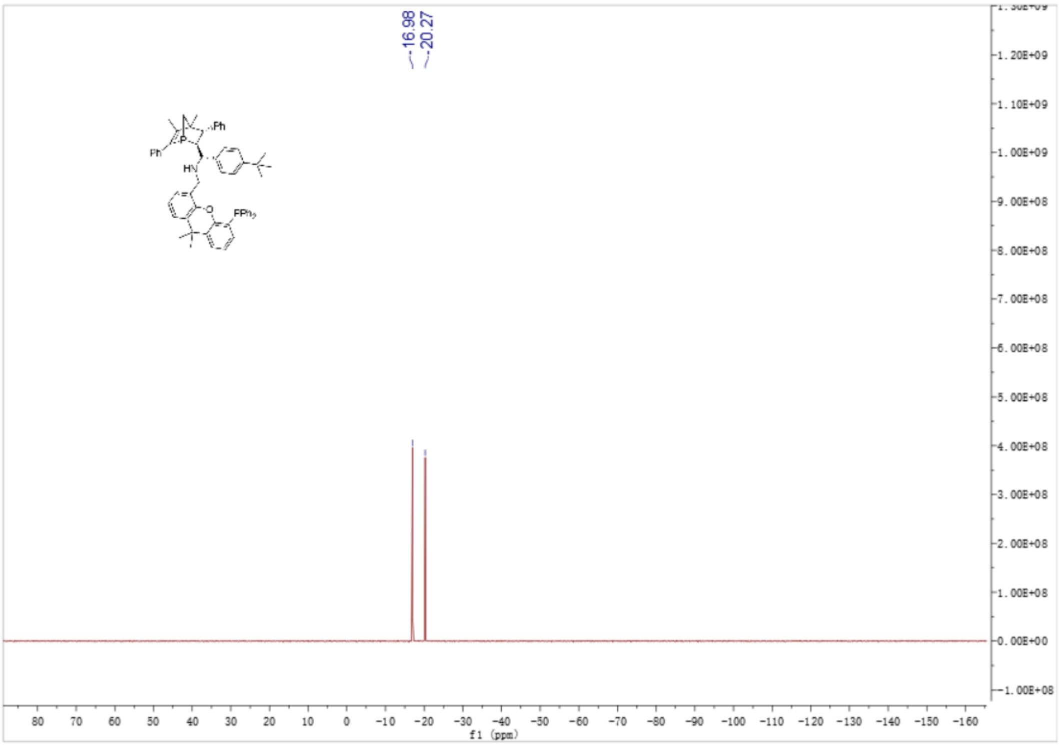

^{31}P ($CDCl_3$, 162 MHz) NMR of compound **ZU-1**

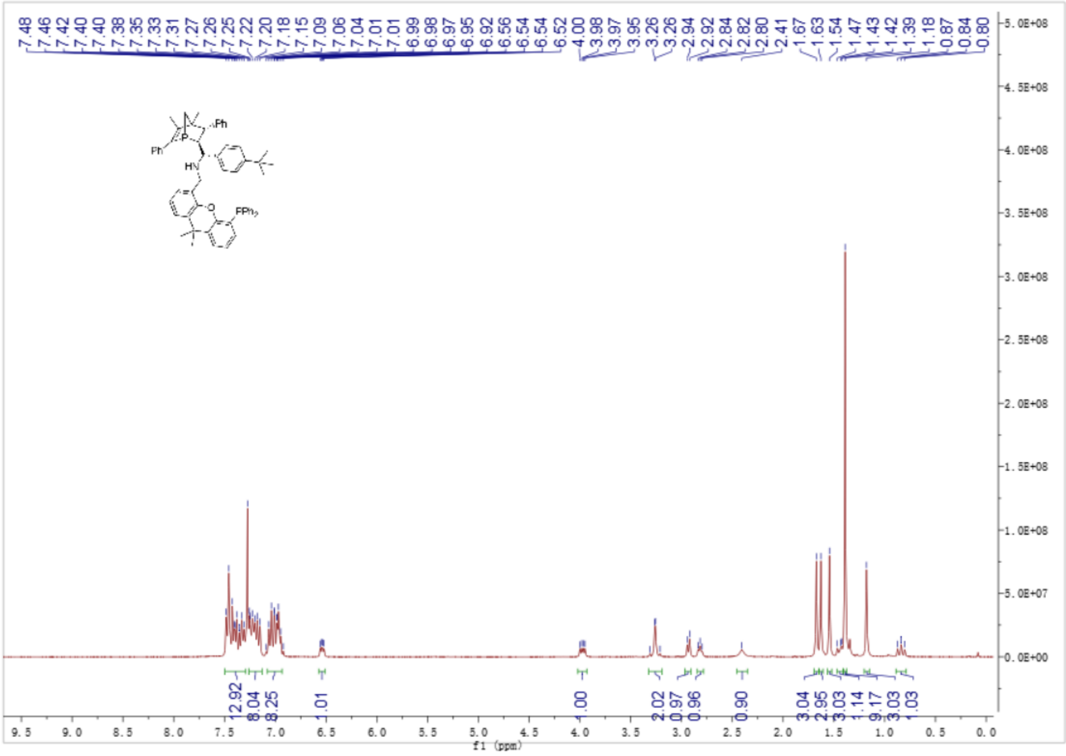

^{1}H ($CDCl_3$, 300 MHz) NMR of compound **ZU-1**

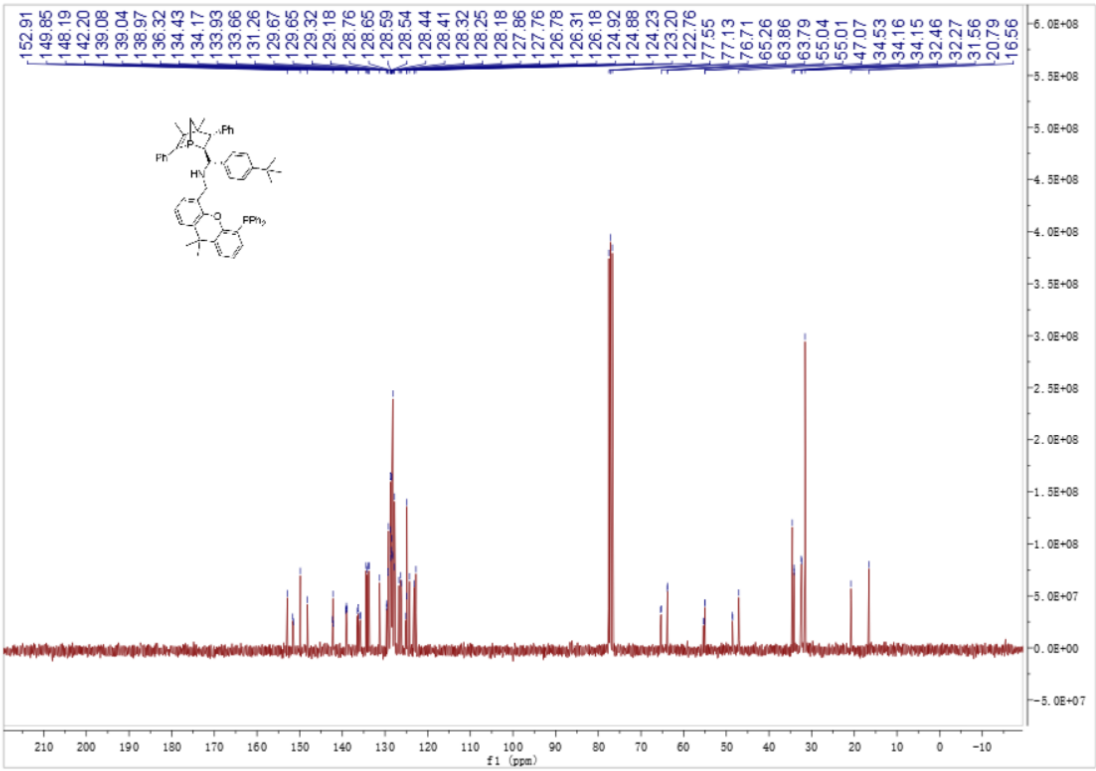

^{13}C ($CDCl_3$, 75 MHz) NMR of compound **ZU-1**

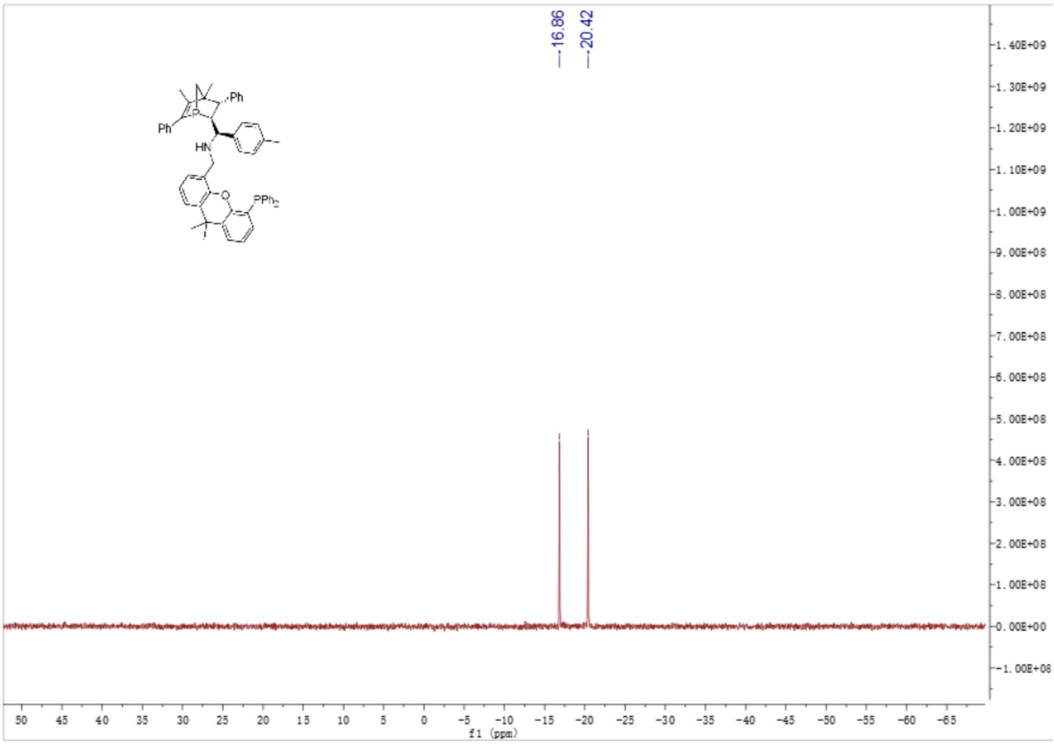

^{31}P ($CDCl_3$, 121 MHz) NMR of compound **ZU-2**

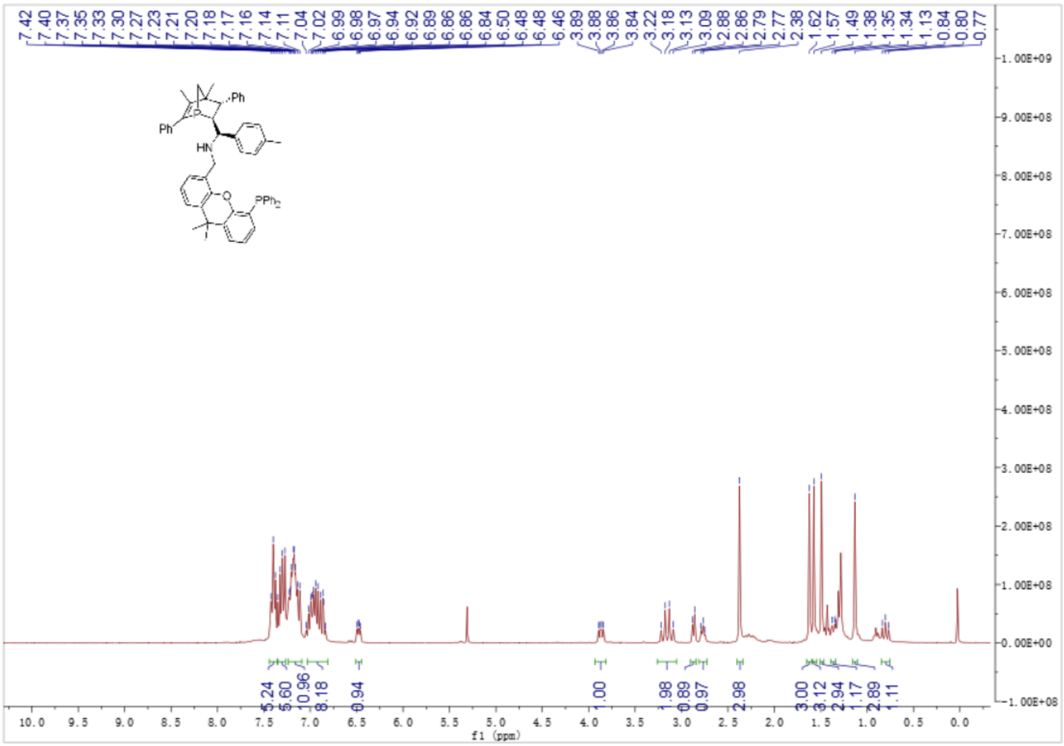

^{1}H ($CDCl_3$, 400 MHz) NMR of compound **ZU-2**

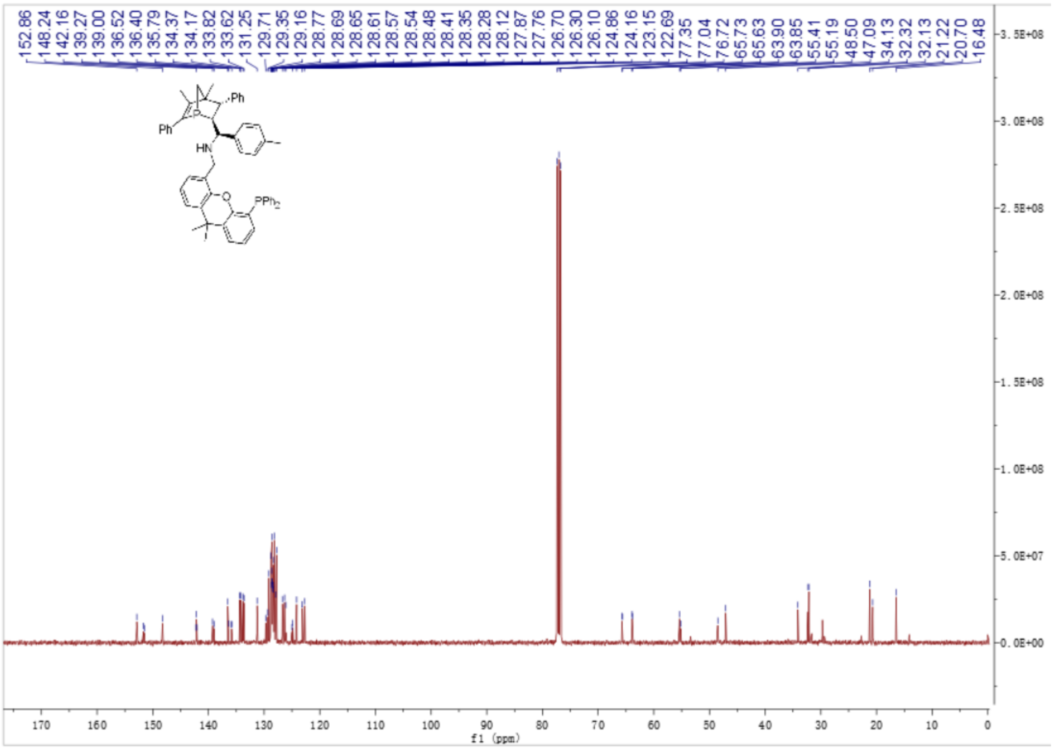

^{13}C ($CDCl_3$, 75 MHz) NMR of compound **ZU-2**

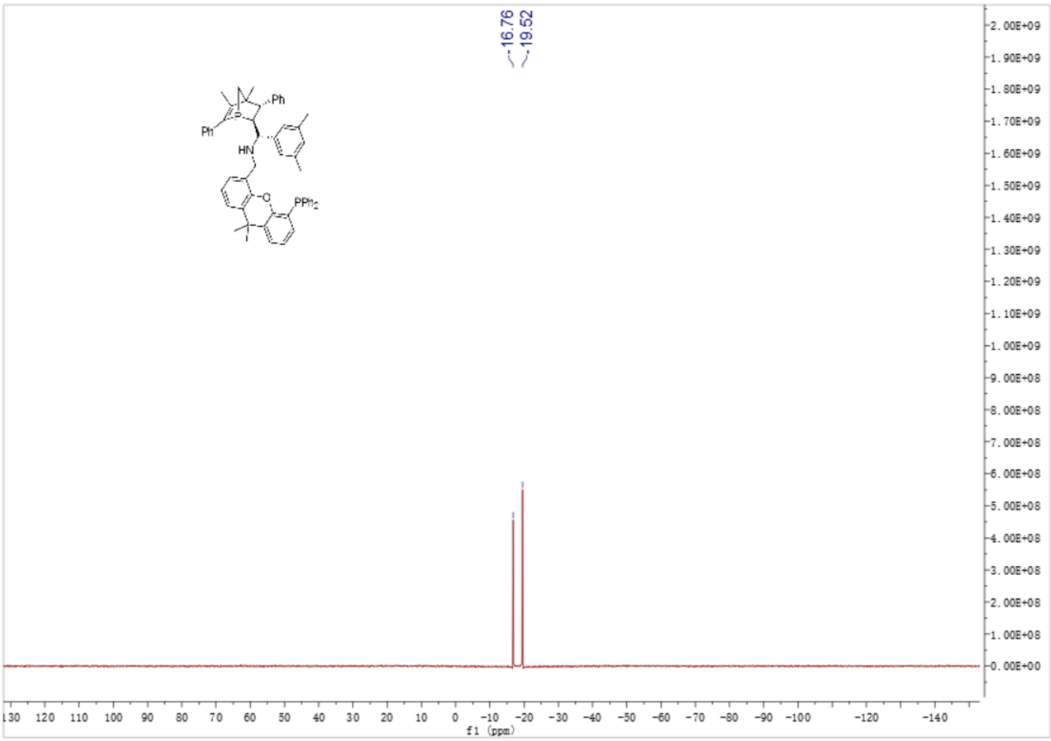

^{31}P ($CDCl_3$, 121 MHz) NMR of compound **ZU-3**

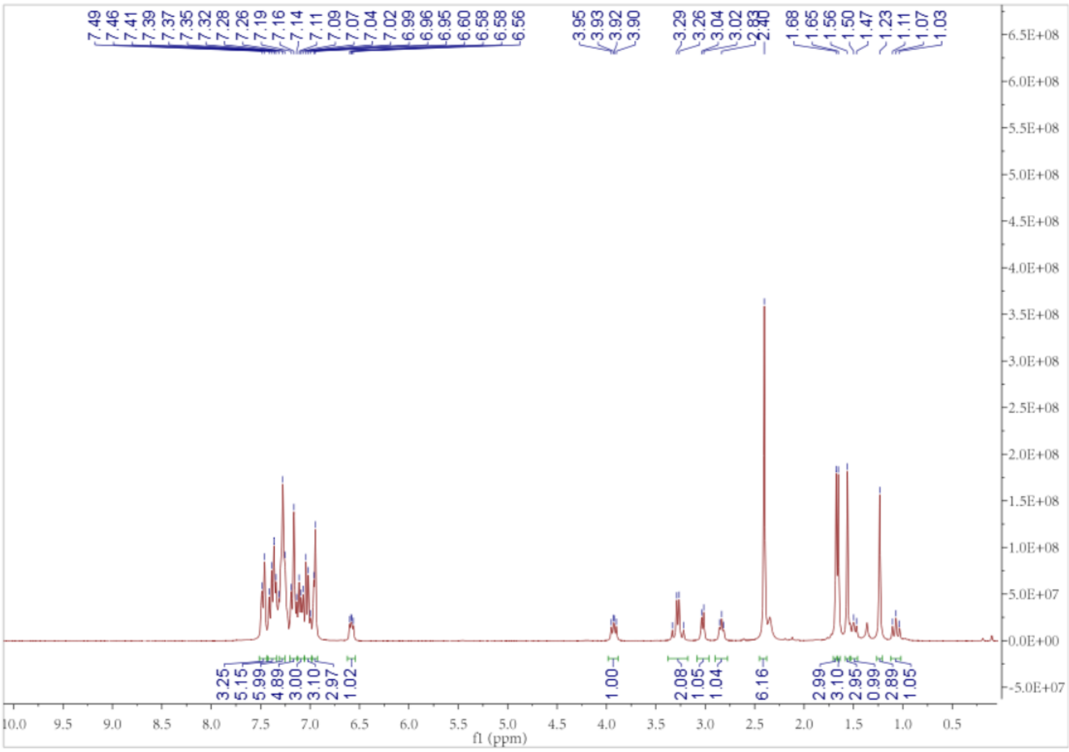

^{1}H ($CDCl_3$, 400 MHz) NMR of compound **ZU-3**

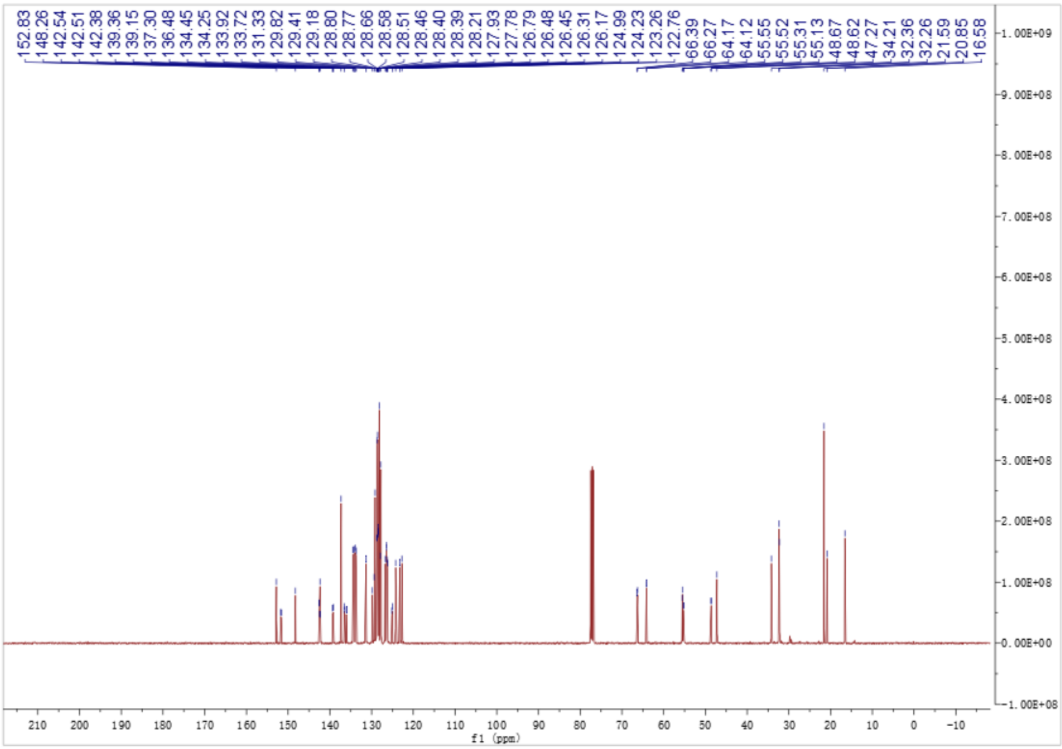

^{13}C ($CDCl_3$, 75 MHz) NMR of compound **ZU-3**

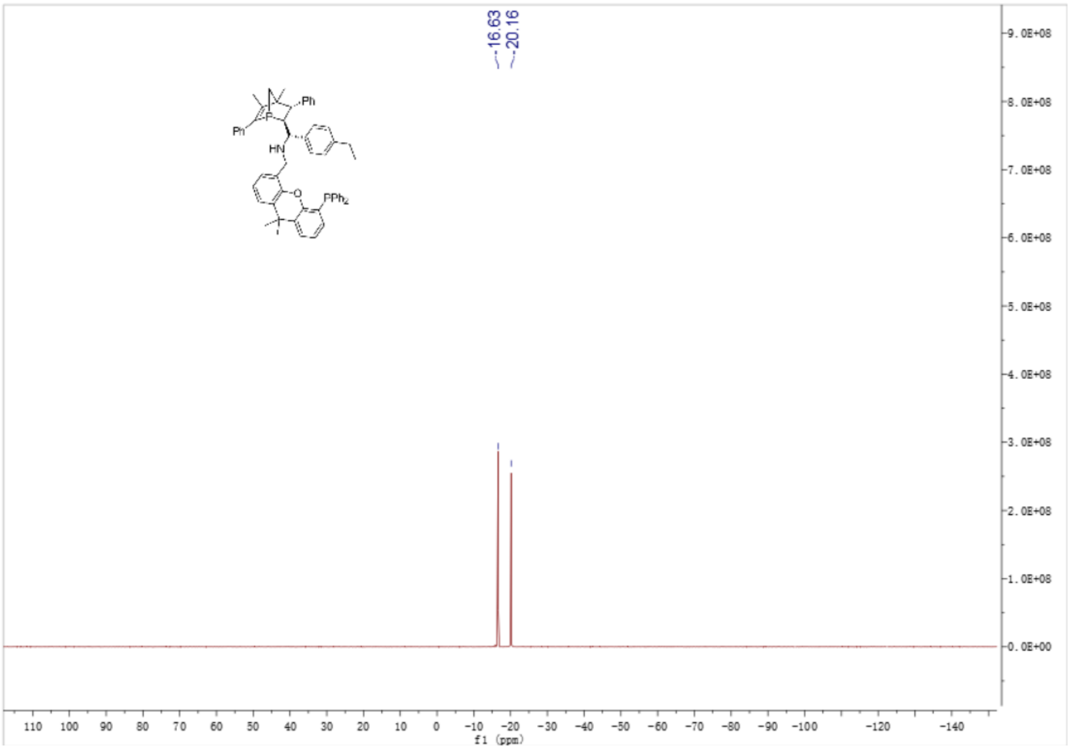

^{31}P ($CDCl_3$, 121 MHz) NMR of compound **ZU-4**

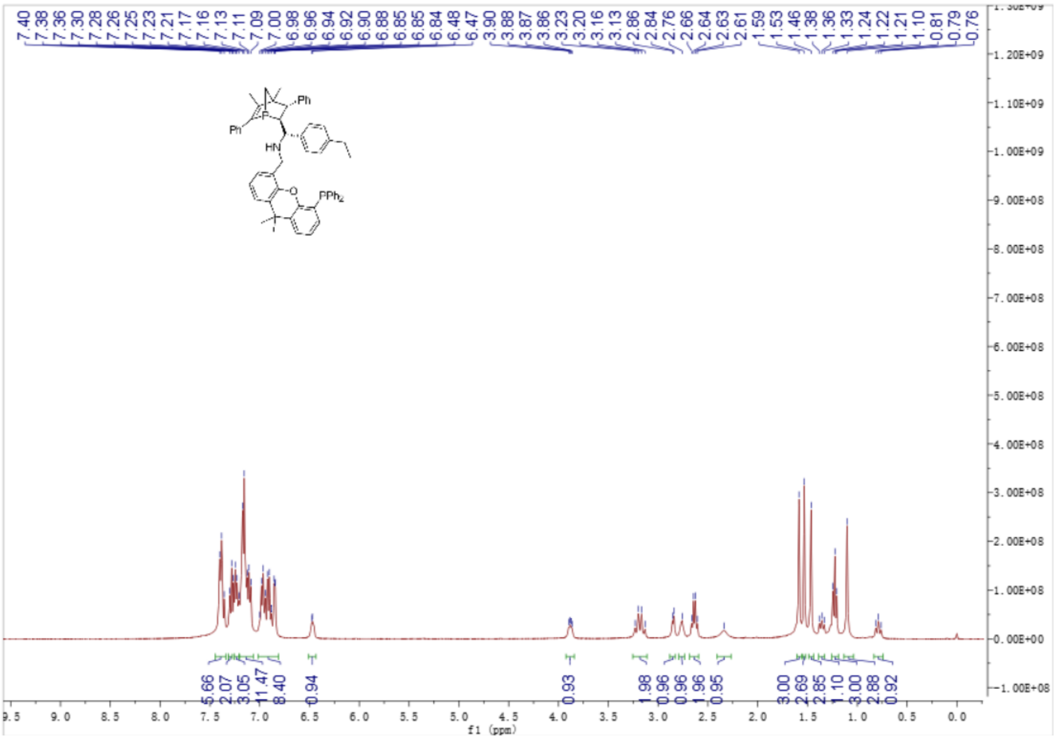

^{1}H ($CDCl_3$, 400 MHz) NMR of compound **ZU-4**

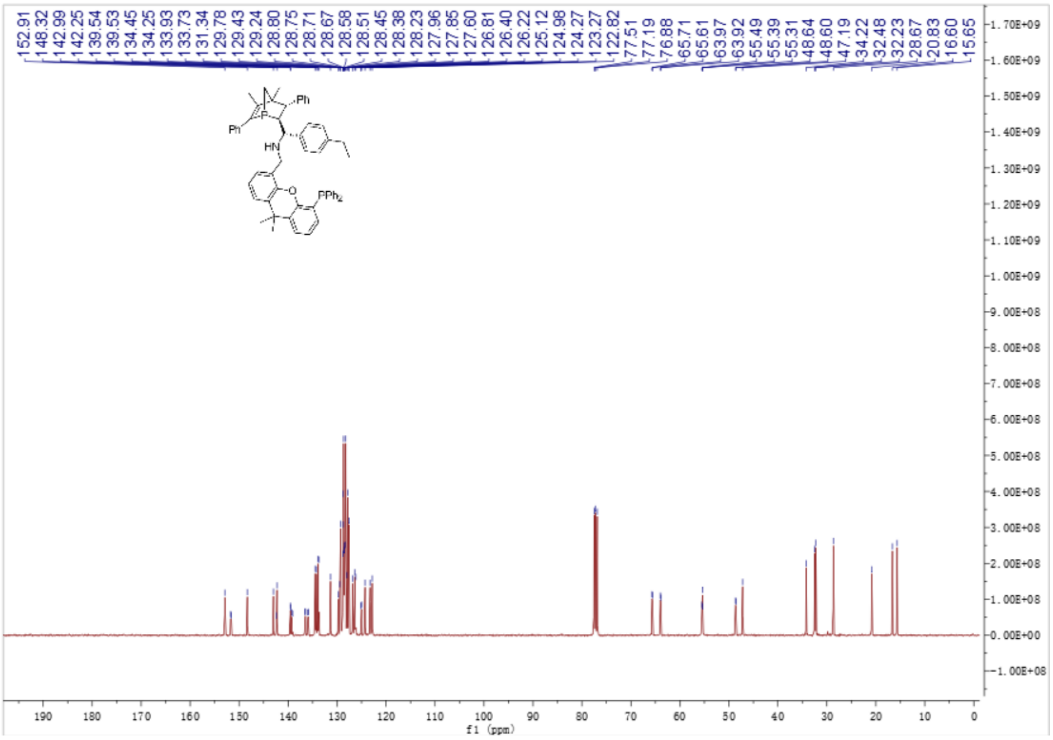

^{13}C ($CDCl_3$, 75 MHz) NMR of compound **ZU-4**

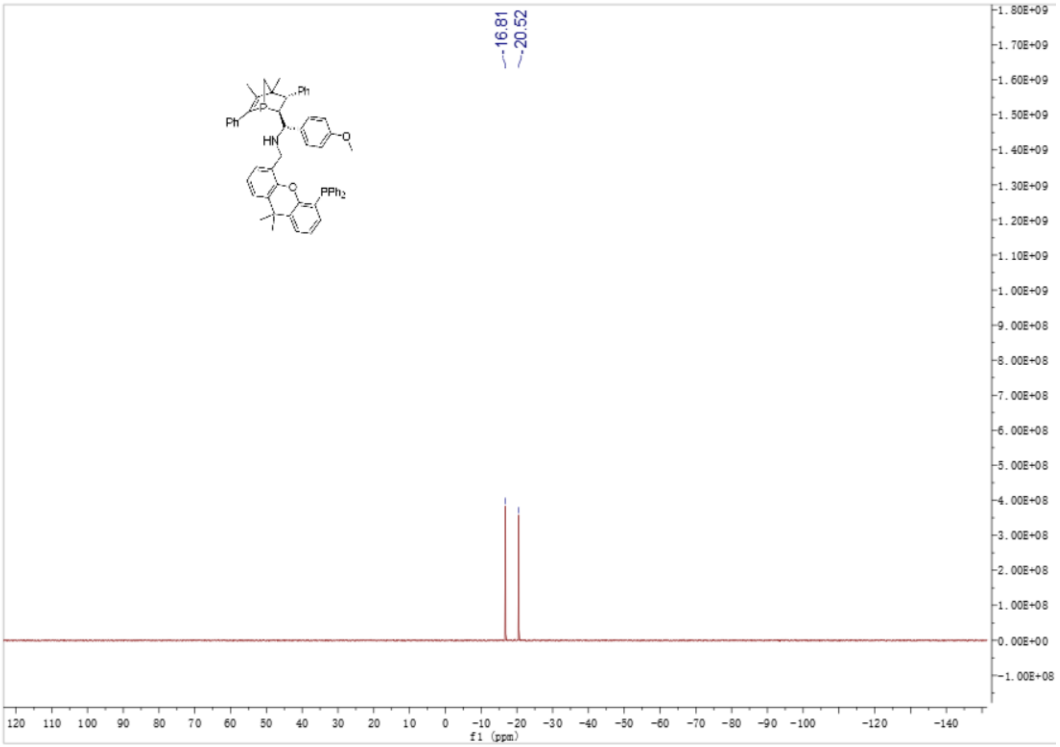

^{31}P ($CDCl_3$, 121 MHz) NMR of compound **ZU-5**

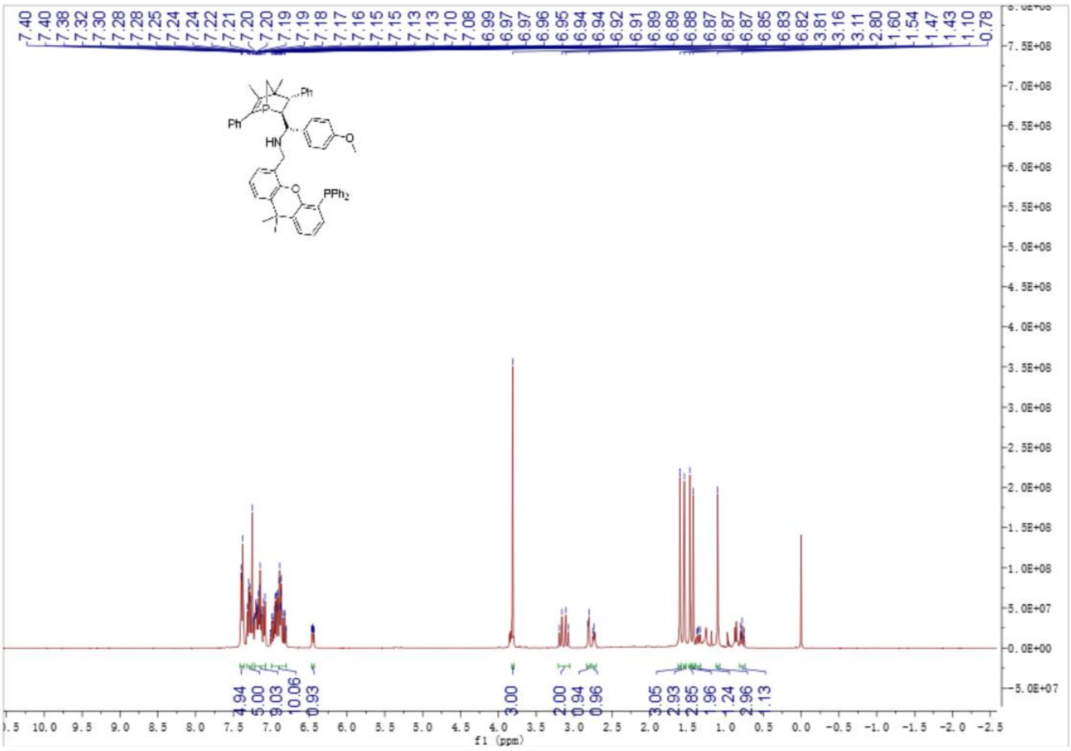

^{1}H ($CDCl_3$, 400 MHz) NMR of compound **ZU-5**

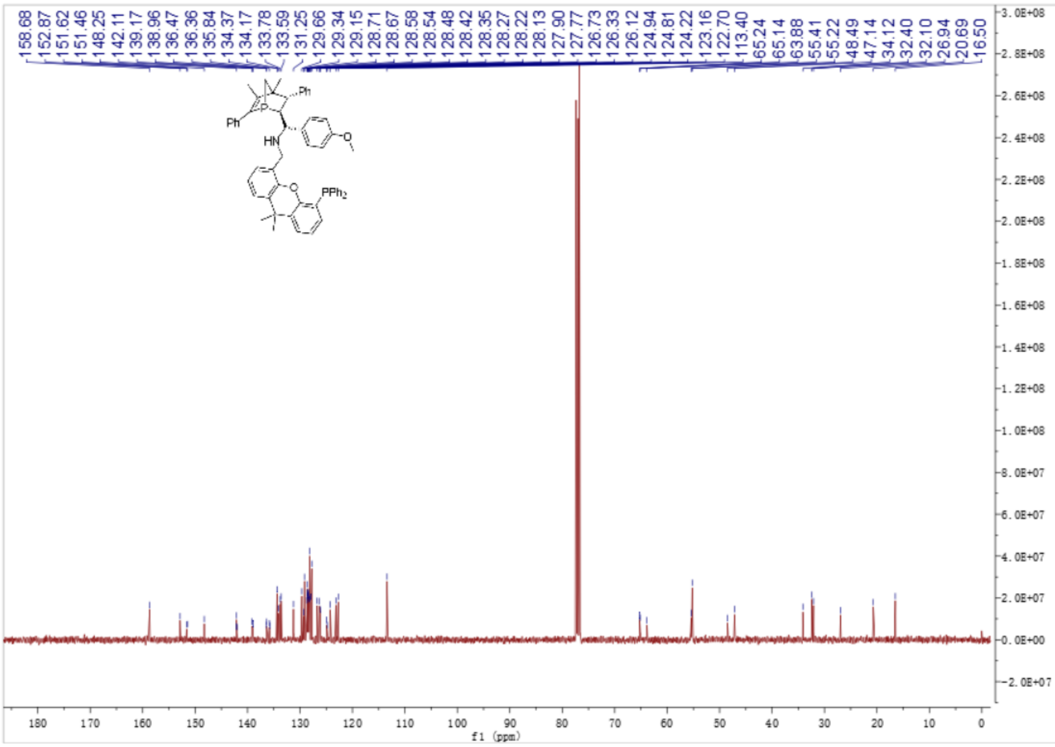

^{13}C ($CDCl_3$, 75 MHz) NMR of compound **ZU-5**

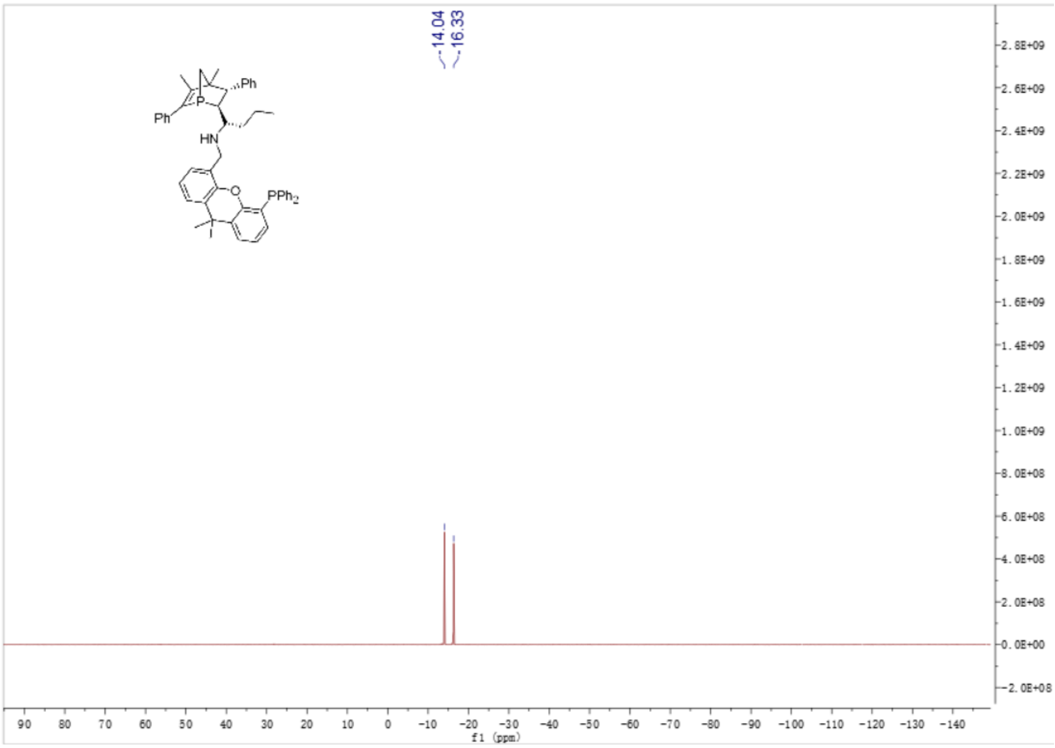

^{31}P ($CDCl_3$, 121 MHz) NMR of compound **ZU-6**

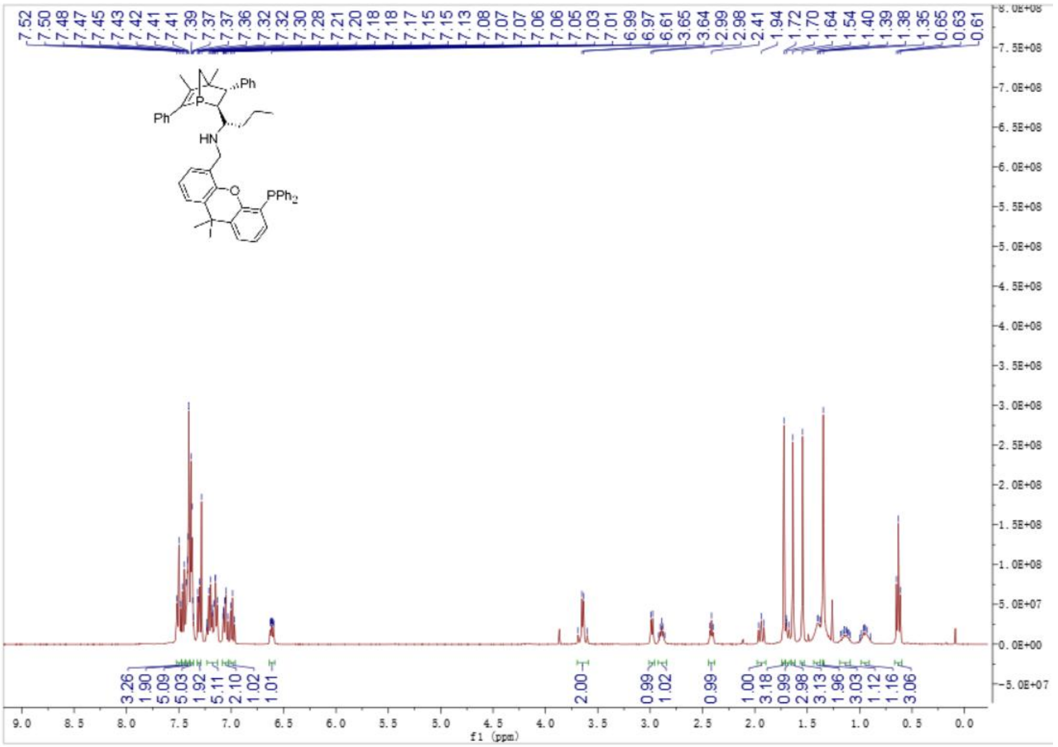

^{1}H ($CDCl_3$, 400 MHz) NMR of compound **ZU-6**

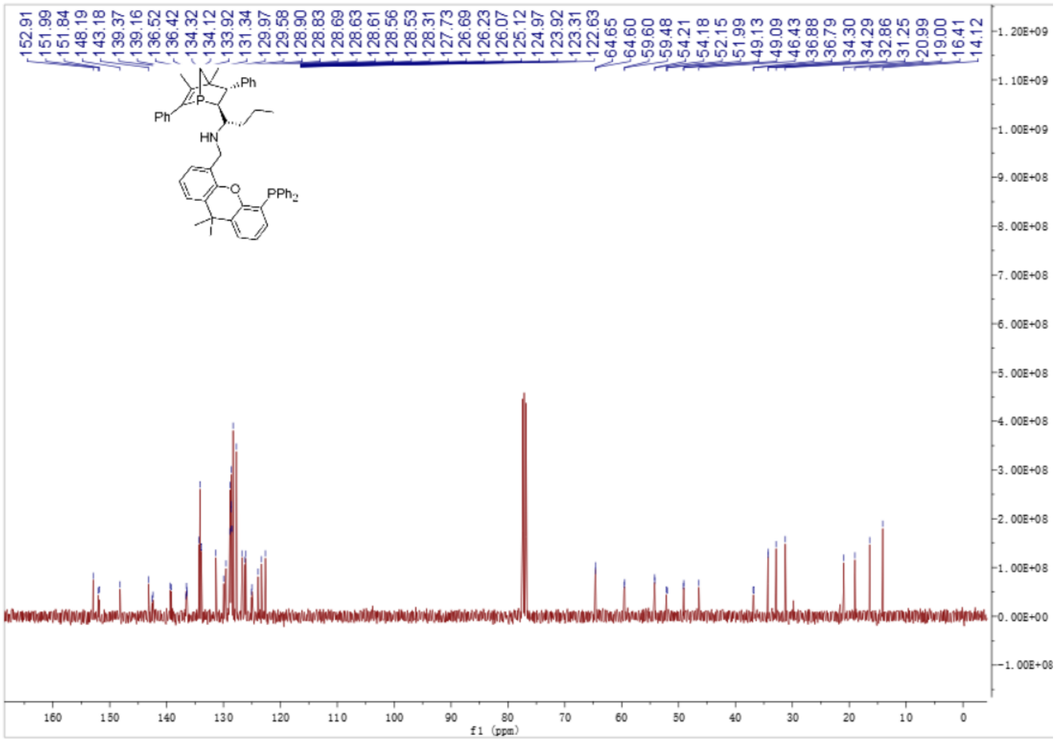

^{13}C ($CDCl_3$, 75 MHz) NMR of compound **ZU-6**

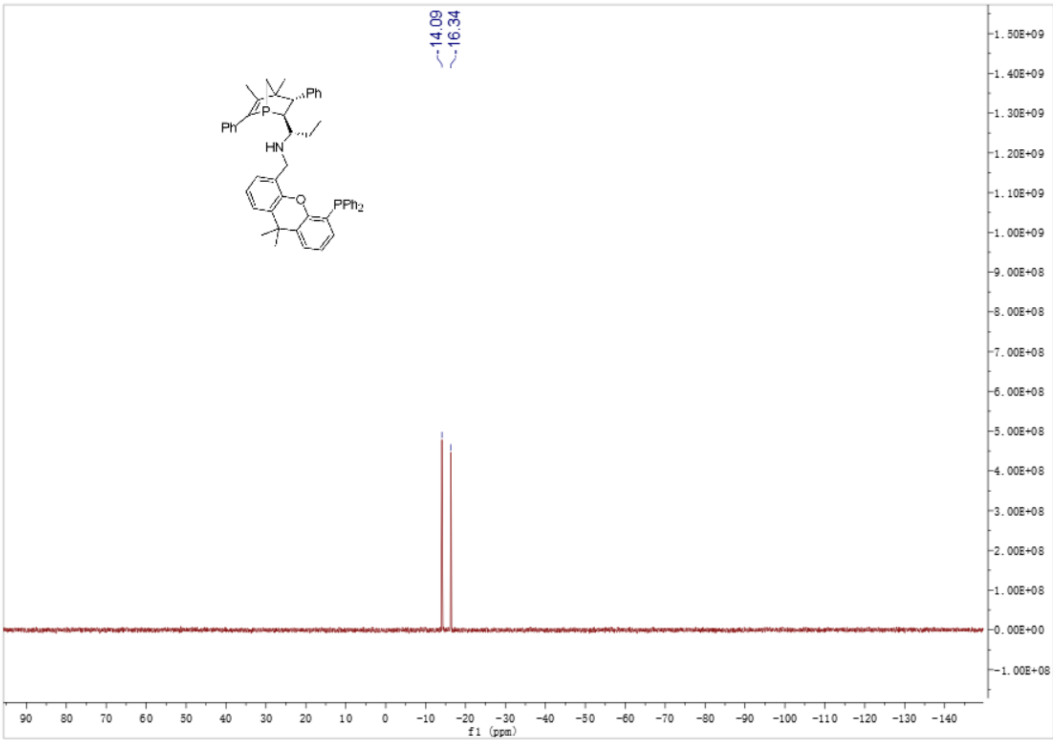

^{31}P ($CDCl_3$, 121 MHz) NMR of compound **ZU-7**

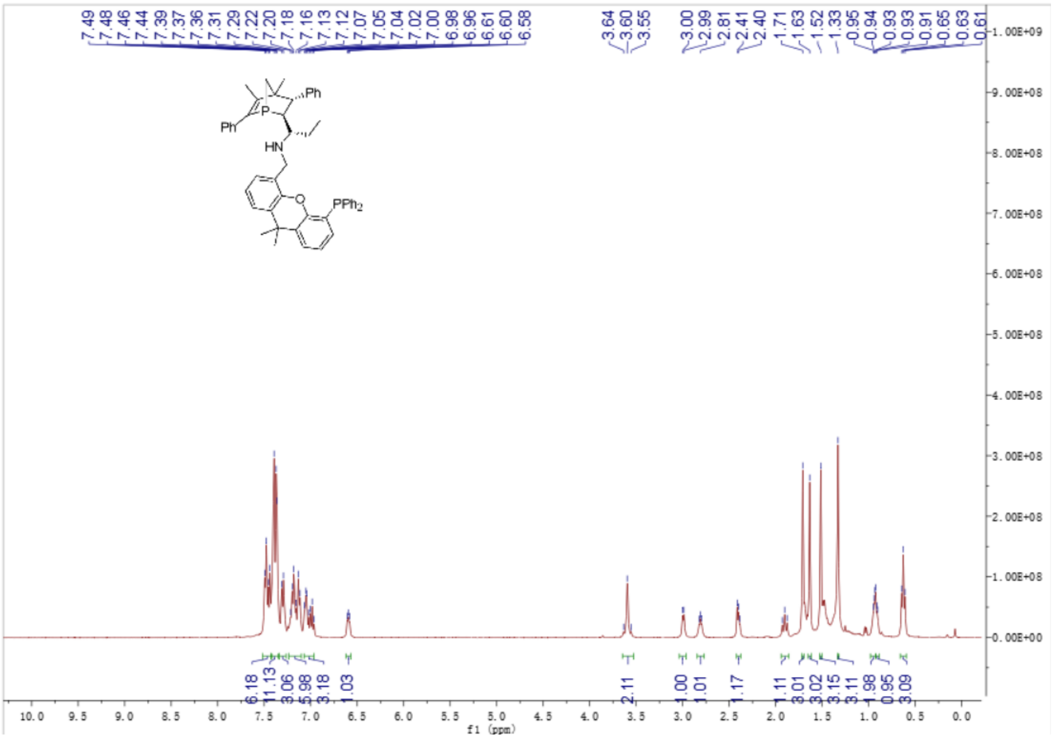

^{1}H ($CDCl_3$, 400 MHz) NMR of compound **ZU-7**

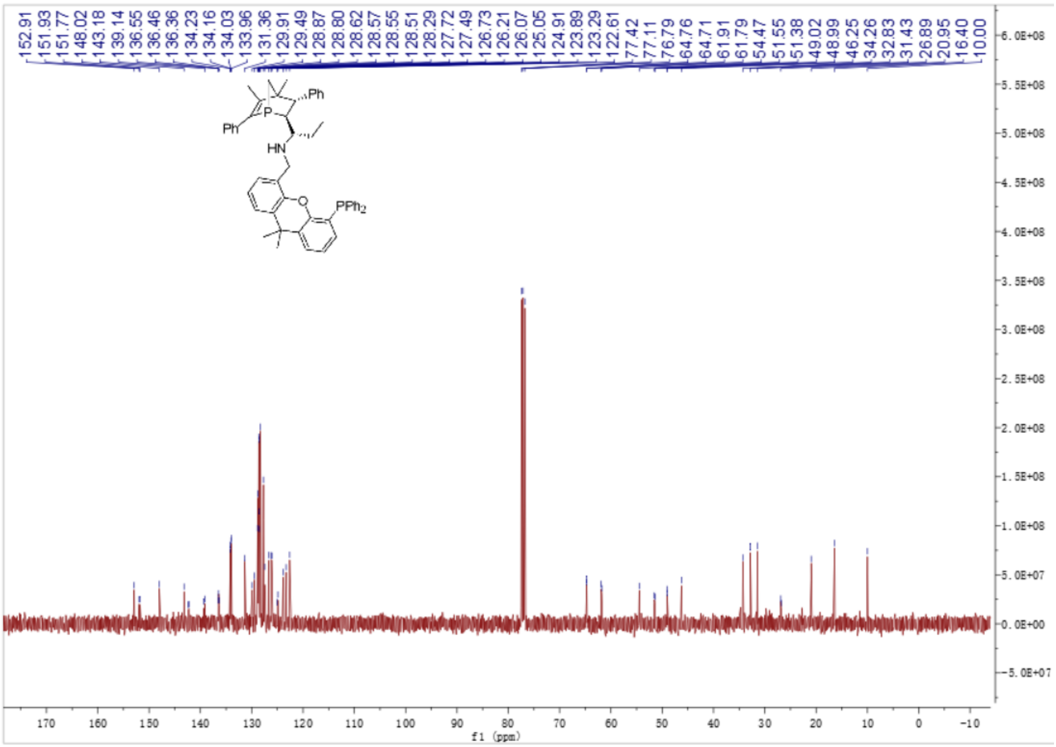

^{13}C ($CDCl_3$, 75 MHz) NMR of compound **ZU-7**

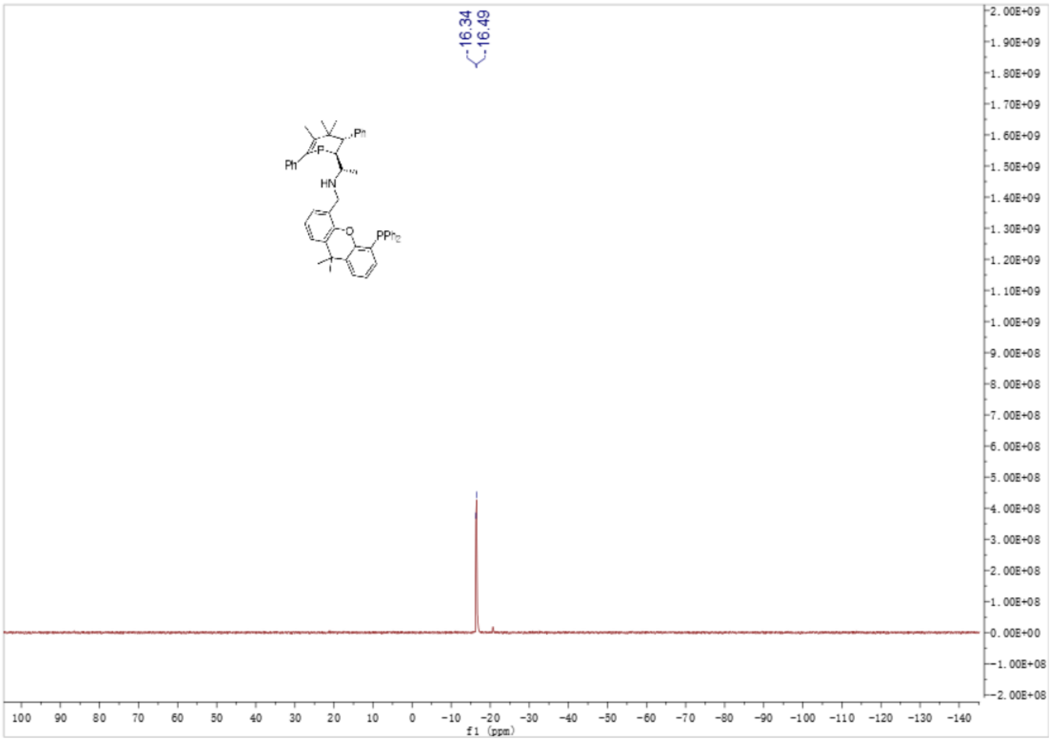

^{31}P ($CDCl_3$, 121 MHz) NMR of compound **ZU-8**

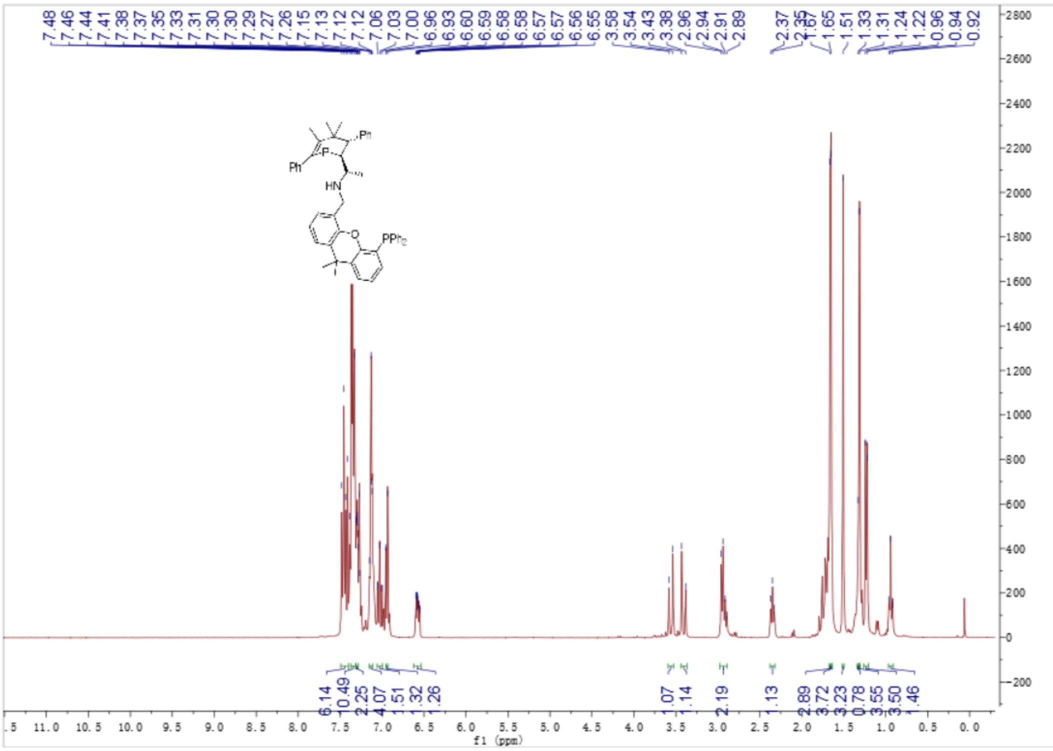

^{1}H ($CDCl_3$, 400 MHz) NMR of compound **ZU-8**

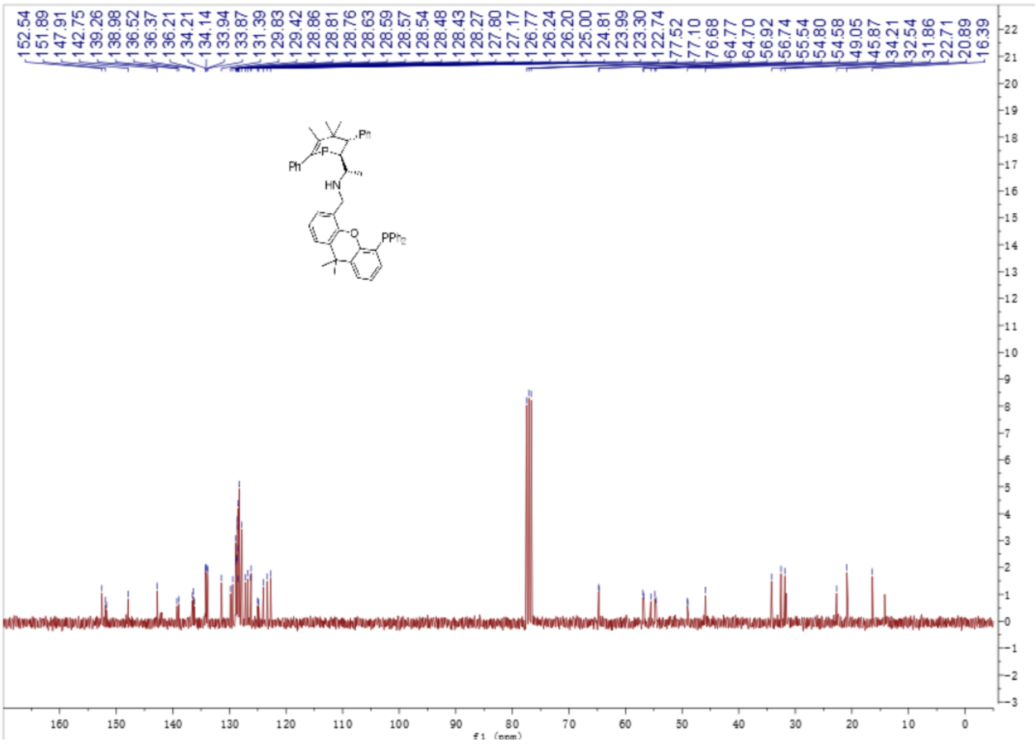

^{13}C ($CDCl_3$, 75 MHz) NMR of compound **ZU-8**

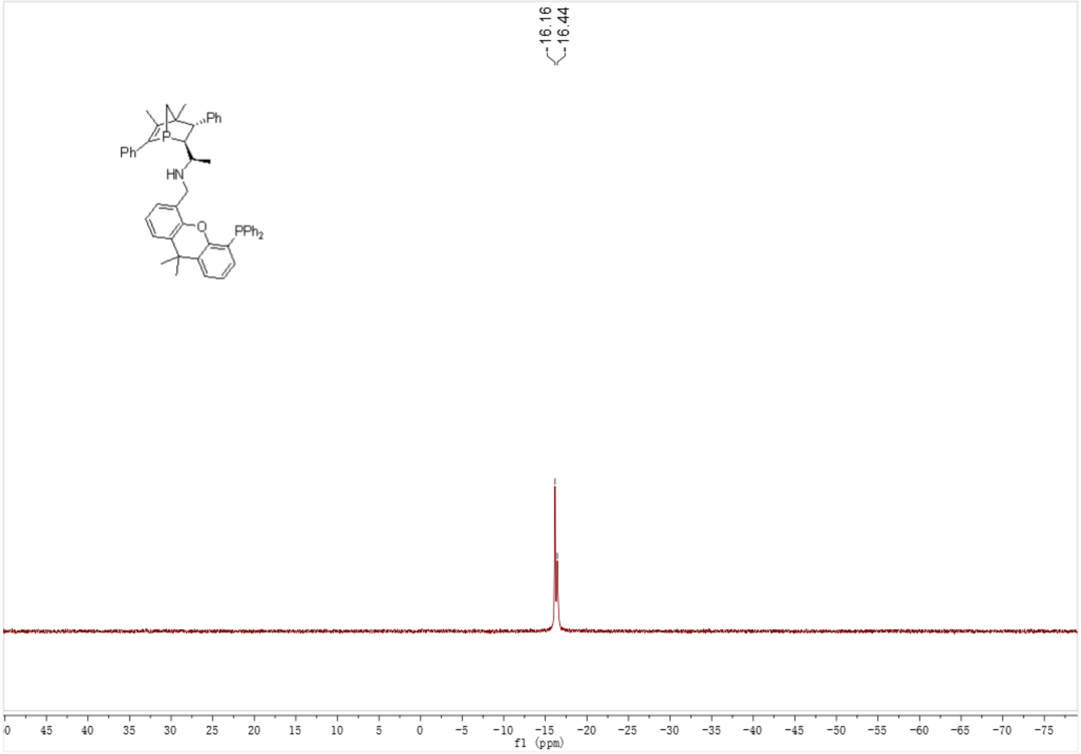

^{31}P ($CDCl_3$, 243 MHz) NMR of compound **ZU-9**

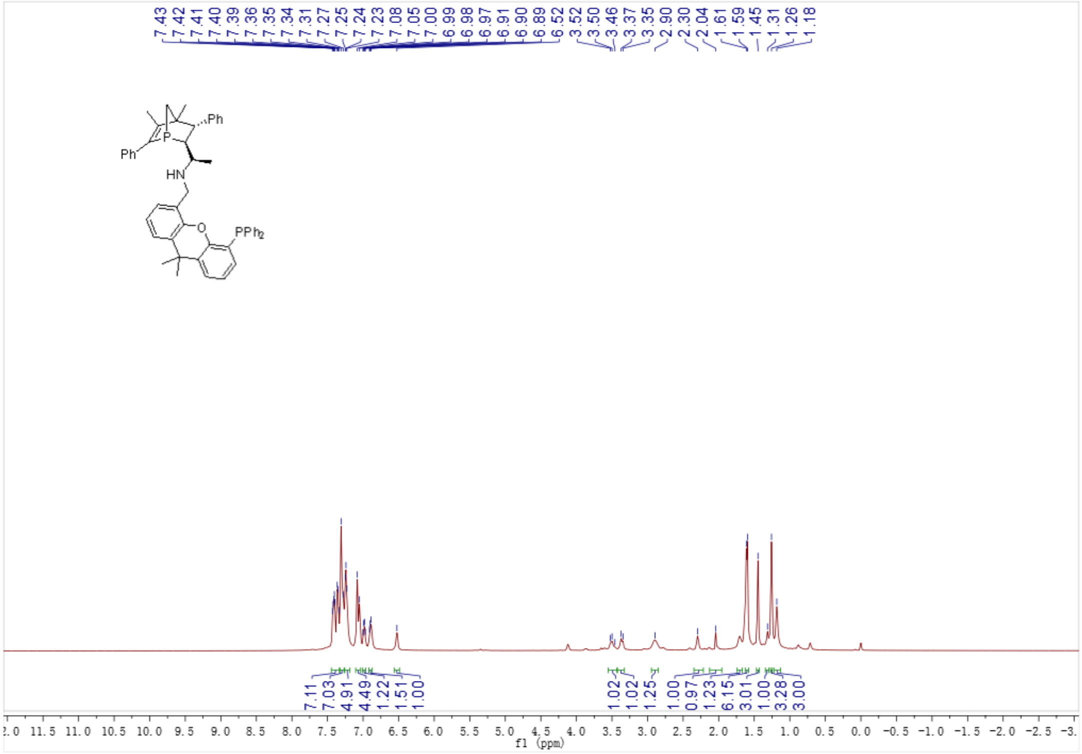

^{1}H ($CDCl_3$, 400 MHz) NMR of compound **ZU-9**

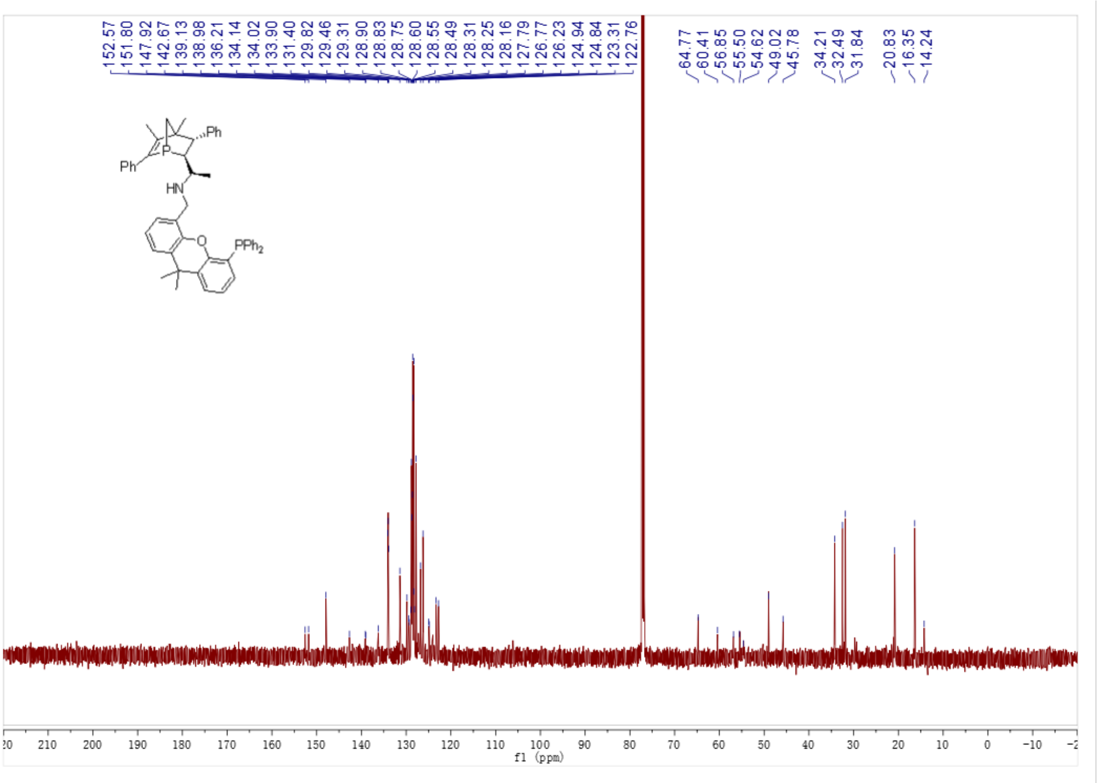

^{13}C ($CDCl_3$, 75 MHz) NMR of compound **ZU-9**

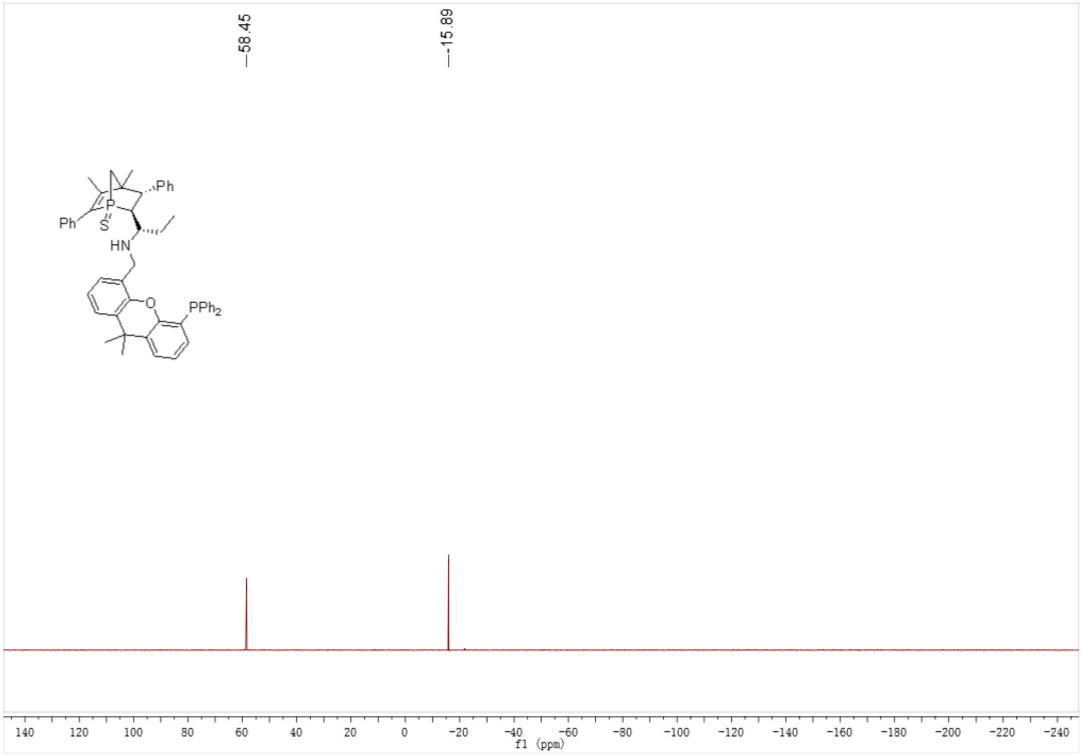

^{31}P ($CDCl_3$, 121 MHz) NMR of compound **ZU-10**

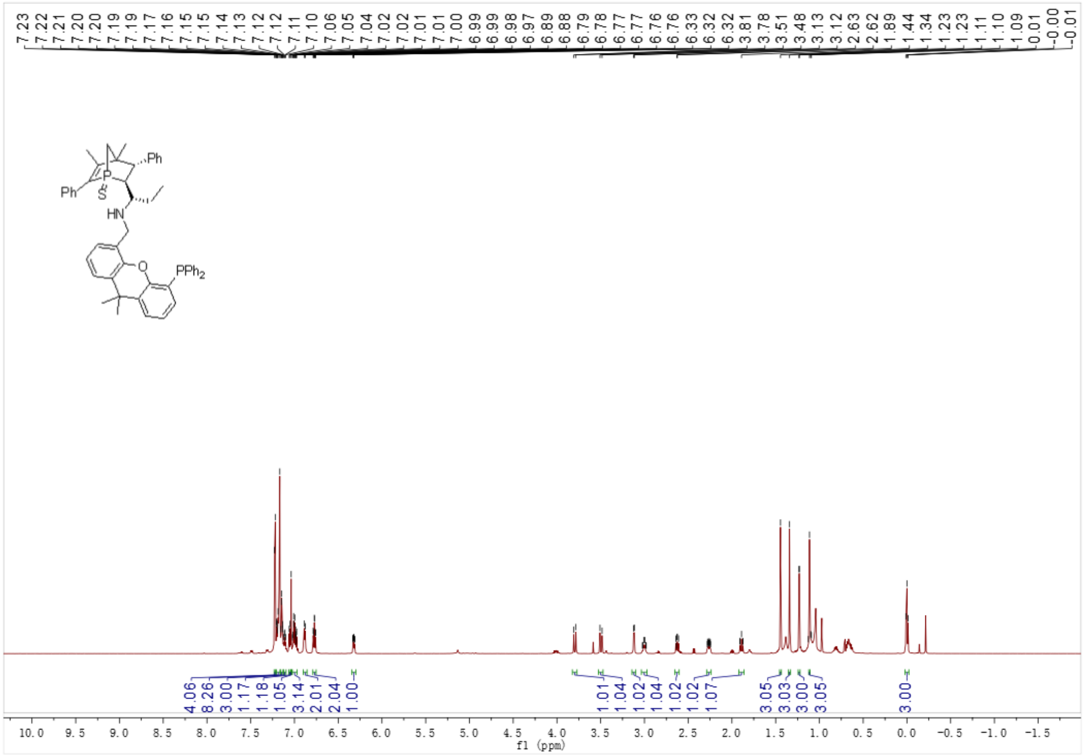

^{1}H ($CDCl_3$, 400 MHz) NMR of compound **ZU-10**

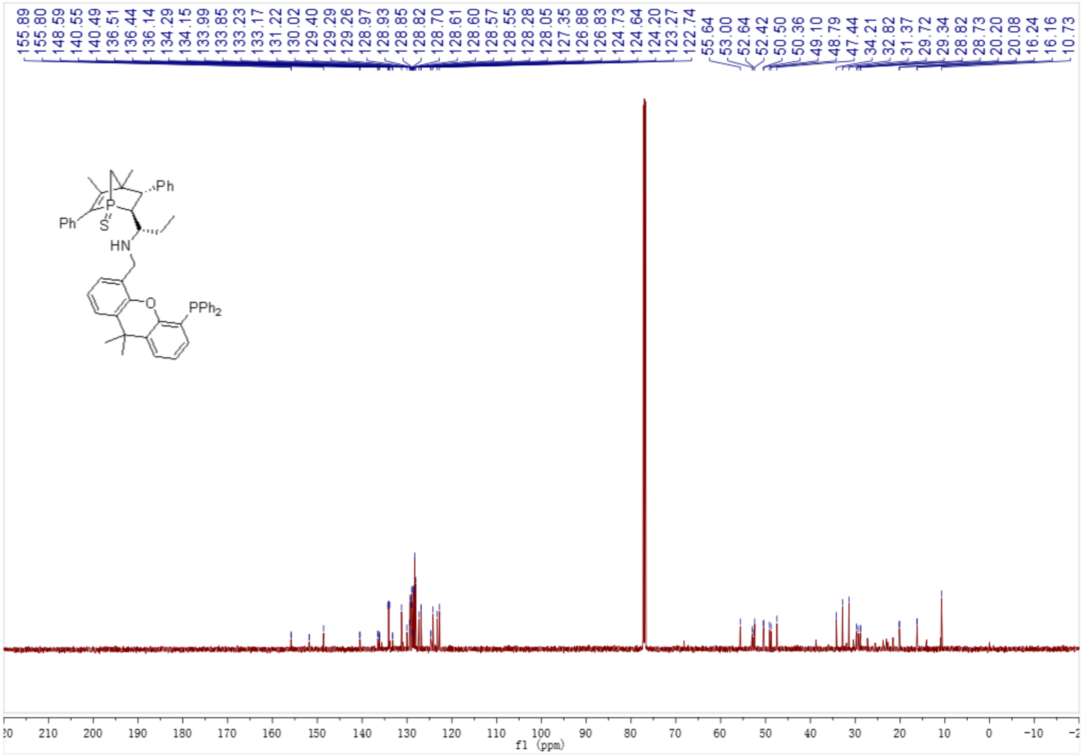

^{13}C ($CDCl_3$, 75 MHz) NMR of compound **ZU-10**

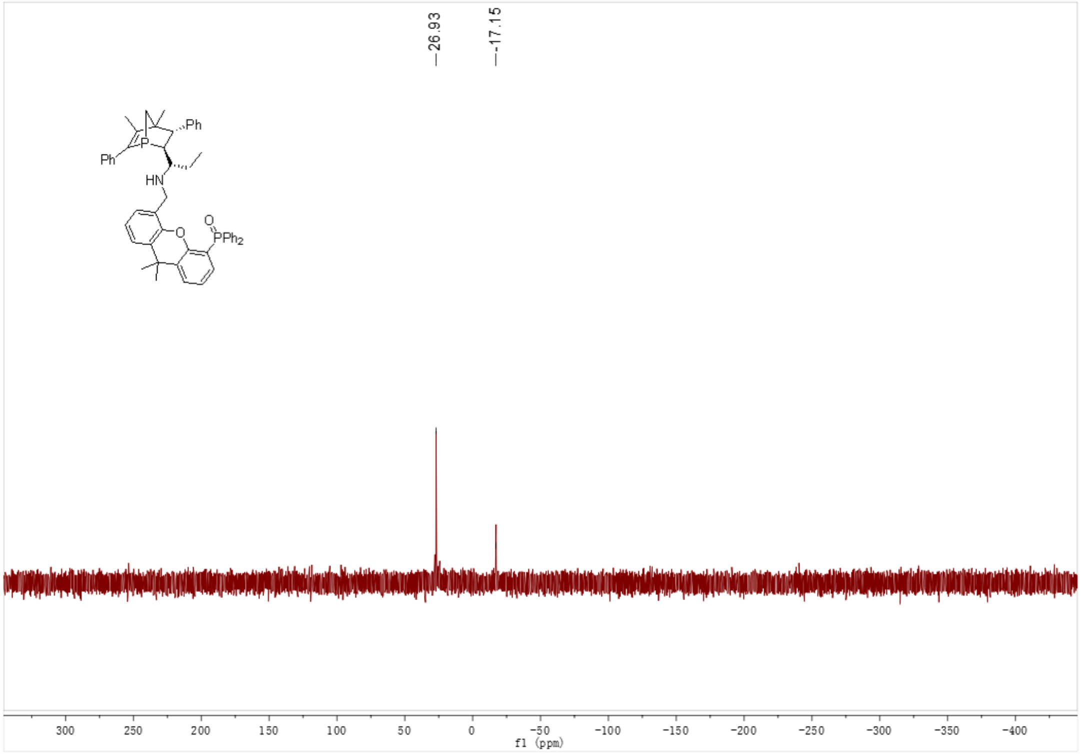

^{31}P ($CDCl_3$, 121 MHz) NMR of compound **ZU-11**

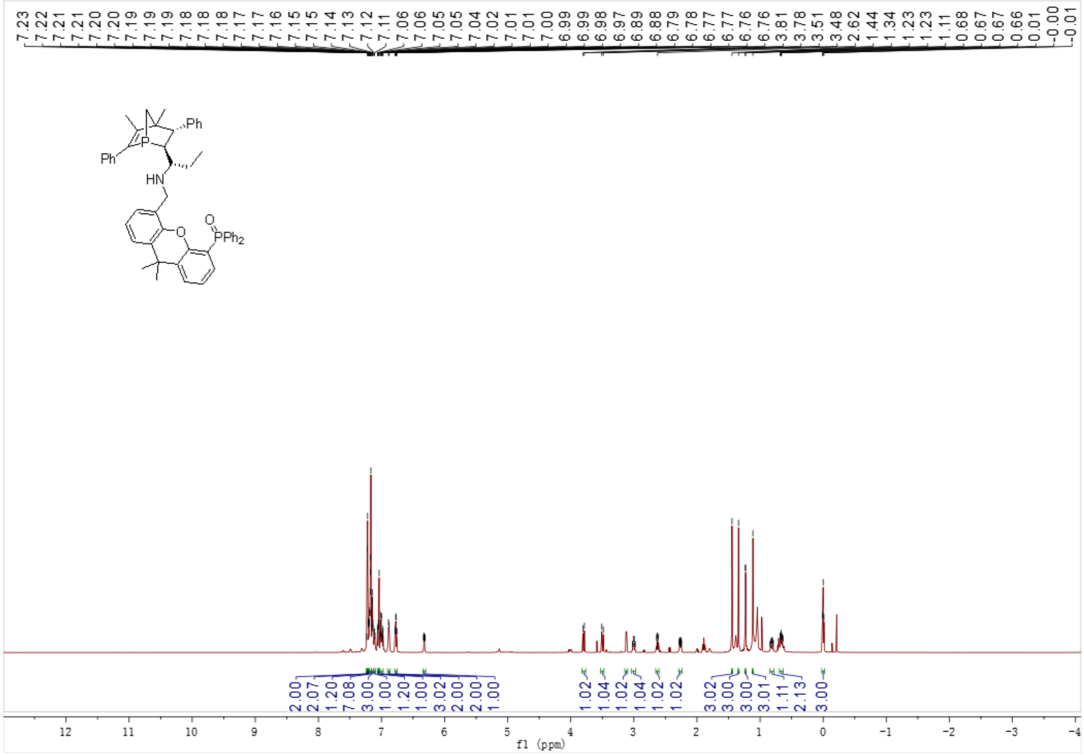

^{1}H ($CDCl_3$, 600 MHz) NMR of compound **ZU-11**

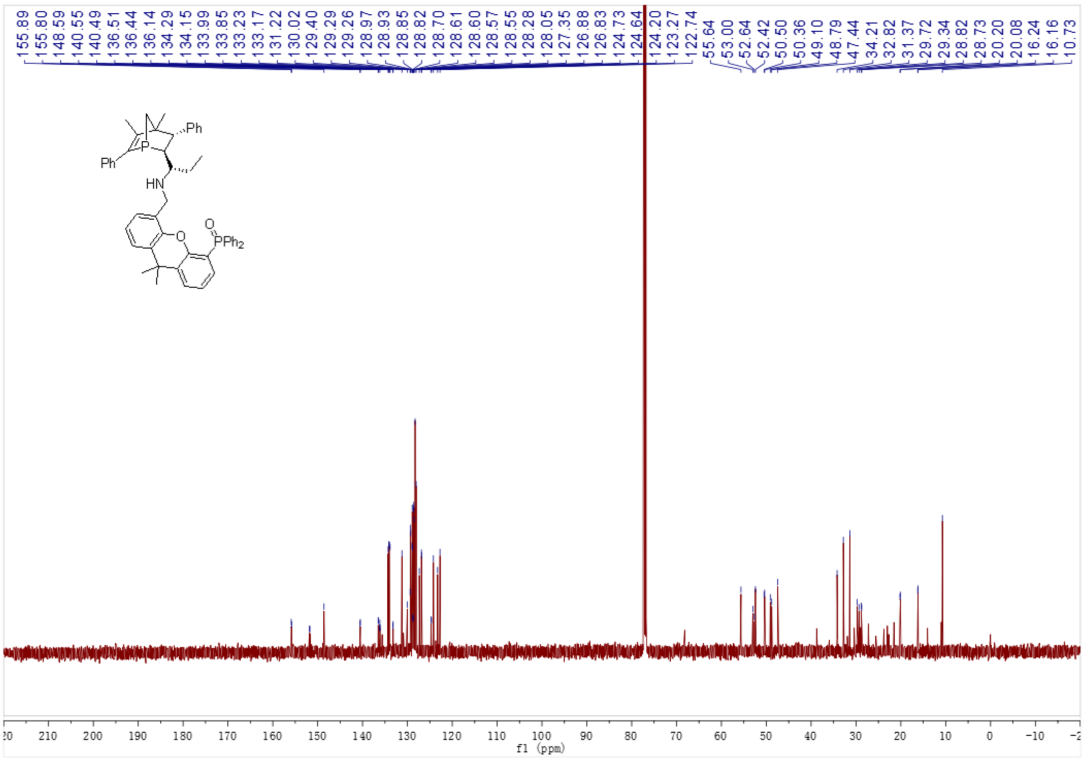

^{13}C ($CDCl_3$, 151 MHz) NMR of compound **ZU-11**

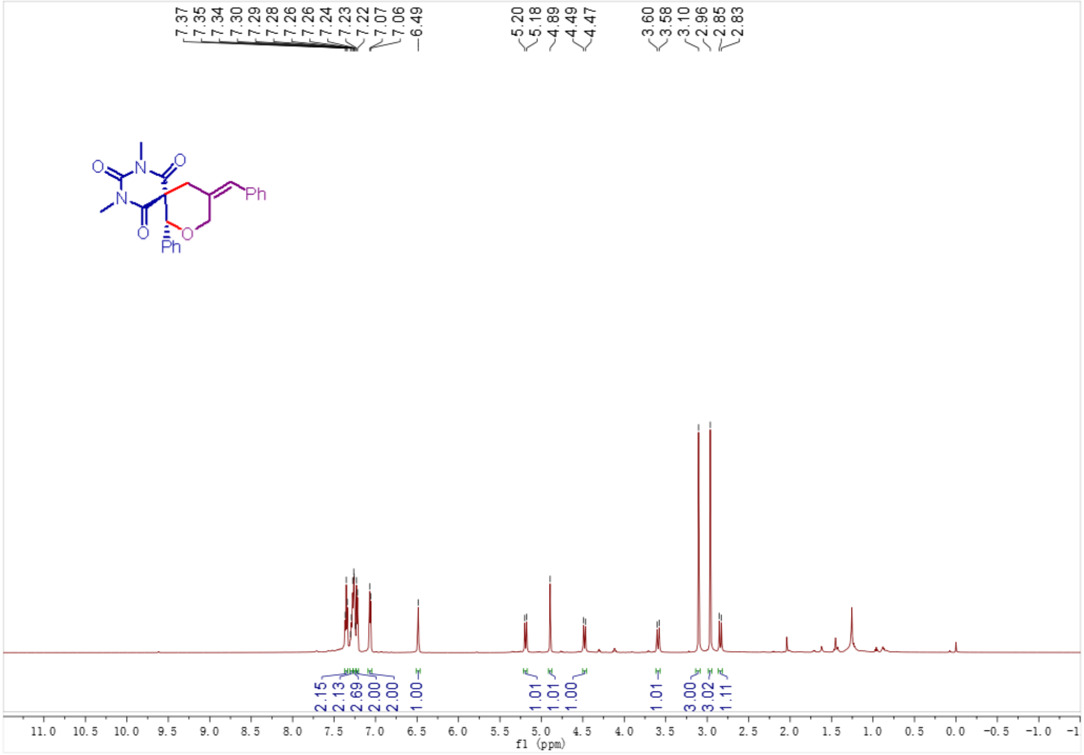

^{1}H ($CDCl_3$, 600 MHz) NMR of compound **3**

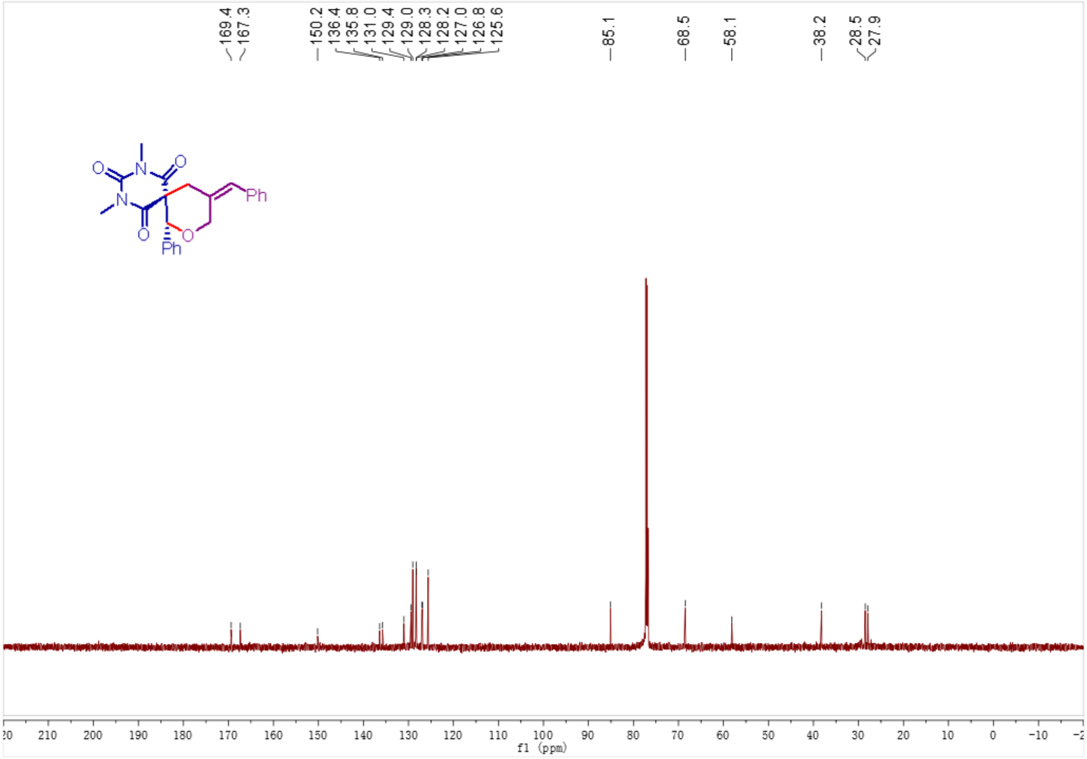

^{13}C ($CDCl_3$, 151 MHz) NMR of compound **3**

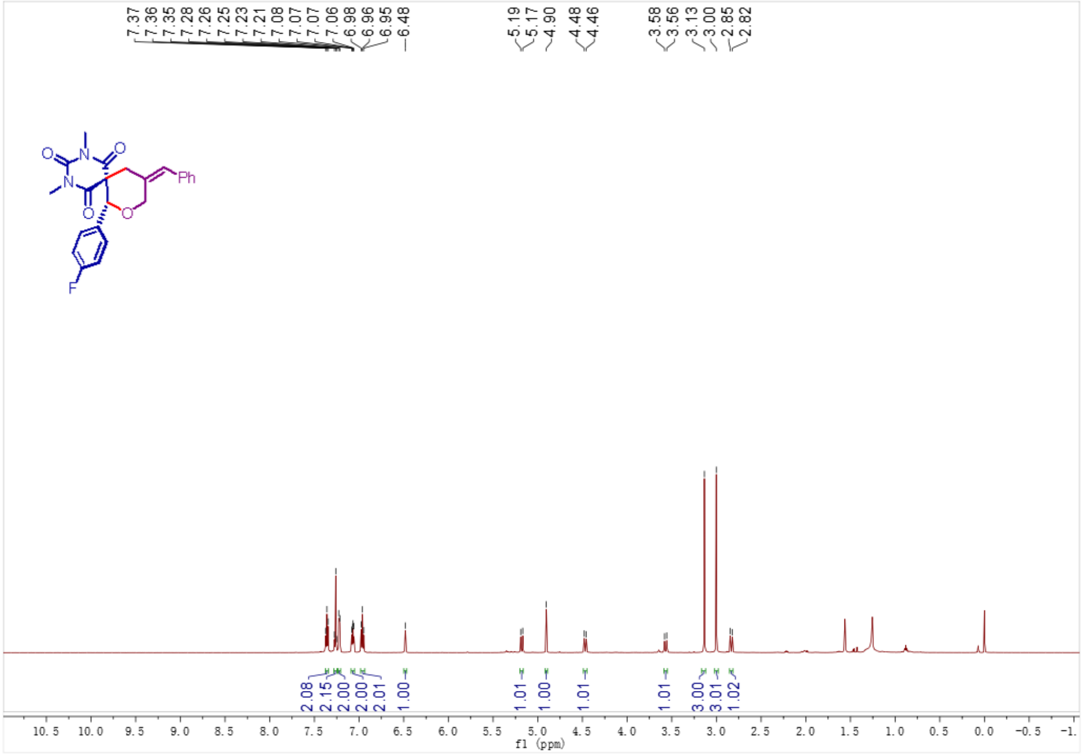

^{1}H ($CDCl_3$, 600 MHz) NMR of compound **4**

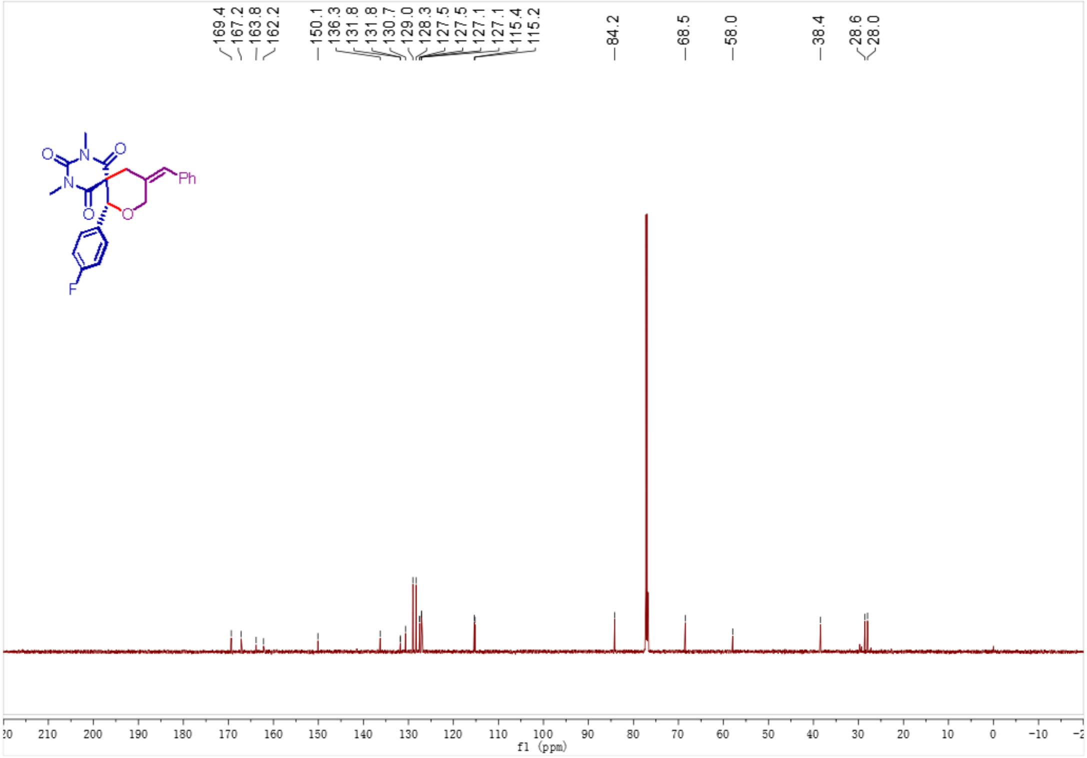

^{13}C (CDCl$_3$, 151 MHz) NMR of compound **4**

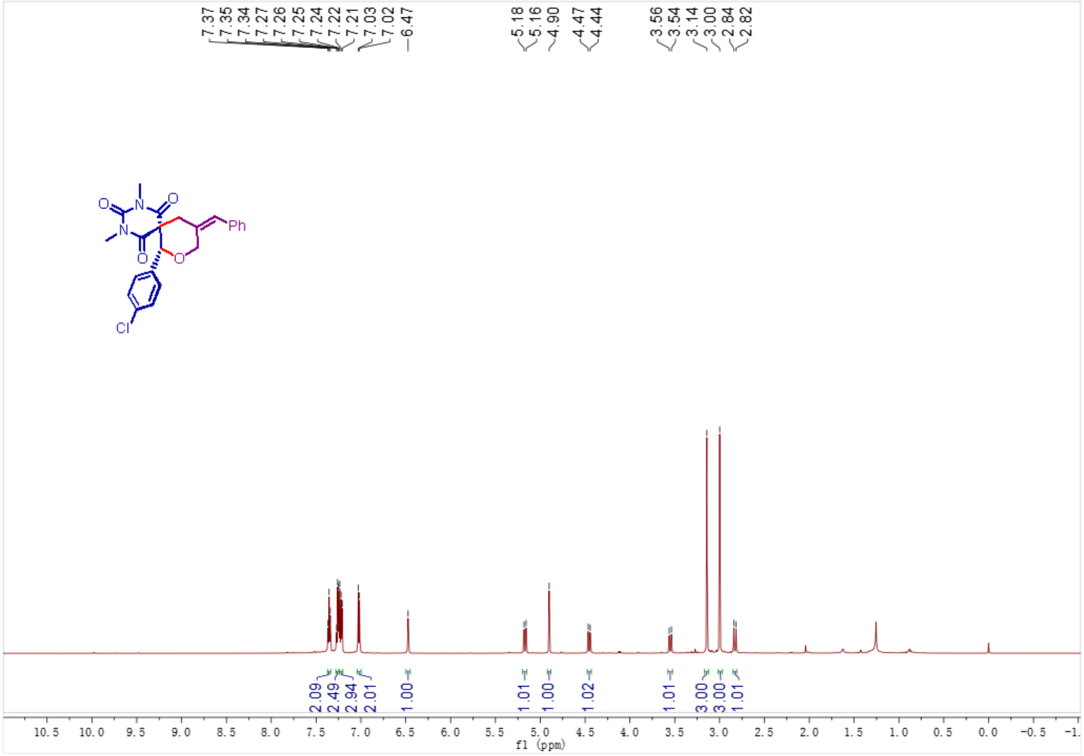

^{1}H (CDCl$_3$, 600 MHz) NMR of compound **5**

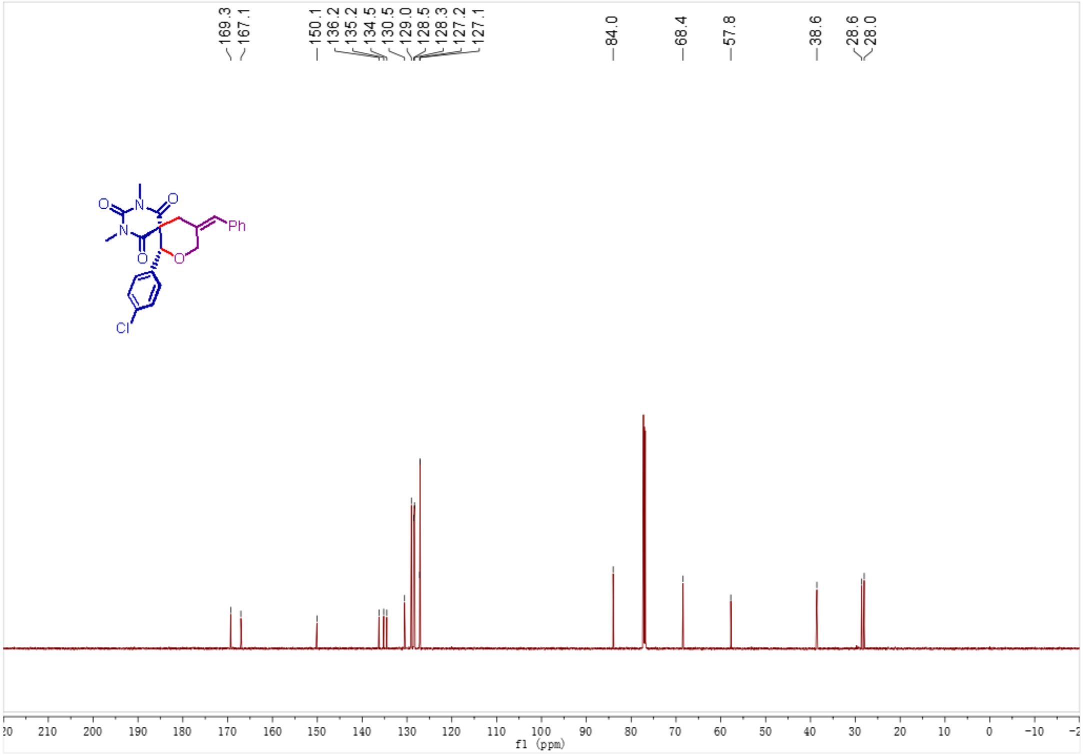

^{13}C ($CDCl_3$, 151 MHz) NMR of compound **5**

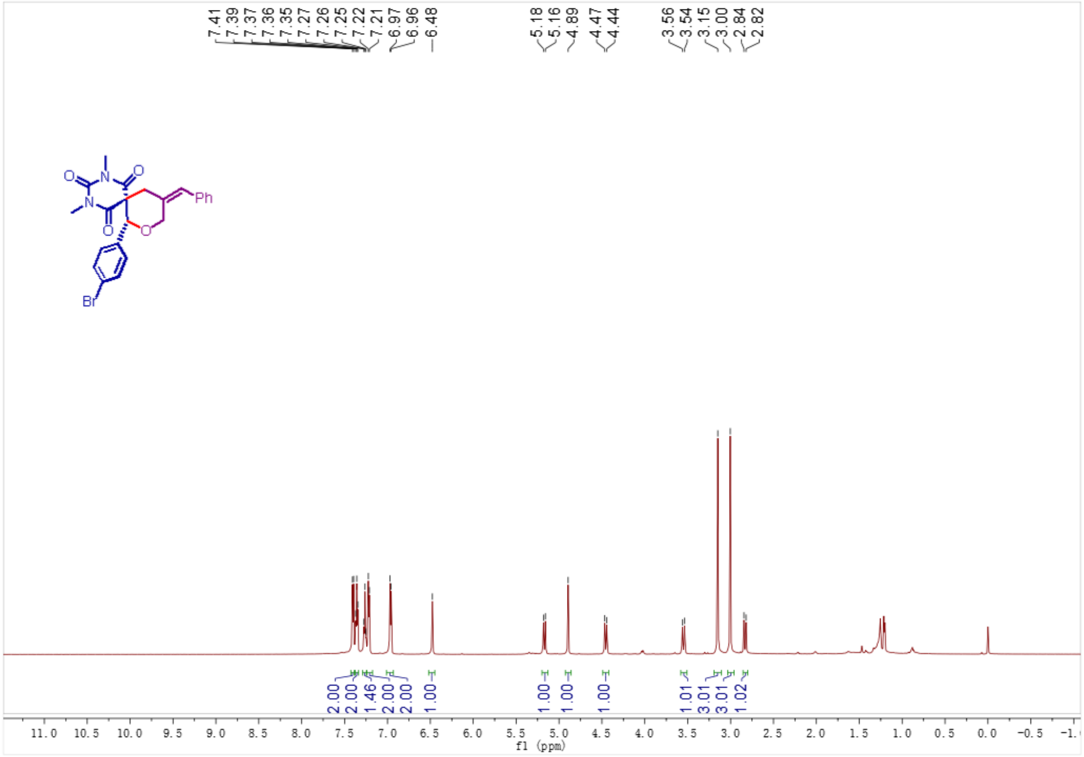

^{1}H ($CDCl_3$, 600 MHz) NMR of compound **6**

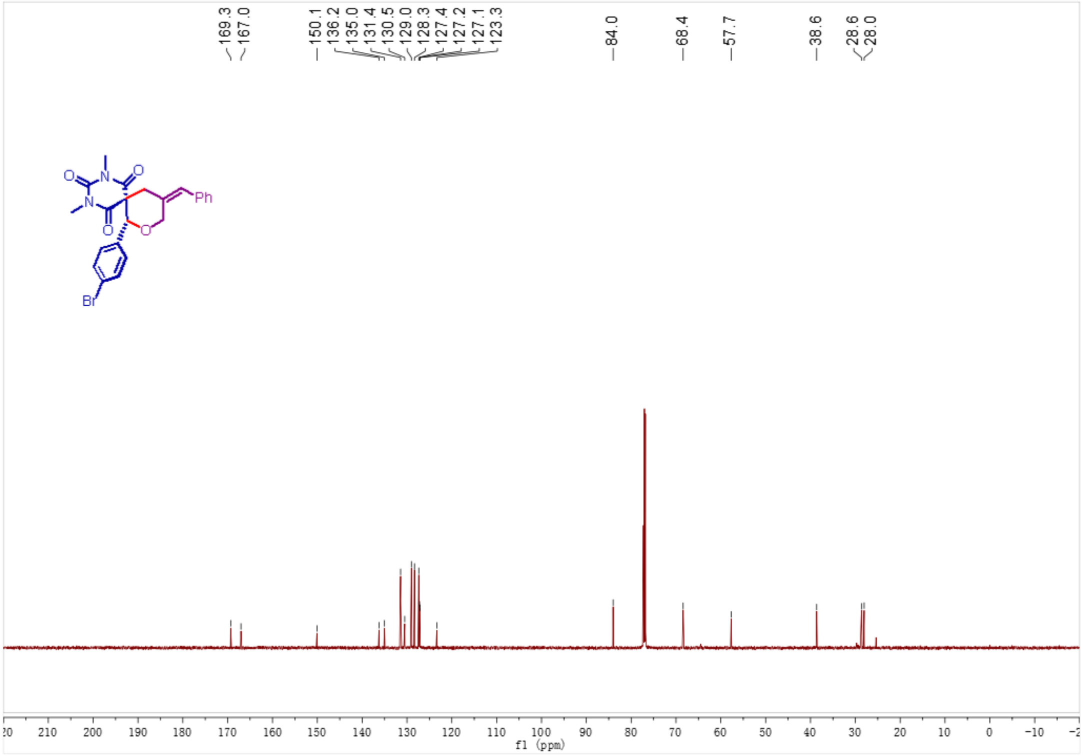

^{13}C ($CDCl_3$, 151 MHz) NMR of compound **6**

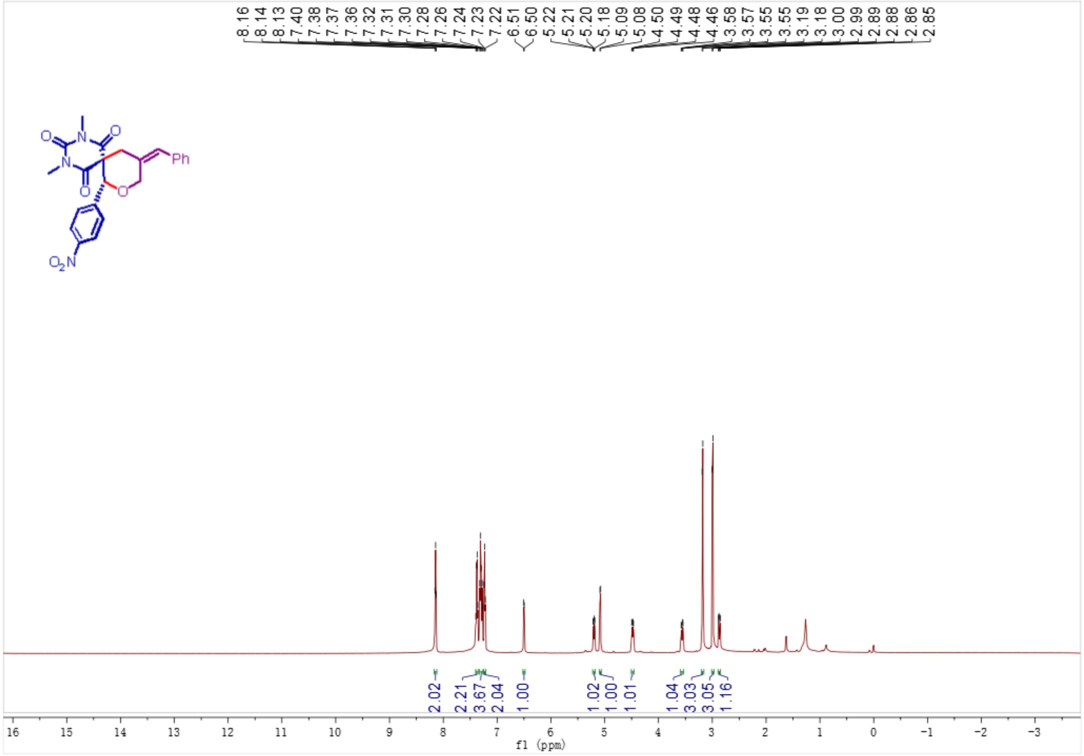

^{1}H ($CDCl_3$, 600 MHz) NMR of compound **7**

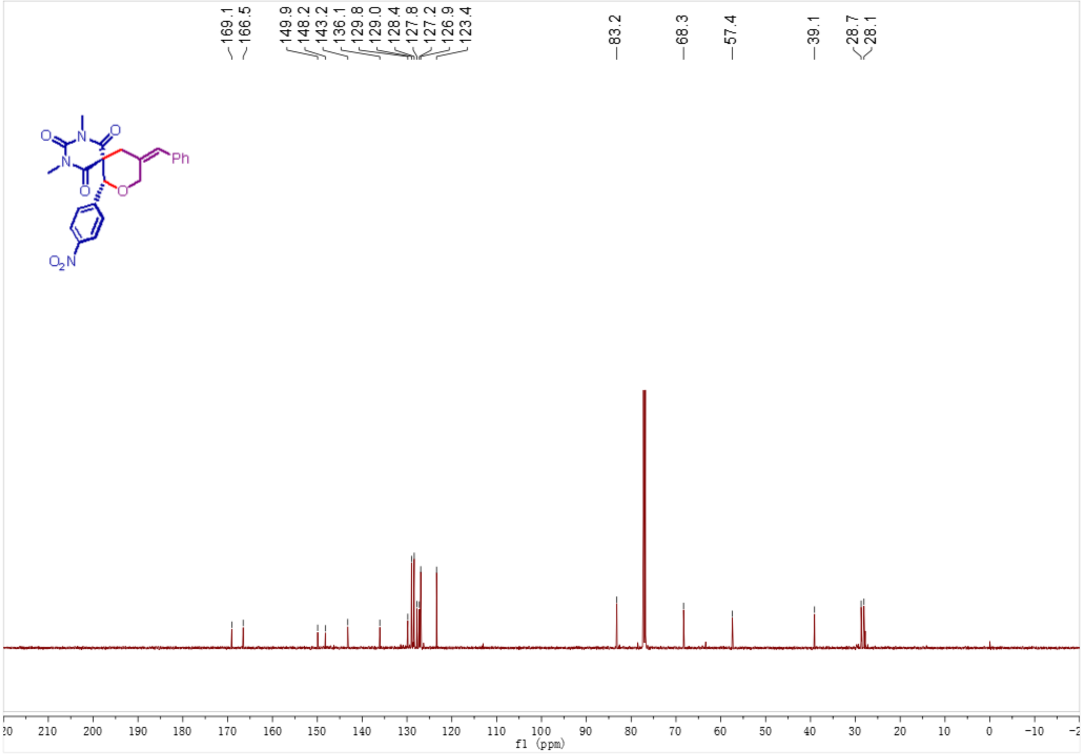

^{13}C ($CDCl_3$, 151 MHz) NMR of compound **7**

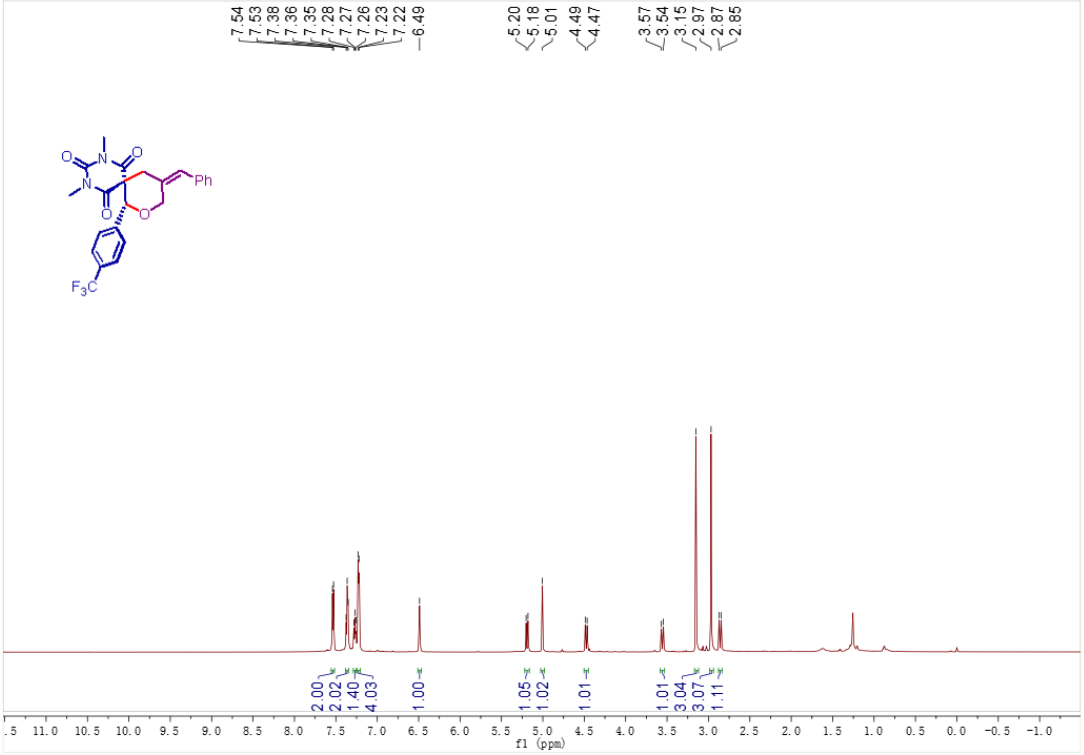

^{1}H ($CDCl_3$, 600 MHz) NMR of compound **8**

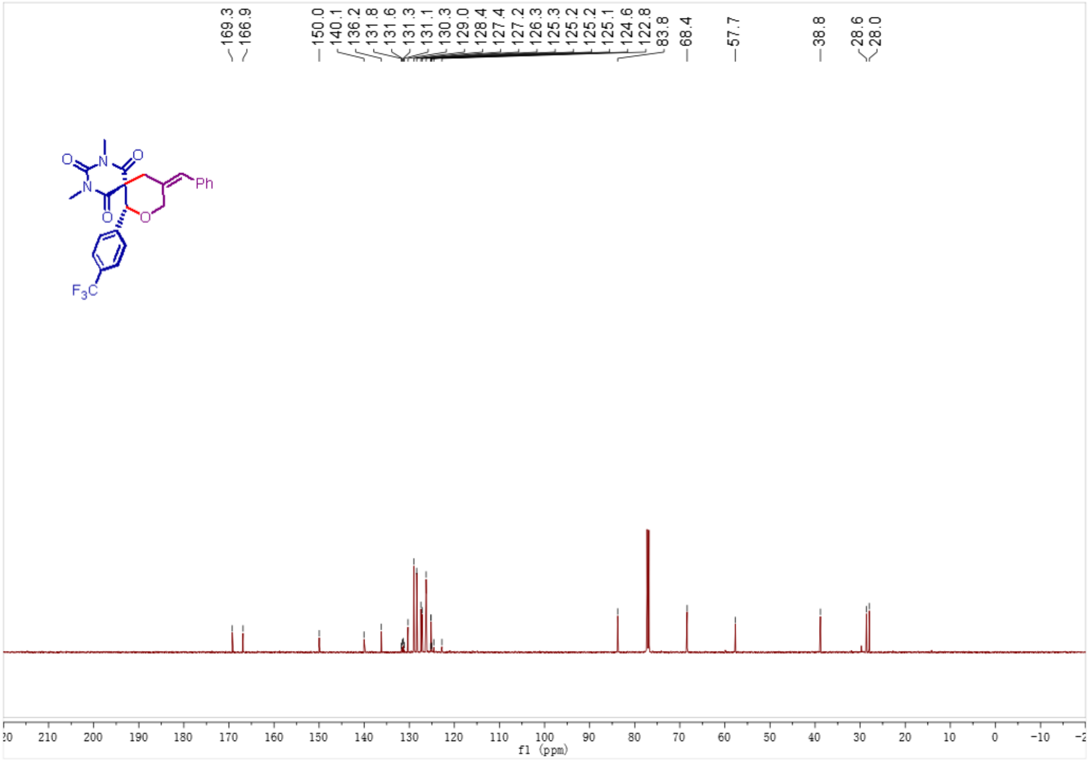

^{13}C ($CDCl_3$, 151 MHz) NMR of compound **8**

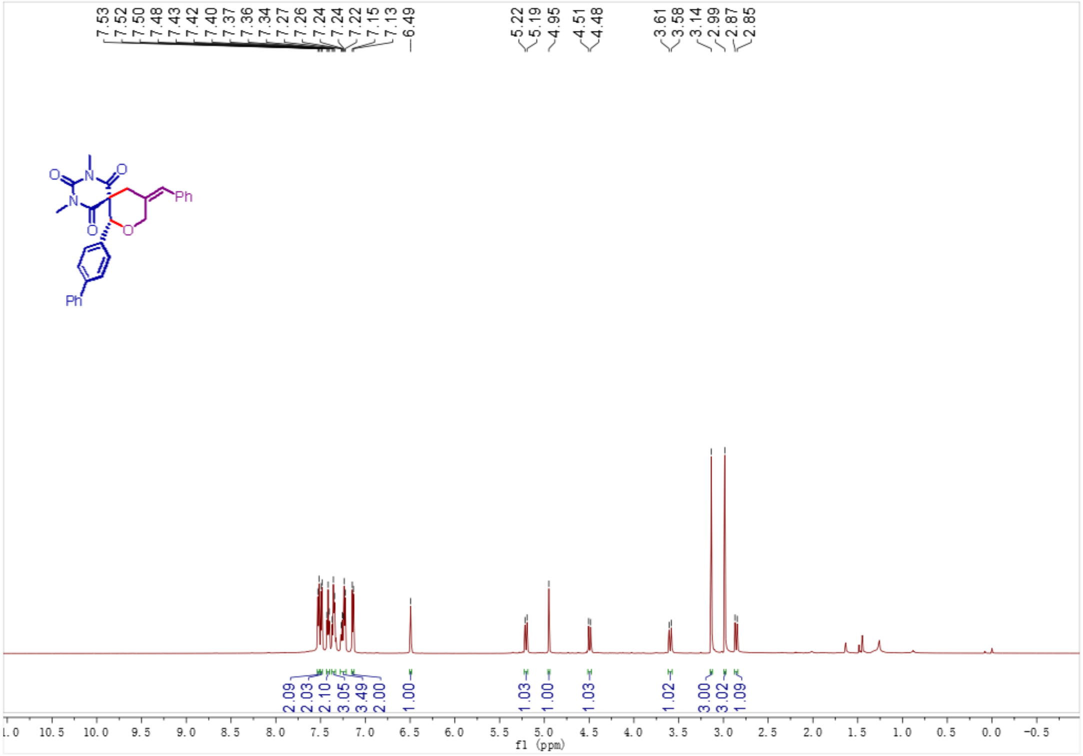

^{1}H ($CDCl_3$, 600 MHz) NMR of compound **9**

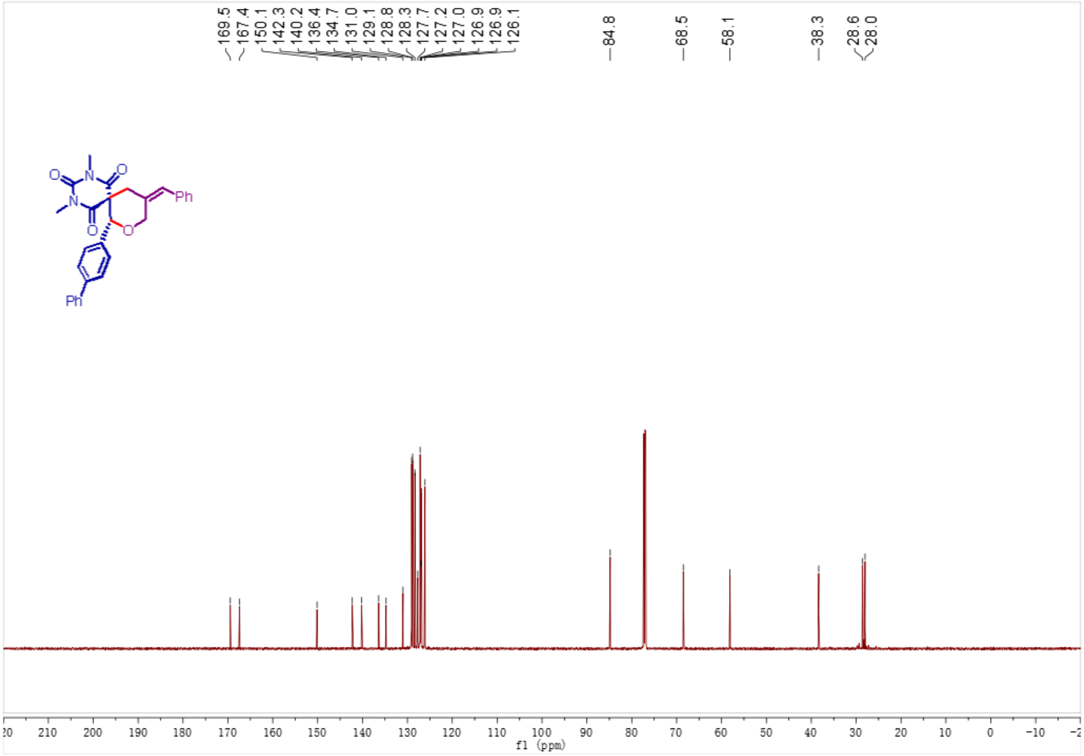

^{13}C ($CDCl_3$, 151 MHz) NMR of compound **9**

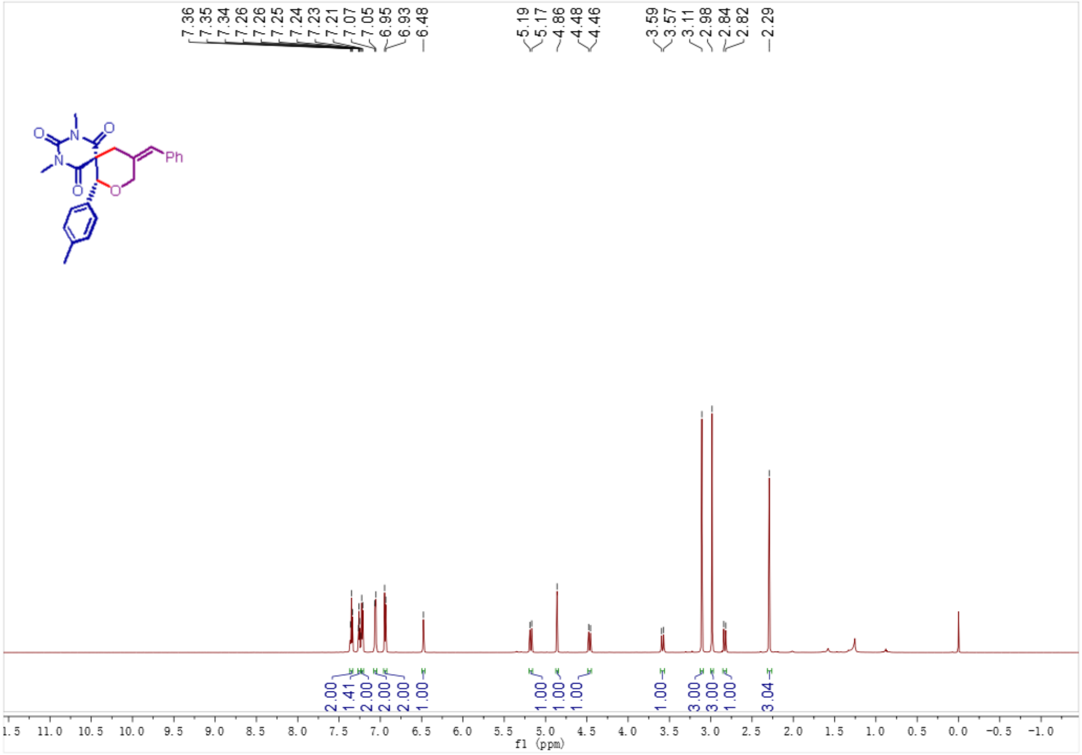

^{1}H ($CDCl_3$, 600 MHz) NMR of compound **10**

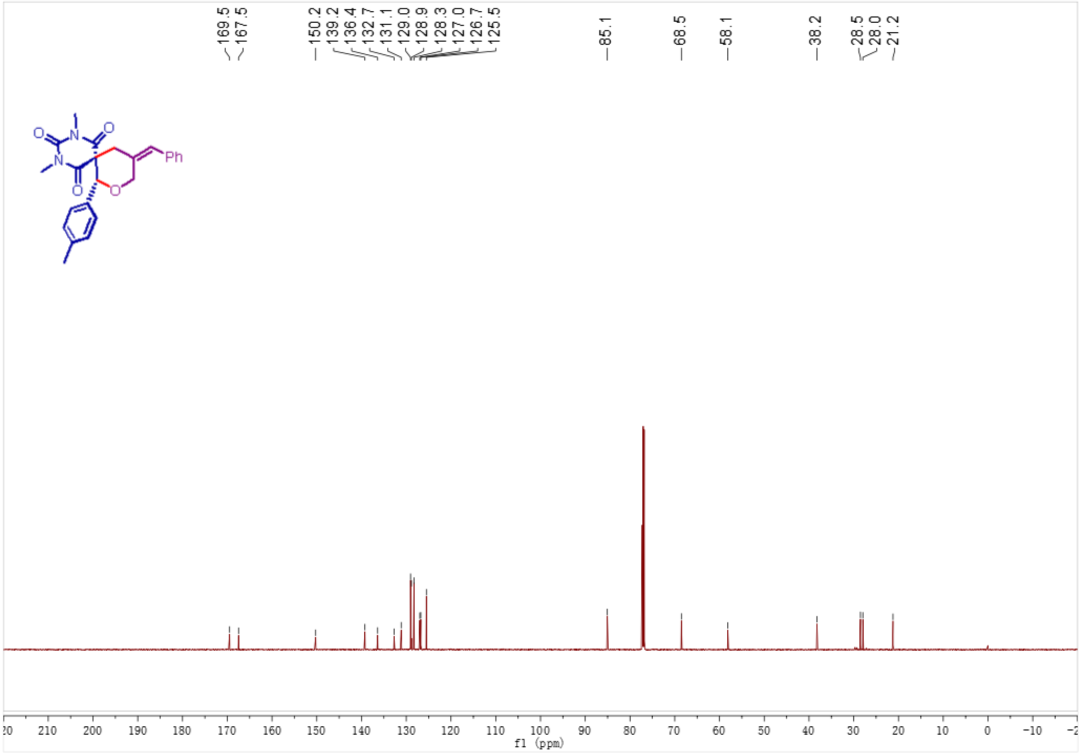

^{13}C ($CDCl_3$, 151 MHz) NMR of compound **10**

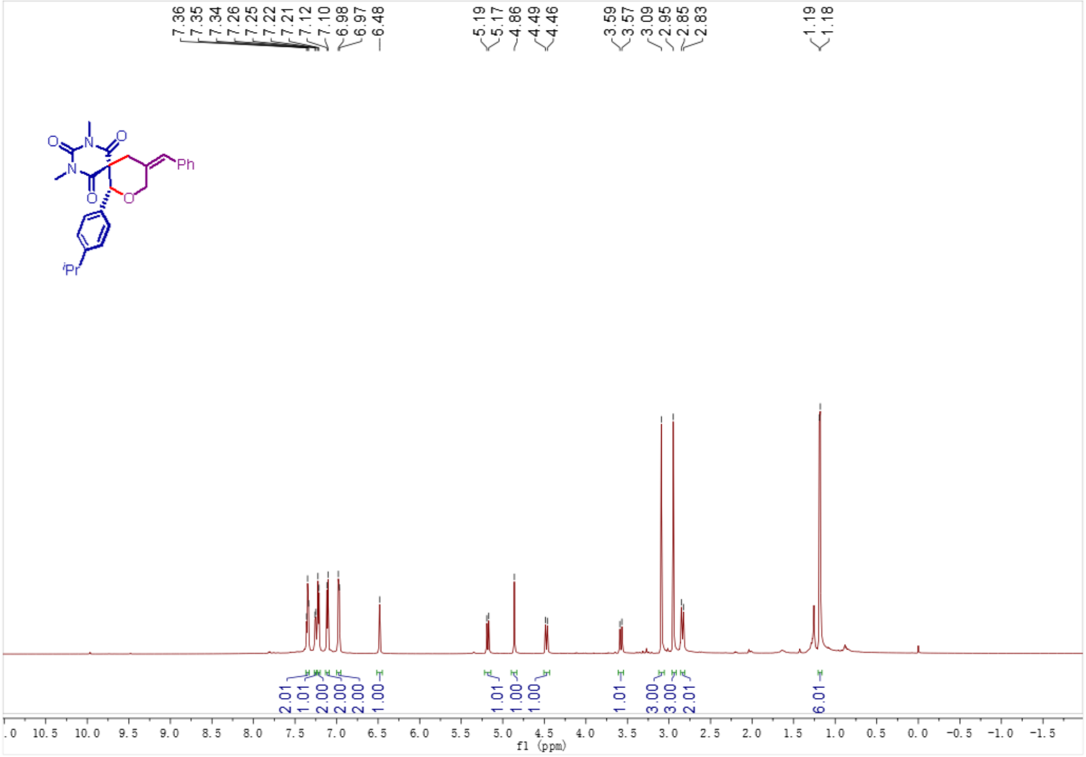

^{1}H ($CDCl_3$, 600 MHz) NMR of compound **11**

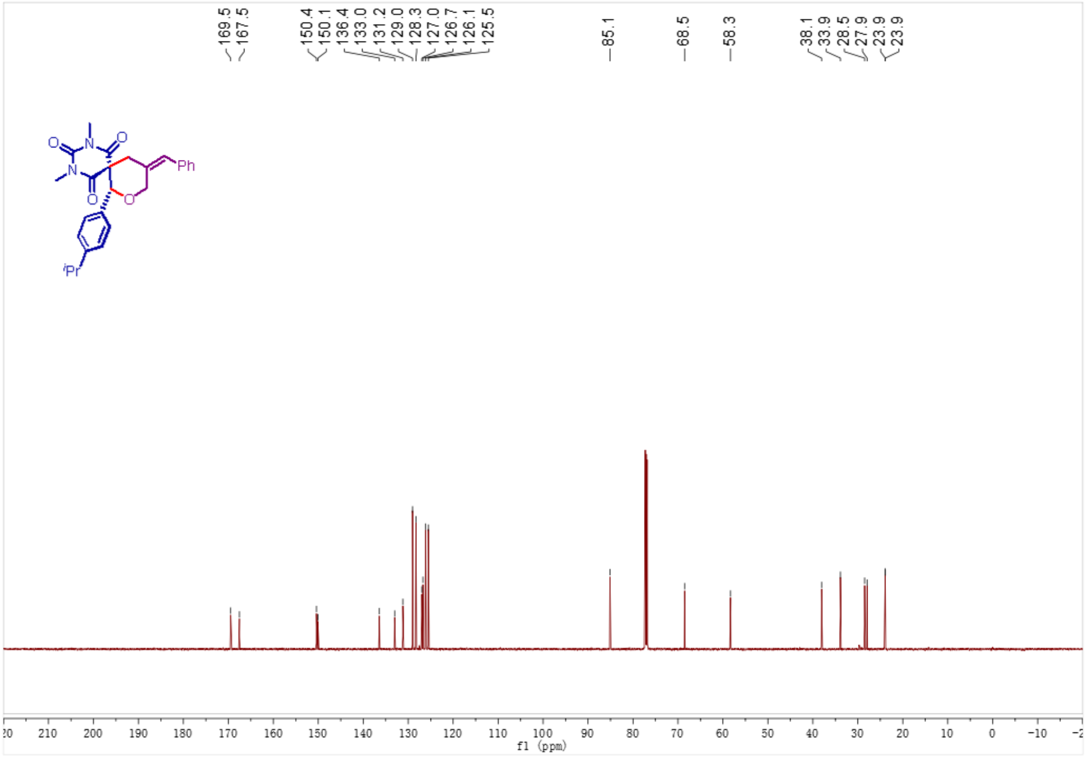

^{13}C ($CDCl_3$, 151 MHz) NMR of compound **11**

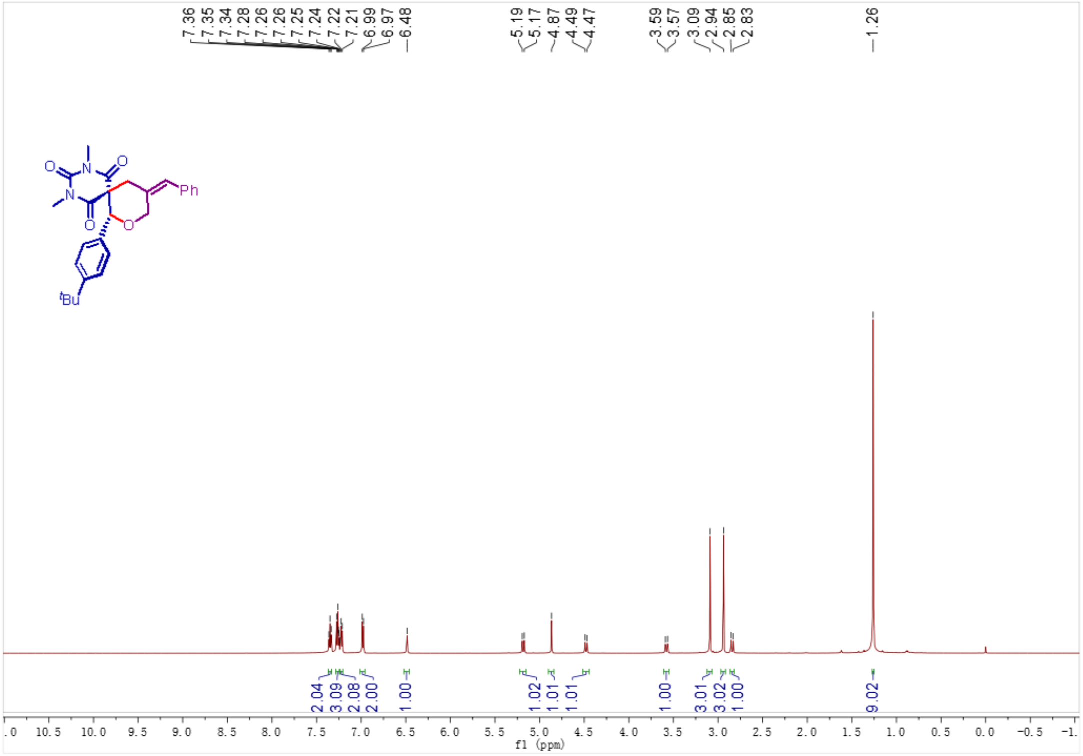

^{1}H ($CDCl_3$, 600 MHz) NMR of compound **12**

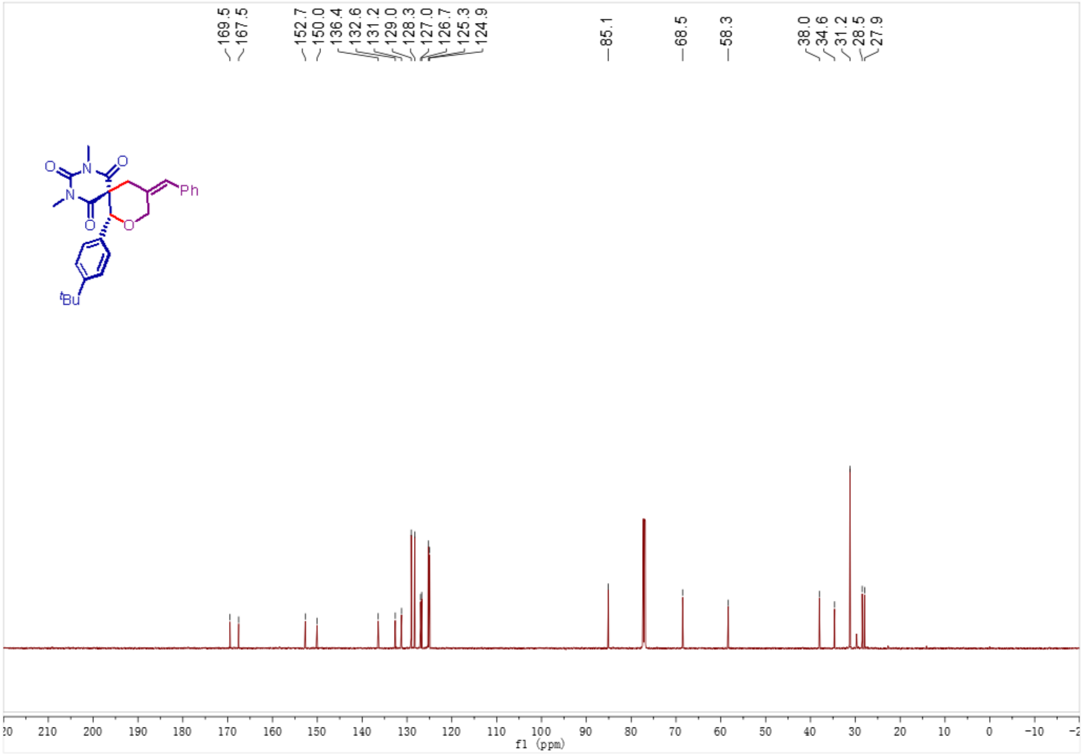

^{13}C ($CDCl_3$, 151 MHz) NMR of compound **12**

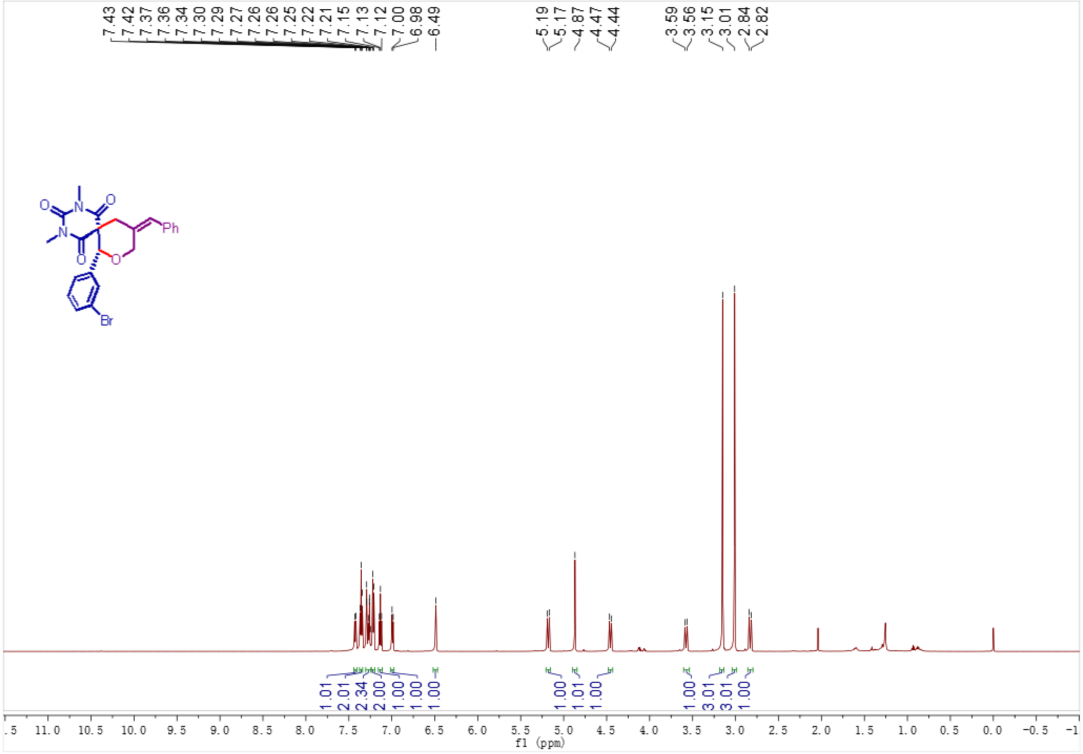

^{1}H ($CDCl_3$, 600 MHz) NMR of compound **13**

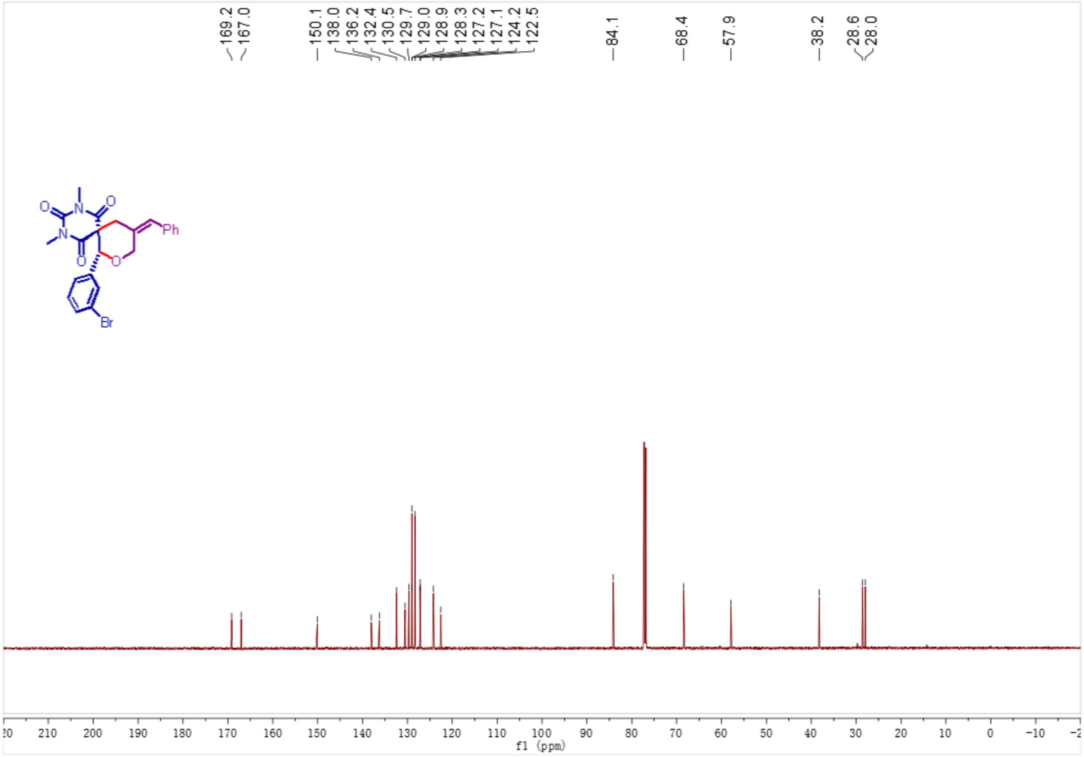

^{13}C ($CDCl_3$, 151 MHz) NMR of compound **13**

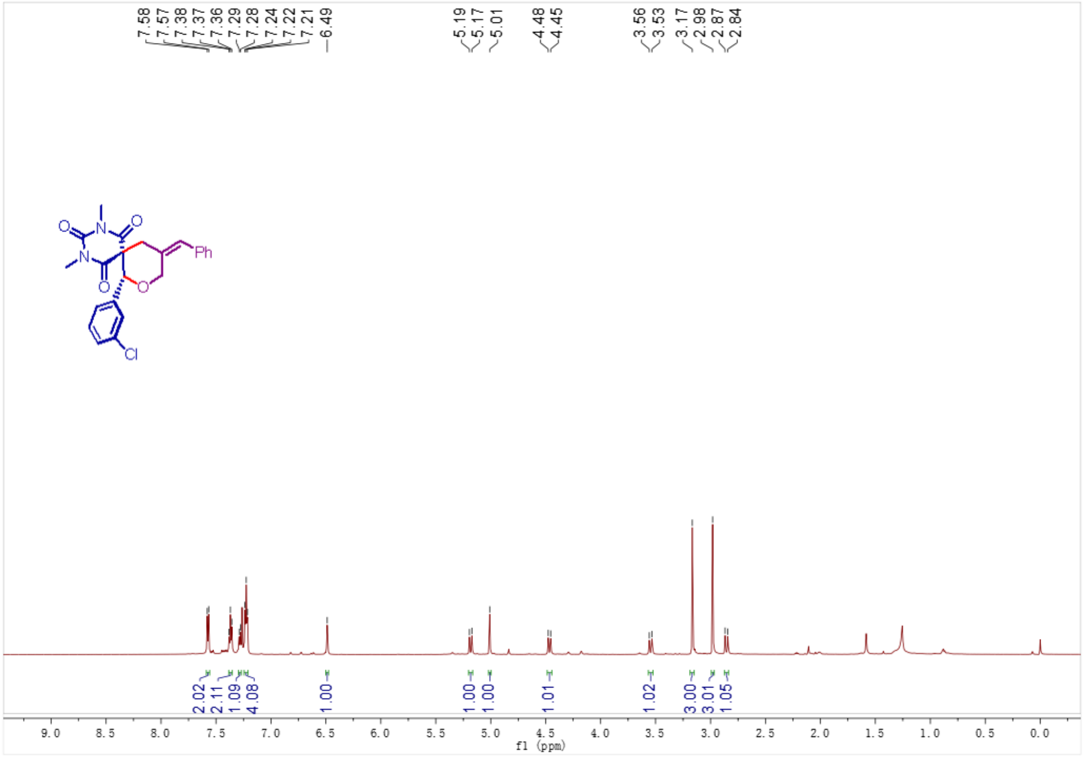

^{1}H ($CDCl_3$, 600 MHz) NMR of compound **14**

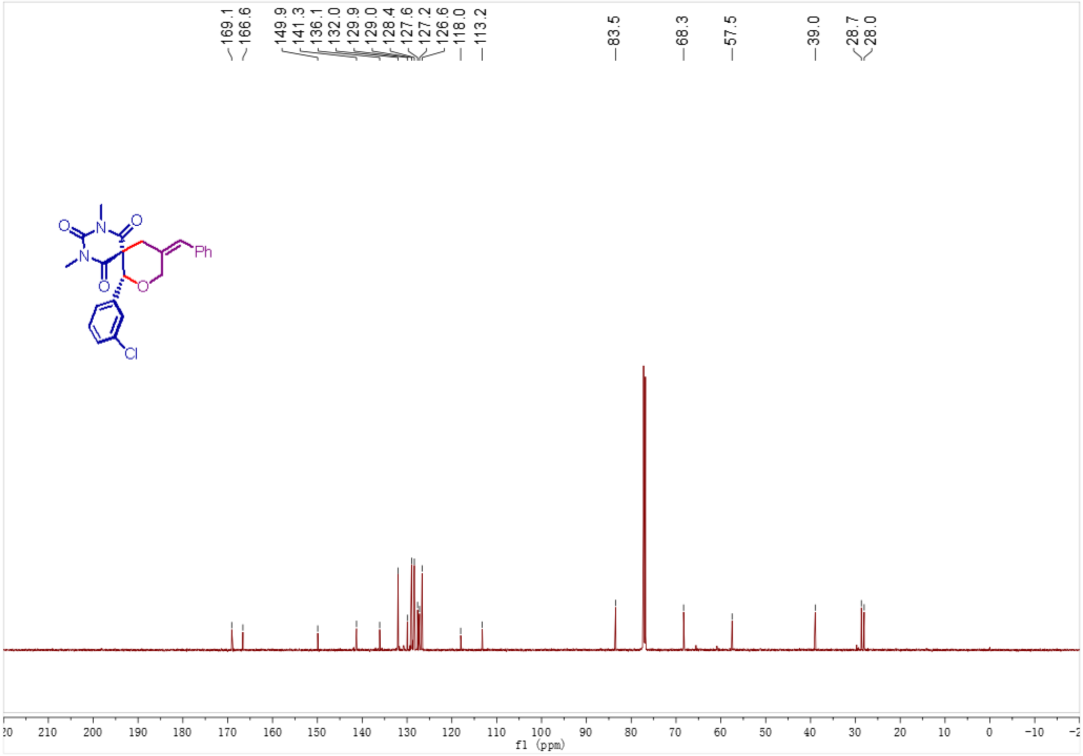

^{13}C ($CDCl_3$, 151 MHz) NMR of compound **14**

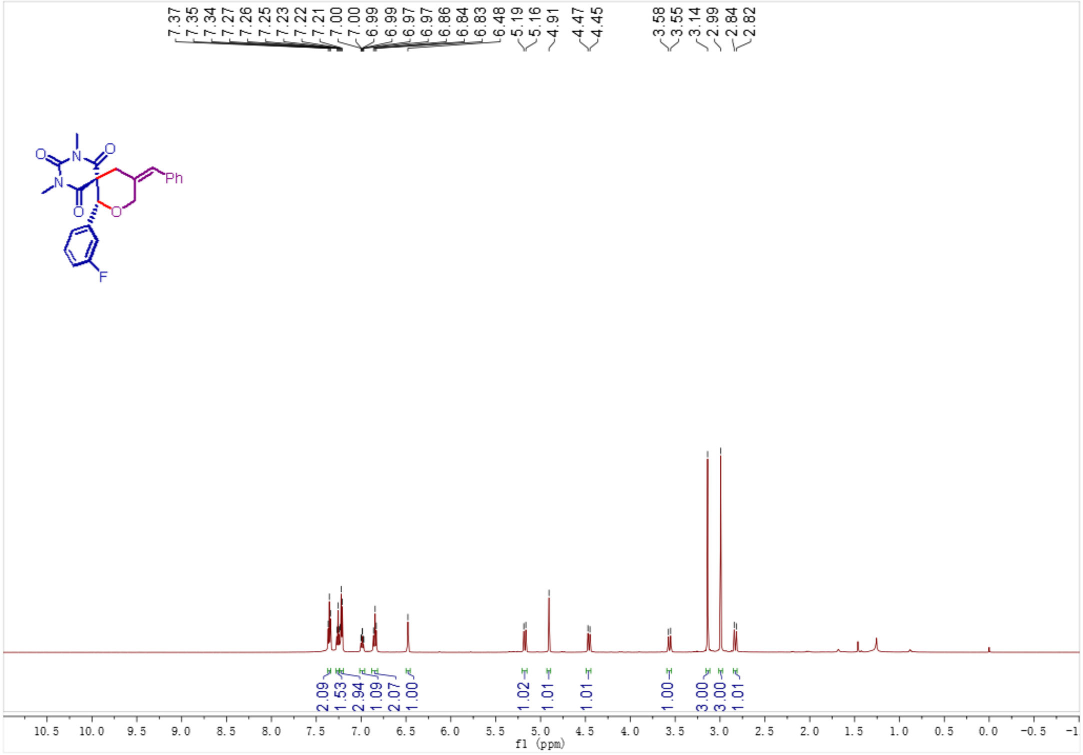

^{1}H ($CDCl_3$, 600 MHz) NMR of compound **15**

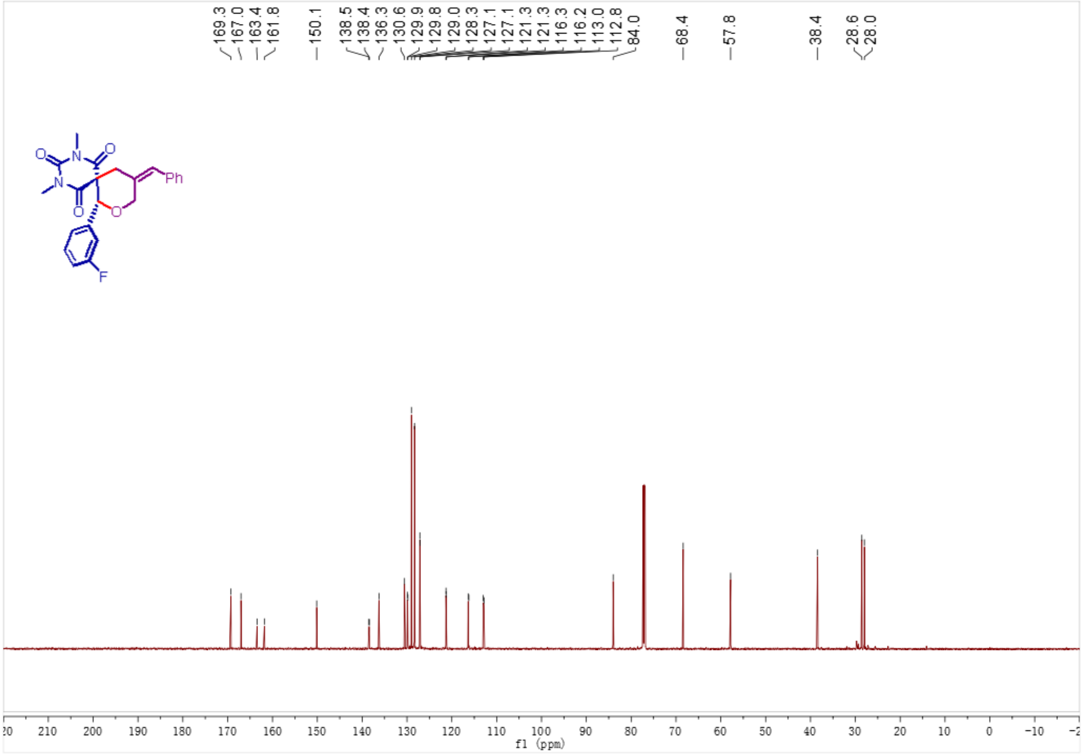

^{13}C ($CDCl_3$, 151 MHz) NMR of compound **15**

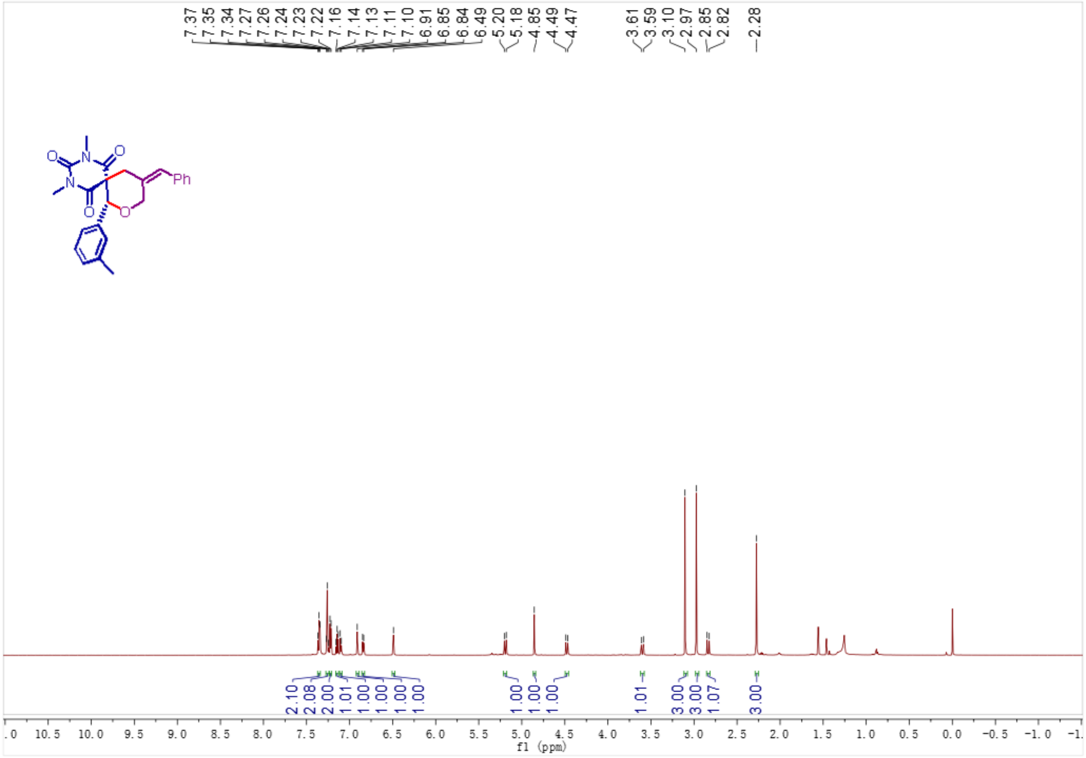

^{1}H ($CDCl_3$, 600 MHz) NMR of compound **16**

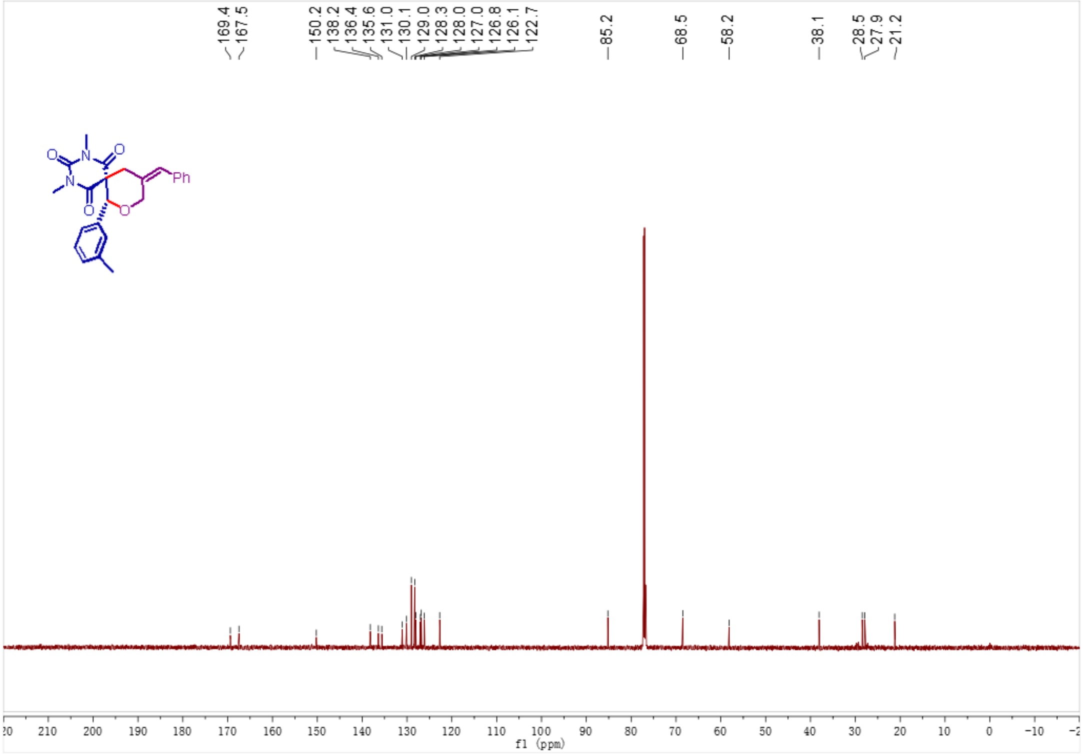

^{13}C ($CDCl_3$, 151 MHz) NMR of compound **16**

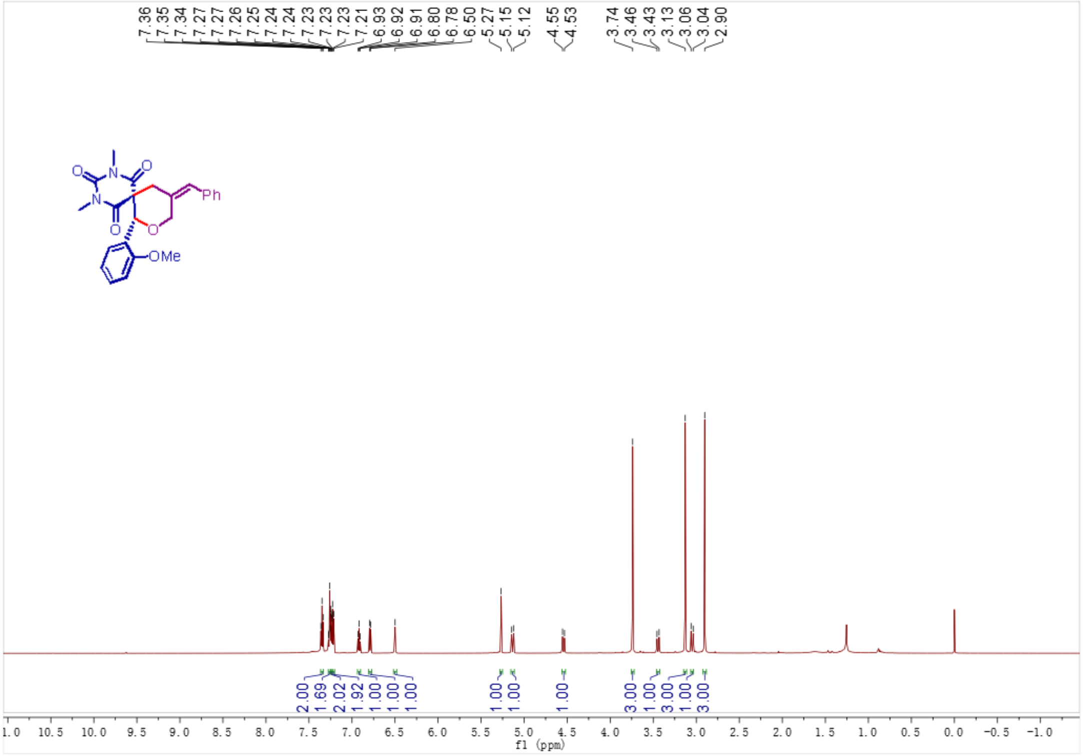

^{1}H ($CDCl_3$, 600 MHz) NMR of compound **17**

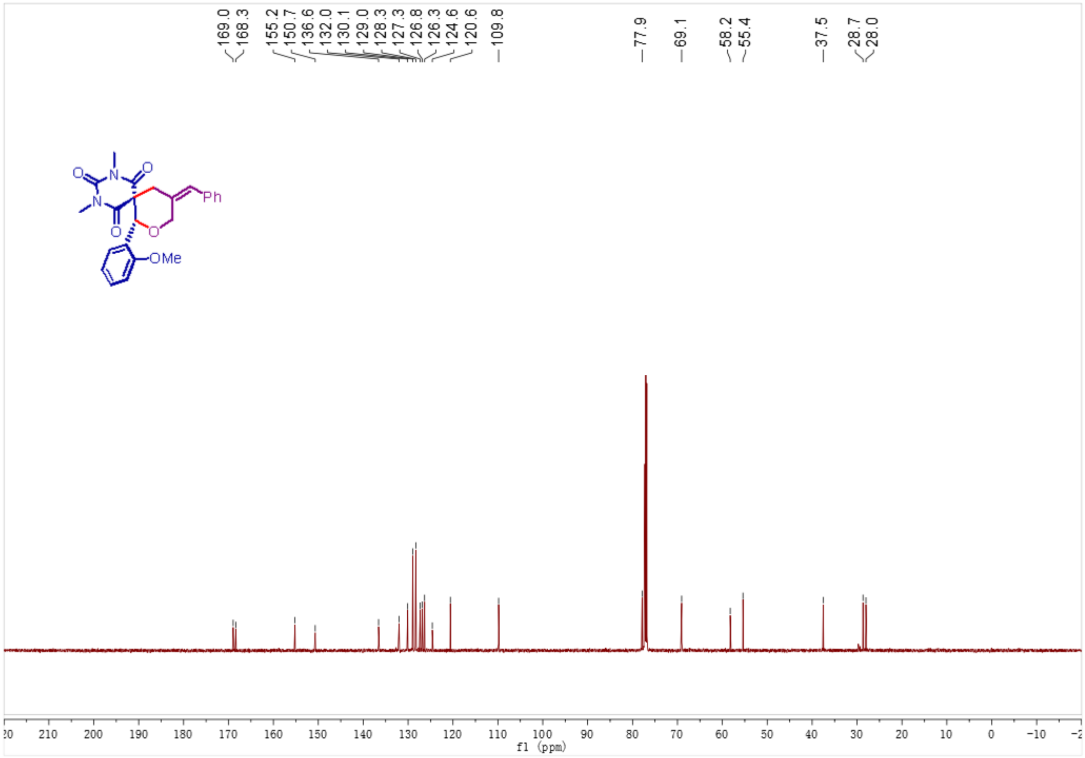

^{13}C (CDCl$_3$, 151 MHz) NMR of compound **17**

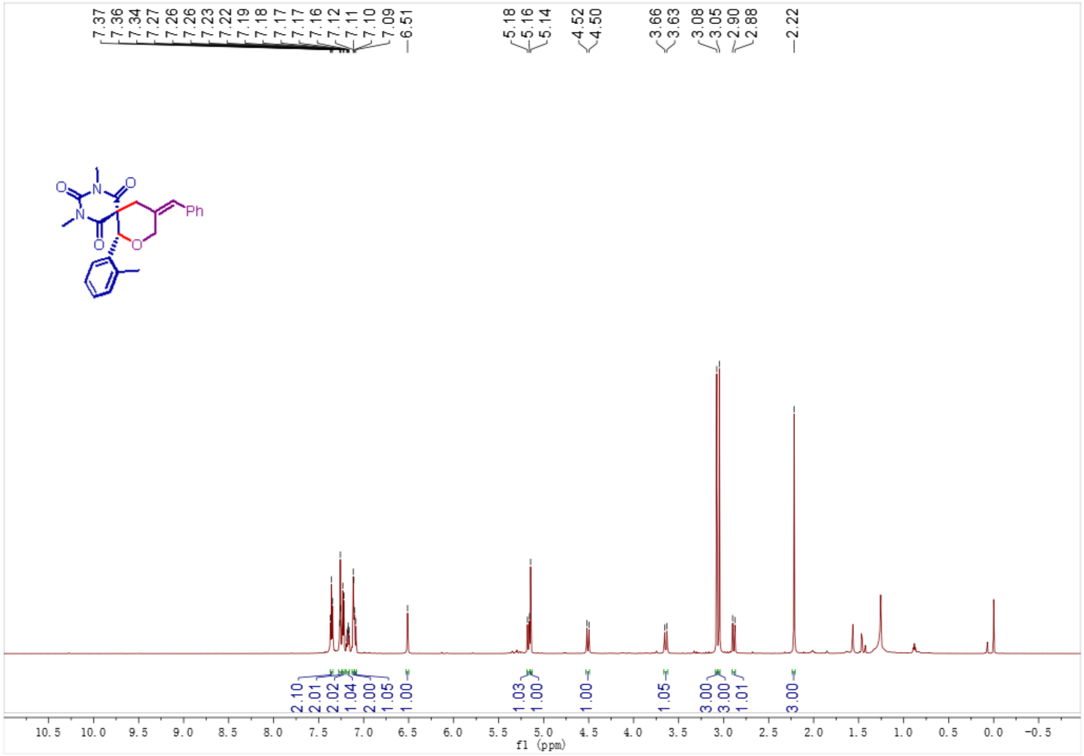

^{1}H (CDCl$_3$, 600 MHz) NMR of compound **18**

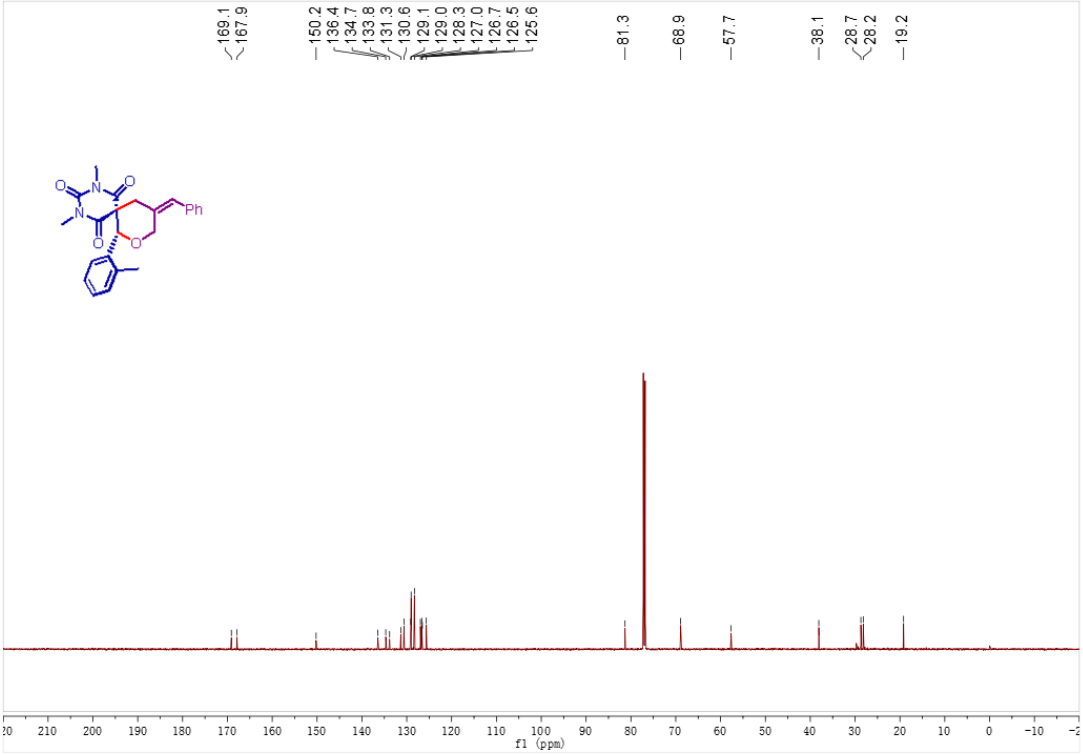

^{13}C ($CDCl_3$, 151 MHz) NMR of compound **18**

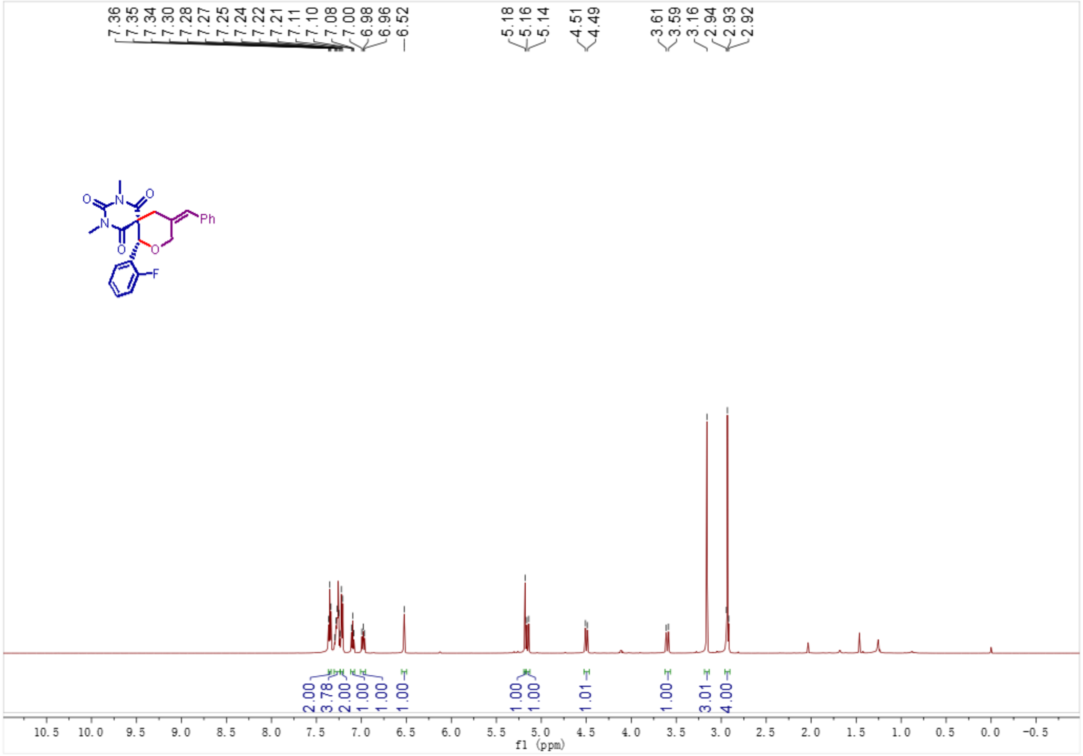

^{1}H ($CDCl_3$, 600 MHz) NMR of compound **19**

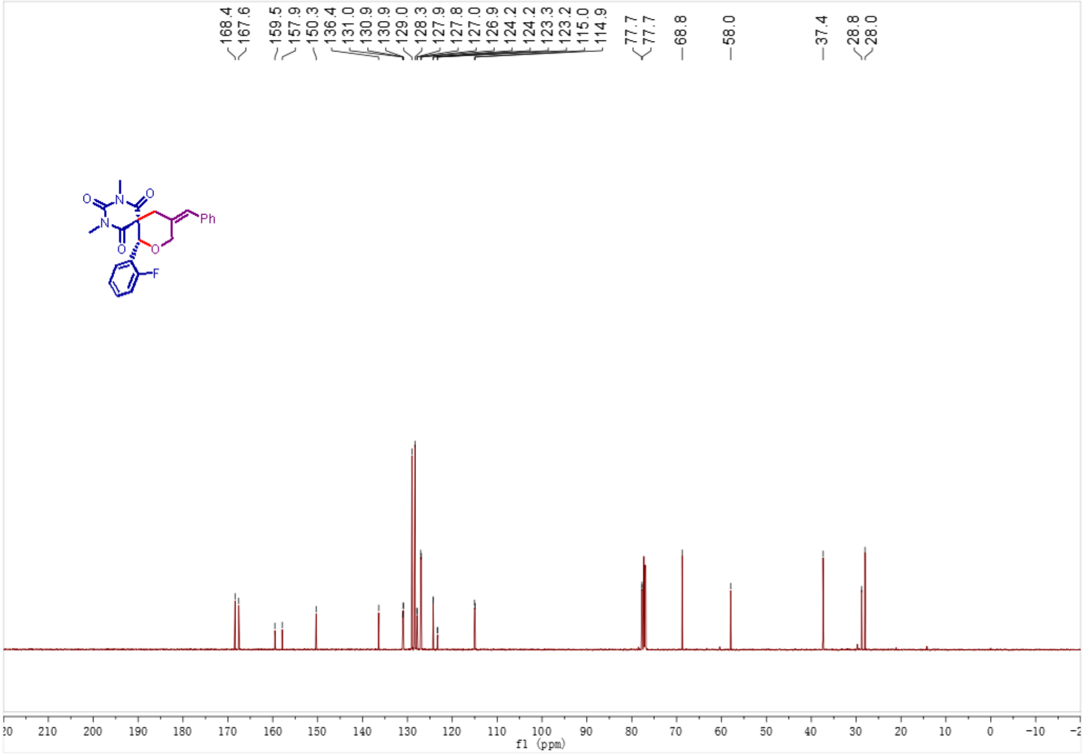

^{13}C ($CDCl_3$, 151 MHz) NMR of compound **19**

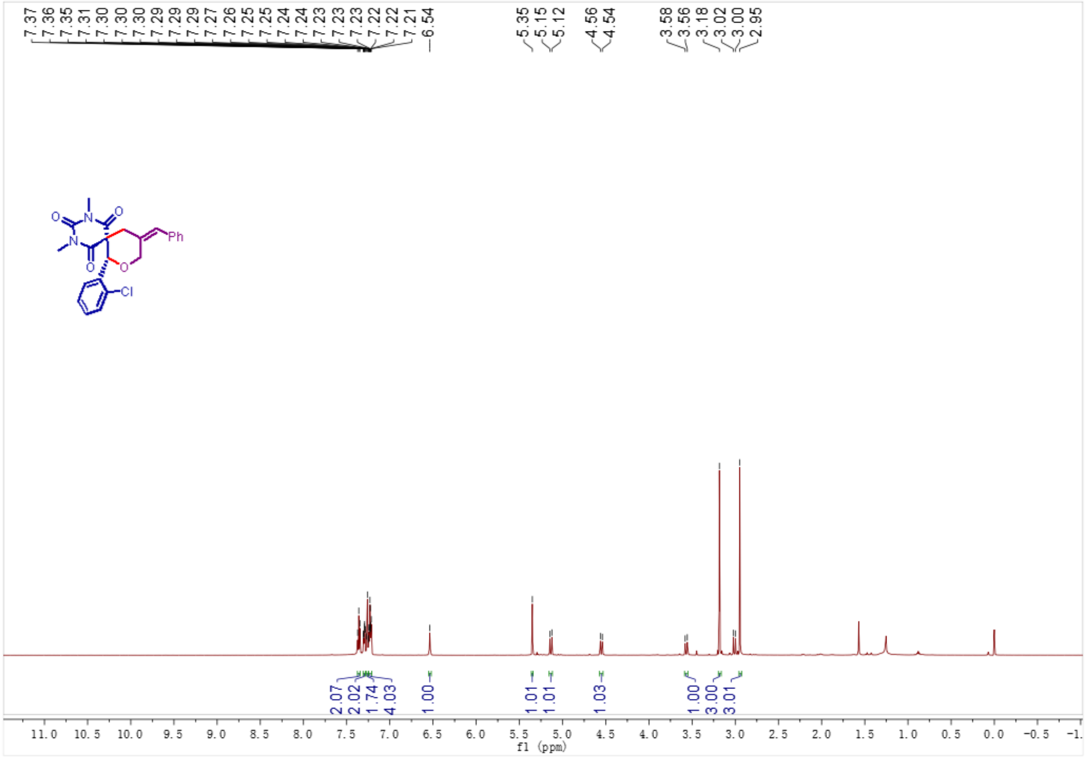

^{1}H ($CDCl_3$, 600 MHz) NMR of compound **20**

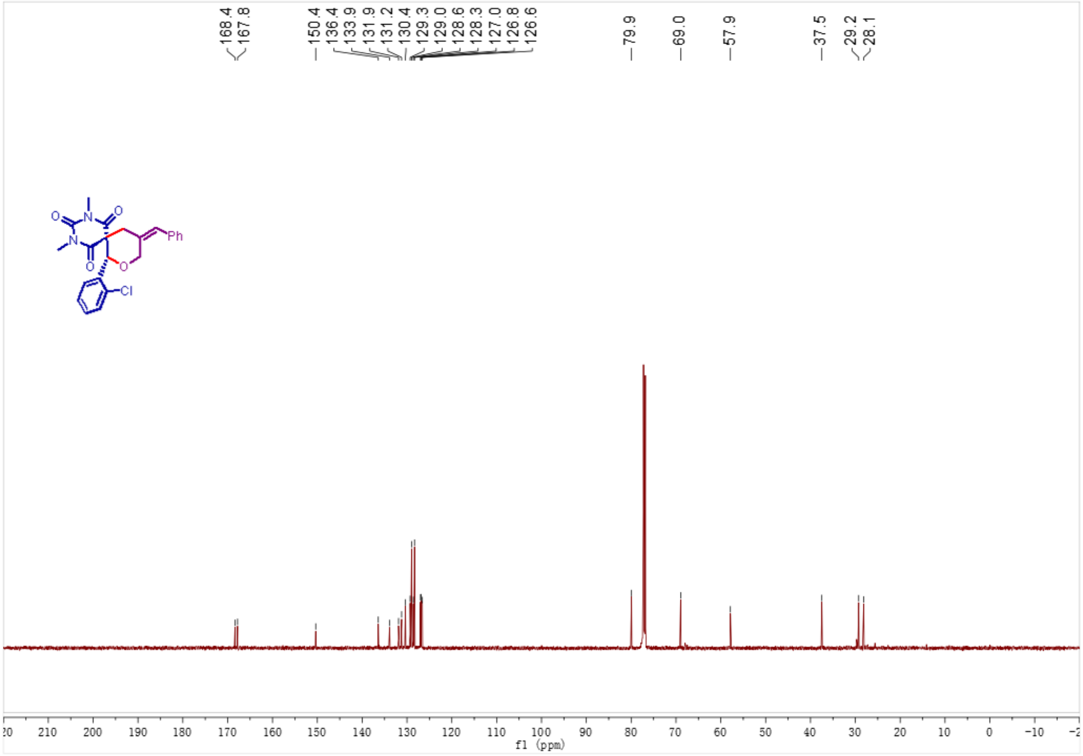

^{13}C ($CDCl_3$, 151 MHz) NMR of compound **20**

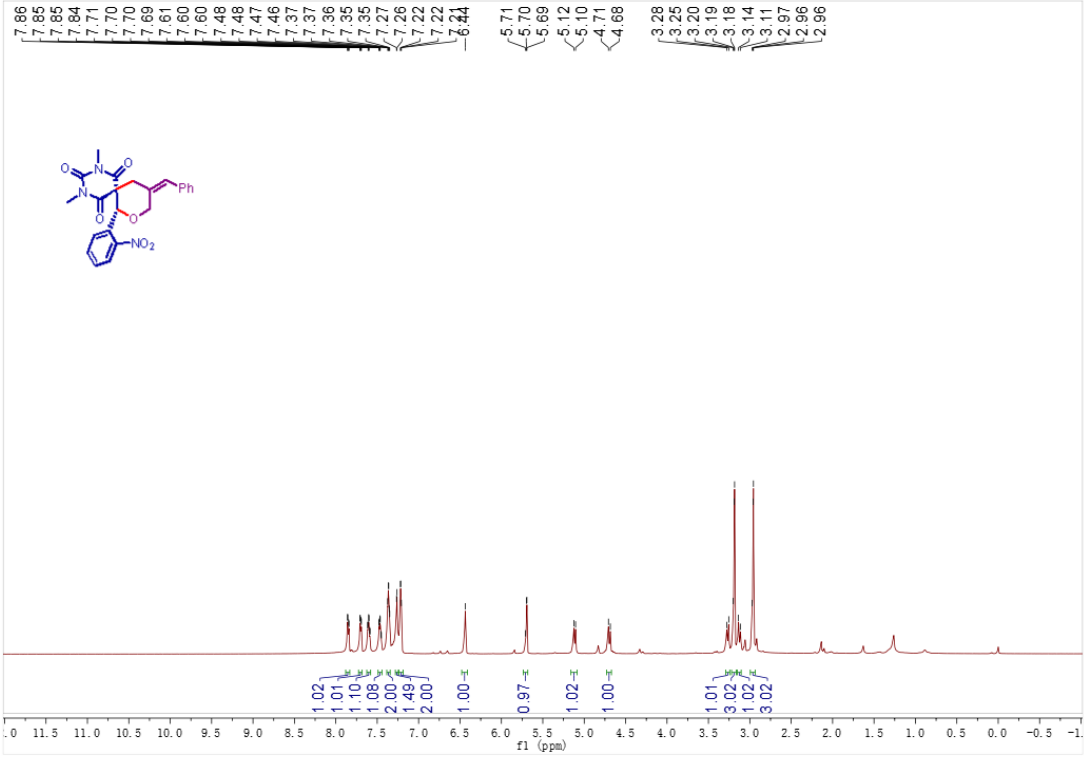

^{1}H ($CDCl_3$, 600 MHz) NMR of compound **21**

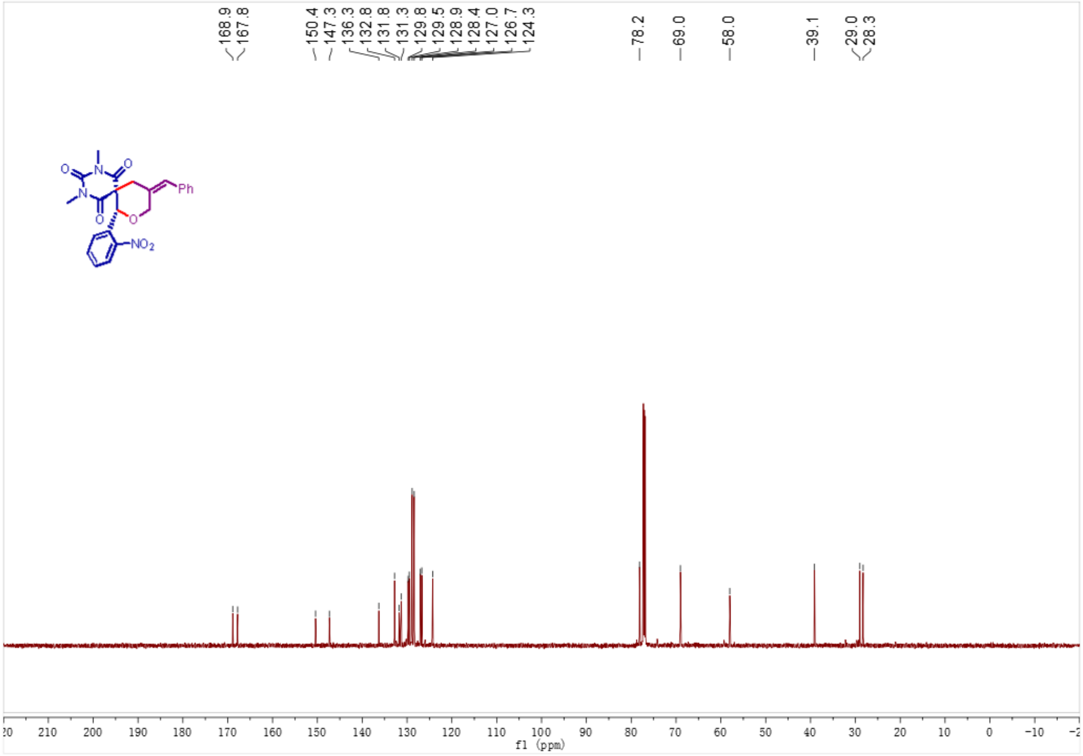

^{13}C ($CDCl_3$, 151 MHz) NMR of compound **21**

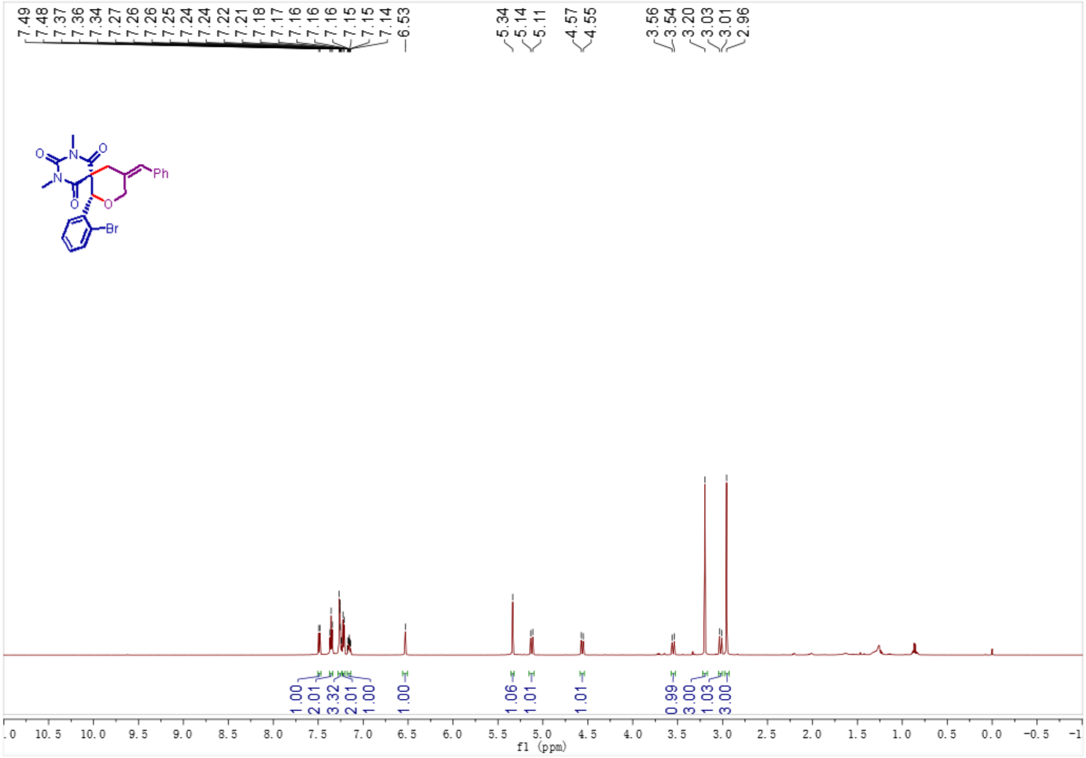

^{1}H ($CDCl_3$, 600 MHz) NMR of compound **22**

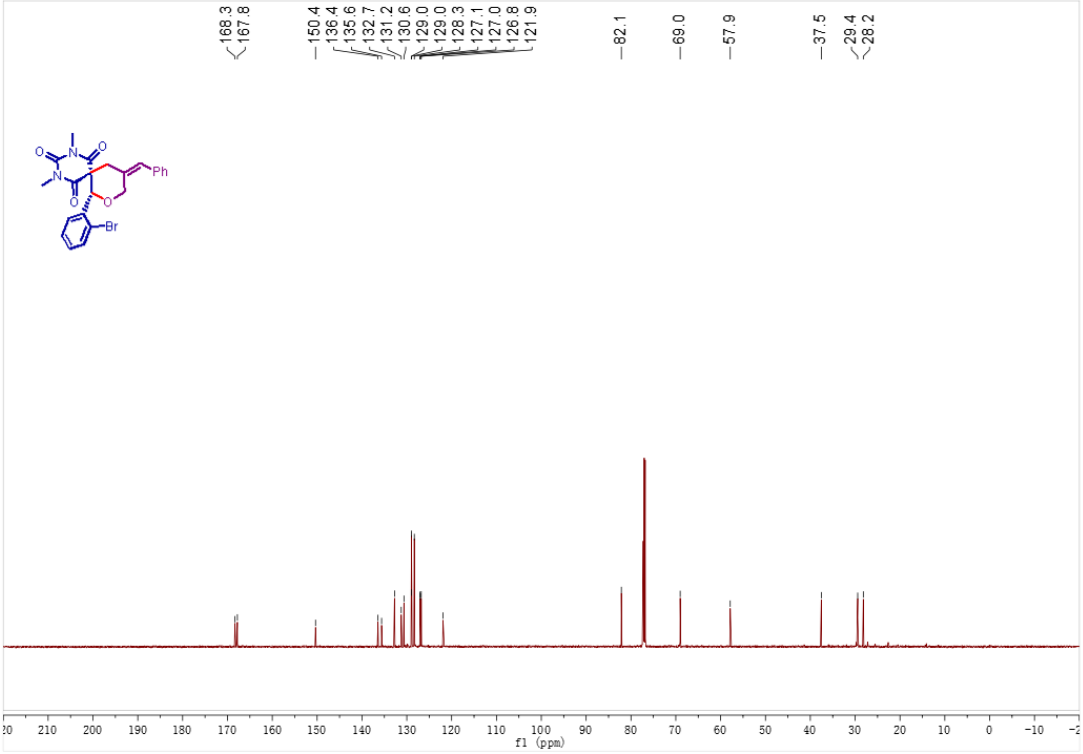

^{13}C ($CDCl_3$, 151 MHz) NMR of compound **22**

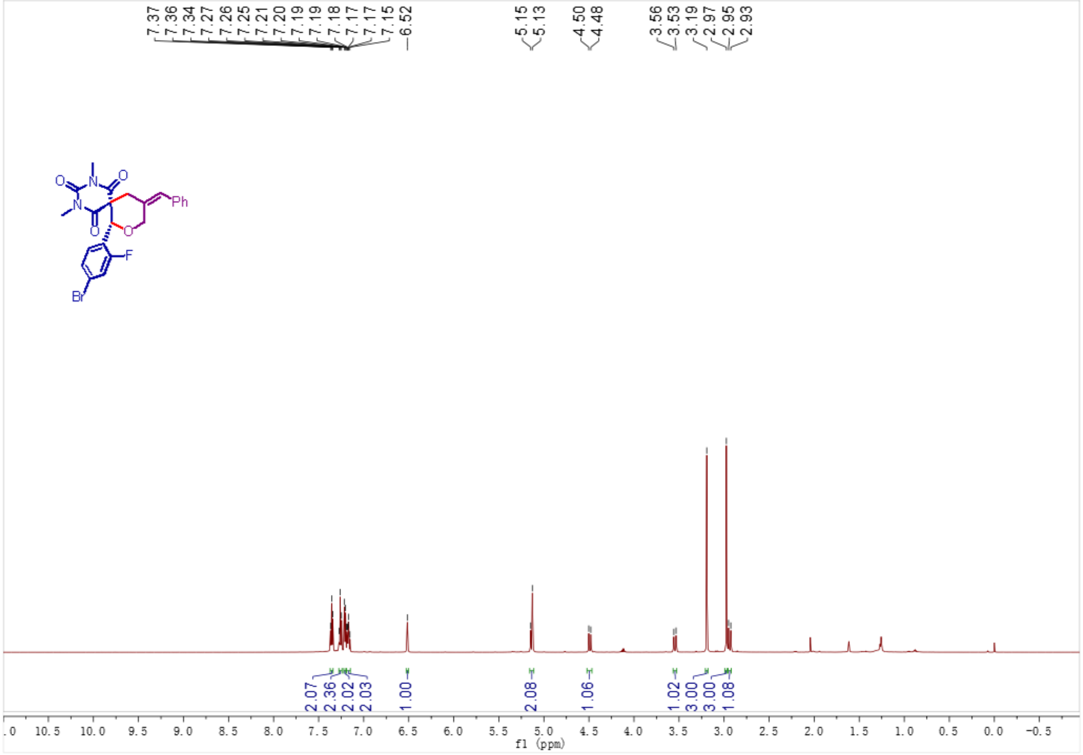

^{1}H ($CDCl_3$, 600 MHz) NMR of compound **23**

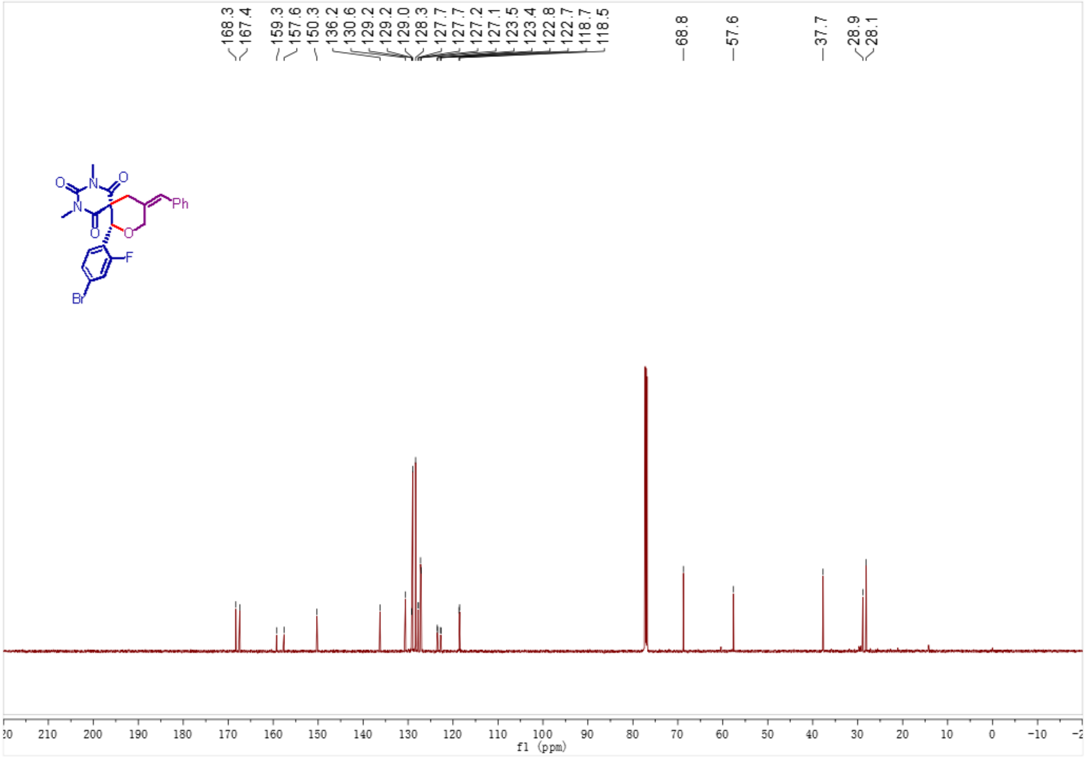

^{13}C (CDCl$_3$, 151 MHz) NMR of compound **23**

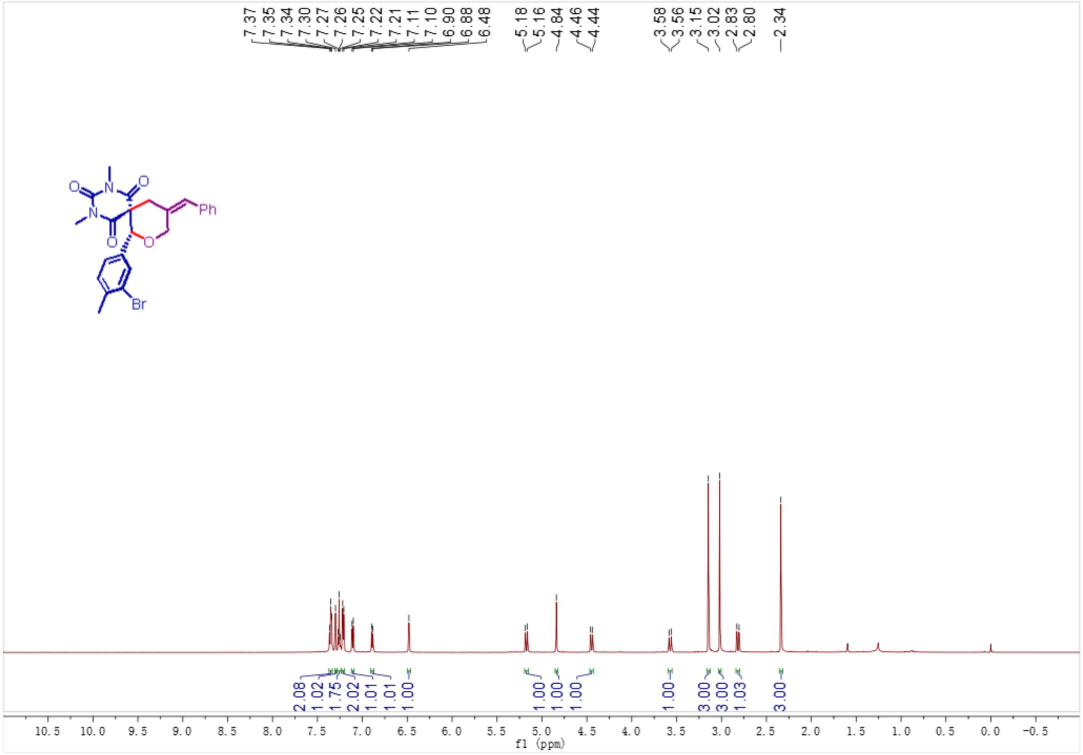

^{1}H (CDCl$_3$, 600 MHz) NMR of compound **24**

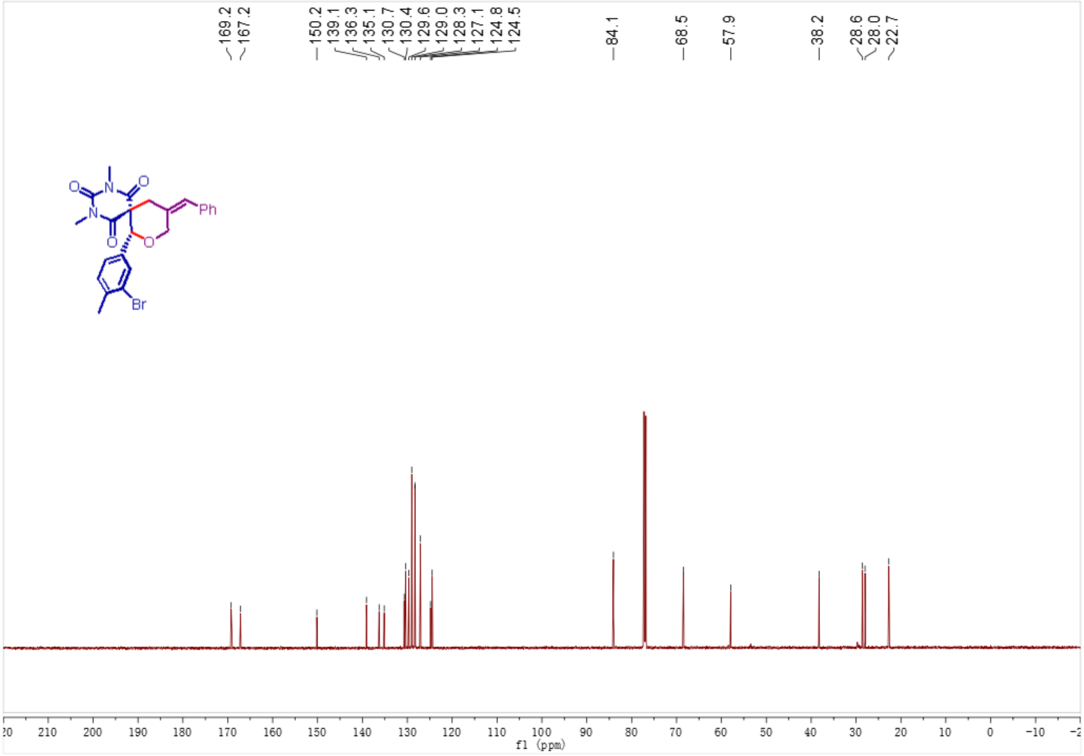

^{13}C ($CDCl_3$, 151 MHz) NMR of compound **24**

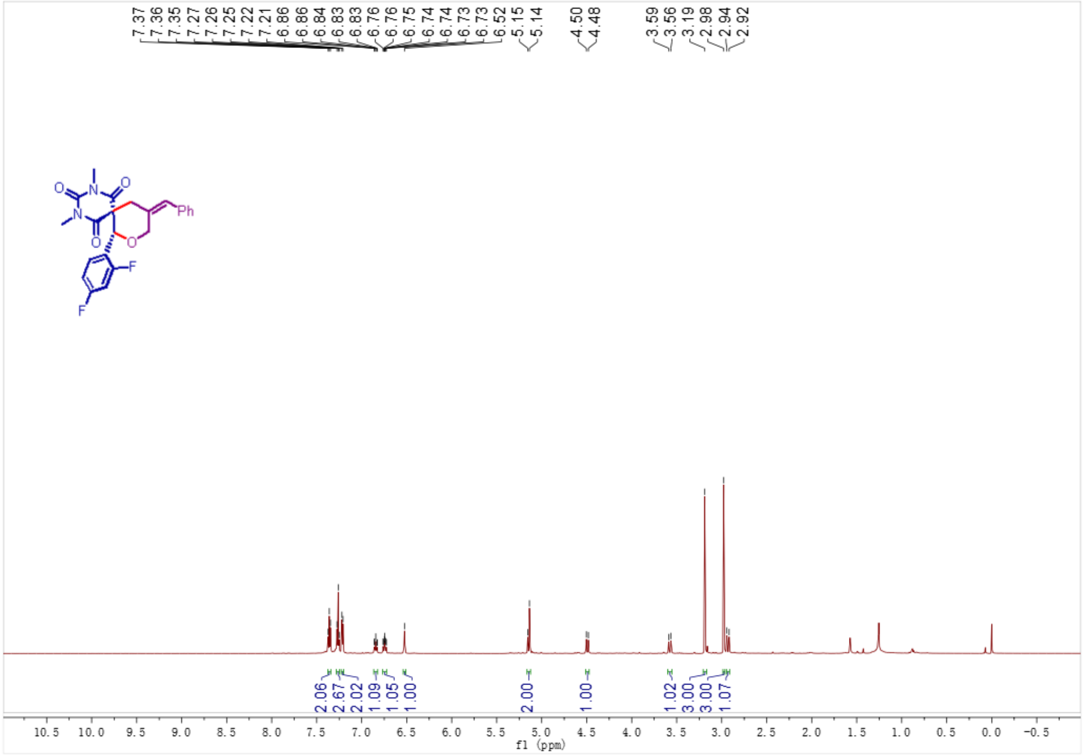

^{1}H ($CDCl_3$, 600 MHz) NMR of compound **25**

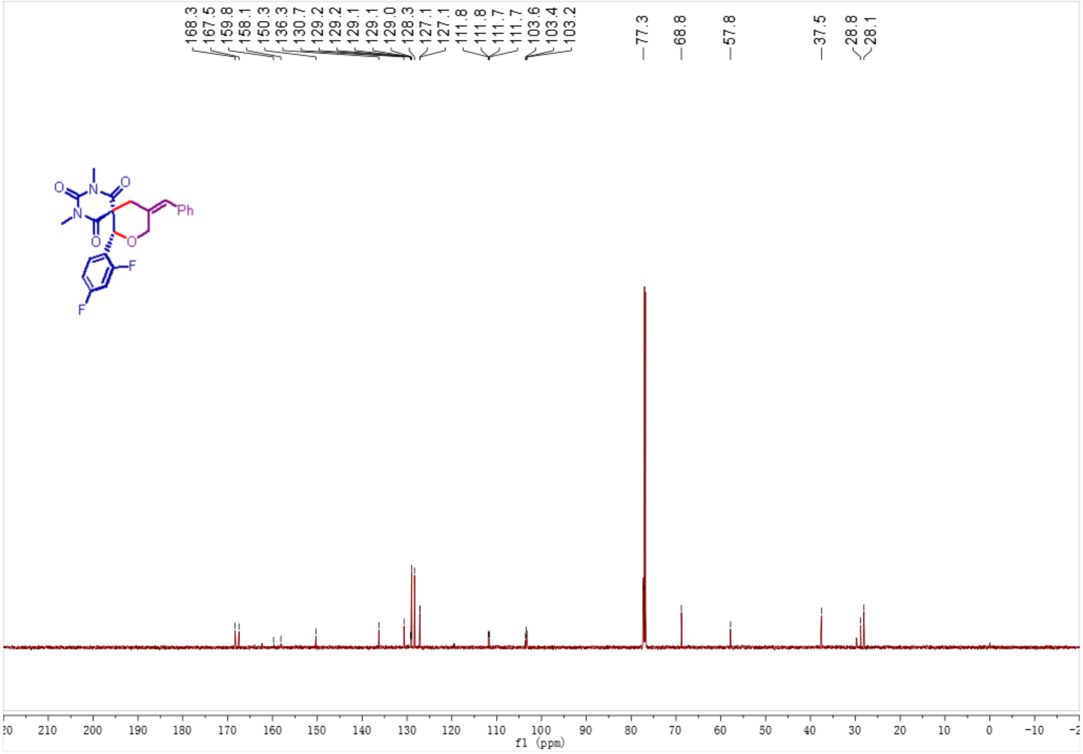

^{13}C ($CDCl_3$, 151 MHz) NMR of compound **25**

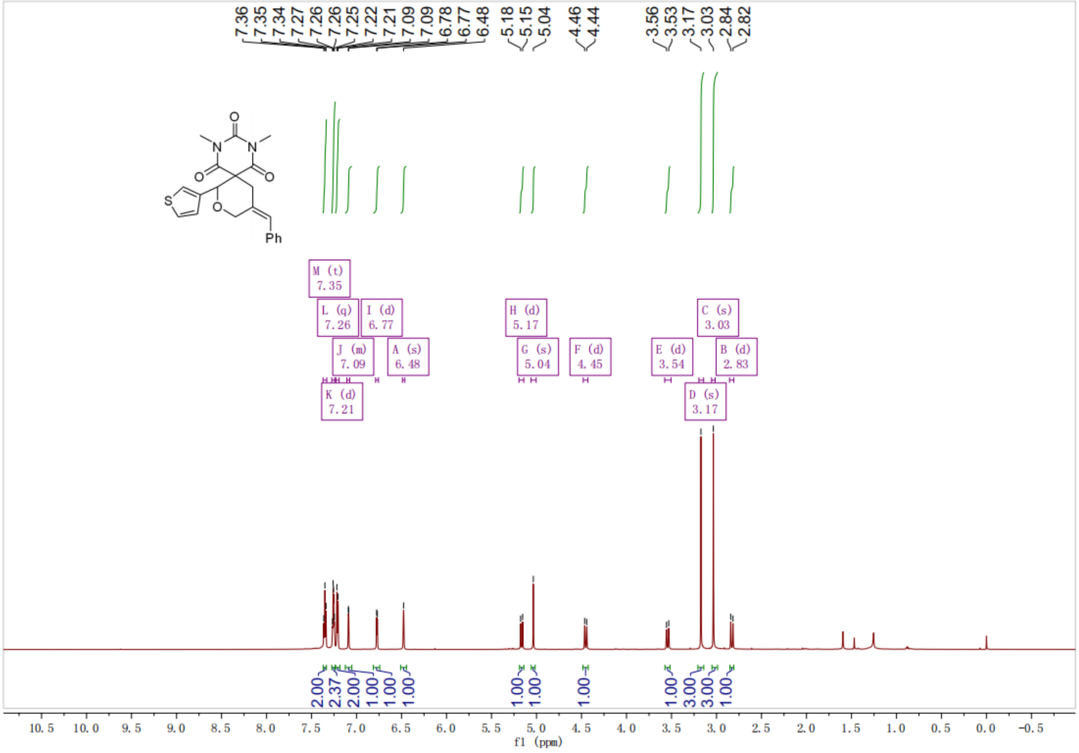

^{1}H ($CDCl_3$, 600 MHz) NMR of compound **26**

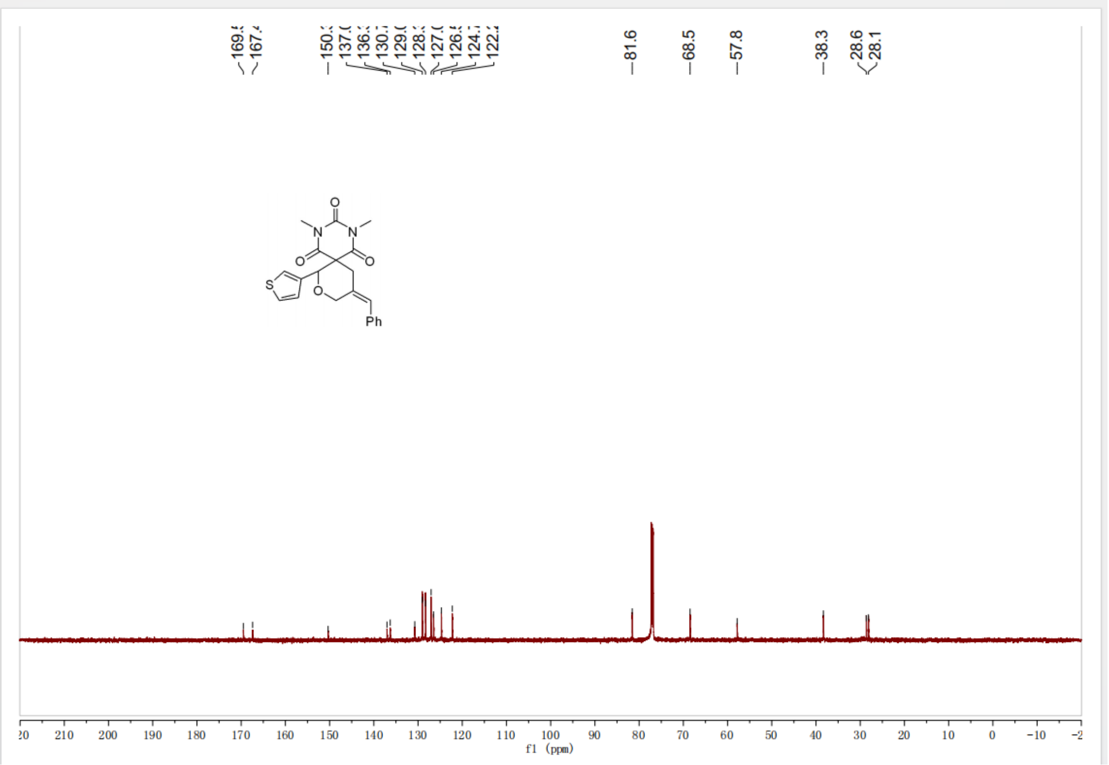

^{13}C ($CDCl_3$, 151 MHz) NMR of compound **26**

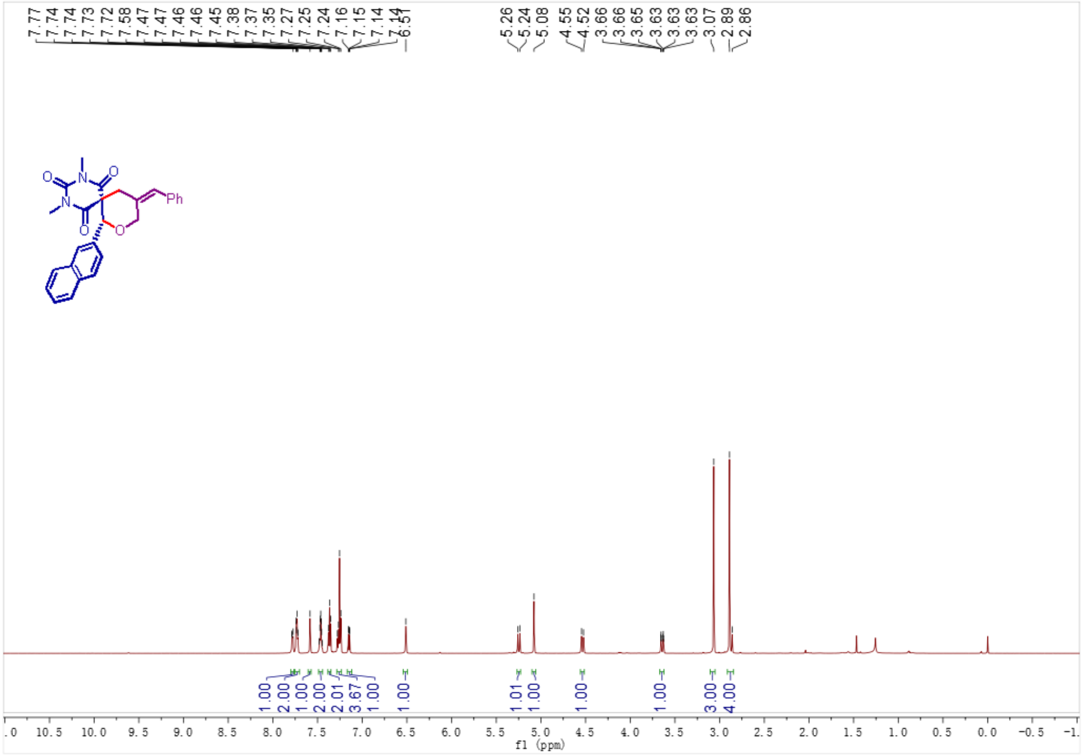

^{1}H ($CDCl_3$, 600 MHz) NMR of compound **27**

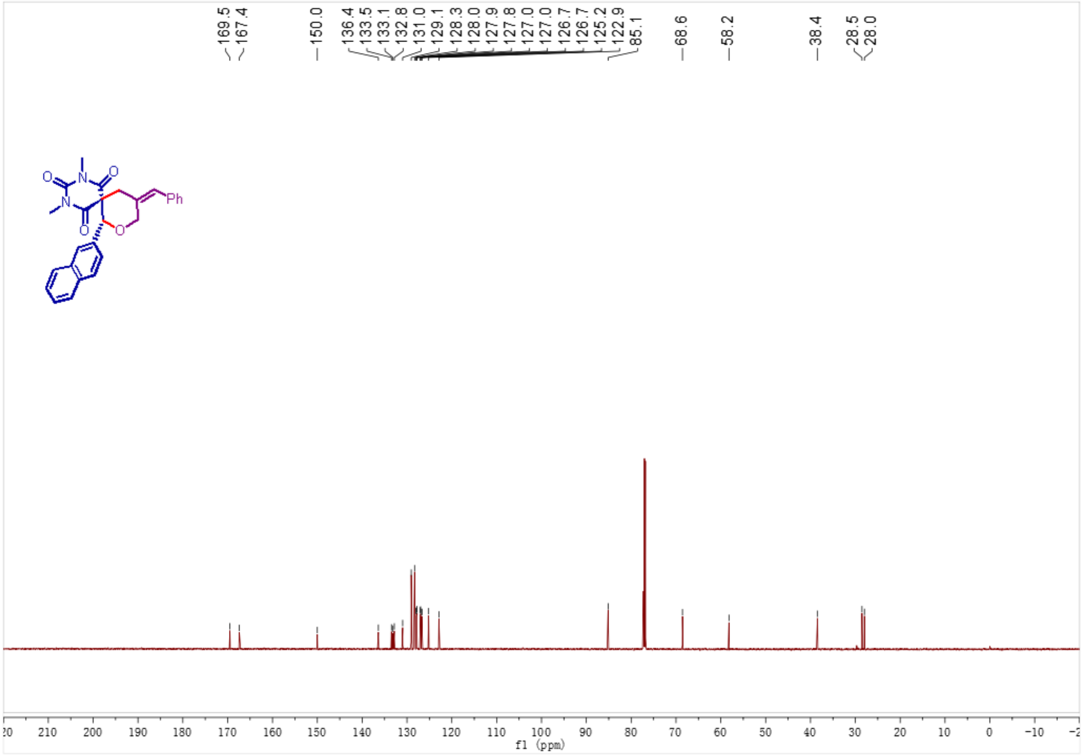

^{13}C ($CDCl_3$, 151 MHz) NMR of compound **27**

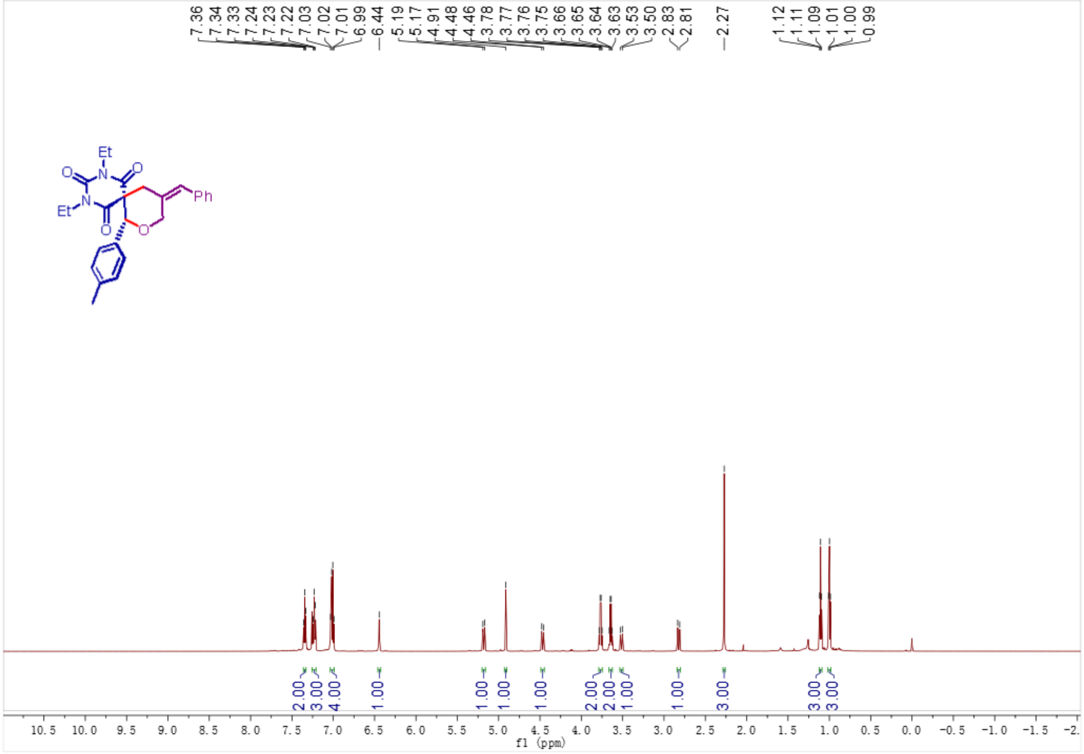

^{1}H ($CDCl_3$, 600 MHz) NMR of compound **28**

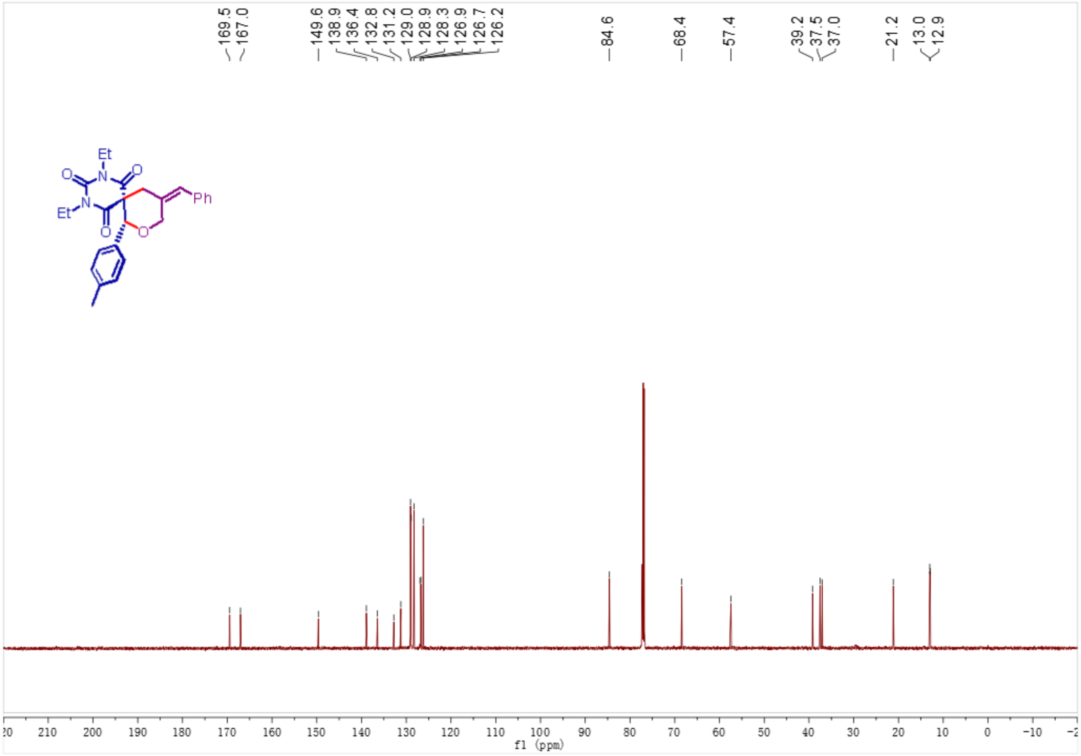

^{13}C (CDCl$_3$, 151 MHz) NMR of compound **28**

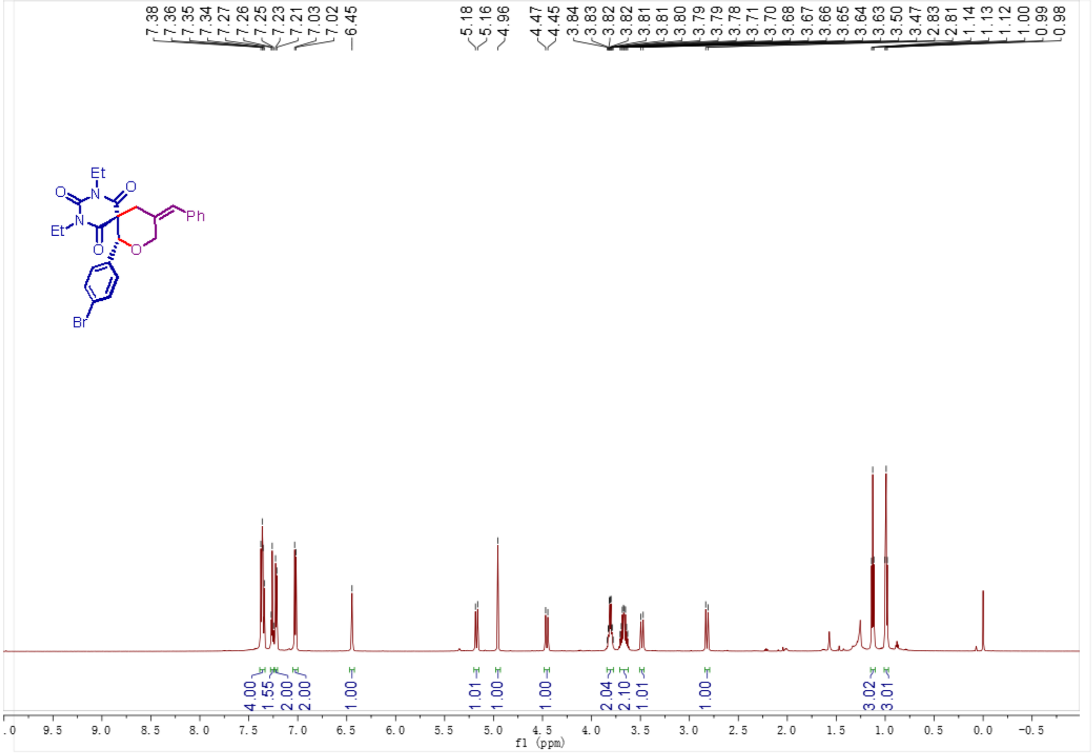

^{1}H (CDCl$_3$, 600 MHz) NMR of compound **29**

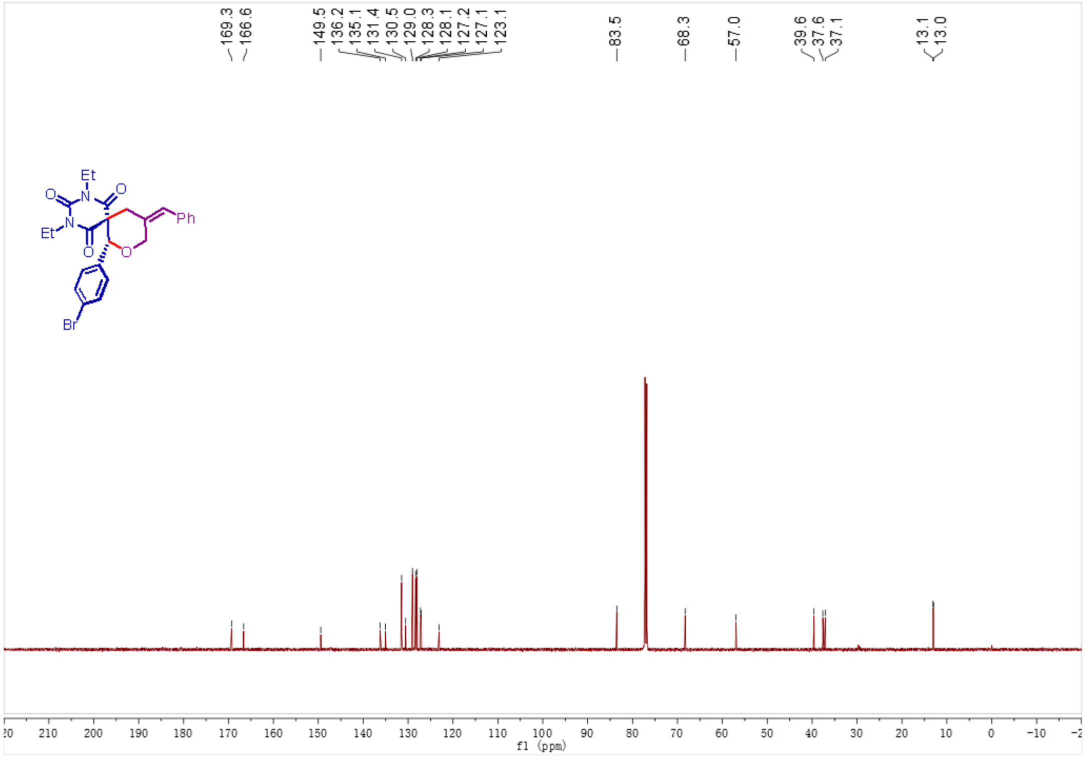

^{13}C (CDCl$_3$, 151 MHz) NMR of compound **29**

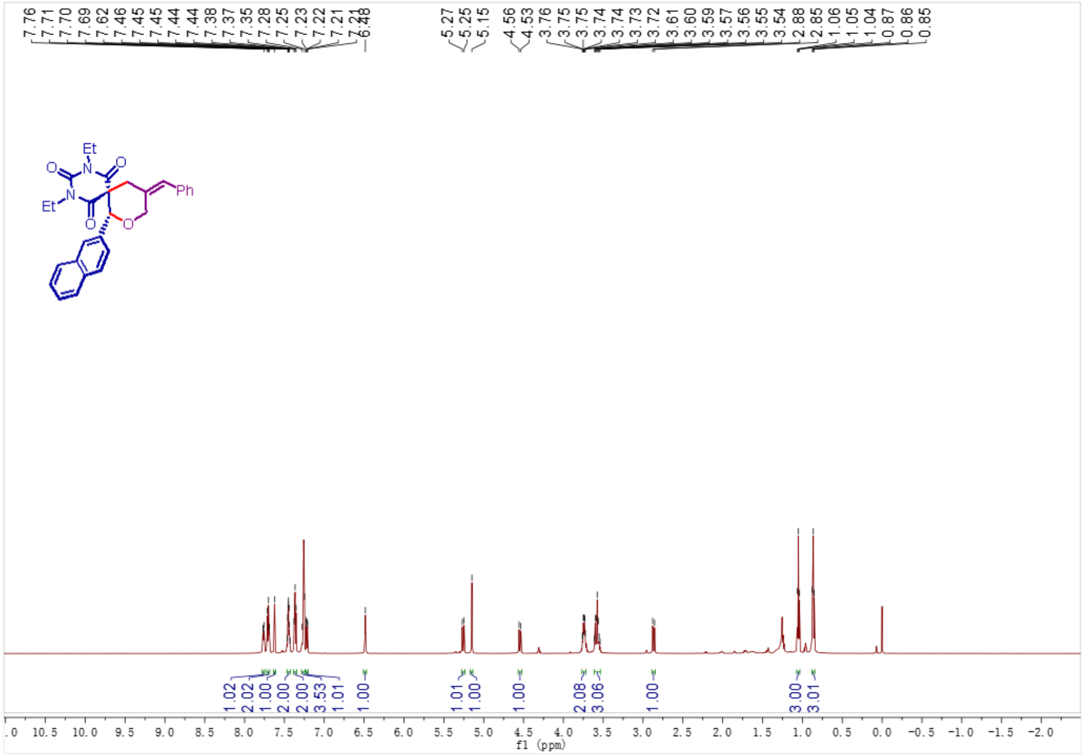

^{1}H (CDCl$_3$, 600 MHz) NMR of compound **30**

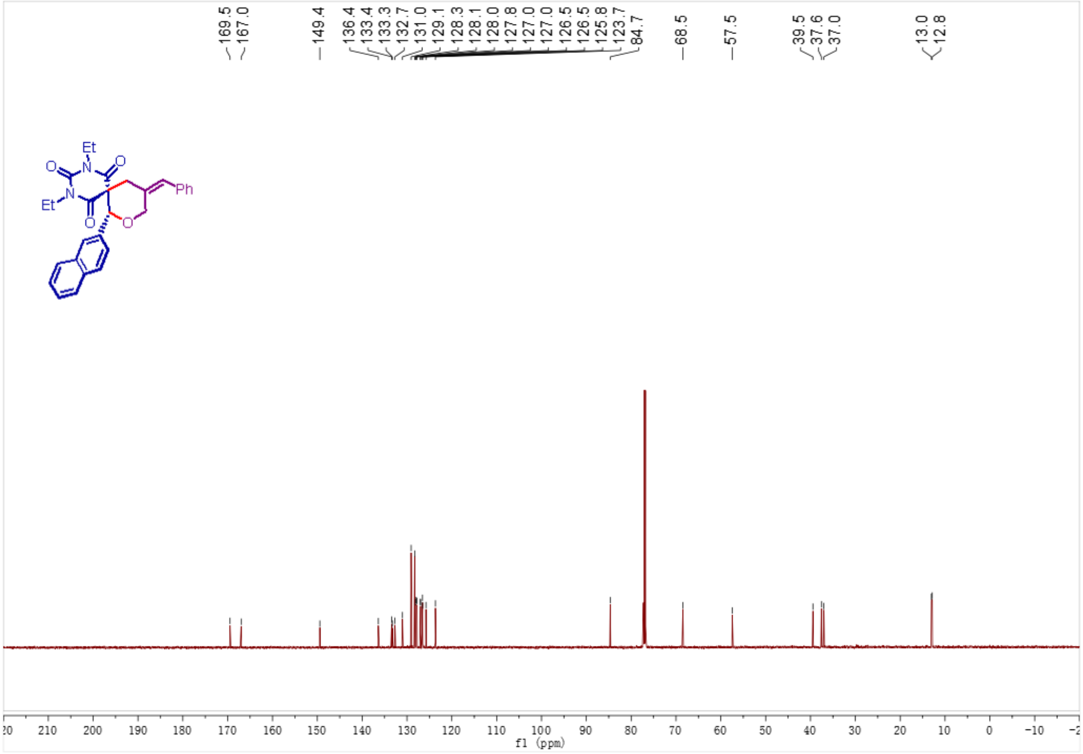

^{13}C ($CDCl_3$, 151 MHz) NMR of compound **30**

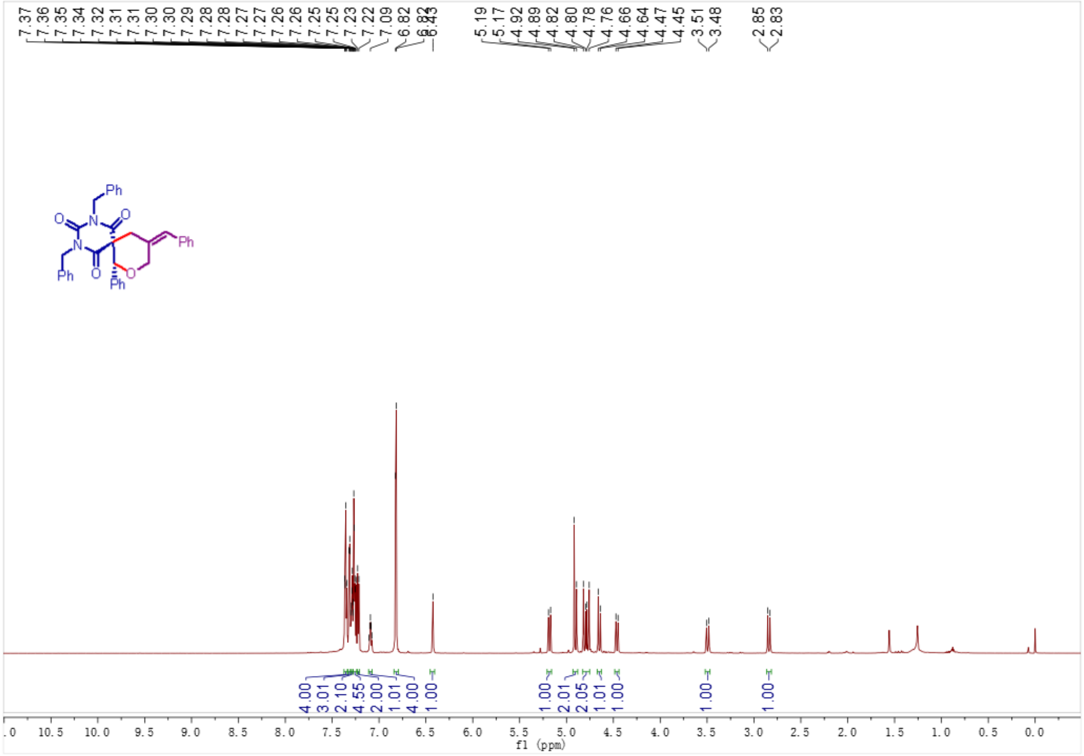

^{1}H ($CDCl_3$, 600 MHz) NMR of compound **31**

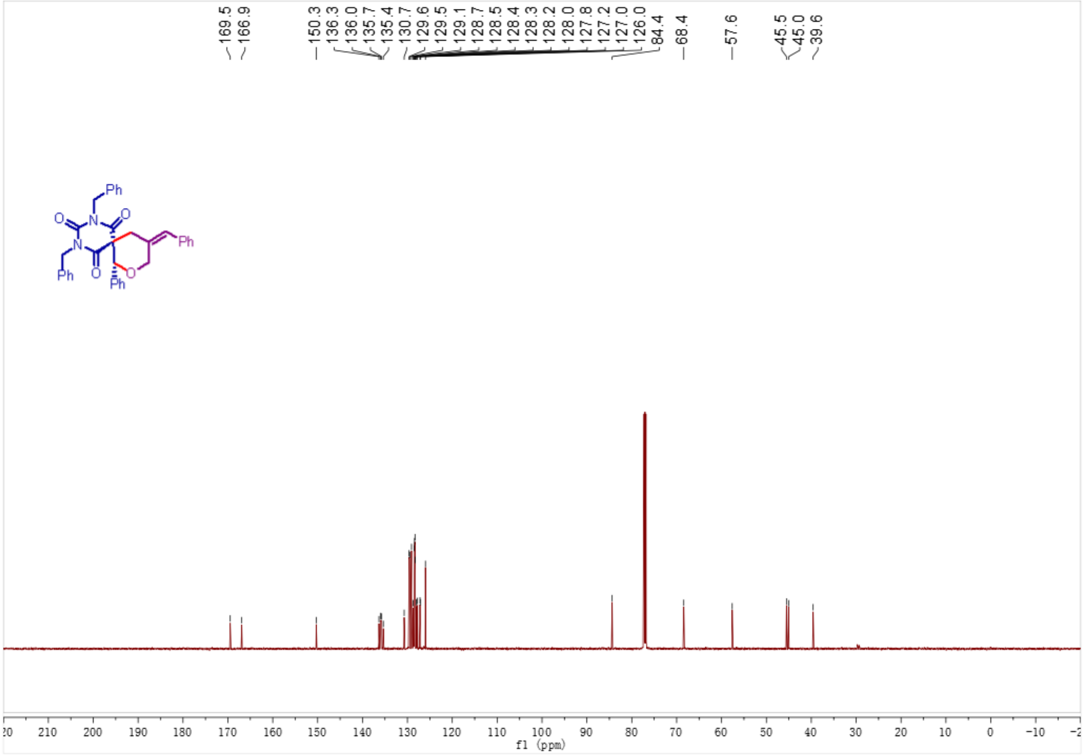

^{13}C ($CDCl_3$, 151 MHz) NMR of compound **31**

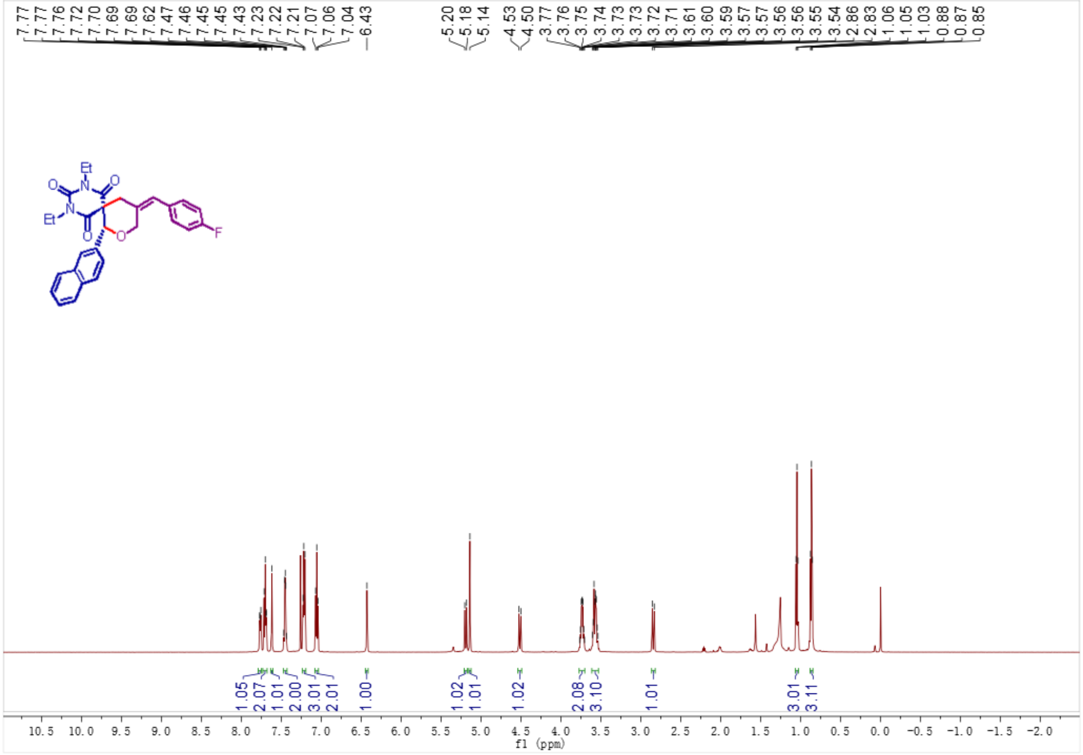

^{1}H ($CDCl_3$, 600 MHz) NMR of compound **32**

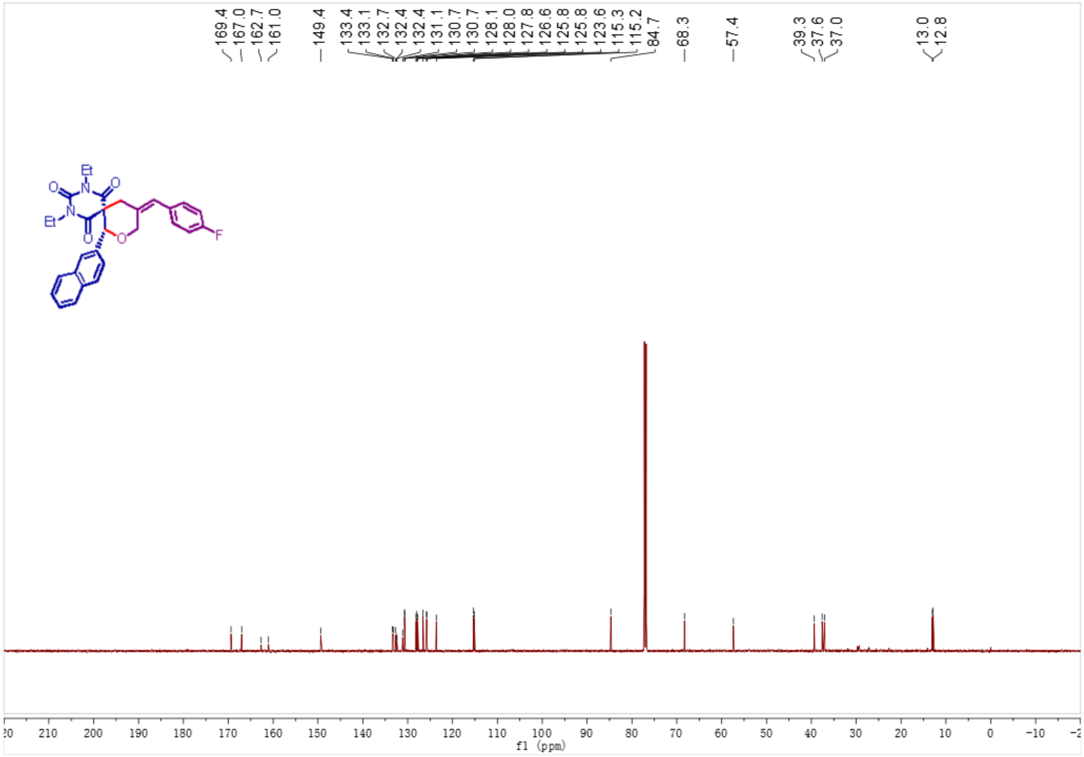

^{13}C ($CDCl_3$, 151 MHz) NMR of compound **32**

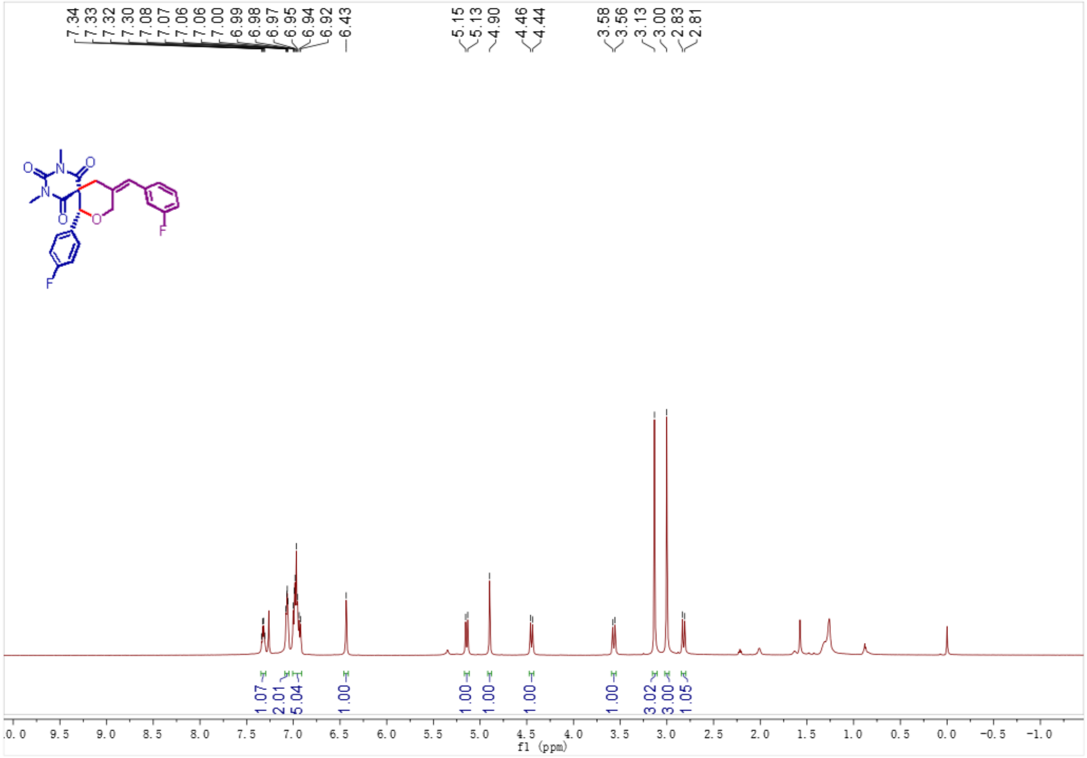

^{1}H ($CDCl_3$, 600 MHz) NMR of compound **33**

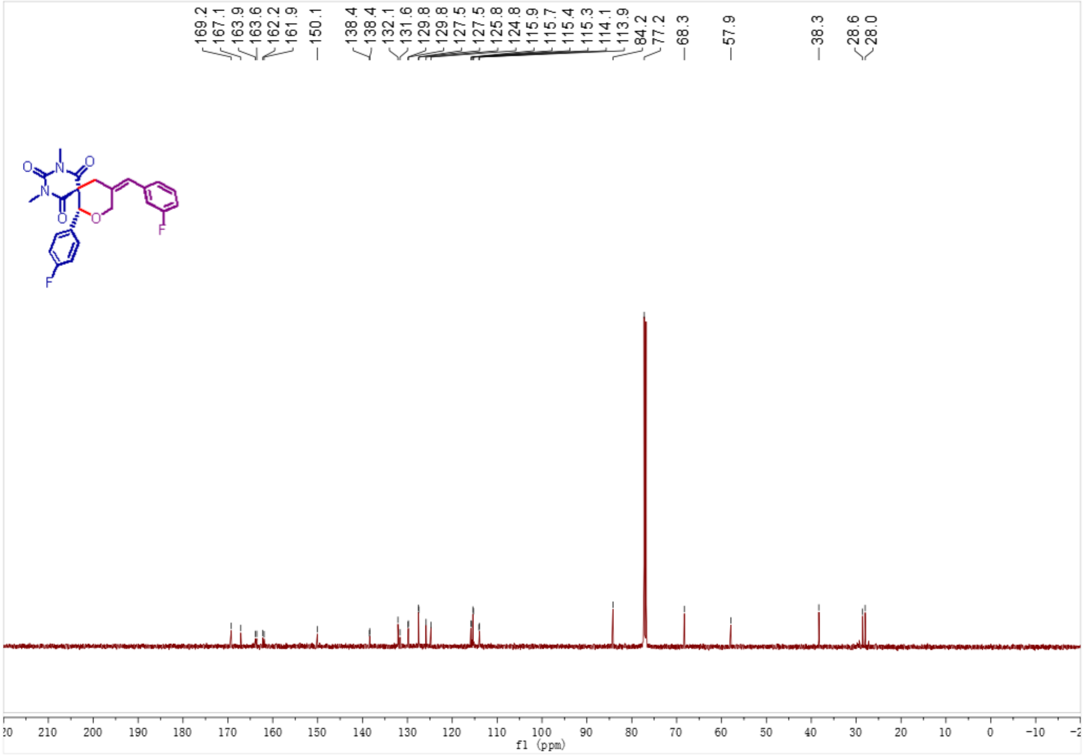

^{13}C ($CDCl_3$, 151 MHz) NMR of compound **33**

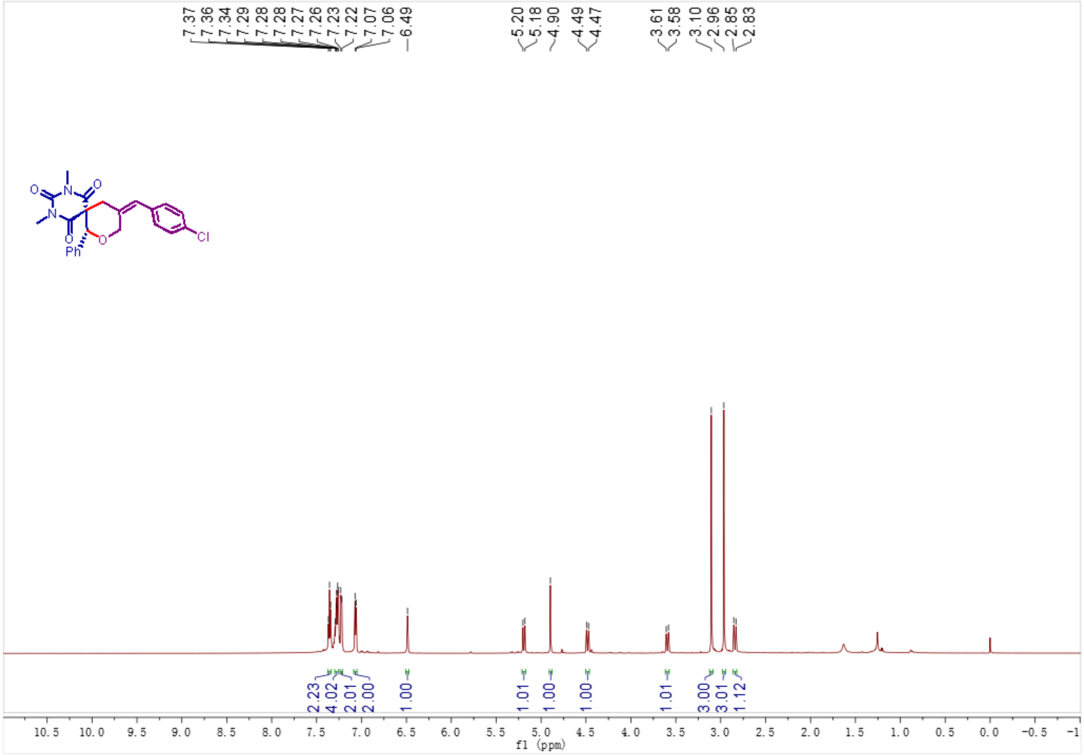

^{1}H ($CDCl_3$, 600 MHz) NMR of compound **34**

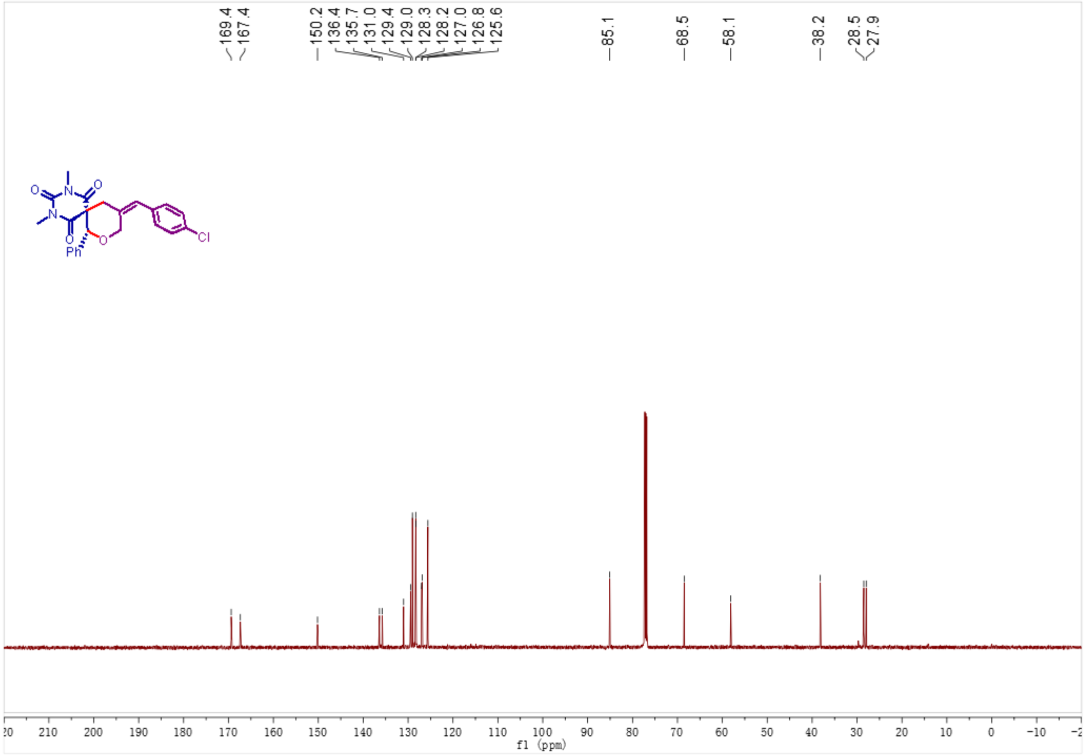

^{13}C ($CDCl_3$, 151 MHz) NMR of compound **34**

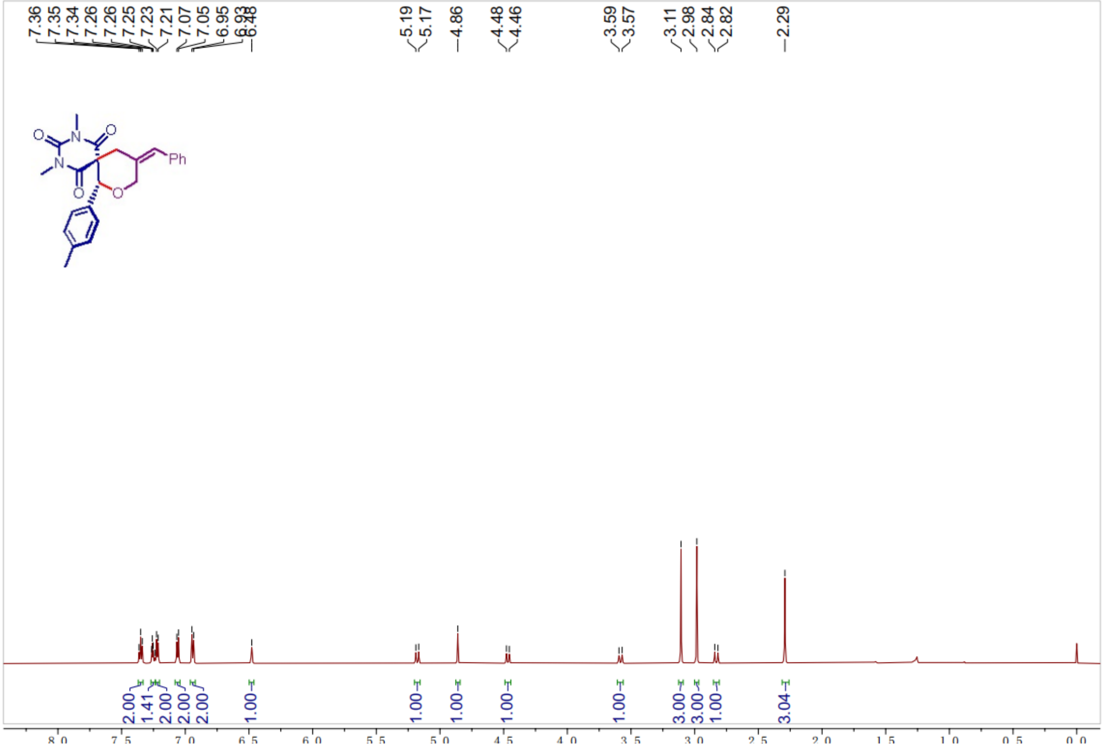

^{1}H ($CDCl_3$, 600 MHz) NMR of compound **35**

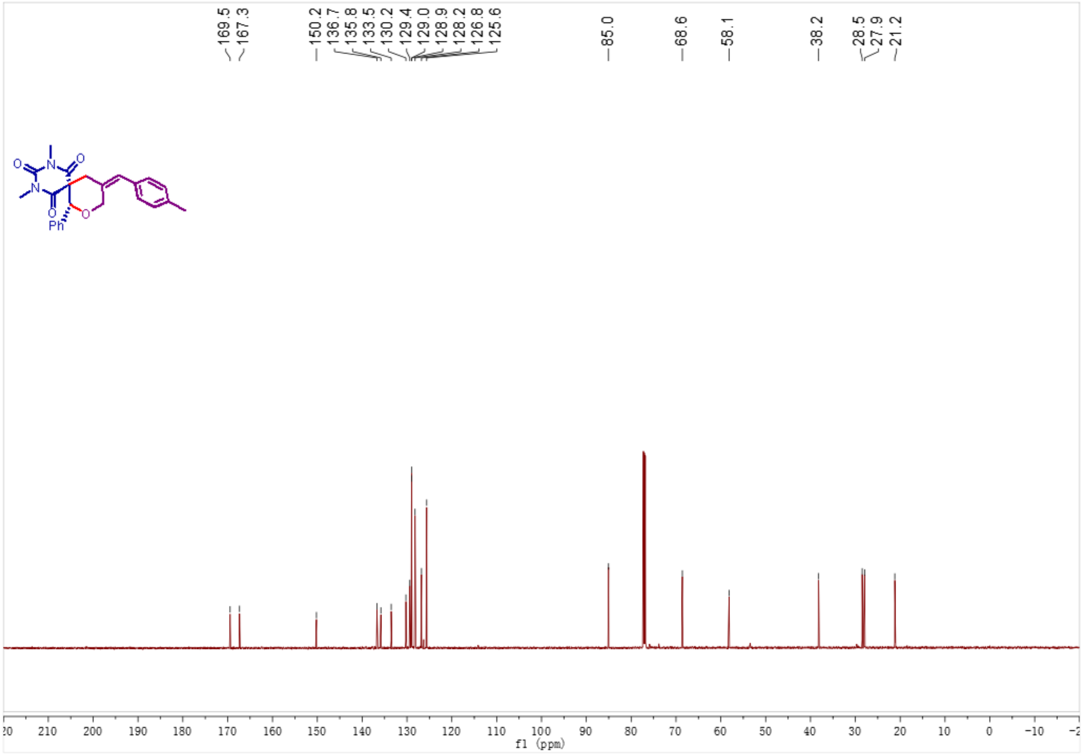

^{13}C (CDCl$_3$, 151 MHz) NMR of compound **35**

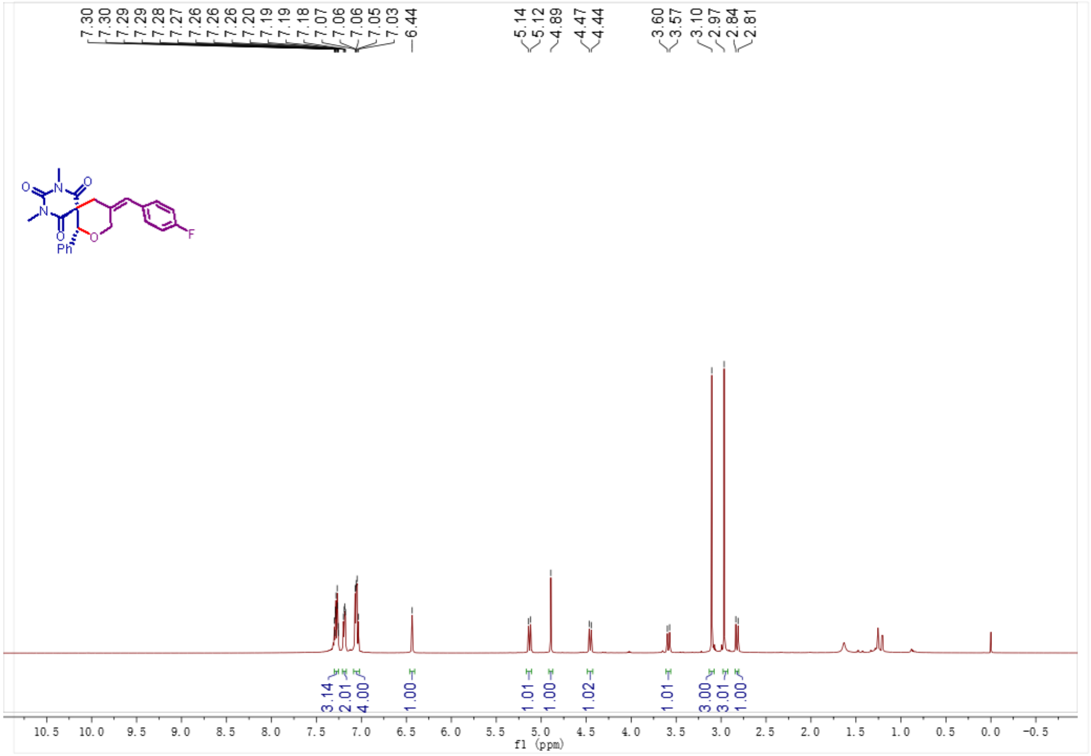

^{1}H (CDCl$_3$, 600 MHz) NMR of compound **36**

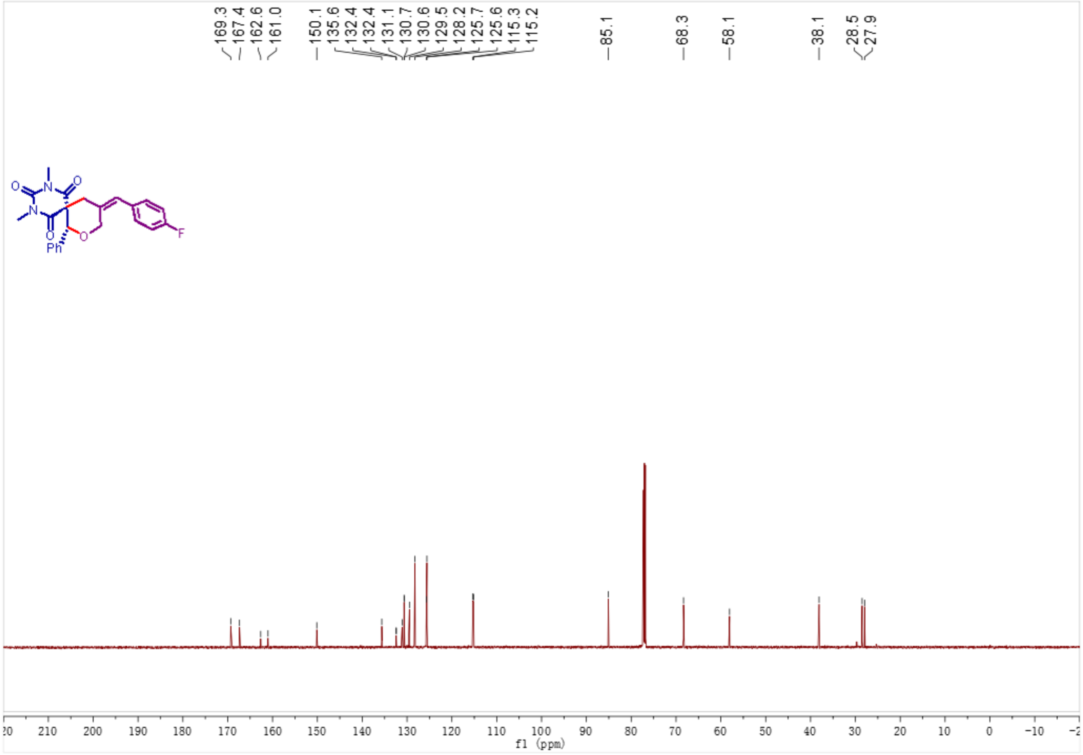

^{13}C ($CDCl_3$, 151 MHz) NMR of compound **36**

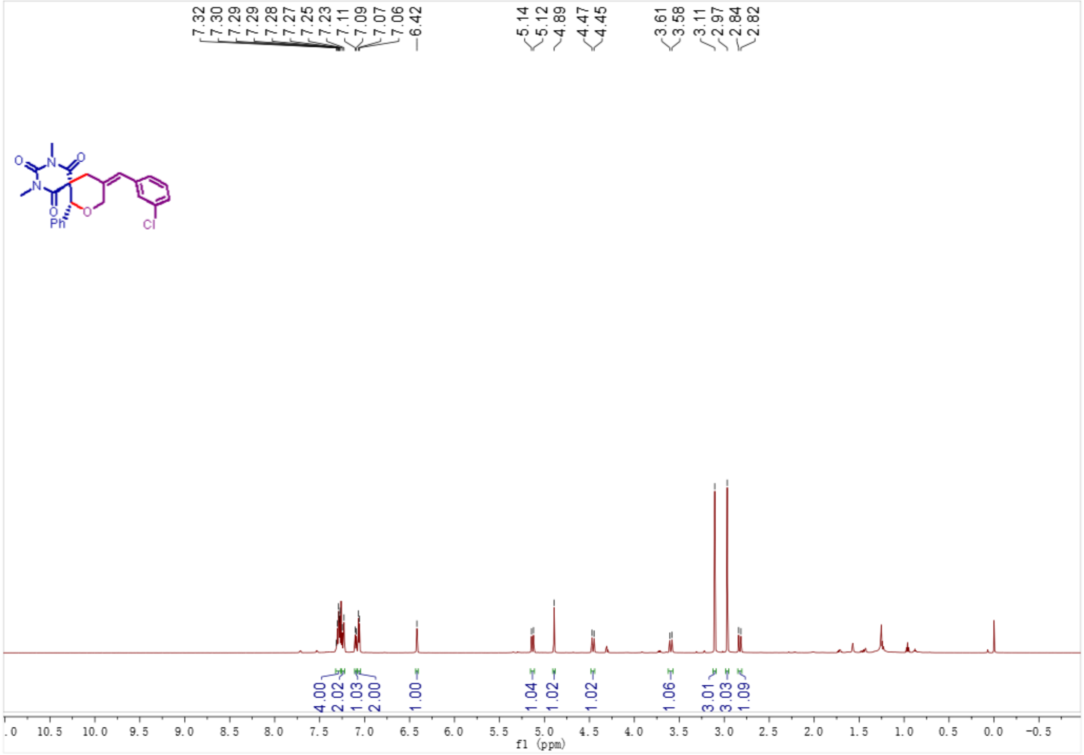

^{1}H ($CDCl_3$, 600 MHz) NMR of compound **37**

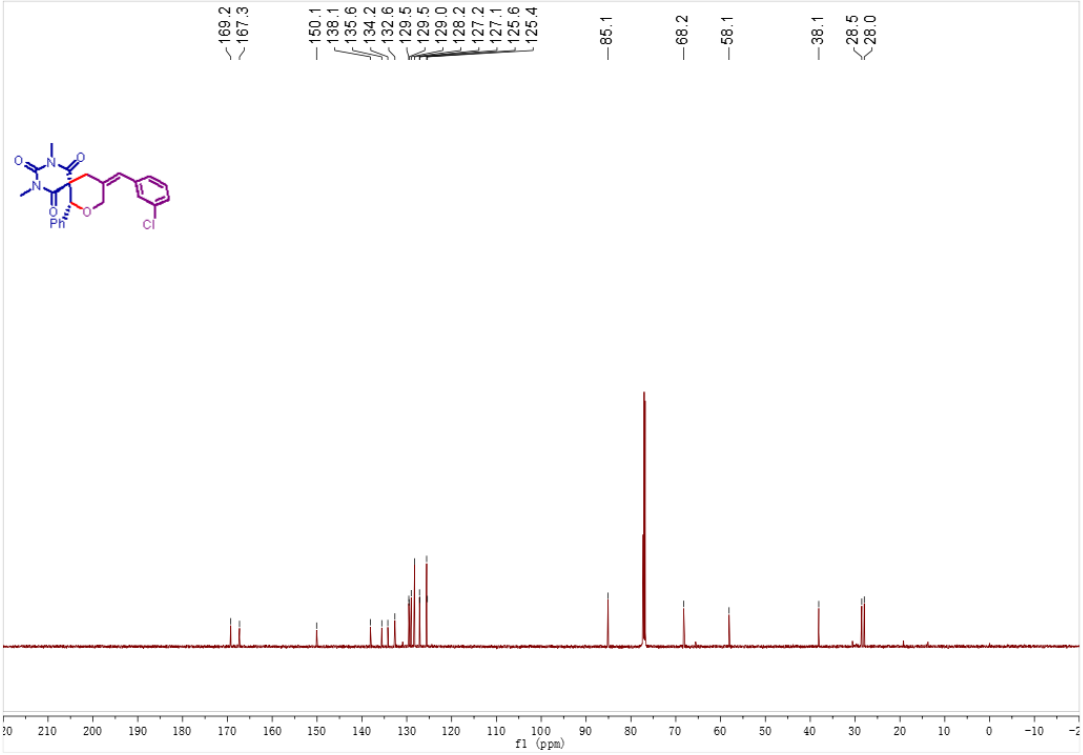

^{13}C (CDCl$_3$, 151 MHz) NMR of compound **37**

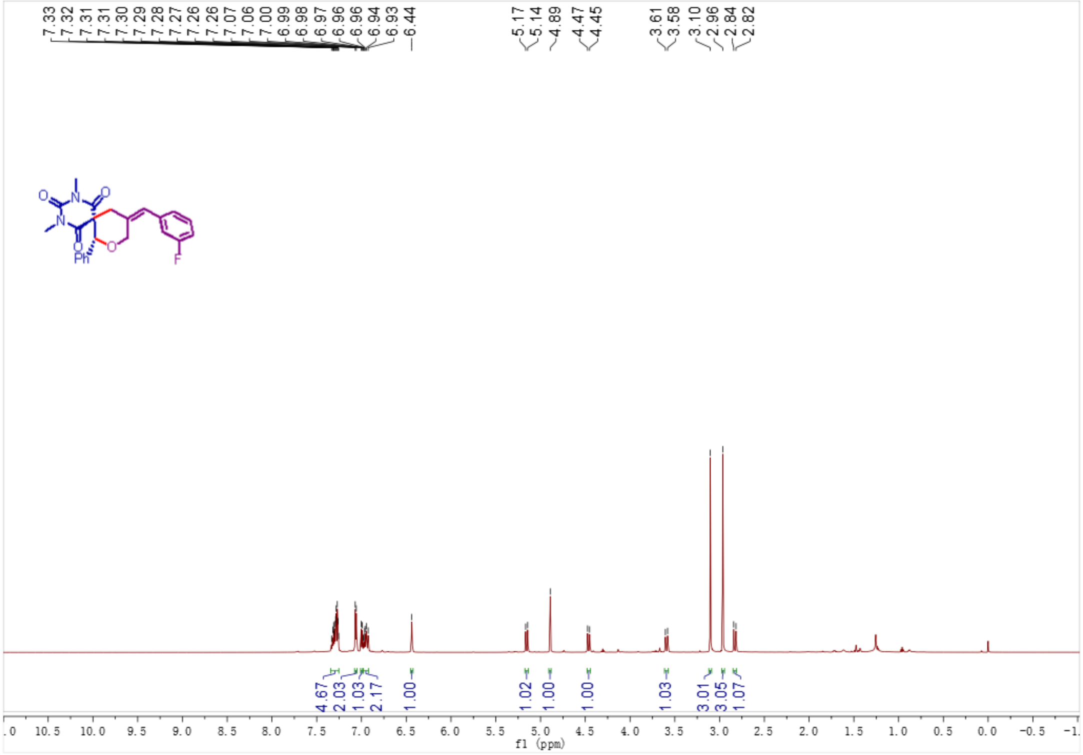

^{1}H (CDCl$_3$, 600 MHz) NMR of compound **38**

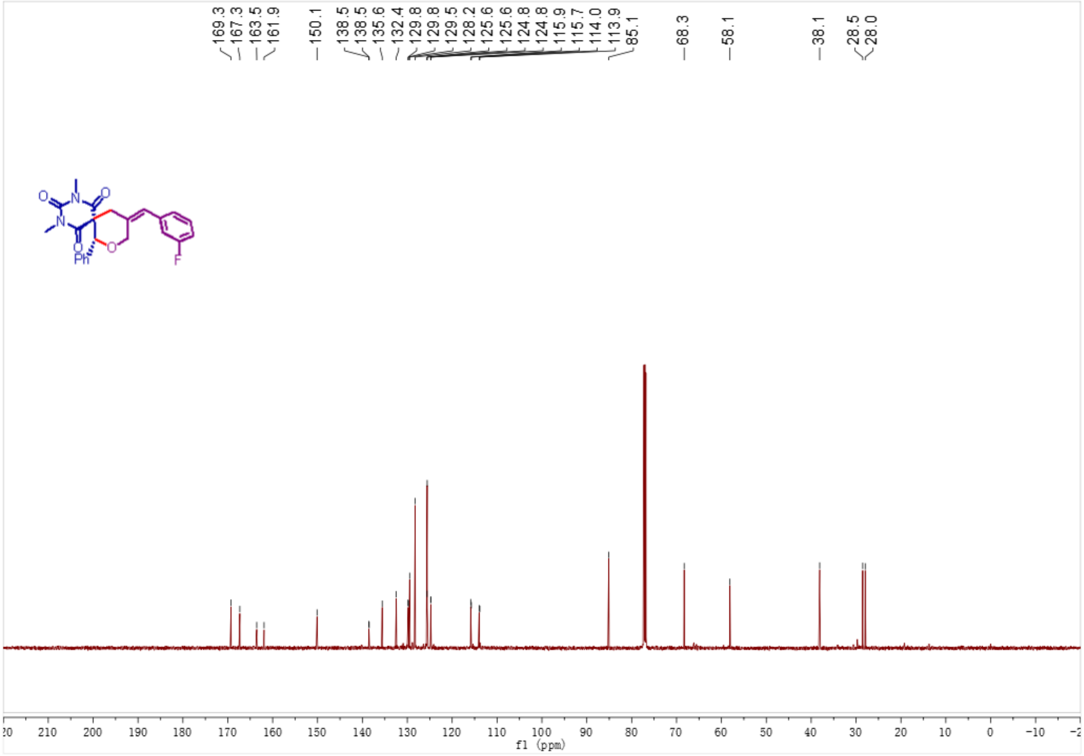

^{13}C ($CDCl_3$, 151 MHz) NMR of compound **38**

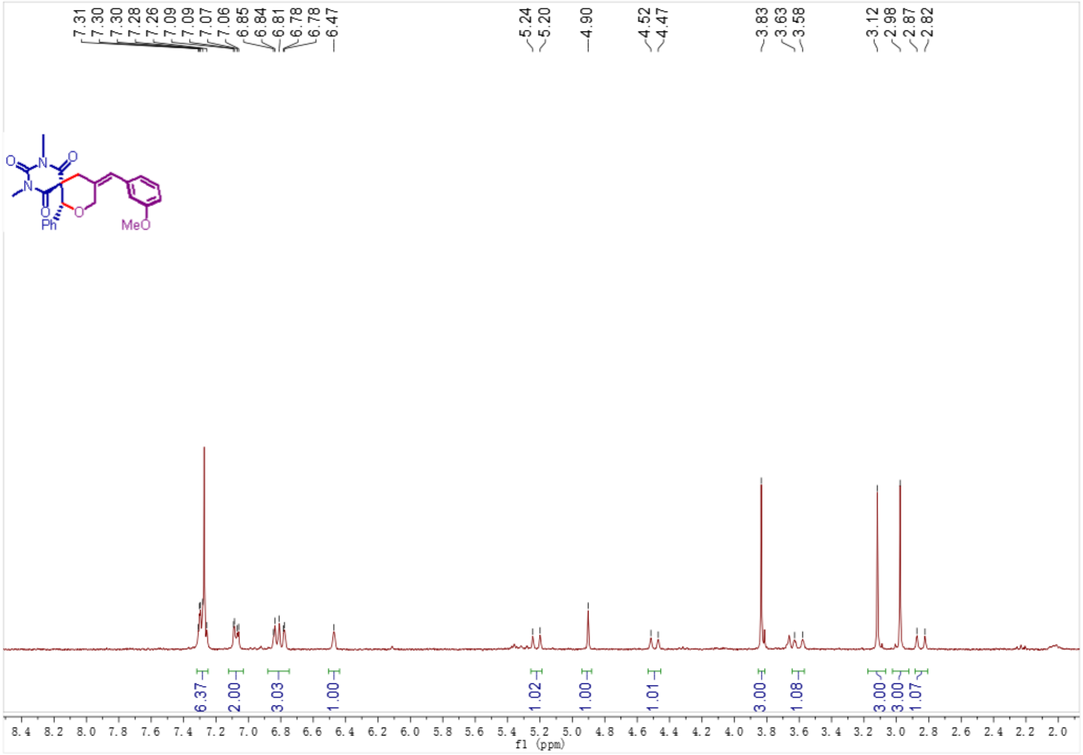

^{1}H ($CDCl_3$, 300 MHz) NMR of compound **39**

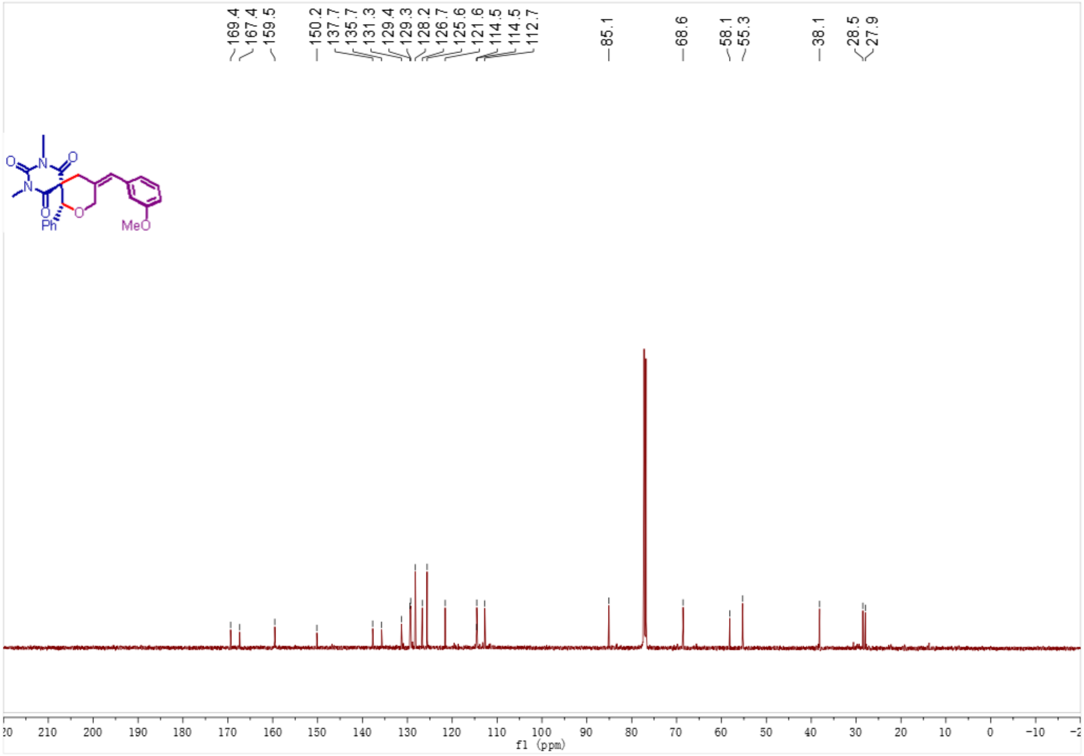

^{13}C ($CDCl_3$, 151 MHz) NMR of compound **39**

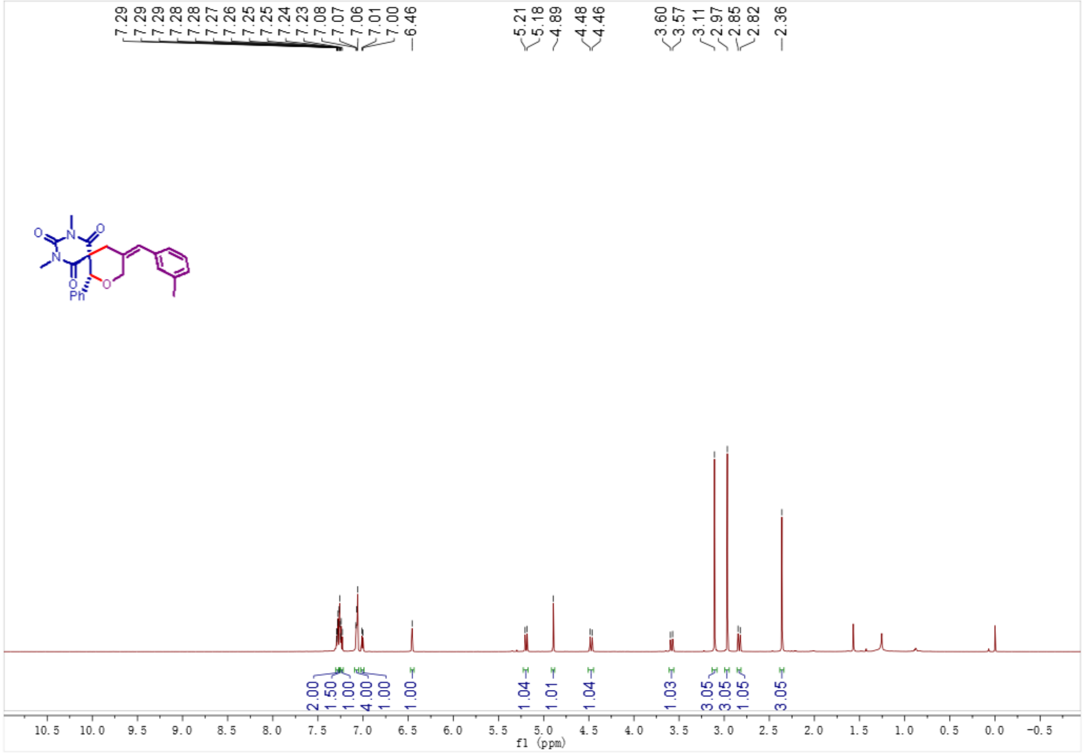

^{1}H ($CDCl_3$, 600 MHz) NMR of compound **40**

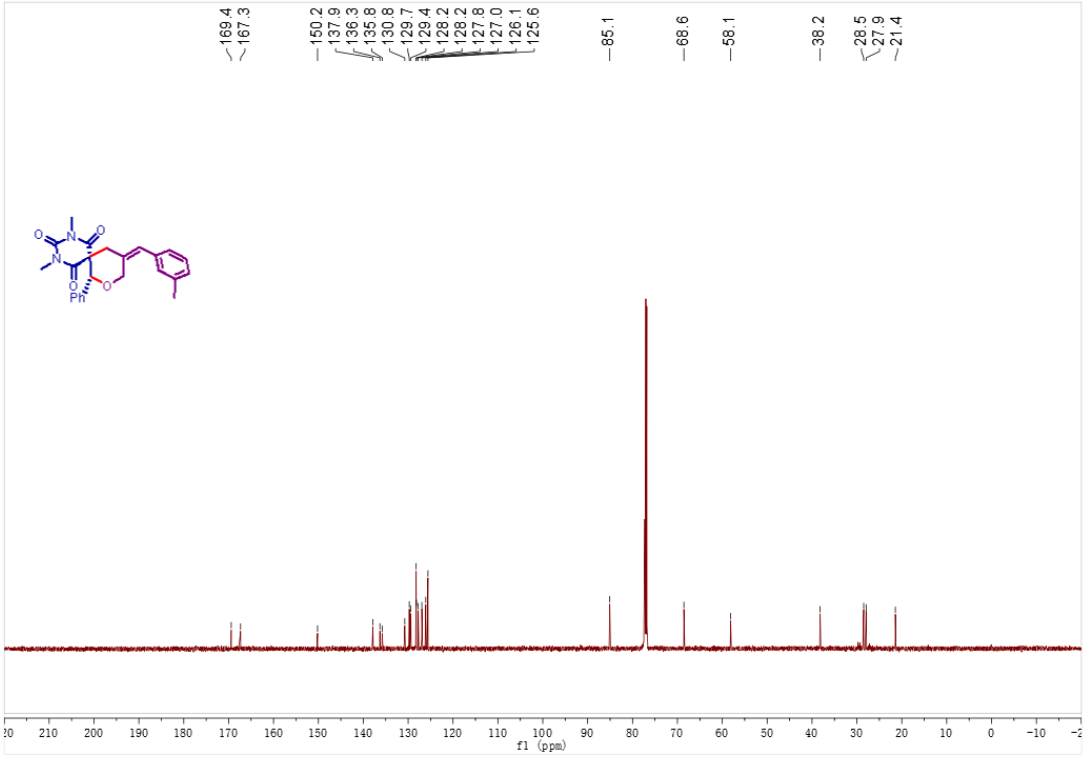

^{13}C ($CDCl_3$, 151 MHz) NMR of compound **40**

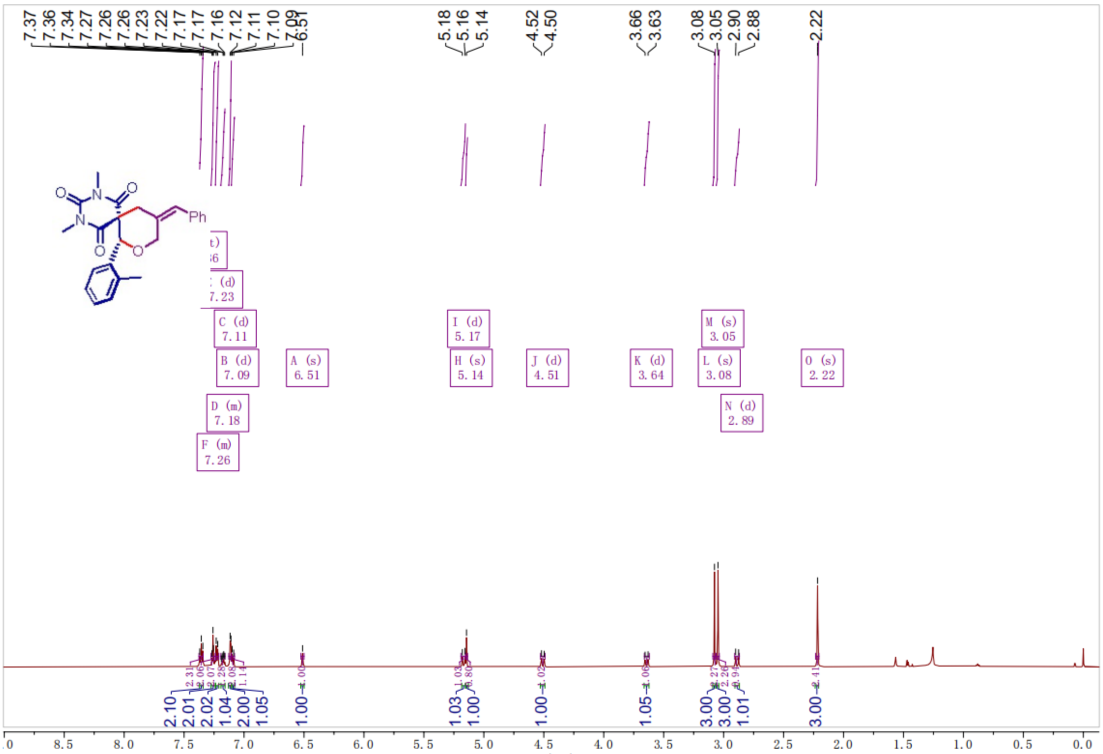

^{1}H ($CDCl_3$, 600 MHz) NMR of compound **41**

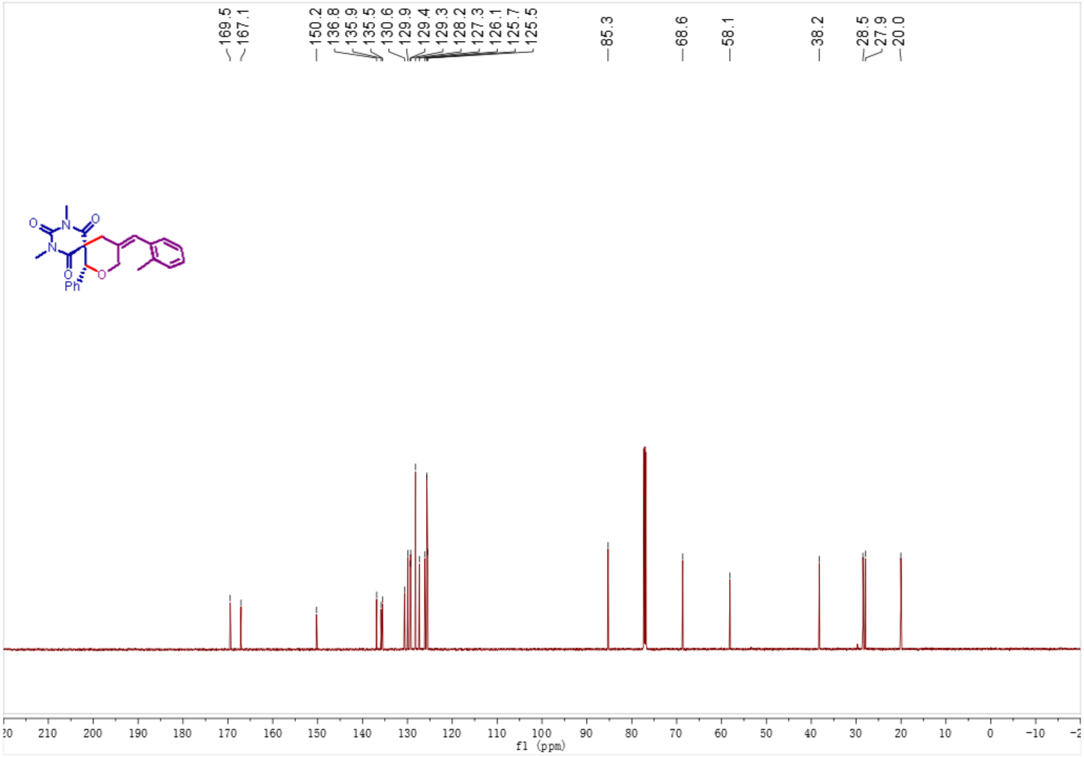

^{13}C ($CDCl_3$, 151 MHz) NMR of compound **41**

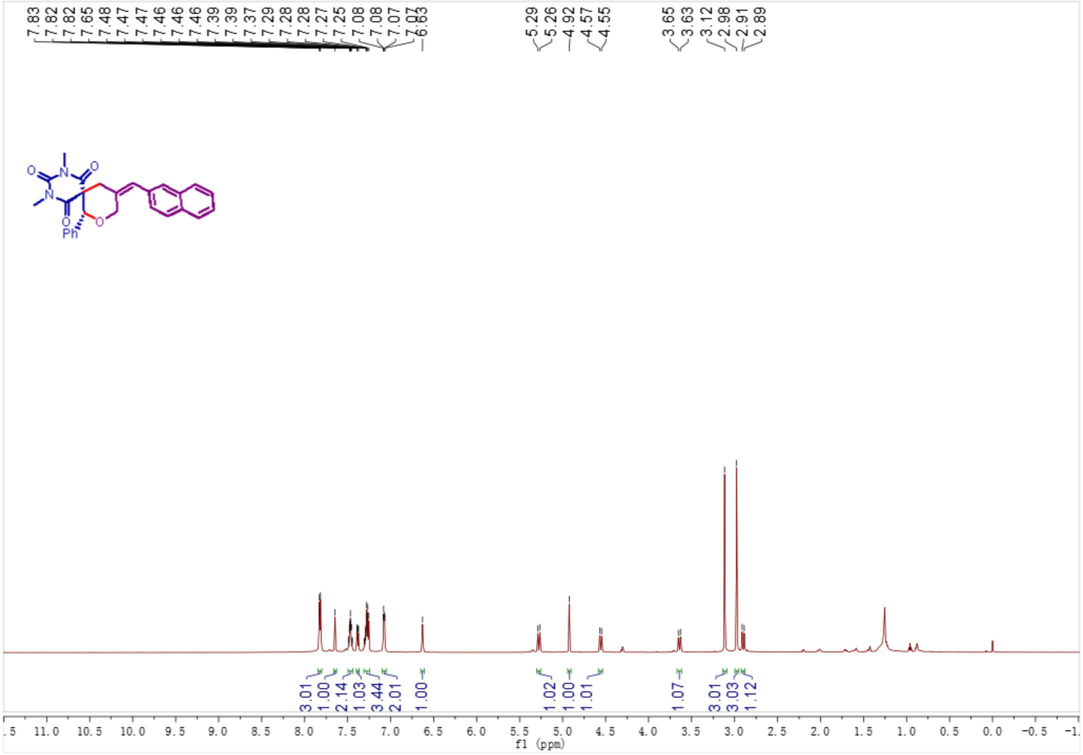

^{1}H ($CDCl_3$, 600 MHz) NMR of compound **42**

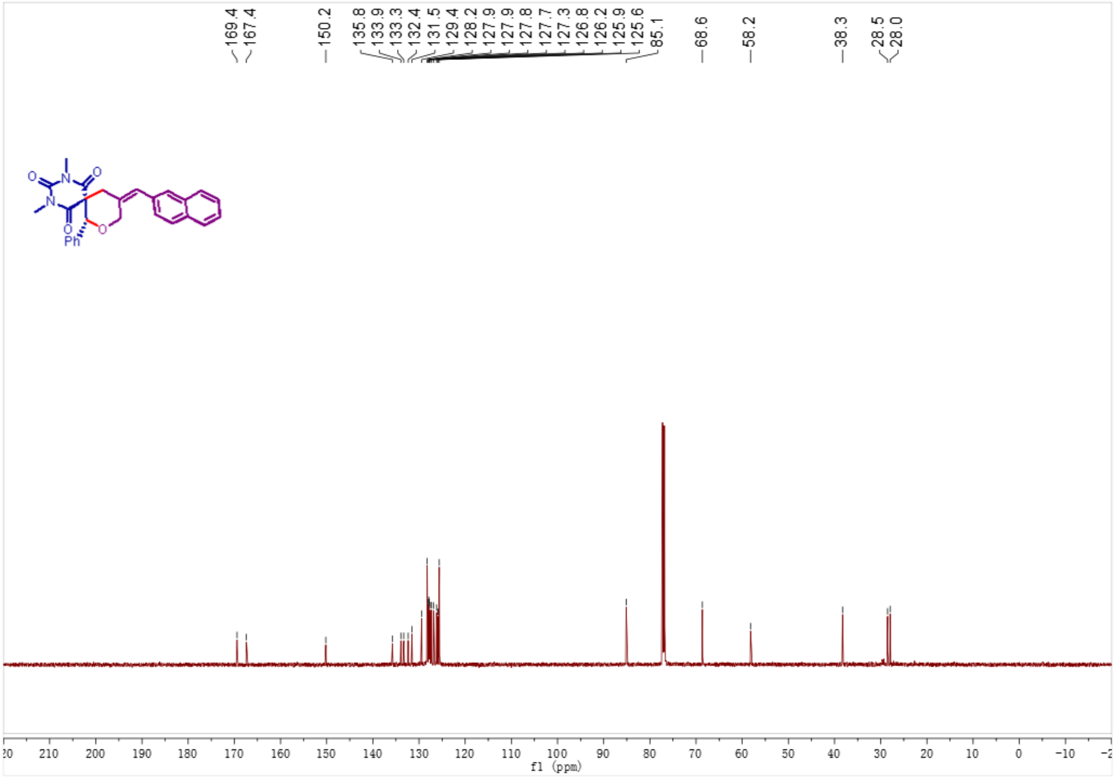

^{13}C ($CDCl_3$, 151 MHz) NMR of compound **42**

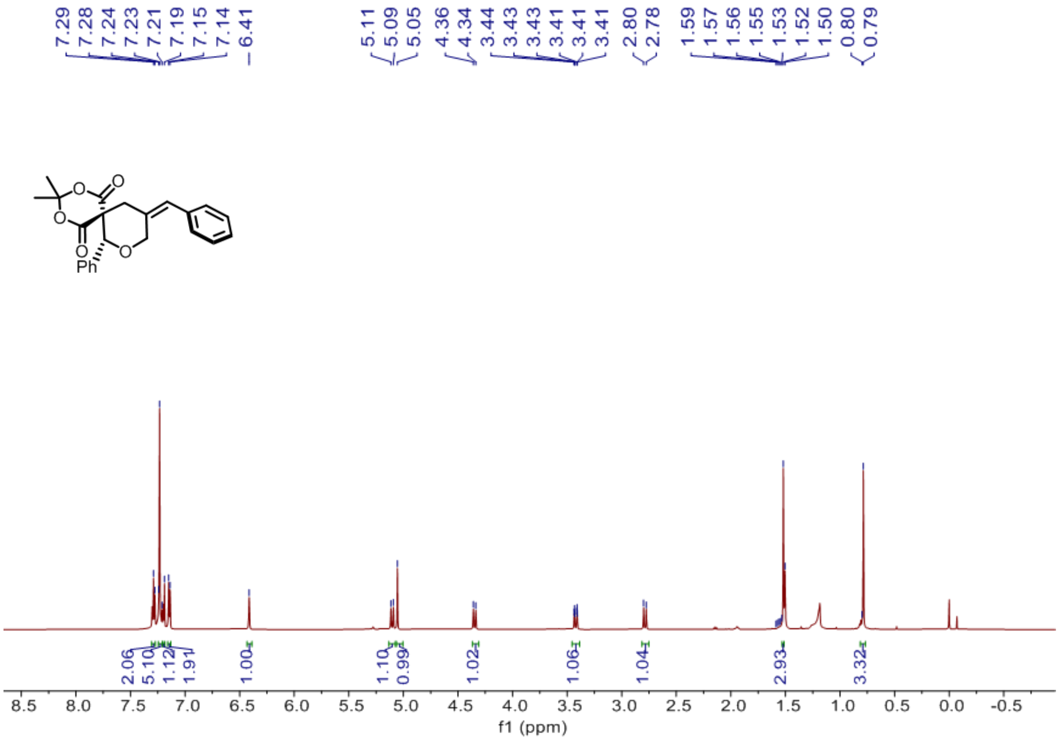

^{1}H ($CDCl_3$, 600 MHz) NMR of compound **44**

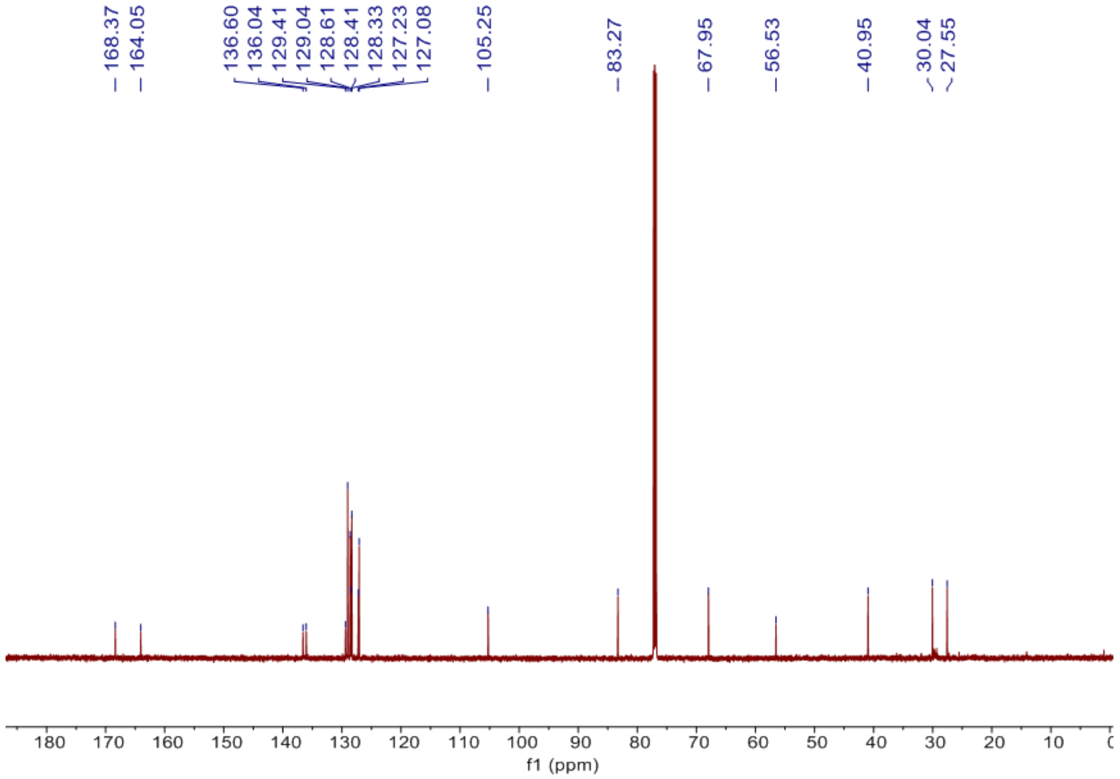

^{13}C ($CDCl_3$, 151 MHz) NMR of compound **44**

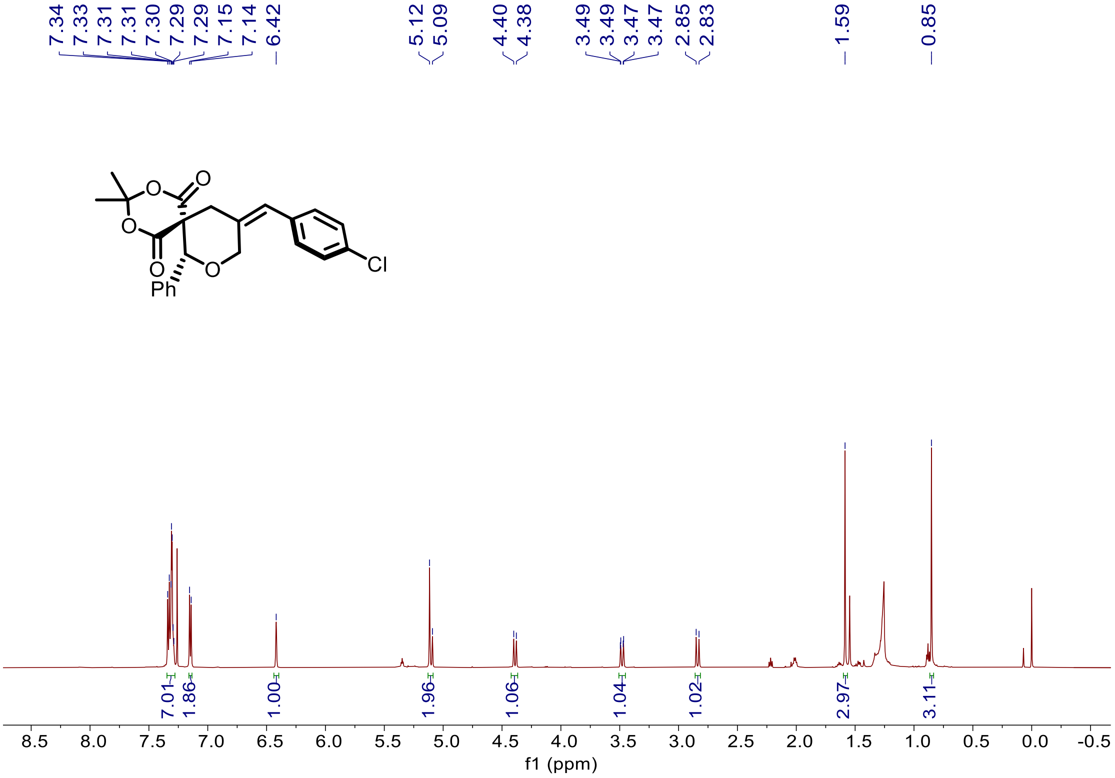

^{1}H ($CDCl_3$, 600 MHz) NMR of compound **45**

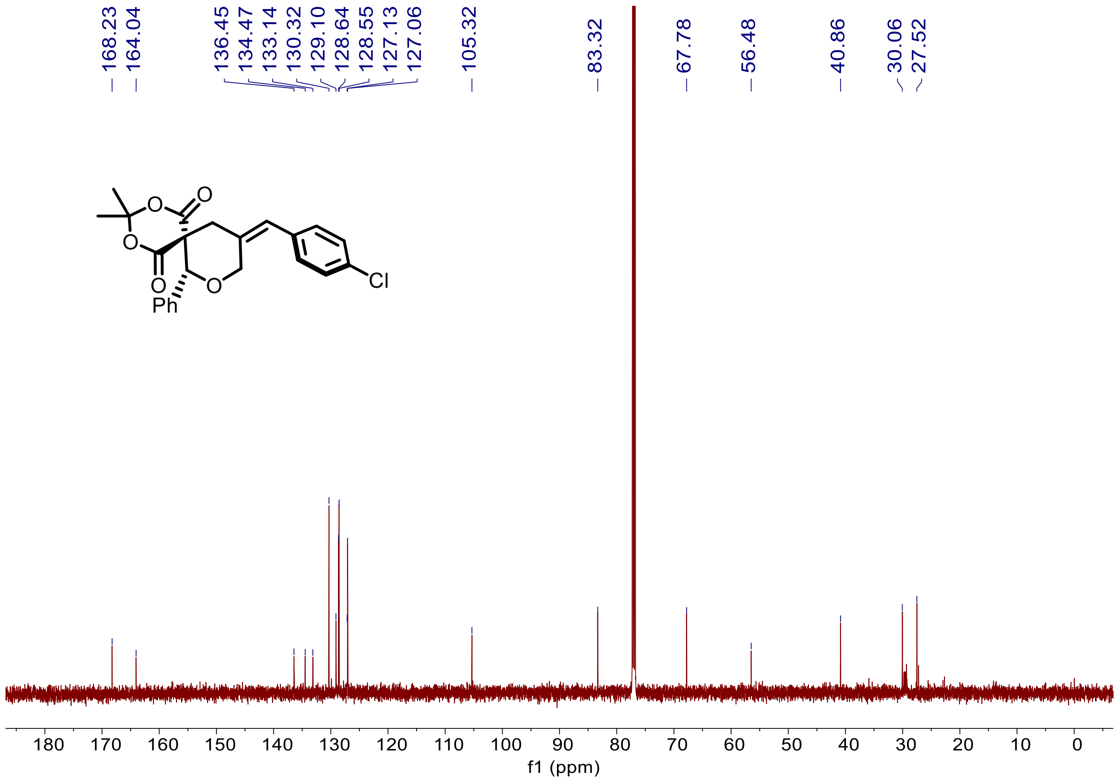

^{13}C ($CDCl_3$, 151 MHz) NMR of compound **45**

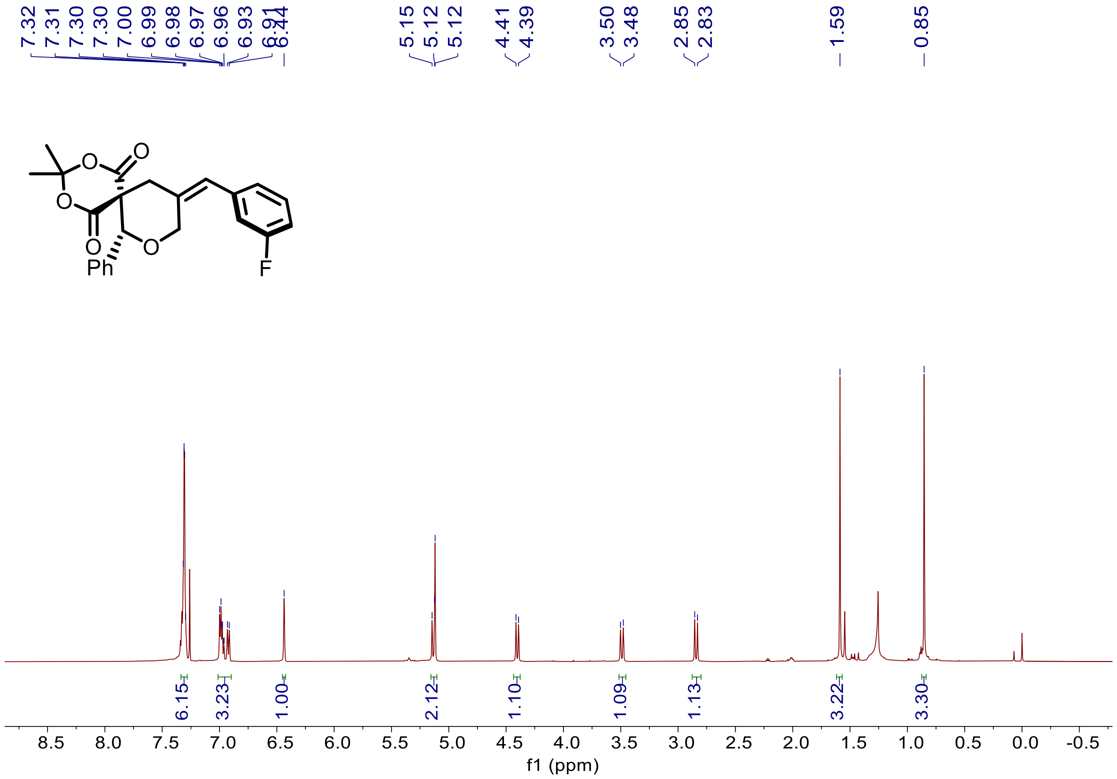

^{1}H ($CDCl_3$, 600 MHz) NMR of compound **46**

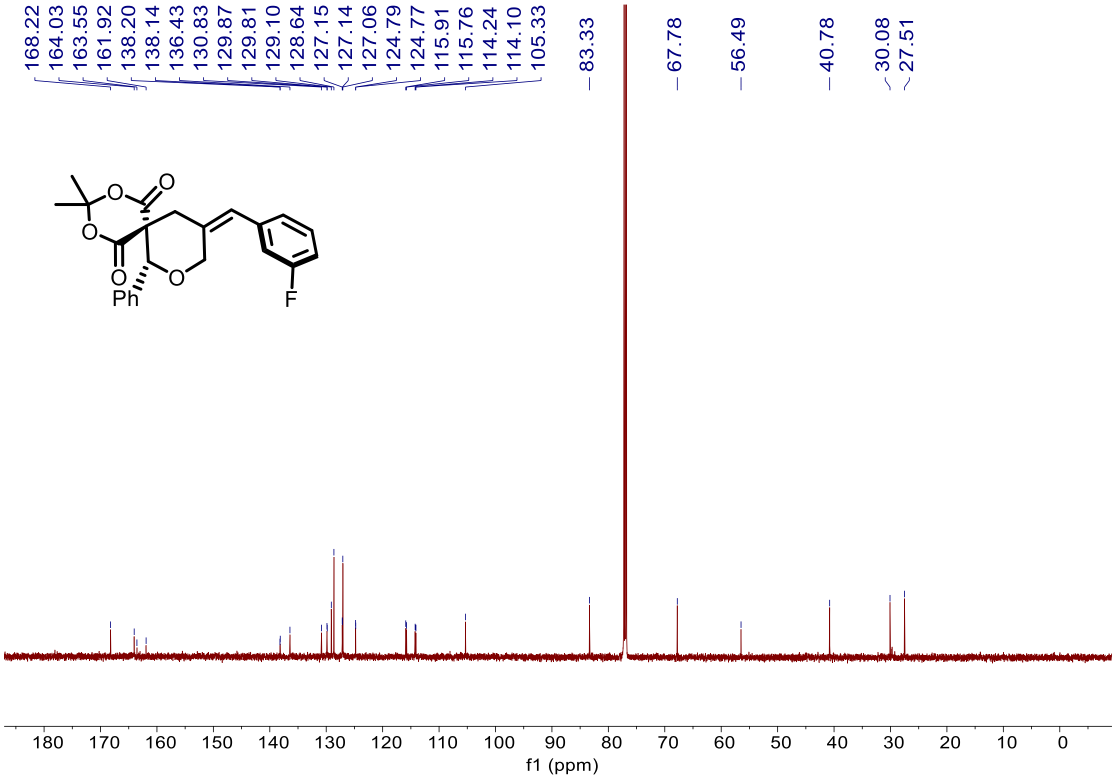

^{13}C ($CDCl_3$, 151 MHz) NMR of compound **46**

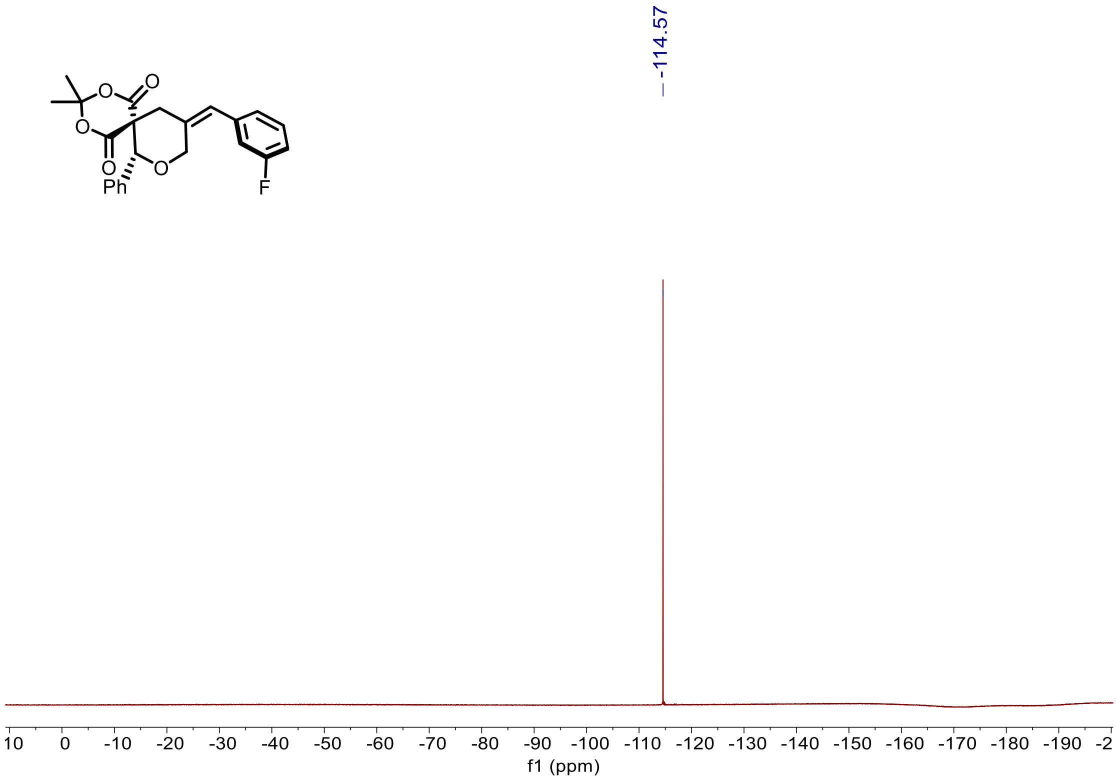

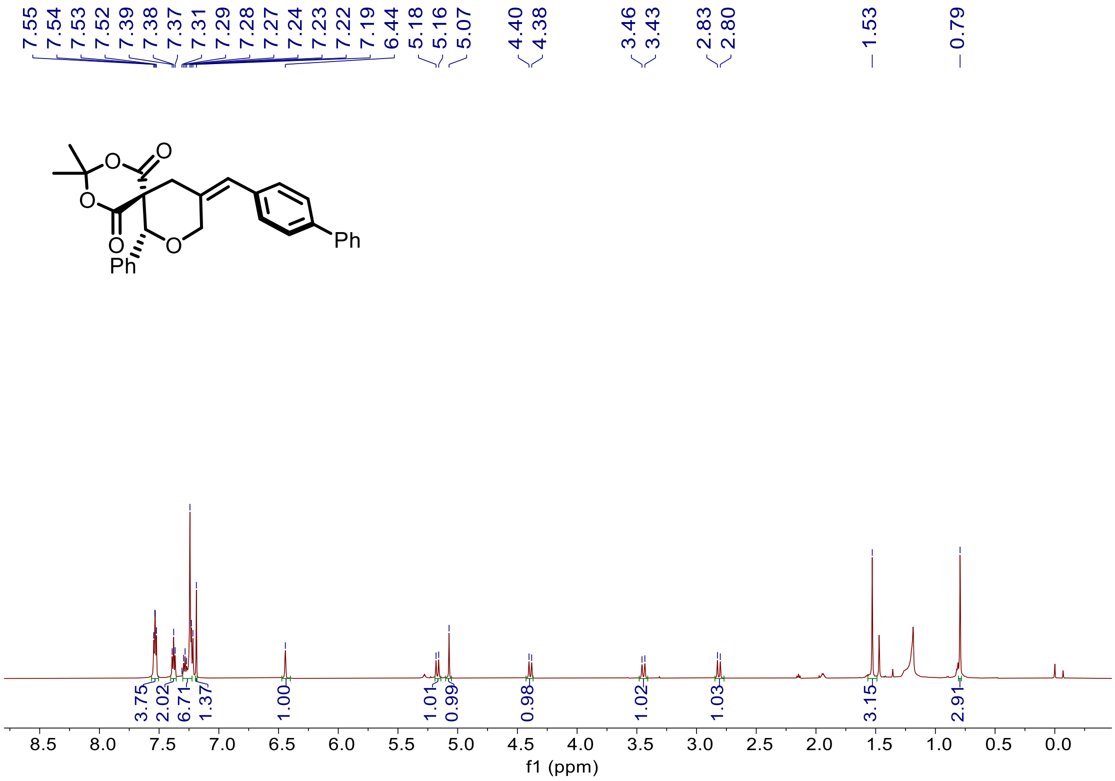

^{1}H ($CDCl_3$, 600 MHz) NMR of compound **47**

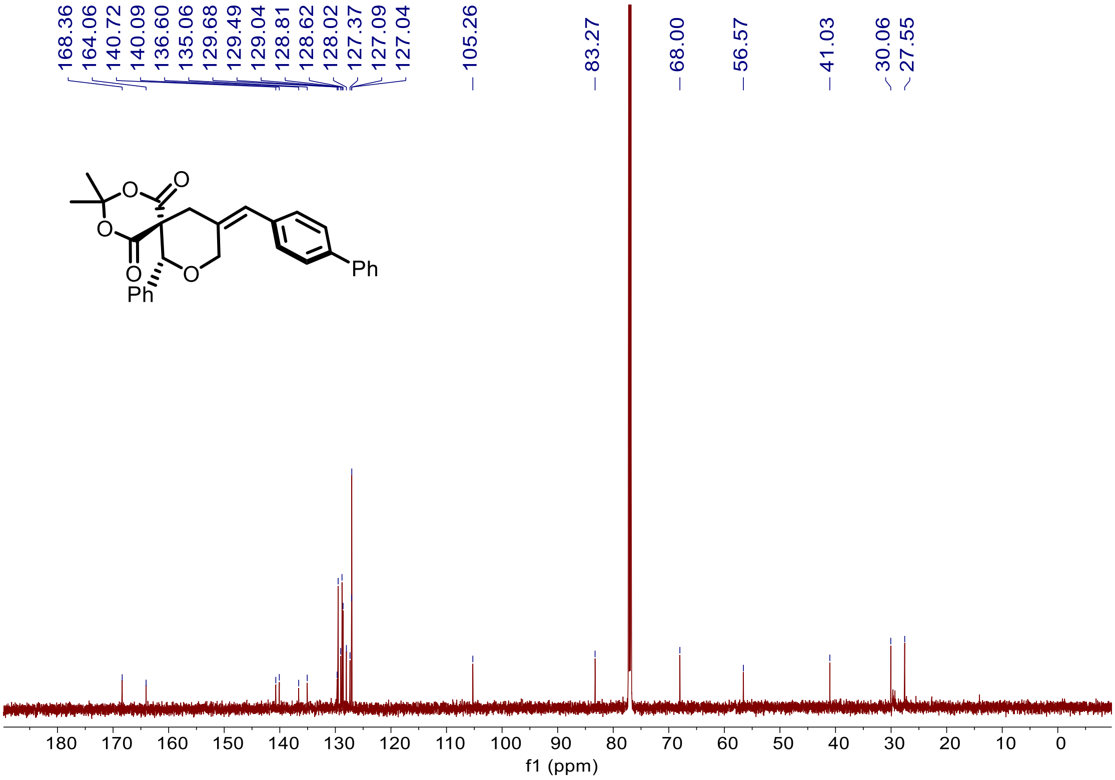

^{13}C ($CDCl_3$, 151 MHz) NMR of compound **47**

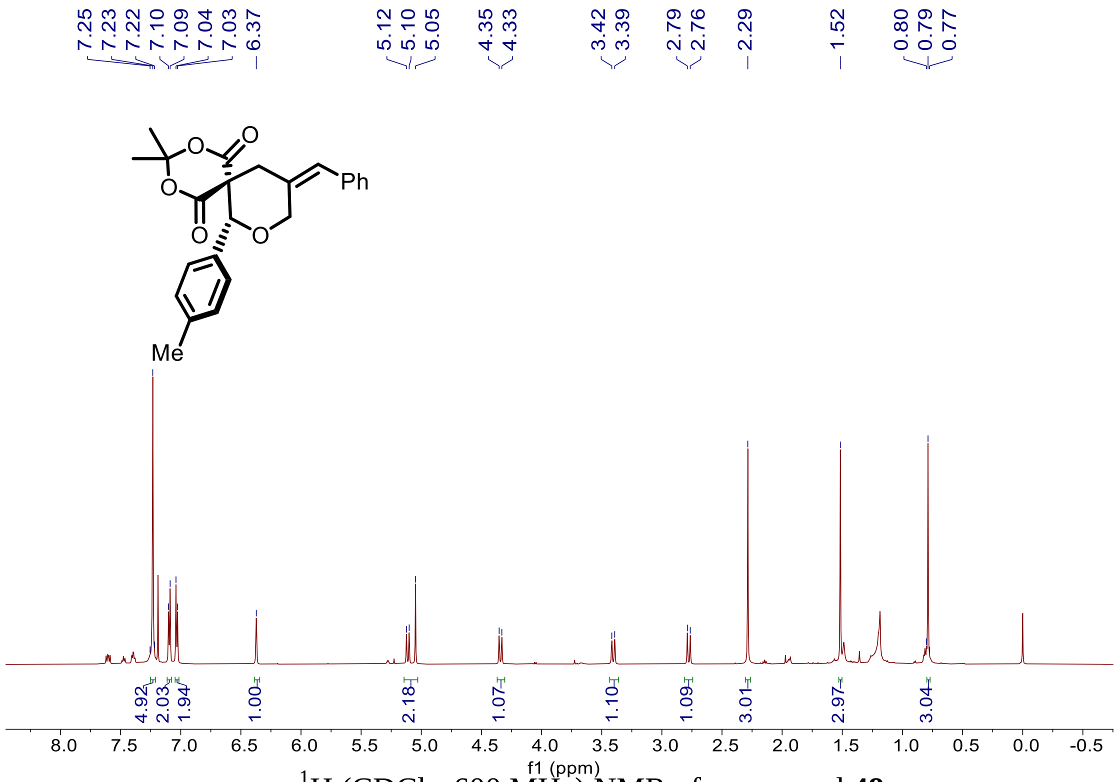

^{1}H ($CDCl_3$, 600 MHz) NMR of compound **48**

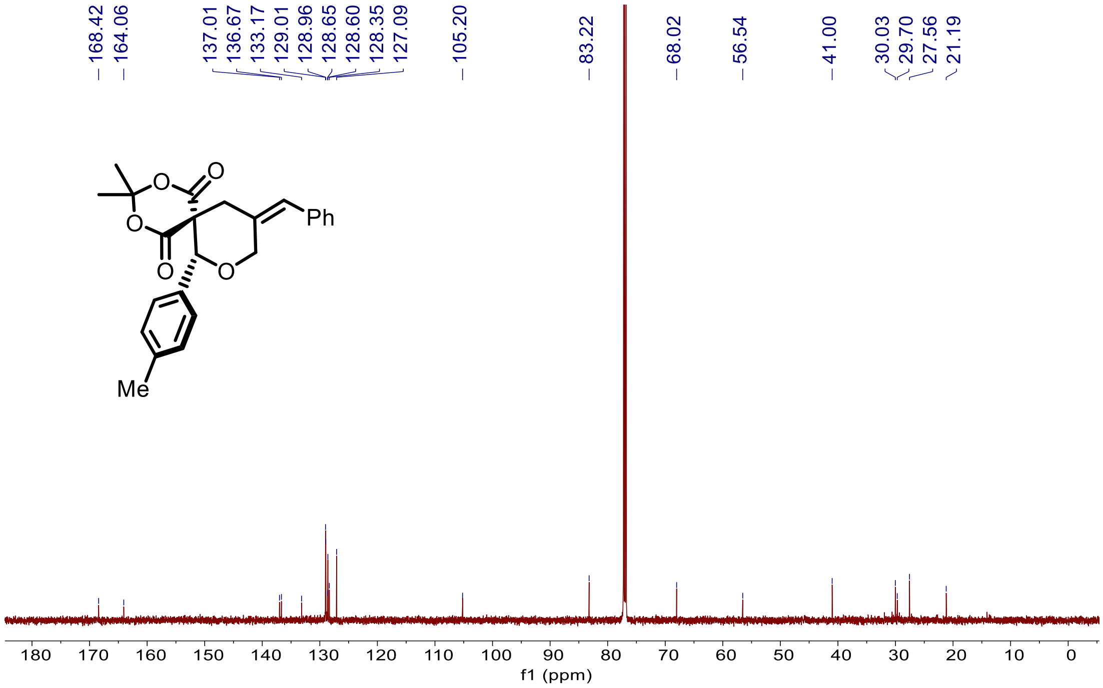

^{13}C ($CDCl_3$, 151 MHz) NMR of compound **48**

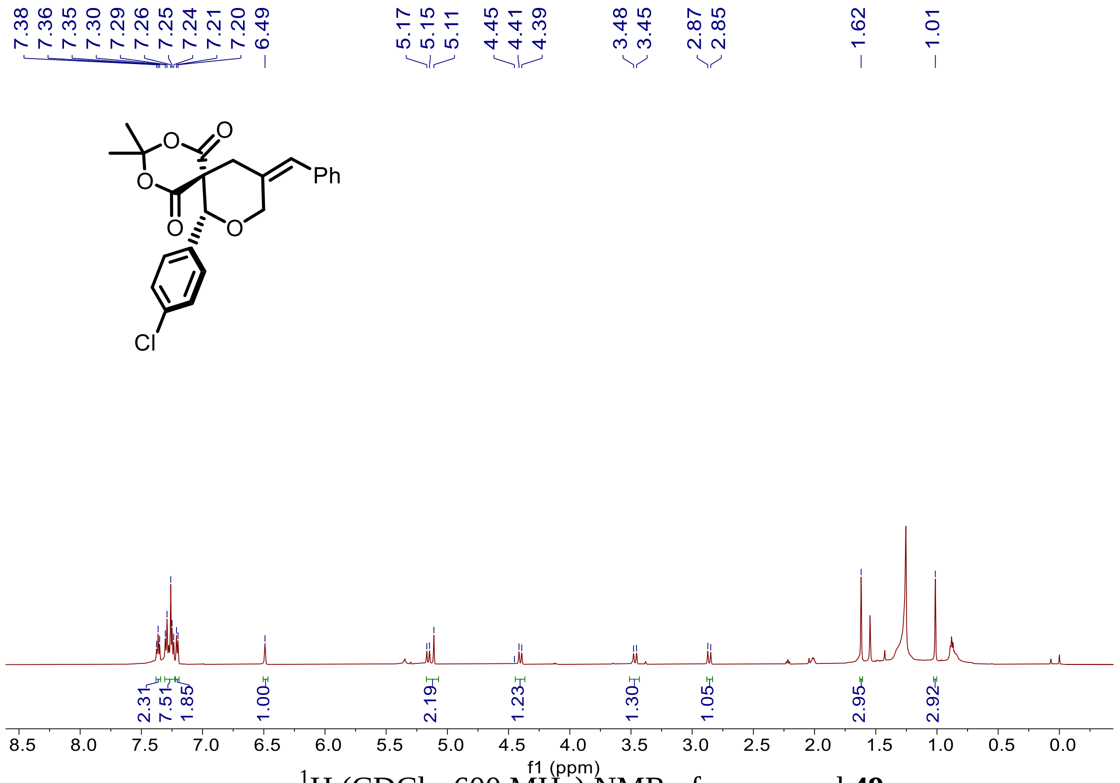

^{1}H ($CDCl_3$, 600 MHz) NMR of compound **49**

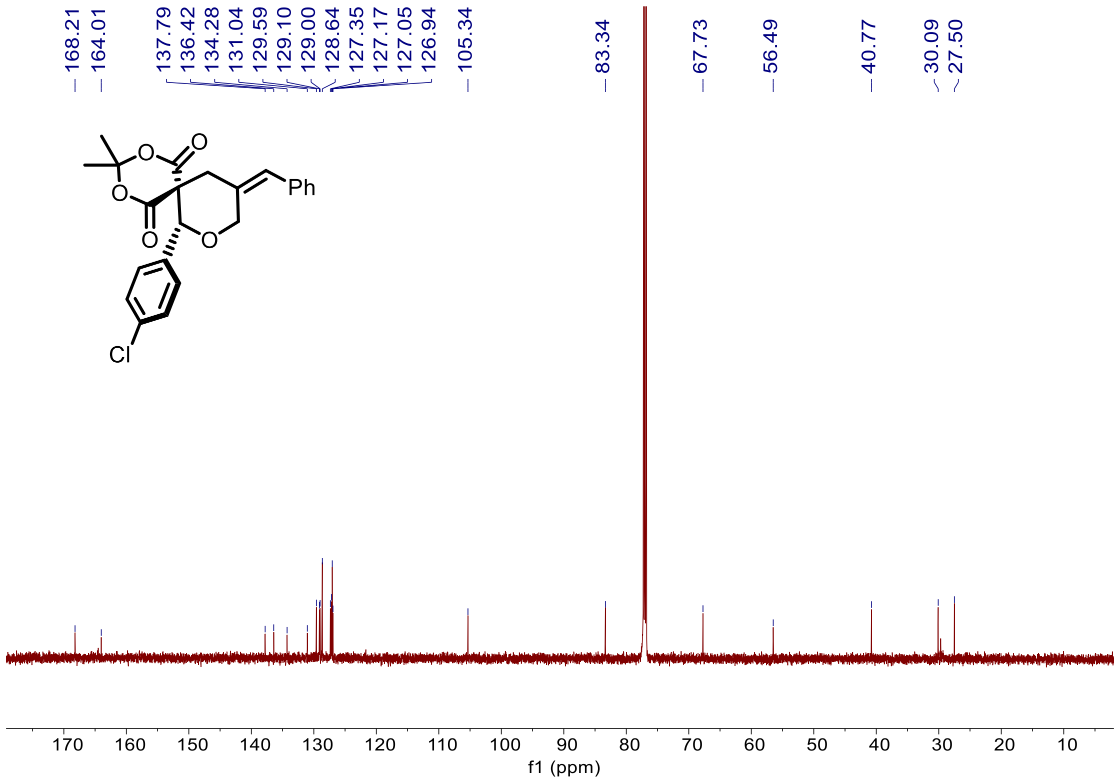

^{13}C ($CDCl_3$, 151 MHz) NMR of compound **49**

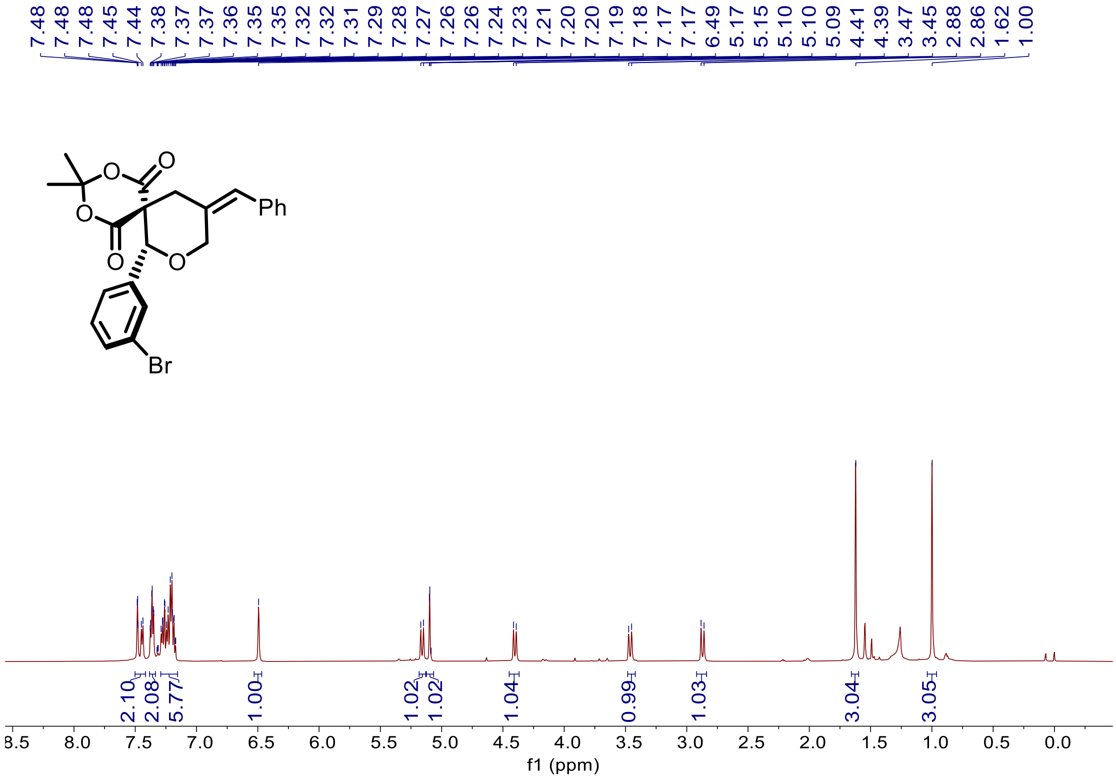

^{1}H ($CDCl_3$, 600 MHz) NMR of compound **50**

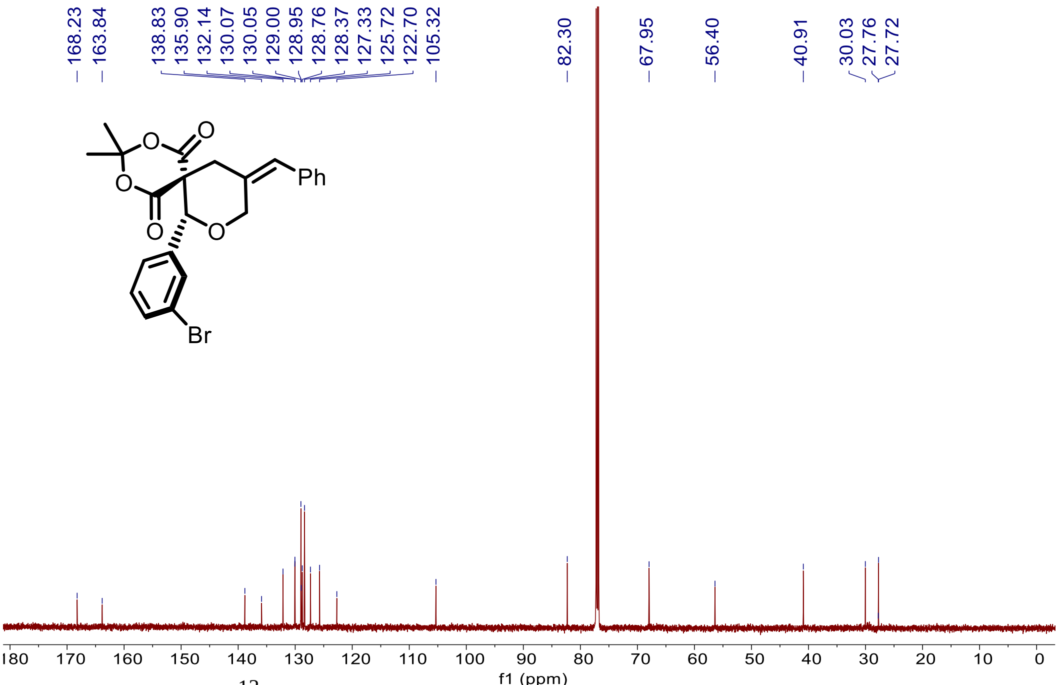

^{13}C ($CDCl_3$, 151 MHz) NMR of compound **50**

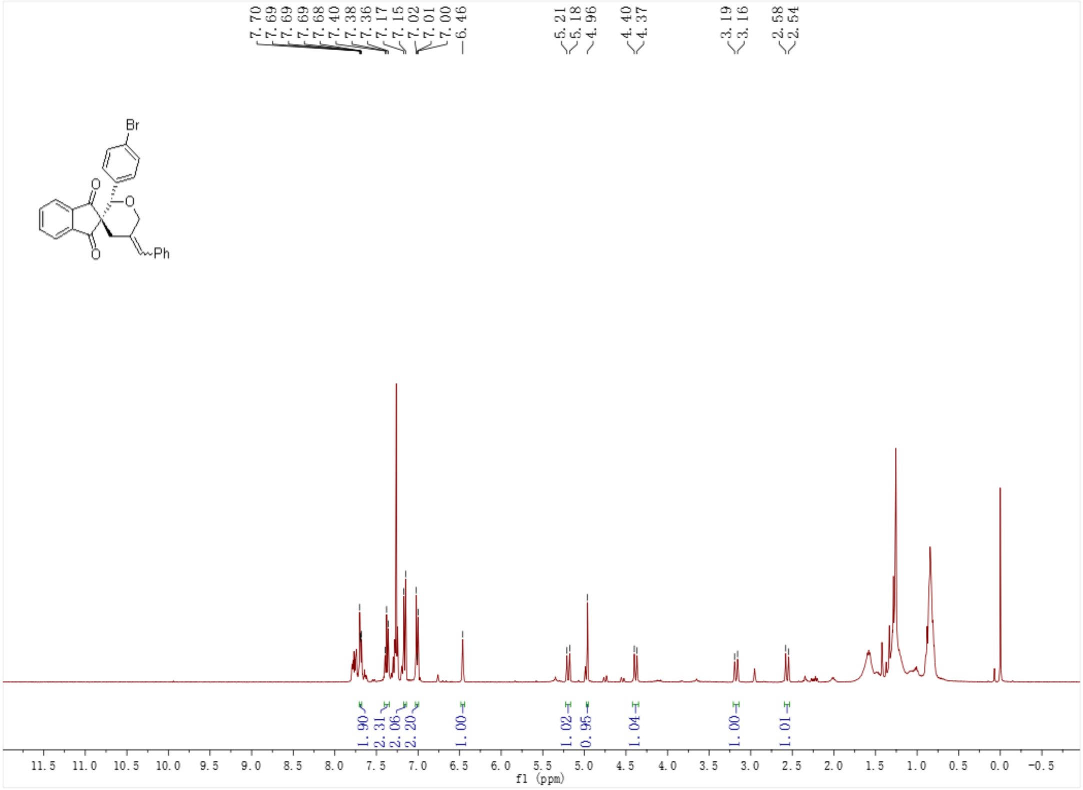

^{1}H ($CDCl_3$, 300 MHz) NMR of compound **52**

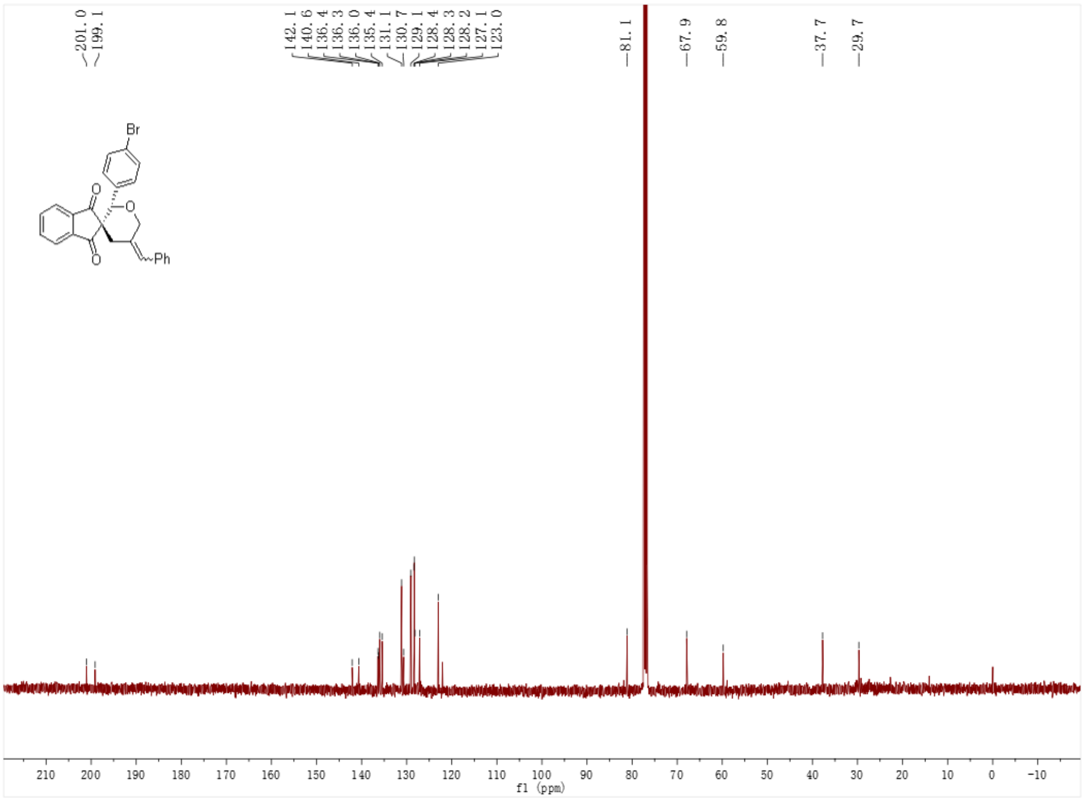

^{13}C ($CDCl_3$, 75 MHz) NMR of compound **52**

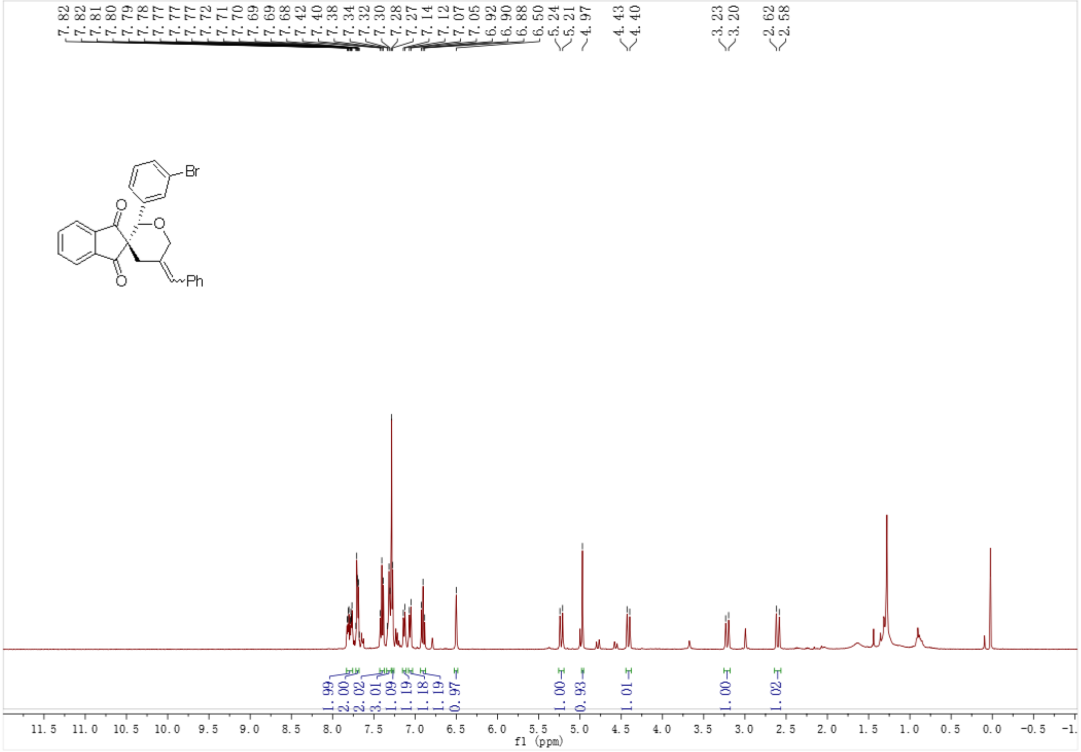

^{1}H ($CDCl_3$, 300 MHz) NMR of compound **53**

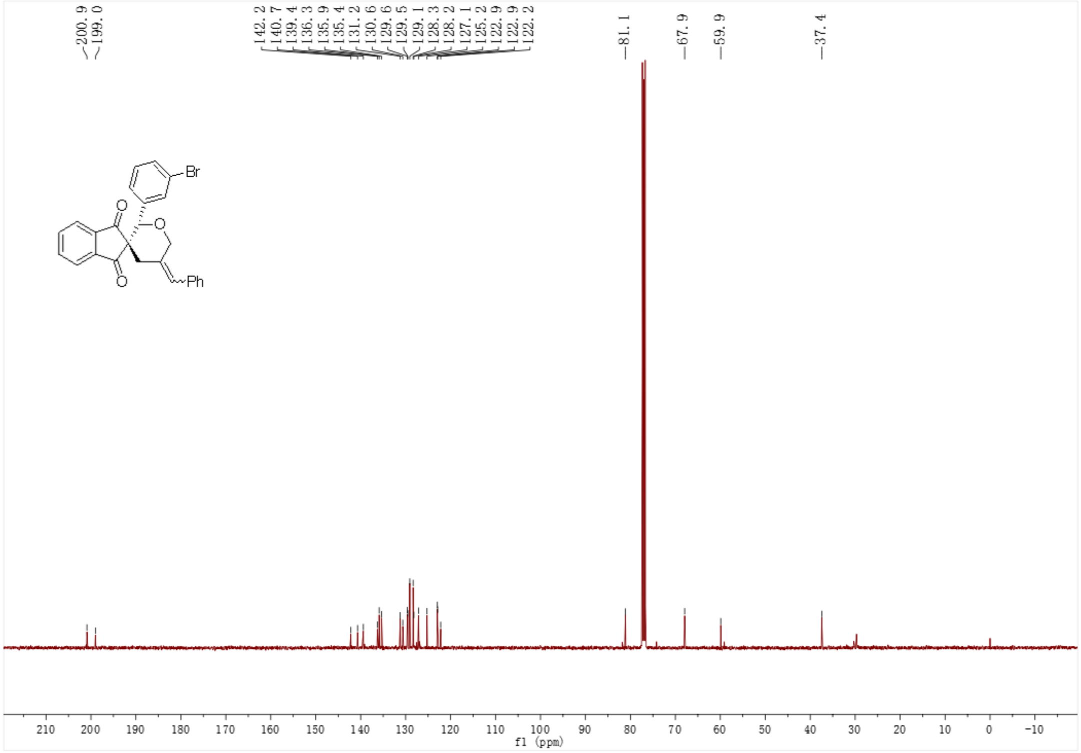

^{13}C ($CDCl_3$, 75 MHz) NMR of compound **53**

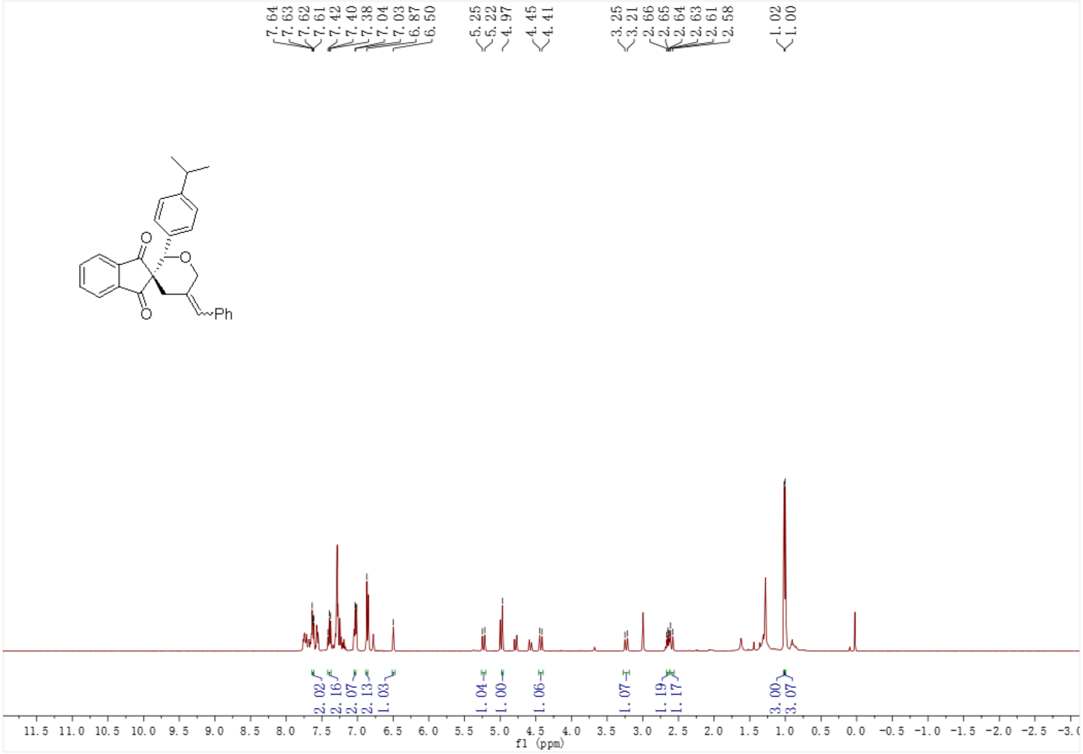

^{1}H ($CDCl_3$, 300 MHz) NMR of compound **54**

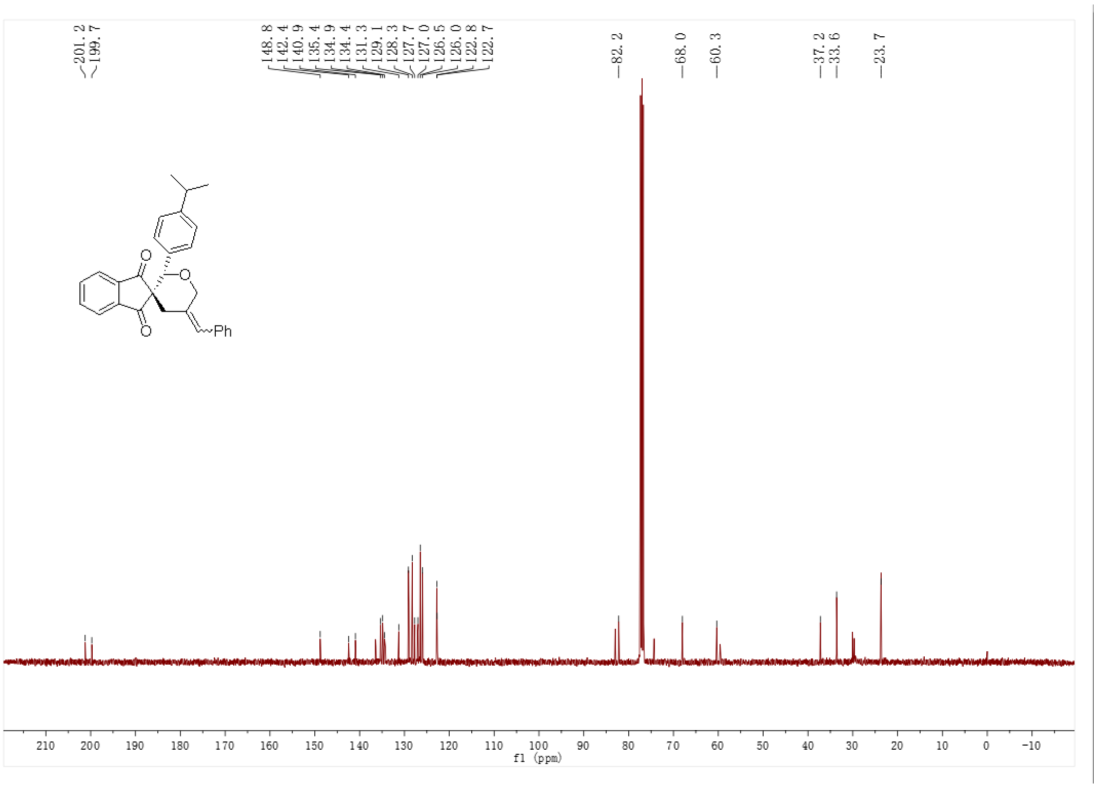

^{13}C ($CDCl_3$, 75 MHz) NMR of compound **54**

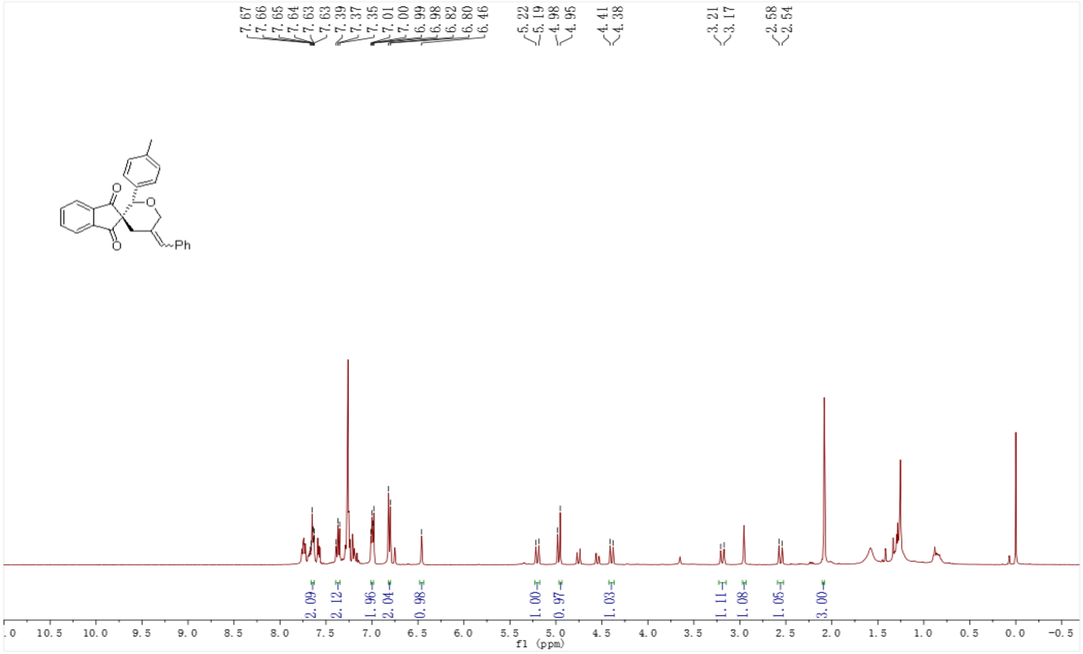

^{1}H ($CDCl_3$, 300 MHz) NMR of compound **55**

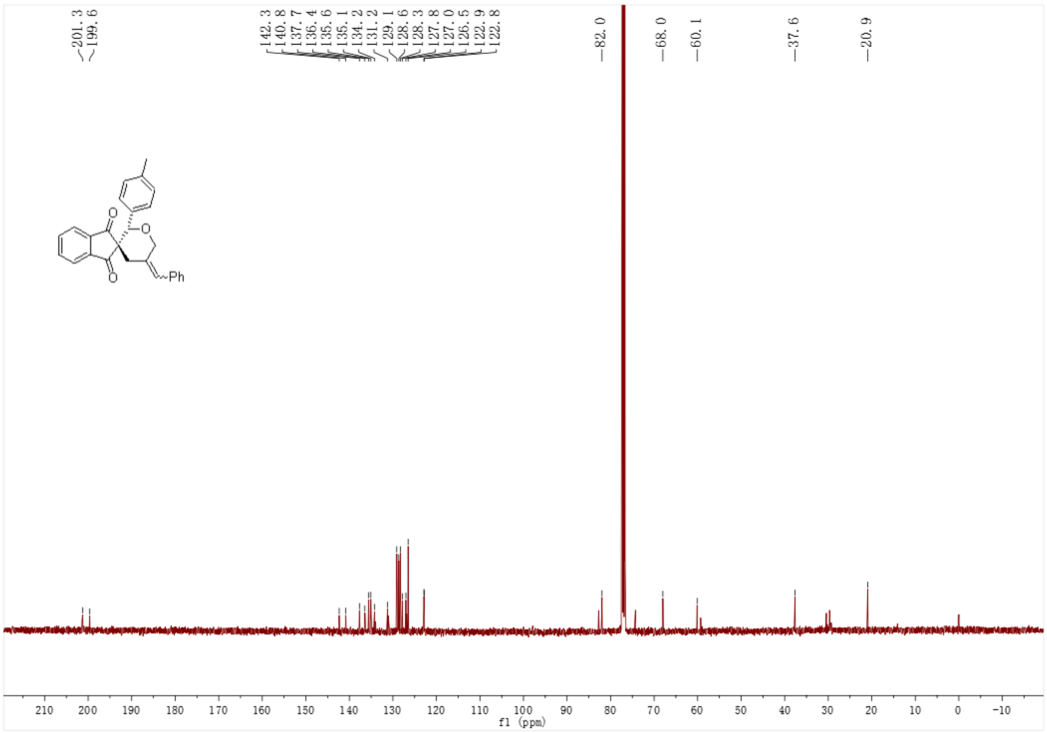

^{13}C ($CDCl_3$, 75 MHz) NMR of compound **55**

6. Copies of HPLC Chromatograms

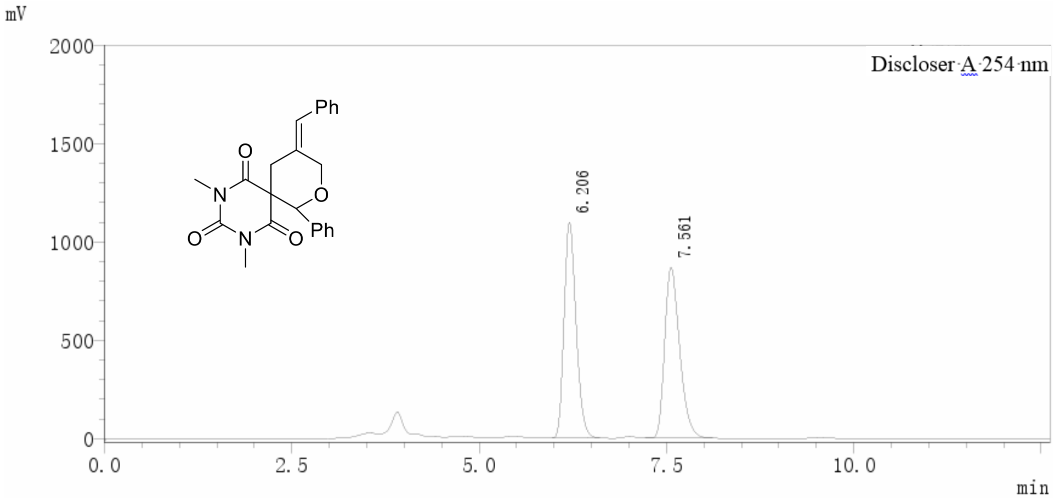

Discloser A 254 nm

Peak	Retention time (min)	Area (%)
1	6.206	50.257
2	7.561	49.743
total		100

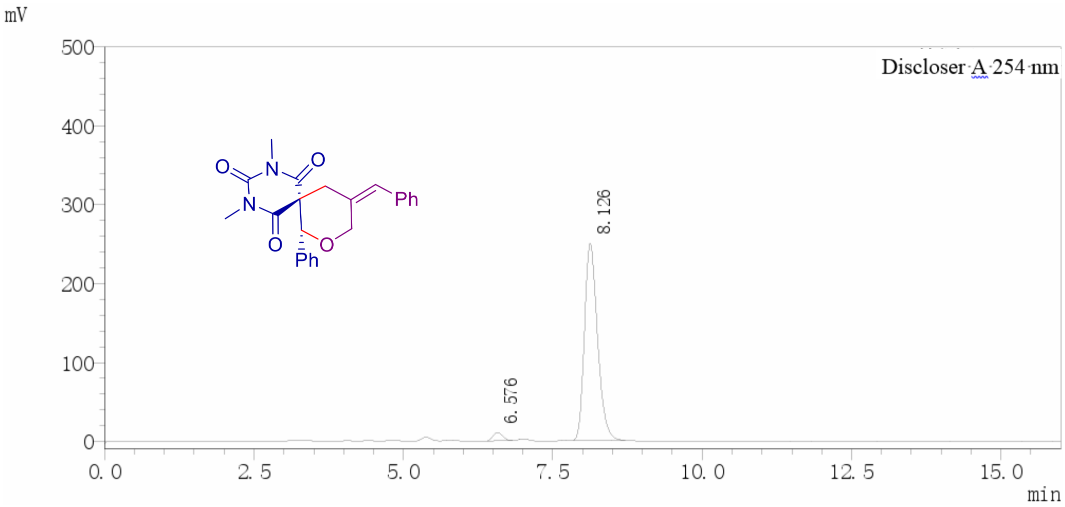

Discloser A 254 nm

Peak	Retention time (min)	Area (%)
1	6.576	2.991
2	8.126	97.009
total		100

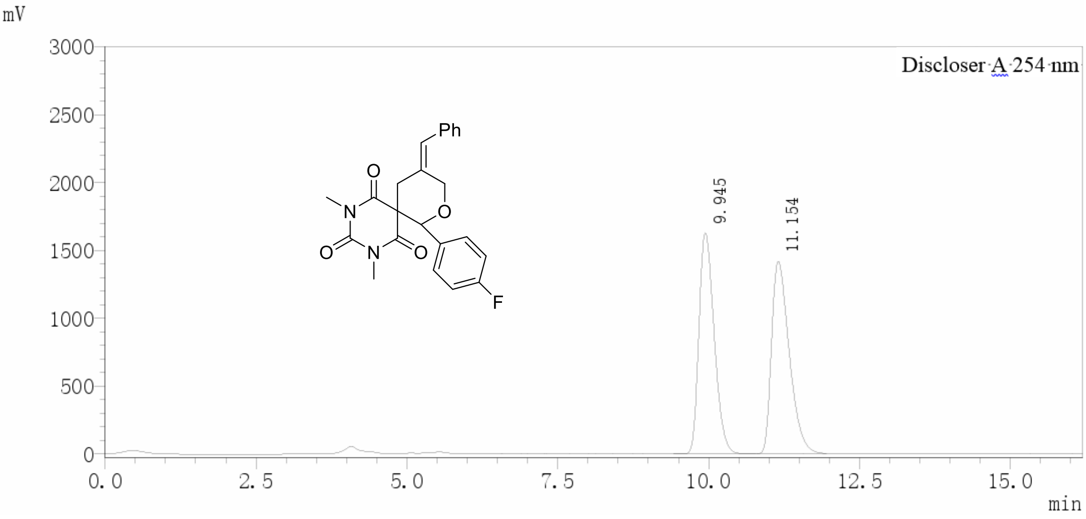

Discloser A 254 nm

Peak	Retention time (min)	Area (%)
1	9.945	49.884
2	11.154	50.116
total		100

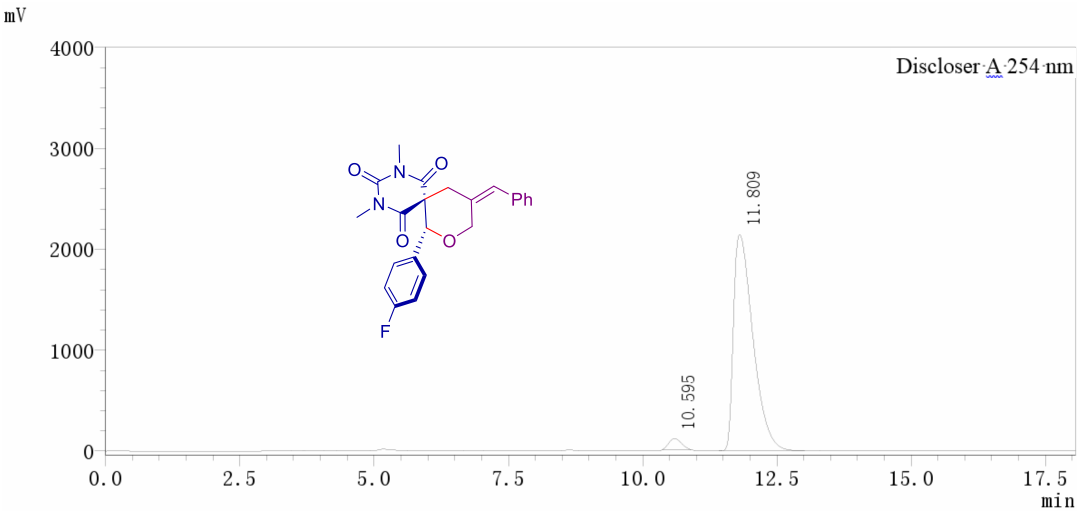

Discloser A 254 nm

Peak	Retention time (min)	Area (%)
1	10.595	3.190
2	11.809	96.810
total		100

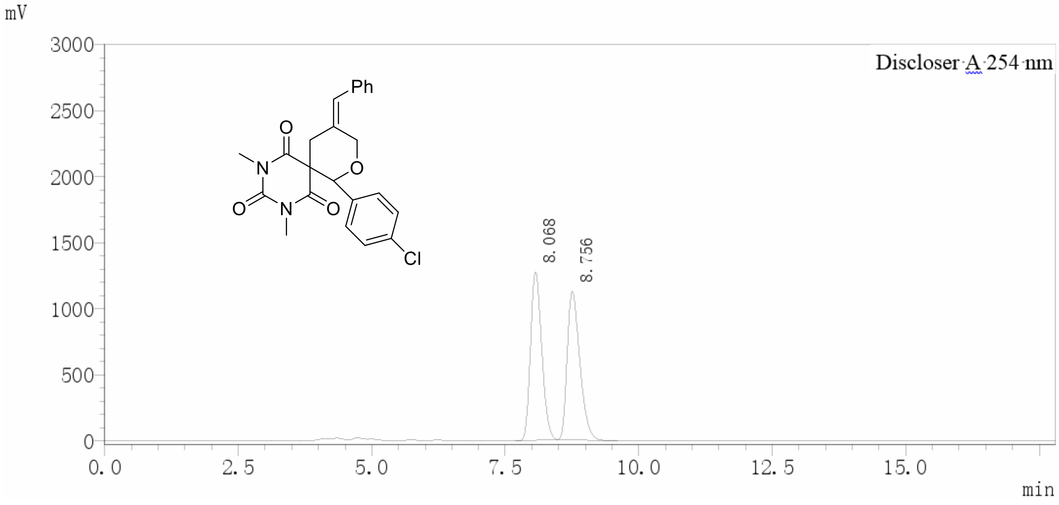

Discloser A 254 nm

Peak	Retention time (min)	Area (%)
1	8.068	50.162
2	8.756	49.838
total		100

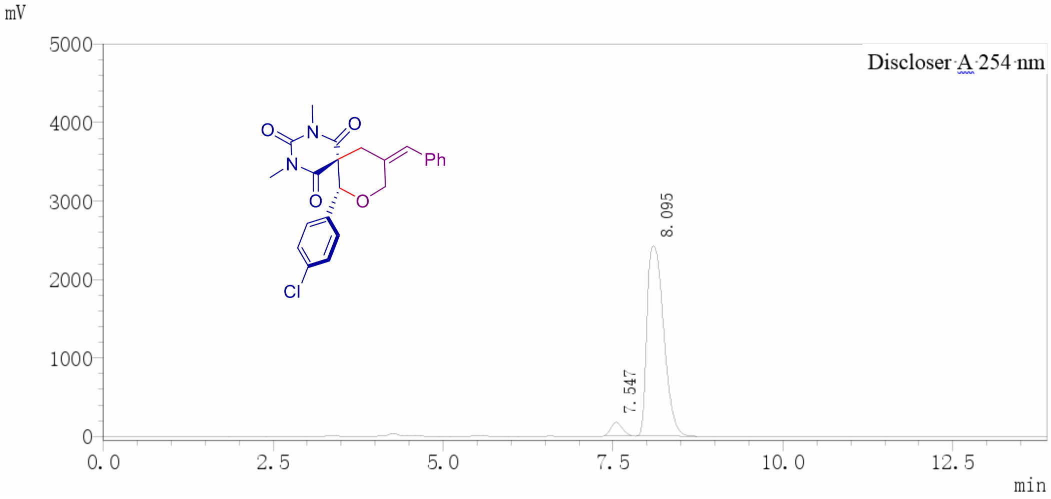

Discloser A 254 nm

Peak	Retention time (min)	Area (%)
1	7.547	4.452
2	8.095	95.548
total		100

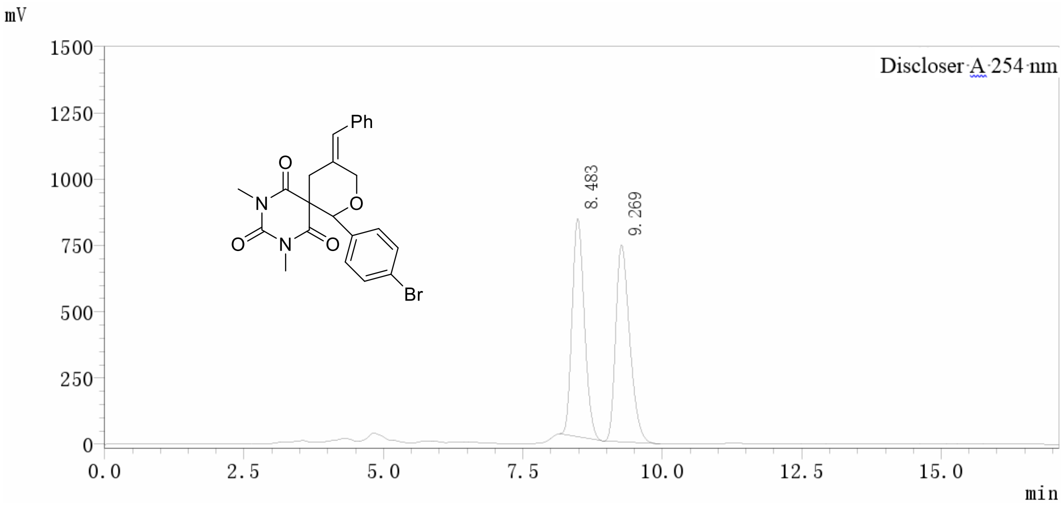

Discloser A 254 nm

Peak	Retention time (min)	Area (%)
1	8.483	49.210
2	9.269	50.790
total		100

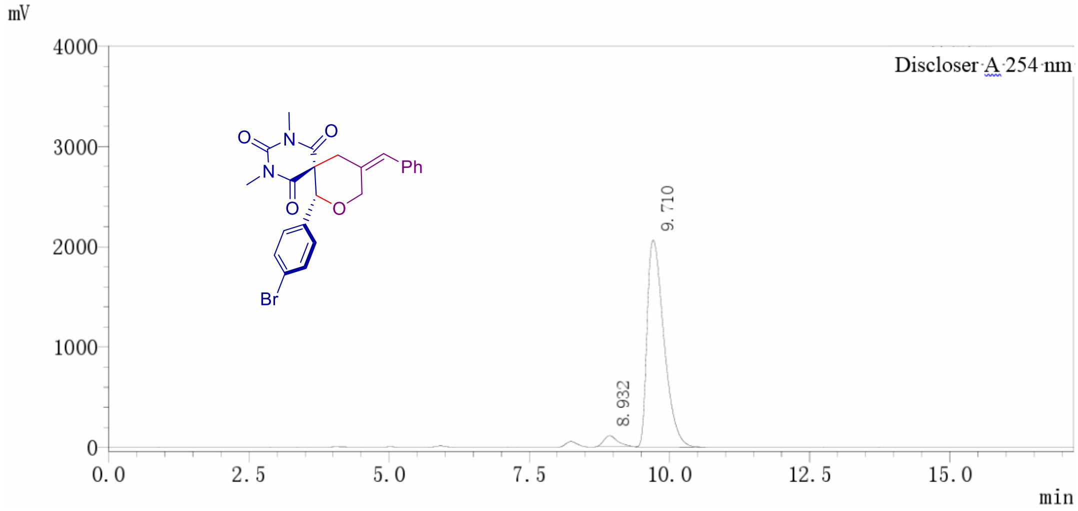

Discloser A 254 nm

Peak	Retention time (min)	Area (%)
1	8.932	3.694
2	9.710	96.306
total		100

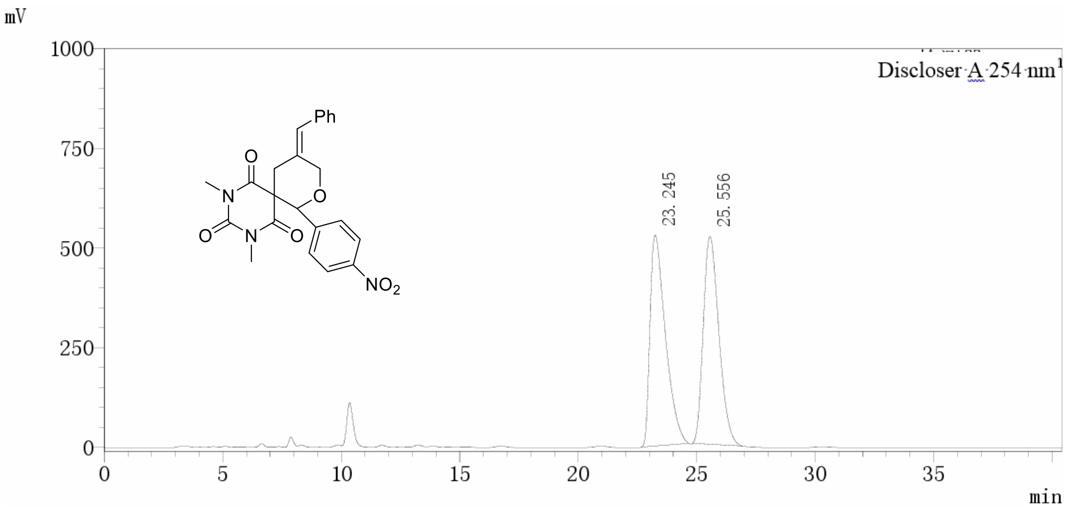

Discloser A 254 nm

Peak	Retention time (min)	Area (%)
1	23.245	49.954
2	25.556	50.046
total		100

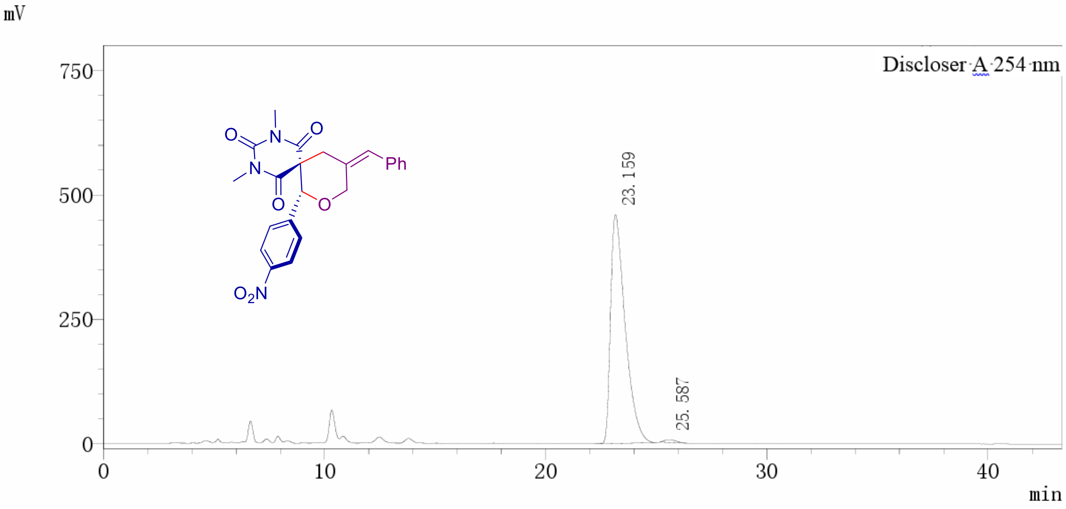

Discloser A 254 nm

Peak	Retention time (min)	Area (%)
1	23.159	99.094
2	25.587	0.906
total		100

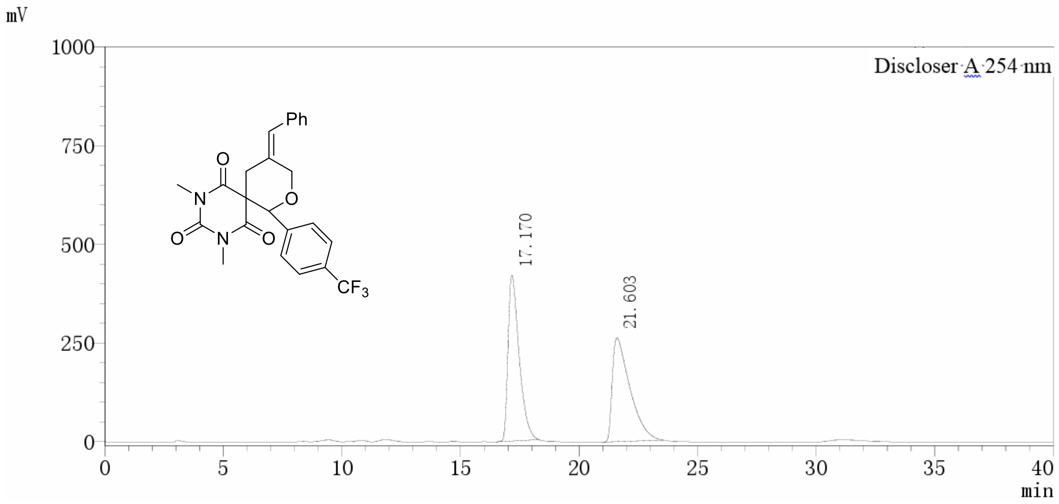

Discloser A 254 nm

Peak	Retention time (min)	Area (%)
1	17.170	50.264
2	21.603	49.736
total		100

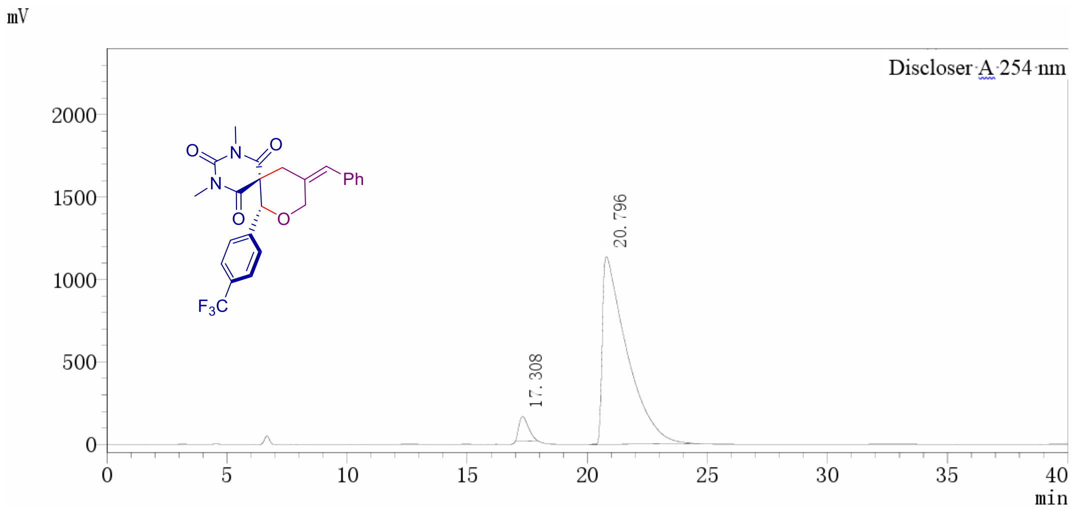

Discloser A 254 nm

Peak	Retention time (min)	Area (%)
1	17.308	4.855
2	20.796	95.145
total		100

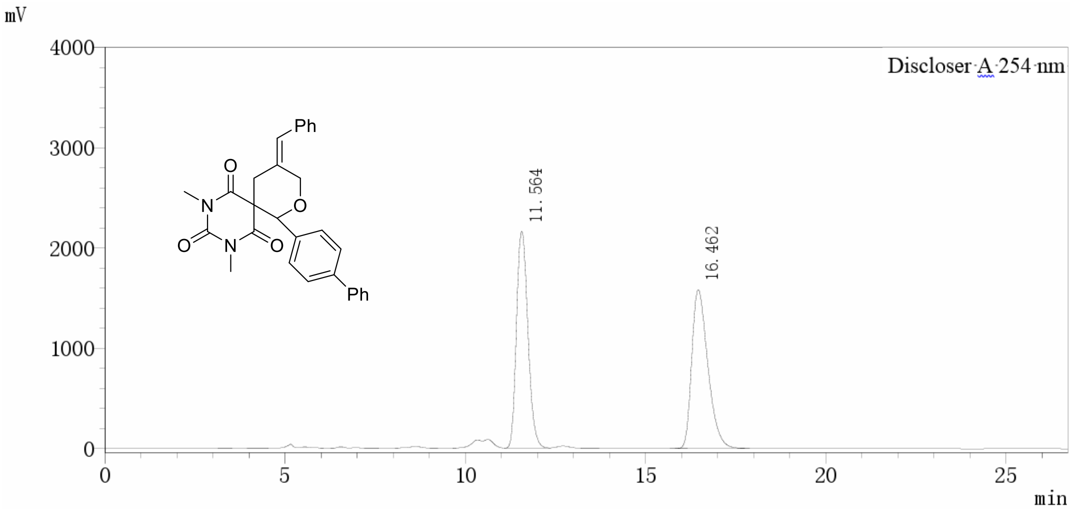

Discloser A 254 nm

Peak	Retention time (min)	Area (%)
1	11.564	49.488
2	16.462	50.512
total		100

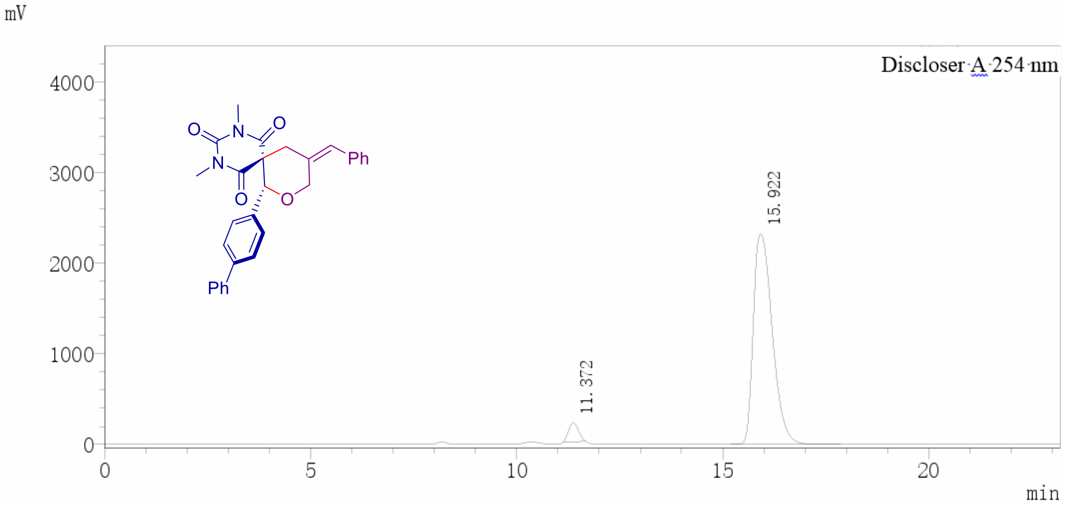

Discloser A 254 nm

Peak	Retention time (min)	Area (%)
1	11.372	4.496
2	15.922	95.504
total		100

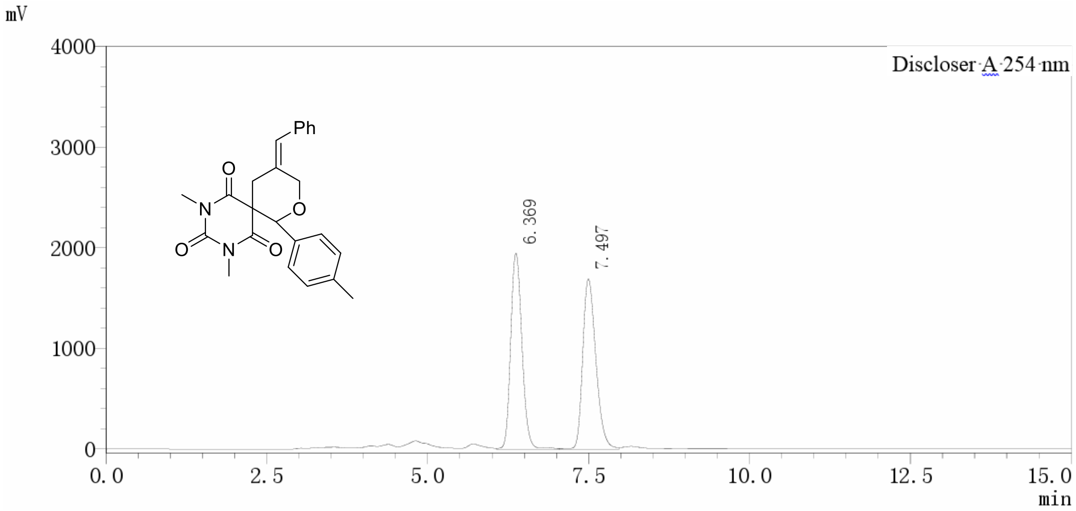

Discloser A 254 nm

Peak	Retention time (min)	Area (%)
1	6.369	49.884
2	7.497	50.116
total		100

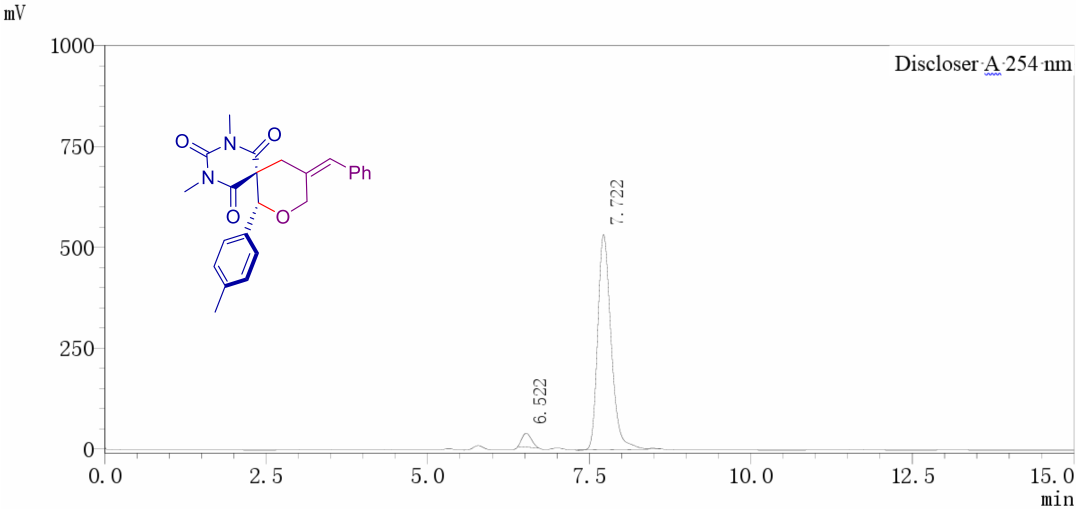

Discloser A 254 nm

Peak	Retention time (min)	Area (%)
1	6.522	4.369
2	7.722	95.631
total		100

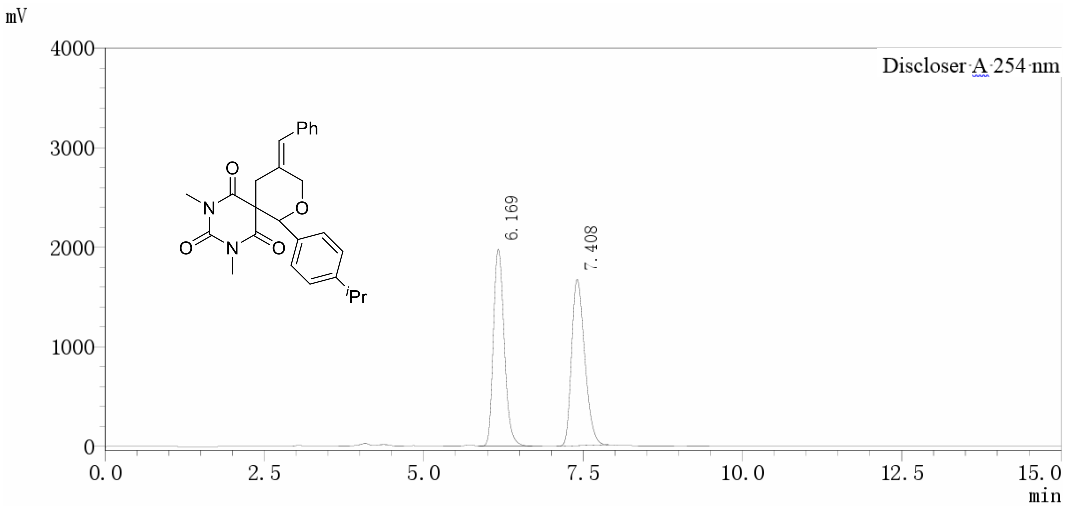

Discloser A 254 nm

Peak	Retention time (min)	Area (%)
1	6.169	49.481
2	7.408	50.519
total		100

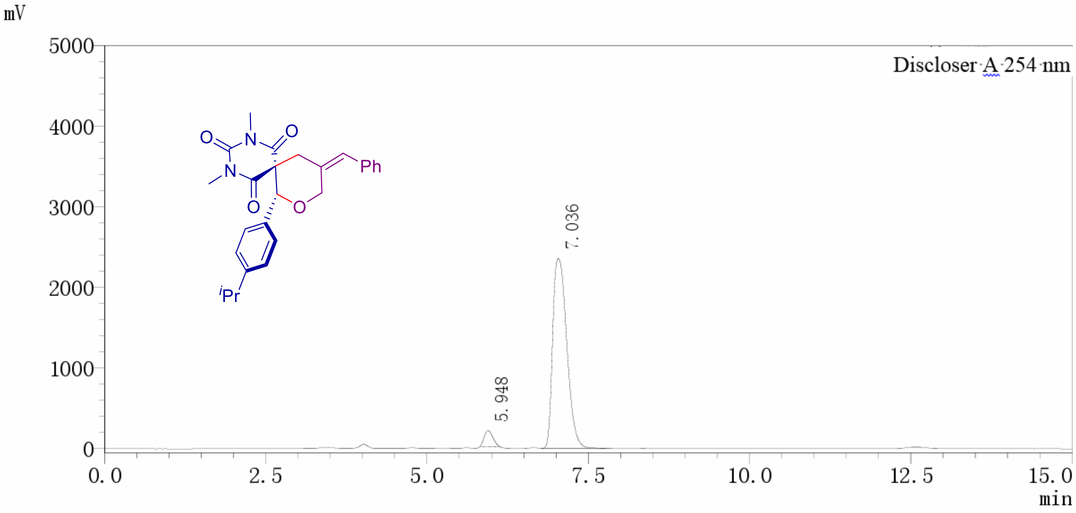

Discloser A 254 nm

Peak	Retention time (min)	Area (%)
1	5.948	4.886
2	7.036	95.134
total		100

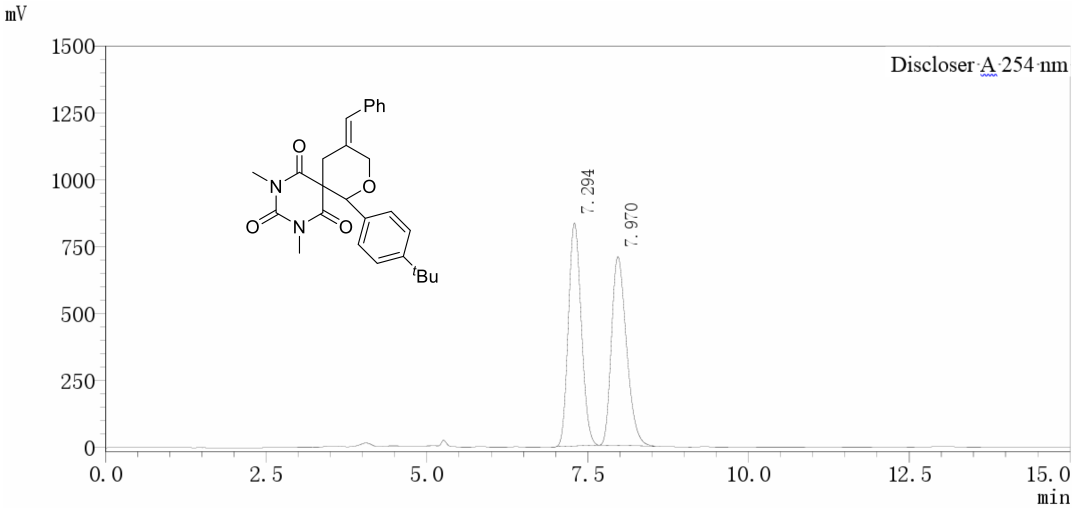

Discloser A 254 nm

Peak	Retention time (min)	Area (%)
1	7.294	50.357
2	7.970	49.643
total		100

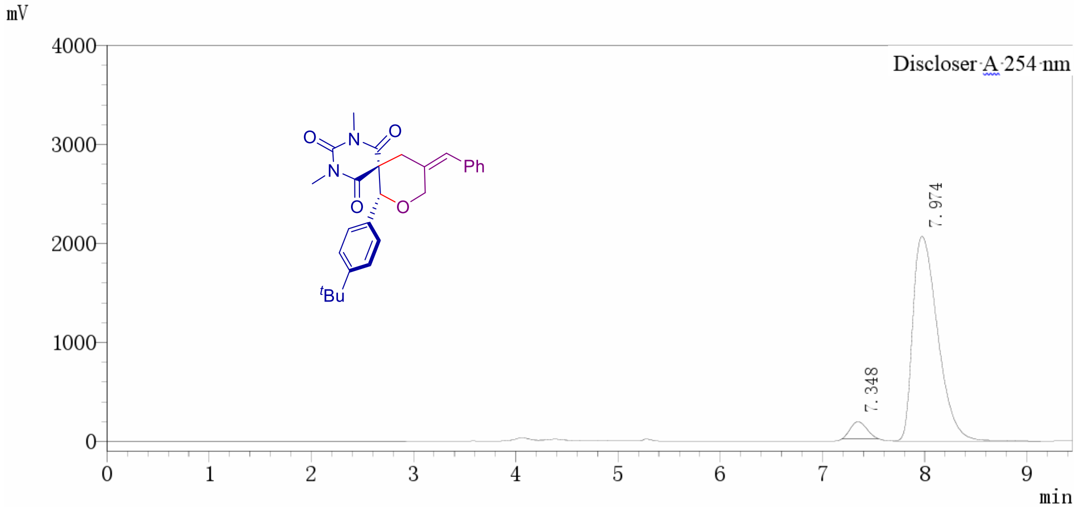

Discloser A 254 nm

Peak	Retention time (min)	Area (%)
1	7.348	5.175
2	7.974	94.825
total		100

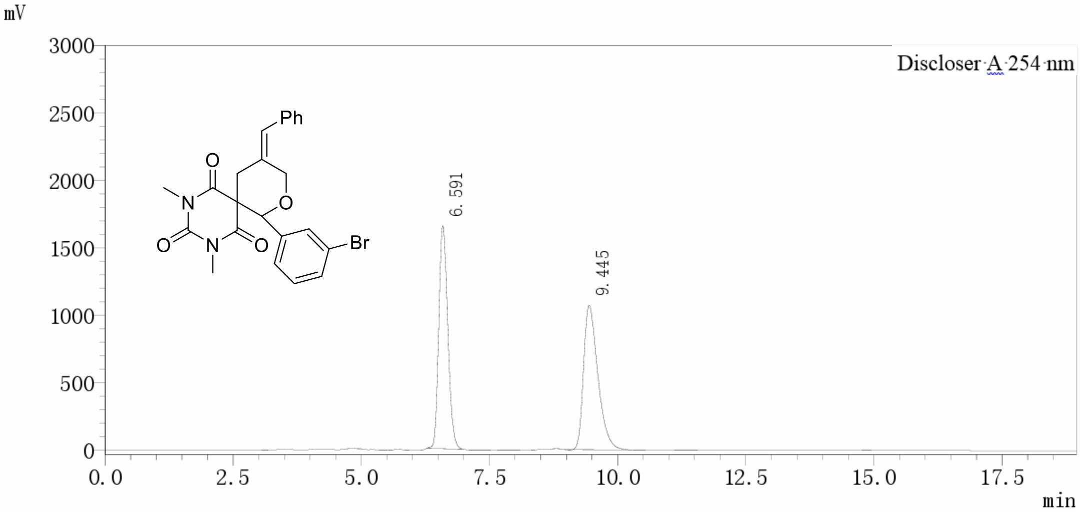

Discloser A 254 nm

Peak	Retention time (min)	Area (%)
1	6.591	49.314
2	9.445	50.686
total		100

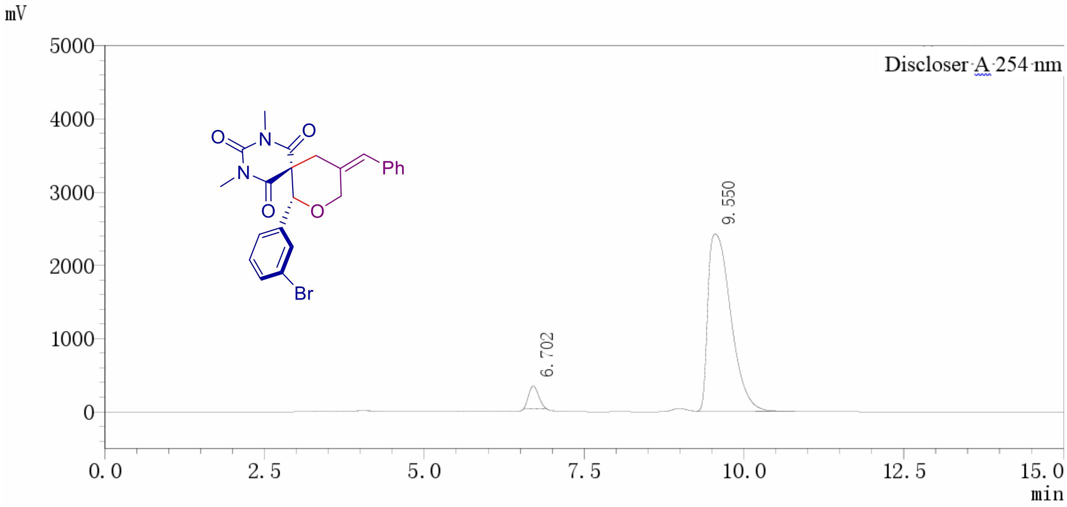

Discloser A 254 nm

Peak	Retention time (min)	Area (%)
1	6.702	5.188
2	9.550	94.812
total		100

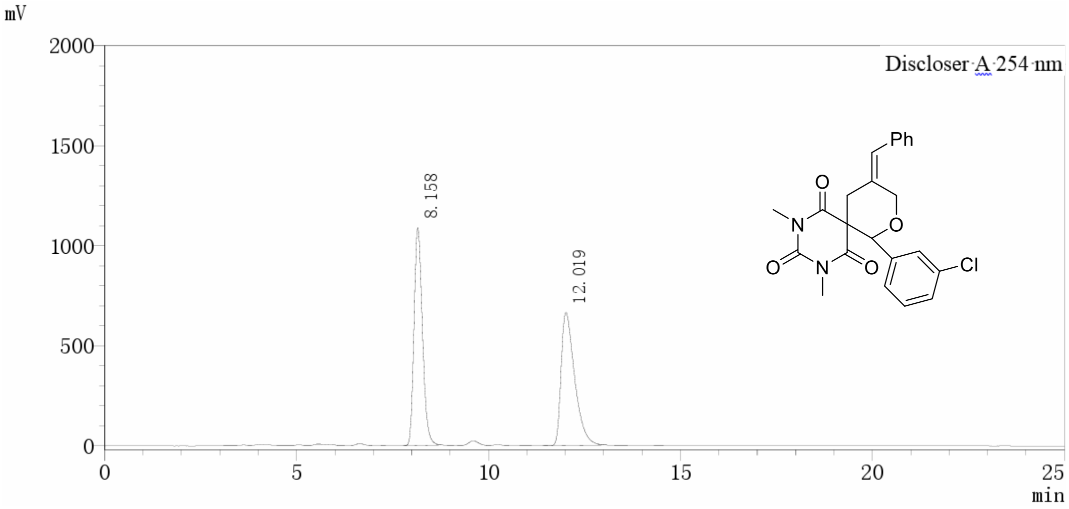

Discloser A 254 nm

Peak	Retention time (min)	Area (%)
1	8.158	50.454
2	12.019	49.546
total		100

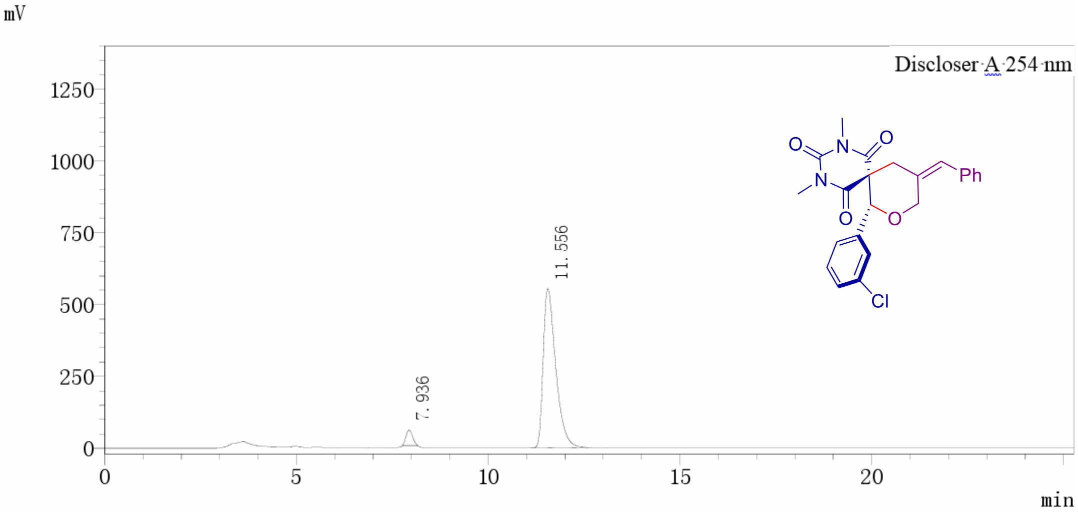

Discloser A 254 nm

Peak	Retention time (min)	Area (%)
1	7.936	4.862
2	11.556	95.138
total		100

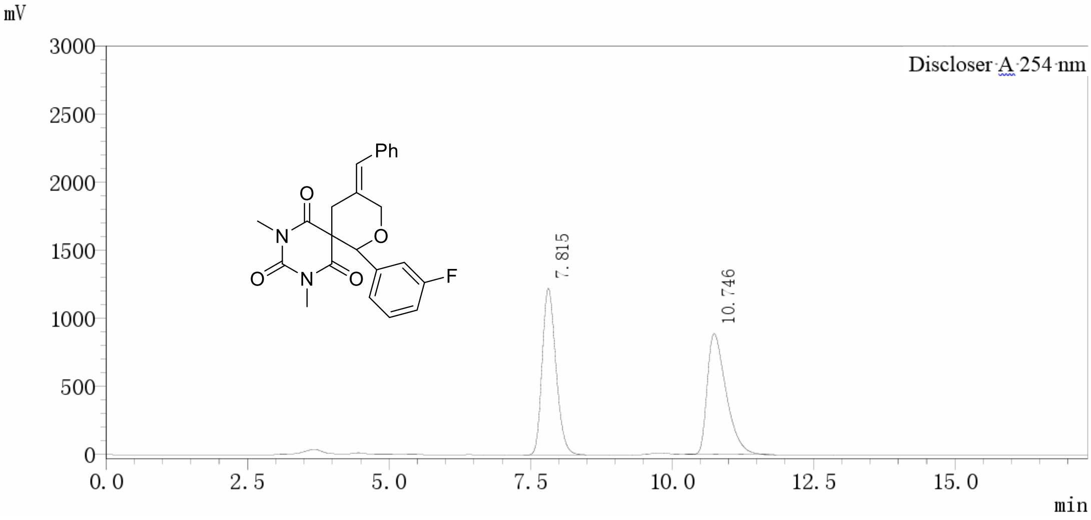

Discloser A 254 nm

Peak	Retention time (min)	Area (%)
1	7.815	50.188
2	10.746	49.812
total		100

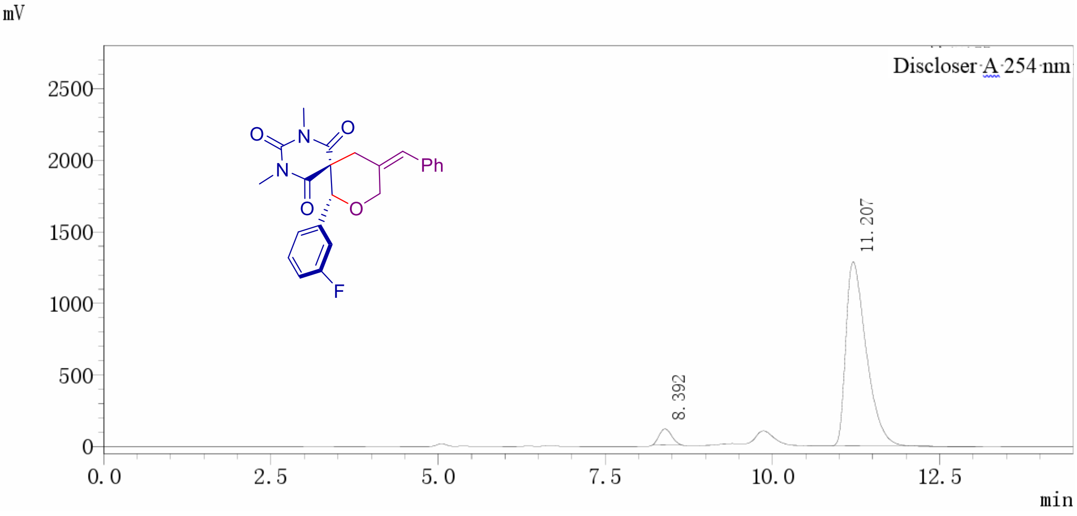

Discloser A 254 nm

Peak	Retention time (min)	Area (%)
1	8.392	5.046
2	11.207	94.954
total		100

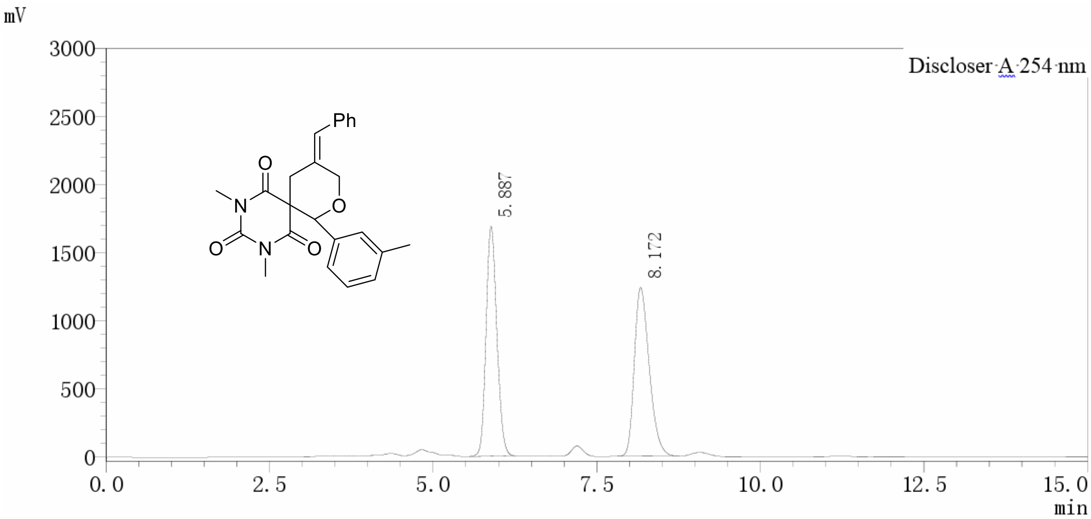

Discloser A 254 nm

Peak	Retention time (min)	Area (%)
1	5.887	49.434
2	8.172	50.566
total		100

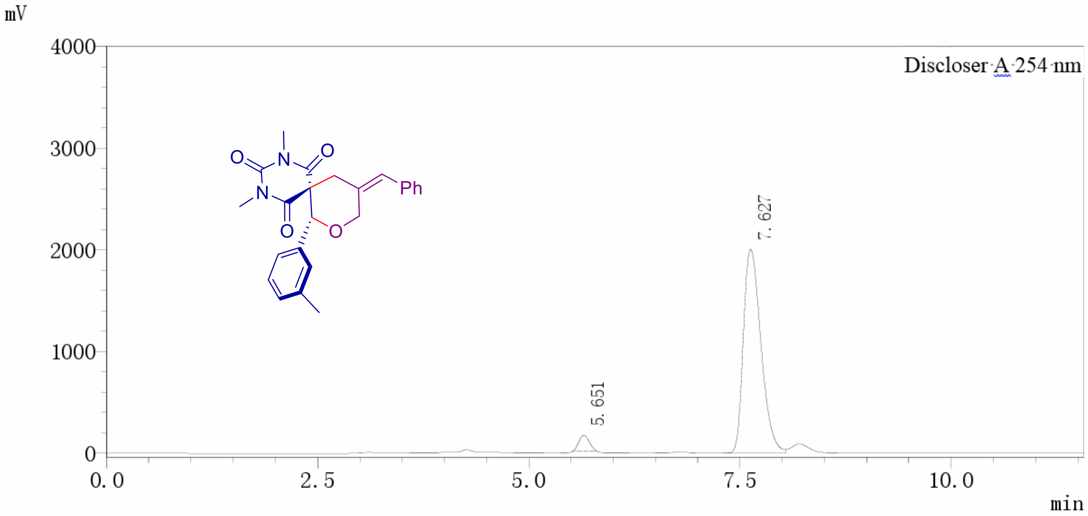

Discloser A 254 nm

Peak	Retention time (min)	Area (%)
1	5.651	4.398
2	7.627	95.602
total		100

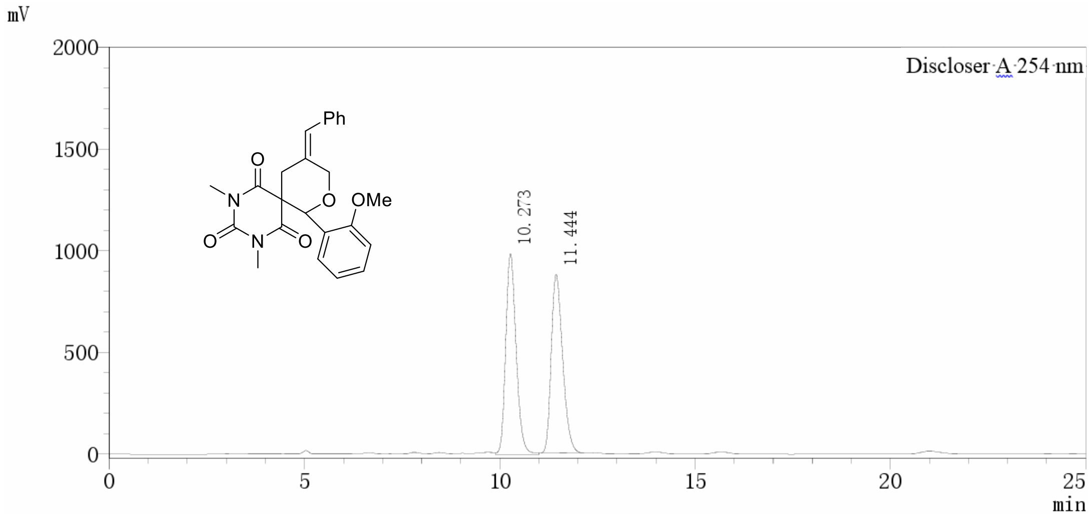

Discloser A 254 nm

Peak	Retention time (min)	Area (%)
1	10.273	50.540
2	11.444	49.460
total		100

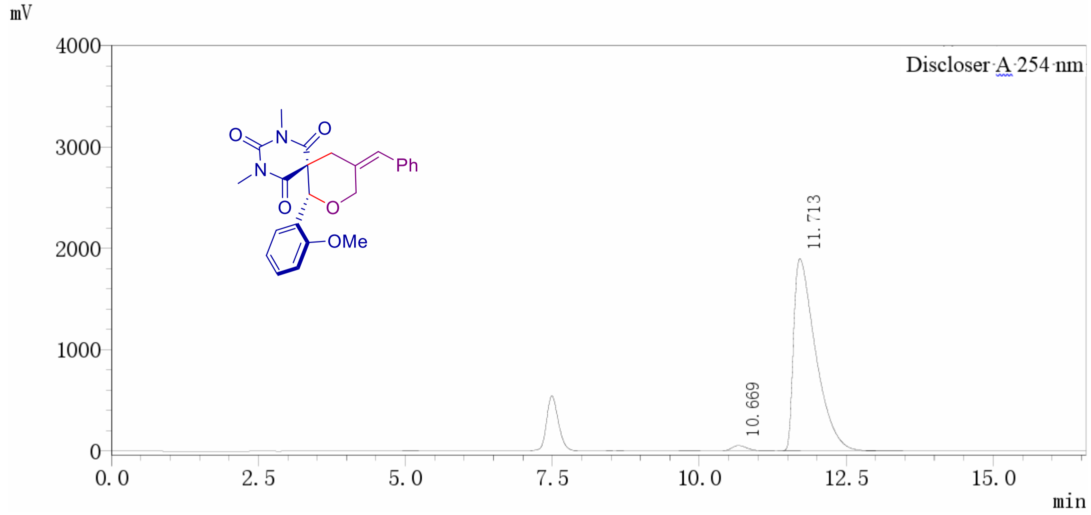

Discloser A 254 nm

Peak	Retention time (min)	Area (%)
1	10.669	1.552
2	11.713	98.448
total		100

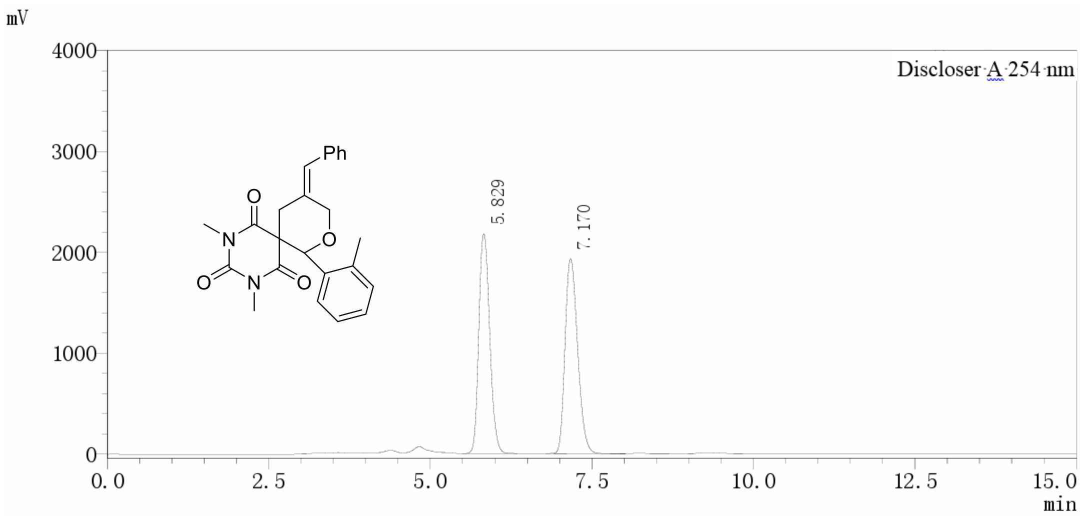

Discloser A 254 nm

Peak	Retention time (min)	Area (%)
1	5.829	49.348
2	7.170	50.652
total		100

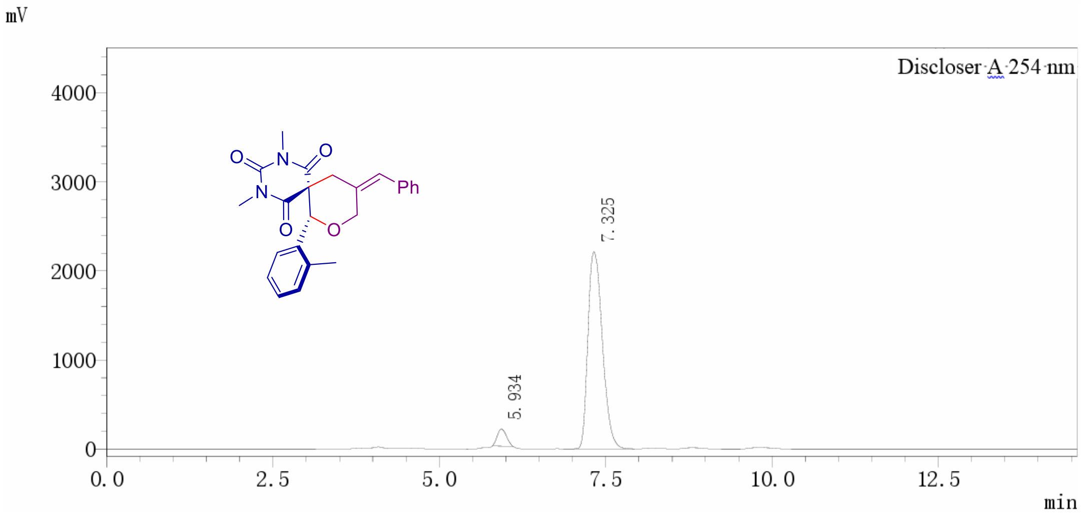

Discloser A 254 nm

Peak	Retention time (min)	Area (%)
1	5.934	5.205
2	7.325	94.795
total		100

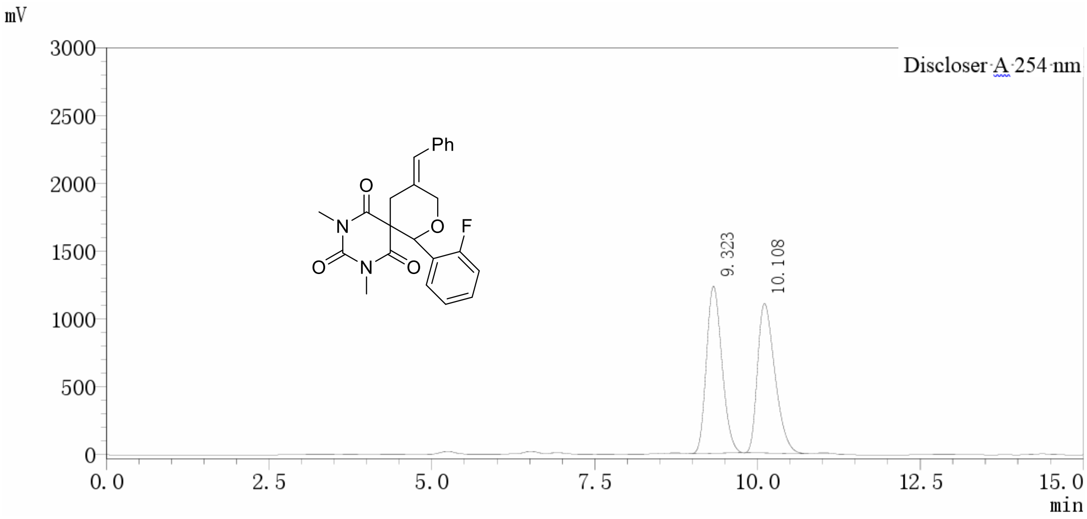

Discloser A 254 nm

Peak	Retention time (min)	Area (%)
1	9.323	49.861
2	10.108	50.139
total		100

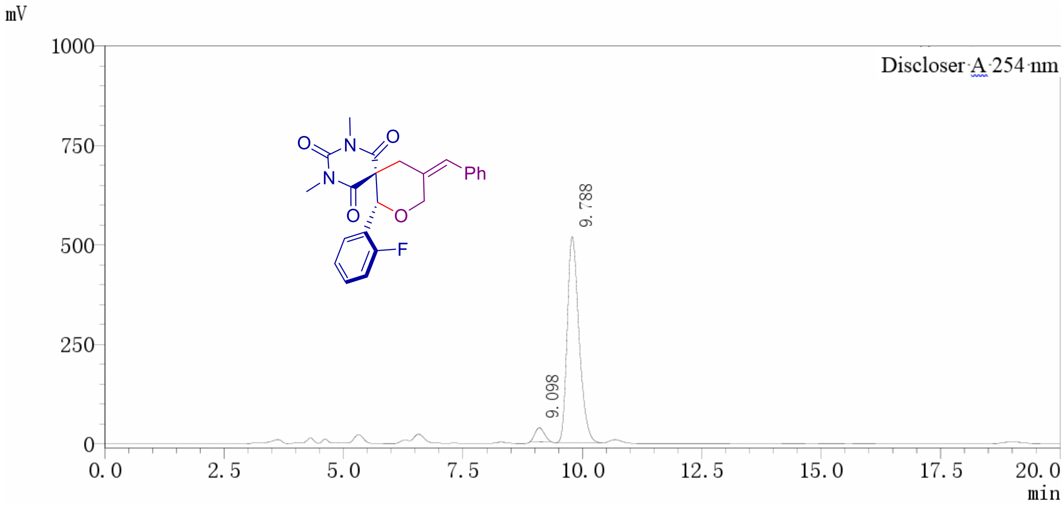

Discloser A 254 nm

Peak	Retention time (min)	Area (%)
1	9.098	5.017
2	9.788	94.983
total		100

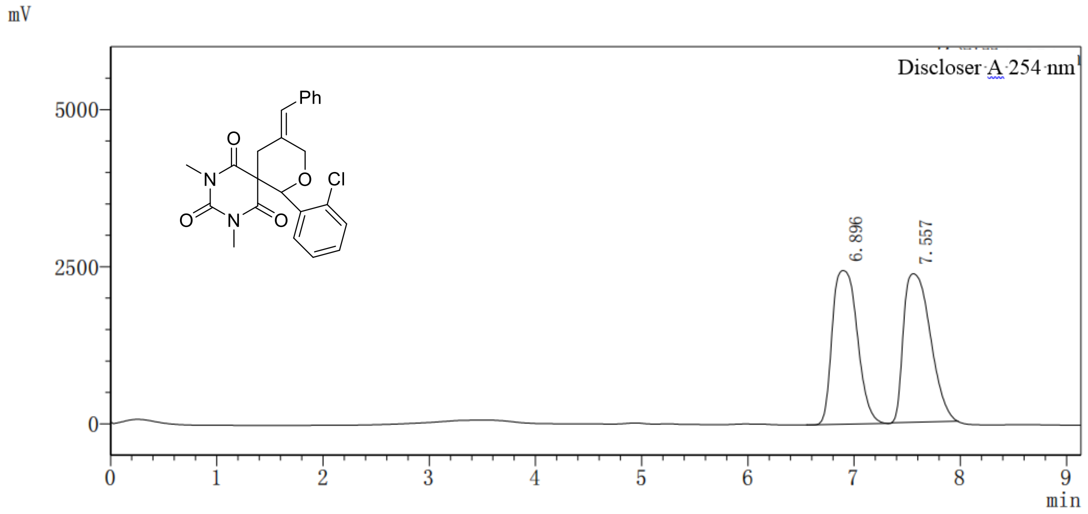

Discloser A 254 nm

Peak	Retention time (min)	Area (%)
1	6.896	49.017
2	7.557	50.983
total		100

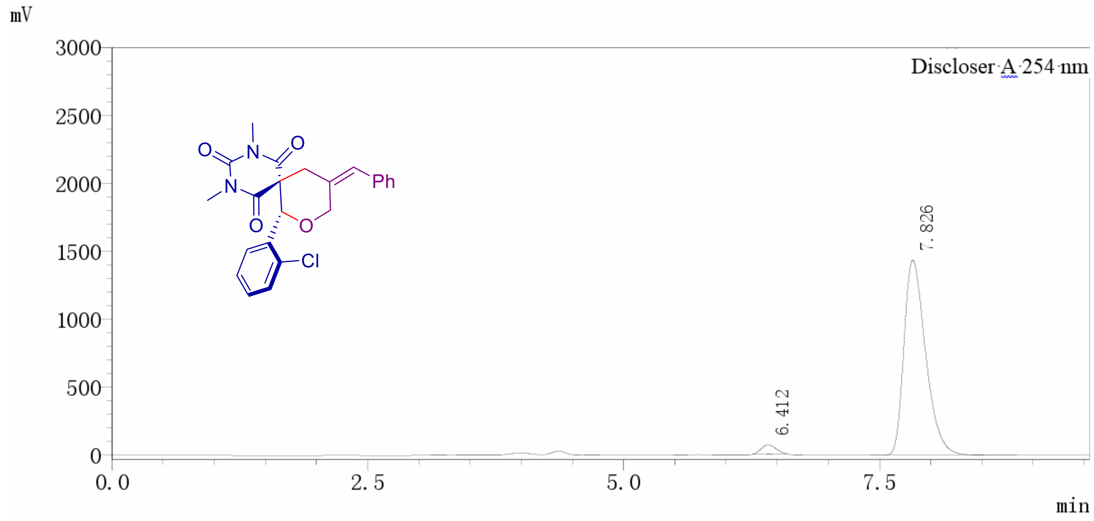

Discloser A 254 nm

Peak	Retention time (min)	Area (%)
1	6.412	3.184
2	7.826	96.816
total		100

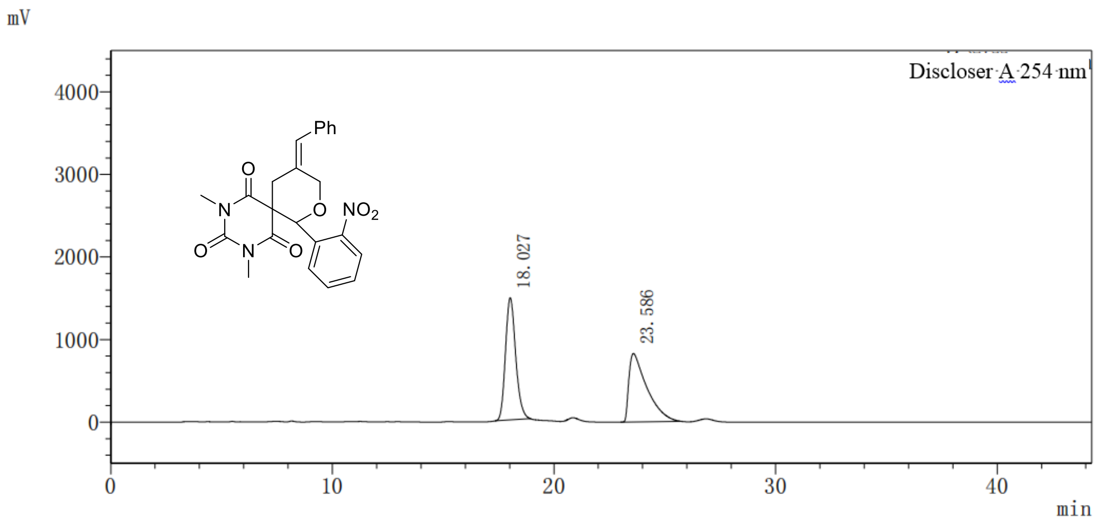

Discloser A 254 nm

Peak	Retention time (min)	Area (%)
1	18.027	50.436
2	23.586	49.564
total		100

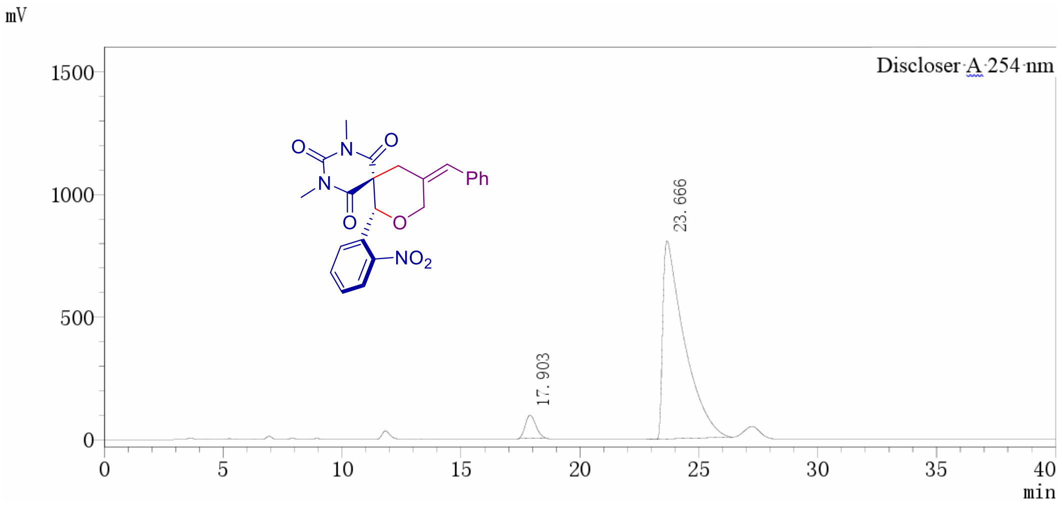

Discloser A 254 nm

Peak	Retention time (min)	Area (%)
1	17.903	4.968
2	23.666	95.032
total		100

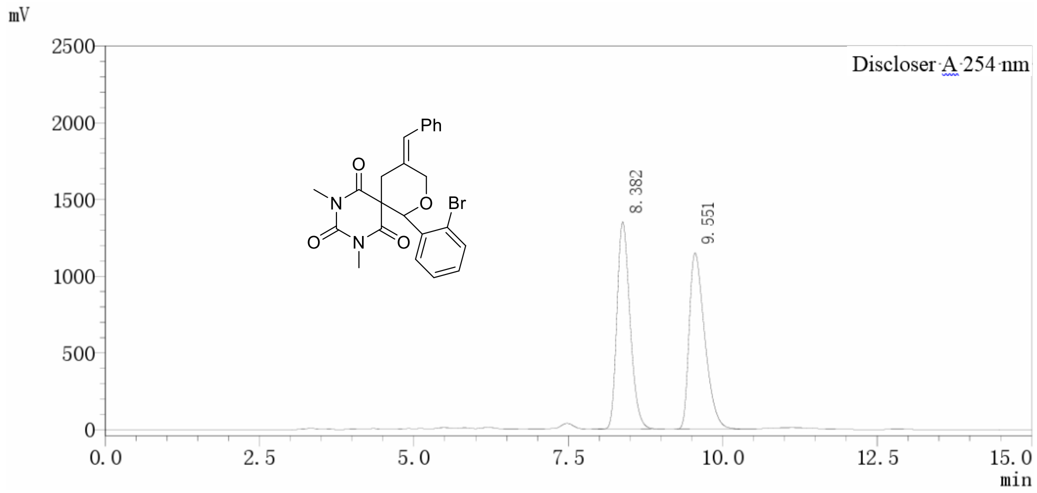

Discloser A 254 nm

Peak	Retention time (min)	Area (%)
1	8.382	49.601
2	9.551	50.399
total		100

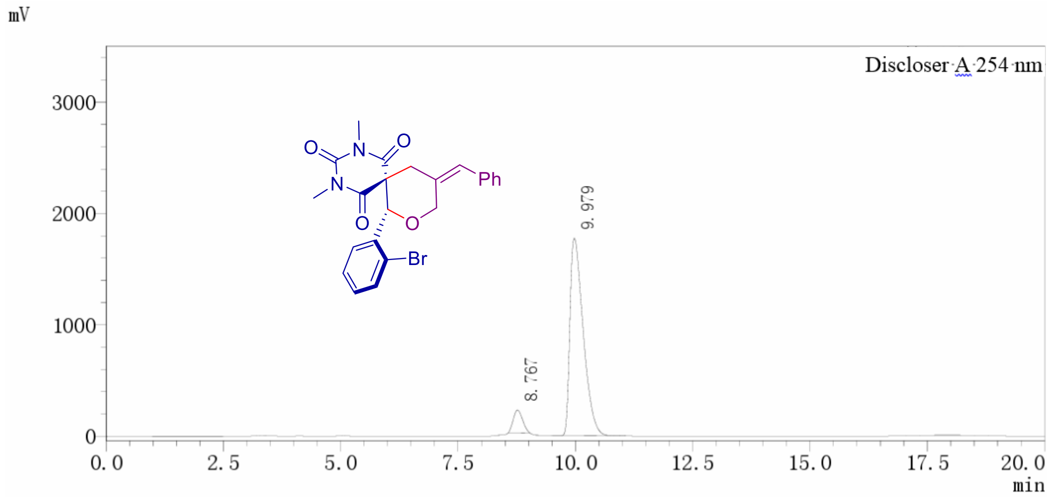

Discloser A 254 nm

Peak	Retention time (min)	Area (%)
1	8.767	7.585
2	9.979	92.415
total		100

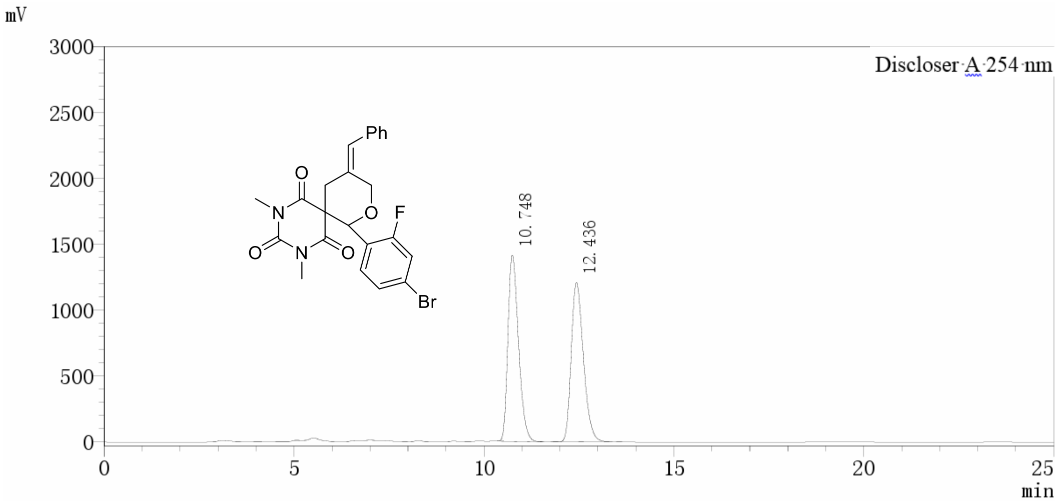

Discloser A 254 nm

Peak	Retention time (min)	Area (%)
1	10.748	50.395
2	12.436	49.605
total		100

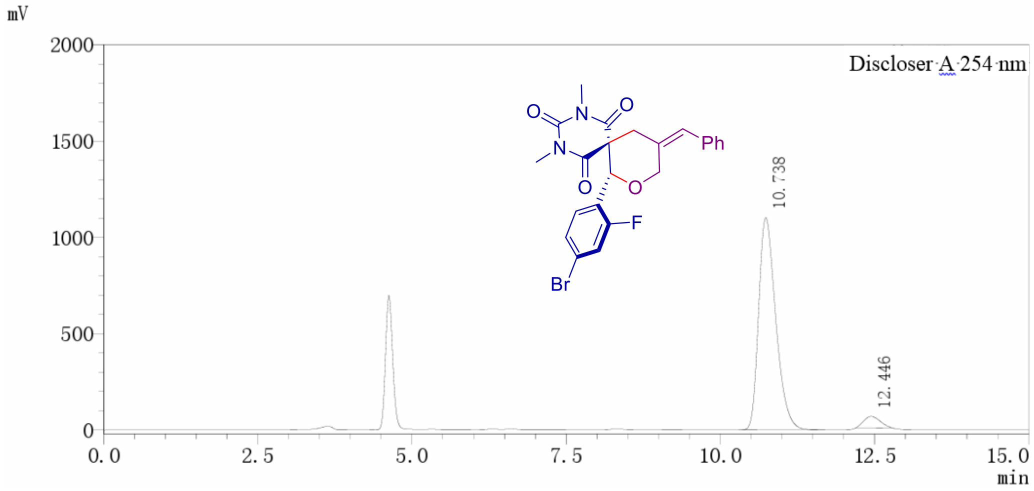

Discloser A 254 nm

Peak	Retention time (min)	Area (%)
1	10.738	94.887
2	12.446	5.113
total		100

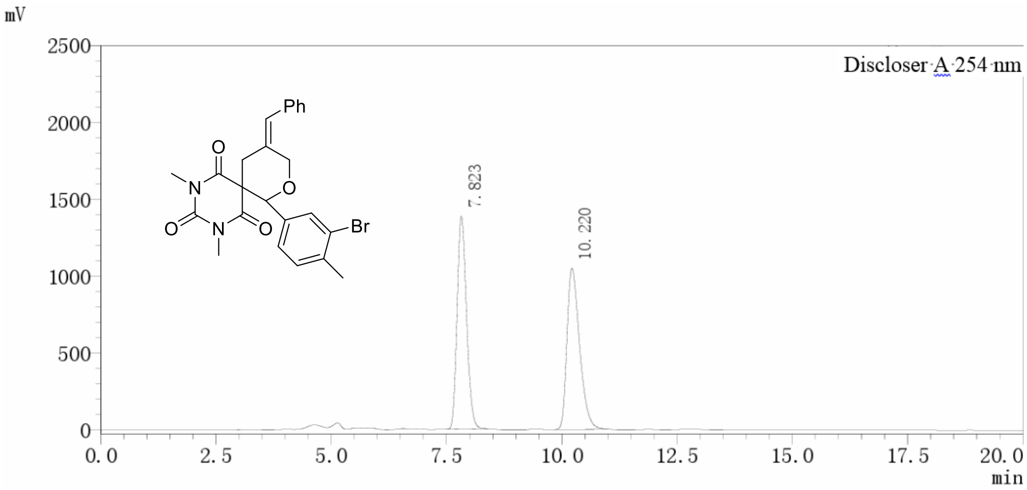

Discloser A 254 nm

Peak	Retention time (min)	Area (%)
1	7.823	49.696
2	10.220	50.304
total		100

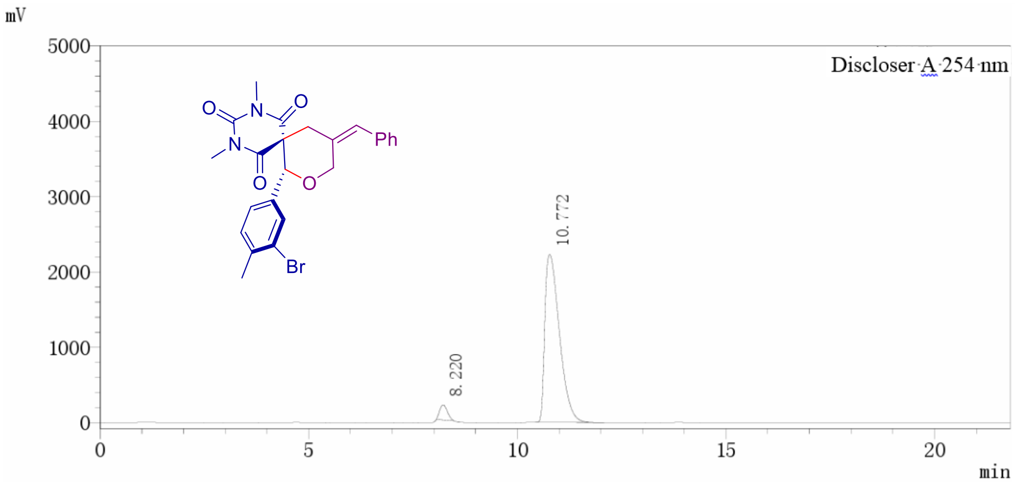

Discloser A 254 nm

Peak	Retention time (min)	Area (%)
1	8.220	4.491
2	10.772	95.509
total		100

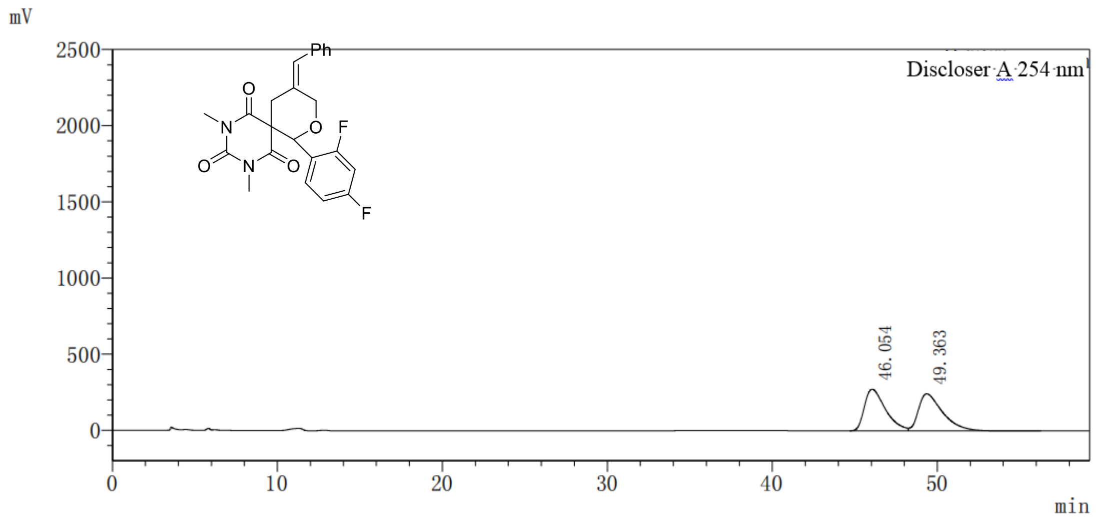

Discloser A 254 nm

Peak	Retention time (min)	Area (%)
1	46.054	49.372
2	49.363	50.628
total		100

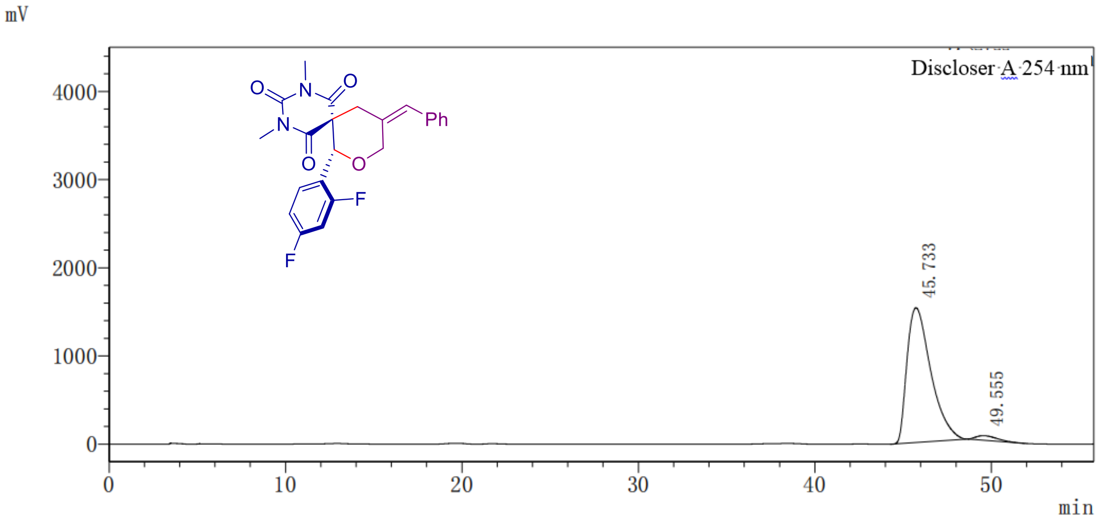

Discloser A 254 nm

Peak	Retention time (min)	Area (%)
1	45.733	97.028
2	49.555	2.972
total		100

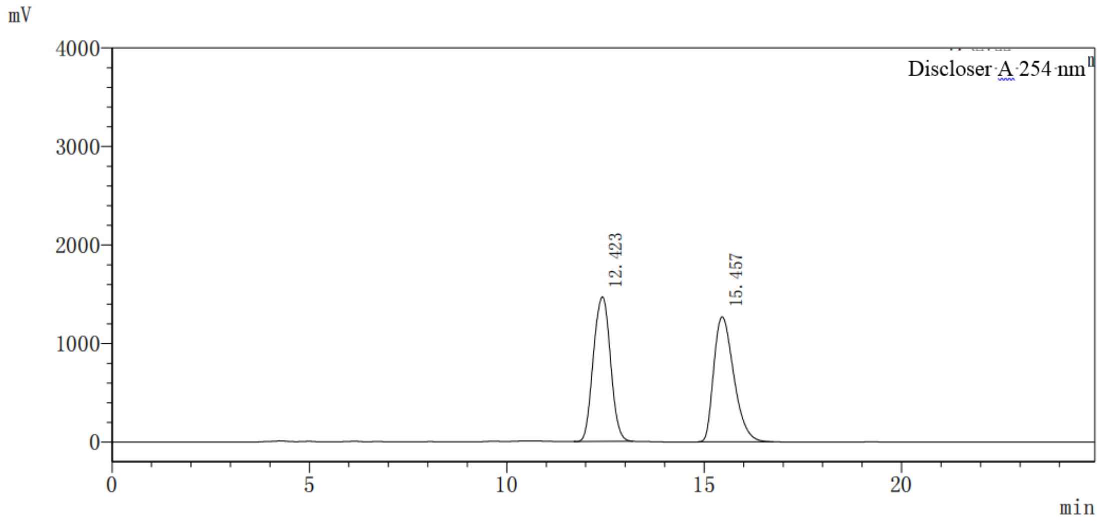

Discloser A 254 nm

Peak	Retention time (min)	Area (%)
1	12.423	49.699
2	15.457	50.301
total		100

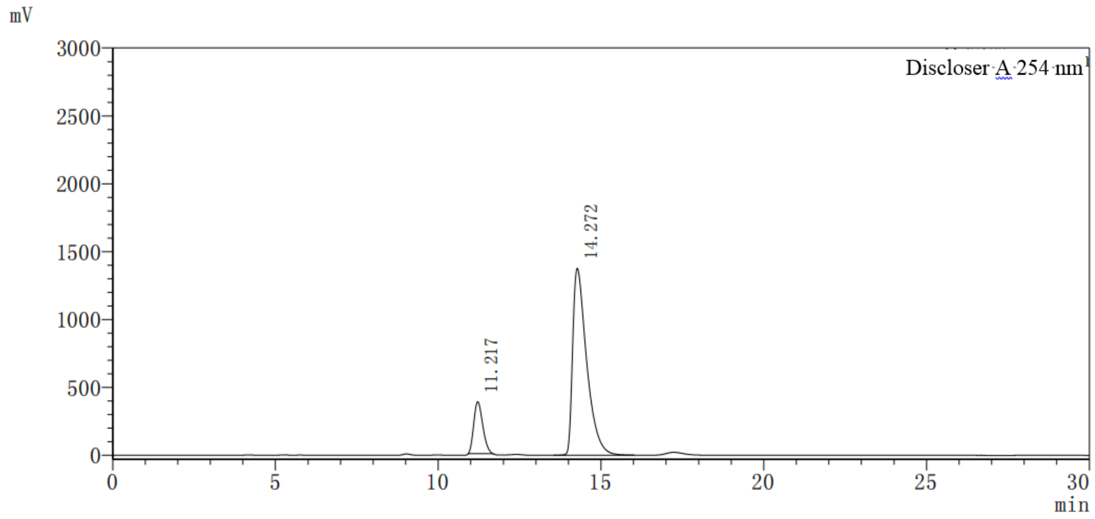

Discloser A 254 nm

Peak	Retention time (min)	Area (%)
1	11.217	15.164
2	14.272	84.836
total		100

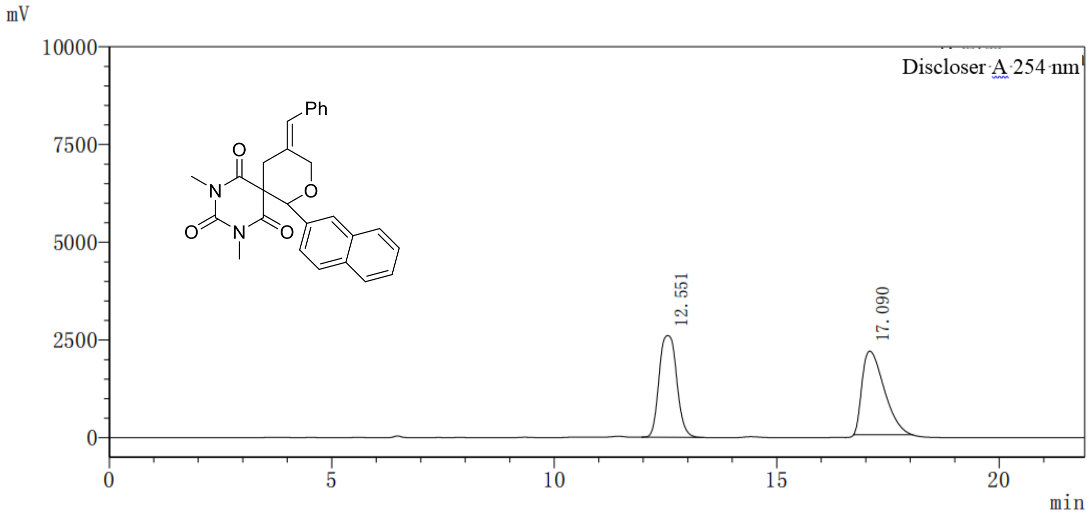

Discloser A 254 nm

Peak	Retention time (min)	Area (%)
1	12.551	49.076
2	17.090	50.924
total		100

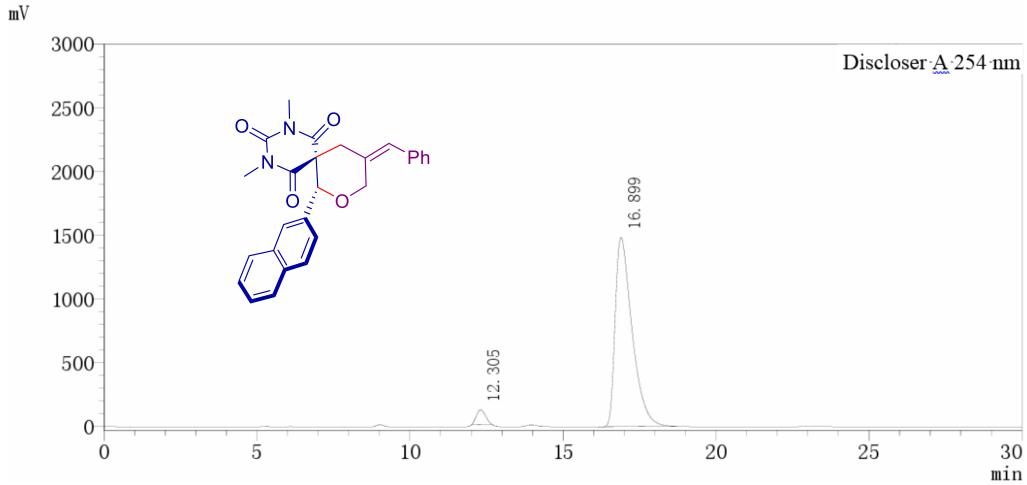

Discloser A 254 nm

Peak	Retention time (min)	Area (%)
1	12.305	4.089
2	16.899	95.911
total		100

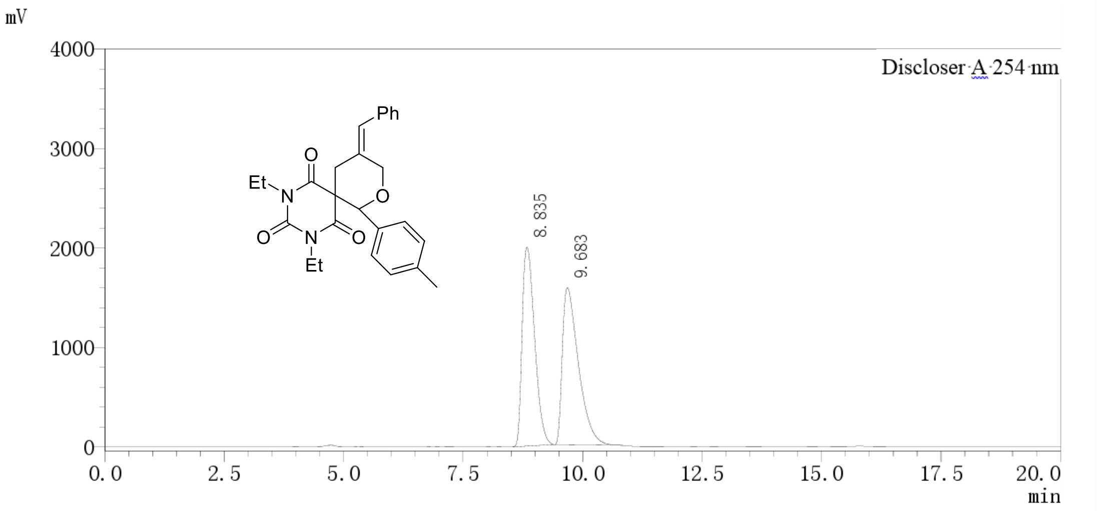

Discloser A 254 nm

Peak	Retention time (min)	Area (%)
1	8.835	49.519
2	9.683	50.481
total		100

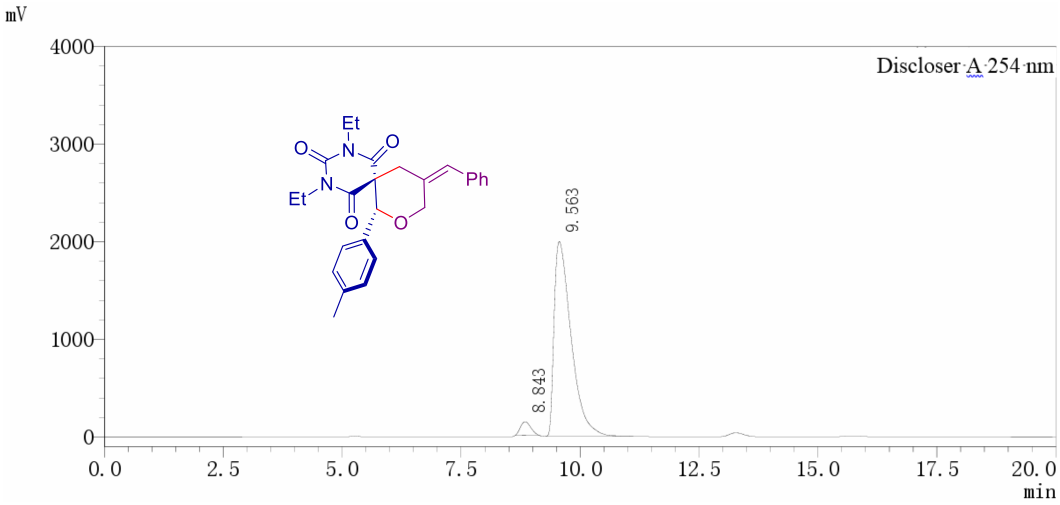

Discloser A 254 nm

Peak	Retention time (min)	Area (%)
1	8.843	4.093
2	9.563	95.907
total		100

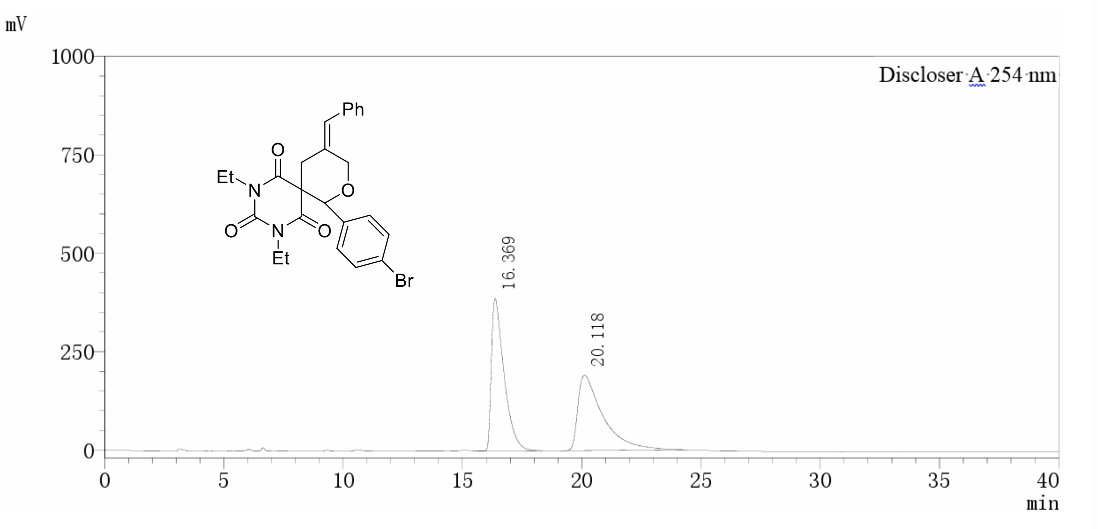

Discloser A 254 nm

Peak	Retention time (min)	Area (%)
1	16.369	50.400
2	20.118	49.600
total		100

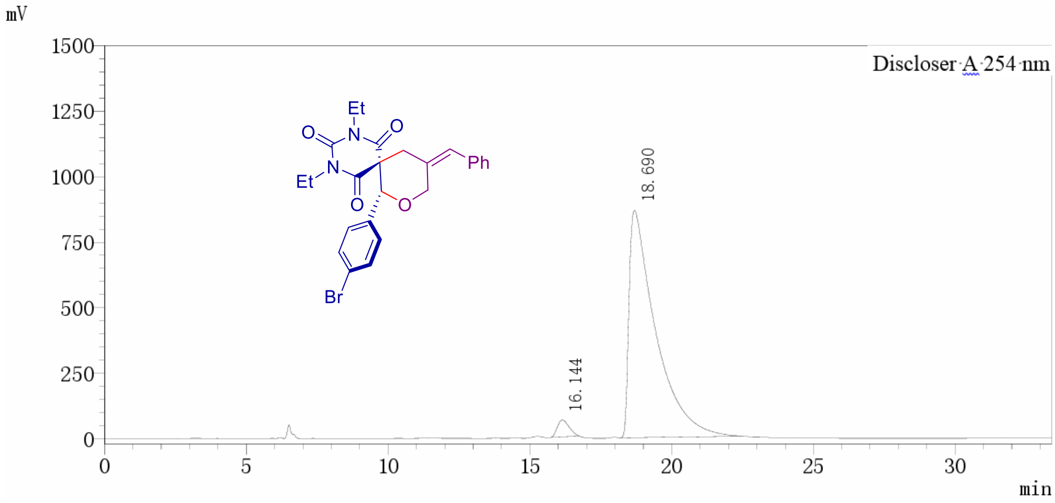

Discloser A 254 nm

Peak	Retention time (min)	Area (%)
1	16.144	3.151
2	18.690	96.849
total		100

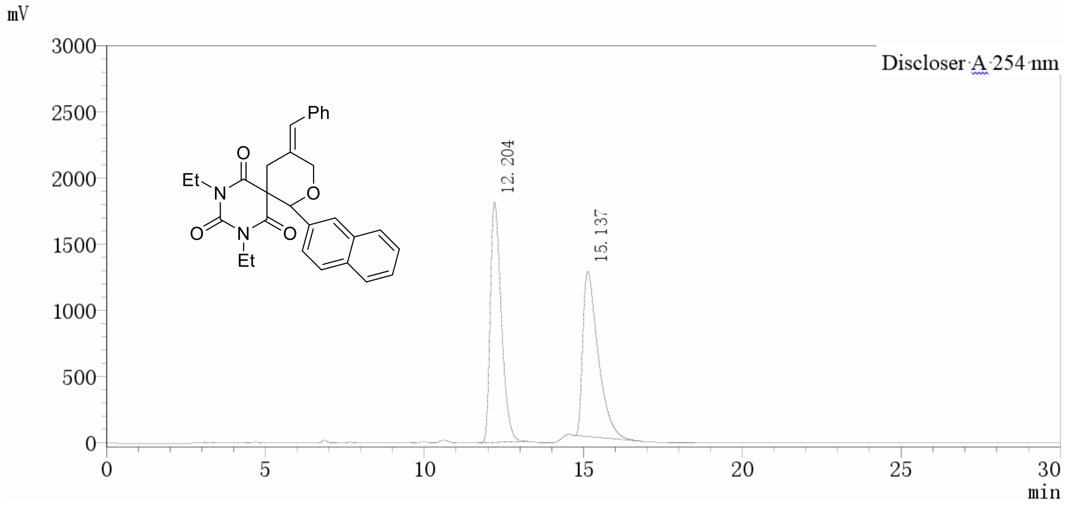

Discloser A 254 nm

Peak	Retention time (min)	Area (%)
1	12.204	50.857
2	15.137	49.143
total		100

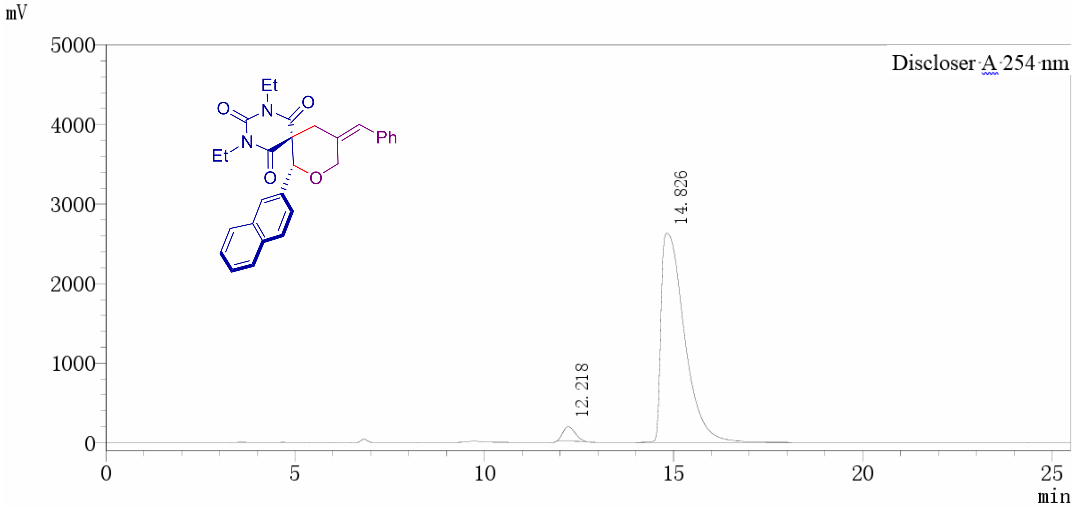

Discloser A 254 nm

Peak	Retention time (min)	Area (%)
1	12.218	3.550
2	14.826	96.450
total		100

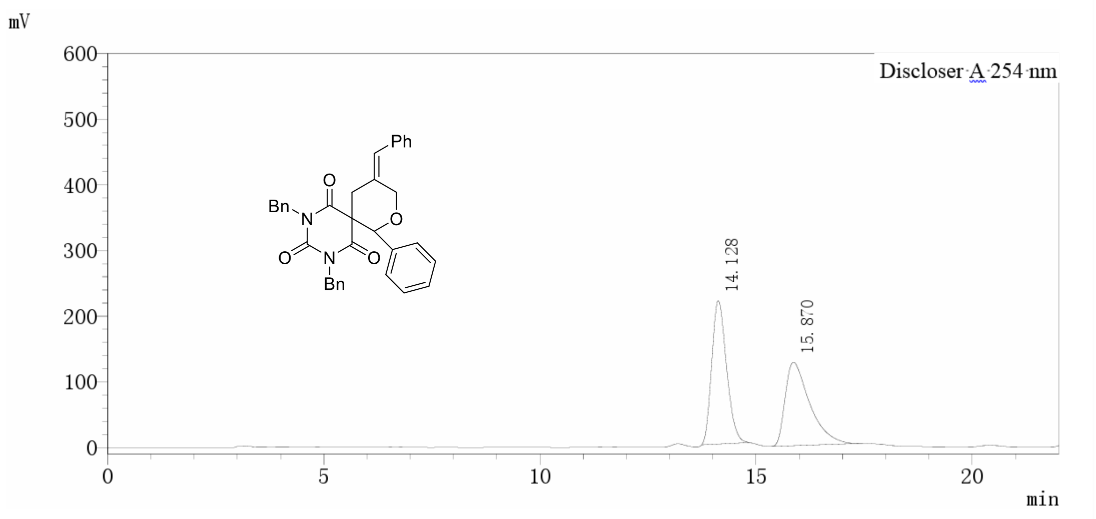

Discloser A 254 nm

Peak	Retention time (min)	Area (%)
1	14.128	50.895
2	15.870	49.105
total		100

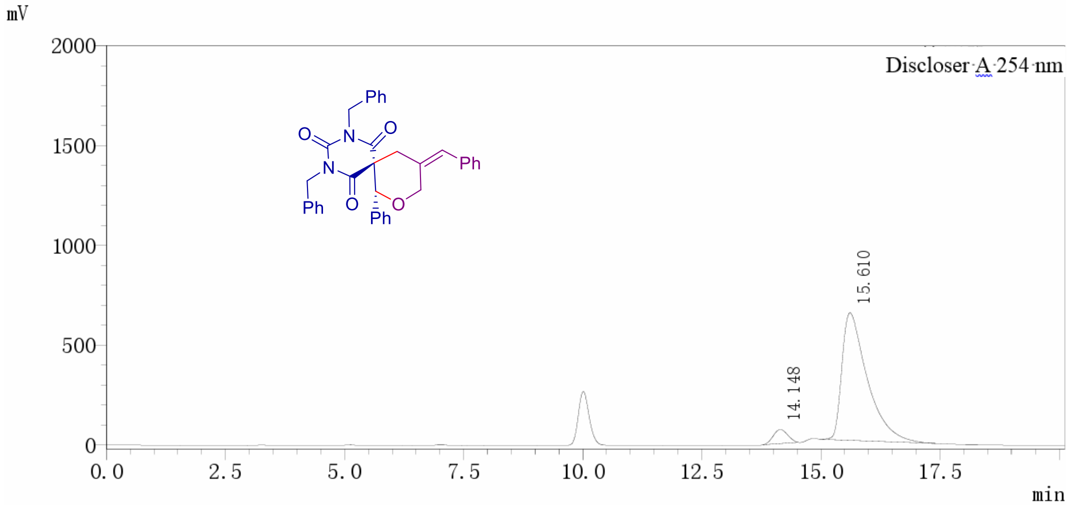

Discloser A 254 nm

Peak	Retention time (min)	Area (%)
1	14.148	5.886
2	15.610	94.114
total		100

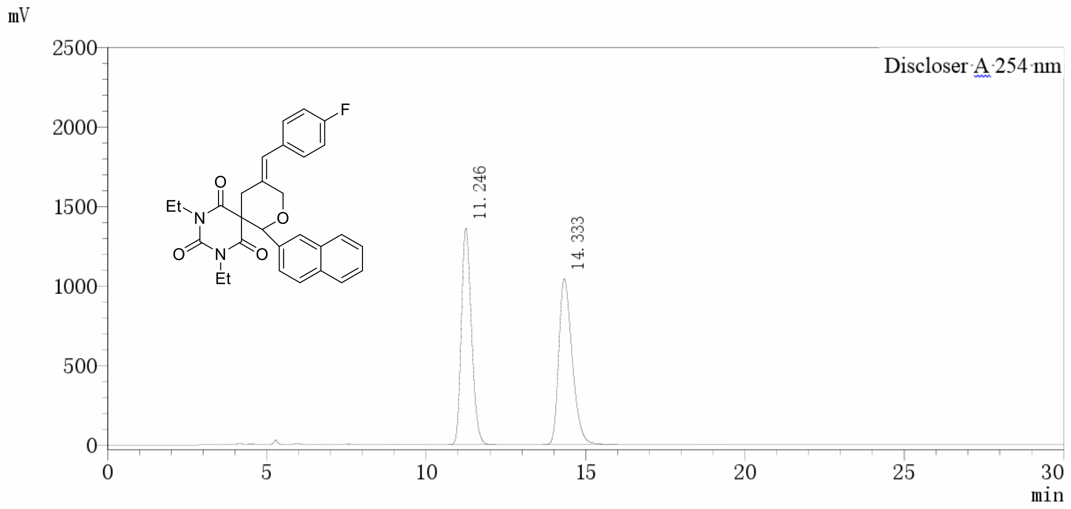

Discloser A 254 nm

Peak	Retention time (min)	Area (%)
1	11.246	49.515
2	14.333	50.485
total		100

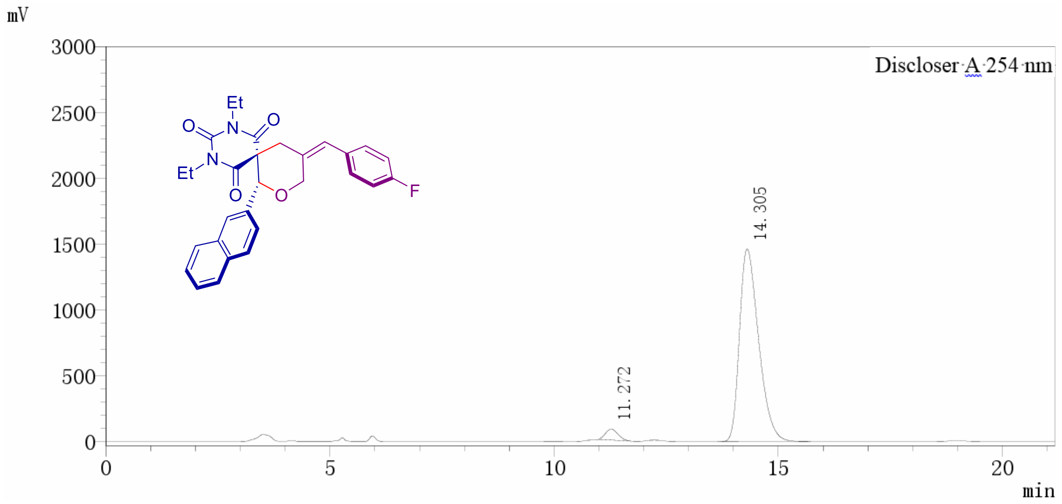

Discloser A 254 nm

Peak	Retention time (min)	Area (%)
1	11.272	3.552
2	14.305	96.448
total		100

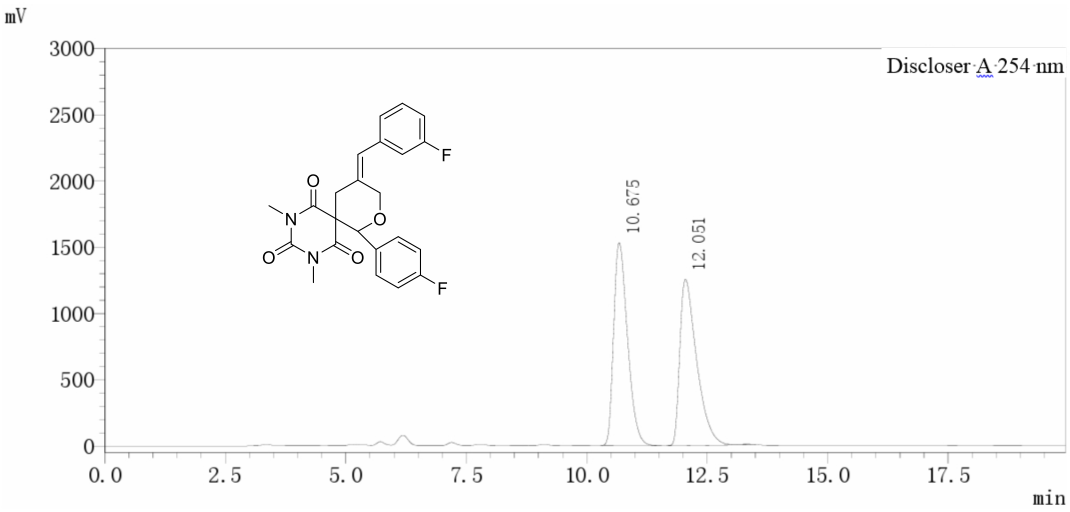

Discloser A 254 nm

Peak	Retention time (min)	Area (%)
1	10.675	49.913
2	12.051	50.087
total		100

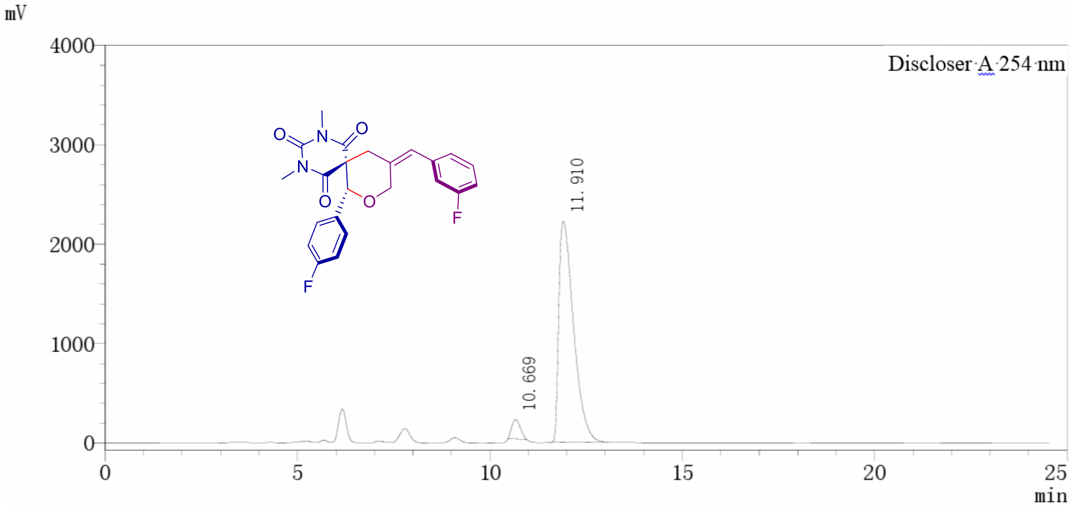

Discloser A 254 nm

Peak	Retention time (min)	Area (%)
1	10.669	4.873
2	11.910	95.127
total		100

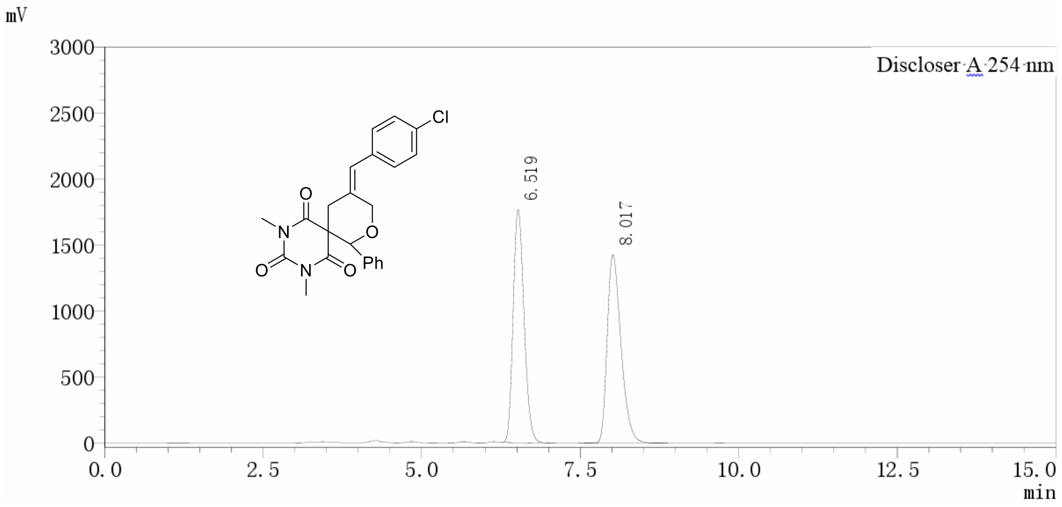

Discloser A 254 nm

Peak	Retention time (min)	Area (%)
1	6.519	49.458
2	8.017	50.542
total		100

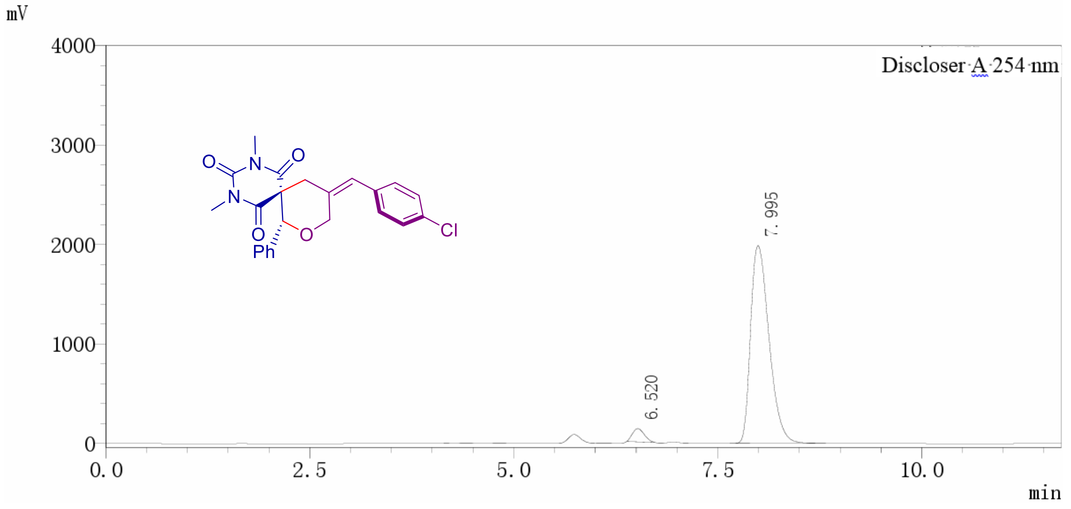

Discloser A 254 nm

Peak	Retention time (min)	Area (%)
1	6.520	4.141
2	7.995	95.859
total		100

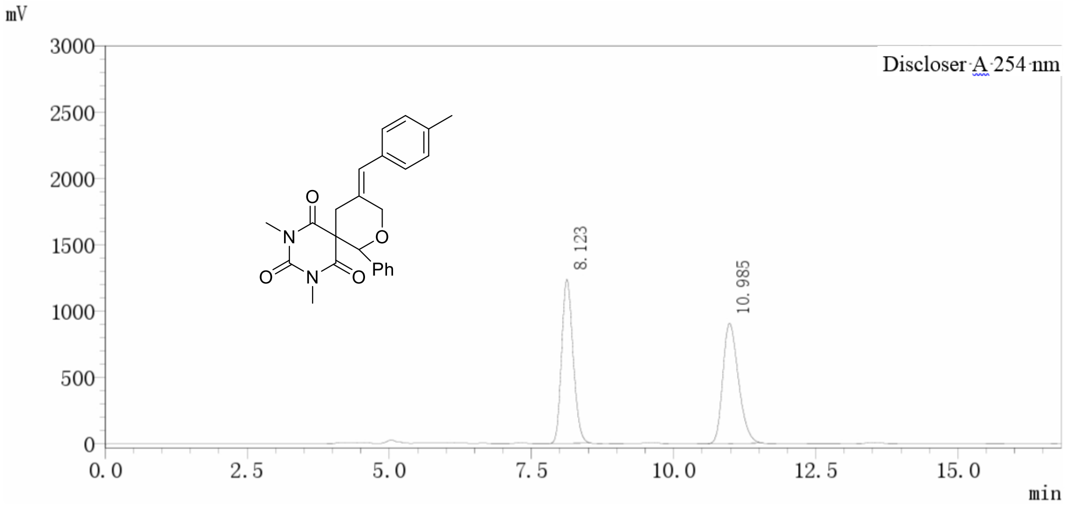

Discloser A 254 nm

Peak	Retention time (min)	Area (%)
1	8.123	50.051
2	10.985	49.949
total		100

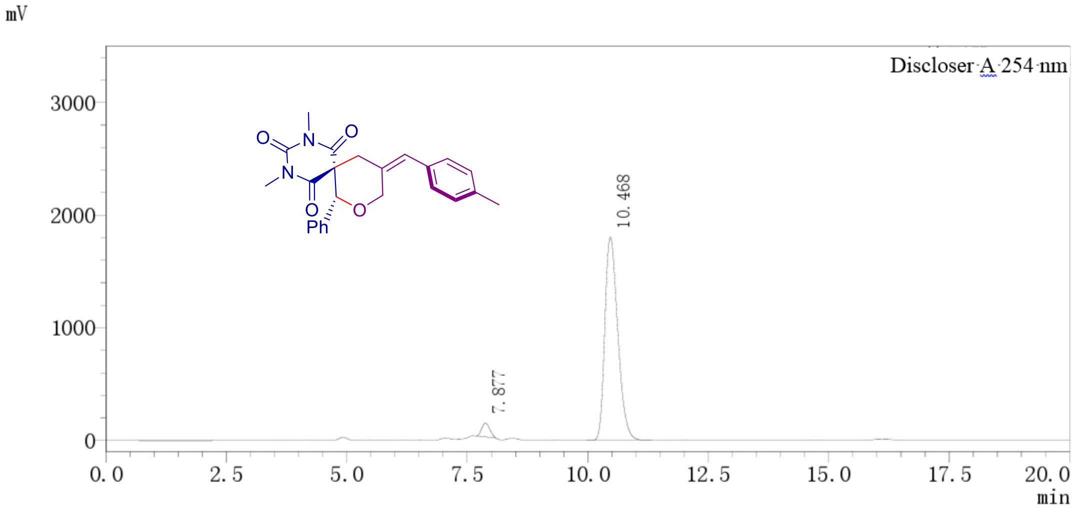

Discloser A 254 nm

Peak	Retention time (min)	Area (%)
1	7.877	4.000
2	10.468	96.000
total		100

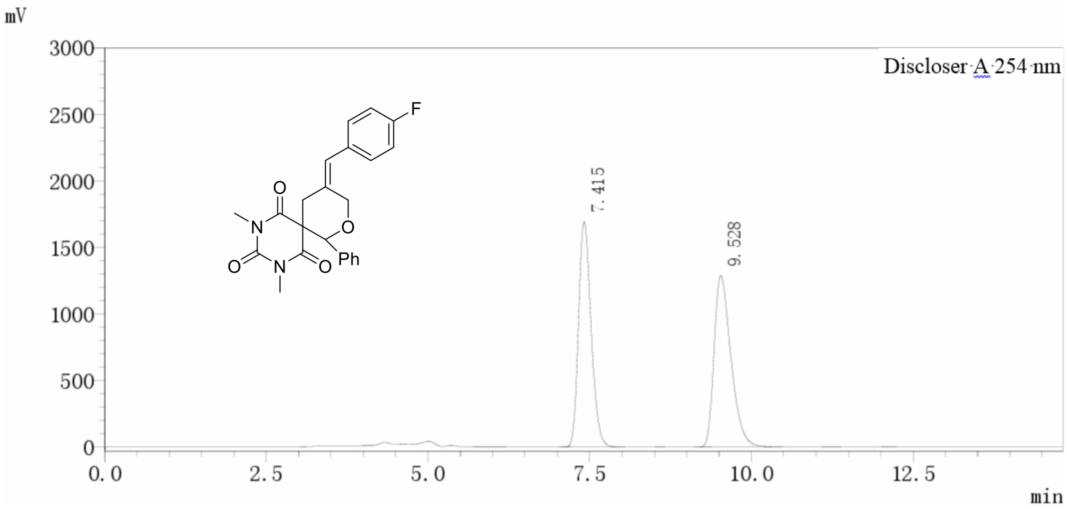

Discloser A 254 nm

Peak	Retention time (min)	Area (%)
1	7.415	49.487
2	9.528	50.513
total		100

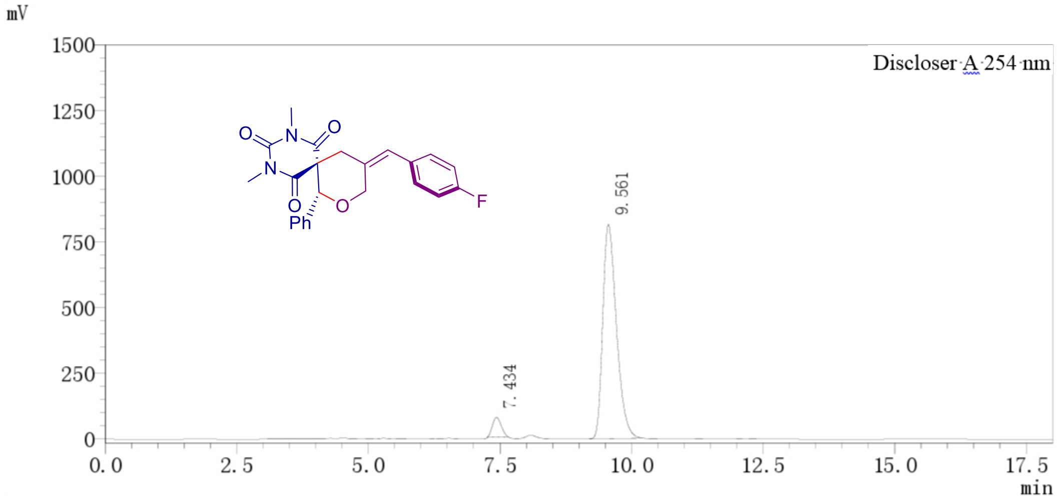

Discloser A 254 nm

Peak	Retention time (min)	Area (%)
1	7.434	5.657
2	9.561	94.343
total		100

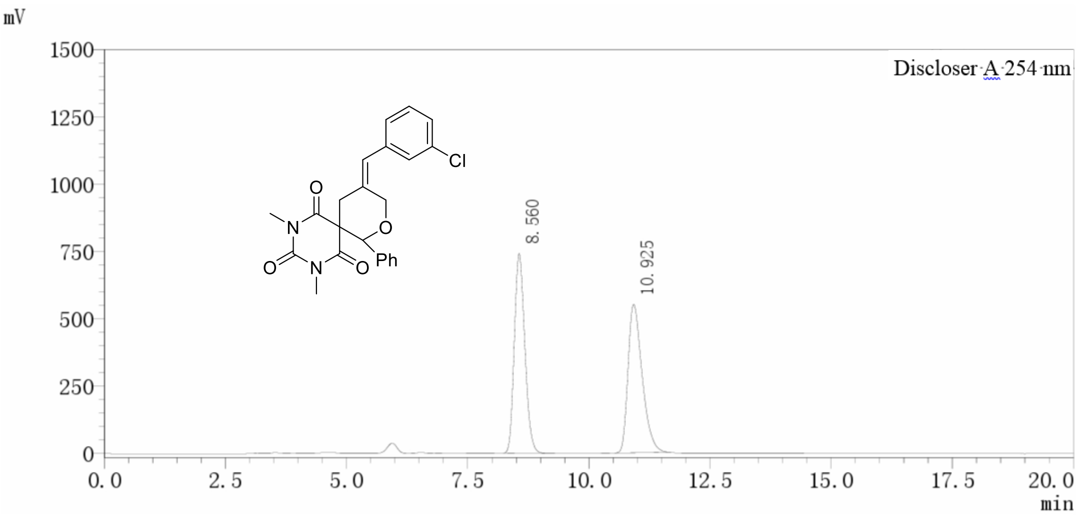

Discloser A 254 nm

Peak	Retention time (min)	Area (%)
1	8.560	49.900
2	10.925	50.100
total		100

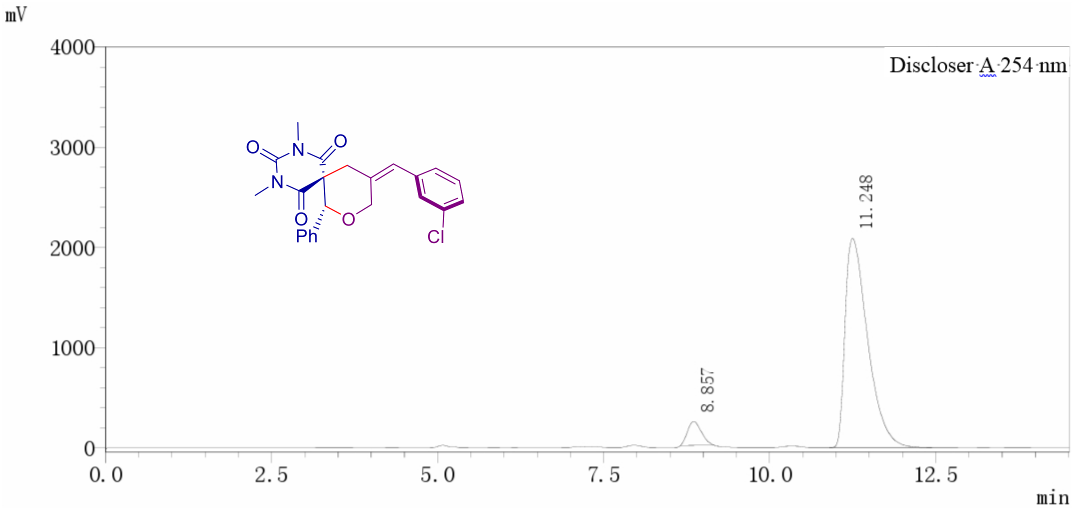

Discloser A 254 nm

Peak	Retention time (min)	Area (%)
1	8.857	6.567
2	11.248	93.433
total		100

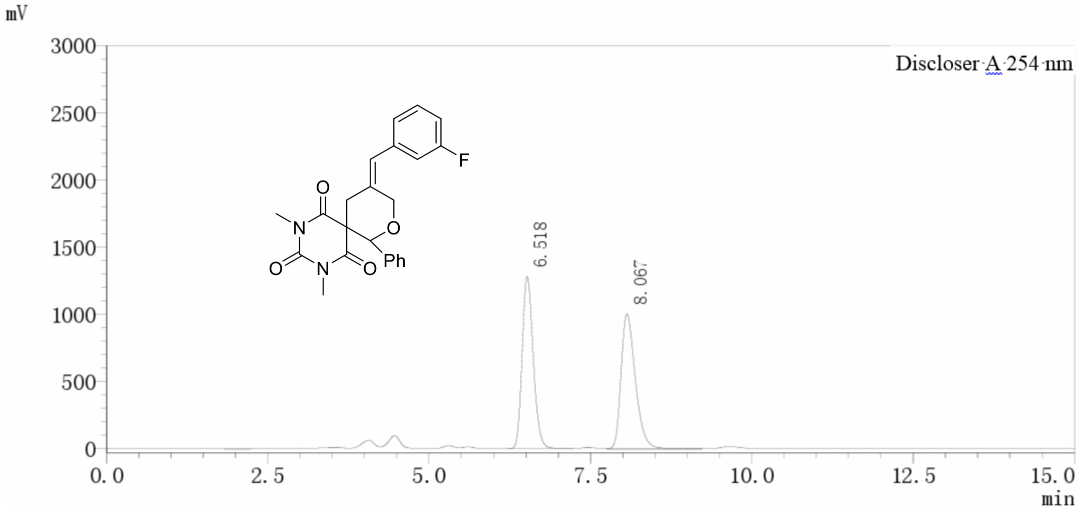

Discloser A 254 nm

Peak	Retention time (min)	Area (%)
1	6.518	49.812
2	8.067	50.188
total		100

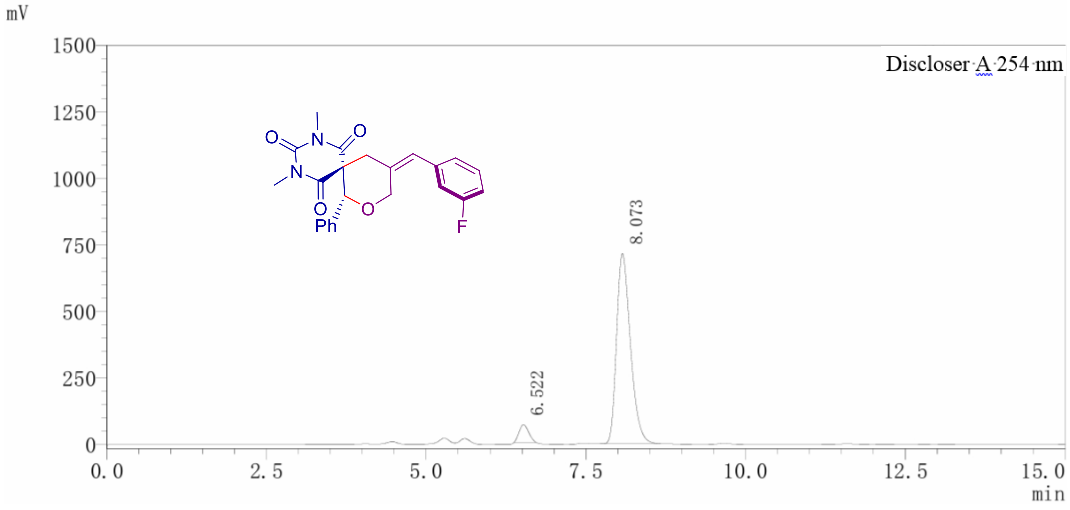

Discloser A 254 nm

Peak	Retention time (min)	Area (%)
1	6.522	5.954
2	8.073	94.046
total		100

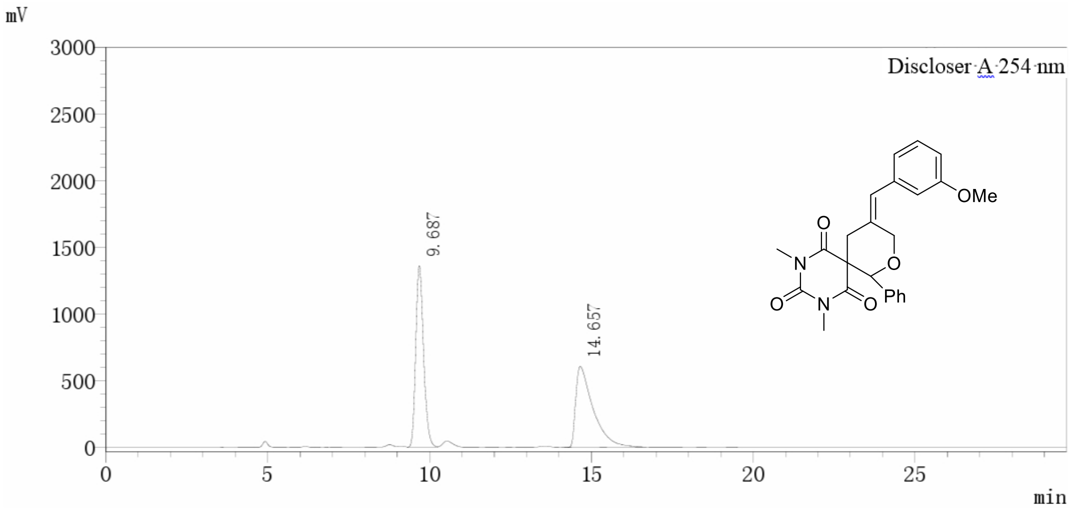

Discloser A 254 nm

Peak	Retention time (min)	Area (%)
1	9.687	49.884
2	14.657	50.116
total		100

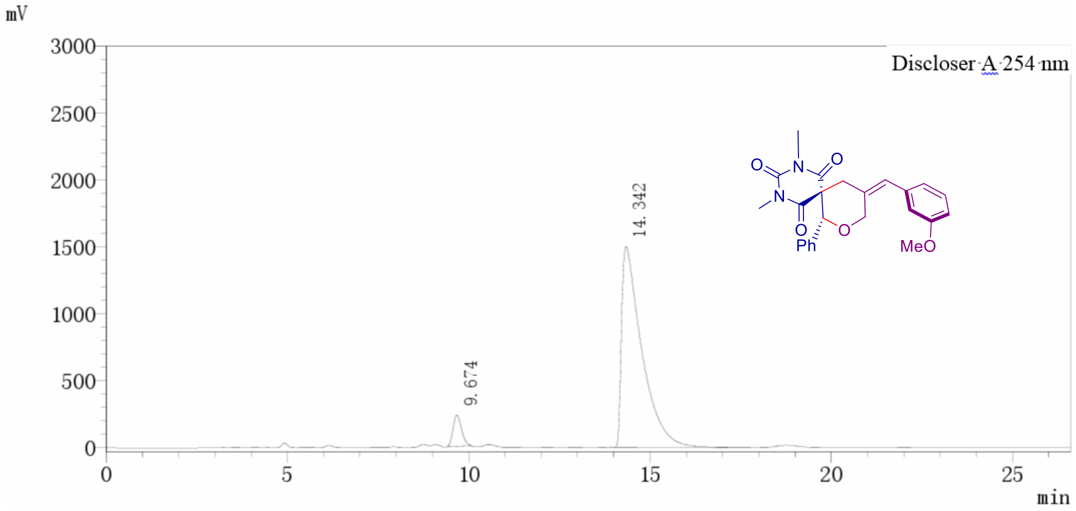

Discloser A 254 nm

Peak	Retention time (min)	Area (%)
1	9.674	6.119
2	14.342	93.881
total		100

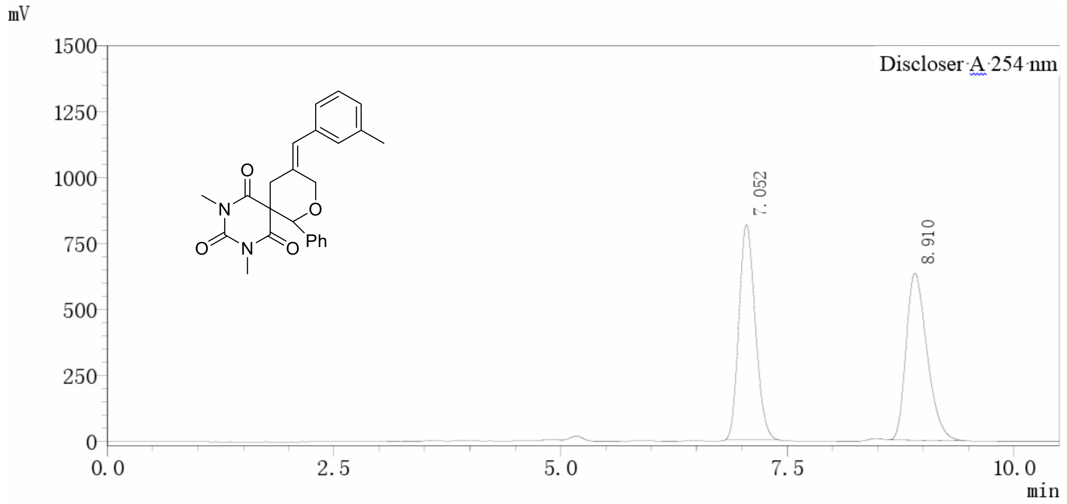

Discloser A 254 nm

Peak	Retention time (min)	Area (%)
1	7.052	50.000
2	8.910	50.000
total		100

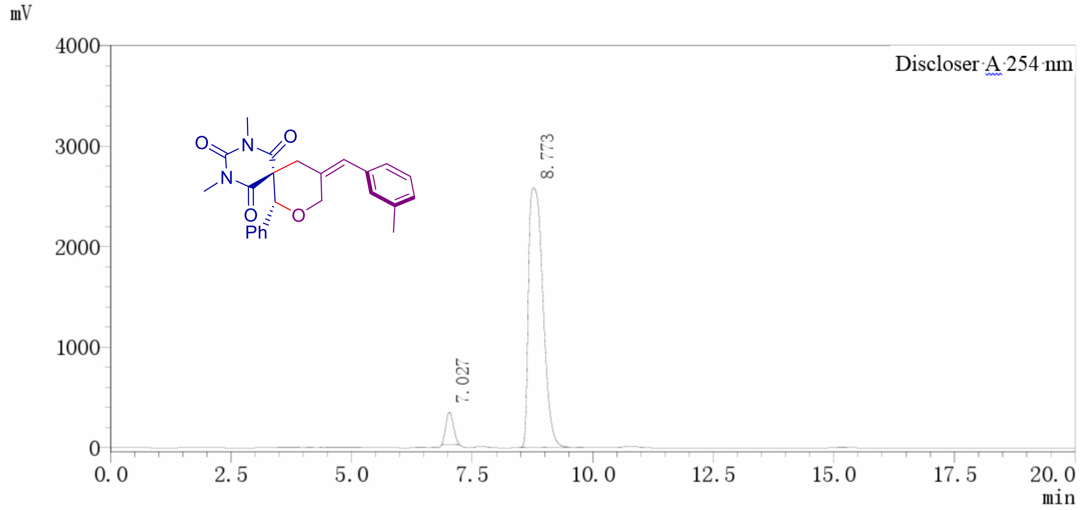

Discloser A 254 nm

Peak	Retention time (min)	Area (%)
1	7.027	6.254
2	8.773	93.746
total		100

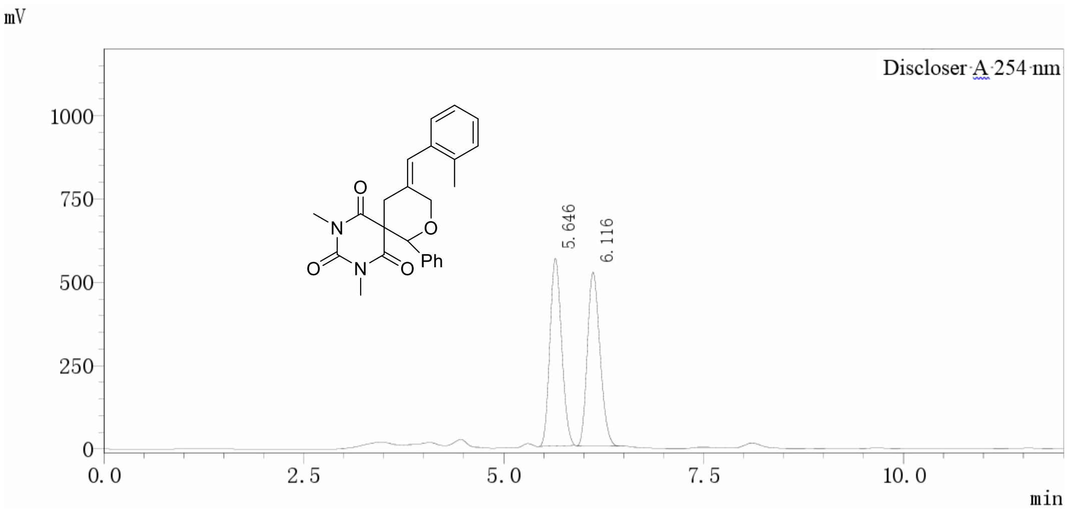

Discloser A 254 nm

Peak	Retention time (min)	Area (%)
1	5.646	50.046
2	6.116	49.954
total		100

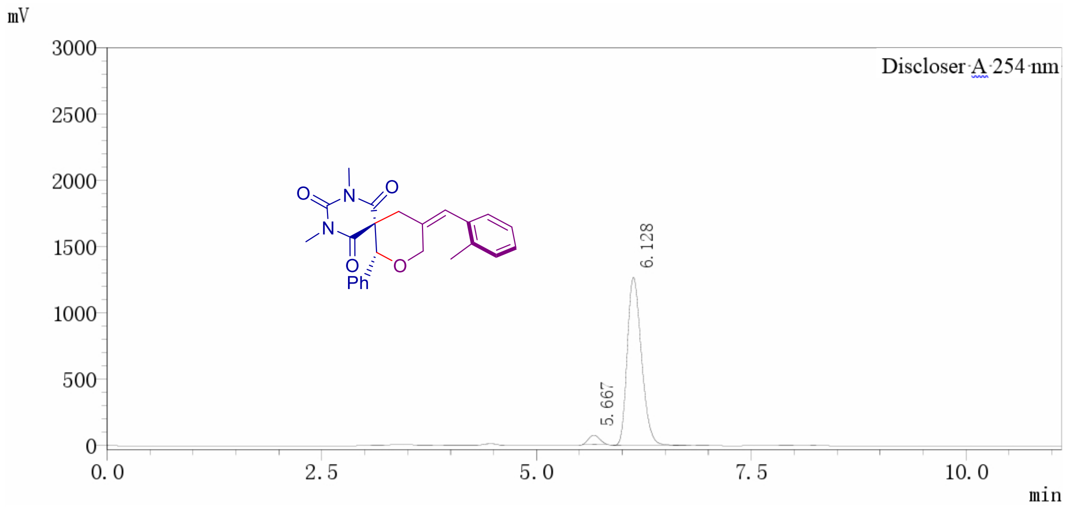

Discloser A 254 nm

Peak	Retention time (min)	Area (%)
1	5.667	4.146
2	6.128	95.854
total		100

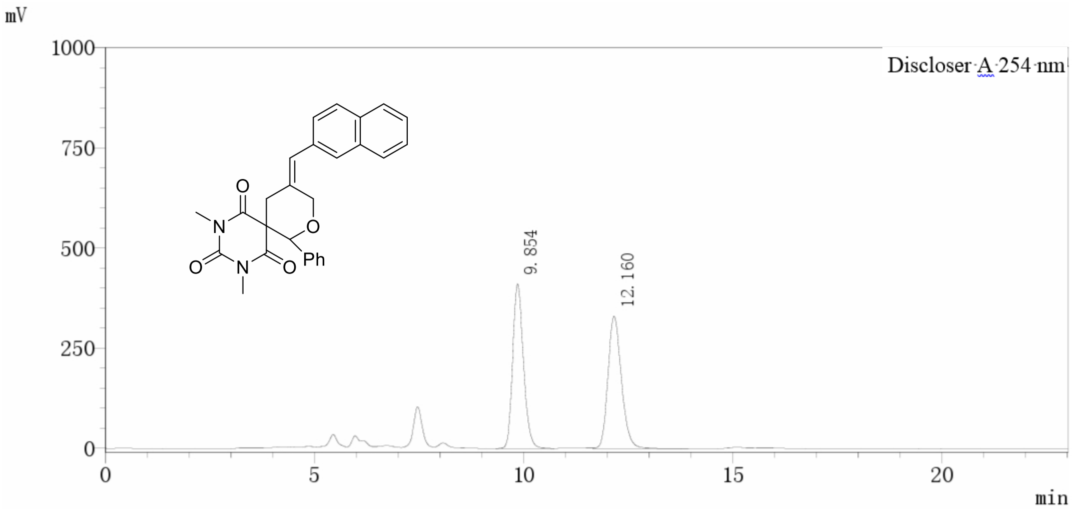

Discloser A 254 nm

Peak	Retention time (min)	Area (%)
1	9.854	49.894
2	12.160	50.106
total		100

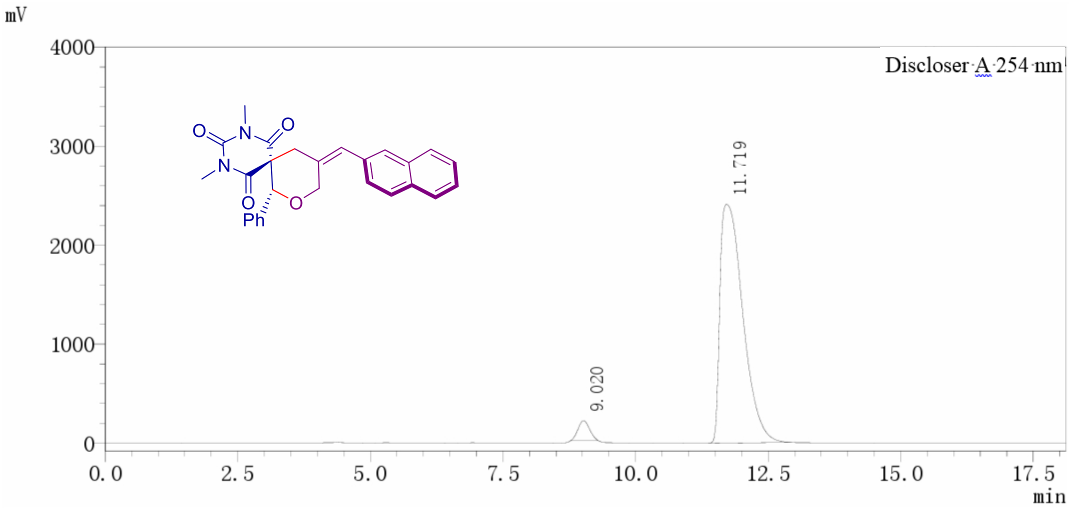

Discloser A 254 nm

Peak	Retention time (min)	Area (%)
1	9.020	4.129
2	11.719	95.871
total		100

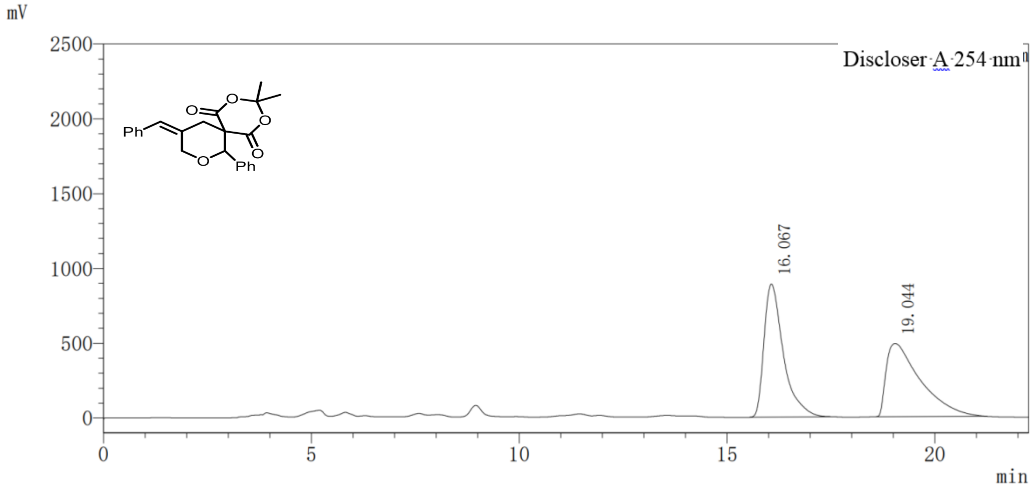

Discloser A 254 nm

Peak	Retention time (min)	Area (%)
1	16.067	50.801
2	19,044	49.199
total		100

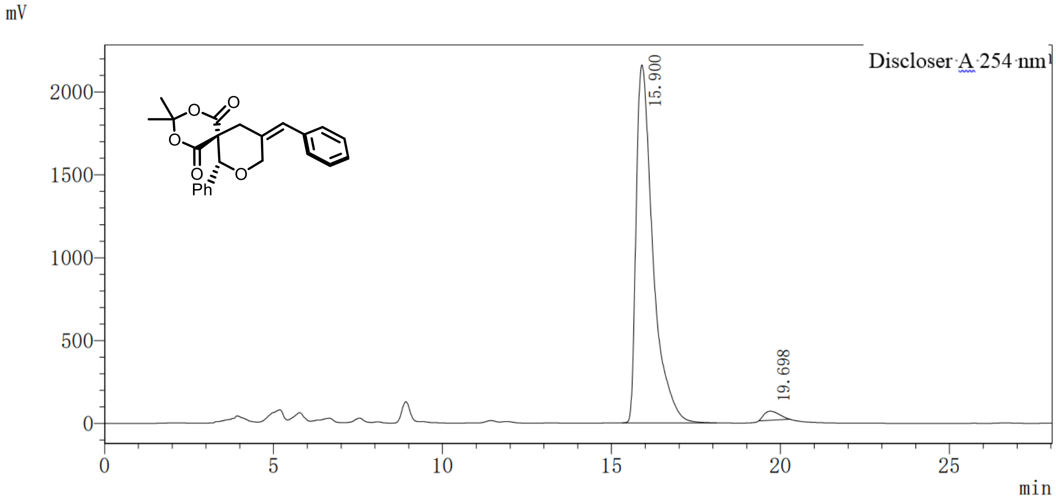

Discloser A 254 nm

Peak	Retention time (min)	Area (%)
1	15.900	97.755
2	19.698	2.245
total		100

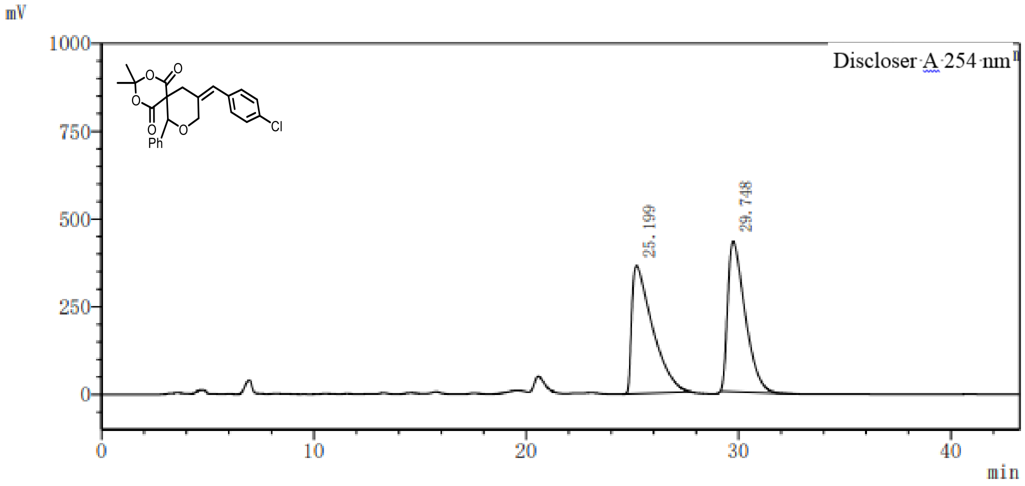

Discloser A 254 nm

Peak	Retention time (min)	Area (%)
1	25.199	50.373
2	29.748	49.627
total		100

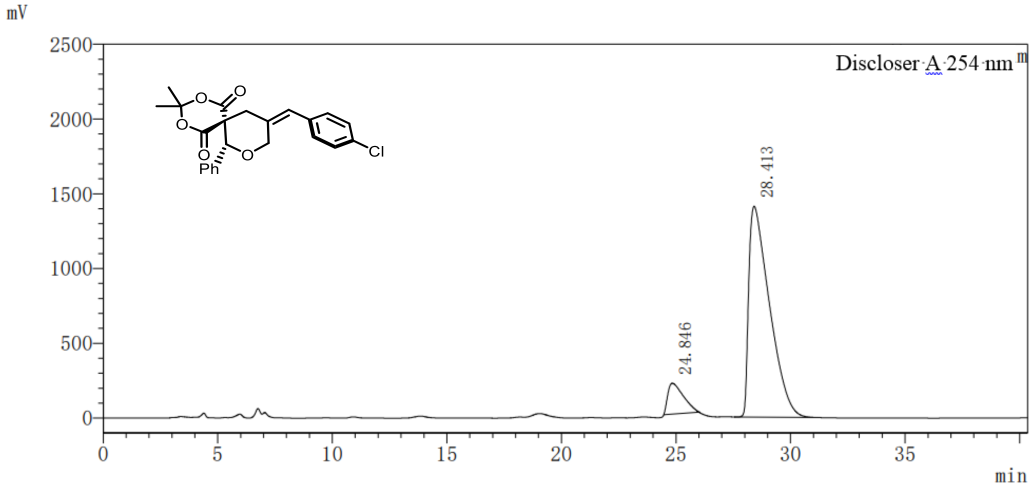

Discloser A 254 nm

Peak	Retention time (min)	Area (%)
1	24.846	9.867
2	28.413	90.133
total		100

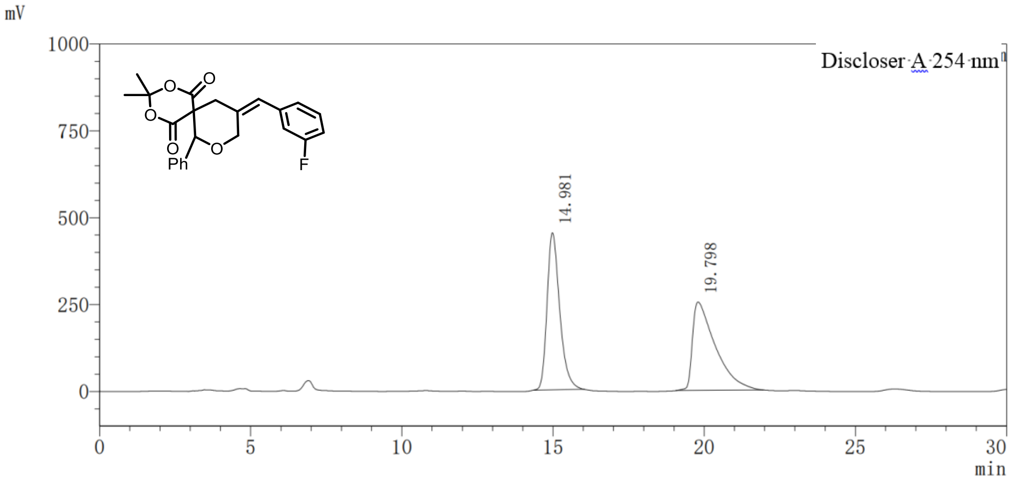

Discloser A 254 nm

Peak	Retention time (min)	Area (%)
1	14.981	49.558
2	19,798	50.442
total		100

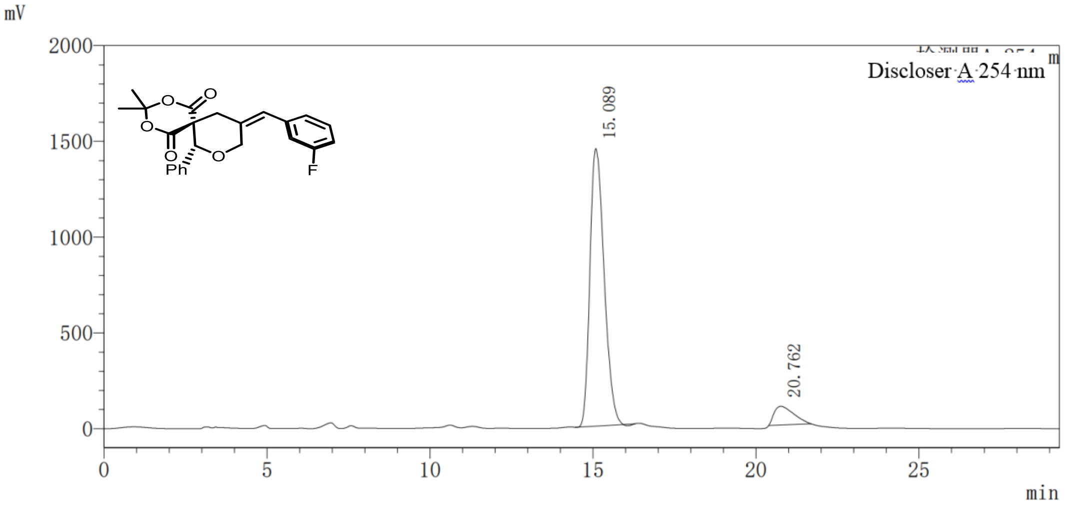

Discloser A 254 nm

Peak	Retention time (min)	Area (%)
1	15.089	91.588
2	20.762	8.412
total		100

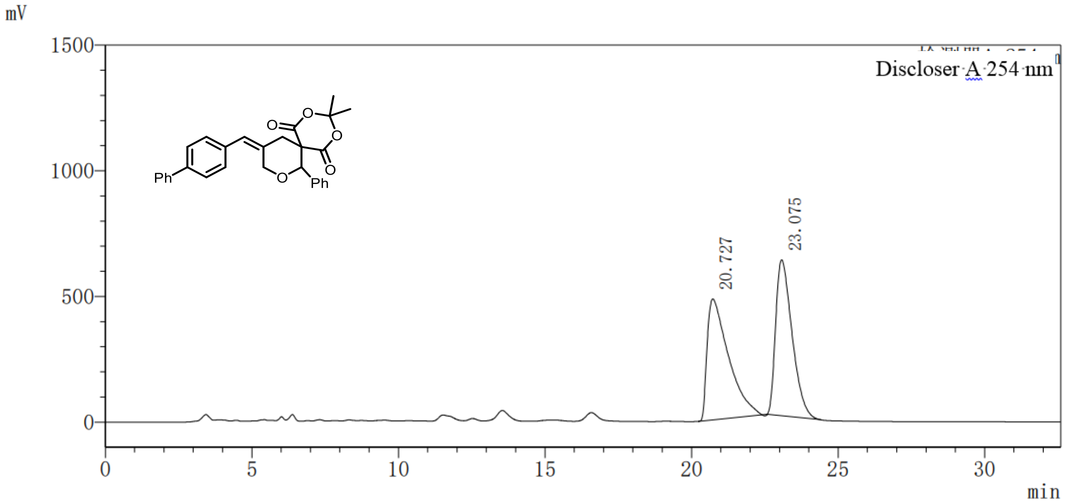

Discloser A 254 nm

Peak	Retention time (min)	Area (%)
1	20.727	50.244
2	23.075	49.756
total		100

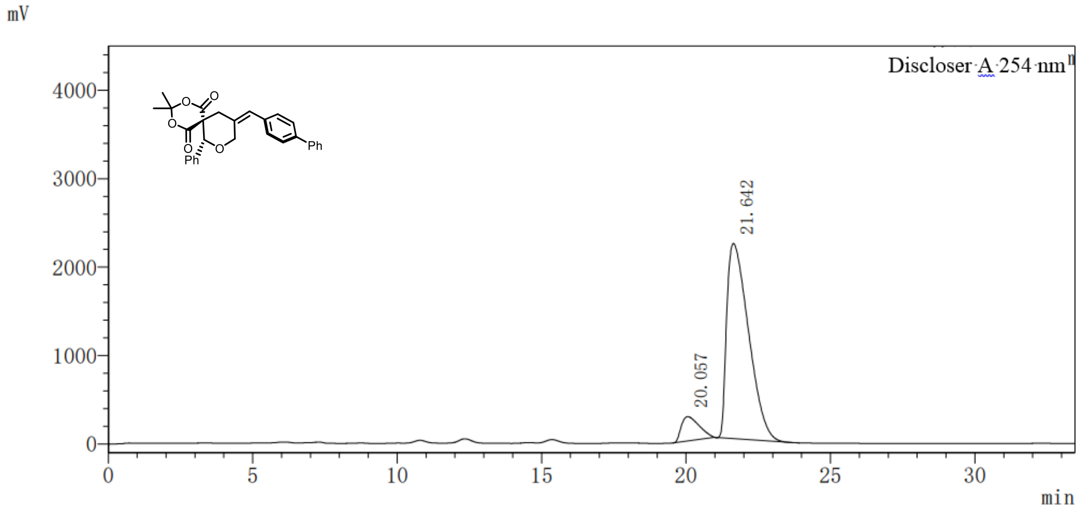

Discloser A 254 nm

Peak	Retention time (min)	Area (%)
1	20.057	9.206
2	21.642	90.794
total		100

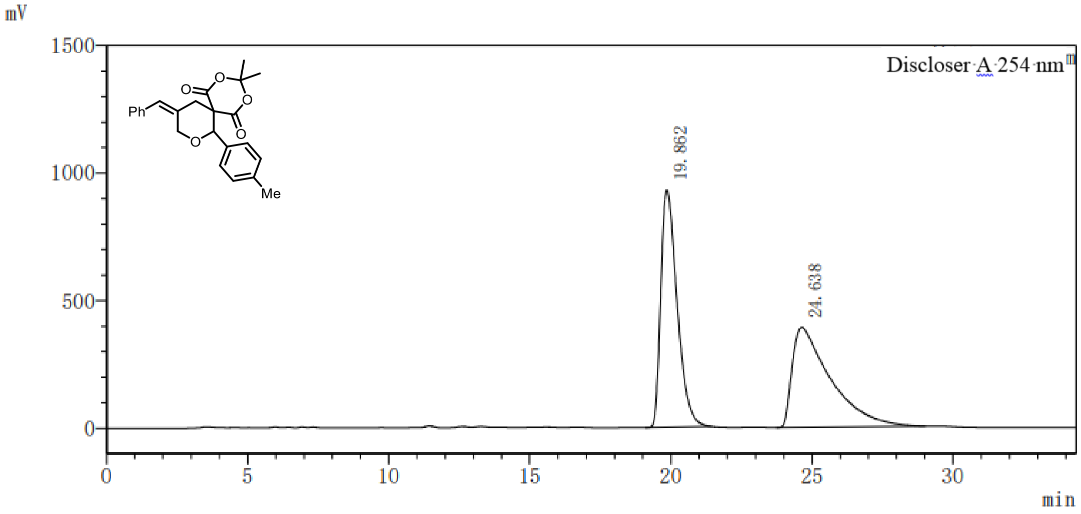

Discloser A 254 nm

Peak	Retention time (min)	Area (%)
1	19.862	50.300
2	24.638	49.700
total		100

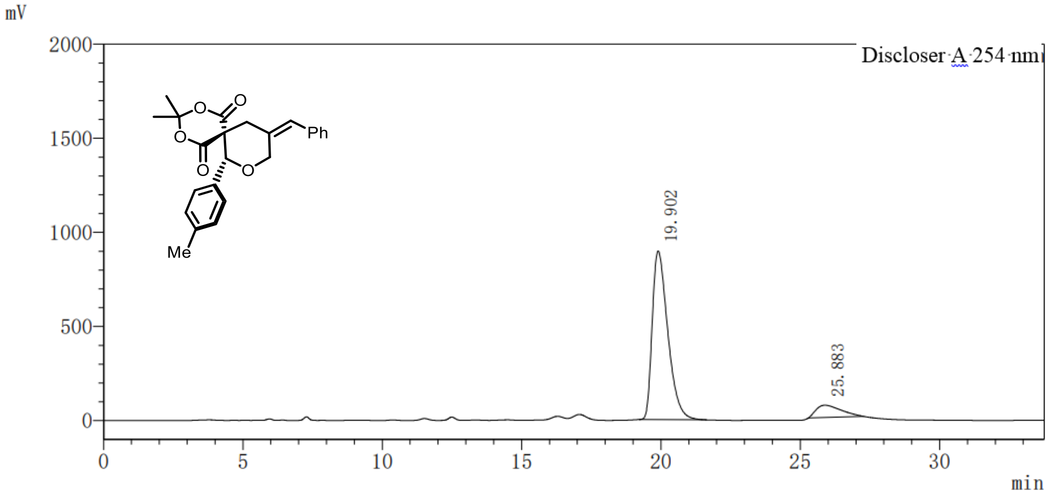

Discloser A 254 nm

Peak	Retention time (min)	Area (%)
1	19.902	89.524
2	25.883	10.476
total		100

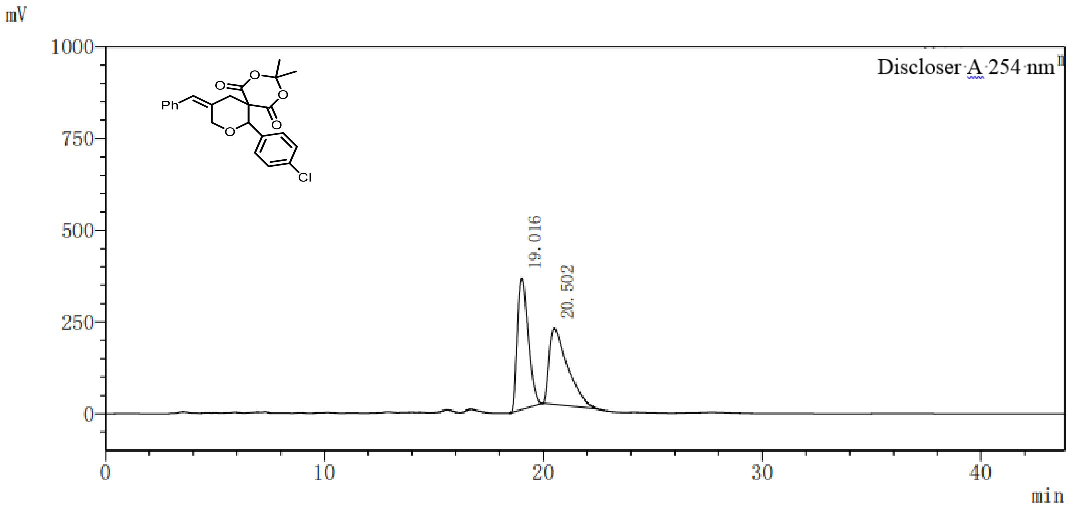

Discloser A 254 nm

Peak	Retention time (min)	Area (%)
1	19.015	50.243
2	20.502	49.757
total		100

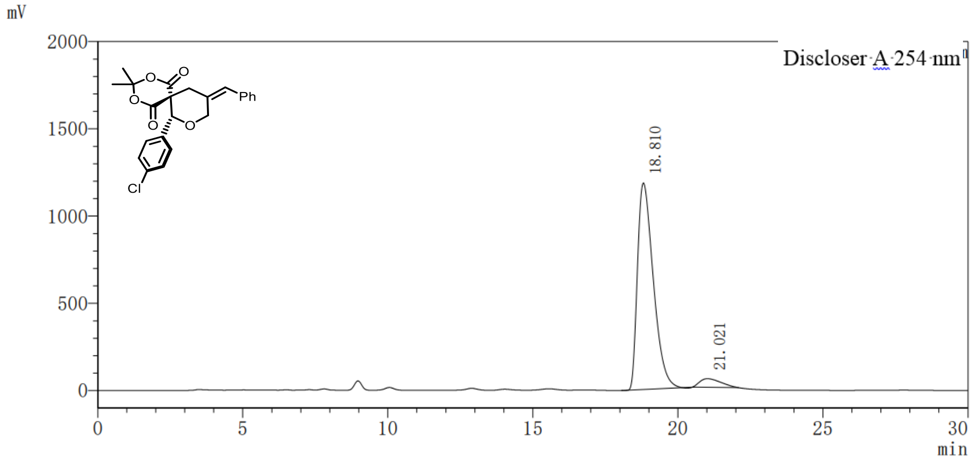

Discloser A 254 nm

Peak	Retention time (min)	Area (%)
1	18.810	94.748
2	21.021	5.252
total		100

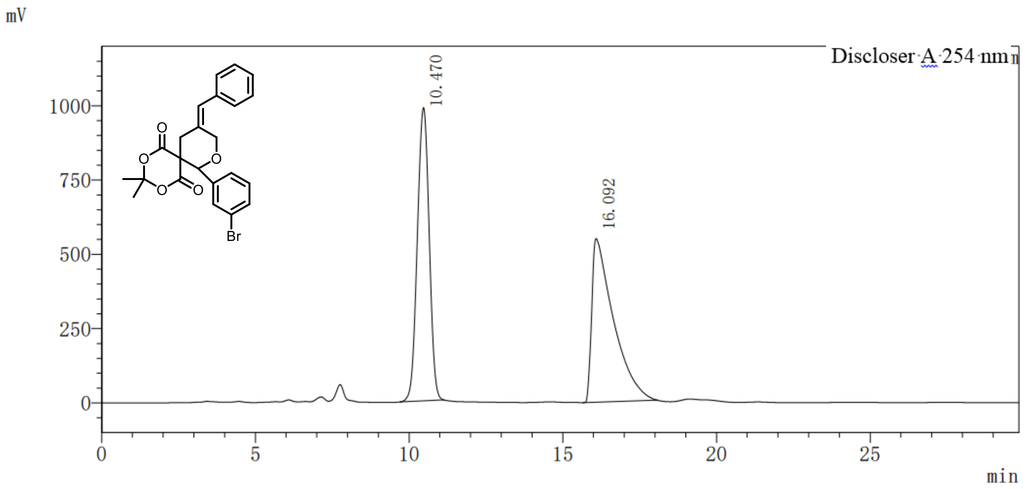

Discloser A 254 nm

Peak	Retention time (min)	Area (%)
1	10.470	49.691
2	16.092	50.309
total		100

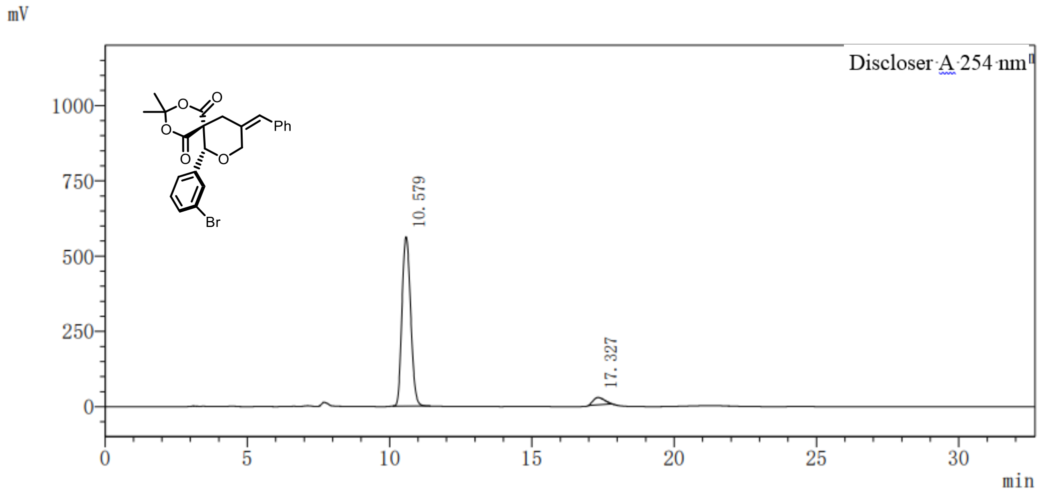

Discloser A 254 nm

Peak	Retention time (min)	Area (%)
1	10.579	94.737
2	17.327	5.263
total		100

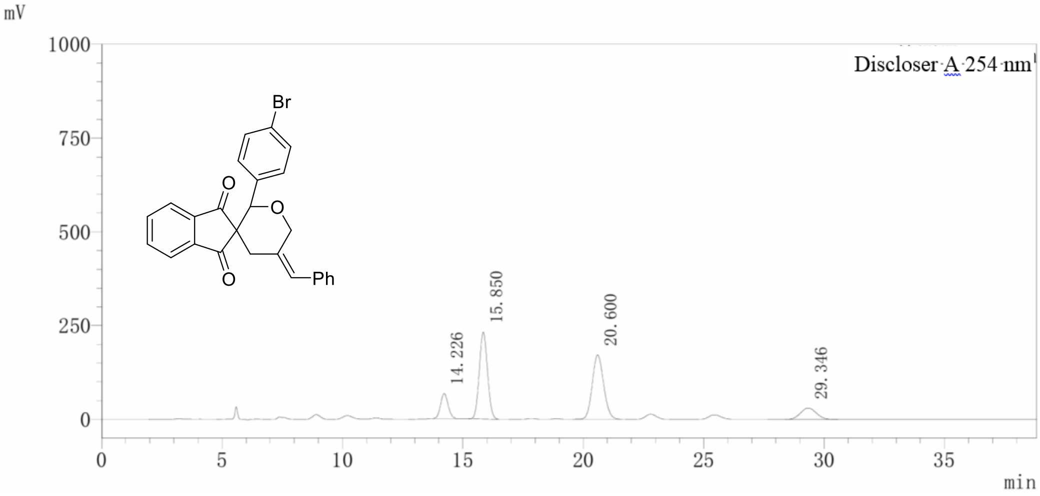

Discloser A 254 nm

Peak	Retention time (min)	Area (%)
1	14.226	10.353
2	15.850	39.332
3	20.600	40.244
4	29.346	10.071
total		100

Condition A:

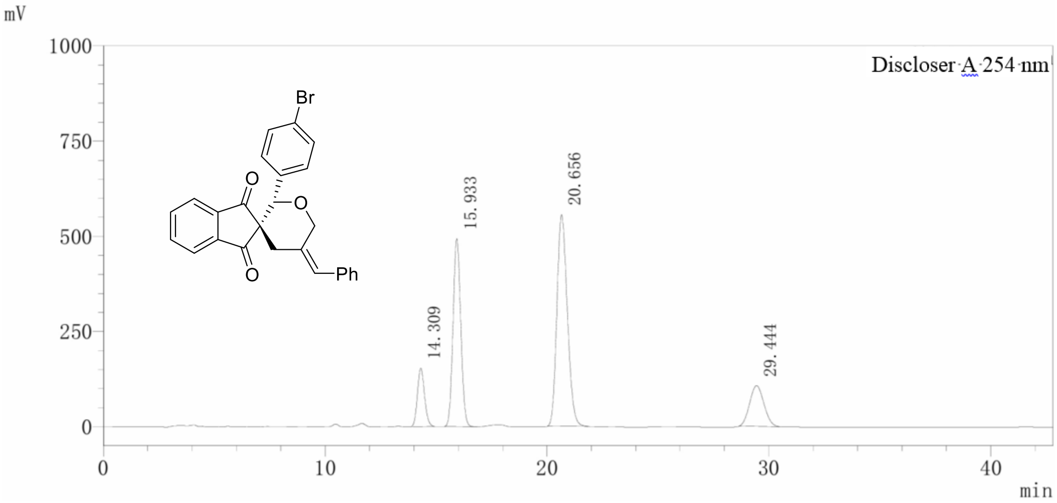

Discloser A 254 nm

Peak	Retention time (min)	Area (%)
1	14.309	8.845
2	15.933	31.118
3	20.656	47.521
4	29.444	12.517

total		100

Condition B:

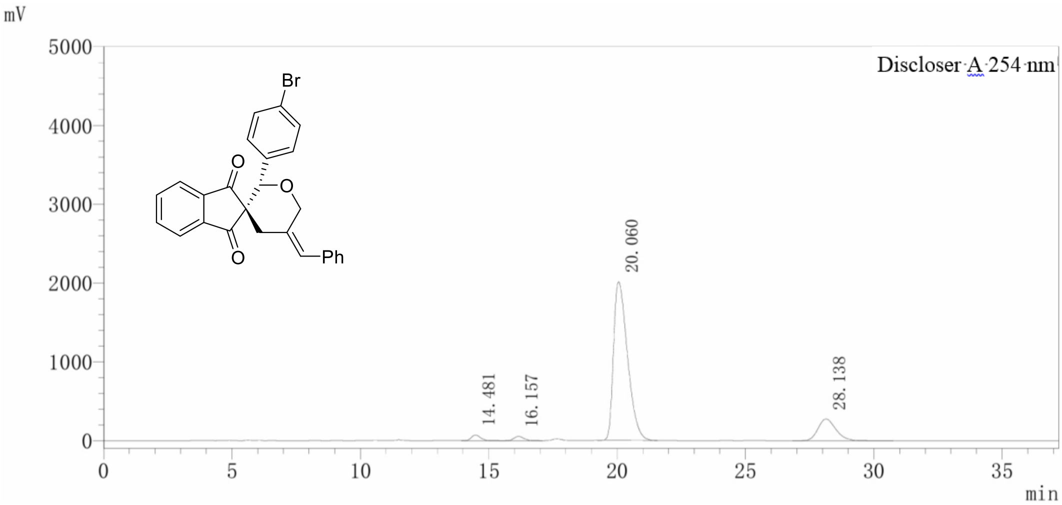

Discloser A 254 nm

Peak	Retention time (min)	Area (%)
1	14.481	2.041
2	16.157	1.635
3	20.060	81.944
4	28.138	14.381
total		100

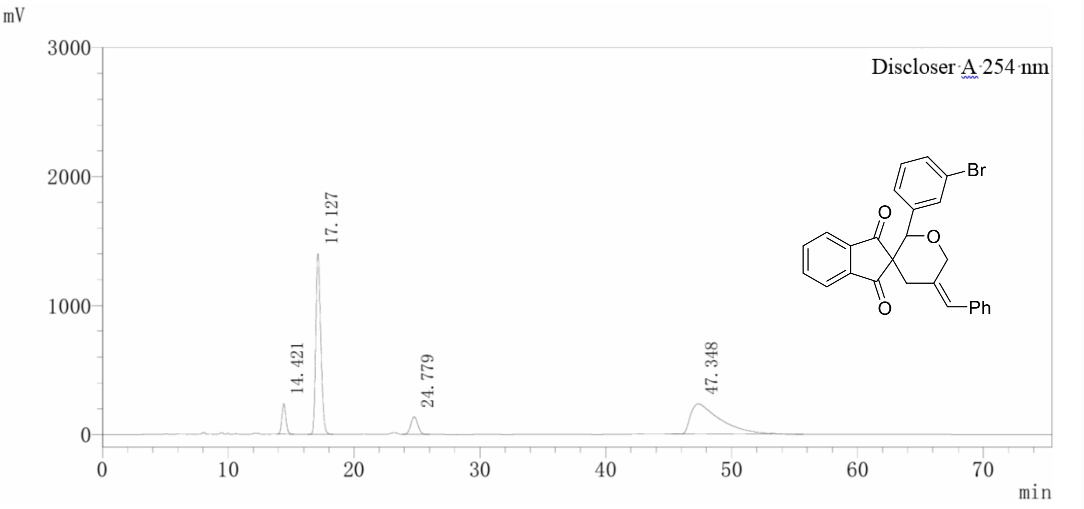

Discloser A 254 nm

Peak	Retention time (min)	Area (%)
1	14.421	6.037
2	17.127	44.531
3	24.779	6.026
4	47.348	43.406
total		100

Condition A:

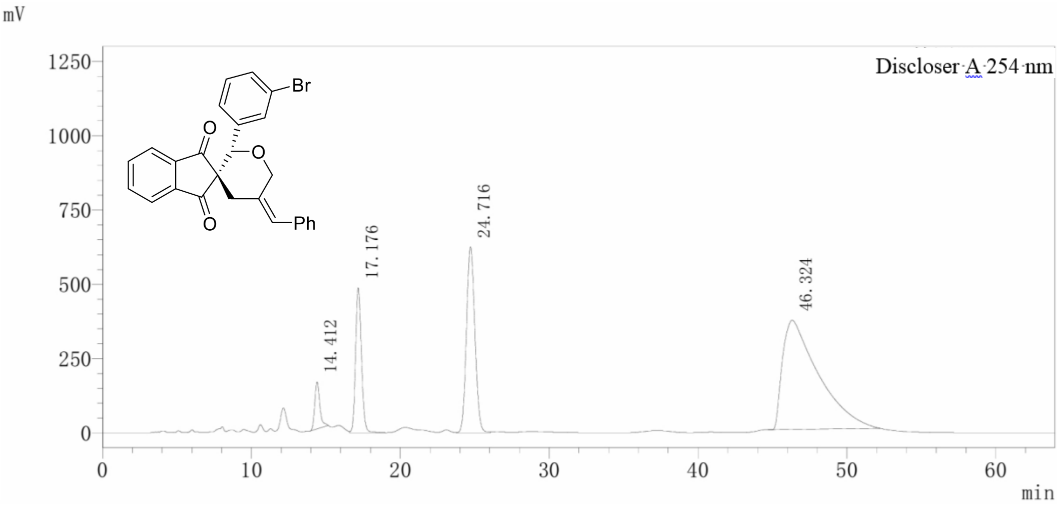

Discloser A 254 nm

Peak	Retention time (min)	Area (%)
1	14.412	3.632
2	17.176	13.013
3	24.716	23.865
4	46.324	59.490
total		100

Condition B:

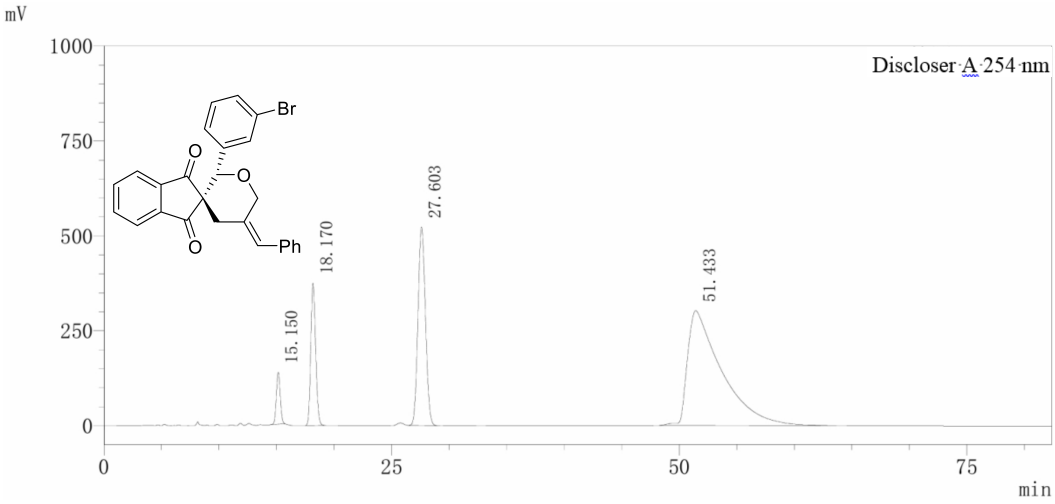

Discloser A 254 nm

Peak	Retention time (min)	Area (%)
1	15.150	3.235
2	18.170	11.040
3	27.603	23.544
4	51.433	62.180

total		100

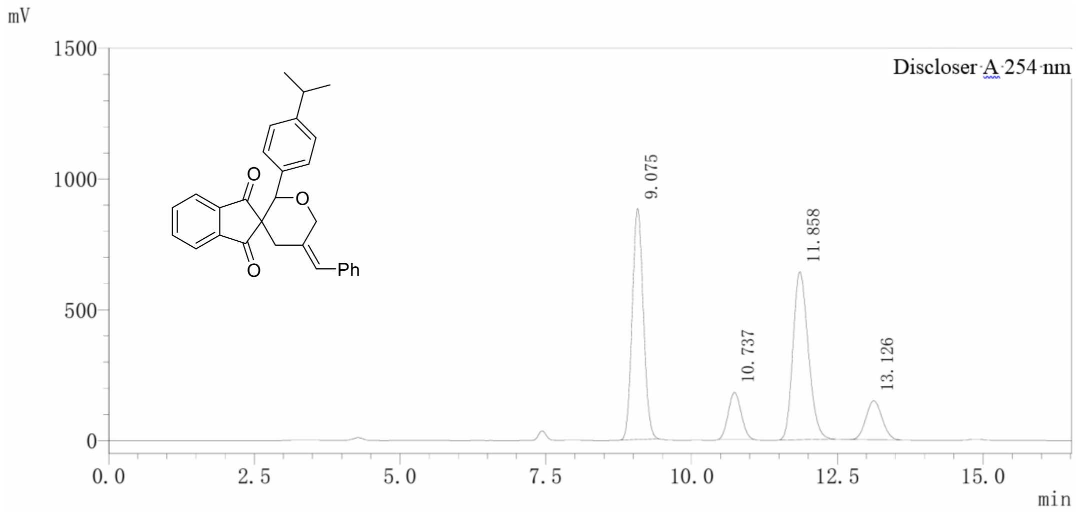

Discloser A 254 nm

Peak	Retention time (min)	Area (%)
1	9.075	40.282
2	10.737	9.498
3	11.858	40.373
4	13.126	9.847
total		100

Condition A:

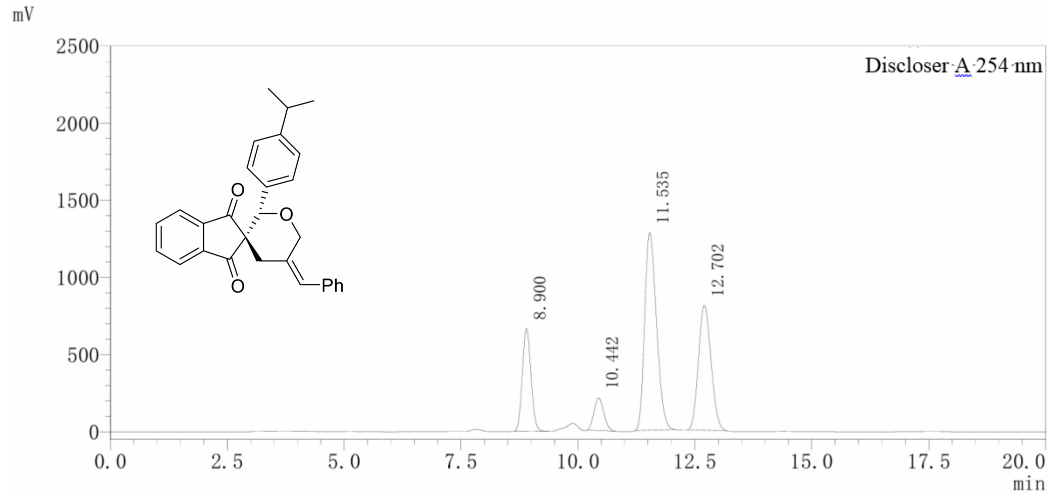

Discloser A 254 nm

Peak	Retention time (min)	Area (%)
1	8.900	17.338
2	10.442	6.300
3	11.535	45.664
4	12.702	30.698
total		100

Condition B:

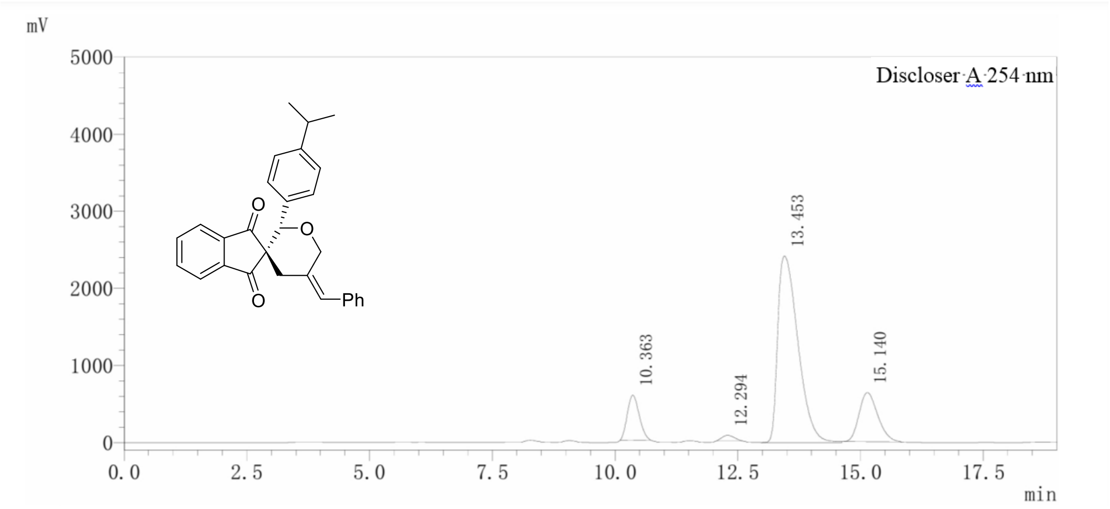

Discloser A 254 nm

Peak	Retention time (min)	Area (%)
1	10.363	10.085
2	12.294	1.395
3	13.453	71.205
4	15.140	17.315
total		100

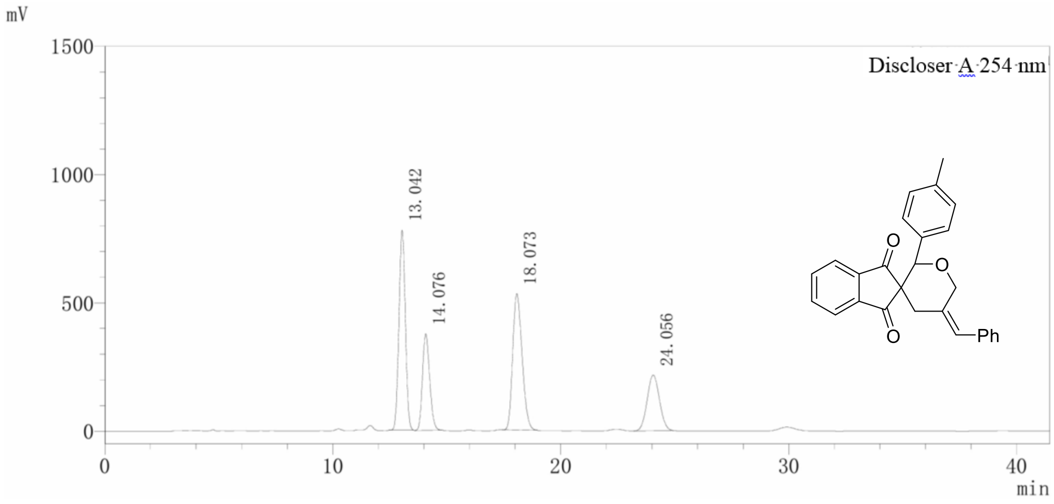

Discloser A 254 nm

Peak	Retention time (min)	Area (%)
1	13.042	32.502
2	14.076	17.531

3	18.073	32.462
4	24.056	17.505
total		100

Condition A:

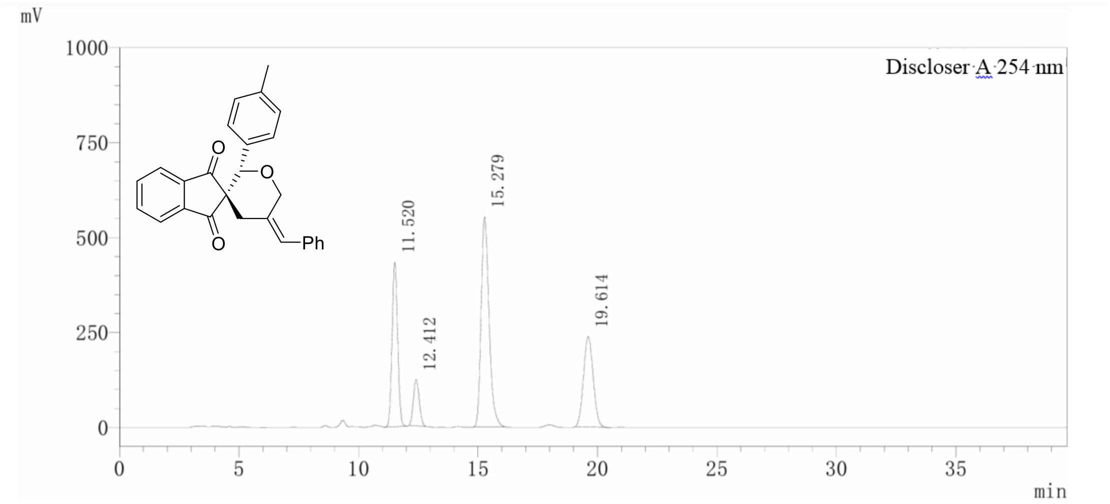

Discloser A 254 nm

Peak	Retention time (min)	Area (%)
1	11.520	24.600
2	12.412	7.286
3	15.279	44.651
4	19.614	23.463
total		100

Condition B:

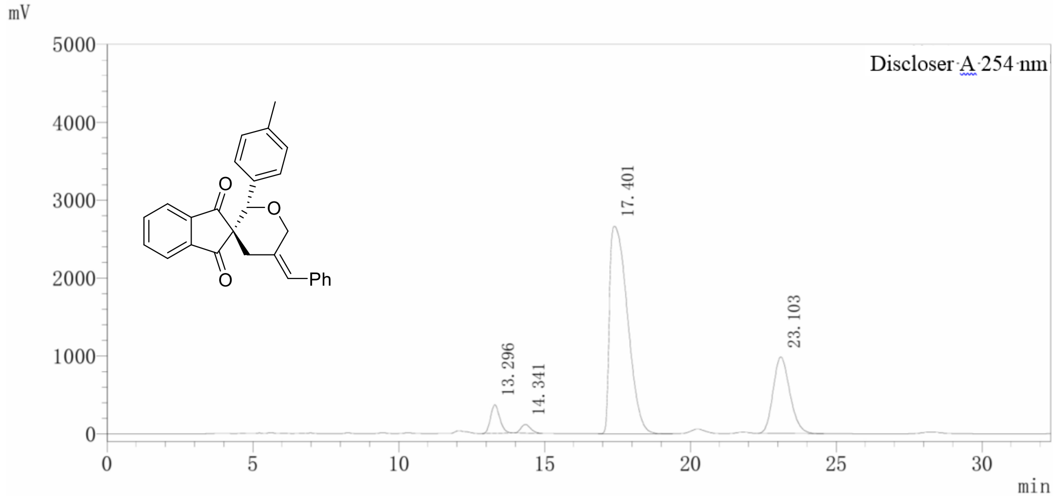

Discloser A 254 nm

Peak	Retention time (min)	Area (%)
1	13.296	4.891
2	14.341	1.562

3	17.401	69.607
4	23.103	23.941
total		100

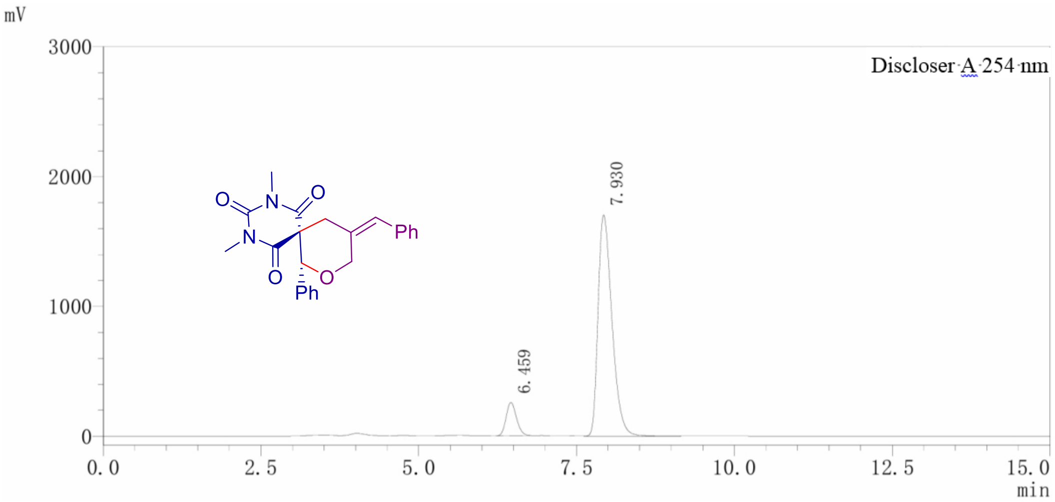

Discloser A 254 nm

Peak	Retention time (min)	Area (%)
1	6.459	9.959
2	7.930	90.041
total		100

7. X-ray crystal structures

The X-ray crystallographic structures for **6** and **(*S*,*Z*)-5'-benzylidene-2'-phenyl-5',6'-dihydro-2'*H*,4'*H*-spiro[indene-2,3'-pyran]-1,3-dione.** ORTEP view of the molecules of complex showing ellipsoids at 30% probability level. Crystal data have been deposited to CCDC, number **6** (2348299) and **(*S*,*Z*)-5'-benzylidene-2'-phenyl-5',6'-dihydro-2'*H*,4'*H*-spiro[indene-2,3'-pyran]-1,3-dione** (2356661). A summary of the fundamental crystal and refinement data are given in the Table S1 of the Supporting Information. Atomic coordinates, anisotropic displacement parameters and bond lengths and angles can be found in the cif files.

Crystals suitable for X-ray diffraction (Gemini E) were grown by n-hexane/ethyl acetate solution.

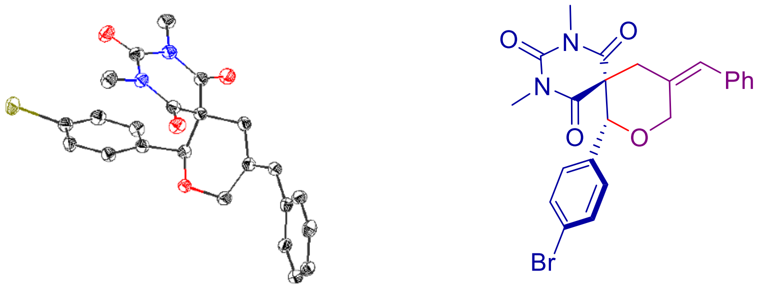

Crystal structure of **6 (2348299)**

Table S10 Crystal data and structure refinement for 6.

Identification code	2348299
Empirical formula	$C_{23}H_{21}BrN_2O_4$
Formula weight	469.33
Temperature/K	150.0
Crystal system	monoclinic
Space group	$P2_1$
a/Å	8.0945(3)
b/Å	8.3620(2)
c/Å	15.1556(6)
α/°	90

β/°	93.6130(10)
γ/°	90
Volume/$Å^3$	1023.79(6)
Z	2
$\rho_{calc}g/cm^3$	1.522
μ/mm^{-1}	2.041
F(000)	480.0
Crystal size/mm^3	0.12 × 0.1 × 0.1
Radiation	MoKα (λ = 0.71073)
2Θ range for data collection/°	5.042 to 52.8
Index ranges	$-10 \le h \le 10$, $-10 \le k \le 10$, $-18 \le l \le 18$
Reflections collected	24601
Independent reflections	4191 [R_{int} = 0.0457, R_{sigma} = 0.0426]
Data/restraints/parameters	4191/1/273
Goodness-of-fit on F^2	1.010
Final R indexes [I>=2σ (I)]	R_1 = 0.0286, wR_2 = 0.0609
Final R indexes [all data]	R_1 = 0.0393, wR_2 = 0.0644
Largest diff. peak/hole / e $Å^{-3}$	0.21/-0.17
Flack parameter	0.005(5)

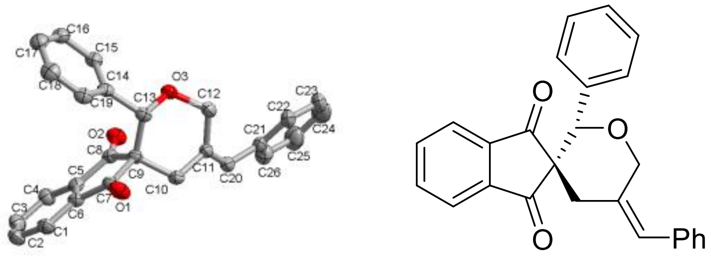

Crystal structure of **(*S*,*Z*)-5'-benzylidene-2'-phenyl-5',6'-dihydro-2'*H*,4'*H*-spiro [indene-2,3'-pyran]-1,3-dione 48 (2356661)**

Table S11 Crystal data and structure refinement for 2356661.

Identification code	2356661
Empirical formula	$C_{26}H_{20}O_3$

Formula weight	380.42
Temperature/K	293(2)
Crystal system	monoclinic
Space group	P21/c
a/Å	10.5399(2)
b/Å	21.5341(5)
c/Å	8.7976(2)
α/°	90
β/°	90.973(2)
γ/°	90
Volume/Å3	1996.48(8)
Z	4
ρcalcg/cm3	1.266
μ/mm 1	0.654
F(000)	800.0
Crystal size/mm3	0.15 × 0.13 × 0.1
Radiation	CuKα (λ = 1.54184)
2Θ range for data collection/°	8.212 to 134.146
Index ranges	-12 ≤ h ≤ 12, -25 ≤ k ≤ 25, -10 ≤ l ≤ 6
Reflections collected	14336
Independent reflections	3577 [Rint = 0.0307, Rsigma = 0.0260]
Data/restraints/parameters	3577/0/262
Goodness-of-fit on F2	1.045
Final R indexes [I>=2σ (I)]	R1 = 0.0491, wR2 = 0.1336
Final R indexes [all data]	R1 = 0.0655, wR2 = 0.1499
Largest diff. peak/hole / e Å-3	0.21/-0.21

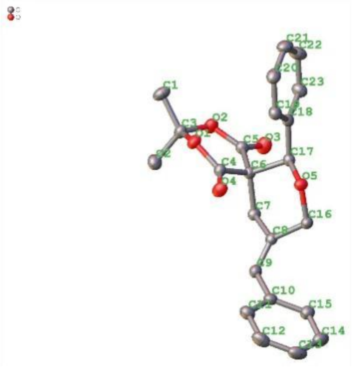

Crystal structure of **44 (CCDC 2429799)**

Table S12 Crystal data and structure refinement for 44.

**Identification code**	**44**
Empirical formula	$C_{23}H_{22}O_5$
Formula weight	378.40
Temperature/K	150.00(10)
Crystal system	monoclinic
Space group	$P2_1/n$
a/Å	8.7418(2)
b/Å	24.5997(5)
c/Å	8.8433(2)
α/°	90
β/°	91.486(2)
γ/°	90
Volume/Å^3	1901.07(7)
Z	4
ρ_{calc}g/cm^3	1.322
μ/mm^{-1}	0.093
F(000)	800.0
Crystal size/mm^3	0.05 × 0.05 × 0.05
Radiation	Mo Kα (λ = 0.71073)
2Θ range for data collection/°	4.896 to 62.348
Index ranges	$-11 \le h \le 11$, $-34 \le k \le 34$, $-12 \le l \le 12$
Reflections collected	35778
Independent reflections	5238 [R_{int} = 0.0488, R_{sigma} = 0.0297]
Data/restraints/parameters	5238/0/255
Goodness-of-fit on F^2	1.052

Final R indexes [I>=2σ (I)]	R_1 = 0.0416, wR_2 = 0.1048
Final R indexes [all data]	R_1 = 0.0483, wR_2 = 0.1081
Largest diff. peak/hole / e Å^{-3}	0.39/-0.24

8. Biological activity experiments

HepG2 cells were cultured in DMEM complete medium supplemented with 10% fetal bovine serum (FBS) and 1% penicillin-streptomycin, and maintained in an incubator at 37 °C with 5% CO_2. The cells were harvested and passaged when they reached an appropriate concentration. Cells in the logarithmic growth phase were centrifuged and resuspended. The cell suspension was mixed uniformly and added to a 96-well plate at 150 μL per well, with at least six replicate wells set up for each condition. An equal volume of PBS was added to the four edges of the 96-well plate, and the plate was then incubated in a cell culture incubator. The cells were treated with drugs, including a control group and various drug concentration gradients. After another 24 hours of incubation in the cell culture incubator, CCK-8 was diluted 1:9 with medium. The original medium in the 96-well plate was aspirated, and the wells were washed twice with PBS. Then, 100 μL of CCK-8 solution was added to each well. The plate was covered with tin foil and incubated in the incubator for 45 minutes. Finally, the optical density (OD) at a wavelength of 450 nm was measured using a microplate reader. To further elucidate the biological applications and potential therapeutic value of the targeted spiro barbituric acid derivatives, we embarked on an extensive series of rigorous biological activity evaluations. The experimental results obtained from these assessments unequivocally demonstrated that compounds 14, 16, 23, and 33 exhibited remarkable tumor cell inhibitory activity within a concentration range of 20 to 125 nanomoles. This pivotal finding underscores their significant potential as drug lead compounds, thereby warranting further exploration and development in the realm of pharmaceutical research and development.

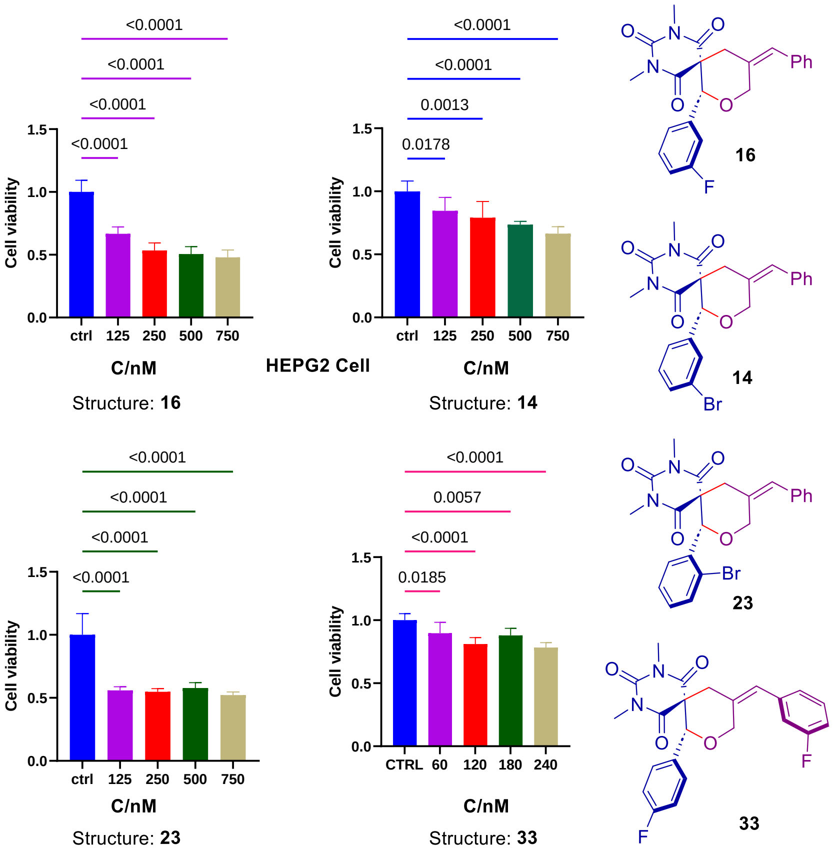

Figure S5. Inhibitory activity against HepG2 cells based on **14**, **16**, **23**, **33**.

9. Computational details

All DFT calculations were carried out using the CP2K code.[1] All calculations employed a mixed Gaussian and planewave basis sets. Core electrons were represented with norm-conserving Goedecker-Teter-Hutter pseudopotentials,[2-4] and the valence electron wavefunction was expanded in a double-zeta basis set with polarization functions[5] along with an auxiliary plane wave basis set with an energy cutoff of 450 eV. The generalized gradient approximation exchange-correlation functional of Perdew, Burke, and Enzerhof (PBE)[6] was used. Each configuration was optimized with the Broyden-Fletcher-Goldfarb-Shanno (BGFS) algorithm with SCF convergence criteria of 1.0×10^{-6} au. To compensate the long-range van der Waals dispersion interaction between the adsorbate and the skeleton, the DFT-D3[7] with an empirical damped potential term was added into the energies obtained from exchange-correlation functional in all calculations.

The Gibbs free energy change for each elementary step was calculated at 298.15 K, defining as follows:

$$\Delta G = \Delta E_{DFT} + \Delta E_{ZPE} - T\Delta S$$

where is the difference of electronic energy calculated with CP2K, is the difference of zero-point energy (ZEP), and is the changed entropy value. E_{ZPE} and were calculated using the following equations for each reaction intermediates,

$$E_{ZPE} = \frac{1}{2}\sum_i hv_i$$

$$TS = \sum_i hv_i\left(\frac{1}{e^{hv_i/k_BT}}\right) - k_BT\sum_i \ln\left(1 - e^{-hv_i/k_BT}\right)$$

where h, v_i, and are Planck's constant, vibrational frequencies, and Boltzmann constant, respectively.

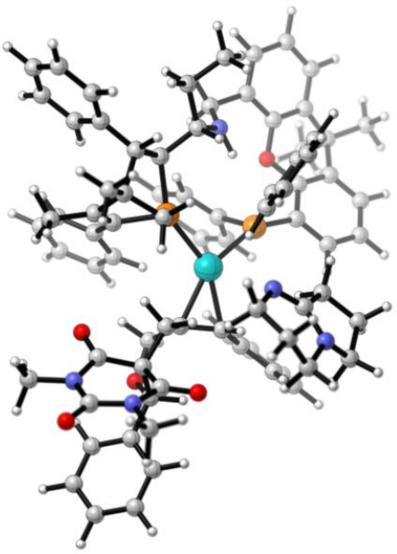

Int 1

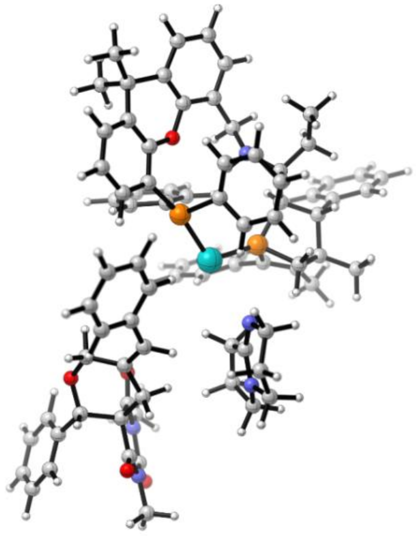

TS 1

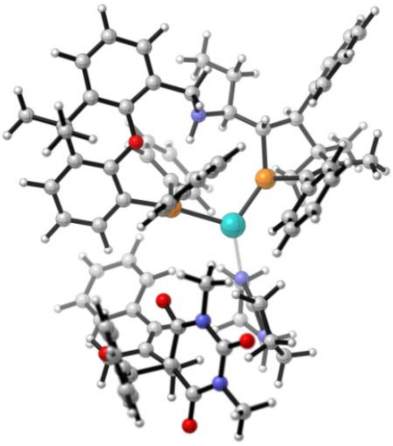

Int (2+3)

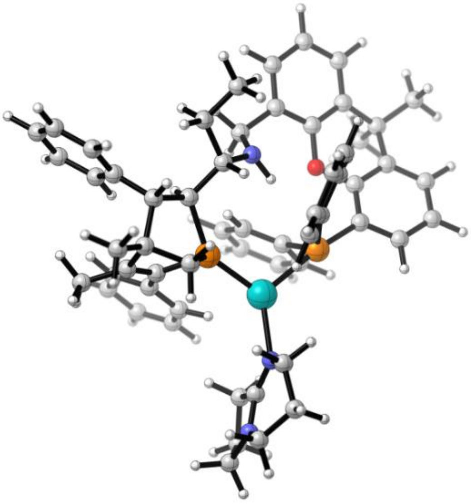

Int 3

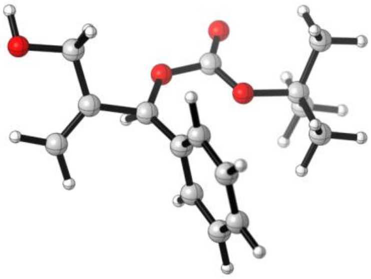

Int 4

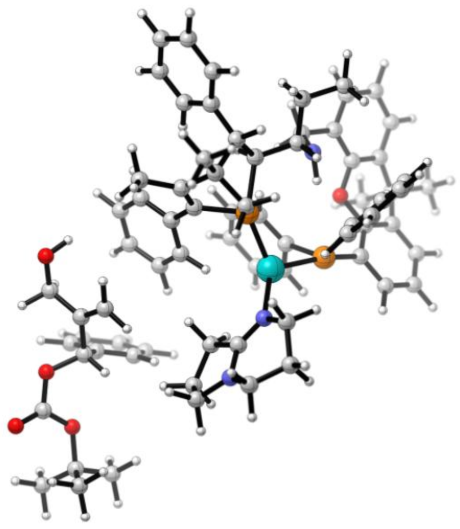

Int (3+4)

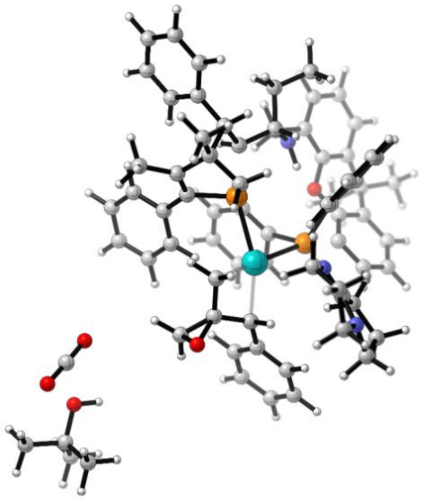

TS 2

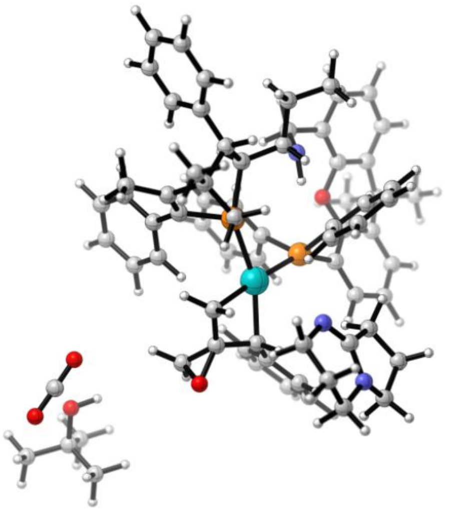

Int (5+6)

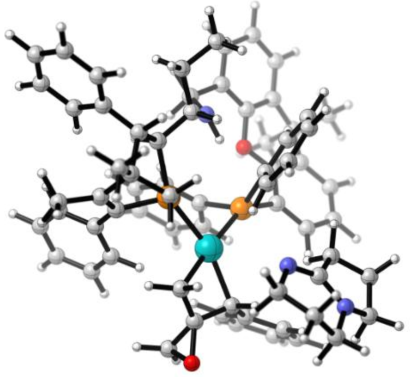

Int 6

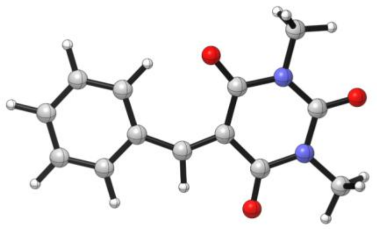

Int 7

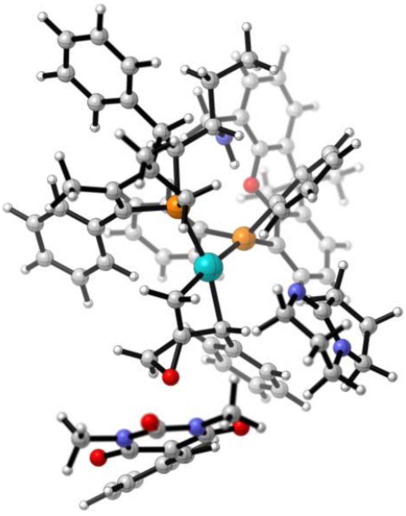

Int (6+7)

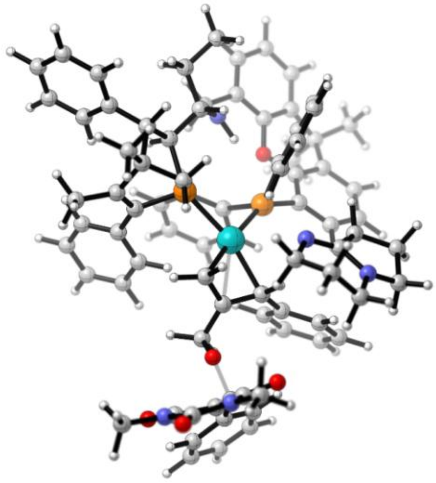

TS 3

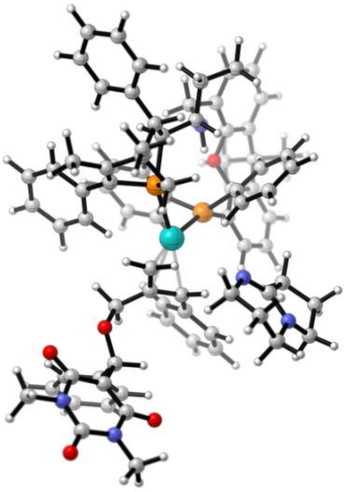

Int 8

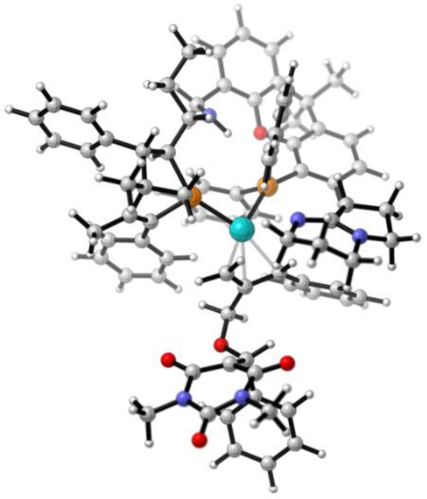

TS 4

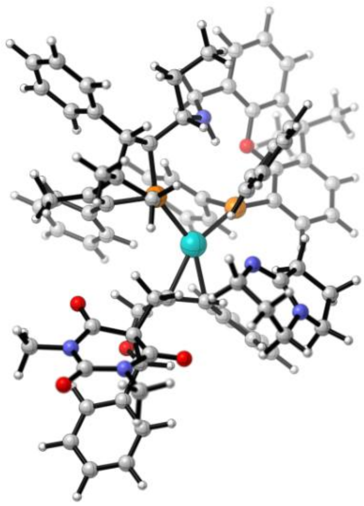

Int 1

Figure S6. DFT-optimized configuration of reaction intermediates with DNB during the reaction.

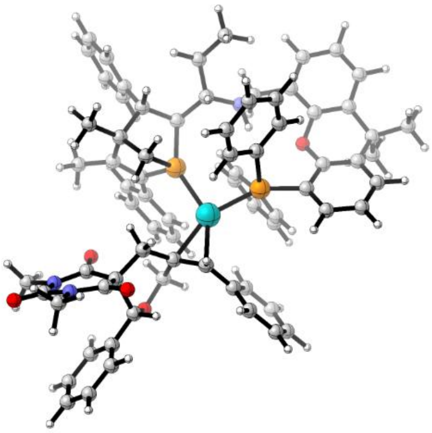

Int 1’

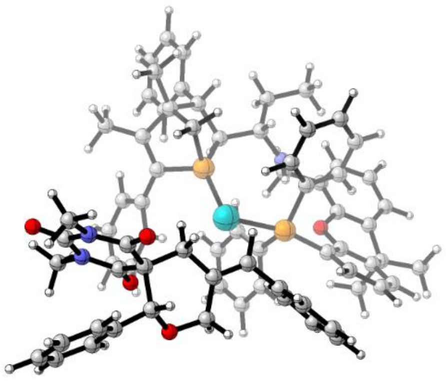

TS 1’

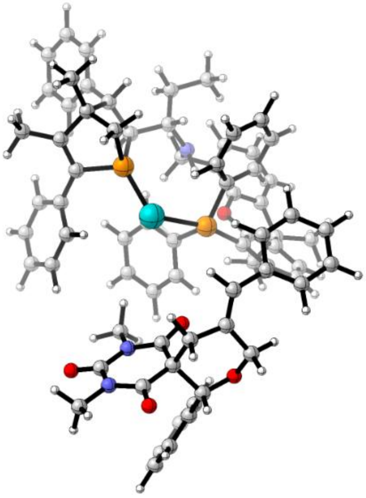

Int (2+3)'

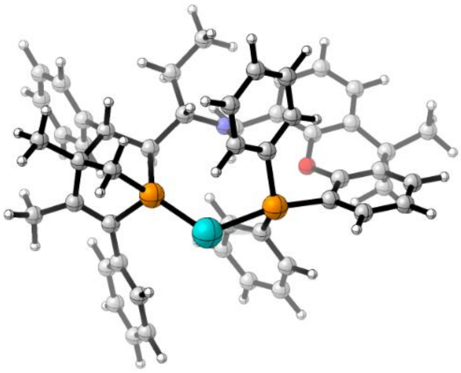

Int 3'

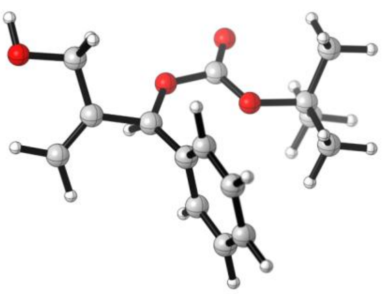

Int 4

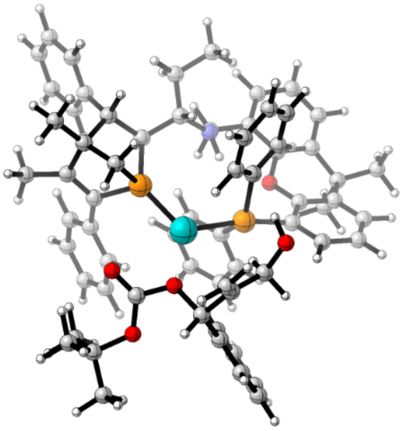

Int (3+4)'

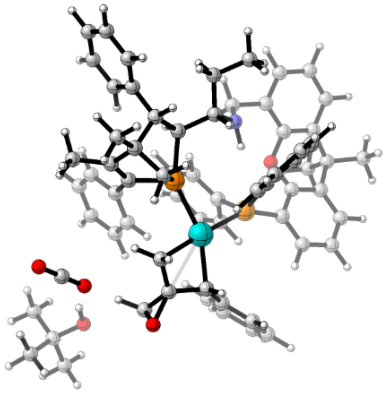

TS 2'

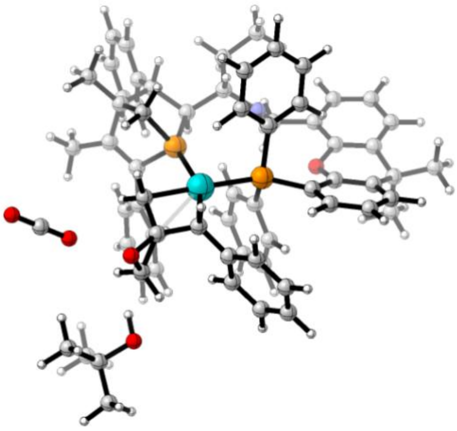

Int (5+6)'

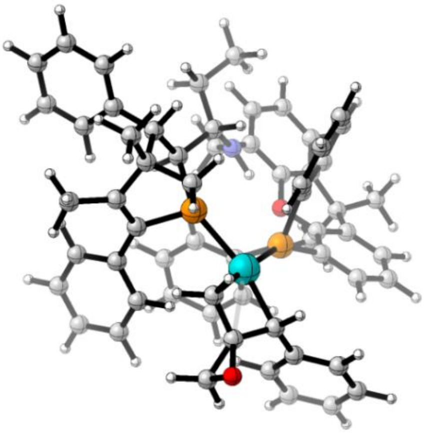

Int 6'

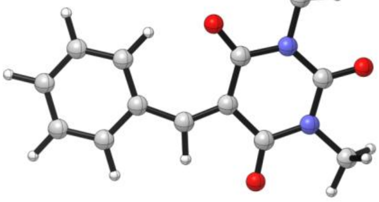

Int 7

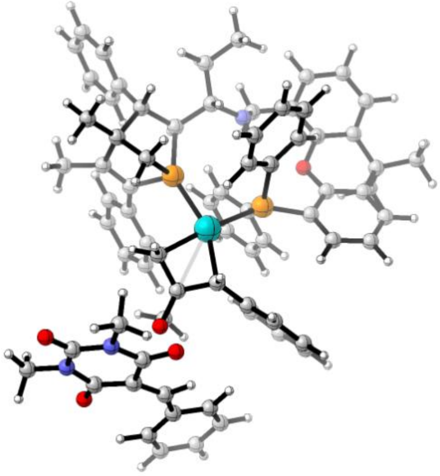

Int (6+7)'

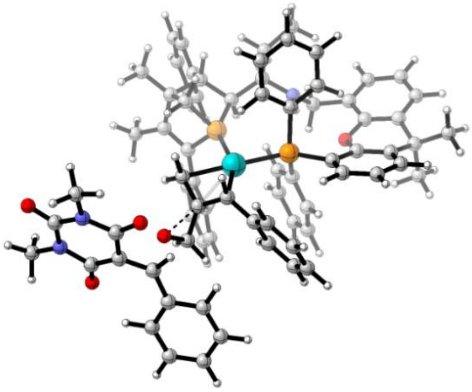

TS 3'

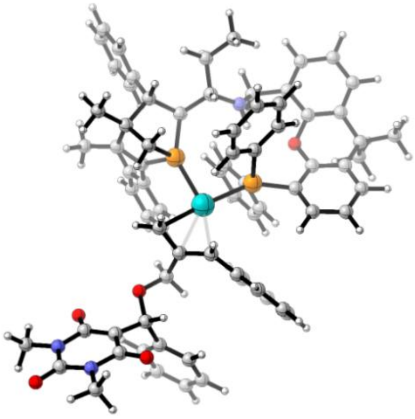

Int 8'

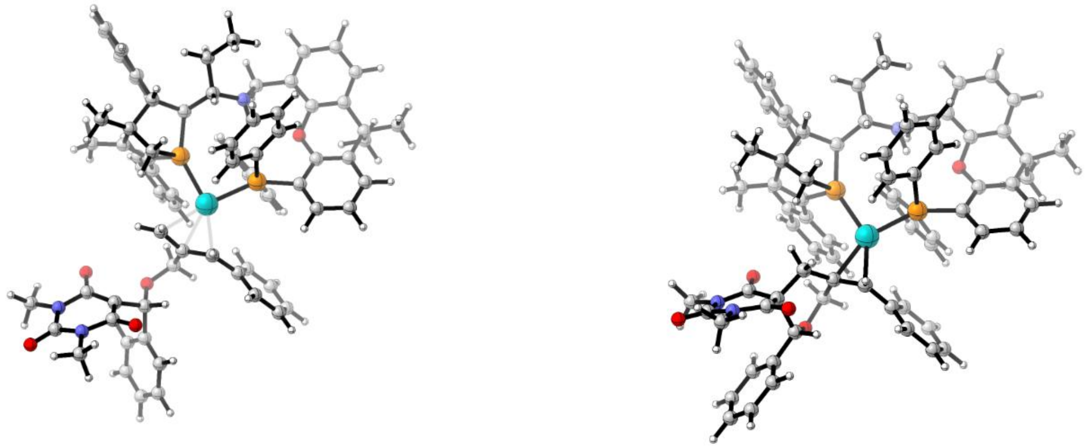

TS 4' **Int 1'**

Figure S7. DFT-optimized configuration of reaction intermediates without DNB during the reaction.

Coordinates and their Gibbs energy with the DBN

Int 1 -780.8933576 a.u.

C 10.014351728 15.955445652 12.106588104

C 10.909616625 16.007594758 9.681899478

C 10.189385394 16.838428866 10.790688690

C 11.394021805 15.293144397 12.216132364

C 9.056452654 14.813437891 11.774460736

C 9.599538630 13.935591206 10.882944171

C 8.903811099 12.903286412 10.097303369

C 7.681777126 14.762482284 12.370167215

C 7.562708529 13.079847721 9.683731458

C 6.904765762 12.108106162 8.928067686

C 7.567386734 10.932707873 8.556679763

C 8.904230312 10.757431609 8.925301067

C 9.563423823 11.734204007 9.670823630

C 9.647931784 16.806049751 13.322546488

C 8.910283942 17.521764158 10.354735854

C 7.958141594 16.886951361 9.540888687

C 6.764498631 17.526774275 9.193808038

C 6.499009955 18.822652680 9.647570139

C 7.440610179 19.472109988 10.451409563

C 8.630552000 18.825917231 10.797936976

C 12.029473387 16.846073148 8.955252246

C 12.209333450 16.209720435 6.430223514

C 13.365953485 16.273584078 5.430402764

C 14.369076214 15.285320166 5.329496651

C 15.378066082 15.310266113 4.354318493

C 15.428854137 16.417945308 3.496450051

C 14.477134919 17.434418023 3.587714146

C 13.449990004 17.345189501 4.531199700

C 15.509175660 13.565987901 6.540406452

C 16.539217266 13.501484608 5.595740037

C 17.717576556 12.836793126 5.970781937

C 17.844740960 12.235644970 7.225576070

C 16.793356463 12.300019882 8.145847853

C 15.614648985 12.978760997 7.822637608

C 16.272304527 14.075428248 4.203951416

C 17.574403154 14.390164072 3.449654802

C 15.465896287 13.009558930 3.403024476

C 12.924190366 12.661377252 7.834216199

C 14.776730207 14.975393025 9.479100505

C 14.668325767 15.271642825 10.851724144

C 15.188477801 16.450033898 11.392596749

C 15.827463482 17.375257281 10.565676737

C 15.971714844 17.087979152 9.203869057

C 15.470032560 15.899228506 8.669944287

C 13.229810336 11.405207488 7.267059876

C 12.321655198 10.752232921 6.429800624

C 11.087934135 11.336335577 6.128493591

C 10.764780098 12.566366596 6.704894379

C 11.665946572 13.209329286 7.556396979

C 11.545538964 18.261223194 8.565514415

C 12.683685578 19.146931034 8.040945610

P 11.373893596 14.359781610 10.639515327

P 14.249768368 13.235620081 9.030995611

N 12.679025630 16.228798671 7.805887171

O 14.325594022 14.239303022 6.248448534

Pd 12.919814714 12.568236022 10.806518362

H 10.187792852 15.678809771 8.923427963

H 10.891769195 17.624648697 11.108155386

H 11.480542417 14.619762206 13.075168581

H 12.221080165 16.014505151 12.244966420

H 7.211398086 13.786200828 12.205546439

H 7.028893933 15.545612986 11.952471371

H 7.731427192 14.939570941 13.454277917

H 7.039900096 13.997452322 9.939596767

H 5.873901760 12.273509870 8.621902631

H 7.052990447 10.173354723 7.971877521

H 9.446836145 9.866309133 8.617170267

H 10.619483952 11.613034269 9.913449550

H 9.605828205 16.191008135 14.231481678

H 8.676061571 17.302313547 13.200781138

H 10.404650782 17.586020651 13.483945977

H 8.148726534 15.879981995 9.175689866

H 6.043488919 17.011804791 8.562109081

H 5.572823890 19.322386288 9.372544348

H 7.251380068 20.483191060 10.805861600

H 9.361600351 19.341188900 11.419831681

H 12.836273227 16.987154771 9.693203069

H 13.216875591 15.408281629 8.044061537

H 11.574617154 17.086330686 6.254312262

H 11.592167976 15.325869074 6.184312924

H 16.206197613 16.480424738 2.740174602

H 14.525590994 18.286926237 2.914454833

H 12.695049806 18.127795919 4.584764036

H 18.543902253 12.774601176 5.267599393

H 18.762538442 11.712765348 7.484102952

H 16.879941063 11.829197851 9.124024211

H 18.179196672 13.483462070 3.325213716

H 18.177428993 15.142303950 3.975012287

H 17.358512931 14.759539761 2.439518354

H 15.209936570 13.396283458 2.407604047

H 14.534124218 12.749128853 3.922259888

H 16.060495448 12.093610777 3.285607896

H 14.225479841 14.543493587 11.533820669

H 15.114413628 16.599565063 12.467916557

H 16.232297113 18.298126053 10.977066082

H 16.481842818 17.788525861 8.546189140

H 15.625259631 15.714114457 7.614071327

H 14.183791832 10.928519144 7.481092113

H 12.588267665 9.783814927 6.010826421

H 10.382910253 10.833090685 5.470937159

H 9.795752973 13.023472543 6.514962863

H 11.387188630 14.158387831 7.996402035

H 11.093757888 18.746997749 9.437046250

H 10.729269914 18.189949782 7.831741402

H 13.484400547 19.228500369 8.789262677

H 12.324060356 20.160262239 7.818138562

H 13.144017518 18.736933027 7.134043413

C 14.883515669 14.300996004 15.297262781

C 16.332928501 13.445714441 13.649098026

C 16.733635768 12.730207561 15.959871647

C 15.998668773 14.026454714 16.320537932

C 18.031410060 11.831348162 13.957851945

C 18.149881882 12.326014101 12.500852215

C 16.845051816 13.114609061 12.260184738

C 12.253617401 11.163414626 12.417576877

C 13.636570576 11.003988773 12.104321271

C 14.302638748 9.905884262 11.366092614

C 15.510659228 9.394799783 11.884285560

C 16.233351920 8.407128981 11.210008020

C 15.761383622 7.894287272 9.998000297

C 14.560824784 8.386086050 9.470785701

C 13.845494361 9.379050686 10.140833446

C 9.888895003 11.209840569 17.345873322

C 12.043660405 11.266050938 16.107886449

C 11.296437923 11.059681259 14.810204090

C 9.797082386 11.251716051 14.883116669

C 7.737288873 11.025156260 16.231558123

C 11.984396240 11.455854761 18.566739196

C 11.877344075 11.957496510 13.657538338

C 11.585078973 9.519286661 14.396353795

C 11.207411639 10.138920583 12.068949907

C 11.152013081 8.529008966 15.454486513

C 12.039583604 8.170318584 16.480818130

C 11.628737493 7.316084791 17.508699106

C 10.328993561 6.800069269 17.513963856

C 9.447059327 7.136338801 16.481105234

C 9.854800918 7.995845420 15.456970525

N 15.277012960 14.149817148 13.889533882

N 17.173327877 12.839476767 14.575113563

N 9.200865413 11.115785225 16.138726112

N 11.286020658 11.366127591 17.276402558

O 9.291504982 11.196256071 18.416015836

O 9.111249475 11.457041889 13.885995609

O 13.277492870 11.289043166 16.145223583

O 10.940241267 9.205390767 13.190106257

H 14.038701985 13.621848202 15.492105985

H 14.497569084 15.322162981 15.430232833

H 17.613908748 12.576871055 16.599935725

H 16.063337313 11.862996172 16.089220463

H 16.719418315 14.855769394 16.321063396

H 15.580277236 13.950986769 17.334177901

H 18.999350151 11.778262812 14.475378287

H 17.568756872 10.829926738 14.011056444

H 18.278854254 11.496028804 11.796225332

H 19.019416062 12.987616929 12.405717903

H 16.084949497 12.515637749 11.737498496

H 16.985782744 14.025200713 11.665973391

H 14.310825672 11.519093471 12.796635107

H 15.883785455 9.789504778 12.828731306

H 17.166009771 8.038818474 11.632839278

H 16.324685385 7.131278663 9.465731335

H 14.188916047 8.010304155 8.519402748

H 12.956078845 9.802851429 9.678161727

H 7.477223565 10.203371310 16.905919179

H 7.360034771 10.845445123 15.221722167

H 7.315585677 11.956832100 16.626089357

H 12.028894966 10.466281608 19.038936804

H 11.437806707 12.139013783 19.222427610

H 12.996561252 11.816760126 18.369423487

H 12.751846628 12.506369697 14.024778784

H 11.086724615 12.671353219 13.402850415

H 12.683805318 9.500194532 14.296204466

H 10.245836790 10.621058968 11.863505677

H 11.494936895 9.504244396 11.224498555

H 13.051089844 8.573020233 16.475964325

H 12.325998441 7.048124106 18.299648100

H 10.007920793 6.134441419 18.312357056

H 8.438528299 6.728279438 16.473327288

H 9.178056322 8.259530274 14.646972041

Int 2 -227.5397093 a.u.

N 11.548615307 11.803438887 16.752185300

N 10.217168622 10.174616756 15.628092097

C 10.274104711 11.278811734 16.492175846

C 11.302000792 9.640336333 14.914732798

C 12.689180691 10.140712812 15.263497111

C 12.743027104 11.308756433 16.243070260

C 11.588389197 12.933655888 17.695063941

C 8.900393435 9.573448848 15.366806081

C 13.457304389 10.571559941 13.972443512

C 13.517912530 8.945125032 15.943271883

C 14.607595614 8.328909342 13.904767789

C 12.761843388 8.371202550 17.120635669

C 12.886179550 8.964886364 18.385917160

C 12.113378663 8.510557088 19.458902769

C 11.217502802 7.452100848 19.279046031

C 11.107709439 6.842912375 18.024628208

C 11.874067476 7.298989500 16.947974938

C 13.939483956 9.426008888 13.125077032

C 13.787874347 9.446349264 11.782987623

C 14.298742555 8.491268815 10.791443433

C 15.556282362 7.864272377 10.903566781

C 15.995891430 6.956732163 9.936484363

C 15.196856423 6.665114749 8.827007692

C 13.958435980 7.301197311 8.684023000

C 13.519864412 8.207820068 9.649282213

O 9.285495078 11.792341159 17.007629942

O 13.818535835 11.805000877 16.577194392

O 11.111171086 8.774264294 14.065475610

O 13.797210326 7.905452652 15.023281902

H 11.226930276 12.608207060 18.676830625

H 10.940503699 13.737078967 17.330729368

H 12.626686292 13.265822030 17.759690610

H 8.644464365 9.680154060 14.307007737

H 8.168219516 10.086736192 15.993796341

H 8.944160068 8.506408892 15.612365318

H 12.837350931 11.259207272 13.381241450

H 14.331463563 11.140261596 14.329711003

H 14.452229851 9.424917095 16.283492395

H 14.746413228 7.425400600 13.300840943

H 15.593831370 8.659350652 14.281758851

H 13.586907755 9.786382694 18.527297226

H 12.216418902 8.978863360 20.435280475

H 10.616167507 7.097770026 20.113625180

H 10.423109696 6.009171453 17.882884575

H 11.790982200 6.837209334 15.967026210

H 16.210186302 8.120749768 11.733902448

H 16.971487441 6.487211901 10.043380866

H 15.541398304 5.960111762 8.073596077

H 13.334774620 7.090777541 7.817693686

H 12.553627271 8.696293074 9.535716631

H 13.191754510 10.262717503 11.369453065

Int (2+3) -780.9009593 a.u.

C 10.174898382 16.721142616 12.274158735

C 10.844930170 16.133729325 9.860781284

C 10.260309841 17.253174008 10.775611487

C 11.530639445 16.017042894 12.432481894

C 9.148104664 15.591107735 12.305057116

C 9.585517466 14.485469359 11.640599926

C 8.805320495 13.289369402 11.282883557

C 7.821183262 15.781437693 12.978458353

C 7.457520228 13.379388328 10.870734536

C 6.724466613 12.235735872 10.543134409

C 7.317402628 10.970860379 10.617059817

C 8.660526479 10.866694893 10.994807773

C 9.394342580 12.009948911 11.307053434

C 9.952692778 17.856306511 13.274358147

C 8.969760672 17.881204253 10.291898269

C 7.920430442 17.120300942 9.748625316

C 6.712810496 17.717120646 9.374671309

C 6.528926878 19.095583656 9.526186526

C 7.568412526 19.870515902 10.049372518

C 8.772442986 19.266635379 10.424654541

C 11.960956094 16.647046555 8.879185905

C 11.542564654 15.459856459 6.632126210

C 12.401512288 15.306286138 5.380546958

C 13.358094125 14.279274874 5.239290295

C 14.084840773 14.067657717 4.058809469

C 13.908014894 14.988259143 3.014844047

C 13.005644018 16.045702735 3.134792108

C 12.245533838 16.182024860 4.300482179

C 14.694138591 12.688284621 6.434432445

C 15.450014683 12.388219804 5.293964808

C 16.626544037 11.643677470 5.477499731

C 17.005679384 11.194012438 6.743152247

C 16.214097684 11.486222488 7.858525674

C 15.052238533 12.254038289 7.732434571

C 14.897814045 12.777715021 3.921373283

C 16.011142608 12.910238452 2.867594695

C 13.912801638 11.652936946 3.480976432

C 12.439985850 12.141709035 8.422524367

C 14.728385480 14.481007567 9.283912408

C 14.878763134 15.030432265 10.574714564

C 15.550328413 16.233946574 10.798307551

C 16.100519014 16.937402207 9.721826588

C 15.981307470 16.405916882 8.435822071

C 15.317704383 15.193265456 8.221386989

C 12.547980599 10.848586775 7.866307134

C 11.447498203 10.220965250 7.277319343

C 10.194988101 10.841262844 7.260829316

C 10.063647244 12.101390365 7.848084415

C 11.171264978 12.736717905 8.416440634

C 11.626582324 18.026479133 8.261109865

C 12.758815208 18.571642279 7.381682675

P 11.343665456 14.737166952 11.125561781

P 14.036005370 12.721196133 9.239131599

N 12.344888793 15.723645488 7.818378655

O 13.537697363 13.453177668 6.336304568

Pd 12.731158466 13.032923847 11.366816533

H 10.048371415 15.694216337 9.247061463

H 11.011986267 18.053798040 10.838654853

H 11.648740791 15.537065567 13.411466838

H 12.387651184 16.677189388 12.241374925

H 7.267441507 14.838677104 13.055931064

H 7.196096164 16.505808711 12.431986676

H 7.957593592 16.188351459 13.991237036

H 6.987674875 14.357146400 10.793251316

H 5.688197223 12.334373412 10.226185592

H 6.744805846 10.079227104 10.370892261

H 9.139046945 9.890600393 11.036320729

H 10.459619614 11.946424194 11.546969934

H 9.967404217 17.479878564 14.305989957

H 8.995299766 18.369250841 13.112297637

H 10.751894123 18.604767492 13.182493419

H 8.047539213 16.047116905 9.623045788

H 5.915796207 17.103537250 8.959643911

H 5.591425030 19.561204707 9.230569071

H 7.444434512 20.945494905 10.162818922

H 9.578212103 19.878022172 10.829110211

H 12.878972649 16.785046043 9.474476907

H 12.819072190 14.897525488 8.162044250

H 10.860085278 16.301609659 6.463942560

H 10.908803319 14.560810928 6.729494675

H 14.466425603 14.867770032 2.090711733

H 12.880006766 16.749358960 2.315472160

H 11.518835208 16.988388990 4.385092164

H 17.245378617 11.397994534 4.618644833

H 17.913737229 10.607676524 6.863607939

H 16.501719412 11.122954656 8.841721294

H 16.555685421 11.964967933 2.754360471

H 16.729445826 13.696167454 3.135146060

H 15.589435734 13.144026918 1.882375211

H 13.453435616 11.903502566 2.515308332

H 13.111836960 11.524057631 4.221480374

H 14.446594904 10.697882539 3.383984245

H 14.439673479 14.486363671 11.418948923

H 15.646607554 16.613921479 11.813696512

H 16.622828563 17.878367962 9.883729999

H 16.403744083 16.934320425 7.583625312

H 15.267695836 14.820091111 7.206272691

H 13.499125522 10.323011673 7.888543316

H 11.576921766 9.234347213 6.835692557

H 9.337332848 10.348705166 6.808394894

H 9.095136467 12.597159418 7.877525788

H 11.044116539 13.720215174 8.849983785

H 11.422728091 18.743810021 9.064988597

H 10.685357094 17.966335175 7.695413347

H 13.696367016 18.633837756 7.951686271

H 12.519853647 19.576803573 7.009626565

H 12.956829102 17.921733624 6.521164289

C 14.479968844 12.853626719 13.992113335

C 13.034861698 10.999449932 13.766387290

C 14.258967994 11.223329305 15.899532993

C 15.238035919 12.034793019 15.044429578

C 12.428121518 9.477544757 15.470724770

C 11.808900886 8.954910898 14.157583843

C 11.943329890 10.140976279 13.180212590

C 11.111709638 6.386575715 11.582640292

C 13.170194410 7.523609772 10.779895777

C 14.029316669 6.615623997 11.641204617

C 13.279794235 5.637391749 12.536448053

C 11.145668869 4.695217688 13.335675939

C 11.007190021 8.067531903 9.794569032

C 14.988117692 7.451626684 12.544877904

C 14.954661687 5.724227775 10.685971272

C 16.788815901 7.290988034 10.803715071

C 14.113326377 4.955338150 9.689725186

C 13.570420461 3.712215923 10.047723642

C 12.707335432 3.040461295 9.176372832

C 12.387433874 3.601144366 7.935794020

C 12.940812225 4.832463501 7.568006214

C 13.799324685 5.509112682 8.439311108

C 16.053408492 8.172829355 11.771827588

C 16.261358072 9.490249641 11.991254091

C 17.267508008 10.402762443 11.445465968

C 18.560328606 10.011863322 11.038820450

C 19.454005574 10.946073153 10.509031403

C 19.086895206 12.291427861 10.389571244

C 17.818550815 12.701751139 10.814419428

C 16.925693819 11.769325952 11.341214581

N 13.532454997 12.054682037 13.189834649

N 13.365725826 10.501540555 15.005583344

N 11.889870099 5.639030304 12.487390210

N 11.791056213 7.289153220 10.765395443

O 9.893352678 6.248645839 11.546963604

O 13.888912949 4.865956472 13.281526134

O 13.633730417 8.415947193 10.076706398

O 15.885574634 6.514065284 9.978818279

H 13.915347953 13.670776455 14.467431871

H 15.192906674 13.328361935 13.305830840

H 14.789552608 10.495691396 16.530460317

H 13.682332517 11.883106952 16.569015446

H 15.938177043 11.351857271 14.543127891

H 15.834896876 12.699028940 15.684621725

H 12.961345225 8.699692129 16.034284525

H 11.667922369 9.917864755 16.136493568

H 10.770001760 8.630284563 14.286801633

H 12.379254738 8.089965558 13.802922303

H 11.022842807 10.740462037 13.146780833

H 12.183426441 9.861194730 12.145995927

H 10.721058546 3.893081323 12.720645630

H 10.328966364 5.225033098 13.835059286

H 11.851203437 4.281310146 14.059530274

H 11.223313963 9.134565790 9.907329371

H 9.950525294 7.854585475 9.962926148

H 11.297694022 7.770838624 8.781053092

H 14.415438452 8.160508040 13.157092073

H 15.451999568 6.723499218 13.230085731

H 15.465456319 5.029078687 11.375116765

H 17.378261626 7.872690354 10.086433696

H 17.462765407 6.598835338 11.342454163

H 13.822582140 3.273906440 11.012065822

H 12.291389312 2.077258591 9.463815142

H 11.716469067 3.078430162 7.257338246

H 12.703329747 5.268447878 6.599846921

H 14.230098855 6.469504025 8.165865547

H 18.881055806 8.981535495 11.171704169

H 20.446399969 10.624131152 10.200108610

H 19.784776726 13.013981490 9.971989113

H 17.510680206 13.741385510 10.713443198

H 15.919618732 12.078193859 11.620797109

H 15.540202215 9.975898954 12.652836623

Int 3 -553.3552302 a.u.

C 9.386186302 17.257642246 11.103447943

C 10.670718763 16.864363797 8.905183536

C 9.820142905 17.895009356 9.710244426

C 10.694992171 16.596950774 11.558730363

C 8.448720871 16.086801777 10.812917079

C 9.098860636 15.049776227 10.214980418

C 8.502700716 13.850751862 9.604216554

C 6.989963494 16.170788157 11.153496264

C 7.281115152 13.908956522 8.897002824

C 6.725168724 12.764207173 8.320744994

C 7.377116352 11.530882979 8.427678747

C 8.603201305 11.461189793 9.097230551

C 9.158694019 12.605819767 9.666592666

C 8.855665811 18.313085210 12.074238119

C 8.663595180 18.512593936 8.955691308

C 7.805917659 17.750976010 8.144385896

C 6.699413537 18.332006348 7.518307206

C 6.432520943 19.696018039 7.678061210

C 7.286590319 20.472418647 8.467281421

C 8.387139294 19.883160401 9.097001641

C 11.962169958 17.494626624 8.264050650

C 12.206627783 16.426139453 5.934436220

C 13.363640101 16.315446988 4.947044117

C 14.334342710 15.294734252 5.016357198

C 15.351967510 15.142117024 4.062245565

C 15.445417089 16.106149394 3.047967994

C 14.525494658 17.152898890 2.969424657

C 13.486416778 17.237243539 3.900479335

C 15.377839610 13.720721320 6.483937727

C 16.419260531 13.490288287 5.574208429

C 17.570292260 12.846411770 6.053374951

C 17.661858720 12.426008114 7.381579807

C 16.595900966 12.647788631 8.258522333

C 15.436296537 13.305440159 7.833713195

C 16.198035868 13.867896721 4.109417394

C 17.521945337 14.021445121 3.342581348

C 15.363001515 12.726213682 3.456116358

C 12.752966092 12.997212055 7.898793369

C 14.623204505 15.459439050 9.301044163

C 14.406386763 15.977285973 10.595889715

C 14.912892089 17.216049677 10.992296992

C 15.661448621 17.984218988 10.093935311

C 15.909675551 17.482275026 8.814557220

C 15.410529356 16.234290278 8.427667137

C 12.999575445 11.648292429 7.562146736

C 12.092936277 10.917062638 6.787679599

C 10.918579666 11.513162295 6.319963600

C 10.656578581 12.843927507 6.656190829

C 11.554930123 13.565491645 7.444063707

C 11.700956076 18.888537503 7.644349369

C 12.978522322 19.544659523 7.105993212

P 10.912484653 15.407648328 10.172546598

P 14.071281777 13.676573371 9.058471668

N 12.672624186 16.666117143 7.294165153

O 14.235311298 14.408204847 6.079547436

Pd 12.258055672 13.718503981 10.669701599

H 10.072220775 16.437272249 8.090142356

H 10.492528797 18.712921910 10.009323793

H 10.589432768 16.052780545 12.505330561

H 11.534527153 17.301727967 11.631535867

H 6.492092986 15.200304387 11.045593950

H 6.469656900 16.899381486 10.511027564

H 6.854716060 16.516755359 12.188807790

H 6.775079975 14.864657871 8.782656811

H 5.784221883 12.838273746 7.779308337

H 6.943412512 10.640184250 7.978224687

H 9.143405526 10.518241476 9.156250503

H 10.144614343 12.570309589 10.140524078

H 8.633385165 17.869409201 13.054046287

H 7.941228486 18.794306192 11.702484129

H 9.607046459 19.099712522 12.228695416

H 8.001855253 16.689459800 8.008037504

H 6.047569408 17.718029217 6.900139228

H 5.574072481 20.149314918 7.187412871

H 7.097851905 21.536615703 8.592119941

H 9.047937606 20.494175664 9.710687883

H 12.686886742 17.646240995 9.080693361

H 13.090309871 15.843183636 7.711897505

H 11.582727146 17.270254044 5.616931751

H 11.579109526 15.522476158 5.834838026

H 16.231611354 16.029477453 2.302078908

H 14.608282565 17.891667354 2.175933365

H 12.753799109 18.039183333 3.826310841

H 18.401365665 12.660906406 5.378375252

H 18.560798098 11.922786131 7.730041835

H 16.656817629 12.321862267 9.295580848

H 18.090194595 13.083257247 3.356655604

H 18.148939658 14.815099886 3.769874128

H 17.334896488 14.255214066 2.287242928

H 15.137525408 12.969079300 2.409079336

H 14.413944303 12.580252003 3.989009079

H 15.922268722 11.781460463 3.489195572

H 13.818123096 15.376856446 11.295243711

H 14.727633725 17.574031494 12.003155528

H 16.056501847 18.953757662 10.390939765

H 16.492153319 18.062761105 8.102142021

H 15.639398140 15.883768884 7.428192797

H 13.913784227 11.166236982 7.903065171

H 12.312456129 9.877879962 6.550608471

H 10.212281137 10.947426691 5.716799173

H 9.735189657 13.321066628 6.327870444

H 11.320320329 14.588569826 7.710123095

H 11.255764126 19.544437361 8.401791937

H 10.936767801 18.813256188 6.856856940

H 13.736452483 19.623193925 7.897849057

H 12.774127156 20.555534357 6.728893378

H 13.430373313 18.957842308 6.297433297

C 12.955438547 13.634023490 13.677125141

C 11.557339495 11.811524859 13.134199171

C 11.835269699 12.353172555 15.532404509

C 13.153076744 12.963858394 15.043403558

C 10.350249810 10.439784684 14.643632154

C 10.413121042 9.709376204 13.286629083

C 10.776746112 10.832190235 12.294034531

N 12.319661285 12.759933703 12.672527739

N 11.297115845 11.538497554 14.455509623

H 12.344402418 14.544768782 13.780276122

H 13.924750888 13.953598541 13.271527559

H 11.994039347 11.713535099 16.412700020

H 11.120314213 13.140897159 15.821423776

H 13.905696047 12.168374281 14.954936435

H 13.528144135 13.694031404 15.773644125

H 10.659841581 9.809601206 15.489161343

H 9.338882883 10.822059768 14.858167101

H 9.472362319 9.204390374 13.039058265

H 11.209121381 8.954284893 13.312775259

H 9.873647626 11.337425550 11.920989522

H 11.355094840 10.508913816 11.419641292

Int (3+4) -714.3947451 a.u.

C 9.615010282 16.379510794 12.103439343

C 10.331796038 16.410967825 9.629113855

C 9.702086008 17.257820837 10.778086374

C 10.993611081 15.704150574 12.114233551

C 8.632848846 15.237737167 11.846867867

C 9.117865801 14.341994854 10.942414322

C 8.389826593 13.239569309 10.293788539

C 7.299147630 15.205795591 12.533338392

C 7.034036082 13.367061237 9.917772273

C 6.353442200 12.313912156 9.301923614

C 7.009506779 11.106835477 9.037151512

C 8.360575178 10.974510843 9.374116555

C 9.039068450 12.028679126 9.983459859

C 9.335888078 17.235686289 13.339117183

C 8.399136495 17.946848116 10.437604749

C 7.369864040 17.298553922 9.734609786

C 6.146341285 17.932035820 9.498781657

C 5.928466739 19.238408414 9.949561226

C 6.948949988 19.904820554 10.634842003

C 8.167553005 19.262388332 10.874150198

C 11.432179097 17.190225480 8.817915097

C 11.106516638 16.537439084 6.348837994

C 11.991587007 16.639461644 5.111051530

C 12.948398255 15.660721402 4.771195498

C 13.706108720 15.710363430 3.591114238

C 13.554559648 16.833558708 2.765090345

C 12.644864903 17.841499699 3.088542461

C 11.860646091 17.727616500 4.239929080

C 14.311367370 13.905660934 5.655657507

C 15.102703579 13.872155843 4.498968816

C 16.333953376 13.203125764 4.572957040

C 16.741729354 12.570194407 5.748698695

C 15.918667994 12.599696756 6.878380213

C 14.692210426 13.272855222 6.860765372

C 14.536117227 14.480678767 3.216073258

C 15.636795930 14.812663360 2.194974076

C 13.566102860 13.432423270 2.593371632

C 12.102850224 12.852292265 7.507172991

C 14.260542713 15.116889798 8.827257049

C 14.362838426 15.401617475 10.205758448

C 14.952336795 16.575542197 10.677578655

C 15.464143170 17.512271401 9.773107918

C 15.396087993 17.241860569 8.404671174

C 14.816005064 16.057549406 7.938615751

C 12.258484776 11.589606829 6.894690615

C 11.190619089 10.963144082 6.243769546

C 9.939391901 11.582270629 6.178047781

C 9.768681236 12.826534400 6.790660958

C 10.831710979 13.440903278 7.454703591

C 11.032237788 18.656799924 8.527301509

C 12.143937046 19.443771936 7.822107365

P 10.868647787 14.773302292 10.532729720

P 13.662913703 13.379421899 8.419684956

N 11.886612088 16.564557984 7.579415243

O 13.106458432 14.605462048 5.658641138

Pd 12.290968162 13.079267121 10.397886542

H 9.553846336 16.103078545 8.918552378

H 10.428320191 18.040023367 11.045060745

H 11.117949107 15.005410621 12.950665553

H 11.827382853 16.419400700 12.105724108

H 6.788015060 14.248179488 12.381445019

H 6.640930210 16.010548450 12.168022248

H 7.417795533 15.367890442 13.614774042

H 6.517256510 14.307662191 10.093044631

H 5.309975850 12.440358754 9.020481431

H 6.478590749 10.287544942 8.557210487

H 8.897225622 10.057087606 9.140030268

H 10.110011757 11.953295230 10.196317894

H 9.355131504 16.625768758 14.252304693

H 8.359808545 17.735848785 13.283650925

H 10.103916810 18.014409417 13.443685918

H 7.525106058 16.283442583 9.375099046

H 5.363636056 17.404972391 8.956954193

H 4.978052563 19.733185183 9.762327530

H 6.797929986 20.924784632 10.982100487

H 8.957864303 19.787638384 11.409057013

H 12.332473487 17.231465092 9.452640202

H 12.390938463 15.700450040 7.738014028

H 10.426209964 17.397479830 6.338675273

H 10.471681100 15.639133314 6.247167459

H 14.137922176 16.914000345 1.852101931

H 12.535669728 18.705397148 2.437375261

H 11.133314051 18.501067403 4.480966114

H 16.977723994 13.166259569 3.698359926

H 17.697115938 12.051646962 5.781792171

H 16.226795856 12.107376725 7.799307683

H 16.189856318 13.908564199 1.912077091

H 16.349790033 15.547706123 2.591131150

H 15.201661706 15.212277169 1.270668182

H 13.095687990 13.838089507 1.687692626

H 12.772950103 13.161899873 3.303100349

H 14.115007363 12.518319253 2.329647568

H 13.959037133 14.669785624 10.910763718

H 15.016581905 16.751591073 11.749579580

H 15.921002883 18.433227409 10.130043727

H 15.791089690 17.956004918 7.685121757

H 14.796843143 15.888666642 6.868425788

H 13.226917800 11.093776867 6.920587630

H 11.344547274 9.987831675 5.786206280

H 9.107541454 11.098065095 5.671795329

H 8.796302186 13.315128010 6.777667418

H 10.670082964 14.394900816 7.940793499

H 10.783781381 19.158488602 9.470009112

H 10.100801819 18.684313929 7.942951154

H 13.070355821 19.418484536 8.412675112

H 11.856783384 20.494642662 7.684038217

H 12.386688518 19.018958720 6.840833523

C 13.828710747 12.923374416 13.377225105

C 12.466927195 11.088869760 12.803538618

C 13.595603036 11.010741072 15.003435157

C 14.574470724 11.971727849 14.322230554

C 11.939338545 9.227431243 14.167669477

C 11.476542861 8.902177916 12.733935356

C 11.457708340 10.274731411 12.032720975

C 7.652646699 3.061669587 13.330627004

C 8.616007890 4.248903194 13.351006681

C 9.187939799 4.523058389 14.742775336

C 9.730525240 4.078687051 12.317676947

C 6.867879631 5.982468099 13.520071980

C 5.408507140 9.626650069 11.956783055

C 6.849530685 9.368087444 12.330991137

C 7.349676061 7.949550098 12.140669149

C 7.433075230 7.486134764 10.698194106

C 7.655440116 10.309021614 12.836780709

C 8.658326533 7.576391343 10.023149311

C 8.764917009 7.190718299 8.684078485

C 7.643050813 6.705826004 8.003950236

C 6.420854184 6.598434683 8.676580063

C 6.316837191 6.983213716 10.016889773

N 12.939474900 12.237851743 12.423009465

N 12.758662908 10.421961589 13.970522604

O 7.914520497 5.468745909 12.844691724

O 6.323125988 5.540344233 14.509929979

O 4.951361100 10.948785953 12.232626504

O 6.420515844 7.124725278 12.935103871

H 13.229668658 13.648945410 13.950482691

H 14.561768102 13.506692760 12.806132068

H 14.129139615 10.204480980 15.527336845

H 12.979605946 11.538392506 15.750292651

H 15.304857762 11.390900915 13.742318776

H 15.131842902 12.543499920 15.076756606

H 12.536684161 8.425141843 14.622981083

H 11.087267876 9.435383279 14.836565214

H 10.505615415 8.392258442 12.720153089

H 12.209046535 8.246237921 12.246048318

H 10.480375124 10.773234453 12.115724203

H 11.712287593 10.236526808 10.966925954

H 6.838075341 3.202975344 14.049383140

H 8.199776891 2.145615008 13.590782542

H 7.227340055 2.933218931 12.327196434

H 8.389651811 4.668152920 15.479118029

H 9.826523003 5.415891619 14.725070914

H 9.805079569 3.669909858 15.055344657

H 10.330147315 3.191117171 12.556641020

H 10.392071482 4.954723072 12.312035892

H 9.309847373 3.958941830 11.311103036

H 5.389767999 11.539955585 11.591377638

H 5.261983712 9.366558035 10.894206116

H 4.766779679 8.951712999 12.541622371

H 8.346632538 7.866959122 12.590508866

H 8.679539061 10.081278005 13.122314359

H 7.310815295 11.329750560 12.981462004

H 9.533675523 7.958846501 10.546825773

H 9.723990277 7.265313815 8.174315624

H 7.722697488 6.404858631 6.961508986

H 5.546991212 6.210018958 8.157824327

H 5.371972191 6.876657846 10.545796458

Int 4 -161.016955 a.u.

C 15.958831048 17.686306455 9.270672738

C 15.757516782 16.262552592 9.789530443

C 16.161289656 15.193207947 8.773788497

C 16.468177668 16.048692526 11.126549766

C 13.363121691 16.091793492 9.225419760

C 9.646508918 16.306750859 10.507071243

C 10.415343652 15.217875473 11.217053701

C 11.927179615 15.365969679 11.130381531

C 12.929740189 15.390482826 12.256343583

C 9.817989818 14.222710293 11.882679827

C 13.548293663 14.154291674 12.487589828

C 14.396680036 13.985376255 13.585100121

C 14.622413176 15.045763762 14.470669754

C 13.996156236 16.276839502 14.252011256

C 13.158490555 16.447781537 13.144942537

O 14.322752568 16.060445875 10.166617990

O 13.460467390 16.408003026 8.056287279

O 8.238057386 16.058987981 10.596296533

O 12.153098314 15.709801249 9.714295058

H 15.420553271 17.842319337 8.329295329

H 17.029568845 17.862619932 9.100293625

H 15.606733844 18.416981996 10.009965817

H 15.642178966 15.335408162 7.819622907

H 15.929327498 14.193858689 9.164506026

H 17.243719299 15.251411418 8.597314027

H 17.552432347 16.153948936 10.992724468

H 16.255296063 15.046272220 11.520124961

H 16.136740207 16.789507762 11.865251989

H 7.794671267 16.674083630 9.992436076

H 9.909999666 17.281412409 10.960030221

H 9.973064867 16.344025859 9.457746323

H 11.546269495 14.455834212 10.337382666

H 10.394559367 13.444748839 12.375494068

H 8.733740884 14.177956237 11.938856985

H 13.371269368 13.334317204 11.794295194

H 14.880281940 13.025102445 13.751406333

H 15.282428464 14.912819301 15.325347451

H 14.167487012 17.105936606 14.935312886

H 12.687164511 17.412210068 12.961292340

Int (5+6) -714.3861309 a.u.

C 9.752004862 16.329451954 11.776731625

C 10.850433312 16.272817904 9.450049657

C 10.075203213 17.168936297 10.463213485

C 11.105884983 15.658035368 12.058245238

C 8.823231537 15.180532869 11.383206578

C 9.455280663 14.233576451 10.635399485

C 8.842367360 13.103618007 9.912231299

C 7.379743428 15.190712563 11.788093696

C 7.633621861 13.274993128 9.203940838

C 7.041292537 12.212099533 8.517392246

C 7.642767980 10.949141761 8.521445568

C 8.851552590 10.768365256 9.201390521

C 9.448673068 11.831884162 9.877365444

C 9.265642138 17.213911555 12.924494779

C 8.865745181 17.884133906 9.895863682

C 7.969901811 17.266529164 9.008842569

C 6.840635040 17.941699493 8.535807932

C 6.583866936 19.256310410 8.937472660

C 7.469134212 19.888305475 9.815567263

C 8.595475594 19.207047297 10.285532205

C 12.026615090 17.032321470 8.728831110

C 12.265814184 16.252800164 6.242953195

C 13.465829168 16.245380330 5.291697364

C 14.445551608 15.229154538 5.285491479

C 15.503245871 15.175858835 4.366038701

C 15.626896173 16.240442936 3.461915374

C 14.700524883 17.284415451 3.457768164

C 13.624369870 17.269764537 4.349846684

C 15.437561820 13.494531579 6.608115707

C 16.507379062 13.340072064 5.722146605

C 17.604791582 12.585161539 6.168207209

C 17.608486658 11.984147313 7.429979316

C 16.519555894 12.145266529 8.293557367

C 15.426883129 12.923210032 7.902225970

C 16.357305033 13.903232324 4.307582272

C 17.720710314 14.152372320 3.641833200

C 15.565781001 12.852267483 3.472503477

C 12.699562704 12.817762385 7.788731710

C 14.664814486 15.071700818 9.412855561

C 14.521211388 15.434893841 10.766784600

C 15.088327953 16.606032074 11.276379611

C 15.810601293 17.456249976 10.437647075

C 15.994402015 17.096426047 9.098678025

C 15.444830357 15.915328798 8.595911403

C 12.957002169 11.551700084 7.221966339

C 12.001373437 10.910026449 6.429407542

C 10.765830399 11.511043460 6.176350178

C 10.495406525 12.756952272 6.744093887

C 11.446977440 13.391803507 7.544845893

C 11.623135911 18.446974321 8.256775346

C 12.819705206 19.252167589 7.731637707

P 11.236488943 14.676458369 10.522447291

P 14.025381395 13.352376864 9.003498537

N 12.679736066 16.325015814 7.634527397

O 14.320434774 14.236169440 6.249225943

Pd 12.725735102 12.847392690 10.843660252

H 10.163768668 15.909582970 8.675486751

H 10.777156984 17.939999651 10.817490645

H 11.082271198 14.994988751 12.930085815

H 11.931989200 16.373367032 12.163238155

H 6.884240217 14.244434396 11.542978911

H 6.833093279 16.008857160 11.292856567

H 7.286603730 15.362381424 12.870556247

H 7.163645919 14.255724990 9.183197982

H 6.110237743 12.371965809 7.977469018

H 7.178811704 10.118611105 7.993833635

H 9.340092745 9.796018963 9.199578369

H 10.406139893 11.688577181 10.376206040

H 9.120206499 16.622341097 13.838227874

H 8.318050618 17.714714571 12.686606333

H 10.008396905 17.992394516 13.146747571

H 8.154735807 16.244642297 8.684112893

H 6.162697595 17.439632100 7.848615088

H 5.708500286 19.783814416 8.565064851

H 7.286362576 20.913253643 10.131576360

H 9.284076973 19.709323746 10.963888318

H 12.815691885 17.173193066 9.485486816

H 13.184770914 15.504120191 7.931706781

H 11.657877918 17.132603737 6.000193996

H 11.640802287 15.369503565 6.016309450

H 16.441141774 16.247286946 2.742638909

H 14.807648227 18.101549548 2.748324723

H 12.890327891 18.073418330 4.325956422

H 18.461094566 12.446731930 5.513229099

H 18.459297017 11.381492732 7.739283749

H 16.508196304 11.661213657 9.268540446

H 18.297118839 13.221499693 3.575284889

H 18.314195925 14.890733075 4.196807356

H 17.592121317 14.510500775 2.613053742

H 15.394649944 13.226311373 2.454258649

H 14.590427586 12.641364413 3.930763740

H 16.128505666 11.910795010 3.415106064

H 14.023724694 14.759156904 11.466292998

H 14.978658077 16.816772801 12.338414291

H 16.248934940 18.374674339 10.823993817

H 16.571514336 17.734655719 8.432982607

H 15.633292762 15.676398135 7.557107481

H 13.902721505 11.049723838 7.407670596

H 12.233305013 9.931997082 6.012008164

H 10.021228558 11.011547484 5.561029325

H 9.529202589 13.232743641 6.590078247

H 11.209542567 14.358720546 7.966833739

H 11.166628731 18.992086996 9.090521690

H 10.829233120 18.378603762 7.498718579

H 13.598636052 19.332870557 8.502755615

H 12.516756133 20.268950453 7.448631329

H 13.288224969 18.776773058 6.861682042

C 14.509925905 13.809881001 15.078267744

C 16.161060427 13.432776100 13.448288038

C 16.509219764 12.388125539 15.645280759

C 15.584106842 13.508079667 16.136384061

C 18.005799304 11.958547622 13.627809592

C 18.152819973 12.695088415 12.279877681

C 16.784470886 13.374857312 12.067819862

C 11.392215137 9.710978874 12.381535351

C 12.203874925 10.930688779 12.499437329

C 13.537187047 11.146122039 11.844996767

C 14.191584396 10.139734833 10.984238733

C 11.630186392 12.301373639 12.581882092

C 15.539550621 9.788868289 11.211971317

C 16.206262241 8.872333273 10.394913181

C 15.539204142 8.270364550 9.322497373

C 14.198536524 8.600121287 9.084223096

C 13.537057420 9.520585605 9.898235143

C 9.311211302 3.927519365 10.525397163

C 8.906729592 4.511170391 11.886465529

C 7.656428054 3.816518512 12.430078287

C 10.064939193 4.432613344 12.889779360

C 7.605038332 7.707415460 13.482528116

N 15.023862485 13.983554437 13.713275844

N 16.999453168 12.754355838 14.324230479

O 12.067035270 9.978628608 13.635444780

O 8.509504914 5.903502746 11.713872987

O 7.898419803 8.591392486 12.762057374

O 7.290503279 6.862140961 14.238508757

H 13.762668993 12.998908074 15.059802594

H 13.960645839 14.722144622 15.352255306

H 17.368092811 12.256814504 16.319181794

H 15.968326113 11.426754765 15.606090728

H 16.184639627 14.409693992 16.319341360

H 15.120711381 13.221528590 17.090934480

H 18.940275730 11.926183660 14.205271974

H 17.663474342 10.917506820 13.490675719

H 18.418403438 12.012263790 11.464063532

H 18.945513628 13.449351262 12.356159117

H 16.118693335 12.781375449 11.423910881

H 16.853709045 14.370071425 11.614972277

H 14.233719528 11.605931891 12.557572093

H 10.300774745 9.791729080 12.403748265

H 11.783576996 8.856109854 11.821823034

H 12.074206058 12.899889570 13.386105010

H 10.541380957 12.392232545 12.553860426

H 16.060487137 10.242585933 12.054058927

H 17.246042754 8.623789051 10.599642781

H 16.054536632 7.559793219 8.680691612

H 13.669296708 8.149205817 8.246500454

H 12.511756855 9.807718276 9.670613228

H 9.566348801 2.862113704 10.612227124

H 10.191242652 4.448051475 10.121198600

H 8.488109111 4.031795575 9.806981585

H 6.828416814 3.909558433 11.715874235

H 7.348023568 4.275691984 13.377783345

H 7.851078985 2.750295698 12.602677946

H 10.361000920 3.390374042 13.072719984

H 9.769866769 4.884955657 13.845627296

H 10.946818039 4.969571360 12.512302631

H 9.263699171 6.382913447 11.330555315

Int 5-1 -37.74796777 a.u.

C 13.194316110 15.277700836 10.980890289

O 13.623271504 16.305601981 10.594813829

O 12.765408546 14.249800530 11.366951534

Int 5-2 -44.61763422 a.u.

C 13.520416808 14.078511510 12.243086237

C 13.173951853 12.583305009 12.293149697

C 13.183696922 11.972957391 10.889667403

C 14.136442478 11.830750514 13.223693554

O 11.806286849 12.412878455 12.759949763

H 14.533856896 14.240079225 11.849826342

H 13.478971571 14.522497785 13.247917546

H 12.807627900 14.613028747 11.601958284

H 12.468615765 12.495852293 10.241727891

H 12.897173108 10.914348028 10.933714746

H 14.182087671 12.046492267 10.439908122

H 15.173231099 11.903240724 12.866363801

H 13.859670116 10.770216624 13.280686628

H 14.104722824 12.250035694 14.239407465

H 11.752206680 12.794835405 13.651719116

Int 6 -632.0180423 a.u.

C 9.738534281 16.335911891 11.766627415

C 10.845578164 16.278577464 9.443453387

C 10.065884838 17.174411383 10.453300119

C 11.092181201 15.665865656 12.053396353

C 8.812560865 15.185053409 11.371348217

C 9.448246453 14.239230403 10.624814156

C 8.842215171 13.112339248 9.891666427

C 7.368420366 15.191857854 11.773379703

C 7.630032713 13.279843683 9.187846210

C 7.055150020 12.223436355 8.477081266

C 7.678853281 10.971594479 8.448737454

C 8.889702463 10.794357684 9.125815365

C 9.467141548 11.850580573 9.829793139

C 9.246160076 17.222810828 12.910448688

C 8.857621502 17.888465971 9.882679126

C 7.958399113 17.266563093 9.001787236

C 6.828939416 17.941096845 8.527800563

C 6.575469663 19.258646920 8.922237354

C 7.463910287 19.894202621 9.794717973

C 8.590717316 19.214090785 10.264930454

C 12.024752299 17.036703602 8.725322956

C 12.268121980 16.258379098 6.240515312

C 13.468129339 16.246964441 5.289605801

C 14.446322918 15.229167113 5.284256696

C 15.502930606 15.173095954 4.363692889

C 15.625973608 16.235010223 3.456212233

C 14.699691906 17.279051731 3.449524218

C 13.625347872 17.267960013 4.343768269

C 15.436430051 13.494733067 6.608585522

C 16.506697595 13.338970937 5.723421479

C 17.602663826 12.582975363 6.171403167

C 17.604464319 11.983295961 7.433982494

C 16.514711013 12.145830792 8.296386724

C 15.423279692 12.924327103 7.902957378

C 16.356442716 13.899907410 4.307798712

C 17.719457691 14.147613979 3.640702149

C 15.563545932 12.847296445 3.475861680

C 12.696292984 12.823527233 7.781797962

C 14.664888709 15.076035317 9.408850302

C 14.523622046 15.438630682 10.763224086

C 15.100248030 16.604616203 11.274408456

C 15.831630688 17.449109192 10.437784847

C 16.010231106 17.091047319 9.097473734

C 15.449319879 15.916319506 8.592298624

C 12.953356290 11.555781294 7.218327624

C 12.001028810 10.916582823 6.419861847

C 10.770675156 11.523049726 6.154732027

C 10.500922936 12.770722851 6.718410316

C 11.447373258 13.401780017 7.528608013

C 11.623849901 18.451721655 8.252310461

C 12.821382658 19.253887649 7.725043517

P 11.229922222 14.683941313 10.517740895

P 14.019961862 13.357205571 9.000412489

N 12.679808326 16.328200668 7.632920307

O 14.320230938 14.236949715 6.248796021

Pd 12.721315172 12.853884146 10.842186505

H 10.161371319 15.914897084 8.666817779

H 10.765958564 17.946165821 10.809897378

H 11.065720533 15.002646713 12.925089925

H 11.917155985 16.382148595 12.161572020

H 6.877345579 14.241677105 11.534399446

H 6.818301857 16.003737744 11.271608990

H 7.272402755 15.371026177 12.854484808

H 7.145400050 14.253568885 9.185230544

H 6.122302996 12.381119335 7.939349503

H 7.232538981 10.148946827 7.894149700

H 9.399103643 9.833028570 9.095599833

H 10.427963776 11.711965737 10.325356293

H 9.102191760 16.634689054 13.826716499

H 8.297007908 17.718934260 12.668978559

H 9.985128779 18.005583269 13.130381073

H 8.140625340 16.242385428 8.682583778

H 6.148767991 17.436924014 7.844641207

H 5.700738312 19.785919842 8.548118844

H 7.284048503 20.921432486 10.105020252

H 9.281478475 19.718879140 10.938973640

H 12.811935599 17.176815500 9.484187691

H 13.183738242 15.506920732 7.931038945

H 11.663649267 17.140456520 5.997856284

H 11.640250388 15.377649300 6.012060330

H 16.438891273 16.238745115 2.735420360

H 14.805389780 18.093132553 2.736367425

H 12.891197737 18.071452998 4.317772324

H 18.459506635 12.442685036 5.517476237

H 18.454269496 11.379972016 7.744867233

H 16.501643258 11.662660032 9.271848225

H 18.297180053 13.217282542 3.578388225

H 18.312032105 14.889456512 4.191992115

H 17.590240554 14.500772860 2.610233828

H 15.388854864 13.219579230 2.457580994

H 14.589776251 12.636273387 3.937514181

H 16.126693651 11.906043524 3.418379539

H 14.021012364 14.765661365 11.461639892

H 14.992179989 16.814809384 12.336617436

H 16.279895889 18.361831692 10.826145094

H 16.593513753 17.724965129 8.433093760

H 15.633776141 15.679032752 7.552350364

H 13.896317426 11.051145848 7.410553470

H 12.231817399 9.936331332 6.006820492

H 10.029059246 11.025821379 5.533988778

H 9.538210518 13.250468576 6.555372968

H 11.209598550 14.369115010 7.949568530

H 11.169110682 18.998559423 9.085949207

H 10.829199392 18.384075641 7.494911373

H 13.606851805 19.323313139 8.490547061

H 12.522362183 20.274763576 7.452645920

H 13.280520152 18.783516479 6.847441762

C 14.538492469 13.847356833 15.092881764

C 16.158478701 13.427419832 13.444476196

C 16.544674151 12.435939088 15.661778326

C 15.635955634 13.576666162 16.136118237

C 17.999435367 11.949155673 13.622021905

C 18.133681866 12.667974262 12.262711145

C 16.765664924 13.350570566 12.057608911

C 11.392561405 9.715547668 12.379729143

C 12.202768546 10.935340827 12.499678479

C 13.535271828 11.153017230 11.844652492

C 14.191539826 10.145780900 10.985755151

C 11.629052755 12.306436416 12.582467091

C 15.536673434 9.788983067 11.221159522

C 16.203965263 8.869611375 10.407521202

C 15.539770485 8.271263233 9.331414678

C 14.202335570 8.607441292 9.085503120

C 13.540457854 9.530891371 9.895869581

N 15.026575901 13.986736405 13.714462823

N 17.004554728 12.758386425 14.319185698

O 12.066864129 9.980053956 13.634142346

H 13.791907437 13.035665971 15.111987707

H 13.993692928 14.766467316 15.353664274

H 17.418743656 12.322404038 16.318817595

H 15.998020870 11.477588874 15.666987096

H 16.243702068 14.482567333 16.270786918

H 15.195484290 13.327384895 17.111529555

H 18.940608697 11.919181982 14.188591652

H 17.650592800 10.908489188 13.501415489

H 18.389037105 11.973925419 11.452798576

H 18.928488858 13.421983726 12.321175849

H 16.089121162 12.756093156 11.426054971

H 16.836007906 14.341553352 11.595509774

H 14.230030664 11.613753799 12.558240418

H 10.301371050 9.802024584 12.401286223

H 11.778370029 8.859854015 11.818340718

H 12.075063112 12.905151570 13.385685939

H 10.540150757 12.398041280 12.555199679

H 16.054113063 10.238617224 12.067588499

H 17.241349106 8.615738607 10.617817127

H 16.054416441 7.556855565 8.693535192

H 13.675219516 8.158845874 8.245110697

H 12.517189341 9.820981228 9.663705046

Int (6+7) -780.8843493 a.u.

C 9.654999427 16.535345994 11.610615617

C 10.780163981 16.425949092 9.294282710

C 9.998152741 17.346655067 10.282322842

C 10.991014815 15.835532515 11.906734392

C 8.699964559 15.404758191 11.226212435

C 9.310404957 14.442009228 10.479651951

C 8.675740385 13.337180046 9.737798037

C 7.257797210 15.452860207 11.631107622

C 7.450414294 13.530914389 9.063007427

C 6.845409402 12.492400171 8.351260098

C 7.449155848 11.231761629 8.293715564

C 8.674325033 11.029456952 8.936621535

C 9.283137274 12.069357887 9.638089590

C 9.187437397 17.449225131 12.743057193

C 8.793418461 18.050395709 9.690535647

C 7.905406871 17.411058633 8.810413699

C 6.767447994 18.065297214 8.329981176

C 6.496448830 19.382576957 8.713042745

C 7.377292698 20.039022639 9.577932187

C 8.511228229 19.377037507 10.058225725

C 11.996103464 17.153361942 8.607008626

C 12.337082923 16.329298795 6.145327064

C 13.579396325 16.281083185 5.251908942

C 14.520138453 15.228734373 5.281895316

C 15.610958832 15.139019661 4.404328254

C 15.809461527 16.201444387 3.510459813

C 14.924821868 17.279800102 3.474206208

C 13.813655657 17.301590860 4.322008729

C 15.399345751 13.454434714 6.629043980

C 16.488163292 13.251078596 5.777807312

C 17.525837066 12.432826247 6.255201219

C 17.455471528 11.826171969 7.512430274

C 16.353068234 12.046765584 8.345550835

C 15.319888883 12.885317785 7.920733126

C 16.421049173 13.837014619 4.366111619

C 17.822046830 14.046313334 3.766812531

C 15.631263017 12.828879761 3.479341954

C 12.600054904 12.908448057 7.688349997

C 14.612433587 15.092386749 9.376071817

C 14.472539747 15.441238087 10.735105227

C 15.090054217 16.576583418 11.267628780

C 15.860120521 17.403518998 10.448655680

C 16.038614719 17.056694708 9.105282402

C 15.439557577 15.910466243 8.579176320

C 12.836624239 11.636416186 7.124858650

C 11.885255824 11.023520611 6.304818948

C 10.676756683 11.662267050 6.016179542

C 10.428040165 12.916528388 6.576278536

C 11.373558803 13.521557608 7.406996124

C 11.626937719 18.562739640 8.092999699

C 12.855120258 19.352615691 7.622135657

P 11.104311041 14.836275993 10.383083254

P 13.899662311 13.405646655 8.949088935

N 12.681152583 16.415144147 7.554219584

O 14.323373739 14.241416061 6.242282587

Pd 12.535153796 12.970651201 10.733584666

H 10.108894884 16.075655414 8.500479001

H 10.699214601 18.122196259 10.628184093

H 10.949385531 15.183878301 12.786769431

H 11.834153792 16.531903155 12.005679174

H 6.742618954 14.511413961 11.409168279

H 6.726375402 16.270067388 11.117900260

H 7.170346599 15.651604948 12.709356972

H 6.977887988 14.510030568 9.085286952

H 5.902190416 12.670201799 7.838727085

H 6.975330229 10.421435646 7.743895301

H 9.169477455 10.061940672 8.881913495

H 10.255614409 11.911347239 10.102888977

H 9.028103387 16.876296778 13.666316917

H 8.251606306 17.967219672 12.495779216

H 9.947750934 18.213911211 12.953344458

H 8.100247135 16.386474980 8.500991552

H 6.094229770 17.545532770 7.651366601

H 5.613750917 19.894394829 8.335914168

H 7.183833124 21.066805680 9.877969208

H 9.193832037 19.896724502 10.729501291

H 12.756329806 17.297348252 9.393346354

H 13.185920435 15.608816319 7.888948323

H 11.761544342 17.218186573 5.860235006

H 11.704221180 15.456661644 5.900024874

H 16.650792858 16.178948663 2.823489841

H 15.090565312 18.094378116 2.773182433

H 13.110631616 18.131270953 4.271595831

H 18.394529957 12.249895295 5.628019842

H 18.260638358 11.174672202 7.844007605

H 16.286710597 11.563384619 9.319010610

H 18.369653911 13.097143717 3.718269539

H 18.412747742 14.759251273 4.356747544

H 17.754323313 14.416842060 2.736655694

H 15.518431032 13.224948058 2.461303328

H 14.629583685 12.646362599 3.890775037

H 16.162155761 11.868663318 3.429731457

H 13.934402096 14.781503309 11.420089494

H 14.982035928 16.772927053 12.332581147

H 16.338041111 18.293706002 10.853549081

H 16.651798252 17.676785717 8.454900949

H 15.626004639 15.680028217 7.538012965

H 13.764357796 11.109720892 7.331127111

H 12.099807221 10.039699599 5.891810930

H 9.936012282 11.185806927 5.378383888

H 9.482419479 13.422526323 6.392455692

H 11.154659224 14.494747037 7.825211987

H 11.126241501 19.123617140 8.890198697

H 10.876570275 18.484268061 7.292708953

H 13.588421255 19.447557152 8.435261021

H 12.575232144 20.364346396 7.299951653

H 13.370967746 18.856184597 6.791391921

C 14.399982288 13.797973043 15.074677868

C 16.012947736 13.300714951 13.453665816

C 16.281674003 12.238463096 15.670073432

C 15.462027051 13.450518950 16.131175674

C 17.783674578 11.725433330 13.653189813

C 17.991218258 12.481138656 12.321084872

C 16.660675676 13.225471335 12.086030595

C 11.049118691 9.868234712 11.960704933

C 11.888354854 11.039624159 12.247437927

C 13.279665315 11.249372585 11.746124556

C 14.011885740 10.215587253 10.987573147

C 11.351527797 12.413525408 12.414213184

C 15.273517215 9.780837108 11.445235681

C 16.009024505 8.815767493 10.751705092

C 15.497470201 8.250455185 9.578242648

C 14.241309964 8.663284646 9.114211144

C 13.511555374 9.630040887 9.806880238

C 10.619091236 10.339162590 17.664056928

C 12.554650189 9.656777278 16.250200305

C 11.718819012 8.590720560 15.650185427

C 10.291580865 8.538792534 15.967800377

C 8.392673070 9.391170560 17.252293292

C 12.791613628 11.419784857 17.957562288

C 12.358671756 7.912527100 14.637253518

C 12.034395297 6.886094611 13.666585022

C 10.935118375 5.998476952 13.703216772

C 10.754087079 5.063918103 12.684002157

C 11.641157936 5.002861368 11.601932969

C 12.735841698 5.873934189 11.548431140

C 12.938888985 6.788563765 12.577301585

N 14.936998484 13.970591181 13.717259691

N 16.745348270 12.504942765 14.314979572

N 9.826498792 9.450484412 16.938147073

N 11.967188599 10.412920317 17.279902967

O 11.626550618 9.984050521 13.283266437

O 10.161421007 11.028850780 18.575257847

O 9.457019189 7.794552836 15.445648771

O 13.715574947 9.898195541 15.896479436

H 13.633646262 13.003665962 15.048956494

H 13.878421727 14.724397329 15.354260506

H 17.157413518 12.083714887 16.317182082

H 15.667056680 11.324105447 15.703334537

H 16.131406603 14.310563544 16.271499987

H 14.994068366 13.235802118 17.101526045

H 18.692886412 11.692302106 14.270863140

H 17.459039706 10.683145608 13.491324411

H 18.247398729 11.801750637 11.499342092

H 18.813415049 13.199067452 12.429560151

H 15.983881154 12.666354997 11.423162148

H 16.783178368 14.221432186 11.645863813

H 13.894275407 11.695868032 12.538814371

H 9.962675945 9.988981967 11.913580051

H 11.446606182 9.039510815 11.367436174

H 11.789337915 12.945596259 13.267222025

H 10.268092744 12.540755358 12.354778157

H 15.662996816 10.203406216 12.370058932

H 16.979612296 8.501625039 11.131738367

H 16.069075185 7.504366921 9.031095667

H 13.833453275 8.237369585 8.199108235

H 12.554906707 9.972763887 9.416404320

H 8.183725851 10.160317585 17.998325087

H 8.136241533 8.398855336 17.640580060

H 7.814921797 9.562556864 16.337727086

H 12.418771526 11.544226553 18.977159558

H 12.732264332 12.381569709 17.432081401

H 13.828023599 11.072815648 17.948033814

H 10.228513775 6.073113849 14.522595834

H 9.906991159 4.382621015 12.729431321

H 11.480335860 4.278689806 10.805428747

H 13.429784547 5.846552988 10.711015892

H 13.785899174 7.472817866 12.529594332

H 13.379154224 8.284459982 14.518703236

Int 7 -148.8607534 a.u.

N 12.757738374 10.783634213 11.101814090

N 14.154100523 10.967614679 13.017870044

C 13.585402637 10.148778250 12.032911037

C 13.999154379 12.364562672 13.103381110

C 13.097262182 12.994198099 12.093829738

C 12.530648472 12.166272573 11.021622765

C 12.159208211 9.960465164 10.040314673

C 15.031302961 10.290996524 13.985375225

C 12.904405394 14.342768066 12.293300783

C 12.147747578 15.429264398 11.703265205

C 11.276869580 15.363287429 10.588359326

C 10.600386573 16.500966694 10.153968013

C 10.774929439 17.730213328 10.801472054

C 11.635155307 17.820311574 11.902490354

C 12.307858451 16.686552175 12.346823115

O 13.806120021 8.941565394 11.986607517

O 11.882990045 12.589236868 10.059996848

O 14.584055525 13.011420261 13.976169737

H 12.353839363 8.913671729 10.281592394

H 12.601935825 10.218890865 9.071396552

H 11.083862696 10.161255533 9.994026545

H 15.929753990 9.915429969 13.481702853

H 14.498742187 9.444583936 14.430214623

H 15.304318110 11.027637973 14.744643562

H 11.159922907 14.413165831 10.078245550

H 9.935757371 16.430394938 9.295564734

H 10.246787067 18.614244115 10.448548772

H 11.778628772 18.771995279 12.409867102

H 12.973630471 16.755744983 13.206129674

H 13.481538975 14.672694885 13.163087091

Int 8 -780.8695535 a.u.

C 9.891321172 16.206854425 11.970625411

C 10.909201641 16.151146008 9.596376743

C 10.152064326 17.034742779 10.636351324

C 11.259312927 15.541329382 12.196628063

C 8.946832174 15.053041821 11.630484514

C 9.540909600 14.110087813 10.845076907

C 8.907197598 12.990322553 10.126799286

C 7.529208908 15.056289810 12.113197520

C 7.633015598 13.132124958 9.535649666

C 7.035320306 12.072357160 8.849621782

C 7.694142749 10.843908976 8.734578347

C 8.966638599 10.693207247 9.293863911

C 9.565263174 11.756045479 9.968271812

C 9.456808542 17.104591286 13.127918398

C 8.906663888 17.715757368 10.104551328

C 7.985325471 17.059833096 9.272767971

C 6.817961548 17.699308072 8.844817279

C 6.550261627 19.015101176 9.234907987

C 7.462165427 19.685843355 10.055563494

C 8.625984373 19.039776122 10.482439523

C 12.065393703 16.926934011 8.854942128

C 12.292614994 16.245570945 6.328208747

C 13.506710557 16.289948031 5.395799334

C 14.477567655 15.268173430 5.350187709

C 15.551810755 15.253067972 4.450339570

C 15.700322761 16.364371031 3.607786375

C 14.781063263 17.414426180 3.643060401

C 13.688297614 17.362174795 4.513479103

C 15.437616280 13.472490066 6.614822059

C 16.522499916 13.354750569 5.739432018

C 17.618003235 12.588945174 6.170164305

C 17.616642591 11.946178865 7.410717461

C 16.522972777 12.078393565 8.272079462

C 15.427318271 12.860014307 7.893317846

C 16.399935445 13.979148360 4.347543714

C 17.776397304 14.249511871 3.717651796

C 15.619025043 12.969542594 3.454402837

C 12.693471191 12.748120300 7.836717321

C 14.696240210 14.951640121 9.501502673

C 14.641654575 15.234442669 10.881422627

C 15.221824794 16.387428093 11.417141501

C 15.873023534 17.295985439 10.581201822

C 15.975210741 17.012433857 9.215136653

C 15.412083173 15.851183753 8.683175364

C 12.936835369 11.466242012 7.297593850

C 11.973327298 10.824749733 6.515317362

C 10.747752786 11.441574758 6.250412618

C 10.492447923 12.700650025 6.794544664

C 11.451111449 13.339409227 7.584865497

C 11.639220757 18.346724698 8.421273202

C 12.820473840 19.180202575 7.906135664

P 11.307636877 14.557990756 10.658963890

P 14.043192572 13.265075817 9.021480675

N 12.681168126 16.238634934 7.729576223

O 14.323064785 14.224765720 6.258660087

Pd 12.800234817 12.719931504 10.908036925

H 10.206366508 15.786621934 8.837593780

H 10.849924626 17.822593923 10.958937113

H 11.272711989 14.892877659 13.079008338

H 12.087867690 16.259274034 12.254305533

H 7.033772518 14.097635260 11.922502851

H 6.946148506 15.855267313 11.628066761

H 7.495145772 15.255391983 13.194291902

H 7.115677607 14.086193206 9.601628924

H 6.053613767 12.208766263 8.400666450

H 7.224810666 10.016897722 8.206095392

H 9.500279456 9.749768948 9.197941981

H 10.572645405 11.643853996 10.368853852

H 9.355636919 16.526180697 14.055893623

H 8.496789616 17.597136201 12.925622594

H 10.204377315 17.890004610 13.304156514

H 8.176001929 16.035661091 8.958633417

H 6.119190647 17.168717617 8.201327314

H 5.644829750 19.514677142 8.897189659

H 7.270744338 20.712570219 10.360492226

H 9.333363261 19.571395136 11.117824180

H 12.872970618 17.058220464 9.594704516

H 13.194147257 15.410780005 7.986930109

H 11.682362134 17.132834645 6.123468155

H 11.675326438 15.371993911 6.048898332

H 16.527589222 16.403170435 2.904581566

H 14.906704564 18.266655908 2.979424733

H 12.960041472 18.171388619 4.517254235

H 18.481578599 12.478826384 5.519508732

H 18.469683792 11.340555954 7.707508581

H 16.514054884 11.578908298 9.239980492

H 18.347781476 13.318866906 3.615373146

H 18.365172462 14.957376020 4.315577199

H 17.665984957 14.655708624 2.704733772

H 15.470074403 13.386634581 2.449493644

H 14.633252714 12.745571464 3.882788631

H 16.177192442 12.027660663 3.367492665

H 14.203869254 14.518517261 11.580484903

H 15.178299092 16.538932001 12.494030402

H 16.318217775 18.201970352 10.988439509

H 16.499105373 17.695702579 8.550084168

H 15.537474157 15.672418779 7.623474795

H 13.879532062 10.959035646 7.489358861

H 12.189996610 9.837106525 6.112961445

H 9.998438388 10.942203000 5.640473951

H 9.533878172 13.187063200 6.626176860

H 11.231176037 14.319381301 7.985735046

H 11.184684968 18.868439993 9.270337886

H 10.838782426 18.284775105 7.669888382

H 13.594638271 19.271763852 8.680649259

H 12.495985138 20.193054609 7.632994371

H 13.301132915 18.725663133 7.032002235

C 14.573918928 13.647900536 15.161867204

C 16.418417742 13.419345306 13.720029514

C 16.772348406 12.863920691 16.092491558

C 15.581452238 13.803145627 16.310973520

C 18.536176537 12.521681812 14.265413440

C 18.649862120 13.111014365 12.845107937

C 17.182855887 13.317222185 12.413157038

C 11.617172547 9.822731064 12.376407774

C 12.526698199 11.039796266 12.428414004

C 13.895151353 11.079087998 12.020531342

C 14.585215984 10.012099541 11.281947639

C 11.983939971 12.257266485 12.901922133

C 15.895526179 9.650963155 11.654827812

C 16.585399238 8.649201919 10.968824276

C 15.981944388 7.991748204 9.891278506

C 14.684564825 8.347694863 9.502640015

C 13.995735936 9.348714477 10.186494461

C 10.789488073 7.874738747 18.558753659

C 10.163453821 8.511748900 16.205987901

C 11.545794025 8.461008869 15.856579423

C 12.562694576 8.156685966 16.812289240

C 13.153156082 7.531553844 19.121746009

C 8.467646431 8.254588357 18.017313579

C 12.019712582 8.585838721 14.431115826

C 12.000187646 7.259544771 13.685856513

C 10.778283222 6.646508078 13.359157949

C 10.766813997 5.451511086 12.637951459

C 11.970645167 4.853605914 12.235809538

C 13.187443758 5.457187879 12.564406589

C 13.200143077 6.657171037 13.285781924

N 15.161887802 13.717129969 13.811889268

N 17.282165308 13.097981788 14.747832423

N 12.125154400 7.897135429 18.148854235

N 9.868530614 8.247953280 17.594378885

O 11.196607162 9.557420475 13.713030343

O 10.426704125 7.555636683 19.703398367

O 13.794981786 8.077268972 16.579366649

O 9.201460386 8.759272756 15.447998828

H 14.044334520 12.686058603 15.263704369

H 13.803377672 14.429657161 15.233213249

H 17.576641966 13.064040710 16.814469833

H 16.467549493 11.811266278 16.216821673

H 15.945326292 14.839141217 16.347422416

H 15.101722399 13.585469864 17.275254977

H 19.369808418 12.809056373 14.920812713

H 18.484977754 11.419431964 14.250105028

H 19.210863372 12.457692194 12.166999402

H 19.168949860 14.076458599 12.888665348

H 16.798166664 12.453027365 11.850360146

H 17.022600527 14.200849558 11.784554721

H 14.534084017 11.778886961 12.572772389

H 10.703902051 10.038738376 11.804112230

H 12.116043420 8.958051588 11.918368345

H 12.627389457 12.977967000 13.410309268

H 10.920385481 12.296193569 13.136721252

H 16.359027024 10.146399980 12.507074979

H 17.591841152 8.376476508 11.279793927

H 16.519363732 7.212874857 9.354759890

H 14.212374962 7.848452706 8.659216051

H 13.005620420 9.652945163 9.851062730

H 12.654750462 7.372189704 20.081588207

H 13.897242735 8.332919732 19.196428087

H 13.672312400 6.619543914 18.802627093

H 8.337627753 8.921539020 18.876842648

H 8.149874714 7.248042751 18.316192042

H 7.884282284 8.598831142 17.158719358

H 9.855597036 7.131379387 13.678418983

H 9.818007309 4.982590195 12.385330698

H 11.956164627 3.924335387 11.670201070

H 14.125596026 5.001907202 12.254322731

H 14.142887063 7.135613419 13.547411869

H 13.065479041 8.934638094 14.471403802

TS1 -780.8787958 a.u.

C 10.697999954 17.881999969 11.458999634

C 11.342000008 17.003999710 9.116000175

C 10.883999825 18.260999680 9.920000076

C 11.953000069 17.034000397 11.715999603

C 9.534000397 16.898000717 11.559000015

C 9.838000298 15.694000244 10.998999596

C 8.930999756 14.583999634 10.668000221

C 8.227000237 17.305000305 12.168999672

C 7.629000187 14.836000443 10.178999901

C 6.777999878 13.791000366 9.814999580

C 7.210000038 12.463999748 9.918000221

C 8.500000000 12.194999695 10.385999680

C 9.347000122 13.239999771 10.756999969

C 10.597999573 19.121000290 12.347000122

C 9.694000244 18.995000839 9.340999603

C 8.581000328 18.319000244 8.814000130

C 7.465000153 19.018999100 8.347999573

C 7.441999912 20.416999817 8.388999939

C 8.548000336 21.106000900 8.895000458

C 9.659000397 20.399000168 9.362999916

C 12.529999733 17.302999496 8.119999886

C 12.118000031 15.994999886 5.923999786

C 13.045999527 15.621000290 4.771999836

C 13.859000206 14.468999863 4.797999859

C 14.663000107 14.076000214 3.716000080

C 14.704999924 14.920999527 2.596999884

C 13.935999870 16.083999634 2.546000004

C 13.102000237 16.413000107 3.618999958

C 14.861000061 12.826999664 6.219999790

C 15.692000389 12.361000061 5.193999767

C 16.725999832 11.475999832 5.539999962

C 16.861999512 11.003999710 6.844999790

C 15.996000290 11.461000443 7.840000153

C 15.029000282 12.428999901 7.563000202

C 15.331000328 12.699999809 3.750999928

C 16.547000885 12.618000031 2.812000036

C 14.272000313 11.644000053 3.312999964

C 12.406999588 12.762999535 8.069999695

C 15.003999710 14.854000092 8.855999947

C 15.177000046 15.482999802 10.111000061

C 15.928000450 16.653999329 10.248000145

C 16.548000336 17.228000641 9.135000229

C 16.434000015 16.593999863 7.895999908

C 15.685000420 15.420000076 7.761000156

C 12.340999603 11.387000084 7.760000229

C 11.182000160 10.817999840 7.228000164

C 10.050999641 11.604000092 6.989999771

C 10.092000008 12.961000443 7.317999840

C 11.248999596 13.527000427 7.859000206

C 12.338000298 18.645000458 7.372000217

C 13.546999931 19.017999649 6.505000114

P 11.612000465 15.692999840 10.515000343

P 14.032999992 13.227000237 8.906999588

N 12.848999977 16.257999420 7.156000137

O 13.815999985 13.711000443 5.961999893

Pd 12.746000290 13.791999817 10.718999863

H 10.508999825 16.610000610 8.520999908

H 11.729999542 18.962999344 9.939000130

H 12.013999939 16.636999130 12.736000061

H 12.890000343 17.558000565 11.480999947

H 7.561999798 16.443000793 12.305999756

H 7.705999851 18.045999527 11.541999817

H 8.387000084 17.780000687 13.147999763

H 7.293000221 15.864000320 10.064999580

H 5.782000065 14.013999939 9.439000130

H 6.551000118 11.649000168 9.623000145

H 8.859000206 11.168999672 10.447999954

H 10.366000175 13.022999763 11.088000298

H 10.545000076 18.839000702 13.406999588

H 9.713000298 19.726999283 12.112000465

H 11.484000206 19.757999420 12.215999603

H 8.583000183 17.232000351 8.774000168

H 6.614999771 18.471000671 7.947999954

H 6.574999809 20.962999344 8.024000168

H 8.548000336 22.194000244 8.923999786

H 10.519000053 20.943000793 9.753000259

H 13.440999985 17.409000397 8.732000351

H 13.234000206 15.421999931 7.577000141

H 11.574000359 16.899999619 5.629000187

H 11.361000061 15.197999954 6.027999878

H 15.329999924 14.657999992 1.748000026

H 13.975000381 16.722999573 1.667999983

H 12.482999802 17.308000565 3.569999933

H 17.409000397 11.123999596 4.771999836

H 17.632999420 10.276000023 7.087999821

H 16.069999695 11.072999954 8.850000381

H 16.986999512 11.614000320 2.832999945

H 17.322999954 13.343999863 3.089999914

H 16.252000809 12.803000450 1.773000002

H 13.935999870 11.833999634 2.285000086

H 13.397999763 11.675000191 3.976999998

H 14.704999924 10.635999680 3.368000031

H 14.713000298 15.048999786 11.003000259

H 16.027999878 17.107000351 11.232999802

H 17.125000000 18.145999908 9.232000351

H 16.920999527 17.013999939 7.018000126

H 15.635999680 14.968000412 6.777999878

H 13.208000183 10.753000259 7.928999901

H 11.170999527 9.755000114 6.997000217

H 9.151000023 11.163999557 6.566999912

H 9.213999748 13.588000298 7.171000004

H 11.253000259 14.579999924 8.109999657

H 12.161999702 19.447000504 8.097999573

H 11.418999672 18.608999252 6.769000053

H 14.458999634 19.072000504 7.114999771

H 13.399999619 19.996000290 6.026999950

H 13.737000465 18.275999069 5.721000195

C 15.612999916 15.182000160 14.210000038

C 13.494000435 14.194999695 14.449999809

C 14.753999710 14.640000343 16.520999908

C 15.968999863 14.595000267 15.586999893

C 12.328000069 13.876999855 16.489000320

C 11.413999557 13.319000244 15.371999741

C 12.116000175 13.710000038 14.048999786

C 9.875000000 8.899000168 17.254999161

C 10.897000313 9.163000107 14.996000290

C 12.262000084 8.885000229 15.571999550

C 12.350999832 8.805999756 17.082000732

C 11.270999908 8.791999817 19.280000687

C 8.446000099 9.149000168 15.291000366

C 13.262000084 9.968000412 15.100000381

C 12.772999763 7.461999893 15.034999847

C 13.883999825 8.354000092 13.118000031

C 11.855999947 6.344999790 15.475000381

C 12.081000328 5.705999851 16.702999115

C 11.178000450 4.750999928 17.179000854

C 10.046999931 4.421999931 16.426000595

C 9.831999779 5.039000034 15.189000130

C 10.732000351 5.997000217 14.713000298

C 14.050000191 9.678999901 13.843000412

C 14.925999641 10.628999710 13.456999779

C 16.040000916 10.498000145 12.505000114

C 16.954000473 9.435000420 12.645999908

C 18.069999695 9.331000328 11.817000389

C 18.309999466 10.307000160 10.845000267

C 17.437000275 11.392999649 10.729000092

C 16.305000305 11.487000465 11.543000221

N 14.369999886 14.659999847 13.623000145

N 13.614999771 14.062000275 15.824000359

N 11.170999527 8.850000381 17.812000275

N 9.798000336 9.038000107 15.862000465

O 8.869000435 8.854999542 17.958999634

O 13.446999550 8.684000015 17.631999969

O 10.758000374 9.463000298 13.812000275

O 12.852999687 7.477000237 13.628999710

H 15.522000313 16.277999878 14.281000137

H 16.430000305 14.989000320 13.498999596

H 14.937999725 14.062000275 17.438999176

H 14.532999992 15.677000046 16.827999115

H 16.291000366 13.550999641 15.468000412

H 16.806999207 15.144000053 16.038999557

H 12.430999756 13.189000130 17.340000153

H 11.949999809 14.836000443 16.881999969

H 10.399999619 13.727000237 15.439000130

H 11.324999809 12.229999542 15.460000038

H 11.590999603 14.517000198 13.520000458

H 12.197999954 12.871000290 13.340999603

H 10.326999664 8.409999847 19.673999786

H 11.454999924 9.796999931 19.680999756

H 12.111000061 8.145000458 19.545000076

H 8.541000366 9.633000374 14.314999580

H 7.815999985 9.736000061 15.965000153

H 8.010999680 8.149999619 15.170999527

H 12.737999916 10.925999641 14.996000290

H 13.987999916 10.083000183 15.913000107

H 13.767000198 7.341000080 15.498999596

H 13.588999748 8.555000305 12.078000069

H 14.829999924 7.791999817 13.095000267

H 12.960000038 5.967999935 17.288999557

H 11.359000206 4.265999794 18.135000229

H 9.338999748 3.684000015 16.798999786

H 8.958999634 4.776000023 14.593999863

H 10.572999954 6.484000206 13.753000259

H 16.799999237 8.713000298 13.444999695

H 18.763000488 8.501999855 11.946000099

H 19.176000595 10.227999687 10.189999580

H 17.624000549 12.163999557 9.984999657

H 15.607000351 12.314000130 11.418000221

H 14.868000031 11.595000267 13.967000008

TS2 -714.3847757 a.u.

C 9.326999664 16.017999649 11.935999870

C 10.381999969 15.960000038 9.588999748

C 9.748999596 16.886999130 10.673000336

C 10.550999641 15.104000092 12.130999565

C 8.211000443 15.081000328 11.487000465

C 8.659000397 14.109000206 10.642000198

C 7.839000225 13.185999870 9.836000443

C 6.800000191 15.312000275 11.939999580

C 6.623000145 13.612000465 9.255999565

C 5.828999996 12.732000351 8.517000198

C 6.230000019 11.404000282 8.335000038

C 7.447999954 10.977000237 8.871999741

C 8.244999886 11.859000206 9.598999977

C 9.019000053 16.899000168 13.147999763

C 8.649999619 17.818000793 10.182000160

C 7.659999847 17.410999298 9.272000313

C 6.636000156 18.277999878 8.876999855

C 6.580999851 19.583000183 9.376999855

C 7.564000130 20.010999680 10.274000168

C 8.583000183 19.136999130 10.668000221

C 11.668999672 16.604999542 8.954000473

C 11.753000259 15.967000008 6.445000172

C 12.892999649 15.946000099 5.433000088

C 13.739999771 14.833000183 5.244999886

C 14.737999916 14.800999641 4.260000229

C 14.939999580 15.956000328 3.487999916

C 14.142999649 17.084999084 3.668999910

C 13.119999886 17.065000534 4.619999886

C 14.598999977 12.852999687 6.283999920

C 15.600000381 12.720000267 5.308000088

C 16.662000656 11.843000412 5.576000214

C 16.715999603 11.114999771 6.764999866

C 15.711000443 11.265999794 7.724999905

C 14.638999939 12.142000198 7.513999939

C 15.440999985 13.470999718 3.983000040

C 16.787000656 13.673000336 3.262000084

C 14.501999855 12.633999825 3.065000057

C 11.918000221 12.185000420 7.760000229

C 14.067999840 14.062000275 9.428000450

C 13.904999733 14.361000061 10.795999527

C 14.532999992 15.470999718 11.371999741

C 15.350000381 16.304000854 10.602000237

C 15.559000015 15.996000290 9.255000114

C 14.937999725 14.885999680 8.678999901

C 11.951000214 10.894000053 7.190999985

C 10.925000191 10.449999809 6.356999874

C 9.826000214 11.274000168 6.091000080

C 9.770000458 12.541000366 6.673999786

C 10.805999756 12.991999626 7.498000145

C 11.468000412 18.100999832 8.593999863

C 12.769000053 18.784999847 8.152000427

P 10.501999855 14.204999924 10.531000137

P 13.309092241 12.328926331 9.111963704

N 12.246000290 15.909000397 7.810999870

O 13.538999557 13.725999832 6.077000141

Pd 11.446999550 11.826000214 10.838000298

H 9.664999962 15.767999649 8.781000137

H 10.559000015 17.524000168 11.062000275

H 10.430999756 14.404999733 12.967000008

H 11.489000320 15.661999702 12.253999710

H 6.145999908 14.472999573 11.675000191

H 6.381999969 16.231000900 11.498999596

H 6.768000126 15.446999550 13.031000137

H 6.307000160 14.645999908 9.373999596

H 4.896999836 13.085000038 8.081000328

H 5.605999947 10.713999748 7.769999981

H 7.790999889 9.956000328 8.718999863

H 9.210000038 11.522999763 9.986000061

H 8.795000076 16.288999557 14.032999992

H 8.163999557 17.562999725 12.965999603

H 9.883999825 17.531999588 13.390000343

H 7.686999798 16.400999069 8.869000435

H 5.881999969 17.933000565 8.171999931

H 5.787000179 20.257999420 9.065999985

H 7.539000034 21.025999069 10.666000366

H 9.345999718 19.486000061 11.362999916

H 12.451999664 16.577999115 9.727999687

H 12.677000046 15.027000427 8.048999786

H 11.204999924 16.906000137 6.302999973

H 11.048000336 15.154999733 6.192999840

H 15.711999893 15.967000008 2.723999977

H 14.305000305 17.971000671 3.059000015

H 12.480999947 17.937000275 4.748000145

H 17.452999115 11.720999718 4.840000153

H 17.542999268 10.430999756 6.942999840

H 15.755000114 10.708000183 8.657999992

H 17.259000778 12.711000443 3.030999899

H 17.486000061 14.270999908 3.861999989

H 16.635999680 14.178999901 2.299999952

H 14.326999664 13.166000366 2.119999886

H 13.531999588 12.463000298 3.549000025

H 14.954999924 11.659000397 2.844000101

H 13.331999779 13.694999695 11.442999840

H 14.397000313 15.657999992 12.435000420

H 15.838999748 17.167999268 11.048000336

H 16.208999634 16.618000031 8.642000198

H 15.147000313 14.678999901 7.637000084

H 12.789999962 10.230999947 7.396999836

H 10.987999916 9.454000473 5.923999786

H 9.019000053 10.925999641 5.452000141

H 8.907999992 13.182999611 6.505000114

H 10.751000404 13.984000206 7.927999973

H 11.065999985 18.638999939 9.460000038

H 10.692000389 18.202999115 7.822000027

H 13.527999878 18.725999832 8.944999695

H 12.598999977 19.847999573 7.929999828

H 13.203000069 18.312000275 7.263999939

C 13.451000214 13.095999718 15.031999588

C 14.704999924 11.703000069 13.616999626

C 14.769000053 11.192000389 16.018999100

C 14.404000282 12.673999786 16.163000107

C 15.838000298 9.718999863 14.223999977

C 16.212999344 10.015999794 12.755999565

C 15.196999550 11.095000267 12.319000244

C 8.991000175 9.112999916 12.182999611

C 10.151000023 10.012999535 12.322999954

C 11.531999588 9.807000160 11.776000023

C 11.911999702 8.590000153 11.020999908

C 10.074999809 11.498999596 12.397999763

C 13.119999886 7.926000118 11.338000298

C 13.517999649 6.758999825 10.682000160

C 12.706000328 6.197000027 9.692000389

C 11.494000435 6.822000027 9.373999596

C 11.109000206 8.001999855 10.017000198

C 5.326000214 3.368999958 10.869000435

C 5.109000206 4.339000225 12.038000107

C 3.664999962 4.250999928 12.541999817

C 6.092000008 4.059000015 13.184000015

C 4.908999920 7.835000038 13.102000237

N 13.887000084 12.697999954 13.689999580

N 15.265000343 10.987000465 14.666999817

O 9.642000198 9.204000473 13.468999863

O 5.282999992 5.709000111 11.552000046

O 5.471000195 8.512000084 12.324000359

O 4.335000038 7.208000183 13.913999557

H 12.444999695 12.678999901 15.206000328

H 13.329000473 14.189999580 15.036000252

H 15.555999756 10.904999733 16.732999802

H 13.892000198 10.553000450 16.218999863

H 15.324999809 13.270999908 16.104999542

H 13.951999664 12.859999657 17.146999359

H 16.704000473 9.449000359 14.845000267

H 15.100999832 8.899999619 14.300000191

H 16.173999786 9.114999771 12.131999969

H 17.233999252 10.411999702 12.713000298

H 14.335000038 10.670000076 11.782999992

H 15.621999741 11.864000320 11.661999702

H 12.258999825 10.024999619 12.569999695

H 7.981999874 9.531999588 12.126999855

H 9.107999802 8.140000343 11.696999550

H 10.593999863 11.888999939 13.282999992

H 9.092000008 11.956999779 12.258000374

H 13.743000031 8.326999664 12.137000084

H 14.456999779 6.280000210 10.953000069

H 13.010000229 5.288000107 9.177000046

H 10.845999718 6.386000156 8.616000175

H 10.173000336 8.480999947 9.737999916

H 5.157999992 2.328999996 11.182000160

H 6.352000237 3.440999985 10.484000206

H 4.631000042 3.604000092 10.052000046

H 2.957000017 4.506999969 11.744000435

H 3.500999928 4.934000015 13.383999825

H 3.448999882 3.229000092 12.880999565

H 5.965000153 3.035000086 13.564000130

H 5.922999859 4.756999969 14.013999939

H 7.132999897 4.163000107 12.848999977

H 6.181000233 5.791999817 11.189000130

TS3 -780.8600309 a.u.

C 9.713000298 16.548000336 11.564000130

C 10.812000275 16.422000885 9.232000351

C 10.041000366 17.347999573 10.225999832

C 11.053000450 15.852000237 11.850999832

C 8.751999855 15.413999557 11.204000473

C 9.350999832 14.442999840 10.458000183

C 8.708000183 13.333000183 9.732000351

C 7.315999985 15.463999748 11.628000259

C 7.469999790 13.515999794 9.078000069

C 6.861000061 12.468999863 8.381999969

C 7.473000050 11.211999893 8.319000244

C 8.710000038 11.020999908 8.942000389

C 9.321000099 12.069000244 9.628999710

C 9.258999825 17.472999573 12.692000389

C 8.829000473 18.048000336 9.640000343

C 7.927000046 17.402000427 8.781000137

C 6.784999847 18.055999756 8.307000160

C 6.526000023 19.378999710 8.675999641

C 7.421000004 20.041999817 9.522000313

C 8.557999611 19.379999161 9.996000290

C 12.015999794 17.149999619 8.524000168

C 12.321999550 16.343000412 6.048999786

C 13.557000160 16.304000854 5.143000126

C 14.501000404 15.253999710 5.151999950

C 15.588999748 15.177000046 4.274000168

C 15.781000137 16.246999741 3.388999939

C 14.890000343 17.322000504 3.365999937

C 13.784000397 17.334999084 4.221000195

C 15.390000343 13.477000237 6.495999813

C 16.482000351 13.288999557 5.644999981

C 17.531999588 12.487000465 6.120999813

C 17.472000122 11.881999969 7.379000187

C 16.371000290 12.090999603 8.217000008

C 15.322999954 12.913000107 7.793000221

C 16.399999619 13.875000000 4.232999802

C 17.795000076 14.086000443 3.622999907

C 15.602999687 12.864000320 3.355999947

C 12.595000267 12.913000107 7.618000031

C 14.612999916 15.102000237 9.281999588

C 14.498999596 15.442999840 10.645000458

C 15.111000061 16.586999893 11.166999817

C 15.850999832 17.427000046 10.333999634

C 16.004999161 17.086999893 8.986000061

C 15.409999847 15.934000015 8.468999863

C 12.824999809 11.628000259 7.080999851

C 11.868000031 11.005999565 6.274000168

C 10.661999702 11.645999908 5.980000019

C 10.418000221 12.909999847 6.519999981

C 11.369000435 13.526000023 7.335999966

C 11.637999535 18.559999466 8.020000458

C 12.859000206 19.350000381 7.528999805

P 11.137999535 14.840999603 10.333999634

P 13.914999962 13.418999672 8.845000267

N 12.680000305 16.412000656 7.456999779

O 14.309000015 14.239999771 6.085000038

Pd 12.578000069 12.967000008 10.663000107

H 10.128999710 16.062999725 8.453000069

H 10.746000290 18.125000000 10.557999611

H 11.020999908 15.215000153 12.741999626

H 11.897000313 16.548999786 11.930000305

H 6.800000191 14.519000053 11.425000191

H 6.776000023 16.274000168 11.112000465

H 7.243000031 15.675999641 12.704999924

H 6.991000175 14.491000175 9.104000092

H 5.907999992 12.637000084 7.885000229

H 6.997000217 10.395999908 7.781000137

H 9.211000443 10.057000160 8.885999680

H 10.300000191 11.916000366 10.079999924

H 9.109000206 16.909999847 13.623000145

H 8.319999695 17.988000870 12.449000359

H 10.020000458 18.240999222 12.885999680

H 8.111000061 16.371999741 8.480999947

H 6.102000237 17.531000137 7.642000198

H 5.643000126 19.892000198 8.302000046

H 7.236000061 21.073999405 9.812000275

H 9.250000000 19.906000137 10.652999878

H 12.789999962 17.291999817 9.296999931

H 13.173000336 15.593999863 7.776999950

H 11.743000031 17.233999252 5.778999805

H 11.687000275 15.472999573 5.802000046

H 16.618000031 16.233999252 2.696000099

H 15.045999527 18.141000748 2.667999983

H 13.078000069 18.162000656 4.179999828

H 18.399999619 12.312999725 5.489999771

H 18.287000656 11.241000175 7.709000111

H 16.318000793 11.612999916 9.194000244

H 18.341999054 13.137000084 3.565999985

H 18.388999939 14.798000336 4.210000038

H 17.715999603 14.461000443 2.595000029

H 15.482000351 13.258000374 2.338000059

H 14.604000092 12.682000160 3.773999929

H 16.134000778 11.904000282 3.303999901

H 13.991999626 14.776000023 11.345999718

H 15.024000168 16.780000687 12.234999657

H 16.325000763 18.322999954 10.730999947

H 16.597000122 17.715999603 8.324000359

H 15.576999664 15.706999779 7.422999859

H 13.751999855 11.100999832 7.293000221

H 12.076000214 10.015000343 5.875999928

H 9.918000221 11.161999702 5.350999832

H 9.472999573 13.414999962 6.330999851

H 11.156000137 14.508000374 7.736999989

H 11.154000282 19.120000839 8.826999664

H 10.873000145 18.485000610 7.234000206

H 13.604000092 19.447000504 8.331000328

H 12.571999550 20.361999512 7.211999893

H 13.362000465 18.854999542 6.690000057

C 14.416999817 13.881999969 14.991999626

C 16.104000092 13.420000076 13.428000450

C 16.437999725 12.581000328 15.725999832

C 15.494999886 13.739000320 16.076999664

C 18.016000748 12.053000450 13.767999649

C 18.187000275 12.739000320 12.395000458

C 16.790000916 13.315999985 12.079000473

C 11.196999550 9.965999603 12.079000473

C 12.031999588 11.201000214 12.102999687

C 13.430999756 11.272999763 11.758000374

C 14.142000198 10.213999748 11.020999908

C 11.491000175 12.479000092 12.460000038

C 15.416000366 9.802000046 11.458999634

C 16.134000778 8.822999954 10.769000053

C 15.590999603 8.229000092 9.623999596

C 14.321000099 8.621999741 9.180999756

C 13.607000351 9.602000237 9.869000435

C 10.505999565 9.692000389 18.204999924

C 10.024999619 8.661999702 15.965000153

C 11.446999550 8.656999588 15.704999924

C 12.357000351 9.390000343 16.575000763

C 12.763999939 10.439000130 18.767999649

C 8.196999550 9.312999725 17.499000549

C 12.045999527 8.265999794 14.482999802

C 11.819000244 7.138000011 13.569000244

C 10.661999702 6.334000111 13.505000114

C 10.581999779 5.290999889 12.579000473

C 11.642000198 5.019999981 11.704999924

C 12.807999611 5.789000034 11.777000427

C 12.892000198 6.830999851 12.701000214

N 14.951000214 13.977000237 13.623000145

N 16.902000427 12.784000397 14.359000206

N 11.842000008 9.805000305 17.822000504

N 9.631999969 9.260000229 17.204999924

O 11.453000069 9.871999741 13.463000298

O 10.135000229 9.991999626 19.344999313

O 13.550000191 9.638999939 16.320999146

O 9.126999855 8.246999741 15.210000038

H 13.727999687 13.022000313 15.045000076

H 13.810000420 14.779000282 15.180000305

H 17.311000824 12.562999725 16.393999100

H 15.914999962 11.614999771 15.824999809

H 16.076000214 14.670000076 16.142000198

H 15.036999702 13.562000275 17.059999466

H 18.910999298 12.133000374 14.401000023

H 17.775999069 10.980999947 13.666000366

H 18.544000626 12.043000221 11.626999855

H 18.922000885 13.550000191 12.477999687

H 16.200000763 12.640999794 11.442999840

H 16.819000244 14.286000252 11.569000244

H 14.048999786 11.838999748 12.465000153

H 10.133999825 10.116999626 11.819999695

H 11.605999947 9.133000374 11.484999657

H 12.050000191 13.083999634 13.180000305

H 10.406999588 12.586999893 12.505999565

H 15.829999924 10.251000404 12.361000061

H 17.114999771 8.519000053 11.128000259

H 16.150999069 7.472000122 9.079000473

H 13.892000198 8.168999672 8.289999962

H 12.640000343 9.932999611 9.494000435

H 12.319999695 10.380999565 19.764999390

H 12.918999672 11.493000031 18.499000549

H 13.729000092 9.925999641 18.725999832

H 8.043000221 10.034000397 18.305999756

H 7.817999840 8.331999779 17.812000275

H 7.668000221 9.614000320 16.589000702

H 9.833999634 6.559999943 14.170999527

H 9.678000450 4.685999870 12.534000397

H 11.560000420 4.217000008 10.975000381

H 13.649000168 5.583000183 11.119000435

H 13.803000450 7.425000191 12.760999680

H 13.095999718 8.562999725 14.487999916

TS4 -780.860568 a.u.

C 9.947999954 15.760000229 12.189000130

C 10.906000137 15.956000328 9.791000366

C 10.145999908 16.714000702 10.927000046

C 11.336999893 15.123000145 12.312000275

C 9.027000427 14.614999771 11.767000198

C 9.616999626 13.782999992 10.862000465

C 8.977999687 12.748999596 10.027999878

C 7.631999969 14.515999794 12.305000305

C 7.659999847 12.914999962 9.543999672

C 7.053999901 11.939000130 8.750000000

C 7.744999886 10.767999649 8.418999672

C 9.059000015 10.600000381 8.864000320

C 9.668999672 11.586000443 9.640000343

C 9.529999733 16.531000137 13.439000130

C 8.866000175 17.395999908 10.494000435

C 7.943999767 16.790000916 9.626000404

C 6.743999958 17.424999237 9.293999672

C 6.442999840 18.686000824 9.819000244

C 7.357999802 19.308000565 10.673999786

C 8.555000305 18.666999817 11.003999710

C 12.022999763 16.841999054 9.116000175

C 12.218000412 16.370000839 6.550000191

C 13.390999794 16.513999939 5.580999851

C 14.399000168 15.538999557 5.428999901

C 15.439000130 15.647000313 4.494999886

C 15.508999825 16.822000504 3.730999947

C 14.548000336 17.822999954 3.871999979

C 13.494000435 17.656000137 4.775000095

C 15.496999741 13.717000008 6.531000137

C 16.555000305 13.727999687 5.611999989

C 17.714000702 13.012000084 5.951000214

C 17.799999237 12.293000221 7.144999981

C 16.733999252 12.298000336 8.050999641

C 15.571999550 13.022999763 7.767000198

C 16.339000702 14.428999901 4.269000053

C 17.666999817 14.810000420 3.591000080

C 15.564000130 13.442999840 3.345000029

C 12.866000175 12.746999741 7.760000229

C 14.750000000 14.920999527 9.560999870

C 14.673999786 15.116999626 10.953000069

C 15.199000359 16.257999420 11.564999580

C 15.814999580 17.243999481 10.791999817

C 15.935000420 17.052000046 9.411999702

C 15.425999641 15.902000427 8.803999901

C 13.173000336 11.534000397 7.105999947

C 12.253999710 10.932999611 6.242000103

C 11.012000084 11.526000023 6.004000187

C 10.692999840 12.715999603 6.660999775

C 11.605999947 13.309000015 7.535999775

C 11.529999733 18.274000168 8.812000275

C 12.666000366 19.194999695 8.350999832

P 11.371999741 14.291999817 10.689999580

P 14.196000099 13.230999947 8.979999542

N 12.675000191 16.299999237 7.929999828

O 14.324000359 14.411999702 6.252999783

Pd 12.925000191 12.524000168 10.864999771

H 10.199999809 15.644000053 9.010000229

H 10.828000069 17.490999222 11.303999901

H 11.404000282 14.394000053 13.126000404

H 12.149000168 15.855999947 12.411000252

H 7.170000076 13.553999901 12.057000160

H 6.994999886 15.324999809 11.914999962

H 7.640999794 14.621000290 13.399999619

H 7.114999771 13.826000214 9.777000427

H 6.040999889 12.095999718 8.385999680

H 7.269000053 10.003000259 7.808000088

H 9.619999886 9.708000183 8.595000267

H 10.711000443 11.473999977 9.937999725

H 9.477000237 15.862999916 14.309000015

H 8.552000046 17.013999939 13.317000389

H 10.265999794 17.315000534 13.666000366

H 8.159999847 15.807999611 9.208999634

H 6.045000076 16.934000015 8.619999886

H 5.510000229 19.180999756 9.559000015

H 7.142000198 20.294000626 11.081000328

H 9.263999939 19.159999847 11.668000221

H 12.828000069 16.941999435 9.862999916

H 13.204000473 15.461000443 8.114000320

H 11.574999809 17.249000549 6.426000118

H 11.616000175 15.496000290 6.239999771

H 16.306999207 16.947999954 3.003999949

H 14.609000206 18.724000931 3.266999960

H 12.734999657 18.430000305 4.868000031

H 18.555000305 13.001999855 5.263000011

H 18.700000763 11.723999977 7.368000031

H 16.798000336 11.737999916 8.982999802

H 18.277999878 13.918999672 3.400000095

H 18.252000809 15.510000229 4.201000214

H 17.482999802 15.270999908 2.611999989

H 15.345000267 13.920000076 2.380000114

H 14.612000465 13.142000198 3.802999973

H 16.162000656 12.539999962 3.167000055

H 14.262000084 14.340999603 11.604000092

H 15.145000458 16.333999634 12.649000168

H 16.221000671 18.138999939 11.260000229

H 16.430000305 17.799999237 8.795999527

H 15.562000275 15.798000336 7.735000134

H 14.135999680 11.053000450 7.263999939

H 12.520000458 9.998000145 5.751999855

H 10.298000336 11.060999870 5.328000069

H 9.718999863 13.178999901 6.513000011

H 11.335000038 14.236000061 8.026000023

H 11.067999840 18.701000214 9.708000183

H 10.718999863 18.239999771 8.069000244

H 13.454999924 19.242000580 9.114000320

H 12.298999786 20.215999603 8.180000305

H 13.140999794 18.840000153 7.428999901

C 14.838999748 13.579000473 15.248000145

C 16.478000641 13.159000397 13.611000061

C 16.961999893 12.409000397 15.904000282

C 15.965999603 13.506999969 16.291000366

C 18.465999603 11.909999847 13.899000168

C 18.544000626 12.550999641 12.498000145

C 17.104999542 13.043000221 12.232000351

C 12.543999672 10.755000114 12.225000381

C 13.925999641 10.793999672 11.850000381

C 14.623999596 9.802000046 11.022999763

C 15.895000458 9.361000061 11.439999580

C 16.611000061 8.427000046 10.689999580

C 16.073999405 7.920000076 9.501999855

C 14.817999840 8.359999657 9.067999840

C 14.102000237 9.293000221 9.815999985

C 10.439999580 8.748999596 17.993999481

C 12.381999969 9.161999702 16.482999802

C 11.475000381 9.244000435 15.376999855

C 10.059000015 9.387999535 15.595999718

C 8.175000191 8.909999847 17.145999908

C 12.715999603 8.711999893 18.884000778

C 12.071999550 11.833000183 12.989999771

C 11.968999863 8.621999741 14.081999779

C 11.583999634 9.637999535 11.857999802

C 11.897999763 7.102000237 14.241999626

C 13.031000137 6.348000050 14.572999954

C 12.935000420 4.959000111 14.732000351

C 11.706999779 4.313000202 14.562000275

C 10.569000244 5.065999985 14.243000031

C 10.661000252 6.448999882 14.092000008

N 15.291999817 13.625000000 13.845999718

N 17.378999710 12.654000282 14.529999733

N 9.612999916 9.057999611 16.923000336

N 11.802000046 8.972999573 17.774000168

O 9.989999771 8.347000122 19.077999115

O 9.201000214 9.720999718 14.756999969

O 13.631999969 9.199000359 16.400999069

O 11.093999863 8.951999664 12.991999626

H 14.171999931 12.710000038 15.373999596

H 14.220999718 14.470999718 15.427000046

H 17.850999832 12.428000450 16.551000595

H 16.496999741 11.413000107 16.000999451

H 16.495000839 14.468999863 16.337999344

H 15.553000450 13.305999756 17.288999557

H 19.395000458 12.017999649 14.475999832

H 18.229000092 10.833000183 13.845000267

H 18.900999069 11.843999863 11.739999771

H 19.239000320 13.399000168 12.520000458

H 16.527000427 12.317999840 11.640999794

H 17.059999466 13.996999741 11.694000244

H 14.565999985 11.371000290 12.526000023

H 16.304000854 9.744999886 12.373999596

H 17.586000443 8.092000008 11.036999702

H 16.632999420 7.195000172 8.913999557

H 14.399000168 7.982999802 8.137000084

H 13.151000023 9.670000076 9.442999840

H 7.958000183 7.889999866 17.482999802

H 7.685999870 9.119999886 16.190000534

H 7.828000069 9.611000061 17.913999557

H 13.105999947 7.686999798 18.829999924

H 12.163000107 8.840999603 19.818000793

H 13.559000015 9.406000137 18.812000275

H 12.772999763 12.444999695 13.560999870

H 11.015000343 11.892000198 13.246999741

H 13.012999535 8.916000366 13.892000198

H 10.678999901 10.062000275 11.404000282

H 12.045999527 8.946000099 11.140000343

H 13.982999802 6.855999947 14.713000298

H 13.821999550 4.381999969 14.980999947

H 11.633000374 3.233999968 14.677000046

H 9.609000206 4.572000027 14.109000206

H 9.781999588 7.041999817 13.845999718

Coordinates and their Gibbs energy without the DBN

Int 1’ -714.2323398 a.u.

C 9.984100 15.939700 12.082800

C 10.868900 15.981100 9.653500

C 10.140900 16.811001 10.758500

C 11.368400 15.286700 12.183400

C 9.029700 14.787500 11.775000

C 9.566600 13.903500 10.885100

C 8.872900 12.852700 10.121800

C 7.664400 14.738600 12.392700

C 7.516000 12.993500 9.748900

C 6.860700 12.003500 9.014700

C 7.543000 10.847500 8.619200

C 8.894400 10.707700 8.946500

C 9.549000 11.700800 9.674500

C 9.627100 16.803699 13.292200

C 8.850500 17.473101 10.324800

C 7.892800 16.813700 9.537500

C 6.687100 17.435200 9.198400

C 6.416300 18.737101 9.631800

C 7.363800 19.411501 10.407800

C 8.565200 18.782700 10.747400

C 11.998700 16.826099 8.947100

C 12.212400 16.202200 6.426300

C 13.370500 16.270000 5.429500

C 14.371900 15.280500 5.328100

C 15.379900 15.301500 4.352300

C 15.433300 16.410700 3.496200

C 14.484600 17.430099 3.589500

C 13.456400 17.342600 4.532100

C 15.494500 13.539800 6.527300

C 16.521000 13.465600 5.580000

C 17.683300 12.766400 5.943500

C 17.797400 12.144500 7.189600

C 16.749701 12.220400 8.112800

C 15.587700 12.931500 7.800400

C 16.265800 14.061400 4.195000

C 17.575300 14.375500 3.453200

C 15.456300 13.011100 3.377400

C 12.890700 12.646300 7.798800

C 14.767100 14.919400 9.480500

C 14.665100 15.193600 10.859900

C 15.173400 16.370399 11.415500

C 15.808000 17.309601 10.598400

C 15.951900 17.041000 9.234400

C 15.451300 15.858700 8.684000

C 13.185000 11.395800 7.213100

C 12.264200 10.756500 6.378700

C 11.028400 11.347300 6.099700

C 10.717400 12.572000 6.693500

C 11.631600 13.201600 7.541200

C 11.518500 18.241501 8.553000

C 12.664600 19.130600 8.051700

P 11.336000 14.335600 10.617200

P 14.221600 13.189100 9.005500

N 12.678100 16.222900 7.805800

O 14.323700 14.237900 6.248600

Pd 12.875200 12.534300 10.775700

H 10.153300 15.654800 8.888200

H 10.833100 17.609699 11.066200

H 11.464800 14.624400 13.050200

H 12.187600 16.017700 12.192700

H 7.201200 13.754000 12.261200

H 6.998400 15.504500 11.963900

H 7.727800 14.945000 13.470700

H 6.976700 13.896900 10.019800

H 5.816000 12.140600 8.743400

H 7.030700 10.074800 8.050200

H 9.451000 9.831100 8.621800

H 10.613000 11.601600 9.891600

H 9.596600 16.201099 14.209900

H 8.652000 17.294399 13.173600

H 10.381500 17.589600 13.434900

H 8.087000 15.801000 9.190200

H 5.960800 16.901100 8.589000

H 5.480500 19.221901 9.362700

H 7.170400 20.427401 10.745500

H 9.300400 19.316200 11.348600

H 12.790800 16.966200 9.701700

H 13.188400 15.385200 8.048000

H 11.575700 17.076799 6.248500

H 11.598700 15.316700 6.180200

H 16.209999 16.471901 2.739100

H 14.536200 18.283400 2.917500

H 12.702500 18.126200 4.585200

H 18.506599 12.692900 5.237900

H 18.702101 11.594900 7.438600

H 16.825300 11.732000 9.082900

H 18.174400 13.466300 3.320300

H 18.180201 15.115900 3.992900

H 17.369400 14.760000 2.446700

H 15.210400 13.409800 2.384100

H 14.518700 12.753700 3.887500

H 16.043501 12.091100 3.254800

H 14.191700 14.447400 11.502500

H 15.080000 16.542700 12.485900

H 16.201700 18.232100 11.020800

H 16.454599 17.754900 8.585500

H 15.597300 15.691900 7.624000

H 14.138000 10.910900 7.411900

H 12.523000 9.792000 5.945800

H 10.314000 10.853700 5.444900

H 9.747800 13.034600 6.520900

H 11.360100 14.146200 7.994000

H 11.047800 18.724600 9.416000

H 10.717200 18.169300 7.803300

H 13.450800 19.213499 8.815100

H 12.306400 20.143299 7.824100

H 13.142600 18.723400 7.152800

C 12.221900 11.151000 12.401100

C 13.585900 10.938500 12.038200

C 14.148000 9.794900 11.278000

C 15.303700 9.164000 11.781400

C 15.905200 8.100500 11.102900

C 15.365400 7.637600 9.899000

C 14.220500 8.255300 9.381800

C 13.625300 9.321500 10.057600

C 9.890900 11.208900 17.346600

C 12.046100 11.264200 16.109501

C 11.298500 11.052900 14.810000

C 9.798200 11.250600 14.882400

C 7.738800 11.027700 16.236000

C 11.982600 11.449000 18.570200

C 11.895100 11.942200 13.659000

C 11.586000 9.515700 14.397200

C 11.147900 10.149000 12.079100

C 11.156600 8.524000 15.455600

C 12.044400 8.165300 16.481800

C 11.632000 7.312100 17.510099

C 10.331100 6.798900 17.516600

C 9.449700 7.134500 16.483101

C 9.858900 7.992400 15.458400

N 9.202400 11.116600 16.138300

N 11.286300 11.363800 17.278200

O 9.291400 11.195700 18.415600

O 9.111800 11.458500 13.886000

O 13.277800 11.290000 16.145000

O 10.936500 9.200600 13.192600

H 14.318600 11.403300 12.705500

H 15.732000 9.519500 12.717400

H 16.801201 7.638500 11.512500

H 15.837300 6.816800 9.363700

H 13.798900 7.920800 8.435800

H 12.778600 9.835500 9.605800

H 7.476300 10.146600 16.830900

H 7.351900 10.946200 15.217600

H 7.331800 11.919600 16.725401

H 11.975600 10.469000 19.063601

H 11.468800 12.172500 19.209299

H 13.011300 11.756000 18.368099

H 12.796000 12.442500 14.032100

H 11.132500 12.693800 13.427900

H 12.684400 9.495100 14.292600

H 10.184500 10.645200 11.923700

H 11.389900 9.525000 11.213500

H 13.056600 8.566500 16.476900

H 12.328700 7.044100 18.301500

H 10.009000 6.135300 18.316200

H 8.440300 6.728700 16.475901

H 9.183500 8.256000 14.647300

Int 2' -227.5397093 a.u.

N 11.548600 11.803400 16.752199

N 10.217200 10.174600 15.628100

C 10.274100 11.278800 16.492201

C 11.302000 9.640300 14.914700

C 12.689200 10.140700 15.263500

C 12.743000 11.308800 16.243099

C 11.588400 12.933700 17.695101

C 8.900400 9.573400 15.366800

C 13.457300 10.571600 13.972400

C 13.517900 8.945100 15.943300

C 14.607600 8.328900 13.904800

C 12.761800 8.371200 17.120600

C 12.886200 8.964900 18.385900

C 12.113400 8.510600 19.458900

C 11.217500 7.452100 19.278999

C 11.107700 6.842900 18.024599

C 11.874100 7.299000 16.948000

C 13.939500 9.426000 13.125100

C 13.787900 9.446300 11.783000

C 14.298700 8.491300 10.791400

C 15.556300 7.864300 10.903600

C 15.995900 6.956700 9.936500

C 15.196900 6.665100 8.827000

C 13.958400 7.301200 8.684000

C 13.519900 8.207800 9.649300

O 9.285500 11.792300 17.007601

O 13.818500 11.805000 16.577200

O 11.111200 8.774300 14.065500

O 13.797200 7.905500 15.023300

H 11.226900 12.608200 18.676800

H 10.940500 13.737100 17.330700

H 12.626700 13.265800 17.759701

H 8.644500 9.680200 14.307000

H 8.168200 10.086700 15.993800

H 8.944200 8.506400 15.612400

H 12.837400 11.259200 13.381200

H 14.331500 11.140300 14.329700

H 14.452200 9.424900 16.283501

H 14.746400 7.425400 13.300800

H 15.593800 8.659400 14.281800

H 13.586900 9.786400 18.527300

H 12.216400 8.978900 20.435301

H 10.616200 7.097800 20.113600

H 10.423100 6.009200 17.882900

H 11.791000 6.837200 15.967000

H 16.210199 8.120700 11.733900

H 16.971500 6.487200 10.043400

H 15.541400 5.960100 8.073600

H 13.334800 7.090800 7.817700

H 12.553600 8.696300 9.535700

H 13.191800 10.262700 11.369500

Int (2+3)' -227.5397093 a.u.

C 9.922100 16.140301 12.497500

C 10.789500 15.812500 10.094100

C 10.183100 16.843000 11.095800

C 11.233000 15.351900 12.692500

C 8.851500 15.067900 12.303800

C 9.319000 14.009300 11.592700

C 8.579300 12.828500 11.119600

C 7.462500 15.248500 12.836300

C 7.368600 12.948800 10.410800

C 6.682600 11.816600 9.961400

C 7.189300 10.539400 10.219000

C 8.389600 10.402500 10.924700

C 9.081800 11.533500 11.358600

C 9.641800 17.158800 13.603100

C 8.996200 17.620300 10.570300

C 7.951100 17.003700 9.863000

C 6.845300 17.736700 9.423600

C 6.765700 19.110500 9.673600

C 7.802200 19.742001 10.367600

C 8.902200 19.001499 10.809900

C 12.007900 16.391500 9.268000

C 11.718000 15.612900 6.824200

C 12.663100 15.550100 5.626900

C 13.510600 14.451800 5.372900

C 14.278400 14.331000 4.204300

C 14.274800 15.410600 3.309400

C 13.492300 16.539900 3.554200

C 12.678000 16.589701 4.689000

C 14.631600 12.589600 6.372600

C 15.409800 12.372100 5.228900

C 16.525400 11.529800 5.359700

C 16.830400 10.910800 6.574100

C 16.024200 11.127800 7.695500

C 14.919400 11.981300 7.615700

C 14.941100 12.982900 3.908400

C 16.084999 13.108800 2.888800

C 13.842900 12.045400 3.322300

C 12.265700 11.966600 8.228300

C 14.687000 14.072500 9.383700

C 14.716300 14.508000 10.725100

C 15.435000 15.640200 11.115900

C 16.150700 16.377600 10.167500

C 16.147900 15.955000 8.835500

C 15.436900 14.814800 8.450300

C 12.281600 10.655300 7.698500

C 11.147200 10.109100 7.093000

C 9.950800 10.830200 7.035000

C 9.910900 12.117200 7.575600

C 11.053200 12.680800 8.152700

C 11.804400 17.872000 8.859400

C 13.037300 18.465200 8.166300

P 11.100700 14.264500 11.217400

P 13.898400 12.394000 9.098200

N 12.420000 15.628900 8.097800

O 13.540600 13.452900 6.336500

Pd 12.279900 12.432300 10.765400

H 10.025200 15.492400 9.374900

H 10.972300 17.571400 11.331500

H 11.233800 14.750900 13.610000

H 12.127500 15.988800 12.667400

H 6.851600 14.352500 12.675800

H 6.963200 16.100901 12.349200

H 7.482100 15.470400 13.913600

H 6.972500 13.941100 10.202900

H 5.752700 11.933700 9.408700

H 6.654100 9.656800 9.875400

H 8.781300 9.411300 11.143000

H 10.020200 11.425700 11.905700

H 9.525300 16.662201 14.575400

H 8.730100 17.737600 13.403700

H 10.475300 17.869400 13.688700

H 8.001700 15.936200 9.657900

H 6.047600 17.234900 8.879900

H 5.909200 19.683599 9.325500

H 7.757100 20.811600 10.561800

H 9.708000 19.500799 11.346400

H 12.883500 16.372200 9.935900

H 12.890900 14.760300 8.314800

H 11.132400 16.535601 6.731100

H 10.997100 14.780200 6.729800

H 14.872500 15.361300 2.403400

H 13.499000 17.368900 2.850600

H 12.039400 17.454500 4.861000

H 17.159401 11.345000 4.496500

H 17.694901 10.255200 6.647800

H 16.255301 10.650100 8.645600

H 16.515600 12.125000 2.665100

H 16.886000 13.764200 3.255300

H 15.715200 13.508900 1.936700

H 13.442300 12.464500 2.389600

H 13.012500 11.923300 4.030500

H 14.263500 11.052400 3.113900

H 14.164300 13.926700 11.466700

H 15.443000 15.935600 12.163300

H 16.714701 17.259800 10.463900

H 16.701799 16.512800 8.083100

H 15.479800 14.515100 7.409200

H 13.185500 10.053900 7.770000

H 11.201200 9.105500 6.674700

H 9.064600 10.395100 6.579000

H 8.987600 12.692100 7.558400

H 11.010100 13.696800 8.521500

H 11.579500 18.470301 9.749600

H 10.911900 17.969000 8.224500

H 13.925900 18.366100 8.805500

H 12.891000 19.531900 7.951000

H 13.268400 17.949699 7.226800

C 11.111100 6.389600 11.585300

C 13.168100 7.528300 10.782300

C 14.027600 6.620000 11.642400

C 13.278800 5.641000 12.539700

C 11.146000 4.695000 13.336600

C 11.013100 8.058900 9.781800

C 14.981000 7.462000 12.547700

C 14.944300 5.726300 10.682600

C 16.787701 7.273600 10.812600

C 14.100500 4.970000 9.677700

C 13.551800 3.724700 10.018900

C 12.693200 3.063400 9.134700

C 12.382400 3.637500 7.898300

C 12.941500 4.870800 7.547100

C 13.796400 5.536300 8.430300

C 16.057699 8.168000 11.773500

C 16.291500 9.484200 11.971400

C 17.292200 10.362700 11.359800

C 18.574699 9.943800 10.948500

C 19.452499 10.834600 10.325000

C 19.078600 12.165600 10.112200

C 17.826300 12.609000 10.551300

C 16.952299 11.720700 11.176400

N 11.888800 5.643300 12.491300

N 11.791100 7.289900 10.764400

O 9.892900 6.249000 11.546000

O 13.888600 4.866100 13.280200

O 13.633000 8.417600 10.075300

O 15.883600 6.511900 9.979000

H 10.745500 3.879700 12.722600

H 10.312900 5.216300 13.817100

H 11.845900 4.299300 14.075900

H 11.120400 9.133200 9.966300

H 9.969600 7.751000 9.859600

H 11.402000 7.845600 8.781300

H 14.395500 8.175800 13.143100

H 15.436000 6.741500 13.246800

H 15.450200 5.022600 11.366500

H 17.394100 7.845300 10.101900

H 17.446699 6.573400 11.359500

H 13.795700 3.276400 10.980800

H 12.273100 2.098400 9.409800

H 11.714300 3.123900 7.210100

H 12.711800 5.317100 6.581800

H 14.232500 6.497500 8.168700

H 18.902201 8.927300 11.152500

H 20.436600 10.489300 10.015200

H 19.761700 12.854700 9.620600

H 17.520901 13.642500 10.398200

H 15.970200 12.066000 11.494700

H 15.599700 9.997200 12.643400

Int 3’ -486.6830351 a.u.

C 8.689100 14.534400 12.004400

C 10.153900 15.097100 9.961300

C 9.062300 15.645800 10.931100

C 10.085500 14.021100 12.410500

C 8.043500 13.359100 11.272400

C 8.945600 12.671800 10.525000

C 8.704000 11.545300 9.609600

C 6.575800 13.080500 11.388500

C 7.711100 11.601700 8.612700

C 7.500100 10.525300 7.746000

C 8.272700 9.366100 7.863600

C 9.261400 9.293000 8.850800

C 9.482200 10.374000 9.704800

C 7.879800 15.111800 13.166100

C 7.862100 16.269100 10.252700

C 7.231700 15.671600 9.148900

C 6.099300 16.246201 8.565600

C 5.576900 17.442101 9.068800

C 6.198100 18.055500 10.160800

C 7.326800 17.471800 10.743500

C 11.316700 16.133101 9.684100

C 11.842800 16.039400 7.158900

C 13.020900 16.523001 6.316800

C 14.180000 15.749300 6.099800

C 15.202900 16.132700 5.218700

C 15.104000 17.395800 4.617700

C 13.998700 18.214899 4.852300

C 12.960600 17.764900 5.672800

C 15.515000 13.962900 6.966200

C 16.569099 14.251100 6.090600

C 17.814400 13.664200 6.366700

C 17.987801 12.804900 7.454100

C 16.912901 12.516700 8.300400

C 15.662600 13.101700 8.077300

C 16.270800 15.098200 4.853600

C 17.538500 15.749800 4.277000

C 15.653600 14.154900 3.777700

C 13.055600 12.348300 7.845800

C 14.437700 14.503300 10.099300

C 14.025900 14.534700 11.448100

C 14.281100 15.634700 12.270300

C 14.960500 16.746300 11.762300

C 15.391000 16.730101 10.432900

C 15.144000 15.622600 9.616600

C 13.566800 11.277000 7.076900

C 12.809600 10.697200 6.055900

C 11.506500 11.132800 5.797100

C 10.977300 12.173300 6.563800

C 11.743700 12.779100 7.564400

C 10.803900 17.589701 9.561000

C 11.944900 18.604099 9.414800

P 10.617900 13.396700 10.766500

P 14.247700 12.847800 9.236700

N 12.191300 15.841200 8.556800

O 14.264500 14.547700 6.790000

Pd 12.340000 12.064200 10.307700

H 9.703200 14.839900 8.994500

H 9.534000 16.427601 11.543600

H 10.040900 13.198400 13.134300

H 10.742400 14.814200 12.792700

H 6.299600 12.158500 10.863400

H 5.984500 13.909400 10.968700

H 6.277700 12.985200 12.443200

H 7.110400 12.503900 8.513400

H 6.732400 10.594000 6.977900

H 8.106300 8.525100 7.193900

H 9.855300 8.387800 8.958700

H 10.256400 10.317300 10.472100

H 7.683600 14.345900 13.928100

H 6.914900 15.515300 12.831000

H 8.433000 15.929100 13.648800

H 7.629700 14.743100 8.744500

H 5.627800 15.762100 7.712900

H 4.700000 17.894400 8.610800

H 5.807200 18.990299 10.557300

H 7.808100 17.957300 11.591500

H 11.979100 16.111300 10.563800

H 12.824400 15.067900 8.713700

H 11.058700 16.803600 7.094400

H 11.431000 15.134400 6.675800

H 15.886600 17.735001 3.944800

H 13.935300 19.191500 4.378400

H 12.081200 18.388100 5.826300

H 18.659201 13.872300 5.715400

H 18.959999 12.355300 7.641700

H 17.039700 11.848900 9.150300

H 18.270300 14.985700 3.987400

H 18.011000 16.428600 4.999000

H 17.305099 16.316000 3.366900

H 15.381700 14.726800 2.880400

H 14.749700 13.662200 4.160000

H 16.375099 13.375700 3.497400

H 13.503100 13.662600 11.846500

H 13.958400 15.614200 13.309400

H 15.165000 17.606600 12.396600

H 15.924700 17.583700 10.019900

H 15.513700 15.648100 8.597700

H 14.560900 10.887000 7.285900

H 13.242700 9.889800 5.468000

H 10.913200 10.667200 5.013700

H 9.959900 12.518800 6.393600

H 11.328800 13.614400 8.112100

H 10.215700 17.847500 10.449000

H 10.096000 17.671101 8.723500

H 12.648700 18.518801 10.254600

H 11.557100 19.631300 9.399700

H 12.526900 18.439100 8.500500

Int (3+4)’ -647.7249012 a.u.

C 9.359100 16.370100 11.968400

C 10.219300 16.455700 9.544600

C 9.567300 17.294001 10.686600

C 10.719400 15.656800 12.059600

C 8.371200 15.265000 11.593900

C 8.910700 14.372500 10.720700

C 8.234100 13.291300 9.987500

C 6.976600 15.259900 12.143900

C 6.969300 13.488800 9.393200

C 6.342700 12.468200 8.673800

C 6.968600 11.225900 8.525000

C 8.228700 11.019000 9.096300

C 8.852700 12.037900 9.815100

C 9.006300 17.184099 13.212800

C 8.328100 18.068800 10.294400

C 7.318100 17.518000 9.488700

C 6.158300 18.239000 9.190900

C 5.986100 19.535801 9.686000

C 6.987400 20.104099 10.479200

C 8.143100 19.375900 10.776000

C 11.365500 17.208000 8.777400

C 11.092500 16.608601 6.279700

C 12.020200 16.749901 5.074000

C 12.973400 15.775300 4.710000

C 13.760300 15.870600 3.551400

C 13.643100 17.032600 2.775900

C 12.740000 18.037100 3.125900

C 11.926100 17.879299 4.250900

C 14.317800 13.986700 5.545000

C 15.135800 13.997200 4.409200

C 16.371099 13.336400 4.497300

C 16.754801 12.672000 5.664500

C 15.905900 12.659000 6.776100

C 14.675500 13.321900 6.737400

C 14.594400 14.654000 3.140000

C 15.717700 15.023200 2.158000

C 13.636100 13.635200 2.453100

C 12.046100 12.919300 7.255500

C 14.243200 15.156900 8.688100

C 14.378300 15.367800 10.078200

C 15.021100 16.497700 10.591700

C 15.543800 17.459200 9.720700

C 15.426400 17.265900 8.342000

C 14.795500 16.126900 7.831600

C 12.241400 11.684400 6.599900

C 11.188200 11.037700 5.945000

C 9.913400 11.609800 5.917100

C 9.705700 12.829900 6.566500

C 10.753200 13.466200 7.234500

C 11.025500 18.691401 8.506200

C 12.185500 19.455400 7.856200

P 10.676700 14.810900 10.436100

P 13.573400 13.458000 8.221700

N 11.818000 16.578400 7.542400

O 13.107300 14.675600 5.551700

Pd 12.168300 13.222000 10.037200

H 9.460600 16.173599 8.803300

H 10.317900 18.025101 11.023200

H 10.763200 14.906100 12.858400

H 11.567100 16.349100 12.149100

H 6.438600 14.344900 11.870500

H 6.401600 16.124701 11.776900

H 6.994600 15.338300 13.241100

H 6.484800 14.458600 9.485400

H 5.369300 12.647000 8.221600

H 6.484900 10.432400 7.959500

H 8.736200 10.065100 8.967400

H 9.842200 11.882100 10.245800

H 8.941100 16.536699 14.097500

H 8.049400 17.711500 13.103300

H 9.781600 17.937799 13.408300

H 7.438100 16.511101 9.093800

H 5.389900 17.788401 8.565900

H 5.085900 20.099100 9.450100

H 6.871400 21.115000 10.864400

H 8.920300 19.826300 11.392200

H 12.252200 17.203899 9.433000

H 12.299000 15.701500 7.699000

H 10.418900 17.473900 6.276800

H 10.455000 15.721100 6.120000

H 14.249900 17.147699 1.882100

H 12.657400 18.931999 2.513800

H 11.203300 18.651300 4.509800

H 17.040001 13.333600 3.640800

H 17.715401 12.164100 5.707400

H 16.202600 12.149200 7.691400

H 16.276100 14.129000 1.854600

H 16.422400 15.741000 2.598200

H 15.305100 15.458100 1.239400

H 13.185800 14.081400 1.556400

H 12.827600 13.335300 3.133000

H 14.189600 12.732900 2.160300

H 13.953200 14.624200 10.760600

H 15.112100 16.620300 11.669200

H 16.043699 18.343500 10.111000

H 15.824800 18.005100 7.650200

H 14.739300 16.014601 6.755100

H 13.227100 11.223400 6.599600

H 11.373000 10.084600 5.452500

H 9.092300 11.109200 5.409400

H 8.714900 13.279700 6.583400

H 10.561200 14.400100 7.748400

H 10.761100 19.179600 9.451700

H 10.116100 18.762699 7.891800

H 13.089000 19.391199 8.478500

H 11.935800 20.517200 7.728900

H 12.449700 19.041901 6.875600

C 9.089600 9.558700 12.416100

C 10.353100 9.191900 13.189100

C 10.313100 9.655900 14.644800

C 10.656900 7.697400 13.086100

C 11.714700 11.120100 12.465600

C 15.878500 11.883800 10.850500

C 15.312300 11.348400 12.146500

C 14.063700 10.491200 12.039400

C 14.061700 9.528700 10.865300

C 15.846100 11.615700 13.345400

C 14.919000 8.418900 10.924600

C 14.973200 7.506900 9.868000

C 14.166900 7.694000 8.739300

C 13.312200 8.797900 8.676100

C 13.261100 9.712400 9.733400

O 11.551200 9.790900 12.509400

O 10.919000 11.988700 12.765100

O 16.839899 12.933000 11.023500

O 12.942800 11.459400 11.977000

H 8.894800 10.635800 12.452200

H 8.232000 9.030100 12.853600

H 9.177900 9.253000 11.366300

H 10.121400 10.732700 14.713200

H 11.260000 9.425600 15.149600

H 9.509000 9.123700 15.170800

H 9.837300 7.119500 13.531900

H 11.586300 7.450000 13.614600

H 10.766500 7.398600 12.035700

H 16.369200 13.778400 10.919400

H 16.386900 11.060200 10.322900

H 15.053000 12.198800 10.190500

H 13.940300 9.920800 12.968600

H 15.416800 11.213600 14.260500

H 16.717899 12.260700 13.427700

H 15.549800 8.275600 11.801100

H 15.642500 6.651300 9.925700

H 14.205300 6.983300 7.916400

H 12.679700 8.958300 7.805100

H 12.596500 10.574800 9.667700

Int 4’ -161.016955 a.u.

C 15.958800 17.686300 9.270700

C 15.757500 16.262600 9.789500

C 16.161301 15.193200 8.773800

C 16.468201 16.048700 11.126500

C 13.363100 16.091801 9.225400

C 9.646500 16.306801 10.507100

C 10.415300 15.217900 11.217100

C 11.927200 15.366000 11.130400

C 12.929700 15.390500 12.256300

C 9.818000 14.222700 11.882700

C 13.548300 14.154300 12.487600

C 14.396700 13.985400 13.585100

C 14.622400 15.045800 14.470700

C 13.996200 16.276800 14.252000

C 13.158500 16.447800 13.144900

O 14.322800 16.060400 10.166600

O 13.460500 16.408001 8.056300

O 8.238100 16.059000 10.596300

O 12.153100 15.709800 9.714300

H 15.420600 17.842300 8.329300

H 17.029600 17.862600 9.100300

H 15.606700 18.417000 10.010000

H 15.642200 15.335400 7.819600

H 15.929300 14.193900 9.164500

H 17.243700 15.251400 8.597300

H 17.552401 16.153900 10.992700

H 16.255301 15.046300 11.520100

H 16.136700 16.789499 11.865300

H 7.794700 16.674101 9.992400

H 9.910000 17.281401 10.960000

H 9.973100 16.344000 9.457700

H 11.546300 14.455800 10.337400

H 10.394600 13.444700 12.375500

H 8.733700 14.178000 11.938900

H 13.371300 13.334300 11.794300

H 14.880300 13.025100 13.751400

H 15.282400 14.912800 15.325300

H 14.167500 17.105900 14.935300

H 12.687200 17.412201 12.961300

Int (5+6)' -647.7179528 a.u.

C 9.837100 16.289301 11.822600

C 10.931800 16.231600 9.491400

C 10.117900 17.111700 10.489500

C 11.221500 15.683400 12.106500

C 8.958600 15.087900 11.471100

C 9.623200 14.154900 10.733000

C 9.054200 12.985000 10.040600

C 7.526300 15.028900 11.910200

C 7.799100 13.067700 9.400200

C 7.251300 11.966600 8.737100

C 7.951500 10.757700 8.684300

C 9.211900 10.668600 9.284800

C 9.755000 11.765200 9.952300

C 9.317500 17.184099 12.948000

C 8.876500 17.758101 9.909800

C 7.985600 17.063601 9.075400

C 6.823600 17.674000 8.594100

C 6.529600 18.999001 8.931800

C 7.410200 19.706900 9.755200

C 8.568900 19.089899 10.235900

C 12.085800 17.021601 8.762400

C 12.285800 16.254801 6.279100

C 13.452200 16.237801 5.292300

C 14.427200 15.218200 5.267900

C 15.457500 15.159300 4.317900

C 15.555100 16.218100 3.402800

C 14.631700 17.263901 3.416700

C 13.582700 17.256500 4.340300

C 15.462200 13.499100 6.576400

C 16.502399 13.337300 5.656000

C 17.615999 12.587600 6.069800

C 17.671900 12.003300 7.337200

C 16.619301 12.178600 8.241000

C 15.508900 12.945400 7.878100

C 16.316401 13.892000 4.243200

C 17.664000 14.148800 3.547500

C 15.516200 12.833300 3.427700

C 12.792900 12.810500 7.876100

C 14.800000 15.101700 9.400200

C 14.713000 15.479400 10.756500

C 15.282200 16.665100 11.227500

C 15.962900 17.511299 10.348800

C 16.091900 17.138300 9.008600

C 15.531500 15.946800 8.541900

C 13.032700 11.515500 7.370800

C 12.070100 10.854000 6.603700

C 10.847900 11.465600 6.313600

C 10.593400 12.738700 6.825200

C 11.549700 13.391200 7.606200

C 11.638700 18.426500 8.300800

C 12.800000 19.260500 7.743600

P 11.382900 14.673000 10.589200

P 14.156400 13.372600 9.034700

N 12.754000 16.342600 7.654800

O 14.326600 14.235000 6.248000

Pd 12.931800 12.885400 10.940300

H 10.258000 15.834500 8.722300

H 10.783000 17.921200 10.827400

H 11.233000 15.040000 12.993200

H 12.014400 16.438801 12.187200

H 7.090900 14.039200 11.730200

H 6.913300 15.778400 11.384400

H 7.443800 15.257100 12.983000

H 7.256900 14.010400 9.408600

H 6.281100 12.058300 8.253200

H 7.528700 9.898100 8.168300

H 9.781200 9.743100 9.212100

H 10.748300 11.693100 10.397500

H 9.201600 16.615299 13.880300

H 8.348100 17.635500 12.699200

H 10.025300 18.001301 13.143400

H 8.200300 16.032600 8.800700

H 6.149600 17.113800 7.949200

H 5.629400 19.476299 8.551200

H 7.199200 20.740900 10.019800

H 9.252400 19.649799 10.873100

H 12.878100 17.178600 9.512900

H 13.256800 15.515000 7.940700

H 11.671900 17.134100 6.050700

H 11.651800 15.371200 6.081900

H 16.349501 16.218599 2.661700

H 14.720200 18.076000 2.698900

H 12.849200 18.060900 4.334400

H 18.446600 12.443000 5.384000

H 18.535999 11.407300 7.621200

H 16.647400 11.717200 9.226400

H 18.243999 13.221300 3.468100

H 18.264999 14.890700 4.089300

H 17.510900 14.505200 2.521500

H 15.322100 13.201900 2.411500

H 14.551700 12.616800 3.905900

H 16.084299 11.895600 3.362500

H 14.206700 14.807500 11.454300

H 15.197800 16.915701 12.282900

H 16.403601 18.439899 10.706200

H 16.630199 17.776501 8.311100

H 15.672500 15.700100 7.497100

H 13.969300 11.006600 7.584000

H 12.286300 9.852800 6.235800

H 10.099600 10.952200 5.714600

H 9.636700 13.223300 6.642400

H 11.326200 14.377400 7.987300

H 11.193300 18.961700 9.146700

H 10.825900 18.339001 7.565200

H 13.599700 19.356899 8.491200

H 12.464200 20.270800 7.474900

H 13.253500 18.800100 6.857800

C 11.651700 9.781700 12.575500

C 12.466900 11.000500 12.654500

C 13.778600 11.199200 11.949700

C 14.379800 10.185200 11.056400

C 11.898900 12.375600 12.719600

C 15.752000 9.875700 11.169800

C 16.360201 8.943300 10.325800

C 15.611200 8.287800 9.342500

C 14.247300 8.580000 9.216800

C 13.643000 9.516300 10.056500

C 8.632600 7.344100 13.128400

C 8.678300 7.032600 11.624700

C 8.954800 5.547800 11.380000

C 7.377200 7.474500 10.937700

C 8.335100 11.587000 14.281900

O 12.334600 10.072600 13.813500

O 9.805300 7.723500 11.021100

O 8.673200 11.284800 13.196500

O 7.987300 11.893000 15.363500

H 14.518100 11.646900 12.625700

H 10.564500 9.884800 12.617700

H 12.011200 8.901700 12.038100

H 12.344800 12.981000 13.517800

H 10.812000 12.469400 12.694000

H 16.339500 10.374900 11.939300

H 17.420000 8.723700 10.440000

H 16.081900 7.561900 8.683600

H 13.651100 8.083000 8.453800

H 12.592600 9.764000 9.917000

H 7.829000 6.784500 13.626700

H 8.450600 8.415000 13.296100

H 9.588600 7.083100 13.600500

H 9.906700 5.258400 11.843700

H 9.022200 5.345200 10.303200

H 8.155800 4.926100 11.803400

H 6.508100 6.944900 11.351900

H 7.428100 7.272400 9.860000

H 7.213100 8.553000 11.075000

H 9.654100 8.676000 11.148900

Int (5-1)’ -37.74796777 a.u.

C 13.194300 15.277700 10.980900

O 13.623300 16.305599 10.594800

O 12.765400 14.249800 11.367000

Int (5-2)’ -44.61763422 a.u.

C 13.520400 14.078500 12.243100

C 13.174000 12.583300 12.293100

C 13.183700 11.973000 10.889700

C 14.136400 11.830800 13.223700

O 11.806300 12.412900 12.759900

H 14.533900 14.240100 11.849800

H 13.479000 14.522500 13.247900

H 12.807600 14.613000 11.602000

H 12.468600 12.495900 10.241700

H 12.897200 10.914300 10.933700

H 14.182100 12.046500 10.439900

H 15.173200 11.903200 12.866400

H 13.859700 10.770200 13.280700

H 14.104700 12.250000 14.239400

H 11.752200 12.794800 13.651700

Int 6’ -565.3501316 a.u.

C 9.736100 16.338200 11.765600

C 10.846300 16.279900 9.444100

C 10.065600 17.176001 10.452300

C 11.090400 15.670700 12.053600

C 8.812600 15.185300 11.371200

C 9.449900 14.238400 10.627400

C 8.844400 13.110200 9.895800

C 7.368200 15.192300 11.771700

C 7.632400 13.279200 9.192300

C 7.054100 12.222900 8.484300

C 7.674500 10.969400 8.458900

C 8.885200 10.790200 9.135900

C 9.466000 11.846800 9.836900

C 9.240800 17.224501 12.908400

C 8.858300 17.890100 9.880600

C 7.960400 17.269300 8.997900

C 6.829500 17.943001 8.526700

C 6.574200 19.258900 8.925300

C 7.462900 19.894501 9.797400

C 8.591000 19.215000 10.265100

C 12.028300 17.036100 8.728600

C 12.270800 16.258801 6.243400

C 13.470300 16.247601 5.292200

C 14.447400 15.228800 5.285600

C 15.503900 15.173100 4.364900

C 15.628500 16.236799 3.459900

C 14.703700 17.282101 3.455000

C 13.628900 17.270500 4.348700

C 15.436600 13.490900 6.607200

C 16.506001 13.336000 5.721100

C 17.603399 12.580900 6.167300

C 17.608801 11.982000 7.429900

C 16.521400 12.143900 8.295300

C 15.427600 12.918700 7.901300

C 16.356701 13.899700 4.306600

C 17.720400 14.148000 3.641400

C 15.564300 12.850000 3.470800

C 12.699900 12.823600 7.788500

C 14.684100 15.061800 9.417800

C 14.561000 15.417400 10.776800

C 15.125900 16.590000 11.284600

C 15.838500 17.444500 10.440100

C 16.004200 17.092100 9.098100

C 15.448300 15.913100 8.595600

C 12.954600 11.555600 7.224500

C 12.001600 10.919900 6.424100

C 10.772700 11.529500 6.159000

C 10.504000 12.775500 6.726400

C 11.451500 13.403500 7.537700

C 11.628700 18.451799 8.256100

C 12.826200 19.254499 7.729600

P 11.230000 14.686700 10.518500

P 14.029100 13.347400 9.004600

N 12.683400 16.326900 7.636400

O 14.320400 14.236200 6.249800

Pd 12.723900 12.856800 10.852300

H 10.163400 15.916300 8.666300

H 10.765000 17.947901 10.809700

H 11.062800 15.008700 12.926200

H 11.911500 16.391800 12.160200

H 6.876000 14.243600 11.529800

H 6.820400 16.006701 11.271500

H 7.271400 15.368700 12.853100

H 7.150600 14.254200 9.188400

H 6.121700 12.382000 7.946500

H 7.225500 10.146300 7.907200

H 9.391000 9.827100 9.108100

H 10.425500 11.705000 10.333200

H 9.092000 16.635401 13.823200

H 8.293100 17.721600 12.663200

H 9.979400 18.006399 13.132300

H 8.144100 16.246099 8.676200

H 6.148900 17.439100 7.843400

H 5.697000 19.784901 8.554900

H 7.281800 20.920900 10.109600

H 9.282100 19.719601 10.939100

H 12.814800 17.174601 9.488800

H 13.177600 15.499200 7.933800

H 11.666600 17.141399 6.002200

H 11.642000 15.378700 6.015500

H 16.441900 16.241199 2.739600

H 14.811100 18.097700 2.744000

H 12.896100 18.075399 4.324700

H 18.459600 12.442200 5.512300

H 18.460899 11.381600 7.739900

H 16.512100 11.666800 9.273700

H 18.296700 13.216900 3.576500

H 18.313601 14.886900 4.196000

H 17.592800 14.504600 2.612000

H 15.394400 13.224200 2.452400

H 14.588300 12.640100 3.928200

H 16.125900 11.907900 3.413600

H 14.027700 14.739300 11.447700

H 15.011900 16.824499 12.340900

H 16.274799 18.364401 10.824700

H 16.569000 17.736200 8.427600

H 15.617900 15.682700 7.551700

H 13.895800 11.047800 7.417300

H 12.231100 9.940300 6.009100

H 10.031800 11.035800 5.534600

H 9.542200 13.257400 6.564100

H 11.215000 14.370400 7.959800

H 11.174000 18.998199 9.090100

H 10.833900 18.384701 7.498900

H 13.608000 19.330999 8.498200

H 12.525000 20.273100 7.451000

H 13.290300 18.781099 6.856100

C 11.399500 9.714200 12.375600

C 12.208400 10.934500 12.504900

C 13.542200 11.159100 11.850000

C 14.197400 10.156300 10.981400

C 11.629100 12.304900 12.588700

C 15.546200 9.807800 11.204700

C 16.203600 8.880500 10.392000

C 15.528700 8.272300 9.328100

C 14.189600 8.607000 9.090500

C 13.535800 9.535600 9.901700

O 12.067000 9.980100 13.633100

H 14.244700 11.598600 12.569900

H 10.308300 9.800300 12.391500

H 11.790600 8.859600 11.816600

H 12.049800 12.890100 13.415200

H 10.540100 12.392100 12.546400

H 16.076200 10.271300 12.035700

H 17.243401 8.629300 10.592800

H 16.037800 7.552300 8.691800

H 13.654900 8.152000 8.258700

H 12.509700 9.822900 9.678200

Int (6+7)’ -714.2211328 a.u.

C 9.644400 16.535700 11.605500

C 10.777800 16.425100 9.293400

C 9.992500 17.346201 10.278300

C 10.979100 15.836100 11.906400

C 8.691800 15.403200 11.220100

C 9.305100 14.439800 10.476700

C 8.674400 13.333700 9.733300

C 7.249400 15.448900 11.623900

C 7.453500 13.527300 9.050700

C 6.853600 12.488200 8.335600

C 7.457600 11.227400 8.282800

C 8.677600 11.025200 8.935400

C 9.282100 12.065700 9.639600

C 9.173700 17.450500 12.735800

C 8.790300 18.050501 9.682600

C 7.903600 17.410801 8.801500

C 6.766700 18.065701 8.319400

C 6.496700 19.383600 8.700700

C 7.376800 20.040300 9.566000

C 8.509200 19.377600 10.048900

C 11.995600 17.152201 8.608600

C 12.337600 16.329599 6.147400

C 13.578900 16.279699 5.252600

C 14.519100 15.226900 5.282100

C 15.609000 15.136100 4.403400

C 15.807700 16.198400 3.509500

C 14.923600 17.277201 3.473800

C 13.813000 17.299801 4.322300

C 15.398500 13.451900 6.629100

C 16.486200 13.247500 5.776700

C 17.523600 12.428400 6.253300

C 17.456600 11.825800 7.512600

C 16.357100 12.049200 8.349100

C 15.322000 12.884000 7.921800

C 16.419001 13.833900 4.365000

C 17.820601 14.044600 3.767100

C 15.631200 12.826000 3.476100

C 12.601100 12.907400 7.693000

C 14.621100 15.087800 9.376200

C 14.480600 15.445800 10.733400

C 15.095300 16.584101 11.261300

C 15.872300 17.403099 10.439100

C 16.049400 17.049801 9.098800

C 15.444900 15.904000 8.576500

C 12.837700 11.636600 7.126600

C 11.885900 11.026700 6.304800

C 10.678000 11.667100 6.017300

C 10.429000 12.919300 6.581500

C 11.374400 13.521000 7.414600

C 11.628200 18.562300 8.095700

C 12.855900 19.351999 7.623300

P 11.098400 14.835700 10.382800

P 13.902700 13.399600 8.953000

N 12.684300 16.414600 7.556900

O 14.322700 14.240300 6.243000

Pd 12.533700 12.971800 10.742400

H 10.108600 16.074800 8.497900

H 10.692800 18.121500 10.626500

H 10.931100 15.184500 12.786200

H 11.819100 16.535900 12.008500

H 6.735600 14.507300 11.399500

H 6.717800 16.267099 11.112600

H 7.161300 15.644600 12.702800

H 6.980500 14.506200 9.070300

H 5.914200 12.665700 7.815900

H 6.988300 10.416900 7.729200

H 9.172400 10.057200 8.886200

H 10.250500 11.906300 10.111500

H 9.010800 16.878901 13.659300

H 8.239200 17.969101 12.485000

H 9.933600 18.215200 12.947800

H 8.098400 16.386101 8.492600

H 6.093600 17.545799 7.640700

H 5.614500 19.895500 8.322400

H 7.183900 21.068600 9.864700

H 9.191200 19.897200 10.720800

H 12.754100 17.295099 9.397000

H 13.168300 15.595000 7.890600

H 11.763400 17.219400 5.862900

H 11.702800 15.458200 5.903800

H 16.648701 16.175501 2.822100

H 15.089100 18.091400 2.772200

H 13.110400 18.129900 4.272300

H 18.390600 12.243300 5.624300

H 18.263399 11.176200 7.843900

H 16.294500 11.574100 9.326800

H 18.369301 13.096200 3.716900

H 18.410601 14.756900 4.358500

H 17.754000 14.416900 2.737500

H 15.520700 13.222200 2.457800

H 14.628600 12.643300 3.885100

H 16.162100 11.865700 3.427500

H 13.897000 14.794100 11.388200

H 14.970400 16.818300 12.316500

H 16.348499 18.295799 10.840000

H 16.662901 17.666901 8.445800

H 15.625000 15.672800 7.534500

H 13.764700 11.108400 7.331800

H 12.100000 10.044100 5.888600

H 9.937800 11.193500 5.376700

H 9.483800 13.426400 6.399000

H 11.154600 14.492400 7.835800

H 11.129300 19.122801 8.894300

H 10.876500 18.484301 7.296800

H 13.589300 19.449699 8.435900

H 12.574100 20.362801 7.299900

H 13.371800 18.855200 6.792800

C 11.048800 9.858300 11.961600

C 11.885900 11.031400 12.252400

C 13.279700 11.250400 11.752200

C 14.015100 10.223200 10.984400

C 11.344400 12.406600 12.414900

C 15.284800 9.795700 11.427200

C 16.004900 8.819500 10.732500

C 15.475000 8.243600 9.572300

C 14.218000 8.661100 9.116400

C 13.500200 9.634700 9.812000

C 10.623700 10.339900 17.664101

C 12.558400 9.653400 16.249399

C 11.720500 8.582400 15.653700

C 10.291900 8.536300 15.969000

C 8.395600 9.392400 17.253300

C 12.802200 11.421500 17.946301

C 12.363200 7.890600 14.653800

C 12.035200 6.868000 13.678900

C 10.934500 5.982600 13.713000

C 10.749800 5.051800 12.690500

C 11.633200 4.994700 11.605200

C 12.727300 5.866500 11.552000

C 12.935800 6.775000 12.585700

N 9.829000 9.449700 16.937700

N 11.969600 10.412700 17.278999

O 11.627400 9.984800 13.282800

O 10.162300 11.028800 18.574499

O 9.456600 7.793300 15.445500

O 13.715700 9.897200 15.894600

H 13.894600 11.669500 12.559700

H 9.962500 9.978900 11.914000

H 11.448800 9.027800 11.373100

H 11.757200 12.925100 13.288200

H 10.261300 12.531400 12.341000

H 15.696400 10.230200 12.337400

H 16.980000 8.505600 11.100700

H 16.035500 7.486800 9.028300

H 13.798800 8.231700 8.208100

H 12.540100 9.978000 9.431100

H 8.189500 10.160600 18.000999

H 8.137800 8.399800 17.639900

H 7.816700 9.566700 16.340000

H 12.434100 11.555600 18.966299

H 12.748600 12.379100 17.412901

H 13.836700 11.068400 17.933701

H 10.229400 6.053700 14.534000

H 9.901800 4.371600 12.734800

H 11.469700 4.274100 10.806000

H 13.418100 5.843200 10.711800

H 13.784400 7.457100 12.539500

H 13.387300 8.255200 14.540500

Int 7' -148.8607534 a.u.

N 12.757700 10.783600 11.101800

N 14.154100 10.967600 13.017900

C 13.585400 10.148800 12.032900

C 13.999200 12.364600 13.103400

C 13.097300 12.994200 12.093800

C 12.530600 12.166300 11.021600

C 12.159200 9.960500 10.040300

C 15.031300 10.291000 13.985400

C 12.904400 14.342800 12.293300

C 12.147700 15.429300 11.703300

C 11.276900 15.363300 10.588400

C 10.600400 16.500999 10.154000

C 10.774900 17.730200 10.801500

C 11.635200 17.820299 11.902500

C 12.307900 16.686600 12.346800

O 13.806100 8.941600 11.986600

O 11.883000 12.589200 10.060000

O 14.584100 13.011400 13.976200

H 12.353800 8.913700 10.281600

H 12.601900 10.218900 9.071400

H 11.083900 10.161300 9.994000

H 15.929800 9.915400 13.481700

H 14.498700 9.444600 14.430200

H 15.304300 11.027600 14.744600

H 11.159900 14.413200 10.078200

H 9.935800 16.430401 9.295600

H 10.246800 18.614201 10.448500

H 11.778600 18.771999 12.409900

H 12.973600 16.755699 13.206100

H 13.481500 14.672700 13.163100

Int 8’ -714.2084084 a.u.

C 9.884500 16.210400 11.969700

C 10.907900 16.153099 9.597300

C 10.149200 17.037300 10.635300

C 11.250700 15.543600 12.200100

C 8.941200 15.056000 11.628900

C 9.537200 14.111200 10.846700

C 8.905100 12.991200 10.127900

C 7.523000 15.058100 12.110000

C 7.631400 13.132000 9.535500

C 7.035600 12.071400 8.849000

C 7.696100 10.843900 8.734500

C 8.968200 10.694300 9.295100

C 9.564400 11.757600 9.970700

C 9.449100 17.108700 13.126000

C 8.904600 17.718901 10.101500

C 7.983600 17.063801 9.268800

C 6.815300 17.703300 8.842800

C 6.546900 19.018101 9.235700

C 7.459100 19.688200 10.056500

C 8.623700 19.042299 10.481400

C 12.065500 16.927601 8.855500

C 12.293300 16.248301 6.329000

C 13.507100 16.292200 5.396800

C 14.477300 15.269900 5.350900

C 15.551700 15.255300 4.451200

C 15.699900 16.366501 3.608600

C 14.780700 17.416599 3.643400

C 13.688300 17.364500 4.514300

C 15.435300 13.470900 6.614100

C 16.519699 13.353800 5.737900

C 17.614100 12.585000 6.166100

C 17.611601 11.937400 7.403900

C 16.519300 12.069900 8.266900

C 15.425500 12.855900 7.891900

C 16.399300 13.981300 4.347000

C 17.776501 14.252800 3.719000

C 15.620200 12.974400 3.449300

C 12.691500 12.744200 7.845900

C 14.698500 14.943800 9.506800

C 14.643100 15.236400 10.885600

C 15.226200 16.387100 11.420100

C 15.886300 17.287399 10.580400

C 15.982700 17.001101 9.215900

C 15.412900 15.840800 8.686700

C 12.934200 11.462900 7.304600

C 11.971000 10.824800 6.519200

C 10.747000 11.444500 6.253700

C 10.492300 12.702700 6.800200

C 11.450600 13.338100 7.593800

C 11.642300 18.348000 8.419200

C 12.827400 19.178301 7.907100

P 11.303800 14.558500 10.663200

P 14.044400 13.255500 9.027500

N 12.682100 16.238199 7.731000

O 14.322000 14.225200 6.258600

Pd 12.798800 12.717400 10.932700

H 10.205800 15.786400 8.838800

H 10.846600 17.825199 10.958900

H 11.258100 14.895600 13.083000

H 12.077400 16.263700 12.260300

H 7.029400 14.098200 11.920600

H 6.938800 15.855300 11.623400

H 7.488000 15.258800 13.190800

H 7.113100 14.085600 9.600500

H 6.053700 12.206300 8.399700

H 7.228400 10.016600 8.205000

H 9.502600 9.751300 9.199600

H 10.571100 11.646600 10.373500

H 9.342700 16.530399 14.053300

H 8.491500 17.604601 12.920500

H 10.198700 17.891500 13.305200

H 8.174400 16.040300 8.952500

H 6.116400 17.173500 8.198800

H 5.640500 19.517300 8.900100

H 7.266900 20.714100 10.363500

H 9.330800 19.573500 11.117400

H 12.872300 17.059500 9.595900

H 13.186600 15.404700 7.987100

H 11.685300 17.137400 6.125700

H 11.673700 15.376900 6.048600

H 16.526899 16.405199 2.905000

H 14.905900 18.268299 2.978900

H 12.960300 18.174000 4.518200

H 18.477699 12.476300 5.515100

H 18.463100 11.328700 7.698800

H 16.511200 11.568500 9.233600

H 18.346201 13.321700 3.611200

H 18.366800 14.956600 4.320200

H 17.666500 14.664400 2.708200

H 15.473500 13.395200 2.445600

H 14.633700 12.748300 3.874800

H 16.179701 12.033500 3.360200

H 14.149900 14.529800 11.556200

H 15.168400 16.568800 12.491200

H 16.334999 18.192400 10.985400

H 16.504999 17.683001 8.548100

H 15.529700 15.663200 7.626000

H 13.875900 10.953900 7.495900

H 12.186900 9.838000 6.114400

H 9.998100 10.948000 5.640800

H 9.535200 13.191400 6.630300

H 11.232200 14.318400 7.994400

H 11.185100 18.871401 9.265800

H 10.845000 18.286400 7.664500

H 13.599700 19.268299 8.683700

H 12.506900 20.192101 7.632700

H 13.309400 18.722500 7.034400

C 11.625500 9.826300 12.380700

C 12.547300 11.036700 12.447500

C 13.917600 11.061100 12.045000

C 14.599300 10.004200 11.288500

C 12.006900 12.246600 12.943000

C 15.917900 9.648000 11.636900

C 16.593700 8.642900 10.943000

C 15.970100 7.982100 9.879000

C 14.668300 8.341200 9.508200

C 13.991500 9.343400 10.201300

C 10.789600 7.874200 18.558901

C 10.161600 8.511500 16.208200

C 11.544700 8.459100 15.857100

C 12.563200 8.154600 16.814501

C 13.154400 7.533100 19.123400

C 8.466700 8.256400 18.018400

C 12.019100 8.584800 14.433300

C 12.000100 7.258200 13.687500

C 10.778600 6.646200 13.358200

C 10.768300 5.451600 12.636300

C 11.972700 4.854100 12.236000

C 13.189000 5.457100 12.567400

C 13.201100 6.656600 13.289500

N 12.125600 7.896400 18.149500

N 9.867600 8.248300 17.594200

O 11.196800 9.556500 13.713000

O 10.426700 7.556000 19.702900

O 13.793600 8.076200 16.579100

O 9.201900 8.759600 15.447700

H 14.576500 11.725300 12.612900

H 10.717000 10.053000 11.805500

H 12.120000 8.960400 11.920100

H 12.641700 12.949700 13.486500

H 10.944500 12.278900 13.185500

H 16.400700 10.151600 12.472900

H 17.605600 8.371800 11.237100

H 16.497299 7.200200 9.336900

H 14.182900 7.842900 8.671700

H 12.998500 9.650600 9.878300

H 12.656500 7.377800 20.084000

H 13.898800 8.334500 19.193300

H 13.671800 6.619300 18.806801

H 8.338500 8.925200 18.876801

H 8.149900 7.250500 18.319401

H 7.881800 8.599500 17.160400

H 9.855300 7.131000 13.675800

H 9.819700 4.982800 12.382400

H 11.959200 3.924700 11.670600

H 14.127600 5.001900 12.258100

H 14.143800 7.133700 13.553800

H 13.065100 8.933400 14.475200

Int 8-2’ -714.1950979 a.u.

C 9.845300 16.285900 11.959800

C 10.911200 16.221399 9.608300

C 10.122200 17.106199 10.623100

C 11.220800 15.648700 12.225900

C 8.933200 15.109600 11.609700

C 9.567800 14.163000 10.862000

C 8.980800 13.008800 10.159600

C 7.499300 15.096000 12.042000

C 7.744700 13.113200 9.487200

C 7.198800 12.017000 8.814600

C 7.872500 10.791300 8.796500

C 9.106900 10.676800 9.443600

C 9.654000 11.774300 10.104700

C 9.365600 17.184401 13.098900

C 8.877400 17.764400 10.059000

C 7.988800 17.089899 9.206700

C 6.820200 17.705601 8.748900

C 6.517700 19.016399 9.130000

C 7.396900 19.705601 9.970700

C 8.563900 19.084299 10.425600

C 12.058300 17.005100 8.857600

C 12.273500 16.275101 6.341200

C 13.477900 16.305401 5.396100

C 14.453900 15.287100 5.344300

C 15.527000 15.284700 4.441700

C 15.665200 16.398001 3.600200

C 14.738000 17.440300 3.637800

C 13.649000 17.378201 4.511800

C 15.447500 13.513000 6.609000

C 16.529699 13.404900 5.729900

C 17.640200 12.665100 6.165700

C 17.656900 12.044600 7.417200

C 16.568800 12.176100 8.284800

C 15.454900 12.926000 7.898000

C 16.392500 14.022900 4.336800

C 17.763300 14.313900 3.703300

C 15.624400 13.003200 3.444900

C 12.713200 12.815000 7.866100

C 14.740200 15.048600 9.464400

C 14.676500 15.383400 10.833000

C 15.268100 16.544399 11.336500

C 15.943900 17.412399 10.475600

C 16.046400 17.084400 9.120900

C 15.468200 15.914600 8.623200

C 12.955500 11.528900 7.338700

C 11.977400 10.867200 6.591800

C 10.741200 11.470100 6.347100

C 10.489300 12.736600 6.877100

C 11.460100 13.392500 7.637300

C 11.615900 18.416300 8.412000

C 12.787700 19.257299 7.889000

P 11.326900 14.651000 10.700100

P 14.075300 13.352500 9.021500

N 12.684400 16.317400 7.736600

O 14.318200 14.236800 6.252000

Pd 12.857000 12.836600 10.939800

H 10.222800 15.829100 8.850500

H 10.801700 17.907400 10.952000

H 11.222800 15.006900 13.113800

H 12.029900 16.387400 12.299700

H 7.021800 14.132200 11.832100

H 6.926000 15.888900 11.536100

H 7.423500 15.294000 13.121300

H 7.217800 14.064500 9.481100

H 6.245200 12.122100 8.301200

H 7.443200 9.937300 8.276900

H 9.648700 9.733200 9.428300

H 10.629300 11.687700 10.582900

H 9.248000 16.610701 14.027800

H 8.403600 17.659700 12.866600

H 10.095300 17.983101 13.289700

H 8.205500 16.068501 8.900400

H 6.147500 17.160700 8.089800

H 5.611000 19.497999 8.770300

H 7.178200 20.728800 10.269200

H 9.245500 19.631300 11.075900

H 12.866500 17.151800 9.593400

H 13.222300 15.508800 8.005200

H 11.654700 17.152201 6.118500

H 11.660000 15.389100 6.096400

H 16.491301 16.444401 2.896300

H 14.854600 18.293800 2.974000

H 12.916100 18.183201 4.520200

H 18.502701 12.560700 5.512500

H 18.522200 11.459500 7.719800

H 16.577200 11.697100 9.261000

H 18.348101 13.391700 3.599800

H 18.343399 15.030600 4.299100

H 17.644501 14.717400 2.690300

H 15.467700 13.419700 2.440900

H 14.642900 12.764000 3.874800

H 16.196501 12.070000 3.354900

H 14.171500 14.702400 11.520200

H 15.204100 16.760099 12.401000

H 16.399599 18.324301 10.856600

H 16.580000 17.740200 8.436100

H 15.591900 15.702900 7.569100

H 13.903400 11.028000 7.517400

H 12.193400 9.874500 6.201600

H 9.980900 10.956300 5.763300

H 9.523900 13.213700 6.722800

H 11.243200 14.376200 8.030100

H 11.154100 18.940599 9.255500

H 10.817500 18.337700 7.660000

H 13.563200 19.361601 8.660600

H 12.453300 20.265100 7.609500

H 13.269800 18.799801 7.017000

C 11.690000 10.006300 12.654200

C 12.663500 11.161100 12.501600

C 13.947300 11.108500 11.853800

C 14.394600 10.060000 10.927400

C 12.283800 12.413300 13.029700

C 15.758500 9.708700 10.887000

C 16.229799 8.759700 9.980200

C 15.348100 8.139700 9.089100

C 13.990600 8.482000 9.112000

C 13.520200 9.434400 10.014700

C 10.703900 7.818100 18.647100

C 10.193000 8.229700 16.227200

C 11.602900 8.261900 15.953200

C 12.570200 8.097600 17.000099

C 13.032500 7.665900 19.375099

C 8.393300 7.999100 17.941200

C 11.948400 8.396000 14.507200

C 13.359100 8.042100 14.087900

C 14.436600 8.897200 14.372900

C 15.734800 8.552600 14.001800

C 15.980200 7.352200 13.322400

C 14.914200 6.507400 13.008400

C 13.610900 6.856200 13.388100

N 12.048900 7.894900 18.315399

N 9.815600 8.062800 17.605600

O 11.640400 9.810400 14.071800

O 10.318400 7.555900 19.795601

O 13.814900 8.082600 16.915701

O 9.291200 8.330700 15.368000

H 14.751600 11.671400 12.336900

H 10.683900 10.258000 12.281800

H 12.053100 9.112700 12.130200

H 13.032800 13.095400 13.440300

H 11.273600 12.523700 13.424500

H 16.444901 10.176100 11.590400

H 17.285801 8.497900 9.973600

H 15.714300 7.400900 8.379600

H 13.298400 8.017600 8.412600

H 12.475000 9.735600 9.980700

H 12.482600 7.542600 20.311001

H 13.720800 8.516600 19.432301

H 13.625100 6.770000 19.154900

H 8.157400 8.732600 18.720600

H 8.129000 7.003400 18.317801

H 7.844600 8.215700 17.020000

H 14.240500 9.817400 14.916500

H 16.562500 9.211900 14.256200

H 16.994301 7.082800 13.034700

H 15.091800 5.580800 12.467500

H 12.779000 6.193800 13.152200

H 11.232200 7.777200 13.943700

TS1' -714.2134295 a.u.

C 9.754000 16.964001 11.669000

C 10.825000 16.556999 9.357000

C 10.154000 17.620001 10.276000

C 11.012000 16.141001 11.992000

C 8.664000 15.929000 11.396000

C 9.144000 14.852000 10.715000

C 8.394000 13.744000 10.100000

C 7.247000 16.181999 11.809000

C 7.193000 13.977000 9.395000

C 6.480000 12.924000 8.815000

C 6.955000 11.613000 8.916000

C 8.158000 11.367000 9.586000

C 8.867000 12.418000 10.168000

C 9.430000 18.034000 12.711000

C 9.029000 18.402000 9.639000

C 8.034000 17.789000 8.859000

C 6.972000 18.531000 8.335000

C 6.892000 19.908001 8.566000

C 7.883000 20.535000 9.327000

C 8.937000 19.785999 9.858000

C 12.123000 17.121000 8.658000

C 12.208000 16.254000 6.225000

C 13.331000 16.156000 5.194000

C 14.200000 15.050000 5.140000

C 15.143000 14.852000 4.121000

C 15.295000 15.878000 3.178000

C 14.488000 17.017000 3.226000

C 13.499000 17.135000 4.209000

C 15.122000 13.260000 6.416000

C 16.079000 12.975000 5.437000

C 17.160000 12.156000 5.797000

C 17.301001 11.687000 7.103000

C 16.341999 12.016000 8.063000

C 15.208000 12.753000 7.725000

C 15.811000 13.477000 4.023000

C 17.089001 13.490000 3.172000

C 14.777000 12.495000 3.398000

C 12.468000 12.736000 7.838000

C 14.682000 14.851000 9.345000

C 14.555000 15.308000 10.672000

C 15.223000 16.452000 11.115000

C 16.045000 17.167999 10.239000

C 16.202999 16.715000 8.927000

C 15.539000 15.568000 8.490000

C 12.547000 11.412000 7.351000

C 11.495000 10.843000 6.624000

C 10.332000 11.572000 6.362000

C 10.237000 12.882000 6.838000

C 11.286000 13.450000 7.565000

C 11.933000 18.563999 8.132000

C 13.227000 19.143000 7.548000

P 10.965000 15.026000 10.542000

P 13.921000 13.184000 8.971000

N 12.696000 16.313000 7.594000

O 14.060000 14.116000 6.155000

Pd 12.181000 13.173000 10.456000

H 10.130000 16.240000 8.569000

H 10.937000 18.339001 10.559000

H 10.928000 15.568000 12.923000

H 11.931000 16.743000 12.010000

H 6.621000 15.292000 11.677000

H 6.809000 17.004000 11.221000

H 7.200000 16.493000 12.863000

H 6.821000 14.994000 9.297000

H 5.553000 13.130000 8.284000

H 6.395000 10.791000 8.474000

H 8.548000 10.354000 9.660000

H 9.810000 12.218000 10.682000

H 9.235000 17.587000 13.695000

H 8.555000 18.629999 12.423000

H 10.280000 18.722000 12.818000

H 8.087000 16.719999 8.667000

H 6.210000 18.033001 7.739000

H 6.072000 20.489000 8.150000

H 7.841000 21.608000 9.506000

H 9.708000 20.278000 10.449000

H 12.904000 17.169001 9.434000

H 13.117000 15.451000 7.912000

H 11.636000 17.163000 6.008000

H 11.521000 15.406000 6.048000

H 16.032000 15.776000 2.386000

H 14.608000 17.798000 2.480000

H 12.841000 18.003000 4.217000

H 17.902000 11.886000 5.050000

H 18.145000 11.057000 7.375000

H 16.466999 11.696000 9.090000

H 17.507000 12.479000 3.097000

H 17.856001 14.149000 3.601000

H 16.877001 13.817000 2.147000

H 14.510000 12.803000 2.380000

H 13.861000 12.461000 4.003000

H 15.199000 11.481000 3.368000

H 13.924000 14.736000 11.355000

H 15.109000 16.774000 12.147000

H 16.570000 18.059000 10.577000

H 16.844000 17.250000 8.230000

H 15.702000 15.245000 7.469000

H 13.438000 10.817000 7.543000

H 11.588000 9.818000 6.271000

H 9.511000 11.128000 5.805000

H 9.336000 13.468000 6.663000

H 11.190000 14.470000 7.913000

H 11.587000 19.214001 8.943000

H 11.120000 18.587000 7.391000

H 14.029000 19.132000 8.299000

H 13.082000 20.179001 7.216000

H 13.592000 18.556000 6.697000

C 9.428000 10.361000 17.077000

C 10.555000 9.914000 14.909000

C 11.904000 9.880000 15.602000

C 11.900000 10.267000 17.073999

C 10.655000 10.464000 19.180000

C 8.110000 9.997000 15.038000

C 12.967000 10.743000 14.895000

C 12.444000 8.362000 15.564000

C 13.546000 8.623000 13.440000

C 11.478000 7.443000 16.264000

C 11.634000 7.134000 17.620001

C 10.652000 6.391000 18.285000

C 9.511000 5.964000 17.599001

C 9.366000 6.256000 16.238001

C 10.351000 6.983000 15.570000

C 13.721000 10.092000 13.764000

C 14.651000 10.813000 13.118000

C 15.551000 10.208000 12.115000

C 16.737000 9.568000 12.514000

C 17.579000 8.983000 11.565000

C 17.224001 9.000000 10.212000

C 16.017000 9.582000 9.821000

C 15.195000 10.204000 10.763000

N 10.676000 10.389000 17.711000

N 9.419000 10.094000 15.704000

O 8.404000 10.578000 17.712999

O 12.950000 10.410000 17.702999

O 10.429000 9.741000 13.700000

O 12.585000 7.908000 14.237000

H 10.262000 9.518000 19.573000

H 10.011000 11.285000 19.504000

H 11.685000 10.612000 19.510000

H 8.158000 10.517000 14.077000

H 7.362000 10.442000 15.697000

H 7.869000 8.941000 14.862000

H 12.534000 11.697000 14.567000

H 13.703000 10.967000 15.679000

H 13.413000 8.405000 16.090000

H 13.195000 8.513000 12.403000

H 14.517000 8.106000 13.512000

H 12.516000 7.486000 18.153999

H 10.779000 6.145000 19.337000

H 8.740000 5.399000 18.117001

H 8.483000 5.926000 15.695000

H 10.257000 7.218000 14.513000

H 16.987000 9.530000 13.572000

H 18.504999 8.509000 11.880000

H 17.889000 8.573000 9.466000

H 15.736000 9.604000 8.773000

H 14.292000 10.729000 10.458000

H 14.772000 11.871000 13.357000

TS2' -647.7171406 a.u.

C 10.229000 16.691000 11.939000

C 10.630000 16.594999 9.396000

C 10.093000 17.480000 10.563000

C 11.661000 16.139999 11.836000

C 9.337000 15.451000 11.873000

C 9.806000 14.517000 10.998000

C 9.114000 13.308000 10.518000

C 8.088000 15.359000 12.697000

C 7.727000 13.329000 10.254000

C 7.061000 12.191000 9.794000

C 7.767000 11.004000 9.572000

C 9.147000 10.975000 9.797000

C 9.811000 12.110000 10.262000

C 10.009000 17.603001 13.145000

C 8.714000 18.066000 10.341000

C 7.655000 17.316999 9.805000

C 6.380000 17.872999 9.656000

C 6.139000 19.198000 10.035000

C 7.186000 19.959999 10.563000

C 8.457000 19.396999 10.713000

C 11.502000 17.400000 8.356000

C 11.031000 16.562000 5.933000

C 11.875000 16.552000 4.659000

C 12.845000 15.565000 4.384000

C 13.570000 15.509000 3.185000

C 13.365000 16.541000 2.257000

C 12.441000 17.556000 2.507000

C 11.693000 17.544001 3.688000

C 14.272000 13.922000 5.386000

C 15.020000 13.766000 4.214000

C 16.233999 13.066000 4.315000

C 16.665001 12.525000 5.528000

C 15.901000 12.694000 6.687000

C 14.703000 13.412000 6.633000

C 14.424000 14.269000 2.899000

C 15.512000 14.548000 1.848000

C 13.471000 13.160000 2.363000

C 12.103000 13.188000 7.396000

C 14.362000 15.591000 8.248000

C 14.642000 16.007999 9.566000

C 15.272000 17.226000 9.833000

C 15.645000 18.066000 8.781000

C 15.409000 17.655001 7.466000

C 14.789000 16.431999 7.201000

C 12.244000 11.886000 6.871000

C 11.132000 11.170000 6.419000

C 9.854000 11.732000 6.470000

C 9.702000 13.011000 7.006000

C 10.811000 13.719000 7.473000

C 10.888000 18.774000 8.009000

C 11.812000 19.627001 7.131000

P 11.433000 15.088000 10.356000

P 13.712000 13.831000 8.114000

N 11.861000 16.708000 7.120000

O 13.061000 14.610000 5.374000

Pd 13.089000 13.364000 10.296000

H 9.785000 16.152000 8.855000

H 10.793000 18.320999 10.684000

H 11.946000 15.526000 12.697000

H 12.414000 16.923000 11.675000

H 7.660000 14.350000 12.668000

H 7.323000 16.070999 12.349000

H 8.299000 15.618000 13.745000

H 7.172000 14.253000 10.394000

H 5.991000 12.235000 9.600000

H 7.252000 10.115000 9.214000

H 9.710000 10.067000 9.587000

H 10.891000 12.084000 10.413000

H 10.181000 17.059999 14.085000

H 8.992000 18.014000 13.169000

H 10.710000 18.448999 13.117000

H 7.825000 16.285000 9.503000

H 5.576000 17.271000 9.238000

H 5.150000 19.632999 9.912000

H 7.016000 20.993999 10.854000

H 9.268000 19.999001 11.120000

H 12.465000 17.606001 8.851000

H 12.457000 15.907000 7.271000

H 10.343000 17.413000 5.867000

H 10.403000 15.652000 5.940000

H 13.920000 16.545000 1.323000

H 12.293000 18.348000 1.776000

H 10.955000 18.323000 3.871000

H 16.844999 12.928000 3.427000

H 17.597000 11.967000 5.571000

H 16.222000 12.265000 7.634000

H 16.084000 13.638000 1.629000

H 16.209999 15.326000 2.184000

H 15.064000 14.867000 0.899000

H 12.986000 13.489000 1.435000

H 12.688000 12.927000 3.097000

H 14.036000 12.239000 2.161000

H 14.378000 15.342000 10.391000

H 15.475000 17.507000 10.864000

H 16.129999 19.018999 8.981000

H 15.705000 18.288000 6.633000

H 14.643000 16.157000 6.164000

H 13.222000 11.415000 6.824000

H 11.278000 10.165000 6.026000

H 8.990000 11.175000 6.116000

H 8.713000 13.458000 7.088000

H 10.663000 14.709000 7.881000

H 10.675000 19.320999 8.934000

H 9.906000 18.636000 7.533000

H 12.784000 19.774000 7.622000

H 11.374000 20.617001 6.946000

H 12.018000 19.153999 6.163000

C 12.443000 10.273000 12.290000

C 13.198000 11.520000 12.111000

C 14.251000 11.739000 11.063000

C 14.619000 10.718000 10.059000

C 12.616000 12.877000 12.304000

C 15.980000 10.457000 9.789000

C 16.365000 9.519000 8.828000

C 15.398000 8.809000 8.109000

C 14.043000 9.052000 8.364000

C 13.660000 9.994000 9.320000

O 13.432000 10.625000 13.280000

H 15.132000 12.232000 11.495000

H 11.408000 10.342000 12.633000

H 12.673000 9.389000 11.692000

H 13.242000 13.522000 12.932000

H 11.562000 12.934000 12.582000

H 16.738001 10.999000 10.352000

H 17.423000 9.338000 8.645000

H 15.695000 8.078000 7.360000

H 13.278000 8.512000 7.810000

H 12.603000 10.202000 9.475000

C 9.195900 7.431500 14.752000

C 9.216200 7.418800 13.212800

C 9.744800 6.060100 12.736000

C 7.806400 7.670400 12.656800

C 9.806600 11.638200 14.719200

O 10.143300 8.396100 12.663300

O 10.420000 11.102000 13.880300

O 9.183800 12.171300 15.558700

H 8.561300 6.613300 15.121900

H 8.786800 8.367400 15.149500

H 10.207800 7.296300 15.155200

H 10.738400 5.864000 13.159800

H 9.824500 6.035200 11.642100

H 9.067800 5.256200 13.054400

H 7.090400 6.934100 13.047500

H 7.812900 7.600200 11.561500

H 7.450000 8.672800 12.929600

H 9.952800 9.291400 13.006800

TS3' -714.1973712 a.u.

C 9.664000 16.528999 11.547000

C 10.788000 16.416000 9.226000

C 10.002000 17.334000 10.215000

C 11.006000 15.839000 11.845000

C 8.715000 15.390000 11.176000

C 9.326000 14.423000 10.435000

C 8.699000 13.308000 9.702000

C 7.275000 15.431000 11.588000

C 7.467000 13.483000 9.034000

C 6.874000 12.434000 8.329000

C 7.497000 11.183000 8.269000

C 8.727000 10.999000 8.908000

C 9.321000 12.049000 9.606000

C 9.195000 17.450001 12.673000

C 8.794000 18.031000 9.620000

C 7.903000 17.382000 8.751000

C 6.764000 18.033001 8.268000

C 6.494000 19.354000 8.641000

C 7.377000 20.018000 9.497000

C 8.513000 19.361000 9.978000

C 11.998000 17.150999 8.533000

C 12.325000 16.365999 6.058000

C 13.565000 16.341000 5.161000

C 14.515000 15.299000 5.191000

C 15.605000 15.217000 4.313000

C 15.797000 16.285999 3.425000

C 14.904000 17.358999 3.393000

C 13.790000 17.368000 4.237000

C 15.400000 13.522000 6.534000

C 16.490999 13.332000 5.680000

C 17.541000 12.529000 6.156000

C 17.487000 11.922000 7.413000

C 16.382999 12.121000 8.248000

C 15.333000 12.939000 7.822000

C 16.424000 13.922000 4.270000

C 17.825001 14.144000 3.673000

C 15.646000 12.910000 3.376000

C 12.604000 12.922000 7.621000

C 14.606000 15.116000 9.305000

C 14.481000 15.457000 10.668000

C 15.081000 16.603001 11.197000

C 15.827000 17.445000 10.371000

C 15.992000 17.107000 9.025000

C 15.404000 15.954000 8.501000

C 12.844000 11.641000 7.080000

C 11.896000 11.018000 6.263000

C 10.688000 11.653000 5.964000

C 10.435000 12.913000 6.508000

C 11.378000 13.531000 7.332000

C 11.619000 18.563000 8.031000

C 12.840000 19.358999 7.550000

P 11.112000 14.832000 10.326000

P 13.914000 13.428000 8.862000

N 12.676000 16.423000 7.469000

O 14.324000 14.317000 6.159000

Pd 12.569000 12.967000 10.674000

H 10.116000 16.056000 8.438000

H 10.700000 18.114000 10.557000

H 10.965000 15.199000 12.734000

H 11.842000 16.545000 11.934000

H 6.766000 14.484000 11.377000

H 6.736000 16.240000 11.070000

H 7.193000 15.639000 12.665000

H 6.981000 14.456000 9.055000

H 5.925000 12.596000 7.821000

H 7.033000 10.364000 7.723000

H 9.234000 10.037000 8.857000

H 10.294000 11.901000 10.071000

H 9.039000 16.884001 13.602000

H 8.257000 17.959999 12.421000

H 9.951000 18.221001 12.876000

H 8.095000 16.354000 8.450000

H 6.087000 17.507000 7.597000

H 5.609000 19.861000 8.263000

H 7.183000 21.048000 9.790000

H 9.198000 19.886999 10.642000

H 12.763000 17.295000 9.315000

H 13.157000 15.596000 7.785000

H 11.742000 17.254999 5.793000

H 11.697000 15.493000 5.801000

H 16.639999 16.274000 2.739000

H 15.066000 18.177999 2.696000

H 13.079000 18.191000 4.188000

H 18.412001 12.362000 5.526000

H 18.308001 11.290000 7.744000

H 16.329000 11.644000 9.225000

H 18.378000 13.198000 3.615000

H 18.412001 14.855000 4.269000

H 17.754000 14.522000 2.646000

H 15.537000 13.309000 2.358000

H 14.643000 12.718000 3.780000

H 16.184999 11.955000 3.325000

H 13.921000 14.792000 11.328000

H 14.967000 16.825001 12.256000

H 16.291000 18.344000 10.773000

H 16.583000 17.742001 8.368000

H 15.577000 15.735000 7.456000

H 13.772000 11.117000 7.296000

H 12.112000 10.030000 5.861000

H 9.951000 11.169000 5.327000

H 9.489000 13.415000 6.314000

H 11.158000 14.510000 7.734000

H 11.127000 19.118000 8.837000

H 10.860000 18.486000 7.240000

H 13.579000 19.458000 8.358000

H 12.550000 20.370001 7.233000

H 13.351000 18.868999 6.714000

C 11.188000 9.949000 12.057000

C 12.020000 11.175000 12.126000

C 13.422000 11.272000 11.763000

C 14.146000 10.226000 11.016000

C 11.471000 12.464000 12.451000

C 15.430000 9.829000 11.441000

C 16.145000 8.846000 10.751000

C 15.594000 8.240000 9.618000

C 14.320000 8.626000 9.182000

C 13.607000 9.607000 9.870000

C 10.636000 9.974000 18.121000

C 10.129000 8.669000 16.055000

C 11.545000 8.668000 15.737000

C 12.502000 9.388000 16.580999

C 12.919000 10.511000 18.752001

C 8.340000 9.314000 17.629999

C 12.108000 8.232000 14.525000

C 11.838000 7.135000 13.594000

C 10.668000 6.349000 13.536000

C 10.569000 5.302000 12.617000

C 11.619000 5.017000 11.734000

C 12.789000 5.783000 11.783000

C 12.894000 6.824000 12.704000

N 11.989000 9.906000 17.794001

N 9.764000 9.293000 17.278000

O 11.456000 9.889000 13.443000

O 10.244000 10.595000 19.112000

O 13.708000 9.547000 16.320999

O 9.206000 8.223000 15.356000

H 14.041000 11.801000 12.497000

H 10.126000 10.095000 11.795000

H 11.609000 9.116000 11.471000

H 11.999000 13.048000 13.210000

H 10.385000 12.569000 12.479000

H 15.857000 10.290000 12.331000

H 17.132999 8.553000 11.101000

H 16.152000 7.479000 9.076000

H 13.885000 8.167000 8.296000

H 12.635000 9.931000 9.501000

H 12.731000 11.585000 18.858999

H 13.926000 10.339000 18.362000

H 12.791000 10.036000 19.731001

H 7.810000 10.111000 17.093000

H 8.262000 9.491000 18.705000

H 7.900000 8.354000 17.347000

H 9.848000 6.591000 14.207000

H 9.661000 4.705000 12.584000

H 11.526000 4.207000 11.014000

H 13.618000 5.572000 11.111000

H 13.809000 7.414000 12.748000

H 13.154000 8.540000 14.480000

TS3-2' -714.1950979 a.u.

C 9.842000 16.541000 11.659000

C 10.889000 16.421000 9.303000

C 10.149000 17.344000 10.318000

C 11.186000 15.834000 11.915000

C 8.868000 15.414000 11.316000

C 9.446000 14.436000 10.562000

C 8.787000 13.328000 9.849000

C 7.440000 15.473000 11.761000

C 7.553000 13.520000 9.193000

C 6.934000 12.474000 8.504000

C 7.533000 11.211000 8.451000

C 8.764000 11.009000 9.081000

C 9.383000 12.055000 9.762000

C 9.418000 17.465000 12.799000

C 8.932000 18.059000 9.762000

C 8.006000 17.427000 8.917000

C 6.859000 18.094000 8.474000

C 6.619000 19.416000 8.862000

C 7.539000 20.063999 9.691000

C 8.681000 19.391001 10.133000

C 12.076000 17.145000 8.562000

C 12.268000 16.361000 6.071000

C 13.468000 16.311001 5.124000

C 14.399000 15.251000 5.103000

C 15.453000 15.170000 4.182000

C 15.625000 16.245001 3.298000

C 14.749000 17.330999 3.312000

C 13.672000 17.346001 4.202000

C 15.326000 13.458000 6.396000

C 16.385000 13.276000 5.500000

C 17.459999 12.487000 5.938000

C 17.459999 11.887000 7.200000

C 16.388000 12.079000 8.077000

C 15.312000 12.882000 7.689000

C 16.259001 13.869000 4.095000

C 17.634001 14.083000 3.439000

C 15.436000 12.869000 3.229000

C 12.570000 12.910000 7.605000

C 14.675000 15.064000 9.203000

C 14.610000 15.417000 10.566000

C 15.259000 16.551001 11.065000

C 15.990000 17.372000 10.204000

C 16.089001 17.025999 8.853000

C 15.455000 15.884000 8.360000

C 12.768000 11.629000 7.048000

C 11.774000 11.023000 6.275000

C 10.563000 11.677000 6.035000

C 10.353000 12.938000 6.594000

C 11.342000 13.539000 7.376000

C 11.698000 18.566999 8.086000

C 12.909000 19.346001 7.553000

P 11.226000 14.828000 10.393000

P 13.938000 13.391000 8.783000

N 12.685000 16.412001 7.463000

O 14.234000 14.246000 6.062000

Pd 12.641000 12.910000 10.648000

H 10.185000 16.066000 8.541000

H 10.868000 18.114000 10.639000

H 11.164000 15.199000 12.808000

H 12.033000 16.531000 11.976000

H 6.913000 14.535000 11.557000

H 6.902000 16.294001 11.260000

H 7.385000 15.676000 12.841000

H 7.084000 14.501000 9.212000

H 5.983000 12.648000 8.005000

H 7.048000 10.396000 7.919000

H 9.250000 10.036000 9.038000

H 10.356000 11.895000 10.225000

H 9.281000 16.900000 13.731000

H 8.478000 17.985001 12.573000

H 10.187000 18.228001 12.980000

H 8.175000 16.398001 8.604000

H 6.156000 17.580000 7.822000

H 5.729000 19.937000 8.515000

H 7.369000 21.096001 9.993000

H 9.393000 19.906000 10.776000

H 12.879000 17.271000 9.307000

H 13.189000 15.588000 7.751000

H 11.694000 17.263000 5.832000

H 11.610000 15.503000 5.843000

H 16.440001 16.229000 2.579000

H 14.894000 18.156000 2.618000

H 12.974000 18.181000 4.193000

H 18.308001 12.327000 5.277000

H 18.301001 11.267000 7.503000

H 16.381001 11.609000 9.057000

H 18.176001 13.133000 3.351000

H 18.254000 14.787000 4.010000

H 17.521000 14.466000 2.418000

H 15.289000 13.276000 2.220000

H 14.449000 12.683000 3.672000

H 15.964000 11.909000 3.149000

H 14.063000 14.770000 11.253000

H 15.196000 16.780001 12.126000

H 16.492001 18.260000 10.582000

H 16.665001 17.646000 8.169000

H 15.580000 15.657000 7.310000

H 13.697000 11.092000 7.218000

H 11.957000 10.034000 5.860000

H 9.790000 11.206000 5.432000

H 9.407000 13.455000 6.444000

H 11.159000 14.523000 7.786000

H 11.254000 19.127001 8.916000

H 10.902000 18.509001 7.330000

H 13.685000 19.431000 8.326000

H 12.619000 20.362000 7.254000

H 13.373000 18.853001 6.691000

C 11.347000 9.948000 12.228000

C 12.253000 11.166000 12.142000

C 13.603000 11.172000 11.646000

C 14.166000 10.127000 10.777000

C 11.775000 12.422000 12.592000

C 15.508000 9.734000 10.945000

C 16.084999 8.783000 10.101000

C 15.336000 8.212000 9.067000

C 14.001000 8.594000 8.889000

C 13.422000 9.541000 9.733000

C 9.885000 9.858000 18.153999

C 9.687000 8.728000 15.902000

C 11.111000 8.812000 15.765000

C 11.909000 9.456000 16.768999

C 12.098000 10.409000 19.028999

C 7.684000 9.406000 17.221001

C 11.840000 8.701000 14.468000

C 12.136000 7.415000 13.706000

C 13.442000 7.346000 13.175000

C 13.901000 6.245000 12.452000

C 13.053000 5.152000 12.257000

C 11.758000 5.187000 12.792000

C 11.293000 6.305000 13.504000

N 11.266000 9.820000 17.980000

N 9.142000 9.338000 17.100000

O 11.184000 9.800000 13.635000

O 9.370000 10.329000 19.180000

O 13.129000 9.722000 16.671000

O 8.868000 8.240000 15.098000

H 14.334000 11.739000 12.231000

H 10.347000 10.143000 11.808000

H 11.777000 9.063000 11.743000

H 12.440000 13.094000 13.142000

H 10.713000 12.528000 12.817000

H 16.091999 10.177000 11.750000

H 17.122999 8.492000 10.249000

H 15.789000 7.481000 8.401000

H 13.413000 8.164000 8.080000

H 12.399000 9.868000 9.555000

H 11.509000 10.434000 19.948999

H 12.406000 11.429000 18.763000

H 13.001000 9.802000 19.143000

H 7.434000 10.241000 17.882000

H 7.273000 8.481000 17.648001

H 7.260000 9.543000 16.222000

H 14.114000 8.184000 13.355000

H 14.914000 6.244000 12.055000

H 13.402000 4.281000 11.703000

H 11.108000 4.325000 12.650000

H 10.283000 6.319000 13.914000

H 12.837000 9.085000 14.727000

TS4' -714.1974791 a.u.

C 9.993000 15.778000 12.211000

C 10.950000 15.969000 9.811000

C 10.198000 16.731001 10.950000

C 11.374000 15.119000 12.327000

C 9.056000 14.644000 11.794000

C 9.631000 13.805000 10.884000

C 8.978000 12.775000 10.058000

C 7.668000 14.556000 12.350000

C 7.648000 12.936000 9.607000

C 7.029000 11.959000 8.825000

C 7.718000 10.793000 8.472000

C 9.045000 10.634000 8.880000

C 9.666000 11.619000 9.646000

C 9.593000 16.555000 13.463000

C 8.921000 17.424000 10.524000

C 7.981000 16.819000 9.674000

C 6.778000 17.459000 9.361000

C 6.496000 18.725000 9.883000

C 7.428000 19.346001 10.720000

C 8.625000 18.698999 11.034000

C 12.084000 16.844999 9.152000

C 12.267000 16.385000 6.591000

C 13.427000 16.518000 5.604000

C 14.416000 15.528000 5.436000

C 15.451000 15.620000 4.495000

C 15.536000 16.798000 3.738000

C 14.591000 17.813000 3.892000

C 13.541000 17.660000 4.801000

C 15.479000 13.669000 6.512000

C 16.532000 13.665000 5.589000

C 17.677999 12.923000 5.918000

C 17.756001 12.190000 7.104000

C 16.691000 12.202000 8.010000

C 15.544000 12.954000 7.737000

C 16.330000 14.389000 4.255000

C 17.667000 14.754000 3.587000

C 15.543000 13.429000 3.315000

C 12.834000 12.737000 7.744000

C 14.783000 14.837000 9.569000

C 14.726000 15.018000 10.967000

C 15.272000 16.143999 11.587000

C 15.897000 17.128000 10.818000

C 16.000000 16.950001 9.436000

C 15.466000 15.817000 8.820000

C 13.112000 11.528000 7.069000

C 12.171000 10.955000 6.209000

C 10.937000 11.574000 5.995000

C 10.647000 12.759000 6.672000

C 11.581000 13.324000 7.545000

C 11.611000 18.285000 8.852000

C 12.761000 19.194000 8.398000

P 11.391000 14.290000 10.702000

P 14.183000 13.174000 8.959000

N 12.747000 16.306999 7.967000

O 14.325000 14.404000 6.256000

Pd 12.914000 12.476000 10.835000

H 10.242000 15.675000 9.025000

H 10.886000 17.502001 11.326000

H 11.428000 14.387000 13.140000

H 12.193000 15.845000 12.426000

H 7.199000 13.594000 12.114000

H 7.028000 15.365000 11.963000

H 7.692000 14.668000 13.443000

H 7.104000 13.843000 9.855000

H 6.005000 12.110000 8.489000

H 7.231000 10.028000 7.872000

H 9.605000 9.747000 8.591000

H 10.717000 11.513000 9.916000

H 9.539000 15.888000 14.334000

H 8.620000 17.049000 13.348000

H 10.340000 17.330000 13.684000

H 8.183000 15.833000 9.260000

H 6.064000 16.969000 8.703000

H 5.562000 19.226000 9.635000

H 7.224000 20.334000 11.127000

H 9.347000 19.188000 11.687000

H 12.883000 16.930000 9.907000

H 13.224000 15.435000 8.145000

H 11.633000 17.271000 6.476000

H 11.651000 15.519000 6.290000

H 16.332001 16.916000 3.009000

H 14.665000 18.718000 3.293000

H 12.794000 18.445999 4.903000

H 18.518000 12.905000 5.228000

H 18.648001 11.608000 7.323000

H 16.745001 11.631000 8.936000

H 18.261999 13.855000 3.386000

H 18.263000 15.435000 4.210000

H 17.496000 15.232000 2.615000

H 15.337000 13.923000 2.355000

H 14.584000 13.136000 3.763000

H 16.127001 12.518000 3.125000

H 14.263000 14.242000 11.581000

H 15.213000 16.242001 12.669000

H 16.316999 18.014999 11.290000

H 16.495001 17.698999 8.821000

H 15.584000 15.723000 7.748000

H 14.066000 11.027000 7.210000

H 12.416000 10.023000 5.702000

H 10.207000 11.131000 5.321000

H 9.680000 13.241000 6.544000

H 11.330000 14.244000 8.056000

H 11.151000 18.715000 9.748000

H 10.803000 18.264999 8.106000

H 13.551000 19.225000 9.161000

H 12.408000 20.219999 8.234000

H 13.230000 18.837999 7.473000

C 12.571000 10.677000 12.154000

C 13.946000 10.693000 11.754000

C 14.582000 9.690000 10.891000

C 15.833000 9.163000 11.267000

C 16.454000 8.180000 10.495000

C 15.843000 7.713000 9.326000

C 14.611000 8.245000 8.928000

C 13.990000 9.227000 9.699000

C 10.495000 8.730000 17.988001

C 12.396000 9.121000 16.422001

C 11.457000 9.181000 15.340000

C 10.053000 9.357000 15.595000

C 8.205000 8.936000 17.216000

C 12.796000 8.660000 18.813999

C 12.109000 11.764000 12.920000

C 11.920000 8.595000 14.019000

C 11.596000 9.564000 11.794000

C 11.877000 7.074000 14.156000

C 13.022000 6.331000 14.471000

C 12.938000 4.941000 14.633000

C 11.711000 4.287000 14.486000

C 10.562000 5.031000 14.185000

C 10.644000 6.413000 14.025000

N 9.637000 9.043000 16.937000

N 11.851000 8.938000 17.728001

O 10.073000 8.341000 19.087000

O 9.195000 9.715000 14.767000

O 13.639000 9.188000 16.309000

O 11.029000 8.954000 12.937000

H 14.628000 11.230000 12.420000

H 16.299999 9.514000 12.186000

H 17.413000 7.775000 10.810000

H 16.326000 6.945000 8.725000

H 14.136000 7.900000 8.012000

H 13.057000 9.673000 9.358000

H 7.956000 7.905000 17.496000

H 7.686000 9.224000 16.297001

H 7.923000 9.596000 18.044001

H 13.158000 7.624000 18.757000

H 12.279000 8.806000 19.766001

H 13.654000 9.331000 18.712000

H 12.808000 12.354000 13.516000

H 11.056000 11.814000 13.199000

H 12.957000 8.908000 13.819000

H 10.731000 9.984000 11.263000

H 12.084000 8.825000 11.142000

H 13.972000 6.845000 14.605000

H 13.832000 4.370000 14.872000

H 11.647000 3.208000 14.605000

H 9.601000 4.532000 14.077000

H 9.759000 7.003000 13.795000
